# Peroxide derivatives as SARS-CoV-2 entry inhibitors

**DOI:** 10.1016/j.virusres.2023.199295

**Published:** 2023-12-12

**Authors:** Ding-qi Zhang, Qin-hai Ma, Meng-chu Yang, Yulia Yu. Belyakova, Zi-feng Yang, Peter S. Radulov, Rui-hong Chen, Li-jun Yang, Jing-yuan Wei, Yu-tong Peng, Wu-yan Zheng, Ivan A. Yaremenko, Alexander O. Terent'ev, Paolo Coghi, Vincent Kam Wai Wong

**Affiliations:** a Dr. Neher's Biophysics Laboratory for Innovative Drug Discovery, State Key Laboratory of Quality Research in Chinese Medicine, Macau University of Science and Technology, Macau, China; b State Key Laboratory of Respiratory Disease, National Clinical Research Center for Respiratory Disease, Guangzhou Institute of Respiratory Health, the First Affiliated Hospital of Guangzhou Medical University, Guangzhou Medical University, Guangzhou, China; c N. D. Zelinsky Institute of Organic Chemistry, Russian Academy of Sciences, Moscow, Russian Federation; d Institute of Translational Medicine, Zhejiang Shuren University, Hangzhou, China; e School of Pharmacy, Macau University of Science and Technology, Macau, China; f Faculty of Chemical and Pharmaceutical Technology and Biomedical Products, D .I . Mendeleev University of Chemical Technology of Russia, Moscow, Russian Federation

**Keywords:** SARS-CoV-2, peroxide, aminoperoxide derivatives, spike protein, RBD, Bio-layer interferometry

## Abstract

Severe acute respiratory syndrome coronavirus 2 (SARS-CoV-2) is the cause of the ongoing coronavirus disease 2019 (COVID-19) pandemic. Host cell invasion is mediated by the interaction of the viral spike protein (S) with human angiotensin-converting enzyme 2 (ACE2) through the receptor-binding domain (RBD). In this work, bio-layer interferometry (BLI) was used to screen a series of fifty-two peroxides, including aminoperoxides and bridged 1,2,4 – trioxolanes (ozonides), with the aim of identifying small molecules that interfere with the RBD-ACE2 interaction. We found that two compounds, compound **21** and **29**, exhibit the activity to inhibit RBD-ACE2. They are further demonstrated to inhibit SARS-CoV-2 cell entry, as shown in pseudovirus assay and experiment with authentic SARS-CoV-2. A comprehensive in silico analysis was carried out to study the physicochemical and pharmacokinetic properties, revealing that both compounds have good physicochemical properties as well as good bioavailability. Our results highlight the potential of small molecules targeting RBD inhibitors as potential therapeutic drugs for COVID-19.

## Highlights

• Bio-layer interferometry (BLI) was used to screen a library of 52 peroxides, including aminoperoxides and bridged 1,2,4 – trioxolanes (ozonides).
• Compounds 4, 21 and 29 exhibit the activity to inhibit RBD-ACE2 in vitro.
• Compounds 21 and 29 inhibit delta strain SARS-CoV-2. But compound 29 showed reduced activity against omicron BA.1 SARS-CoV-2.
• Citotoxicity and in silico studies revealing that 21 and 29 have low cytotoxicity, good physicochemical properties as well as good bioavailability.

## 1 Introduction

Coronavirus Disease 2019 (COVID-19), caused by SARS-CoV-2 coronavirus (Zhou et al., 2020; Zhu et al., 2020), is an ongoing pandemic that has claimed more than 6.9 million lives as of November 2023. Although several vaccines have been approved, these are not intended to treat already infected individuals. In addition, mutant variants of SARS-CoV-2 such as the Delta and Omicron variants of concern (VOC) are able to escape antibody neutralization, rendering vaccines less effective in preventing infection (Aggarwal et al., 2021; Cao et al., 2022). The limitations of vaccines are compounded by the insufficiency of therapeutic drugs for COVID-19 (Adibzadeh et al., 2022). Although several drugs, such as Paxlovid, remdesivir and multiple neutralizing monoclonal antibodies have been approved or granted emergency use authorization in some jurisdictions, their availability and affordability are called into question (Bavaro et al., 2023). Furthermore, the clinical benefit of some of these drugs, such as Remdesivir, is not supported by clinical trials (Consortium, 2022; Wang et al., 2020). Current treatment of COVID-19 largely relies on supportive care. The discovery of small molecule antiviral drugs may fill a gap in the current treatment paradigm of COVID-19 and contribute to the effort of keeping the pandemic in check. Furthermore, evidence suggests that SARS-CoV-2 can persist in patients’ gut (Natarajan et al., 2022), cerebrospinal fluid (Salman et al., 2021), and testes (Costa et al., 2022), potentially contributing to Post-acute Sequalae of COVID-19 (PASC) aka. “Long COVID” (Proal and VanElzakker, 2021). Clearance of viral reservoir is suggested as a strategy for treating PASC. Monoclonal antibodies may be insufficient for this role due to loss of efficacy to variants, inability to cross biological barriers, and being too expensive for long-term dosage. Small molecule antivirals that can be taken on a long-term basis with favorable tissue distribution profile can be incorporated into PASC treatment. Thus, we reason that the pursuit for small-molecule antiviral against SARS-CoV-2 remains highly relevant.

The SARS-CoV-2 life cycle depends critically on the functions of several viral proteins. In addition to main protease and RNA-dependent RNA polymerase for which drugs have been developed (Tian et al., 2021), blocking the entry of SARS-CoV-2 virions into host cell is another promising strategy for anti-viral development. However, the strategy is considered to have challenges. One challenge is that SARS-CoV-2 entry is mediated by the binding of the receptor-binding domain of its spike protein to host cell receptor ACE2, which is a form of protein-protein interaction (PPI) that is difficult to block with small molecules, due to the large area of interaction and free binding energy of PPI. The second challenge is that the spike protein and in particular the RBD is a highly mutated region of SARS-CoV-2 (Harvey et al., 2021). Thus, mutations may drive drugs targeting RBD ineffective. Nonetheless, we reason that small molecules can fit into yet uncharacterized, relatively conserved, cryptic binding pockets of the spike protein to block RBD-ACE2 interaction allosterically, and that such entry inhibitors with pan-sarbecovirus activity are possible. Such allosteric small molecule inhibitors are analogous to existing neutralizing antibodies that target non-ACE2-overlapping epitopes on RBD (McCallum et al., 2022), for example, sotrevimab, which retains efficacy against the highly mutated omicron variant (Cao et al., 2022). Affirming our belief, we have had success identifying natural compounds as effective SARS-CoV-2 entry inhibitors (Chen et al., 2021; Coghi et al., 2021; Yang et al., 2021; Zhang et al., 2021). Some of these remain potent against more recent variants such as the delta and omicron variants (unpublished data).

Compounds containing the peroxide bridge have been successful anti-infective agents. Artemisinin and its derivatives are effective anti-malarial drugs. Their efficacy is attributed to their peroxide bridge structure (Coghi et al., 2009; Haynes et al., 2010, 2013). Artemisinin and its derivative have been shown to be SARS-CoV-2 inhibitors (Ghosh et al., 2021; Herrmann et al., 2022). We previously showed that a derivative of artemisinin can bind to both the receptor binding domain of SARS-CoV-2 and ACE2 receptor, inhibiting viral entry (Coghi et al., 2021). This highlights the potential of peroxide compounds as SARS-CoV-2 entry inhibitors targeting RBD. In this work, we screened a library of peroxide compounds with chemical diversity in the hope of obtaining more potent SARS-CoV-2 entry inhibitors.

## 2 Materials and methods

### 2.1 Plasmid and reagents

Lentiviral packaging plasmids pHDM-Hgpm2 (#164441), pHDM-Tat1b (#164442), pRC—CMV-Rev1b (#164443) and VSV-G plasmid pMD2.G (#12259) were obtained from Addgene (MA, USA). Delta variant spike plasmid, luciferase transfer plasmid, human ACE2-mCherry transfer plasmid and human ACE2-EGFP transfer plasmid were purchased from VectorBuilder (Guangzhou, China). Omicron variant spike plasmid (MC_0101274) was purchased from Genscript (Nanjing, China), engineered in-house with a C-terminal deletion by In-Fusion recombination reaction (Takara, #638947) to enhance packaging efficiency as described previously(Yu et al., 2021) with the forward primer 5′-CGGCAGTTGCTGTTAATGACTCGAGTCTAGAGGGCCC-3′ and the reverse primer 5′-CATTAACAGCAACTGCCGCAGCTACAGCAGCCC-3′. Recombinant RBD proteins from SARS-CoV-2 variants were produced by Sino biological (delta plus: 40592-V08H115; gamma: 40592-V08H113; lambda: 40592-V08H86; omicron: 40592-V08H121). All other reagents are ACS grade, or at equivalent or higher grade, from major laboratory suppliers.

### 2.2 Preparation of compound library

The compounds are described in detail in Supporting Information III-Chemistry. All compounds were dissolved in anhydrous DMSO as 100 mM or 50 mM stock solution and stored at −80 °C.

### 2.3 Cell culture and virus strain

HEK293T overexpressing human ACE2 was established from a single clone of HEK293T cells (ATCC, CRL-3216) transduced with hACE2/mCherry lentivirus. For lentivirus and pseudovirus packaging, passage number 6–10 HEK293T cells (BNCC353535) from BeNa Culture Collection (Henan, China) were used. Vero E6 was obtained from ATCC. All cells were maintained in laboratory-formulated D10 medium composed of 90 % high-glucose DMEM, 10 % FBS, 1 mM pyruvate, 10 mM HEPES, 1 × Penicillin-Streptomycin-Glutamine (Gibco, 10378016) in a 37 °C humidified incubator with 5 % CO_2_. SARS-CoV-2 Delta (B.1.617.2) and Omicron BA.1 (B.1.1.529) were isolated from clinical samples and were deposited at the First Affiliated Hospital of Guangzhou Medical University. Viruses were propagated and stored at −80 °C and the titers of cultured viruses were estimated as 50 % tissue culture infective doses (TCID50) using the Reed–Muench method.

### 2.4 Bio-layer interferometry

All assays were run on an Octet Red96 instrument according to previously described method(Zhang et al., 2021). His-tagged RBD proteins were immobilized onto Ni-NTA probes at a concentration of 10 ng/mL in PBS. For high-throughput screening, probes were dipped in 800 μM solutions of test compounds in PBS. PBS with no compound served as negative control and 100 μM epigallocatechin gallate (EGCG), a compound we previously identified as a SARS-CoV-2 inhibitor that targets RBD(Zhang et al., 2021), served as positive control. The baseline, association and dissociation steps were 60 s, 120 s and 120 s, respectively. The signal was calculated as 100 % × (response of test compound - negative control)/(positive control - negative control). For binding kinetics experiments, probes were dipped in 2-fold serial dilution of compound solutions in PBS. The baseline, association and dissociation steps were 60 s, 120 s and 120 s, respectively. The binding kinetics curves of the same compound at different concentrations were aligned to 55–60 s of the baseline step and fitted to a 1:1 binding kinetics model using the ForteBio Data Analysis software 9.0.

### 2.5 ELISA

The ACE2 SARS-CoV-2 Spike Inhibitor Screening Assay Kit (Cat: 79936) was purchased from BPS Biosciences (San Diego, US). The experiment was done according to the kit manufacturer's protocol.

### 2.6 Immunofluorescence staining

Immunofluorescence of RBD-stained HEK293T cells overexpressing ACE2-EGFP fusion protein was done as described previously (Chen et al., 2021; Yang et al., 2021). Briefly, 293T cells were transfected with plasmid encoding ACE2 protein with EGFP fused to its C-terminus using Lipofectamine 3000 (Invitrogen, Cat. L3000150). The cells were grown on glass coverslip and incubated with recombinant delta plus or omicron BA.1 RBD protein with His-tag. RBD was visualized with anti-His-antibody conjugated to Alexa Fluor 647 (Cell Signaling Technology, 14931S) after fixation in 4 % paraformaldehyde. Images were acquired on a Leica TCS SP8 Laser Scanning Microscope.

### 2.7 Cytotoxicity assay

Compounds were diluted in D10 and serially diluted in a 96-well plate seeded with 1 × 10^4^ HEK293T-hACE2/mCherry or Vero E6 cells. After 48 h of culture, MTT prepared as a 5 mg/mL solution in PBS was added into each well to a final concentration of 0.5 mg/mL. The cells were cultured for another 4 h for the formazan crystals to form. The formazan was solubilized by adding 100 μL 10 % SDS 0.1 M HCl solution. Absorbance at 570 nm was read. Cell viability of each treated well was calculated as 100 % × (A_treated_−A_positive control_)/(A_negative control_ − A_positive control_), wherein positive control indicates wells with no cell and negative control indicates wells with cells but no compound. Median inhibitory concentration (IC_50_) was calculated by fitting data to a sigmoidal curve with Graphpad Prism 8.0.

### 2.8 SARS-CoV-2 pseudovirus and lentivirus packaging

SARS-CoV-2 pseudovirus on a lentiviral backbone and hACE2/mCherry lentivirus were packaged according to a previously described protocol(Crawford et al., 2020). 50 % confluent HEK293T cells in a 10 cm dish was transfected with a mixture of 1.5 μg of each of the packaging plasmids, 3 μg of spike plasmid or VSV-G plasmid, 7.5 μg of luciferase transfer plasmid or human ACE2-mCherry transfer plasmid using Lipo293 (Beyotime, C0521) according to manufacturer's instructions. Medium was refreshed with 10 mL of D10 after overnight incubation. Pseudovirus/lentivirus were allowed to accumulate in the supernatant for 48–56 h, harvested and clarified by centrifugation, and mixed with homemade 5 × concentrator (37.5 % PEG8000 and 1.5 M NaCl in PBS). Pseudovirus/lentivirus particles were precipitated by overnight incubation at 4 °C followed by centrifugation at 4000 g, 4 °C for 90 min. The precipitate was resuspended in 1 % the original volume of D10 and stored at −80 °C as 100 μL single-use aliquots.

### 2.9 SARS-CoV-2 pseudovirus neutralization assay

Concentrated pseudovirus was diluted in D10 containing designated concentrations of compound in a V-bottom 96-well plate and incubated in a 37 °C humidified incubator with 5 % CO_2_ for 1 h, and aliquoted into 96-well plate seeded with 1 × 10^4^ HEK293T-hACE2/mCherry the previous day. The pseudovirus preparation was titrated so that the amount of pseudovirus in each well produces 10^5^ relative luminescence unit (RLU). After 48 h of culture, cells were lysed in firefly luciferase assay reagent (Beyotime, RG051). Chemiluminescence signal was read on a microplate reader. Wells with cells but with no pseudovirus or compound were used as positive control. Wells with cells and pseudovirus but no compound were used as negative control. Data was analyzed similarly as in cytotoxicity assay.

### 2.10 Authentic SARS-CoV-2 neutralization assay

Vero E6 cells were seeded 1 × 10^4^/well in a 96-well plate. Each well was inoculated with 10^2^ 50 % Tissue Culture Infectious Dose (TCID_50_) of SARS-CoV-2. After 2 h of incubation in a 37 °C incubator, the inoculum was aspirated and replaced with 100 μL of D10 containing designated concentration of test compound. Cytopathic effect (CPE) was visually inspected under a microscope 3 days later. This and all subsequent experiments involving live SARS-CoV-2 were performed in the BSL-3 facility at State Key Laboratory of Respiratory Disease, National Clinical Research Center for Respiratory Disease, Guangzhou Institute of Respiratory Health, the First Affiliated Hospital of Guangzhou Medical University, Guangzhou Medical University, Guangzhou, China

### 2.11 Plaque reduction assay

Vero E6 cells monolayers in 12-well plates were rinsed with PBS and incubated with 100 TCID_50_ of SARS-CoV-2. Following 2 h of incubation, the inoculum was removed, and the cells were covered with agar/basic medium mixture, which contained 0.8 % agar and indicated concentrations of **21** or **29**. The plates were then incubated at 37 °C for 72 h, followed by fixation in 4 % formalin for 30 min. The overlays were then removed and stained with 0.1 % crystal violet for 3 min. The plaques were visualized and counted.

### 2.12 TCID50 assay for quantification of virus in the supernatant

2 × 10^5^ Vero E6 cells/well were seeded in 12-well plates in DMEM supplemented with 10 % FBS and cultured for 24 h at 37 °C. The cells were washed twice with PBS before the addition of 500 μL inoculum containing 100 TCID_50_ of SARS-CoV-2. The control wells were setup with DMEM medium containing 2 % FBS. After incubation at 37 °C for 2 h inoculum was removed. The cells were incubated with DMEM medium containing different concentrations of 21 or 29. The supernatants were collected after 48 h for TCID_50_ assay to assess viral virus titer.

### 2.13 Molecular properties calculation and drug-likeness studies

In silico calculations of the molecular properties and drug- likeness parameters for all compounds were performed based on theoretical approaches to identify the compounds which violate the optimum requirements for drug-likeness (Lipinski, 2000; Lipinski et al., 1997). Molecular properties (molecular weight, LogP value, number of hydrogen bond acceptor(s) (HBA), number of hydrogen bond donor(s) (HBD) incorporated in Lipinski's rule of five was predicted using Data Warrior software (version 5.5.0) (Supporting information Table S1)(Sander et al., 2015). Total polar surface area (TSPA), the number of rotatable bonds (nRot) were predicted also using same software as well as nonviolation of drug-likeness. Blood–brain barrier permeation (BBB) and GI absorption were predicted using by BOILED-Egg model (Supporting information Table S2)(Daina and Zoete, 2016). Effect on cytochrome (CYP) was evaluated by SwissADME(Daina et al., 2017). Toxicity prediction was calculated by ProTox web server (http://tox.charite.de/tox) (Banerjee et al., 2018).

### 2.14 Statistical analysis

Prism Graphpad 8.0 was used for data analysis. One-way ANOVA followed by Tukey's test was used to determine statistical significance for comparison between groups.

## 3 Results and discussion

### 3.1 Library of peroxide derivatives and study of their physicochemical properties

**Fig. 1:**
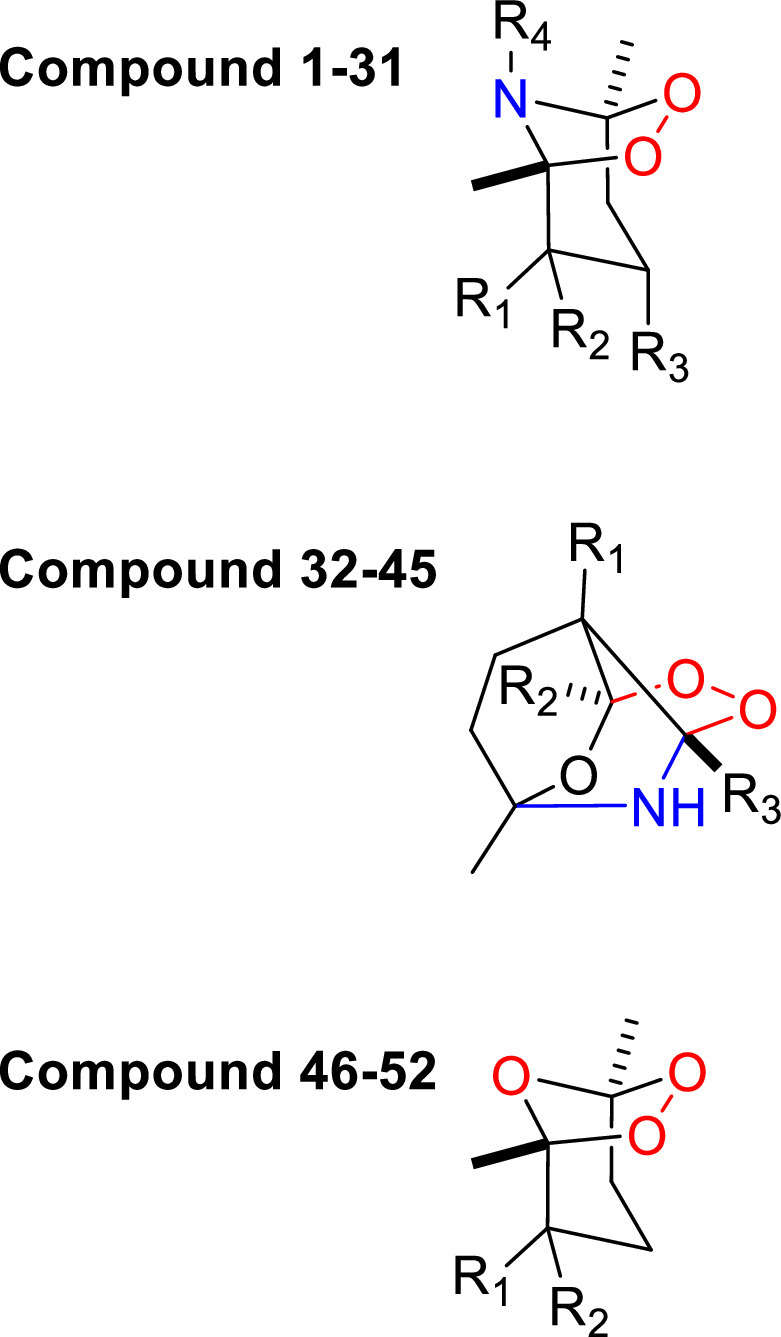
Chemical structures of peroxides in the library.

A library of peroxide derivatives containing 52 compounds was used in this study (Fig. 1, Figure S1). The synthesis of some of these compounds was reported in previous publications (Coghi et al., 2022; Yaremenko et al., 2022a, 2021, 2022b; Yaremenko et al., 2018). All compounds feature a peroxide bridge in a saturated heterocyclic ring system. The variety of compounds are derived from the substitutions on the ring system. All chiral compounds in the library were available as mixed isomers. Although it is possible for isomers to have different pharmacologic properties, costly separation of isomers is not justifiable for the purpose of drug screening and thus was not performed. The synthesis and characterization of compounds are described in detail in Supporting Information III-Chemistry.

As a part of this study, compounds tested were assessed by predicting their physicochemical properties and oral bioavailability. Lipinski rule is commonly used in drug design and states that most “drug-like” molecules have molecular weight (MW≤500), partition coefficient LogP≤5, number of hydrogen donors (HBD≤5), number of acceptors (HBA≤10) and number of rotatable bonds (nRot≤10) (Lipinski et al., 1997). In fact, a compound that meets Lipinski's “Rule of Five” is more likely to be a suitable drug. Compounds violating more than one of these rules may have bioavailability problems (Lipinski, 2000). None of the compounds violate more than one Lipinski's rule.

This finding corroborates the results of gastrointestinal absorption from Data Warrior (Sander et al., 2015) in which compounds were predicted by BOILED-Egg model with different probability to passively permeate through the blood–brain barrier absorption (Daina and Zoete, 2016) (Table S2). Many compounds are predicted to be able to permeate blood-brain-barrier, an important property if clearance of potential viral reservoir in the CNS is intended (Table S2).

Essential is the knowledge about interaction of molecules with cytochromes P450 (CYP) and SwissADME was used for the estimation for compounds analyzed to be inhibitor of the most important CYP isoenzymes (Daina et al., 2017). The prediction of compound toxicities is an important part of the drug design development process and toxicity of all compounds was performed using free web tool ProTox (Table S3) (Banerjee et al., 2018). All compounds showed a good toxicology profile. While some compounds (**24, 29, 50, 51**) are predicted to have hepatotoxicity, none are predicted to have immunotoxicity, activate PPARγ or heat shock response elements, or interfere with mitochondrial membrane potential.

### 3.2 Discovery of SARS-CoV-2 RBD binders from peroxide derivatives

**Fig. 2:**
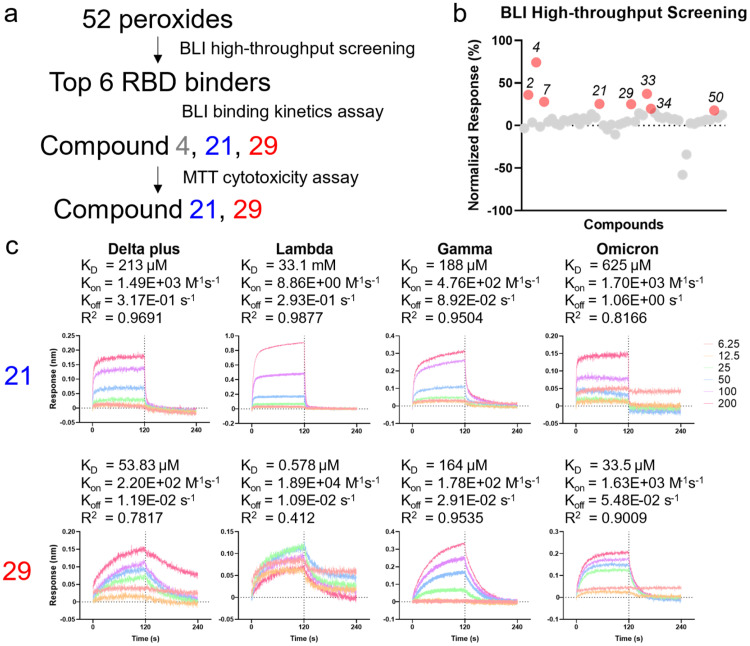
Screening of the peroxide for RBD binder and validation of RBD-binding affinity using BLI **a**, a schematic representation of the compound screening and validation procedure. **b**, Result of high-throughput screening of 52 compounds. Data are shown as BLI response normalized to response elicited by EGCG which is considered 100 %. **c**, Binding affinity of compound 21 and 29 with 4 RBD variants corresponding to delta plus, lambda, gamma and omicron SARS-CoV-2 was measured with BLI binding kinetics assay. A range serial two-fold dilution of compounds from 6.25 to 200 μM was used. K_D_: dissociation constant; Kon: association rate; Koff: dissociation rate; R2: goodness-of-fit to the 1:1 binding kinetics model. Data representative of at least three experiments.

We applied bio-layer interferometry, a method to detect molecular binding, to screen the library of 52 peroxides for ones with binding affinity to RBD (Fig. 2a). The recombinant RBD fragment used in screening corresponds to PANGOLIN B.1.617.2 AY.2/AY.3 “delta plus” variant of SARS-CoV-2, with K417N, L452R, T478K mutations, representative of the prevalent variant when this study was conducted. The signal response elicited by 800 μM peroxide was standardized to the percentage between negative control PBS and 100 μM EGCG, a robust RBD binder we previously identified (Zhang et al., 2021), as positive control. The top 6 compounds were selected to be tested for confirmatory screen based on the practicality of testing this number of compounds (Fig. 1b). Among the six compounds assayed, compounds **4, 21** and **29** showed relatively strong binding affinity to delta plus RBD (Fig. 2c, S2). We further assayed their cytotoxicity since it is an undesirable trait for drug candidates. Compound **4**, despite having the strongest binding affinity to delta plus RBD, was excluded from further investigation due to strong cytotoxic effects (Figure S3). Compounds **21** and **29** were found to have acceptable cytotoxicity (Figure S3). Active compounds **21** and **29** violate only one of Lipinski's rules, indicating their drug-like character and a good chance for oral administration (Table S1). Compounds **21** and **29** were predicted to be orally bioavailable, absorbed in human intestine, and unable to cross BBB (Table S2). Compound **29** was predicted to have potential hepatotoxicity. Other than this, they are predicted to have little toxicity.

### 3.3 Compounds 21 and 29 inhibit RBD-ACE2 binding

**Fig. 3:**
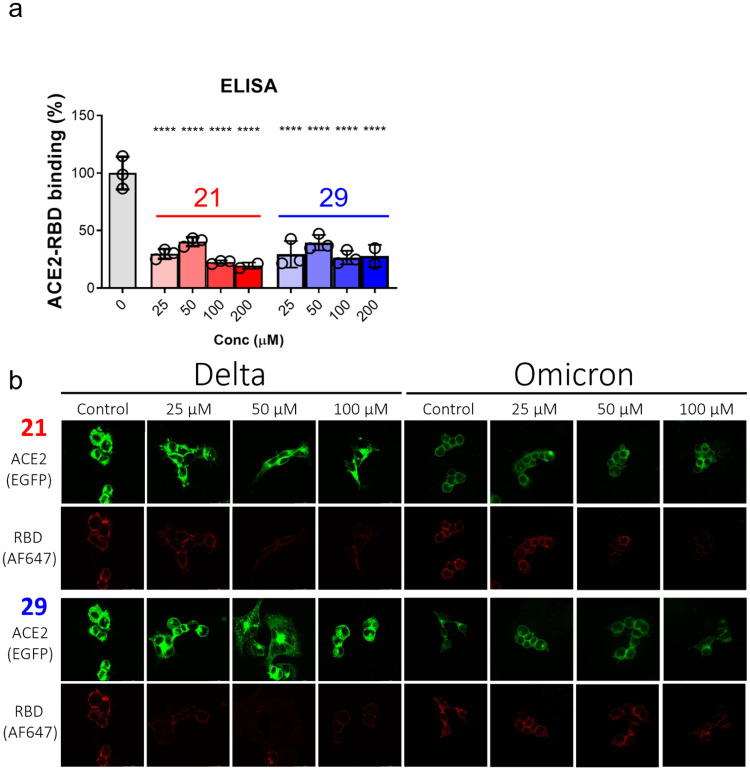
Inhibition of RBD-ACE2 binding by compound **21** and **29** **a**, ELISA was used to determine inhibition of RBD-ACE2 binding. Concentrations of 0, 25, 50, 100 and 200 μM were used for both compounds. Technical triplicate was performed. **b**, Immunofluorescence staining was used to determine the ability of the compounds to inhibit RBD binding to cell surface ACE2. RBD was incubated with HEK293T transfected to overexpress ACE2-EGFP fusion protein in the presence of 0 (control), 25, 50 and 100 μM of compound. The RBD bound to cell surface was visualized with anti-His-tag antibody conjugated to Alexa Fluor 647 (AF647) fluorophore. One-way ANOVA followed by Tukey's test for comparison between groups was used to determine statistical significance between groups in **a**. P value for comparison with the 0 μM group was shown. *: *p*<0.0; **: *p*<0.01; ***: *p*<0.001; ****: *p*<0.0001; non-significant results were not labeled.

A major concern for entry inhibitors is that even though they can bind to RBD, they can be outcompeted by ACE2 for binding as PPI generally has much higher affinity and larger area of interaction. Therefore, binding affinity to RBD does not necessarily correlate with the ability to block RBD-ACE2 interaction. To address this question, we applied an ELISA-based assay to determine whether these compounds block RBD-ACE2 binding. Indeed, both **21** and **29** inhibited the binding between ACE2 and wild type RBD at concentrations of 25, 50, 100 and 200 μM (Fig. 3a). The commercial ELISA assay we used involved only WT RBD protein. We investigated whether our compounds could inhibit the binding between variant RBD and ACE2 by immunostaining live HEK293T cells expressing ACE2 with recombinant RBD protein. Indeed, delta plus and omicron RBD binding to cell surface ACE2 is detected in the absence of compounds, the presence of both compounds **21** and **29** at 25, 50 and 100 μM concentrations reduced RBD immunofluorescence signal (Fig. 3b).

### 3.4 Compounds 21 and 29 inhibit SARS-CoV-2 cell entry

**Fig. 4:**
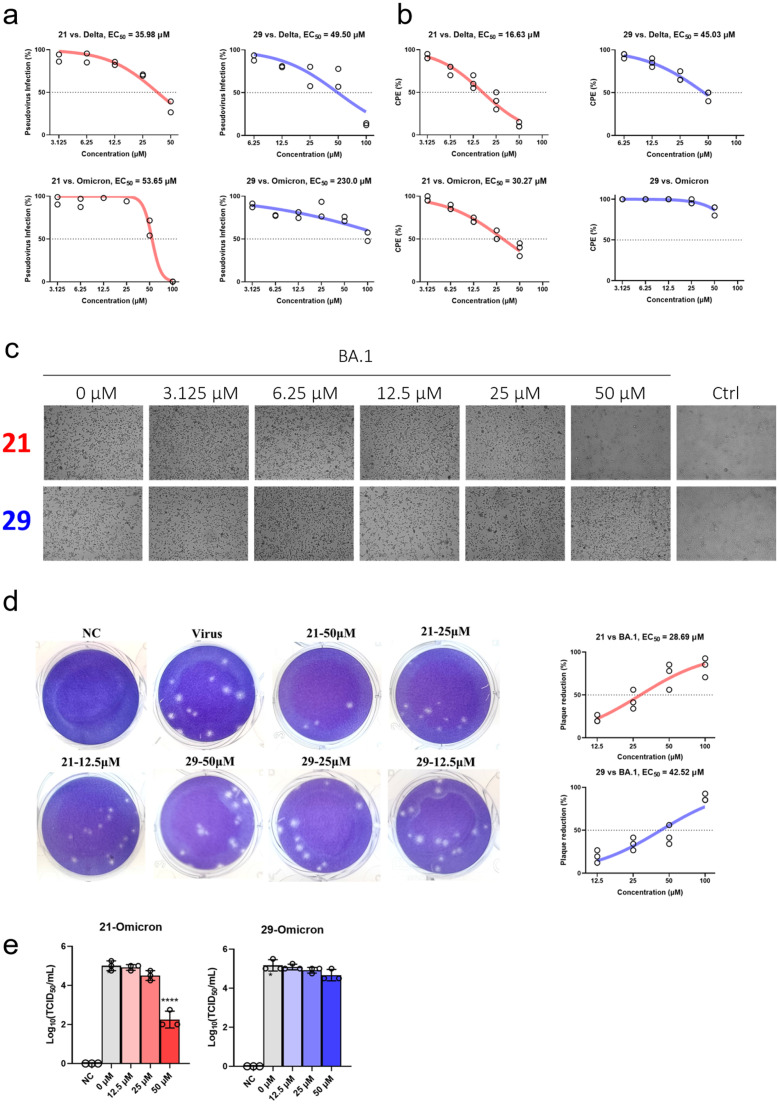
Compound **21** and **29** inhibits SARS-CoV-2 infection in vitro. **a**, Compounds **21** and **29** inhibits delta but not omicron SARS-CoV-2 pseudovirus. **b**, inhibitory effects of compound **21** and **29** on Vero E6 cell CPE induced by authentic SARS-CoV-2 infection. **c,** (related to b) microscopic image of CPE in omicron BA.1-infected cells treated with 0, 3.125, 6.25, 12.5, 25 and 50 μM of compound 21 or 29. **d,** plaque reduction assay for the ability of compound **21** and **29** to inhibit omicron BA.1 entry. Left: image of plaque of Vero E6 cells representative of 3 replicates; right: dose-response curve of plaque reduction. **e,** viral load in supernatant of Vero E6 infected with omicron BA.1 and treated with 0, 12.5, 25 and 50 μM of compound **21** or **29**. Technical duplicate was performed for **a**, triplicate for **b, d** and **e**. Dose-response data in **a, b** and **d** were fitted to a sigmoidal model to calculate EC_50_. One-way ANOVA followed by Tukey's test was used to determine statistical significance between groups in **e**. P value for comparison with the 0 μM group was shown. *: *p*<0.05; **: *p*<0.01; ***: *p*<0.001; ****: *p*<0.0001; non-significant results were not labeled.

We tested the ability of compound **21** and **29** to inhibit SARS-CoV-2 cell entry using an established luciferase reporter SARS-CoV-2 pseudotyped virus platform involving no replication-competent virus. Both compounds inhibit delta (L452R, T478K) pseudovirus entry into HEK293T cells stably expressing ACE2. However, they have markedly reduced inhibitory effects on omicron pseudovirus (Fig. 4a), to the point of being indistinguishable from cytotoxicity.

We further tested the inhibitory effect of these compounds on authentic SARS-CoV-2. In agreement with pseudovirus assay, both compounds effectively inhibit delta strain SARS-CoV-2 replication in Vero E6 cells as indicated by cytopathic effect (CPE) (Fig. 4b). Their inhibitory effects in Omicron SARS-CoV-2 were compromised.

We further performed plaque reduction assay to assess the ability of compound **21** and **29** to inhibit virus entry using the omicron BA.1 variant. Both compounds inhibit virus entry as determined by plaque reduction (Fig. 4d).

To assess the ability of these compounds to inhibit virus replication, we infected Vero E6 cells with omicron BA.1 and treated them with the compounds. Viral load in the supernatant was quantified by measurement of TCID_50_. Both compounds significantly reduced viral load in the supernatant at 50 μM (Fig. 4e), demonstrating the ability to inhibit virus replication.

## 4 Conclusions

In this study, we identified two peroxide compounds that inhibits SARS-CoV-2 coronavirus by binding to the receptor binding domain of its spike glycoprotein. Compound **21** retains much of its inhibitory effects on Omicron BA.1, while compound **29** has markedly reduced bioactivity against the cellular entry of omicron variant SARS-CoV-2 compared with delta variant.

Both compounds **21** and **29** have comparable binding affinity with all tested RBD variants, including omicron, and can inhibit RBD-ACE2 interaction in a cell-based assay. This suggests that the extensive mutations in omicron RBD does not disrupt the binding of compounds to RBD or their ability to inhibit RBD-ACE2 interaction. Although yet to be confirmed by experimental evidence such as X-ray diffraction crystallography, we presume that these peroxide compounds may target a relatively conserved region of the RBD, hence the unaffected binding affinity. We could not obtain a high-confidence model of the binding mode between RBD and these compounds due to lack of binding pocket on RBD (data not shown), suggesting induced fit may be involved in the binding between RBD and the small molecule. We surmise that the escape of omicron variant from the two compounds may be attributed to altered cell entry program mediated by omicron spike glycoprotein. Studies show that the omicron BA.1 is weaker in inducing membrane fusion and poorly utilize TMPRSS2 in membrane entry, possibly preferring endosomal pathway for cell invasion (Meng et al., 2022; Willett et al., 2022). The omicron BA.2 spike, however, restores fusogenicity.

Finally, prediction of the physicochemical and pharmacokinetic properties of the target compounds using software showed that both compounds had good physicochemical properties as well as good bioavailability.

Our work shows that the idea of small molecule SARS-CoV-2 antivirals targeting RBD is feasible. However, resolving the binding mode between RBD and small molecules inhibitors, so is an overall understanding of the cellular entry process of SARS-CoV-2, is necessary to guide design of successful SARS-CoV-2 entry inhibitors.

## Funding

VKWW acknowledges funding from FDCT grant from the Macao Science and Technology Development Fund (Project code 0033/2019/AFJ; 001/2020/ALC). P.C. acknowledges funding from FDCT grant from the Macao Science and Technology Development Fund (Project code 0096/2020/A; 0005/2023/RIA1). The synthesis and analysis of peroxides was supported by the 10.13039/501100006769Russian Science Foundation (Grant No 21–43–04417) to A.O.T and I.A.Y.

The funding sources play no role in the design of the study and interpretation of data.

## Supporting Information

Supporting Information I-Supporting Figures

Supporting Information II-Supporting Tables

Supporting Information III-Chemistry

## CRediT authorship contribution statement

**Ding-qi Zhang:** Data curation, Methodology, Investigation, Writing – original draft. **Qin-hai Ma:** Investigation, Methodology. **Meng-chu Yang:** Investigation, Methodology. **Yulia Yu. Belyakova:** Investigation, Methodology. **Zi-feng Yang:** Project administration. **Peter S. Radulov:** Investigation, Methodology. **Rui-hong Chen:** Investigation, Methodology. **Li-jun Yang:** Investigation, Methodology. **Jing-yuan Wei:** Investigation, Methodology. **Yu-tong Peng:** Investigation, Methodology. **Wu-yan Zheng:** Investigation, Methodology. **Ivan A. Yaremenko:** Investigation, Methodology, Writing – review & editing. **Alexander O. Terent'ev:** Writing – review & editing, Conceptualization, Supervision, Funding acquisition. **Paolo Coghi:** Writing – review & editing, Conceptualization, Supervision, Funding acquisition. **Vincent Kam Wai Wong:** Conceptualization, Methodology, Supervision, Funding acquisition.

## Declaration of Competing Interest

The authors declare that they have no known competing financial interests or personal relationships that could have appeared to influence the work reported in this paper.

## Data Availability

Data will be made available on request.

## Appendix Supplementary materials

Image, image 1

Image, image 2

Image, image 3

Image, image 4

Image, application 5

Image, application 6
