# Supplementary figures and images for: Peroxide derivatives as SARS-CoV-2 entry inhibitors
Source: Virus Res. 2023 Dec 12;340:199295. doi: 10.1016/j.virusres.2023.199295 (PMC10733699; doi:10.1016/j.virusres.2023.199295)

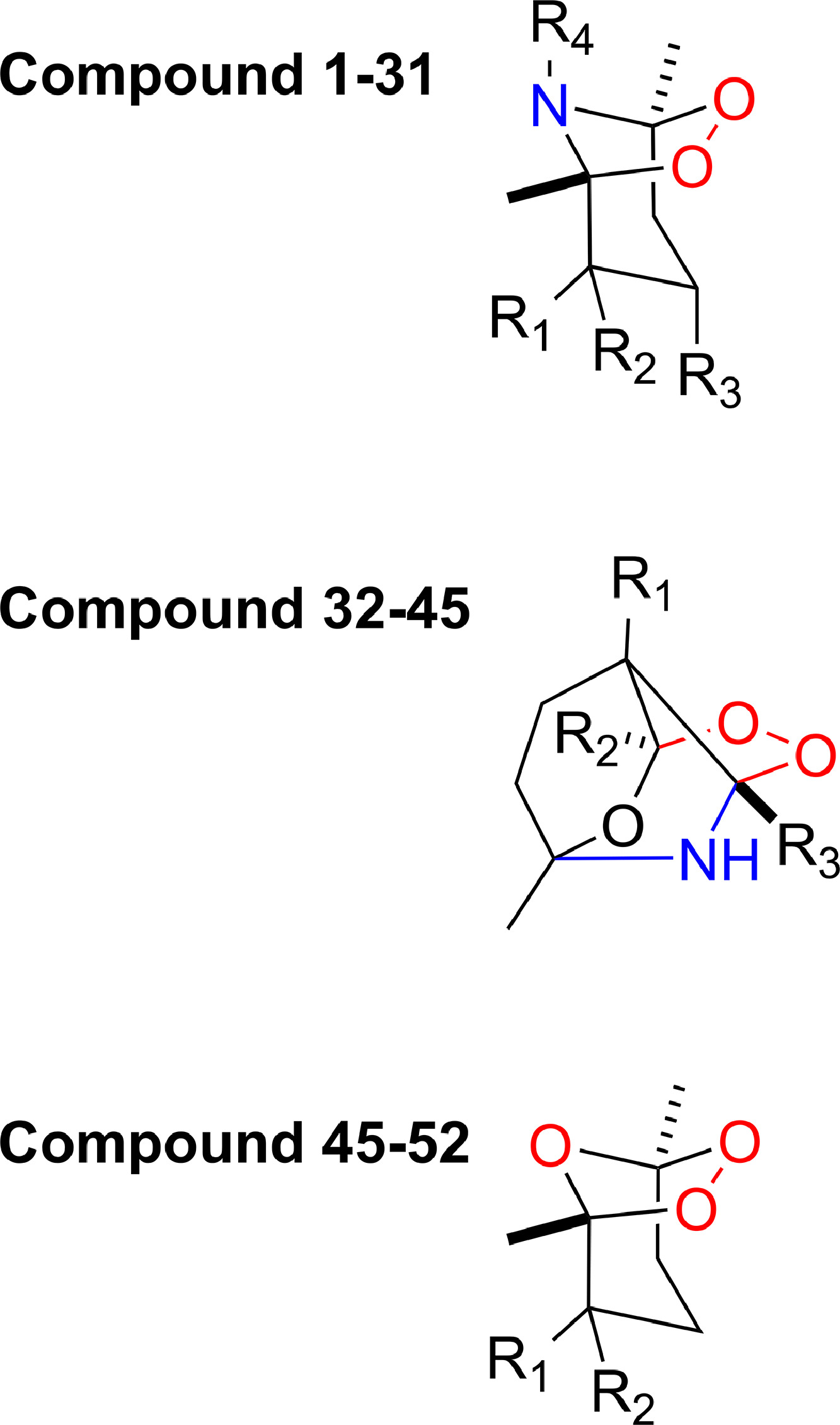

Supplement: Supplementary file 1 [file mmc1.jpg]

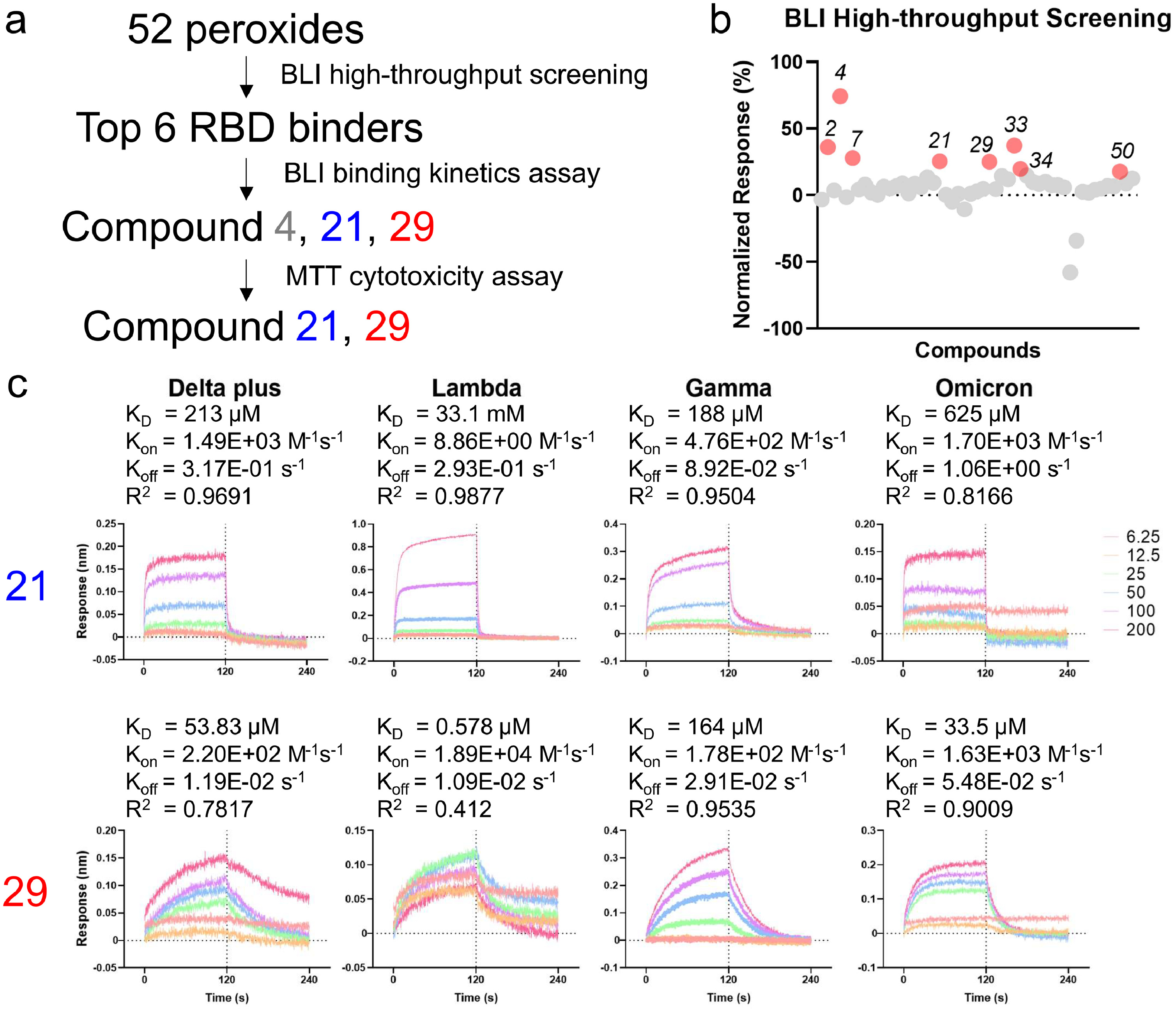

Supplement: Supplementary file 2 [file mmc2.jpg]

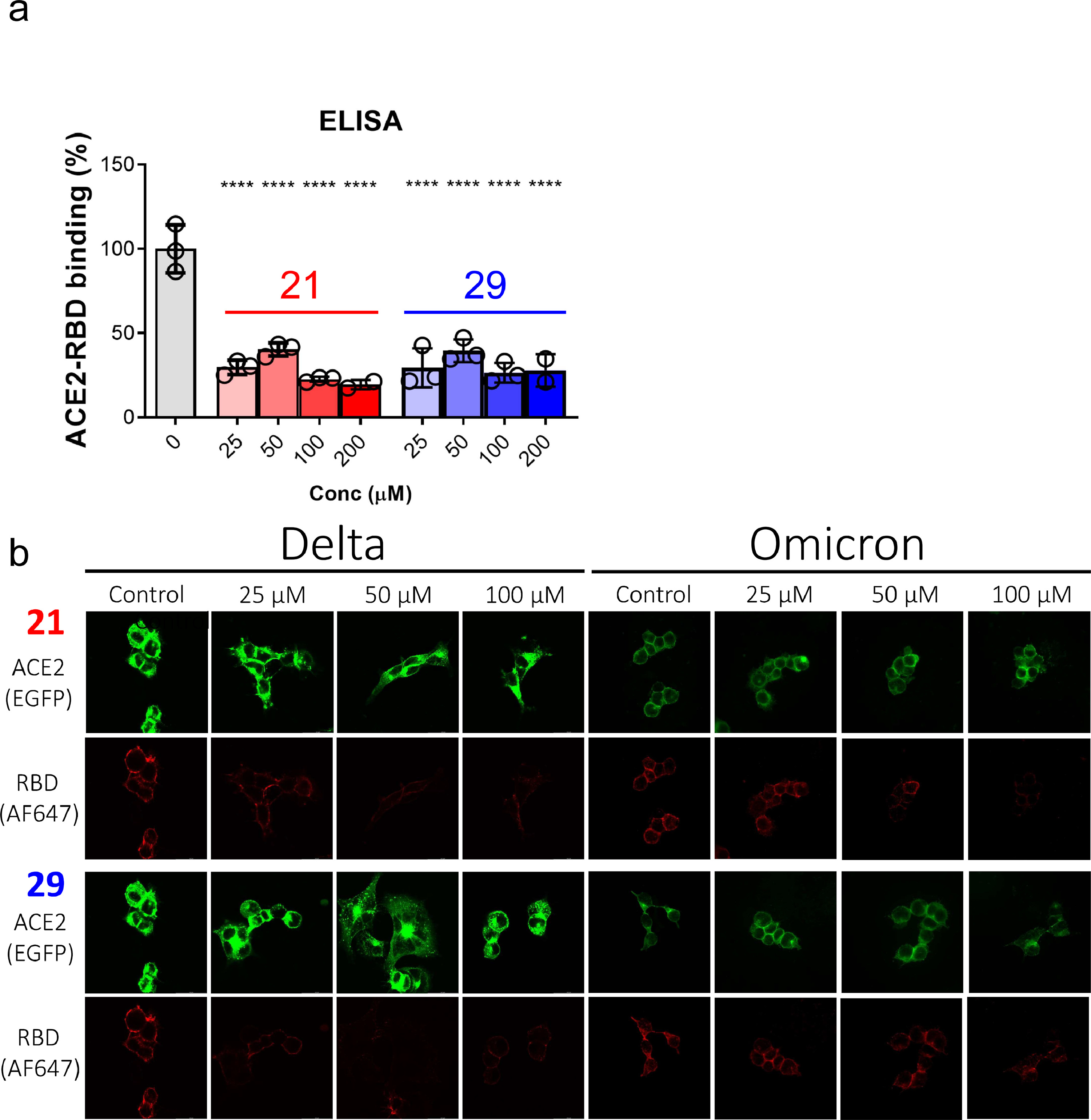

Supplement: Supplementary file 3 [file mmc3.jpg]

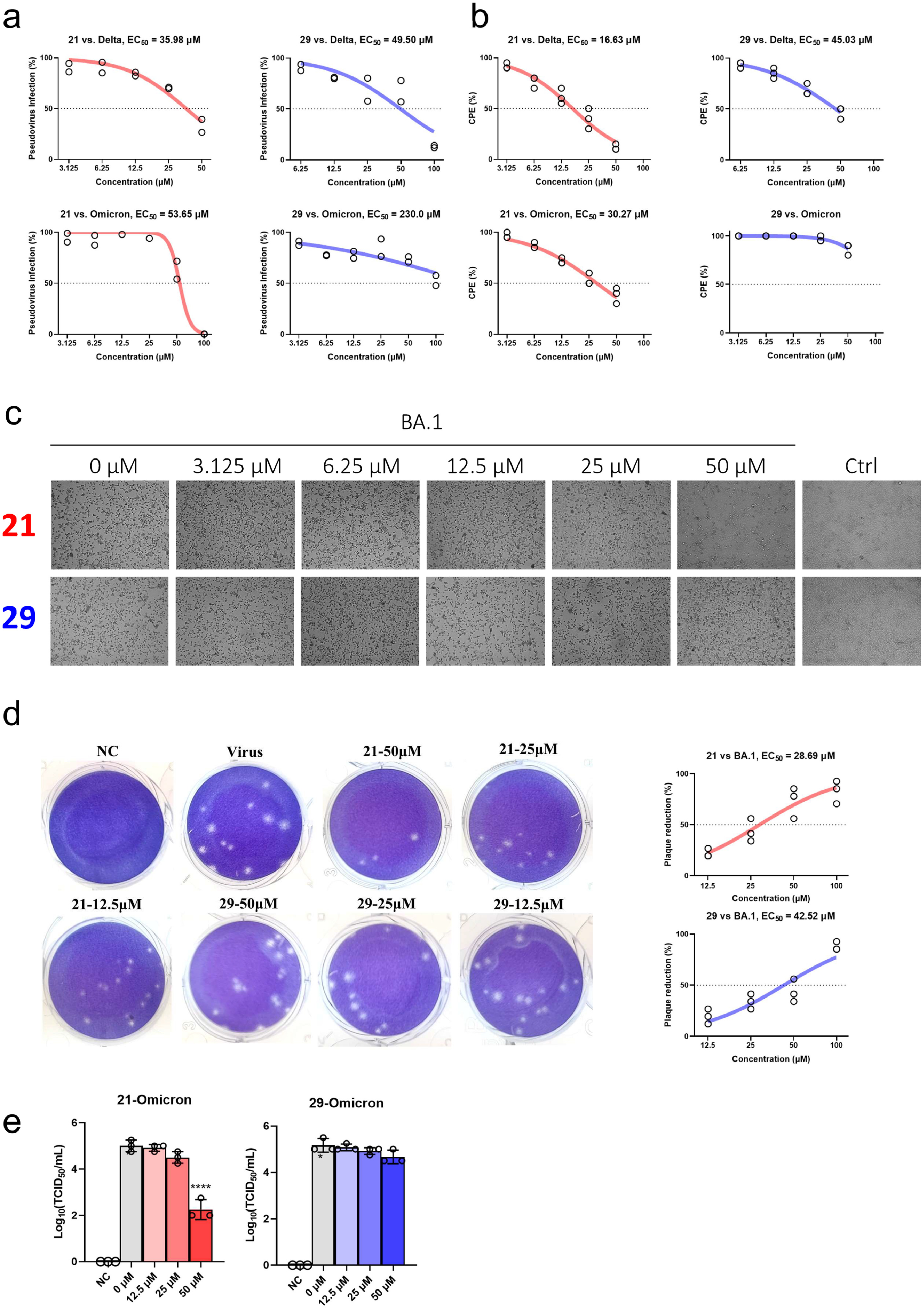

Supplement: Supplementary file 4 [file mmc4.jpg]
