# Supplementary material for: Peroxide derivatives as SARS-CoV-2 entry inhibitors
Source: Virus Res. 2023 Dec 12;340:199295. doi: 10.1016/j.virusres.2023.199295 (PMC10733699; doi:10.1016/j.virusres.2023.199295)
Supplement: Supplementary file 5 [file mmc5.pptx]

## Slide 1
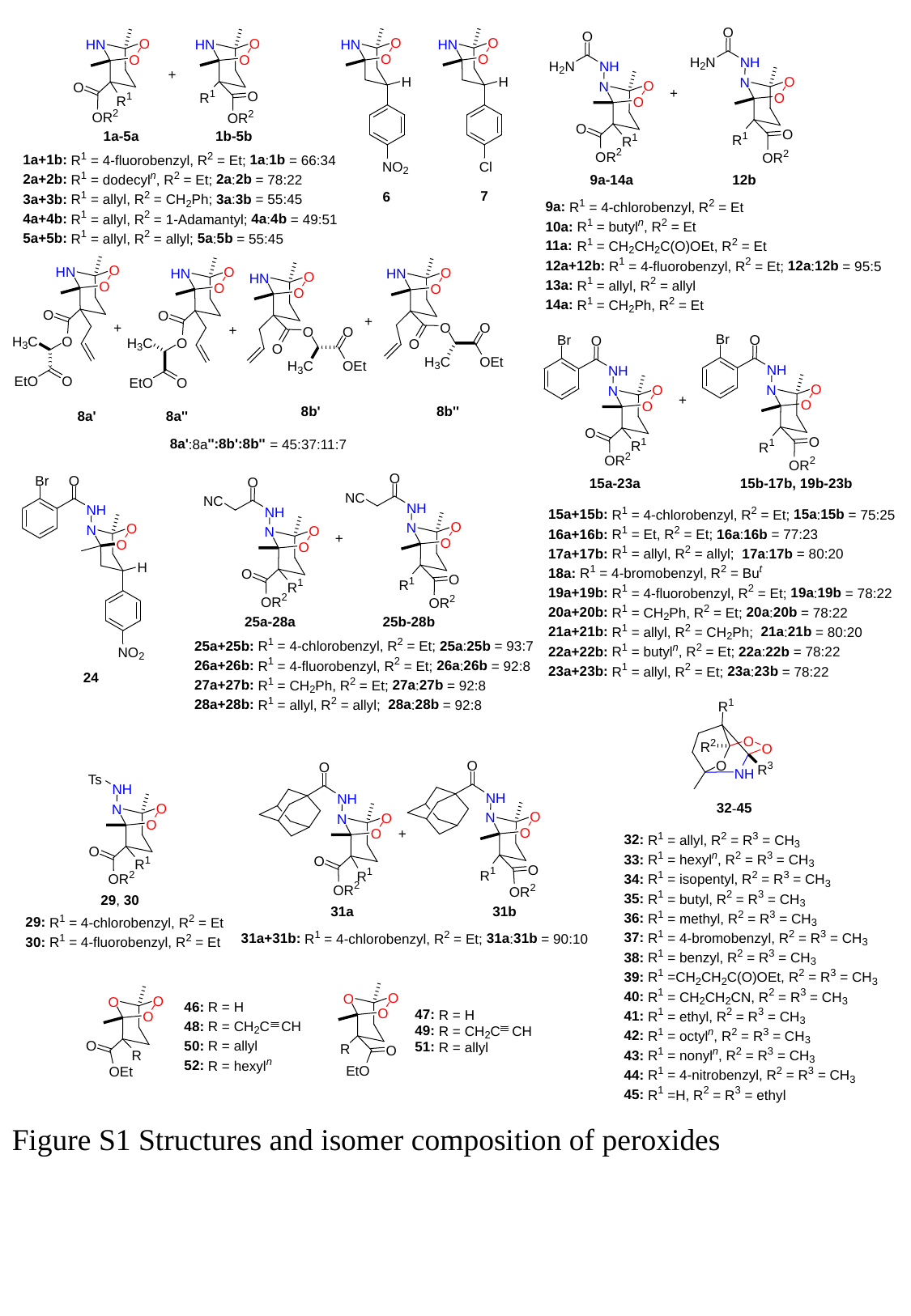

Figure S1 Structures and isomer composition of peroxides

## Slide 2
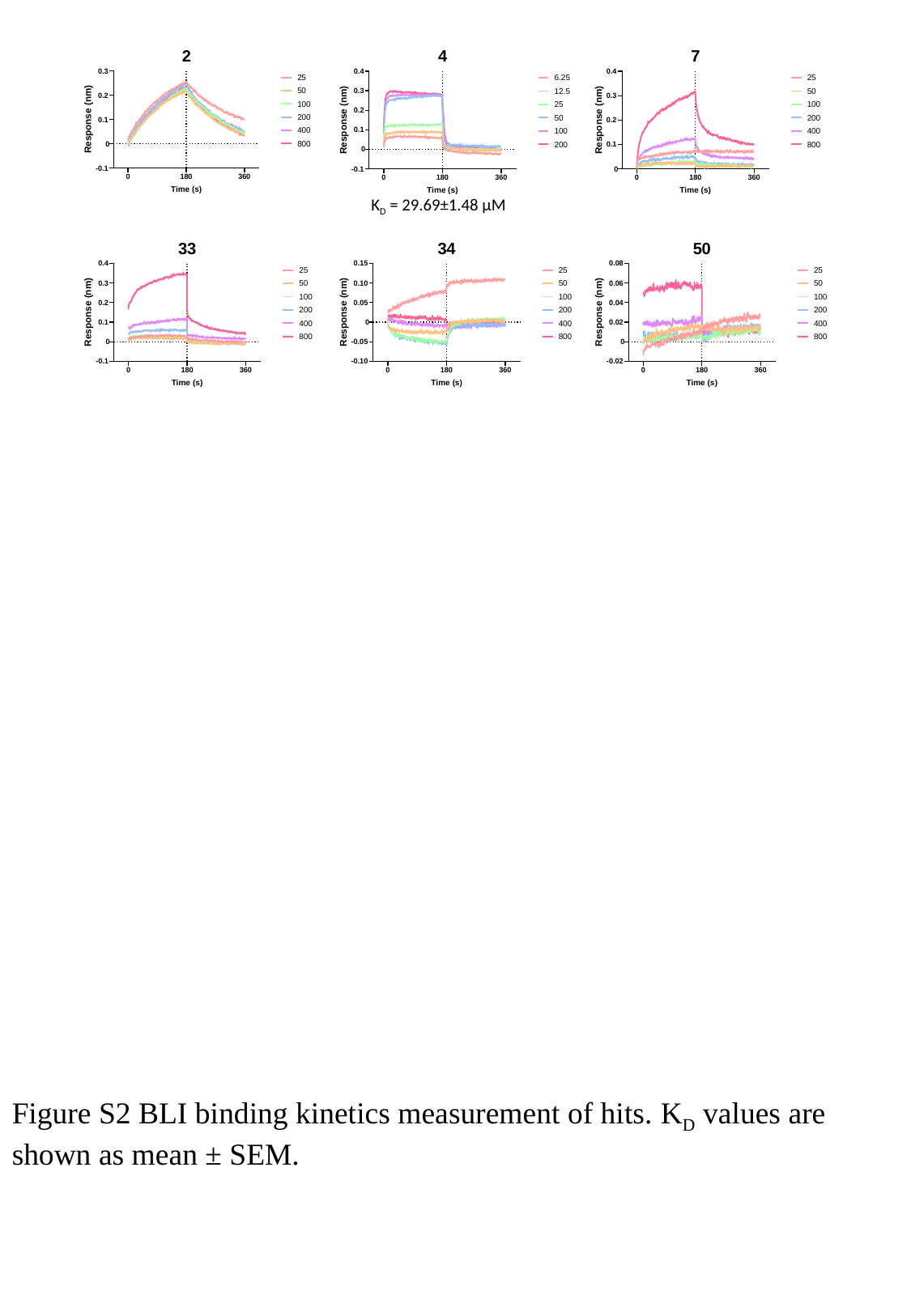

KD = 29.69±1.48 μM
Figure S2 BLI binding kinetics measurement of hits. KD values are shown as mean ± SEM.

## Slide 3
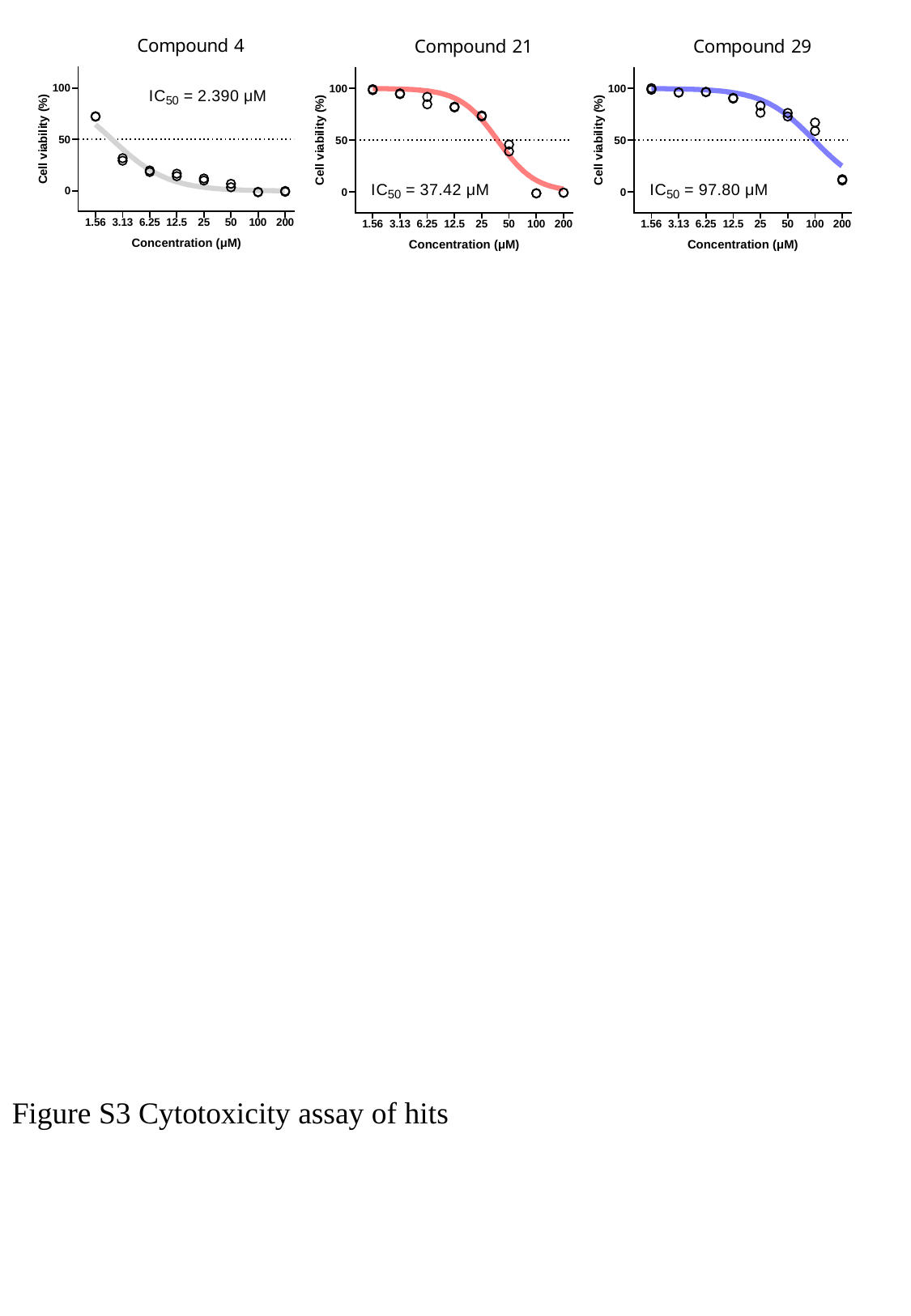

Figure S3 Cytotoxicity assay of hits
