# Supplementary material for: Peroxide derivatives as SARS-CoV-2 entry inhibitors
Source: Virus Res. 2023 Dec 12;340:199295. doi: 10.1016/j.virusres.2023.199295 (PMC10733699; doi:10.1016/j.virusres.2023.199295)
Supplement: Supplementary file 6 [file mmc6.pdf]

Supporting Information III-Chemistry

Peroxide Derivatives as SARS-CoV-2 Entry Inhibitors

Ding-qi Zhang[a*], Qin-hai Ma[b*], Meng-chu Yang[a*], Yulia Yu Belyakova[c], Zi-feng Yang[b], Peter S. Radulov[c], Rui-hong Chen[a], Li-jun Yang[d], Jing-yuan Wei[e], Yu-tong Peng[e], Wu-yan Zheng[a], Ivan A. Yaremenko[c,f], Alexander O. Terent'ev[c#], Paolo Coghi[e#], Vincent Kam Wai Wong[a#]

[a]Dr. Neher's Biophysics Laboratory for Innovative Drug Discovery, State Key Laboratory of Quality Research in Chinese Medicine, Macau University of Science and Technology, Macau, China

[b]State Key Laboratory of Respiratory Disease, National Clinical Research Center for Respiratory Disease, Guangzhou Institute of Respiratory Health, the First Affiliated Hospital of Guangzhou Medical University, Guangzhou Medical University, Guangzhou, China

[c]N. D. Zelinsky Institute of Organic Chemistry, Russian Academy of Sciences, Moscow, Russian Federation

[d]Institute of Translational Medicine, Zhejiang Shuren University, Hangzhou, China

[e]School of Pharmacy, Macau University of Science and Technology, Macau, China

[f]Faculty of Chemical and Pharmaceutical Technology and Biomedical Products, D .I . Mendeleev University of Chemical Technology of Russia, Moscow, Russian Federation

*These authors contributed equally: Ding-qi Zhang, Qin-hai Ma and Meng-chu Yang

#Corresponding authors: Prof. Vincent Kam Wai Wong, Dr. Paolo Coghi, and Prof. Alexander O. Terent'ev

Address correspondence: H703, Block H Science and Technology Building, Macau University of Science and Technology, Avenida Wai Long, Taipa, Macau, China;

Tel.: +853-8897-2408 (VK.W.W.);

Fax: +853-2882-2799 (V.K.W.W.);

E-mail: bowaiwong@gmail.com (V.K.W.W.);

Keywords: SARS-CoV-2, peroxide, aminoperoxide derivatives, spike protein; RBD; bio-layer interferometry.

Experimental part

Caution: Although we have encountered no difficulties in working with the peroxides described below, the proper precautions, such as the use of shields, fume hoods, and the avoidance of transition metal salts, heating and shaking, should be taken whenever possible.

NMR spectra were recorded on a commercial instrument (300.13, 500.13 MHz for ^{1}H, 75.48, 125.76 MHz for ^{13}C, 40.56 MHz for ^{15}N) in $CDCl_3$. The ^{15}N NMR spectra were referenced to external CH_3NO_2 and converted to the liquid ammonia scale.High resolution mass spectra (HRMS) were measured using electrospray ionization (ESI). The measurements were done in a positive ion mode (interface capillary voltage 4500 V); the mass ratio was from m/z 50 to 3000 Da; external/internal calibration was done with Electrospray Calibrant Solution. A syringe injection was used for solutions in MeCN (flow rate 3 μL/min). Nitrogen was applied as a dry gas; interface temperature was set at 180 °C.

The TLC analysis was carried out on silica gel chromatography plates Macherey-Nagel Alugram UV254; Sorbent: Silica 60, specific surface (BET) ~ 500 m^2/g, mean pore size 60 Å, specific pore volume 0.75 mL/g, particle size 5–17 μm; Binder: highly polymeric product, which is stable in almost all organic solvents and resistant towards aggressive visualization reagents. The melting points were determined on a Kofler hot-stage apparatus. Chromatography of triketones was performed on silica gel (0.060-0.200 mm, 60 A, CAS 7631-86-9). Chromatography of peroxides was performed on silica gel (0.040-0.060 mm, 60 A, CAS 7631-86-9). Elemental analysis on carbon, hydrogen, and nitrogen was carried out using a CHN analyzer. Determination of purity of all peroxides was executed by elemental (combustion) analysis. For all peroxides, deviation from the theoretical values for C, H, and N content was less than 0.4%. These data confirm >95% purity of compounds. Structures of compounds were confirmed using ^{1}H and ^{13}C NMR spectra.

Ethyl acetoacetate, methyl vinyl ketone, $CeCl_3 \cdot 7H_2O$, benzyl and alkyl halides, *2*-bromobenzoic acid hydrazide, 2-cyanoacetohydrazide, adamantane-1-carbohydrazide, 4-methylbenzenesulfonohydrazide, semicarbazide hydrochloride were purchased from Acros. Ethyl acetate (EA),petroleum ether (PE) (40/70), Methanol, THF, CH_3CN, $CHCl_3$, H_2O_2 (35% aqueous solution), NaI, NH_4OAc, $MgSO_4$, NaOH, 98% H_2SO_4, $BF_3 \cdot Et_2O$, NH_3 aq. were purchased from commercial suppliers. A solution of H_2O_2 in Et_2O (5.4 M) was prepared by the extraction with Et_2O (5×100 mL) from a 35% aqueous solution (100 mL) followed by drying over $MgSO_4$. Then, part of Et_2O was removed in the vacuum of a membarne vacuum pump at 20–25 ° C. 1,5–Diketones **A**[1-4, 5] and β,δ'–triketones **C**[6, 7, 8] were synthesized according to a known procedures.

Figure S1 Structures and isomer composition of peroxides.

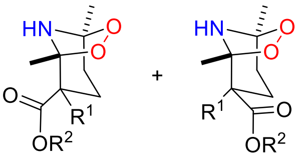

1a-5a **1b-5b**

1a+1b: R^1 = 4-fluorobenzyl, R^2 = Et; **1a**:**1b** = 66:34
2a+2b: R^1 = dodecyl^n, R^2 = Et; **2a**:**2b** = 78:22
3a+3b: R^1 = allyl, R^2 = CH_2Ph; **3a**:**3b** = 55:45
4a+4b: R^1 = allyl, R^2 = 1-Adamantyl; **4a**:**4b** = 49:51
5a+5b: R^1 = allyl, R^2 = allyl; **5a**:**5b** = 55:45

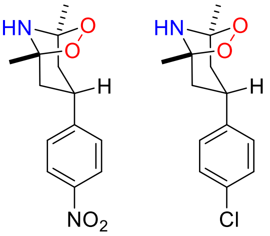

6 **7**

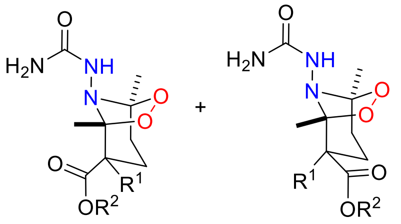

9a-14a **12b**

9a: R^1 = 4-chlorobenzyl, R^2 = Et
10a: R^1 = butyl^n, R^2 = Et
11a: R^1 = $CH_2CH_2C(O)OEt$, R^2 = Et
12a+12b: R^1 = 4-fluorobenzyl, R^2 = Et; **12a**:**12b** = 95:5
13a: R^1 = allyl, R^2 = allyl
14a: R^1 = CH_2Ph, R^2 = Et

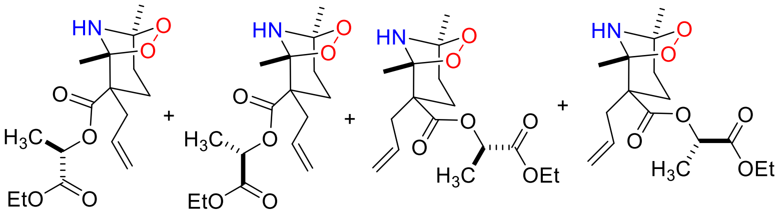

8a' **8a''** **8b'** **8b''**

8a':8a**''**:**8b'**:**8b''** = 45:37:11:7

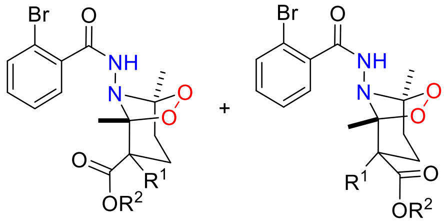

15a-23a **15b-17b, 19b-23b**

15a+15b: R^1 = 4-chlorobenzyl, R^2 = Et; **15a**:**15b** = 75:25
16a+16b: R^1 = Et, R^2 = Et; **16a**:**16b** = 77:23
17a+17b: R^1 = allyl, R^2 = allyl; **17a**:**17b** = 80:20
18a: R^1 = 4-bromobenzyl, R^2 = Bu^t
19a+19b: R^1 = 4-fluorobenzyl, R^2 = Et; **19a**:**19b** = 78:22
20a+20b: R^1 = CH_2Ph, R^2 = Et; **20a**:**20b** = 78:22
21a+21b: R^1 = allyl, R^2 = CH_2Ph; **21a**:**21b** = 80:20
22a+22b: R^1 = butyl^n, R^2 = Et; **22a**:**22b** = 78:22
23a+23b: R^1 = allyl, R^2 = Et; **23a**:**23b** = 78:22

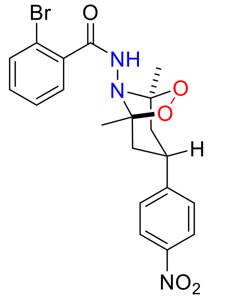

24

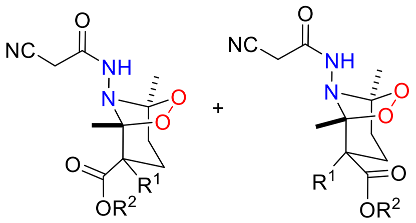

25a-28a **25b-28b**

25a+25b: R^1 = 4-chlorobenzyl, R^2 = Et; **25a**:**25b** = 93:7
26a+26b: R^1 = 4-fluorobenzyl, R^2 = Et; **26a**:**26b** = 92:8
27a+27b: R^1 = CH_2Ph, R^2 = Et; **27a**:**27b** = 92:8
28a+28b: R^1 = allyl, R^2 = allyl; **28a**:**28b** = 92:8

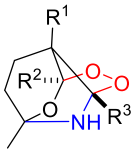

32-45

32: R^1 = allyl, R^2 = R^3 = CH_3
33: R^1 = hexyl^n, R^2 = R^3 = CH_3
34: R^1 = isopentyl, R^2 = R^3 = CH_3
35: R^1 = butyl, R^2 = R^3 = CH_3
36: R^1 = methyl, R^2 = R^3 = CH_3
37: R^1 = 4-bromobenzyl, R^2 = R^3 = CH_3
38: R^1 = benzyl, R^2 = R^3 = CH_3
39: R^1 =$CH_2CH_2C(O)OEt$, R^2 = R^3 = CH_3
40: R^1 = CH_2CH_2CN, R^2 = R^3 = CH_3
41: R^1 = ethyl, R^2 = R^3 = CH_3
42: R^1 = octyl^n, R^2 = R^3 = CH_3
43: R^1 = nonyl^n, R^2 = R^3 = CH_3
44: R^1 = 4-nitrobenzyl, R^2 = R^3 = CH_3
45: R^1 =H, R^2 = R^3 = ethyl

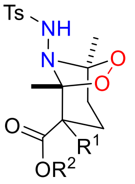

29, **30**

29: R^1 = 4-chlorobenzyl, R^2 = Et
30: R^1 = 4-fluorobenzyl, R^2 = Et

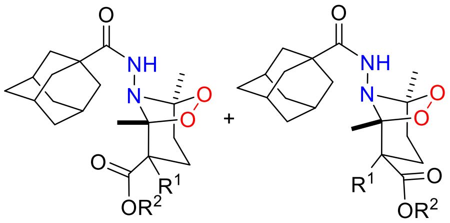

31a **31b**

31a+31b: R^1 = 4-chlorobenzyl, R^2 = Et; **31a**:**31b** = 90:10

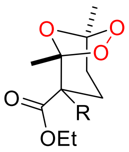

46: R = H
48: R = $CH_2C{\equiv}CH$
50: R = allyl
52: R = hexyl^n

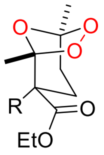

47: R = H
49: R = $CH_2C{\equiv}CH$
51: R = allyl

Synthesis of aminoperoxides 1a – 5a + 1b – 5b, 6, 7, 8a’ + 8a” + 8b’ + 8b”

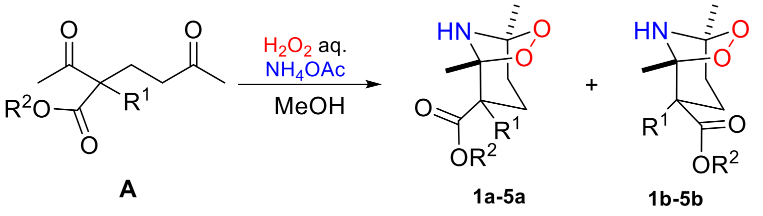

1a+1b: R^1 = 4-fluorobenzyl, R^2 = Et;
2a+2b: R^1 = dodecyl^n, R^2 = Et;
3a+3b: R^1 = allyl, R^2 = CH_2Ph;
4a+4b: R^1 = allyl, R^2 = 1-Adamantyl;
5a+5b: R^1 = allyl, R^2 = allyl;

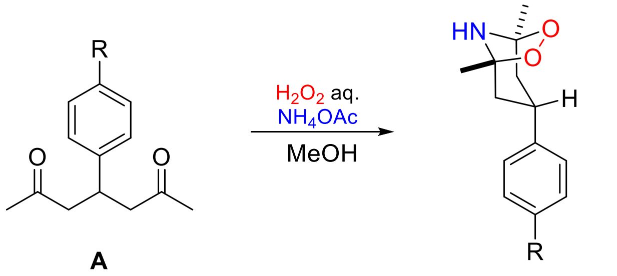

6: R = NO_2
7: R = Cl

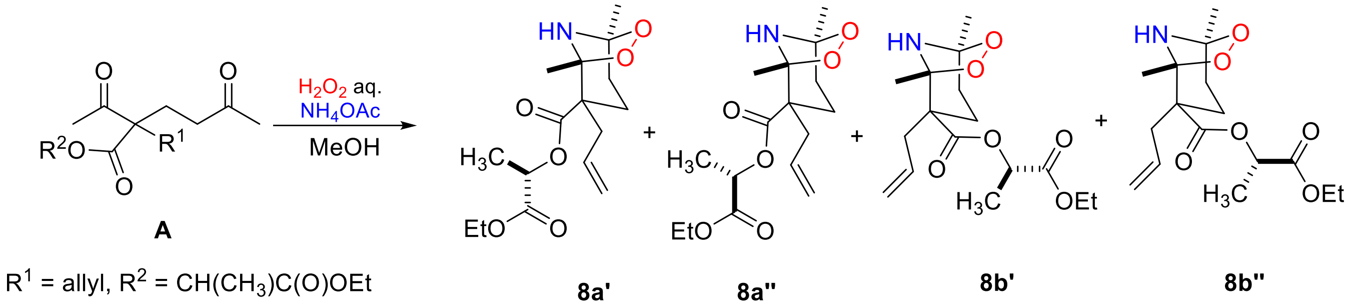

Aminoperoxides **1a – 5a + 1b – 5b, 6, 7, 8a’ + 8a” + 8b’ + 8b”** were synthesized according to the known procedure.[9] Aminoperoxides **3a + 3b, 4a + 4b, 5a + 5b, 6** and **7** are known compounds. Aminoperoxides **1a + 1b, 2a + 2b and 8a’ + 8a” + 8b’ + 8b”** are new compounds.

Procedure for the synthesis of aminoperoxides 1a + 1b, 2a + 2b and 8a’ + 8a” + 8b’ + 8b”

An ammonium acetate (0.340 – 0.370 g., 4.4 – 4.8 mmol, 5 mol of NH_4OAc / 1 mol of 1,5-diketone **A**) and 35 % aq. solution of H_2O_2 (0.128 – 0.140 g., 1.32 – 1.44 mmol, 1.5 mol of H_2O_2 / 1 mol of 1,5-diketone **A** were successively added with stirring to a solution of 1,5-diketone **A** (0.300 g, 0.88 – 0.96 mmol) in MeOH (10 mL) at 20 – 25 °C. The reaction mixture was stirred at 20 – 25°C for 1.5 hours. Then $CHCl_3$ (30 mL) and 5 % aq. NaOH (10 mL) were added. The organic layer was separated, the aqueous layer was extracted with $CHCl_3$ (3 × 30 mL). The combined organic phases were dried over $MgSO_4$. The solvent was removed in vacuum of a water jet pump at 20 – 25 °C. Mixtures of aminoperoxides **1a + 1b**, **1a + 1b** and **8a’ + 8a” + 8b’**

+ **8b**'' were isolated by chromatography on SiO_2 using 1 v/v % Et_3N in $CHCl_3$ mixture as the eluent. The ratio of aminoperoxides **8a**' + **8a**'' + **8b**' + **8b**'' was determined by the ^{1}H NMR spectroscopic data.

Aminoperoxides: **1a + 1b**: 0.286 g., 0.88 mmol, yield 91% (**1a** :**1b** = 66 : 34); **2a + 2b**: 0.275 g., 0.77 mmol, yield 88% (**2a** : **2b** = 78 : 22); **8a**' + **8a**'' + **8b**' + **8b**'': 0.282 g., 0.86 mmol, yield 90% (**8a**' **:** **8a**'' **:** **8b**' **:** **8b**'' = 45 : 37 : 11 : 7).

Ethyl 2-(4-fluorobenzyl)-1,5-dimethyl-6,7-dioxa-8-azabicyclo[3.2.1]octane-2-carboxylate, 1a+1b

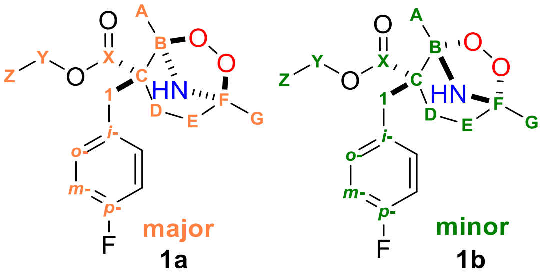

White crystals. Mp = 118-120 °C. R_f = 0.72 (TLC, PE : EA, 2 : 1). **1a : 1b** = 66 : 34.

1a: ^{1}H NMR (300.13 MHz, $CDCl_3$), δ: 1.22 (t, J = 7.1 Hz, 3H, ($\mathbf{H^Z}$)), 1.40 (s, 3H, ($\mathbf{H^G}$)), 1.56 – 1.74 (m, 2H, ($\mathbf{H^D}$, $\mathbf{H^E}$)), 1.77 (s, 3H, $\mathbf{H^A}$), 1.78 – 2.01 (m, 2H, ($\mathbf{H^D}$, $\mathbf{H^E}$)), 2.63 (d, J = 12.8 Hz, 1H, ($\mathbf{H^1}$)), 3.15 (br.s, 1H, (N**H**)), 3.28 (d, J = 12.8 Hz, 1H, ($\mathbf{H^1}$)), 4.15 (q, J = 7.1 Hz, 2H, ($\mathbf{H^Y}$)), 6.85 – 6.95 (m, 2H, ($\mathbf{H}^{o\text{-}}$)), 6.97 – 7.11 (m, 2H, ($\mathbf{H}^{m\text{-}}$)). ^{13}C NMR (75.48 MHz, $CDCl_3$), δ: 14.1 ($\mathbf{C^Z}$), 19.4 ($\mathbf{C^A}$), 21.1 ($\mathbf{C^G}$), 26.8 ($\mathbf{C^D}$), 34.1 ($\mathbf{C^E}$), 40.2 ($\mathbf{C^1}$), 54.8 ($\mathbf{C^C}$), 60.8 ($\mathbf{C^Y}$), 98.0 ($\mathbf{C^F}$), 100.5 ($\mathbf{C^B}$), 115.0 (d, $^2J_{CF}$ = 21.0 Hz, ($\mathbf{C}^{m\text{-}}$)), 131.3 (d, $^3J_{CF}$ = 7.9 Hz, ($\mathbf{C}^{o\text{-}}$)), 131.6 (d, $^4J_{CF}$ = 3.3 Hz, ($C^{i\text{-}}$)), 161.9 (d, $^1J_{CF}$ = 244.8 Hz, ($C^{p\text{-}}$)), 173.1 ($\mathbf{C^X}$).

2a: ^{1}H NMR (300.13 MHz, $CDCl_3$), δ: 1.19 (t, J = 7.1 Hz, 3H, ($\mathbf{H^Z}$)), 1.47 (s, 3H, ($\mathbf{H^G}$)), 1.49 – 1.53 (m, 1H, ($\mathbf{H^D}$)), 1.59 (s, 3H, ($\mathbf{H^A}$)), 1.74 – 1.92 (m, 2H, ($\mathbf{H^E}$)), 2.44 – 2.59 (m, 1H, ($\mathbf{H^D}$)), 3.10 (br.s, 1H, ($\mathbf{H^H}$)), 3.17 – 3.32 (m, 2H, ($\mathbf{H^1}$)), 4.11 (q, J = 7.1 Hz, 2H, ($\mathbf{H^Y}$)), 6.85 – 6.95 (m, 2H, ($\mathbf{H}^{o\text{-}}$)), 6.97 – 7.11 (m, 2H, ($\mathbf{H}^{m\text{-}}$)). ^{13}C NMR (75.48 MHz, $CDCl_3$), δ: 14.1 ($\mathbf{C^Z}$), 20.1 ($\mathbf{C^A}$), 21.2 ($\mathbf{C^G}$), 23.1 ($\mathbf{C^D}$), 32.5 ($\mathbf{C^E}$), 38.1 ($\mathbf{C^1}$), 52.2 ($\mathbf{C^C}$), 61.0 ($\mathbf{C^Y}$), 97.3 ($\mathbf{C^F}$), 101.1 ($\mathbf{C^B}$), 115.0 (d, $^2J_{CF}$ = 21.0 Hz, ($\mathbf{C}^{m\text{-}}$)), 131.3 (d, $^3J_{CF}$ = 7.9 Hz, ($\mathbf{C}^{o\text{-}}$)),132.3 (d, $^4J_{CF}$ = 3.3 Hz, ($C^{i\text{-}}$)), 162.4 (d, $^1J_{CF}$ = 244.8 Hz, ($C^{p\text{-}}$)), 173.0 ($\mathbf{C^X}$). Anal. Calcd. for $C_{17}H_{22}FNO_4$: C, 63.14; H, 6.86; F, 5.88; N, 4.33. Found: C, 63.28; H, 6.96; F, 5.97; N, 4.45. HRMS (ESI-TOF): m/z $[M+H]^+$: calculated for $[C_{17}H_{23}FNO_4]^+$: 324.1606; found: 324.1598.

Ethyl 2-decyl-1,5-dimethyl-6,7-dioxa-8-azabicyclo[3.2.1]octane-2-carboxylate, 2a + 2b

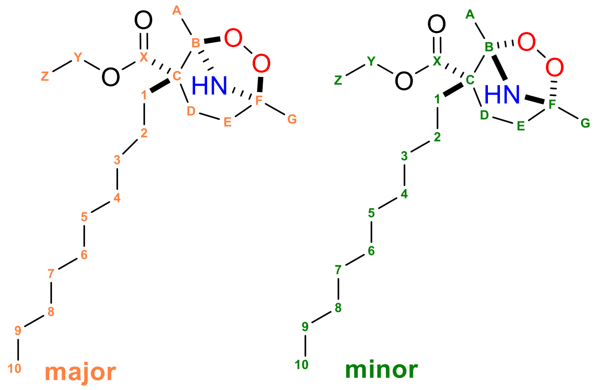

2a **2b**

Colorless oil. **2a : 2b** = 78 : 22. R_f = 0.65 (TLC, PE : EA, 2 : 1);

2a: ^{1}H NMR (300.13 MHz, $CDCl_3$), δ: 0.84 (t, *J* = 7.1 Hz, 3H, (**H^{10}**)), 0.88 – 1.00 (m, 1H, (**H^2**)), 1.14 -1.31 (m, 18H, **H^2**, **H^3**, **H^4**, **H^5**, **H^6**, **H^7**, **H^8**, **H^9**, **H^Z**), 1.38 (s, 3H, (**H^G**)), 1.42 – 1.48 (m, 1H, (**H^1**)), 1.63 (s, 3H, (**H^A**)), 1.67 – 1.88 (m, 3H, (**H^1**, **H^E**, **H^D**)), 1.95 – 2.18 (m, 2H, (**H^E**, **H^D**)), 3.07 (br.s, 1H, (N**H**)), 4.14 (t, *J* = 7.1 Hz, 2H, (**H^Y**)). ^{13}C NMR (75.48 MHz, $CDCl_3$), δ: 14.15 (**C^{10}**), 14.22 (**C^Z**), 18.5 (**C^A**), 21.2 (**C^G**), 22.7 (**C^9**), 23.9 (**C^2**), 26.7 (**C^D**), 29.4, 29.5, 29.6 (**C^3**), 30.1, 32.0 (**C^4**), 34.2 (**C^E**), 35.1 (**C^1**), 53.8 (**C^C**), 60.5 (**C^Y**), 97.8 (**C^F**), 100.6 (**C^B**), 173.8 (**C^X**).

2b: ^{1}H NMR (300.13 MHz, $CDCl_3$), δ: 0.84 (t, *J* = 7.1 Hz, 3H, (**H^{10}**)), 0.98 – 1.11 (m, 1H, (**H^2**)), 1.14 -1.31 (m, 18H, **H^2**, **H^3**, **H^4**, **H^5**, **H^6**, **H^7**, **H^8**, **H^9**, **H^Z**), 1.40 (s, 3H, (**H^G**)), 1.50 (s, 3H, (**H^A**)), 1.66 – 1.88 (m, 5H, (**H^E, H^D, H^1**)), 2.50-2.67 (m, 1H, (**H^D**)), 2.95 (br. s, 1H, (**NH**)), 4.11 (t, *J* = 7.1 Hz, 2H, (**H^Y**)). ^{13}C NMR (75.48 MHz, $CDCl_3$), δ: 14.15 (**C^{10}**), 14.22 (**C^Z**), 20.0 (**C^A**), 21.2 (**C^G**), 22.5 (**C^9**), 25.2 (**C^2**), 29.5, 29.6 (**C^3**), 30.3, 32.0 (**C^4**), 32.5 (**C^E**), 33.1 (**C^1**), 54.1 (**C^C**), 60.7 (**C^Y**), 97.1 (**C^F**), 101.1 (**C^B**), 173.6 (**C^X**). Anal. Calcd. for $C_{20}H_{37}NO_4$: C, 67.57; H, 10.49; N, 3.94. Found: C, 67.70; H, 10.62; N, 4.10. HRMS (ESI-TOF): m/z $[M+H]^+$: calculated for $[C_{20}H_{38}NO_4]^+$: 356.2795; found: 356.2795.

Benzyl 2-allyl-1,5-dimethyl-6,7-dioxa-8-azabicyclo[3.2.1]octane-2-carboxylate, 3a + 3b[9]

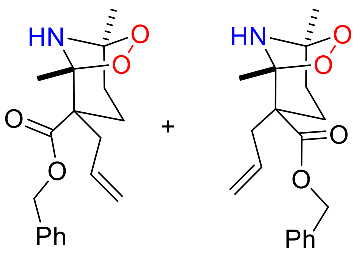

3a **3b**

Slightly yellow oil. **3a : 3b** = 55 : 45.

^{1}H NMR (300.13 MHz, $CDCl_3$), δ: 1.44 (s, 1.65H), 1.47 (s, 1.35H), 1.51 (s, 1.35H), 1.66 (s, 1.65H), 1.69 – 1.84 (m, 2H), 1.88 – 2.31 (m, 2H), 2.58 – 2.76 (m, 2H), 3.05 (br.s, 0.45H), 3.16 (br. s, 0.55H), 4.98 – 5.13 (m, 2H), 5.16 – 5.24 (m, 2H), 5.52 – 5.72 (m, 1H), 7.31 – 7.41 (m, 5H). ^{13}C NMR (75.48 MHz, $CDCl_3$), δ: 19.4,

19.7, 21.1, 21.1, 23.7, 26.8, 32.0, 34.0, 37.6, 39.8, 53.7, 53.9, 66.6, 66.7, 97.1, 97.9, 100.2, 100.6, 118.4, 118.6, 128.1, 128.2, 128.5, 128.6, 128.7, 132.6, 134.1, 135.9, 172.9, 173.0. Anal. Calcd. for $C_{18}H_{23}NO_4$: C, 68.12; H, 7.30; N, 4.41. Found: C, 68.04; H, 7.38; N, 4.32. HRMS (ESI-TOF): m/z $[M+H]^+$: calculated for $[C_{18}H_{24}NO_4]^+$: 318.1700; found: 318.1698.

Adamantan-1-yl 2-allyl-1,5-dimethyl-6,7-dioxa-8-azabicyclo[3.2.1]octane-2-carboxylate, 4a +4b[9]

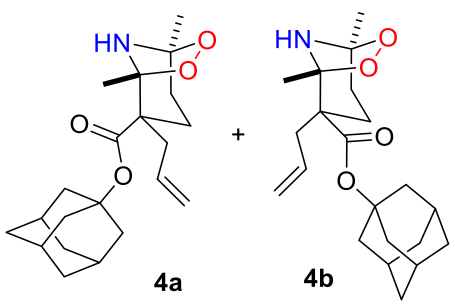

White crystals. Mp = 77 – 79°C. (Lit.[9] Mp = 77 – 79 °C). **4a : 4b** = 50 : 50;

^{1}H NMR (300.13 MHz, $CDCl_3$), δ: 1.42 (s, 1.5H), 1.42 (s, 1.5H), 1.55 (s, 1.5H), 1.56 – 1.91 (m, 10.0H), 1.98 – 2.23 (m, 10.5H), 2.49 – 2.67 (m, 2.0H), 2.98 (br.s, 0.5H), 3.10 (br. s, 0.5H), 5.01 – 5.13 (m, 2H), 5.57 – 5.77 (m, 1H). ^{13}C NMR (75.48 MHz, $CDCl_3$), δ: 19.4, 19.9, 21.1, 23.8, 27.0, 30.8, 32.2, 34.0, 36.2, 37.7, 39.8, 41.3, 41.4, 53.6, 53.8, 81.0, 81.2, 97.0, 97.9, 100.5, 100.6, 118.0, 118.2, 133.0, 134.6, 171.6, 171.7. Anal. Calcd. for $C_{21}H_{31}NO_4$: C, 69.78; H, 8.64; N, 3.87. Found: C, 69.72; H, 8.58; N, 3.93. HRMS (ESI-TOF): m/z [M+Na]+: calculated for $[C_{21}H_{31}NNaO_4]$+: 384.2145; found: 384.2139.

Allyl 2-allyl-1,5-dimethyl-6,7-dioxa-8-azabicyclo[3.2.1]octane-2-carboxylate, 5a + 5b[9]

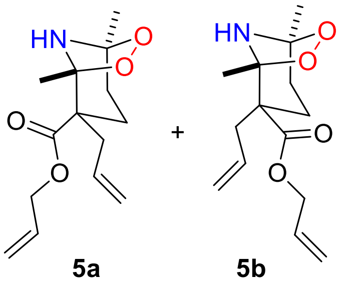

Slightly yellow oil. **5a : 5b** = 55 : 45. R_f = 0.75 (TLC, PE : EA, 2 : 1);

^{1}H NMR (300.13 MHz, $CDCl_3$), δ: 1.44 (s, 1.65H), 1.46 (s, 1.35H), 1.54 (s, 1.35H), 1.70 (s, 1.65H), 1.67 – 2.31 (m, 4H), 2.57 – 2.74 (m, 2H), 3.04 (br.s, 0.45H), 3.14 (br. s, 0.55H), 4.63 (d, J = 5.8 Hz, 2H), 5.03 – 5.14 (m, 2H), 5.20 – 5.41 (m, 2H), 5.56 – 5.74 (m, 1H), 5.85 – 6.00 (m, 1H). ^{13}C NMR (75.48 MHz, $CDCl_3$), δ: 19.4, 19.7, 21.1, 21.1, 23.7, 26.8, 32.0, 34.0, 37.6, 39.7, 53.6, 53.9, 65.4, 65.5, 97.1, 97.9, 100.1, 100.5, 118.2, 118.3, 118.4, 118.5, 132.0, 132.1, 132.7, 134.2, 172.8. Anal. Calcd. for $C_{14}H_{21}NO_4$: C, 62.90; H, 7.92; N, 5.24. Found: C, 62.99; H, 7.82; N, 5.36. HRMS (ESI-TOF): m/z $[M+H]^+$: calculated for $[C_{14}H_{22}NO_4]^+$: 268.1543; found: 268.1542.

1,5-Dimethyl-3-(4-nitrophenyl)-6,7-dioxa-8-azabicyclo[3.2.1]octane, 6[9]

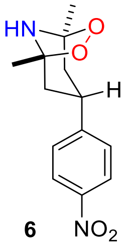

6

White crystals. Mp = 132 – 134° C (Lit.[9] Mp = 132 – 134 °C). R_f = 0.59 (TLC, PE : EA, 1 : 1).

^{1}H NMR (300.13 MHz, $CDCl_3$), δ: 1.51 (s, 6H), 1.83 – 1.97 (m, 2H), 2.08 (dd, *J* = 13.2, 5.8 Hz, 2H), 3.30 (br.s, 1H), 3.64 – 3.83 (m, 1H), 7.40 (d, *J* = 8.4 Hz, 2H), 8.15 (d, *J* = 8.4 Hz, 2H).^{13}C NMR (75.48 MHz, $CDCl_3$), δ: 21.6, 36.7, 41.9, 97.3, 123.9, 128.0, 146.7, 151.7. Anal. Calcd. for $C_{13}H_{16}N_2O_4$: C, 59.08; H, 6.10; N, 10.60. Found: C, 59.18; H, 6.19; N, 10.75. HRMS (ESI-TOF): m/z $[M+H]^+$: calculated for $[C_{13}H_{17}N_2O_4]^+$: 265.1183; found: 265.1187.

1,5-Dimethyl-3-(4-chlorophenyl)-6,7-dioxa-8-azabicyclo[3.2.1]octane, 7[9]

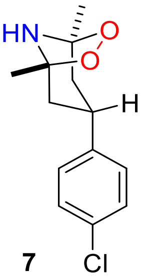

7

White crystals. Mp = 94 – 95°C (Lit.[9] Mp = 94 – 95 °C). R_f = 0.29 (TLC, PE : EA, 2 : 1).

^{1}H NMR (300.13 MHz, $CDCl_3$), δ: 1.52 (s, 6H), 1.81 – 1.94 (m, 2H), 2.07 (dd, *J* = 13.2, 5.8 Hz, 2H), 3.23 (br.s., 1H), 3.52 – 3.58 (m 1H), 7.20 (d, *J* = 8.5 Hz, 2H), 7.27 (d, *J* = 8.5 Hz, 2H). ^{13}C NMR (75.48 MHz, $CDCl_3$), δ: 21.7, 36.1, 42.3, 97.4, 128.5, 128.7, 132.1, 142.5. Anal. Calcd. for $C_{13}H_{16}ClNO_2$: C, 61.54; H, 6.36; Cl, 13.97; N, 5.52. Found: C, 61.65; H, 6.48; Cl, 14.09; N, 5.65. HRMS (ESI-TOF): m/z $[M+H]^+$: calculated for $[C_{13}H_{17}ClNO_2]^+$: 254.0942; found: 254.0947.

1-ethoxy-1-oxopropan-2-yl -2-allyl-1,5-dimethyl-6,7-dioxa-8-azabicyclo[3.2.1]octane-2-carboxylate, 8a', 8a'', 8b', 8b"

8a'

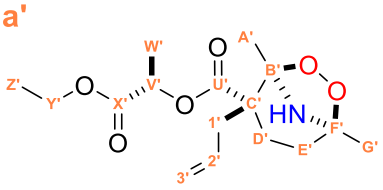

8b'

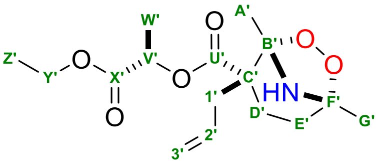

8a''

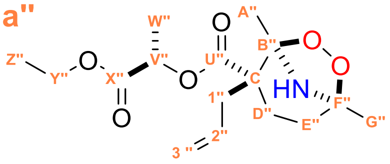

8b''

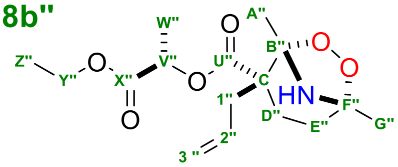

Colorless oil. **8a**' : **8a**'' : **8b**' : **8b**'' = 45 : 37 : 11 : 7. R_f= 0.46 (TLC, PE : EA, 2 : 1).

8a' + 8a'': ^{1}H NMR (300.13 MHz, $CDCl_3$), δ: 1.26 (t, *J* = 7.1 Hz, 3H, (**H**$^{Z'}$ + **H**$^{Z''}$)), 1.41 (s, 3H, (**H**$^{W'}$ + **H**$^{W''}$)), 1.47 and 1.49 (s, 3H, (**H**$^{G'}$ + **H**$^{G''}$)), 1.67 and 1.70 (s, 3H, (**H**$^{A'}$ + **H**$^{A''}$)), 1.70 – 2.17 (m, 4H, (**H**$^{D'}$ + **H**$^{D''}$, **H**$^{E'}$ + **H**$^{E''}$)), 2.71 (dd, *J* = 13.3, 8.9Hz, 1H, (**H**$^{1'}$ + **H**$^{1''}$)), 2.21 (dd, *J* = 13.3, 8.9 Hz, 1H, (**H**$^{1'}$ + **H**$^{1''}$)), 3.17 (br.s, 1H, N**H**), 4.18 (q, *J* = 7.1 Hz, 2H, (**H**$^{Y'}$ + **H**$^{Y''}$)), 5.01 – 5.21 (m, 2H, (**H**$^{3'}$ + **H**$^{3''}$)), 5.08 – 5.23 (m, 2H, (**H**$^{V'}$ + **H**$^{V''}$)), 5.61 – 5.80 (m, 1H, (**H**$^{2'}$ + **H**$^{2''}$)). ^{13}C NMR (75.48 MHz, $CDCl_3$), δ: 14.2 (**C**$^{Z'}$ + **C**$^{Z''}$), 17.12 and 17.15 (**C**$^{W'}$ + **C**$^{W''}$), 19.1 and 19.5 (**C**$^{A'}$ + **C**$^{A''}$), 21.1 and 21.2 (**C**$^{G'}$ + **C**$^{G''}$), 26.9 and 27.0 (**C**$^{D'}$ + **C**$^{D''}$), 33.7 and 34.0 (**C**$^{E'}$ + **C**$^{E''}$), 39.6 and 39.9 (**C**$^{1'}$ + **C**$^{1''}$), 53.1 and 53.2 (**C**$^{C'}$ + **C**$^{C''}$), 61.49 and 61.53 (**C**$^{Y'}$ + **C**$^{Y''}$), 68.8 (**C**$^{V'}$ + **C**$^{V''}$), 98.0 and 98.1 (**C**$^{F'}$ + **C**$^{F''}$), 100.15 and 100.21 (**C**$^{B'}$ + **C**$^{B''}$), 118.71 and 117.74 (**C**$^{3'}$ + **C**$^{3''}$), 132.67 and 132.71 (**C**$^{2'}$ + **C**$^{2''}$), 170.71 and 170.75 (**C**$^{X'}$ + **C**$^{X''}$), 172.5 and 172.66 (**C**$^{U'}$ + **C**$^{U''}$).

8b' + 8b'': ^{1}H NMR (300.13 MHz, $CDCl_3$), δ: 1.24 (t, *J* = 7.1 Hz, 3H, (**H**$^{Z'}$+ **H**$^{Z''}$)), 1.41 (s, 3H, (**H**$^{W'}$ + **H**$^{W''}$)), 1.47 and 1.49 (s, 3H, (**H**$^{G'}$ + **H**$^{G''}$)), 1.52 and 1.60 (s, 3H, (**H**$^{A'}$ + **H**$^{A''}$)), 1.71 – 1.81 (m, 3H, (**H**$^{D'}$ + **H**$^{D''}$ + **H**$^{E'}$ + **H**$^{E''}$)), 2.58 – 2.75 (m, 1H, (**H**$^{D'}$ + **H**$^{D''}$, **H**$^{1'}$ + **H**$^{1''}$)), 3.08 (br. s, 1H, N**H**), 4.18 (q, *J* = 7.1 Hz, 2H, (**H**$^{Y'}$ + **H**$^{Y''}$)), 4.98 – 5.12 (m, 2H, (**H**$^{3'}$ + **H**$^{3''}$)), 5.00 – 5.10 (m, 2H, (**H**$^{V'}$ + **H**$^{V''}$)), 5.61 – 5.84 (m, 1H, (**H**$^{2'}$ + **H**$^{2''}$)). ^{13}C NMR (75.48 MHz, $CDCl_3$), δ: 14.2 (**C**$^{Z'}$ + **C**$^{Z''}$), 17.00 and 17.02 (**C**$^{W'}$ + **C**$^{W''}$), 19.45 and 19.6 (**C**$^{A'}$ + **C**$^{A''}$), 21.1 and 21.2 (**C**$^{G'}$ + **C**$^{G''}$), 23.5 and 23.8 (**C**$^{D'}$ + **C**$^{D''}$), 31.8 (**C**$^{E'}$ + **C**$^{E''}$), 37.4 and 37.5 (**C**$^{1'}$ + **C**$^{1''}$), 53.1 and 53.2 (**C**$^{C'}$ + **C**$^{C''}$), 61.49 and 61.53 (**C**$^{Y'}$ + **C**$^{Y''}$), 69.2 and 69.4 (**C**$^{V'}$ + **C**$^{V''}$), 97.2 and 97.3 (**C**$^{F'}$ + **C**$^{F''}$), 100.5 and 100.7 (**C**$^{B'}$ + **C**$^{B''}$), 118.5 and 118.6 (**C**$^{3'}$ + **C**$^{3''}$), 134.1 and 134.5 (**C**$^{2'}$ + **C**$^{2''}$), 170.75 and 170.79 (**C**$^{X'}$ + **C**$^{X''}$), 172.7 and 172.8 (**C**$^{U'}$ + **C**$^{U''}$). Anal. Calcd for $C_{16}H_{25}NO_6$: C, 58.70; H, 7.70; N, 4.28. Found: C, 58.88; H, 7.90; N, 4.35. HRMS (ESI-TOF): m/z $[M+H]^+$: calculated for$[C_{16}H_{26}NO_6]^+$: 328.1755; found: 328.1758.

Synthesis of aminoperoxides 9a – 11a, 12a + 12b, 13a and 14a

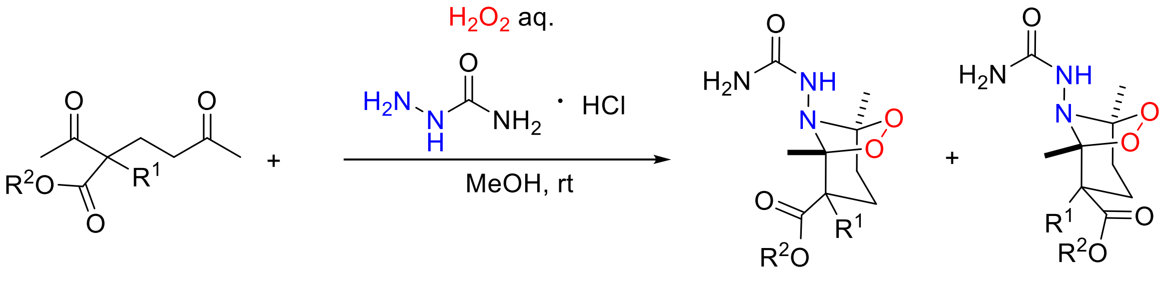

A **9a-14a** **12b**

9a: R^1 = 4-chlorobenzyl, R^2 = Et;
10a: R^1 = butyl^n, R^2 = Et;
11a: R^1 = $CH_2CH_2C(O)OEt$, R^2 = Et;
12a+12b: R^1 = 4-fluorobenzyl, R^2 = Et;
13a: R^1 = allyl, R^2 = allyl
14a: R^1 = CH_2Ph, R^2 = Et

Aminoperoxides **9a – 11a, 12a + 12b, 13a and 14a** were synthesized according to a known procedure.[10]

Aminoperoxides **9a, 10a and 13a** are known compounds. Aminoperoxides **11a**, **12a + 12b and 14a** are new compounds.

Procedure for the synthesis of aminoperoxides 11a, 12a+12b and 14a.

A 1,5-diketone **A** (0.200 g., 0.62 – 0.68 mmol) and 35 % aq. solution of H_2O_2 (108– 118 μL, 1.24 – 1.36 mmol, 2.0 mol of H_2O_2 / 1.0 mol of **A**) were successively added with stirring to a solution of semicarbazide hydrochloride (0.207– 0.227 g., 1.86 – 2.04 mmol, 3.0 mol. of semicarbazide hydrochloride / 1.0 mol of **A**) in MeOH (18-24 mL until the semicarbazide is dissolved in the minimum amount of solvent). The reaction mixture was stirred at 20 – 25°C for 6h. Then water (25 mL) was added, and the reaction mixture was stirred for another 15 minutes.

In the case of **12a+12b** and **14a**: The resulting precipitate was filtered, washed with water and then with petroleum ether. Pure aminoperoxides **12a+12b** and **14a** were obtained.

In the case of **11a**: Then $CHCl_3$ (40 mL) was added to the reaction mixture. The organic phase was separated; the aqueous phase was washed by $CHCl_3$ (2×30 mL). The combined organic phases were dried over $MgSO_4$ and filtered. The solvent was removed in the vacuum of a membrane pump. The crude product was dissolved in 3 ml of $CHCl_3$ and added dropwise to 60 ml of petroleum ether in an ultrasonic bath. The precipitated white crystals of **11a** were filtered and washed with petroleum ether. Pure aminoperoxide **11a** was obtained.

Compounds: **11a:** 0.164 g, 0.44 mmol, yield 66%; **12a+12b:** 0.150 g, 0.40 mmol, yield 61% (**12a: 12b** = 95 : 5); **14a:** 0.162 g, 0.45 mmol, yield 65%.

Ethyl 2-(4-chlorobenzyl)-1,5-dimethyl-8-ureido-6,7-dioxa-8-azabicyclo[3.2.1]octane-2-carboxylate, 9a[10]

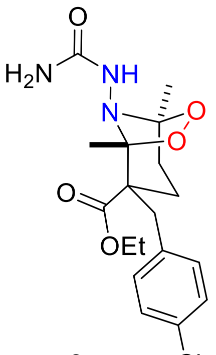

9a

White crystals. Mp = 131 – 133 °C (Lit.[10] Mp = 131 – 133 °C).

^{1}H NMR (300.13 MHz, $CDCl_3$), δ: 1.28 (t, *J* = 7.2 Hz, 3H), 1.43 (s, 3H), 1.51 – 1.65 (m, 1H), 1.76 (s, 3H), 1.80 – 1.98 (m, 2H), 2.09 – 2.15 (m, 1H), 2.65 (d, *J* = 12.8 Hz, 1H), 3.29 (d, *J* = 12.8 Hz, 1H), 4.07 – 4.25 (m, 2H), 5.37 (br.s., 2H), 6.97 (d, *J* = 8.4 Hz, 2H), 7.03 (br.s., 1H), 7.22 (d, *J* = 8.4 Hz, 2H). ^{13}C NMR (75.48 MHz, $CDCl_3$), δ: 14.2, 15.2, 18.3, 26.3, 35.5, 40.9, 57.0, 61.4, 101.3, 104.6, 128.5, 128.7, 131.3, 131.4, 133.0,

134.6, 159.7, 173.3. Anal. Calcd. for $C_{18}H_{24}ClN_3O_5$: C, 54.34; H, 6.08; Cl, 8.91; N, 10.56. Found: C, 54.48; H, 6.20; Cl, 8.98; N, 10.65. HRMS (ESI-TOF): m/z $[M+Na]^+$: calculated for $[C_{18}H_{24}ClN_3NaO_5]^+$: 420.1297; found: 420.1293.

Ethyl 2-butyl-1,5-dimethyl-8-ureido-6,7-dioxa-8-azabicyclo[3.2.1]octane-2-carboxylate, 10a[10]

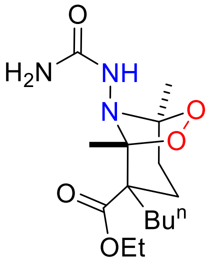

10a

White crystals. Mp = 135 – 137 °C (Lit.[10] Mp = 135 – 137 °C).

^{1}H NMR (300.13 MHz, $CDCl_3$), δ: 0.86 (t, J = 7.2 Hz, 3H), 0.93 – 1.08 (m, 1H), 1.20 – 1.34 (m, 3H), 1.27 (t, J = 7.2 Hz, 3H), 1.39 – 1.55 (m, 1H), 1.42 (s, 3H), 1.62 (s, 3H), 1.73 – 1.94 (m, 3H), 2.02 – 2.22 (m, 2H), 4.08 – 4.23 (m, 2H), 5.38 (br.s., 2H), 6.79 (br.s., 1H). ^{13}C NMR (75.48 MHz, $CDCl_3$), δ: 14.0, 14.3, 15.1, 18.4, 23.2, 26.2, 26.4, 35.5, 35.7, 56.3, 61.1, 101.1, 104.8, 159.7, 174.2. Anal. Calcd. for $C_{15}H_{27}N_3O_5$: C, 54.70; H, 8.26; N, 12.76. Found: C, 54.87; H, 8.36; N, 12.85. HRMS (ESI-TOF): m/z $[M+Na]^+$: calculated for $[C_{15}H_{27}N_3NaO_5]^+$: 352.1843; found: 352.1838.

Ethyl 2-(3-ethoxy-3-oxopropyl)-1,5-dimethyl-8-ureido-6,7-dioxa-8-azabicyclo[3.2.1]octane-2-carboxylate, 11a

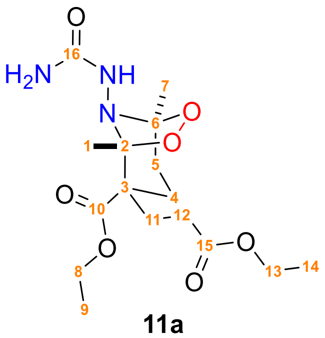

11a

White crystals. Mp = 118 – 120 °C. R_f= 0.57 (TLC, $CHCl_3$:MeOH, 10 : 1).

^{1}H NMR (300.13 MHz, $CDCl_3$), δ: 1.24 (t, J = 7.2 Hz, 3H, (**H^9** or **H^{14}**)) 1.26 (t, J = 7.1 Hz, 3H, (**H^9** or **H^{14}**)), 1.43 (s, 3H, (**H^7**)), 1.65 (s, 3H, (**H^1**)), 1.73 – 2.41 (m, 8H, (**H^4**, **H^5**, **H^{11}**, **H^{12}**)), 4.11 (q, J = 7.2 Hz, 2H, (**H^8** or **H^{13}**)), 4.16 (q, J = 7.2 Hz, 2H, (**H^8** or **H^{13}**)), 5.43 (br.s., 2H, N**H$_2$**), 6.64 (br.s., 1H, N**H**). ^{13}C NMR (75.48 MHz, $CDCl_3$), δ: 14.24 (**C^9** or **C^{14}**), 14.29 (**C^9** or **C^{14}**), 15.2 (**C^1**), 18.3 (**C^7**), 25.9 (**C^4**), 29.4 (**C^{12}**), 30.6 (**C^{11}**), 35.6 (**C^5**), 55.5 (**C^3**), 60.8 (**C^8** or **C^{13}**), 61.5 (**C^8** or **C^{13}**), 101.0 (**C^6**), 104.5 (**C^2**), 159.5 (**C^{16}**), 172.8 (**C^{10}** or **C^{15}**), 173.5 (**C^{10}** or **C^{15}**). ^{15}N (40.56 MHz, $CDCl_3$), δ: 71.8 (**NH$_2$**), 114.6 (**NH**), 139.5 (**N**). Anal. Calcd. for

$C_{16}H_{27}N_3O_7$: C, 51.47; H, 7.29; N, 11.25. Found: C, 51.60; H, 7.39; N, 11.37. HRMS (ESI-TOF): m/z $[M+Na]^+$: calculated for$[C_{16}H_{27}N_3NaO_7]^+$: 396.1741; found: 396.1732.

Ethyl 2-(4-fluorobenzyl)-1,5-dimethyl-8-ureido-6,7-dioxa-8-azabicyclo[3.2.1]octane-2-carboxylate, 12a + 12b

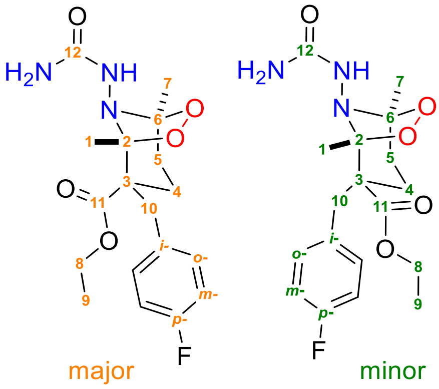

major **12a** minor **12b**

White crystals. Mp = 137 – 139 °C. **12a : 12b** = 95 : 5. R_f= 0.67 (TLC, $CHCl_3$:MeOH, 10 : 1).

12a: ^{1}H NMR (300.13 MHz, $CDCl_3$), δ: 1.27 (t, J = 7.2 Hz, 3H, (**H^9**)), 1.43 (s, 3H, (**H^7**)), 1.55 – 1.68 (m, 1H, (**H^4**)), 1.77 (s, 3H, (**H^1**)), 1.82 – 1.98 (m, 2H, (**H^4, H^5**)), 2.03 – 2.17 (m, 1H, (**H^5**)), 2.66 (d, J = 13.0 Hz, 1H, (**H^{10}**)), 3.30 (d, J = 13.0 Hz, 1H, (**H^{10}**)), 4.00 – 4.27 (m, 2H, (**H^8**)), 5.32 (br.s., 2H, (**N**H_2)), 6.86 (br.s., 1H, (N**H**)), 6.86 – 7.03 (m, 4H, (**H^{m-}**, **H^{o-}**)). ^{13}C NMR (75.48 MHz, $CDCl_3$), δ: 14.2 (**C^9**), 15.2 (**C^1**), 18.3 (**C^7**), 26.3 (**C^4**), 35.5 (**C^5**), 40.8 (**C^{10}**), 57.1 (**C^3**), 61.3 (**C^8**), 101.3 (**C^6**), 104.6 (**C^2**), 115.2 (d, $^2J_{CF}$ = 21.0 Hz, (**C^{m-}**)), 131.3 (d, $^3J_{CF}$ = 7.9 Hz, (**C^{o-}**)), 131.6 (d, $^4J_{CF}$ = 3.8 Hz, (C^{i-})), 159.5 (**C^{12}**), 162.1 (d, $^1J_{CF}$ = 244.8 Hz, (C^{p-})), 173.4 (**C^{11}**). ^{15}N NMR (40.56 MHz, $CDCl_3$), δ: 71.3 (**N**H_2), 114.9 (**N**H), 139.8 (**N**).

12b: ^{1}H NMR (300.13 MHz, $CDCl_3$), δ: 5.74 (br.s., 2H, (N**H**$_2$)), 7.71 (br.s., 1H, (N**H**)). ^{15}N NMR (40.56 MHz, $CDCl_3$), δ: 72.8 (**N**H_2), 119.8 (**N**H). Anal. Calcd. for $C_{18}H_{24}FN_3O_5$: C, 56.68; H, 6.34; F, 4.98; N, 11.02. Found: C, 56.80; H, 6.45; F, 5.05; N, 11.16. HRMS (ESI-TOF): m/z $[M+Na]^+$: calculated for$[C_{18}H_{24}FN_3NaO_5]^+$: 404.1592; found: 404.1587.

Allyl 2-allyl-1,5-dimethyl-8-ureido-6,7-dioxa-8-azabicyclo[3.2.1]octane-2-carboxylate, 13a[10]

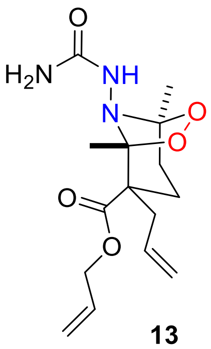

13

White crystals. Mp = 115 – 117 °C (Lit.[10] Mp = 115 – 117 °C).

^{1}H NMR (300.13 MHz, $CDCl_3$), δ: 1.44 (s, 3H), 1.65 (s, 3H), 1.83 – 2.32 (m, 5H), 2.63 (dd, *J* = 13.5, 6.8 Hz, 1H), 4.51 – 4.70 (m, 2H), 4.19 – 5.16 (m, 2H), 5.21 – 5.40 (m, 3H), 5.53 – 5.72 (m, 2H), 5.79 – 6.01 (m, 1H), 6.66 (br.s., 1H). ^{13}C NMR (75.48 MHz, $CDCl_3$), δ: 15.2, 18.4, 26.6, 35.6, 40.3, 56.2, 66.0, 101.2, 104.4, 119.2, 119.7, 131.7, 132.4, 159.5, 173.3. Anal. Calcd. for $C_{15}H_{23}N_3O_5$: C, 55.37; H, 7.13; N, 12.92. Found: C, 55.49; H, 7.26; N, 13.02. HRMS (ESI-TOF): m/z [M+Na]$^+$: calculated for $[C_{15}H_{23}N_3NaO_5]^+$: 348.1530; found: 348.1529.

Ethyl 2-benzyl-1,5-dimethyl-8-ureido-6,7-dioxa-8-azabicyclo[3.2.1]octane-2-carboxylate, 14a

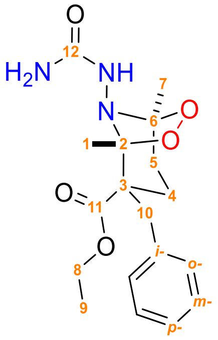

14

White crystals. Mp = 135 – 137 °C. R_f= 0.44 (TLC, $CHCl_3$:MeOH, 10 : 1).

^{1}H NMR (300.13 MHz, $CDCl_3$), δ: 1.29 (t, *J* = 7.2 Hz, 3H, (**H^9**)), 1.46 (s, 3H, (**H^7**)), 1.64 (dd, *J* = 12.8, 5.5 Hz, 1H, (**H^4**)), 1.82 (s, 3H, (**H^1**)), 1.84 – 2.20 (m, 3H, (**H^4, H^5**)), 2.70 (d, *J* = 13.0 Hz, 1H, (**H^{10}**)), 3.36 (d, *J* = 13.0 Hz, 1H, (**H^{10}**)), 4.12 – 4.30 (m, 2H, (**H^8**)), 5.60 (br.s., 2H, (N**H**$_2$)), 6.86 (br.s., 1H, (N**H**)), 7.00 – 7.12 (m, 2H, (**H^{o-}**)), 7.19 – 7.35 (m, 3H, (**H^{m-}**, **H^{p-}**)). ^{13}C NMR (75.48 MHz, $CDCl_3$), δ: 14.2 (**C^9**), 15.3 (**C^1**), 18.4 (**C^7**), 26.3 (**C^4**), 35.6 (**C^5**), 41.6 (**C^{10}**), 57.1 (**C^3**), 61.3 (**C^8**), 101.3 (**C^6**), 104.8 (**C^2**), 127.1 (**C^{p-}**), 128.4 (**C^{m-}**), 130.0 (**C^{o-}**), 136.1(**C^{i-}**), 159.3 (**C^{12}**), 173.6 (**C^{11}**). ^{15}N NMR (40.56 MHz, $CDCl_3$), δ: 71.2 (**N**H_2), 114.8 (**N**H), 139.9 (**N**). Anal. Calcd. for $C_{18}H_{25}N_3O_5$: C, 59.49; H, 6.93; N, 11.56. Found: C, 59.60; H, 7.05; N, 11.68. HRMS (ESI-TOF): m/z [M+Na]$^+$: calculated for$[C_{18}H_{25}N_3NaO_5]^+$: 386.1686; found: 386.1677.

Synthesis of aminoperoxides 15a-23a + 15b-23b and 24

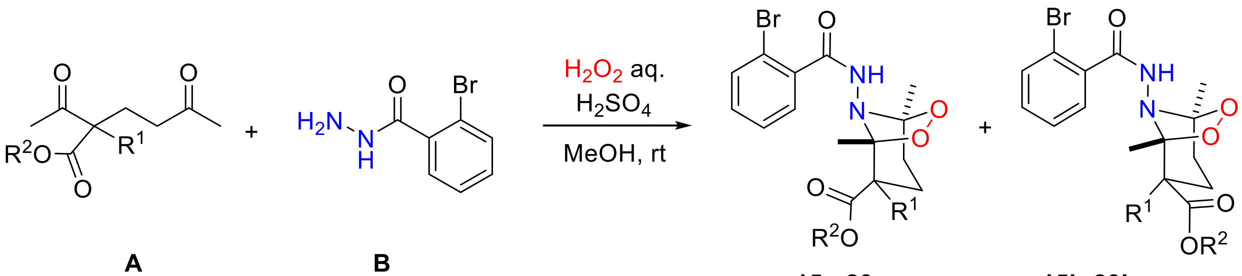

A **B** **15a-23a** **15b-23b**

15a+15b: R^1 = 4-chlorobenzyl, R^2 = Et;
16a+16b: R^1 = Et, R^2 = Et;
17a+17b: R^1 = allyl, R^2 = allyl;
18a: R^1 = 4-bromobenzyl, R^2 = Bu^t;
19a+19b: R^1 = 4-fluorobenzyl, R^2 = Et;
20a+20b: R^1 = CH_2Ph, R^2 = Et;
21a+21b: R^1 = allyl, R^2 = CH_2Ph;
22a+22b: R^1 = $butyl^n$, R^2 = Et;
23a+23b: R^1 = allyl, R^2 = Et

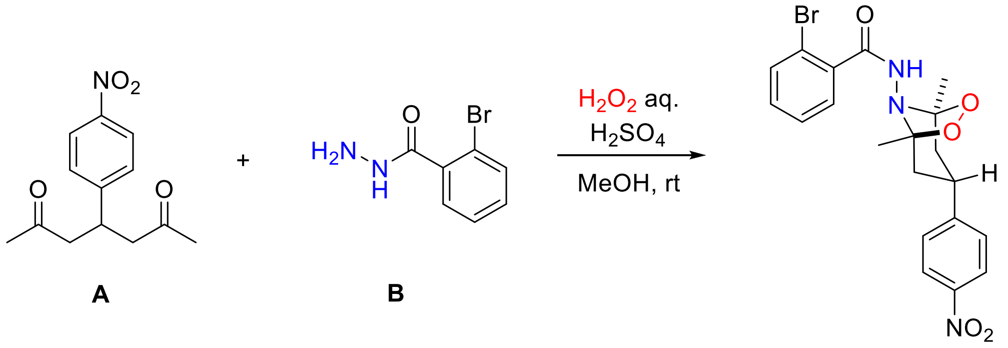

A **B**

24

Aminoperoxides **15a-23a + 15b-23b, 24** were synthesized according to a known procedure.[10] Aminoperoxides **15a+15b, 17a+17b, 22a+22b and 24** are known compounds.[10] Aminoperoxides **16a+16b, 18a, 19a-21a** + **19b-21b** and **23a+23b** are new compounds.

Procedure for the synthesis of aminoperoxides 16a+16b, 18a, 19a-21a + 19b-21b and 23a+23b

A 98% H_2SO_4 (54 μL – 95 μL, 1.00 – 1.76 mmol, 2.0 mol of H_2SO_4 / 1.0 mol of **A**), 2-bromobenzohydrazide **B** (0.108 – 0.188 g, 0.50 – 0.88 mmol, 1.0 mol of **B** / 1.0 mol of **A**) and 35 % aq. solution of H_2O_2 (65 – 114 μL, 0.75 – 1.32 mmol, 1.5 mol of H_2O_2 / 1.0 mol of **A**) were successively added with stirring to a solution of 1,5-diketone **A** (0.200 g, 0.50 – 0.88 mmol) in MeOH (1–2 mL). The reaction mixture was stirred at 20 - 25 °C for 0.5 h in the case of **19a+19b, 20a+20b** and **23a+23b** or 1 h in the case of **16a+16b, 18a and 21a+21b**. The resulting precipitate was filtered, washed with petroleum ether and dissolved in MeOH. Azaperoxides **16a+16b, 18a-21a + 18b-21b** and **23a+23b** were isolated by chromatography on SiO_2 using MeOH as the eluent.

Compounds: **16a+16b:** 0.151 g, 0.34 mmol, yield 39% (**16a** : **16b** = 77 : 23); **18a:** 0.197 g, 0.32 mmol, yield 64%; **19a+19b:** 0.220 g, 0.42mmol, yield 65% (**19a** : **19b** = 78 : 22); **20a+20b:** 0.212 g, 0.42 mmol, yield 61% (**20a** : **20b** = 78 : 22); **21a+21b:** 0.222 g, 0.43 mmol, yield 65% (**21a** : **21b** = 80 : 20); **23a+23b:** 0.234 g, 0.52 mmol, yield 62% (**23a** : **23b** = 78 : 22).

Ethyl 8-(2-bromobenzamido)-2-(4-chlorobenzyl)-1,5-dimethyl-6,7-dioxa-8-azabicyclo[3.2.1]octane-2-carboxylate, 15a+15b[10]

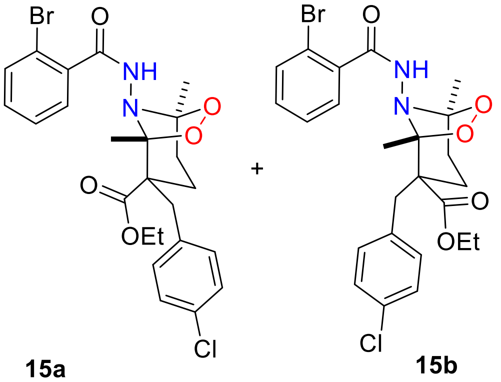

15a **15b**

White crystals. Mp = 127 – 129 °C. (Lit.[10] Mp = 126 – 128 °C). **15a : 15b** = 75 : 25.

^{1}H NMR (300.13 MHz, $CDCl_3$), δ: 0.97 (t, *J* = 7.1 Hz, 0.75H), 1.40 – 1.50 (m, 0.25H), 1.41 (t, *J* = 7.1 Hz, 2.25H), 1.57 (s, 2.25H), 1.60 – 1.69 (m, 0.75H), 1.62 (s, 0.75H), 1.78 – 2.04 (m, 2H), 1.91 (s, 2.25H), 1.92 (s, 0.75H), 2.32 – 2.49 (m, 1H), 2.58 (d, *J* = 12.8 Hz, 0.25H), 2.62 (d, *J* = 12.8 Hz, 0.75H), 3.26 (d, *J* = 12.8 Hz, 0.25H), 3.34 – 3.29 (m, 0.5H), 3.38 (d, *J* = 12.8 Hz, 0.75H), 4.31 – 4.45 (m, 1.5H), 6.93 (d, *J* = 8.5 Hz, 1.5H), 7.01 (d, *J* = 8.5 Hz, 0.5H), 7.15 – 7.50 (m, 5H), 7.28 – 7.30 (m, 1H), 7.61 – 7.67 (m, 1H). ^{13}C NMR (75.48 MHz, $CDCl_3$), δ: 13.9, 14.3, 15.9, 18.8, 19.6, 25.5, 26.2, 35.1, 35.3, 41.0, 41.6, 56.6, 60.7, 61.8, 102.0, 102.1, 105.3, 105.7, 119.6, 120.0, 126.7, 127.7, 128.5, 128.6, 129.1, 129.3, 130.6, 131.2, 131.8, 133.4, 133.7, 134.7, 136.8, 165.4, 170.9, 171.7, 171.9. Anal. Calcd. for $C_{24}H_{26}BrClN_2O_5$: C, 53.60; H, 4.87; Br, 14.86; Cl, 6.59; N, 5.21.Found: C, 53.78; H, 4.98; Br, 14.97; Cl, 6.64; N, 5.28. HRMS (ESI-TOF): m/z $[M+H]^+$: calculated for $[C_{24}H_{27}BrClN_2O_5]^+$: 537.0786; found: 537.0778.

Ethyl 8-(2-bromobenzamido)-2-ethyl-1,5-dimethyl-6,7-dioxa-8-azabicyclo[3.2.1]octane-2-carboxylate, 16a + 16b

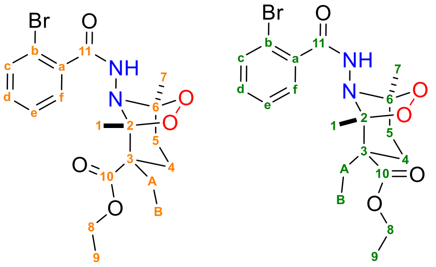

major **16a** minor **16b**

White crystals. Mp = 133-135 °C. **16a : 16b** = 77 : 23. R_f= 0.53 (TLC, PE : EA, 2 : 1).

16a: ^{1}H NMR (300.13 MHz, $CDCl_3$), δ: 0.82 (t, *J* = 7.1 Hz, 3H, (**H^B**)), 1.04 (t, *J* = 7.1 Hz, 3H, (**H^9**)), 1.53 (s, 3H, (**H^7**)), 1.42 – 1.56 (m, 1H, (**H^A**)), 170 – 1.86 (m, 1H, (**H^4**)), 1.76 (s, 3H, (**H^1**)), 1.86 – 1.97 (m, 2H, (**H^A, H^5**)), 2.17 (dd, *J* = 13.3, 5.9 Hz, 1H, (**H^4**)), 2.45 (td, *J* = 13.0, 6.2 Hz, 1H, (**H^5**)), 4.13 – 4.42 (m, 2H, (**H^8**)), 7.26 – 7.46 (m, 3H, (**H^d, H^e, H^f**)), 7.27 (br.s., 1H, (N**H**), 7.58 – 7.66 (m, 1H, (**H^c**)). ^{13}C NMR (75.48 MHz, $CDCl_3$), δ: 8.53 (**C^B**), 14.25 (**C^9**), 15.8 (**C^1**), 18.8 (**C^7**), 25.3 (**C^4**), 28.5 (**C^A**), 35.2 (**C^5**), 56.5 (**C^3**), 61.4 (**C^8**), 101.9 (**C^6**), 105.6 (**C^2**), 119.6 (**C^b**), 127.6 (**C^e**), 129.2 (**C^f**), 131.6 (**C^d**), 133.6 (**C^c**), 137.0 (**C^a**), 165.4 (**C^{11}**), 172.5 (**C^{12}**). ^{15}N (40.56 MHz, $CDCl_3$), δ: 136.5 (**N**), 140.5 (N**H**).

16b: ^{1}H NMR (300.13 MHz, $CDCl_3$), δ: 0.70 (t, *J* = 7.1 Hz, 3H, (**H^B**)), 1.34 (t, *J* = 7.1 Hz, 3H, (**H^9**)), 1.61 (s, 3H, (**H^7**)), 1.42 – 1.56 (m, 1H, (**H^A**)), 1.69 – 2.14 (m, 4H, (**H^4, H^A, H^5**)), 1.78 (s, 3H, (**H^1**)), 2.40 – 2.54 (m, 1H, (**H^5**)), 3.52 (q, *J* = 7.2 Hz, 2H, (**H^8**)), 7.12 – 7.22 (m, 2H, (**H^f**, N**H**)), 7.22 –7.39 (m, 2H, (**H^d, H^e**)), 7.58 – 7.66 (m, 1H, (**H^c**)). ^{13}C NMR (75.48 MHz, $CDCl_3$), δ: 8.3 (**C^B**), 14.0 (**C^9**), 15.8 (**C^1**), 19.5 (**C^7**), 24.8 (**C^4**), 29.3 (**C^A**), 35.3 (**C^5**), 56.0 (**C^3**), 60.5 (**C^8**), 101.9 (**C^6**), 105.8 (**C^2**), 120.1 (**C^b**), 126.6 (**C^e**), 129.2 (**C^f**), 130.6 (**C^d**), 133.4 (**C^c**), 135.9 (**C^a**), 170.8 (**C^{11}**), 172.1 (**C^{10}**). ^{15}N (40.56 MHz, $CDCl_3$), δ: 142.2 (**N**), 143.0 (N**H**). Anal. Calcd.for$C_{19}H_{25}BrN_2O_5$: C, 51.71; H, 5.71; Br, 18.11; N, 6.35. Found: C, 51.89; H, 5.88; Br, 18.30; N, 6.44. HRMS (ESI-TOF): m/z $[M+Na]^+$: calculated for$[C_{19}H_{25}BrN_2NaO_5]^+$: 463.0839, 465.0820; found: 463.0833, 465.0821.

Allyl 2-allyl-8-(2-bromobenzamido)-1,5-dimethyl-6,7-dioxa-8-azabicyclo[3.2.1]octane-2-carboxylate, 17a + 17b[10]

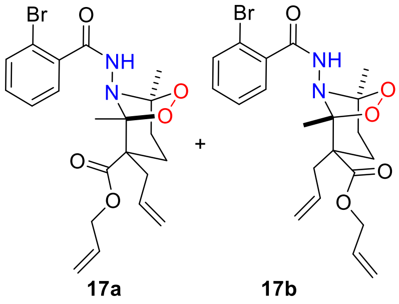

White crystals. Mp = 140 – 142 °C (Lit.[10] Mp = 139 – 141 °C). **17a : 17b** = 80 : 20.

^{1}H NMR (300.13 MHz, $CDCl_3$), δ: 1.56 (s, 2.4H), 1.61 (s, 0.6H), 1.77 – 1.98 (m, 1.8H), 1.80 (s, 3H), 2.03 – 2.75 (m, 4.2H), 3.90 – 4.05 (m, 0.4H), 4.77 (d, *J* = 5.9 Hz, 1.6H), 4.96 – 5.74 (m, 5H), 5.96 – 6.11 (m, 1H), 7.13 – 7.46 (m, 4H), 7.58 – 7.65 (m, 1H). ^{13}C NMR (75.48 MHz, $CDCl_3$), δ: 15.9, 18.8, 19.5, 25.6, 26.2, 35.2, 35.3, 40.3, 41.0, 55.6, 55.9, 65.4, 66.3, 102.0, 105.1, 105.4, 118.6, 119.0, 119.2, 119.6, 126.7, 127.6, 129.1, 129.3, 130.6, 131.6, 132.0, 132.4, 132.7, 133.4, 133.6, 136.9, 165.4, 171.3, 171.8. Anal. Calcd. for $C_{21}H_{25}BrN_2O_5$: C, 54.20; H, 5.42; Br, 17.17; N, 6.02. Found: C, 54.32; H, 5.59; Br, 17.29; N, 6.15. HRMS (ESI-TOF): m/z $[M+Na]^+$: calculated for $[C_{21}H_{25}BrN_2NaO_5]^+$: 487.0839, 489.0820; found: 487.0829, 489.0810.

***tert*-Butyl 8-(2-bromobenzamido)-2-(4-bromobenzyl)-1,5-dimethyl-6,7-dioxa-8-azabicyclo[3.2.1]octane-2-carboxylate, 18a**

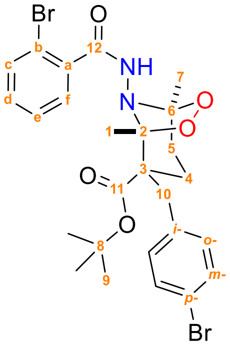

White crystals. Mp = 159 – 161 °C. R_f = 0.54 (TLC, PE : EA, 2 : 1).

^{1}H NMR (300.13 MHz, $CDCl_3$), δ: 1.48 – 1.60 (m, 1H, **H^{4}**), 1.56 (s, 3H, **H^{7}**), 1.59 (s, 9H, **H^{9}**), 1.76 – 1.90 (m, 2H, **H^{4}, H^{5}**), 1.87 (s, 3H, **H^{1}**), 2.27 (m, 1H, **H^{5}**), 2.57 (d, *J* = 12.7 Hz, 1H, **H^{10}**), 3.33 (d, *J* = 12.7 Hz, 1H, **H^{10}**), 7.00 (d, *J* = 8.2 Hz, 2H, **H^{o-}**), 7.17 (br.s, 1H, N**H**), 7.29 – 7.46 (m, 5H, (**H^{d}, H^{e}**, **H^{m-}, H^{f}**), 7.62 (d, *J* = 7.7 Hz, 1H, **H^{c}**). ^{13}C NMR (75.48 MHz, $CDCl_3$), δ: 15.9 (**C^{1}**), 19.0 (**C^{7}**), 26.2 (**C^{4}**), 28.2 (**C^{9}**), 35.1 (**C^{5}**), 41.4 (**C^{10}**), 56.6 (**C^{3}**), 82.8 (**C^{8}**), 102.3 (**C^{6}**), 105.5 (**C^{2}**), 119.6 (**C^{b}**), 120.9 (**C^{p-}**), 127.6 (**C^{e}**), 129.2 (**C^{f}**), 131.4 (**C^{m-}**), 131.7 (**C^{d}**), 131.9 (**C^{o-}**), 133.7 (**C^{c}**), 135.4 (**C^{i-}**), 136.9 (**C^{a}**), 165.4 (**C^{12}**), 170.9 (**C^{11}**). ^{15}N (40.56 MHz, $CDCl_3$), δ: 137.1 (**N**), 140.7 (N**H**). Anal. Calcd. for $C_{26}H_{30}Br_2N_2O_5$: C, 51.17; H, 4.95; Br, 26.18; N, 4.59. Found: C, 51.31; H, 5.07; Br, 26.29; N, 4.72. HRMS (ESI-TOF): m/z $[M+H]^+$: calculated for$[C_{26}H_{31}Br_2N_2O_5]^+$:609,0594; found: 609,0587.

Ethyl 8-(2-bromobenzamido)-2-(4-fluorobenzyl)-1,5-dimethyl-6,7-dioxa-8-azabicyclo[3.2.1]octane-2-carboxylate, 19a + 19b

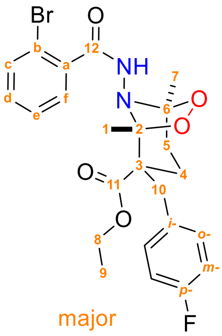

major
19a

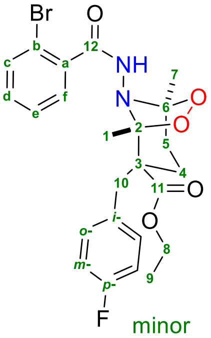

minor
19b

White crystals. Mp = 134 – 136 °C. **19a : 19b** = 78 : 22. R_f= 0.43 (TLC, PE : EA, 2 : 1).

19a: ^{1}H NMR (300.13 MHz, $CDCl_3$), δ: 1.40 (t, *J* = 7.1 Hz, 3H, (**H^{9}**)), 1.56 (s, 3H. (**H^{7}**)), 1.60 – 1.69 (m, 1H, (**H^{4}**), 1.76 – 1.90 (m, 2H, (**H^{4}**, **H^{5}**)) 1.91 (s, 3H, (**H^{1}**)), 2.31 – 2.49 (m, 1H, (**H^{5}**)), 2.62 (d, *J* = 12.8 Hz, 1H, (**H^{10}**)), 3.39 (d, *J* = 12.8 Hz, 1H, (**H^{10}**)), 4.27 – 4.47 (m, 2H, (**H^{8}**)), 6.84 – 7.08 (m, 4H, (**H^{m-}**,**H^{o-}**)), 7.25 – 7.47 (m, 3H, (**H^{f}** ,**H^{e}**,**H^{d}**)), 7.30 – 7.38 (m, 1H, (N**H**), 7.60 – 7.67 (m, 1H, (**H^{c}**)). ^{13}C NMR (75.48 MHz, $CDCl_3$), δ: 14.3 (**C^{9}**), 15.8 (**C^{1}**), 18.8 (**C^{7}**), 26.1 (**C^{4}**), 35.1 (**C^{5}**), 40.7 (**C^{10}**), 56.7 (**C^{3}**), 61.7 (**C^{8}**), 102.0 (**C^{6}**), 105.3 (**C^{2}**),

115.1 (**C**$^{m-}$), 115.0 (d, $^{2}J_{CF}$ = 21.0 Hz, (**C**$^{m-}$)),119.6 (**C**^{b}), 127.6 (**C**^{e}), 129.3 (**C**^{f}), 131.3 (d, $^{3}J_{CF}$ = 7.9 Hz, (**C**$^{o-}$)), 131.7 (**C**^{d}), 131.8 (d, $^{4}J_{CF}$ = 3.3 Hz, (C^{i-})), 133.6 (**C**^{c}), 136.9 (**C**^{a}), 162.0 (d, $^{1}J_{CF}$ = 244.8 Hz, (C^{p-})), 165.4 (**C**^{12}), 171.9 (**C**^{11}). ^{15}N (40.56 MHz, $CDCl_3$), δ: 136.0 (**N**), 140.1 (**NH**).

19b: ^{1}H NMR (300.13 MHz, $CDCl_3$), δ: 0.96 (t, *J* = 7.2 Hz, 3H, (**H**^{9})), 1.43 – 1.49 (m, 1H, (**H**^{4})), 1.61 (s, 3H, (**H**^{7})), 1.76 – 2.00 (m, 2H, , (**H**^{4}, **H**^{5})), 1.91 (s, 3H, (**H**^{1})), 2.33 – 2.49 (m, 1H, (**H**^{5})), 2.59 (d, *J* = 12.8 Hz, 1H, (**H**^{10})), 3.25 (d, *J* = 12.8 Hz, 1H, (**H**^{10})), 3.35 – 3.60 (m, 2H, (**H**^{8})), 6.84 – 7.08 (m, 4H, (**H**$^{m-}$,**H**$^{o-}$)), 7.16 – 7.49 (m, 3H, (**H**^{f} ,**H**^{e},**H**^{d})), 7.19 – 7.23 (m, 1H, (N**H**), 7.60 – 7.67 (m, 1H, (**H**^{c})). ^{13}C NMR (75.48 MHz, $CDCl_3$), δ: 13.8 (**C**^{9}), 15.8 (**C**^{1}), 19.5 (**C**^{7}), 25.5 (**C**^{4}), 35.2 (**C**^{5}), 41.4 (**C**^{10}), 56.3 (**C**^{3}), 60.6 (**C**^{8}), 102.0 (**C**^{6}), 105.6 (**C**^{2}), 115.1 (**C**$^{m-}$), 115.2 (d, $^{2}J_{CF}$ = 21.0 Hz, (**C**$^{m-}$)), 120.0 (**C**^{b}), 126.7 (**C**^{e}), 129.1 (**C**^{f}), 130.6 (**C**^{d}), (d, $^{3}J_{CF}$ = 7.9 Hz, (**C**$^{o-}$)), 131.6 (**C**$^{o-}$), 131.8 (d, $^{4}J_{CF}$ = 3.3 Hz, (C^{i-})), 133.4 (**C**^{c}), 136.0 (**C**^{a}), 162.0 (d, $^{1}J_{CF}$ = 244.8 Hz, (C^{p-})), 170.9 (**C**^{12}), 171.7 (**C**^{11}). ^{15}N (40.56 MHz, $CDCl_3$), δ: 141.9 (**N**), 142.8 (**NH**). Anal. Calcd. For $C_{24}H_{26}BrFN_2O_5$: C, 55.29; H, 5.03; Br, 15.33; F, 3.64; N, 5.37. Found: C, 55.40; H, 5.12; Br, 15.39; F, 3.78; N, 5.49. HRMS (ESI-TOF): m/z [M+Na]$^{+}$: calculated for[$C_{24}H_{26}BrFN_2NaO_5$]$^{+}$: 543.0901, 545.0882; found: 543.0897, 545.0895.

Ethyl 2-benzyl-8-(2-bromobenzamido)-1,5-dimethyl-6,7-dioxa-8-azabicyclo[3.2.1]octane-2-carboxylate, 20a + 20b

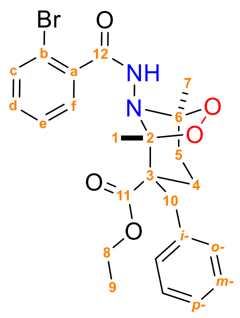

major
20a

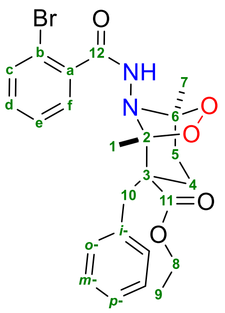

minor
20b

White crystals. Mp = 136 – 138 °C. **20a : 20b** = 78 : 22. R_f= 0.55 (TLC, PE : EA, 2 : 1).

20a: ^{1}H NMR (300.13 MHz, $CDCl_3$), δ: 1.38 (t, *J* = 7.1 Hz, 3H, (**H**^{9})), 1.54 (s, 3H. (**H**^{7})), 1.56 – 1.86 (m, 3H, (**H**^{4}, **H**^{5})) 1.90 (s, 3H, (**H**^{1})), 2.31 – 2.46 (m, 1H, (**H**^{5})), 2.59 (d, *J* = 12.8 Hz, 1H, (**H**^{10})), 3.39 (d, *J* = 12.8 Hz, 1H, (**H**^{10})), 4.27 – 4.47 (m, 2H, (**H**^{8})), 6.04 – 7.11 (m, 2H, (**H**$^{o-}$)), 7.18 – 7.49 (m, 6H, (**H**^{f} ,**H**^{e},**H**^{d} , **H**$^{p-}$, **H**$^{m-}$)), 7.30 – 7.38 (m, 1H, (N**H**), 7.60 – 7.67 (m, 1H, (**H**^{c})). ^{13}C NMR (75.48 MHz, $CDCl_3$), δ: 14.3 (**C**^{9}), 15.9 (**C**^{1}), 18.8 (**C**^{7}), 26.1 (**C**^{4}), 35.1 (**C**^{5}), 41.6 (**C**^{10}), 56.7 (**C**^{3}), 60.6 (**C**^{8}), 102.1 (**C**^{6}), 105.4 (**C**^{2}), 119.6 (**C**^{b}), 126.9 (**C**^{p}), 127.6 (**C**^{e}), 128.4 (**C**$^{m-}$), 129.3 (**C**^{f}), 129.9 (**C**$^{o-}$), 131.6 (**C**^{d}), 133.6 (**C**^{c}), 136.2 (**C**$^{i-}$), 136.9 (**C**^{a}), 165.4 (**C**^{12}), 172.0 (**C**^{11}). ^{15}N (40.56 MHz, $CDCl_3$), δ: 136.0 (**N**), 140.3 (**NH**).

20b: ^{1}H NMR (300.13 MHz, $CDCl_3$), δ: 0.94 (t, *J* = 7.2 Hz, 3H, (**H**^{9})), 1.40 – 1.49 (m, 1H, (**H**^{4})), 1.59 (s, 3H, (**H**^{7})), 1.81 – 1.95 (m, 1H, (**H**^{4}), 1.92 (s, 3H, (**H**^{1})), 1.85 – 2.01 (m, 1H, (**H**^{5})), 2.32 – 2.47 (m, 1H, (**H**^{5})), 2.61 (d, *J* = 12.8 Hz, 1H, (**H**^{10})), 3.31 (d, *J* = 12.8 Hz, 1H, (**H**^{10})), 3.36 – 3.60 (m, 2H, (**H**^{8})), 6.96 – 7.02 (m, 2H,

(**H**$^{o-}$)), 7.18 – 7.41 (m, 6H, (**H^{f},H^{e},H^{d} , H^{p-}, H^{m-}**)), 7.19 – 7.23 (m, 1H, (**NH**), 7.60 – 7.67 (m, 1H, (**H**^{c})). ^{13}C NMR (75.48 MHz, $CDCl_3$), δ: 13.8 (**C**^{9}), 15.9 (**C**^{1}), 19.5 (**C**^{7}), 25.5 (**C**^{4}), 35.3 (**C**^{5}), 42.3 (**C**^{10}), 56.3 (**C**^{3}), 60.6 (**C**^{8}), 102.1 (**C**^{6}), 105.7 (**C**^{2}), 120.0 (**C**^{b}), 126.7 (**C**^{e}), 126.9 (**C**$^{p-}$), 128.3 (**C**$^{m-}$), 129.2 (**C**^{f}), 129.9 (**C**$^{o-}$), 130.6 (**C**^{d}), 133.4 (**C**^{c}), 131.4 (**C**$^{o-}$), 135.9 (**C**$^{i-}$),136.9 (**C**^{a}), 171.0 (**C**^{12}), 171.9 (**C**^{11}). ^{15}N (40.56 MHz, $CDCl_3$), δ: 142.1 (**N**), 143.2 (**NH**). Anal. Calcd.for $C_{24}H_{27}BrN_2O_5$: C, 57.26; H, 5.41; Br, 15.87; N, 5.57. Found: C, 57.35; H, 5.55; Br, 15.99; N, 5.98. HRMS (ESI-TOF): m/z $[M+Na]^+$: calculated for$[C_{24}H_{27}BrN_2NaO_5]^+$: 525.0996, 527.0976; found: 525.0988, 527.0970

Benzyl 2-allyl-8-(2-bromobenzamido)-1,5-dimethyl-6,7-dioxa-8-azabicyclo[3.2.1]octane-2-carboxylate, 21a+21b

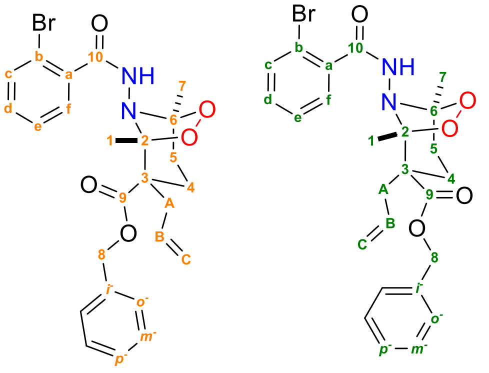

major minor

White crystals. Mp = 121 – 123 °C. **21a : 21b** = 80 : 20. R_f= 0.43 (TLC, PE : EA, 2 : 1).

21a: ^{1}H NMR (300.13 MHz, $CDCl_3$), δ: 1.58 (s, 3H. (**H**^{7})), 1.77 (s, 3H. (**H**^{1})), 1.83 – 1.96 (m, 2H, (**H**^{4}, **H**^{5})), 2.10 – 2.34 (m, 2H, (**H**^{4}, **H**^{A})), 2.42 – 2.73 (2H, (**H**^{5}, **H**^{A})), 5.01 – 5.21 (m, 2H, (**H**^{C})), 5.26 (d, *J* = 12.4 Hz, 1H, (**H**^{8})), 5.35 (d, *J* = 12.4 Hz, 1H, (**H**^{8})), 5.49 – 5.71 (m, 1H, (**H**^{B})), 7.24 – 7.49 (m, 9H, (**H^{d}, H^{e}, H^{f} , H^{o-}, H^{m-}, H^{p-},** NH)), 7.58 – 7.63 (m, 1H, (**H**^{c})). ^{13}C NMR (75.48 MHz, $CDCl_3$), δ: 15.9 (**C**^{1}), 18.8 (**C**^{7}), 26.2 (**C**^{4}), 35.2 (**C**^{5}), 40.3 (**C**^{A}), 56.0 (**C**^{3}), 67.6 (**C**^{8}), 102.1 (**C**^{6}), 105.1 (**C**^{2}), 119.0 (**C**^{C}), 119.6 (**C**^{b}), 127.6 (**C**^{e}), 128.3 (**C**$^{p-}$), 128.6 (**C**$^{m-}$), 129.0 (**C**$^{o-}$), 129.2 (**C**^{f}), 131.7 (**C**^{d}), 132.4 (**C**^{B}), 133.6 (**C**^{c}), 136.1 (**C**$^{i-}$), 136.8 (**C**^{a}), 165.6 (**C**^{10}), 172.0 (**C**^{9}). ^{15}N (40.56 MHz, $CDCl_3$), δ: 136.3 (**N**), 140.5 (**NH**).

21a: ^{1}H NMR (300.13 MHz, $CDCl_3$), δ: 1.63 (s, 3H. (**H**^{7})), 1.85 (s, 3H. (**H**^{1})), 1.83 – 1.96 (m, 2H, (**H**^{4}, **H**^{5})), 2.10 – 2.34 (m, 2H, (**H**^{4}, **H**^{A})), 2.42 – 2.73 (2H, (**H**^{5}, **H**^{A})), 4.45 (d, *J* = 12.4 Hz, 1H, (**H**^{8})), 4.56 (d, *J* = 12.4 Hz, 1H, (**H**^{8})), 4.89 – 4.98 (m, 2H, (**H**^{C})), 5.49 – 5.71 (m, 1H, (**H**^{B})), 7.24 – 7.49 (m, 9H, (**H^{d}, H^{e}, H^{f} , H^{o-}, H^{m-}, H^{p-},** NH)), 7.58 – 7.63 (m, 1H, (**H**^{c})). ^{13}C NMR (75.48 MHz, $CDCl_3$), δ: 16.2 (**C**^{1}), 19.6 (**C**^{7}), 25.5 (**C**^{4}), 35.3 (**C**^{5}), 41.0 (**C**^{A}), 55.6 (**C**^{3}), 66.6 (**C**^{8}), 102.1 (**C**^{6}), 105.4 (**C**^{2}), 119.2 (**C**^{C}), 119.6 (**C**^{b}), 127.6 (**C**^{e}), 128.3 (**C**$^{p-}$), 128.6 (**C**$^{m-}$), 129.0 (**C**$^{o-}$), 129.2 (**C**^{f}), 131.9 (**C**^{d}), 132.0 (**C**^{B}), 133.6 (**C**^{c}), 170.9 (**C**^{10}), 171.6 (**C**^{9}). ^{15}N (40.56 MHz, $CDCl_3$), δ: 142.2 (**N**), 143.0 (**NH**). Anal. Calcd. for $C_{25}H_{27}BrN_2O_5$: C, 58.26; H, 5.28; Br, 15.50; N, 5.44. Found: C, 58.34; H, 5.40; Br, 15.66; N, 5.58.

HRMS (ESI-TOF): m/z [M+Na]$^+$: calculated for[$C_{25}H_{27}BrN_2NaO_5$]$^+$: 537.0996, 539.0976; found: 537.0989, 539.0972.

Ethyl 8-(2-bromobenzamido)-2-butyl-1,5-dimethyl-6,7-dioxa-8-azabicyclo[3.2.1]octane-2-carboxylate, 22a+22b[10]

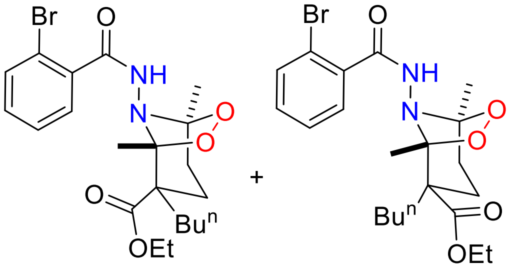

22a **22b**

White crystals. Mp = 128 – 130 °C (Lit.[10] Mp = 128 – 130 °C). **22a : 22b** = 78 : 22.

^{1}H NMR (300.13 MHz, $CDCl_3$), δ: 0.80 – 0.95 (m, 3H), 1.00 – 1.10 (m, 1.66H), 1.20 – 1.51 (m, 6.34H), 1.55 (s, 2.34H), 1.61 (s, 0.66H), 1.71 – 2.56 (m, 5H), 1.78 (s, 0.66H), 1.79 (s, 2.34H), 3.53 (q, *J* = 7.2 Hz, 0.44H), 4.26 – 4.37 (m, 1.56H), 7.14 – 7.45 (m, 3H), 7.31 – 7.37 (m, 1H), 7.58 – 7.65 (m, 1H). ^{13}C NMR (75.48 MHz, $CDCl_3$), δ: 14.0, 14.2, 15.8, 18.8, 19.5, 23.0, 23.2, 25.4, 25.8, 25.9, 26.2, 35.2, 35.5, 35.5, 36.1, 55.6, 56.0, 60.5, 61.4, 101.9, 101.9, 105.6, 105.8, 119.6, 120.0, 126.6, 127.6, 129.2, 129.2, 130.6, 131.6, 133.4, 133.6, 135.9, 136.8, 165.5, 170.9, 172.2, 172.7. Anal. Calcd. for $C_{21}H_{29}BrN_2O_5$: C, 53.74; H, 6.23; Br, 17.02; N, 5.97. Found: C, 53.82; H, 6.33; Br, 17.12; N, 6.10. HRMS (ESI-TOF): m/z [M+Na]$^+$: calculated for [$C_{21}H_{29}BrN_2NaO_5$]$^+$: 491.1152, 493.1133; found: 491.1144, 493.1130.

Ethyl 2-allyl-8-(2-bromobenzamido)-1,5-dimethyl-6,7-dioxa-8-azabicyclo[3.2.1]octane-2-carboxylate, 23a + 23b

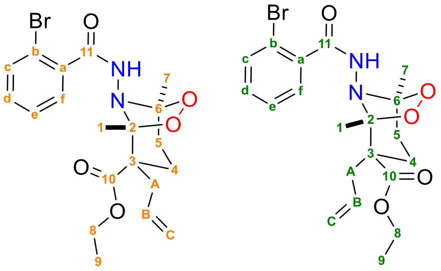

major **23a**

minor **23b**

White crystals. Mp = 135-137 °C. **23a : 23b** = 78 : 22. R_f= 0.50 (TLC, PE : EA, 2 : 1).

23a: ^{1}H NMR (300.13 MHz, $CDCl_3$), δ: 1.36 (t, *J* = 7.2 Hz, 3H, (**H^9**)), 1.55 (s, 3H, (**H^7**)), 1.80 (s, 3H, (**H^1**)), 1.74 – 1.95 (m, 2H, (**H^4**, **H^5**)), 2.00 – 2.26 (m, 2H, (**H^4**, **H^A**)), 2.29 – 2.74 (m, 2H, (**H^A**, **H^5**)), 4.23 – 4.42 (m, 2H, (**H^8**)), 4.96 – 5.16 (m, 2H, (**H^C**)), 5.54 – 5.72 (m, 1H, (**H^B**)), 7.25 – 7.47 (m, 3H, (**H^d**, **H^e**, **H^f**)), 7.31 – 7.40 (m, 1H, (N**H**), 7.56 – 7.66 (m, 1H, (**H^c**)). ^{13}C NMR (75.48 MHz, $CDCl_3$), δ: 14.3 (**C^9**), 15.9 (**C^1**), 18.8 (**C^7**),

26.1 (**C⁴**), 35.2 (**C⁵**), 40.3 (**C^A^**), 55.8 (**C³**), 61.5 (**C⁸**), 102.1 (**C⁶**), 105.2 (**C²**), 118.8 (**C^C^**), 119.6 (**C^b^**), 127.6 (**C^e^**), 129.2 (**C^f^**), 131.6 (**C^d^**), 132.5 (**C^B^**), 133.6 (**C^c^**), 136.9 (**C^a^**), 136.4 (**C^11^**), 172.1 (**C^10^**). ^{15}N (40.56 MHz, $CDCl_3$), δ: 136.3 (**N**), 140.4 (**N**H).

23b: ^{1}H NMR (300.13 MHz, $CDCl_3$), δ: 1.05 (t, *J* = 7.2 Hz, 3H, (**H**^{9})), 1.61 (s, 3H, (**H**^{7})), 1.80 (s, 3H, (**H**^{1})), 1.74 – 1.95 (m, 1H, (**H**^{4})), 2.00 – 2.26 (m, 3H, (**H**^{4}, **H**^{5}, **H**^{A})), 2.29 – 2.74 (m, 2H, (**H**^{A}, **H**^{5})), 3.53 (q, *J* = 7.2 Hz, 2H, (**H**^{8})), 4.96 – 5.16 (m, 2H, (**H**^{C})), 5.40 – 5.59 (m, 1H, (**H**^{B})), 7.13 – 7.23 (m, 1H, (**H**^{f})), 7.19 – 7.23 (m, 1H, (N**H**), 7.24 – 7.40 (m, 2H, (**H**^{d}, **H**^{e})), 7.56 – 7.66 (m, 1H, (**H**^{c})). ^{13}C NMR (75.48 MHz, $CDCl_3$), δ: 14.0 (**C**^{9}), 16.2 (**C**^{1}), 19.5 (**C**^{7}), 25.6 (**C**^{4}), 35.3 (**C**^{5}), 40.9 (**C**^{A}), 55.4 (**C**^{3}), 60.6 (**C**^{8}), 102.1 (**C**^{6}), 105.4 (**C**^{2}), 117.1 (**C**^{C}), 120.0 (**C**^{b}), 126.7 (**C**^{e}), 129.1 (**C**^{f}), 130.6 (**C**^{d}), 132.1 (**C**^{B}), 133.4 (**C**^{c}), 135.9 (**C**^{a}), 170.8 (**C**^{11}), 171.6 (**C**^{10}). ^{15}N (40.56 MHz, $CDCl_3$), δ: 142.0 (**N**), 142.9 (**N**H). Anal. Calcd. for $C_{20}H_{25}BrN_2O_5$: C, 52.99; H, 5.56; Br, 17.63; N, 6.18. Found: C, 53.12; H, 5.71; Br, 17.72; N, 6.27. HRMS (ESI-TOF): m/z $[M+Na]^+$: calculated.for$[C_{20}H_{25}BrN_2NaO_5]^+$:475.0839, 477.0820; found: 475.0834, 477.0815.

2-Bromo-N-(1,5-dimethyl-3-(4-nitrophenyl)-6,7-dioxa-8-azabicyclo[3.2.1]octan-8-yl)benzamide, 24[10]

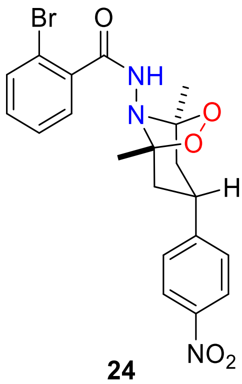

24

Yellow crystals. Mp = 136-138 °C (Lit.[10] Mp = 143 – 145 °C).

^{1}H NMR (300.13 MHz, $CDCl_3$), δ: 1.67 (s, 6H), 2.25 (dd, *J* = 14.1, 5.7 Hz, 2H), 2.36 – 2.48 (m, 2H), 3.64 – 3.80 (m, 1H), 7.30 – 7.52 (m, 6H), 7.64 (dd, *J* = 7.8, 1.3 Hz, 1H), 8.18 (d, *J* = 8.8 Hz, 2H). ^{13}C NMR (75.48 MHz, $CDCl_3$), δ: 19.2, 35.9, 44.0, 101.8, 119.5, 124.1, 127.8, 128.2, 129.4, 132.0, 133.8, 136.5, 147.1, 150.3, 166.1. Anal. Calcd. for $C_{20}H_{20}BrN_3O_5$: C, 51.96; H, 4.36; Br, 17.28; N, 9.09. Found: C, 52.08; H, 4.49; Br, 17.38; N, 9.21. HRMS (ESI-TOF): m/z $[M+H]^+$: calculated for $[C_{20}H_{21}BrN_3O_5]^+$:462.0659, 464.0640; found: 462.0655, 464.0638.

Synthesis of aminoperoxides 25a-28a + 25b-28b

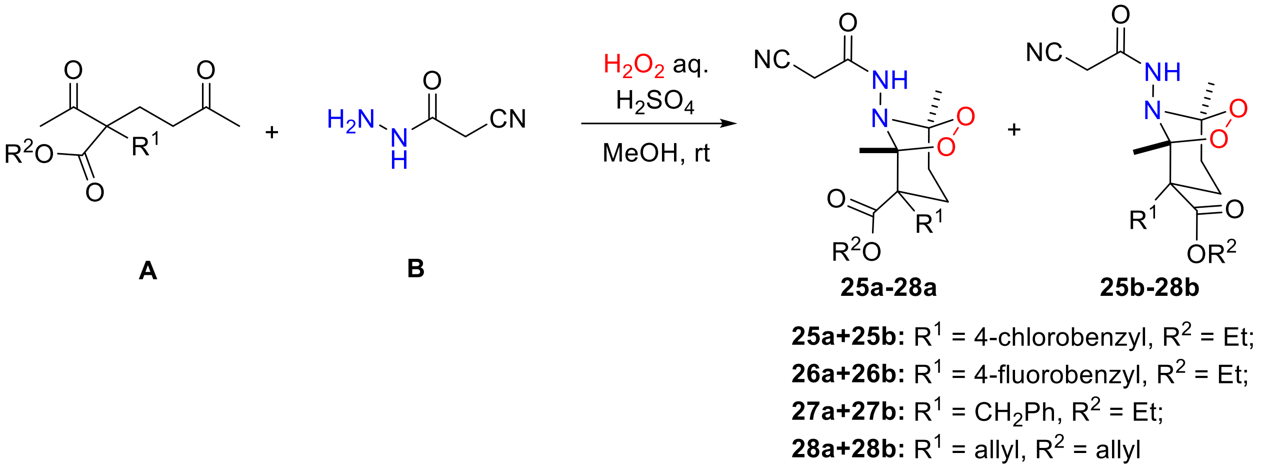

A **B** **25a-28a** **25b-28b**

25a+25b: R^1 = 4-chlorobenzyl, R^2 = Et;
26a+26b: R^1 = 4-fluorobenzyl, R^2 = Et;
27a+27b: R^1 = CH_2Ph, R^2 = Et;
28a+28b: R^1 = allyl, R^2 = allyl

Aminoperoxides **25a-28a + 25b-28b** were synthesized according to a known procedure.[10] Aminoperoxides **25a + 25b** are known compounds.[10] Aminoperoxides **26a-28a** + **26b-28b** are new compounds.

Procedure for the synthesis of aminoperoxides 26a-28a + 26b-28b

A 98% H_2SO_4 (67 µL – 85 µL, 1.24 – 1.58 mmol, 2.0 mol of H_2SO_4 / 1.0 mol of **A**), 2-cyanoacetohydrazide **B** (62 – 79 mg, 0.62 – 0.79 mmol, 1.0 mol of **B**/1.0 mol of **A**) and 35 % aq. solution of H_2O_2 (81 – 102 µL, 0.93 – 1.18 mmol, 1.5 mol of H_2O_2 / 1.0 mol of **A**) were successively added with stirring to a solution of 1,5-diketone **A** (0.200 g, 0.62 – 0.79 mmol) in MeOH (1-2 mL). The reaction mixture was stirred at 20 - 25 ° C for 0.5 h in the case of **26a + 26b** and **27a + 27b** or 1 h in the case of **28a + 28b**. The resulting precipitate was filtered, washed with petroleum ether and dissolved in MeOH. Aminoperoxides **26a + 26b – 28a + 28b** were isolated by chromatography on SiO_2 using MeOH as the eluent.

Mixtures: **26a + 26b:** 0.126 g, 0.31 mmol, yield 48% (**26a : 26b** = 92:8); **27a + 27b :** 0.144 g, 0.37 mmol, yield 54% (**27a : 27b** = 92:8); **28a + 28b :** 0.111 g, 0.32 mmol, yield 40% (**28a : 28b** = 92:8).

Ethyl 2-(4-chlorobenzyl)-8-(2-cyanoacetamido)-1,5-dimethyl-6,7-dioxa-8-azabicyclo[3.2.1]octane-2-carboxylate, 25a + 25b[10]

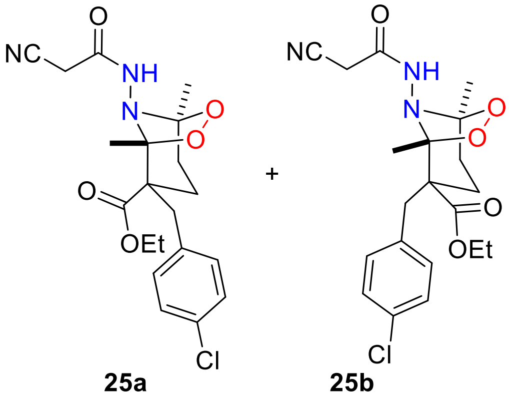

25a **25b**

White crystals. Mp = 143 – 145 °C (Lit.[10] Mp = 143 – 145 °C). **25a : 25b** = 93 : 7.

^{1}H NMR (300.13 MHz, $CDCl_3$), δ: 1.20 (t, *J* = 7.1 Hz, 2.79H), 1.34 (t, *J* = 7.1 Hz, 0.21H), 1.44 (s, 0.21H), 1.53 (s, 2.73H), 1.66 – 2.29 (m, 4H), 1.81 (s, 3H), 2.60 (d, *J* = 12.7 Hz, 0.07H), 2.68 (d, *J* = 12.8 Hz, 0.93H), 3.23 (d, *J* = 12.7 Hz, 0.93H), 3.27 (d, *J* = 17.2 Hz, 0.93H), 3.34 (d, *J* = 12.7 Hz, 0.07H), 3.39 – 3.51 (m, 0.14H), 3.60 (d, *J* = 17.2 Hz, 0.93H), 4.17 (q, *J* = 7.2 Hz, 1.86H), 4.18 – 4.23 (m, 0.14H), 7.00 (d, *J* = 8.3 Hz, 2H), 7.26 (d, *J* = 8.3 Hz, 2H), 7.49 (br.s. 0.93H), 8.29 (br.s. 0.07H). ^{13}C NMR (75.48 MHz, $CDCl_3$), δ: 14.0, 14.2, 15.2, 15.5, 18.2, 24.3, 24.6, 25.4, 26.0, 34.9, 40.9, 41.5, 56.5, 56.8, 61.7, 101.7, 102.1, 105.4, 114.2, 114.2, 128.5, 131.3, 133.2, 134.0, 134.5, 159.2, 165.3, 171.8, 173.6. Anal. Calcd. for $C_{20}H_{24}ClN_3O_5$: C, 56.94; H, 5.73; Cl, 8.40; N, 9.96. Found: 57.05; H, 5.84; Cl, 8.52; N, 10.07. HRMS (ESI-TOF): m/z $[M+Na]^+$: calculated for $[C_{20}H_{24}ClN_3NaO_5]^+$: 444.1297; found: 444.1290.

Ethyl 8-(2-cyanoacetamido)-2-(4-fluorobenzyl)-1,5-dimethyl-6,7-dioxa-8-azabicyclo[3.2.1]octane-2-carboxylate, 26a + 26b

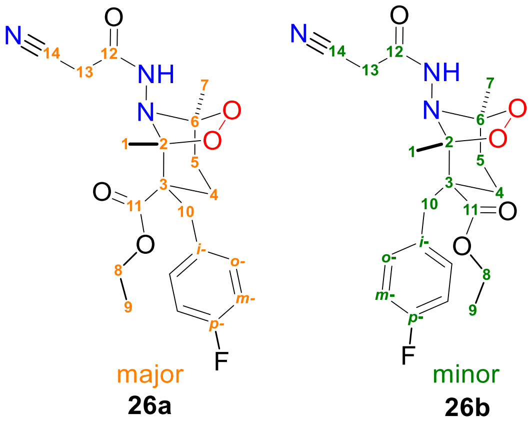

major **26a** minor **26b**

White crystals. Mp = 93 – 95 °C. **26a : 26b** = 92 : 8. R_f= 0.23 (TLC, PE : EA, 2 : 1).

26a: ^{1}H NMR (300.13 MHz, $CDCl_3$), δ: 1.17 (t, *J* = 7.2 Hz, 3H, (**H**^{9})), 1.51 (s, 3H, (**H**^{7})), 1.66 – 1.76 (m, 1H, (**H**^{4})), 1.79 (s, 3H, (**H**^{1})), 1.84 – 2.04 (m, 2H, (**H**^{4}, **H**^{5})), 2.10 – 2.25 (m, 1H, (**H**^{5})), 2.67 (d, *J* = 12.9 Hz, 1H, (**H**^{10})), 3.20 (d, *J* = 12.9 Hz, 1H, (**H**^{10})), 3.25 (d, *J* = 17.1 Hz, 1H, (**H**^{13})), 3.58 (d, *J* = 17.1 Hz, 1H, (**H**^{13})), 4.15 (q, *J* = 7.2 Hz, 2H, (**H**^{8})), 6.88 – 7.05 (m, 4H, (**H**$^{m-}$, **H**$^{o-}$)), 7.43 (br.s., 1H, (N**H**)). ^{13}C NMR (75.48 MHz, $CDCl_3$), δ: 13.95 (**C**^{9}), 15.48 (**C**^{1}), 18.25 (**C**^{7}), 24.26 (**C**^{13}), 25.45 (**C**^{4}), 34.9 (**C**^{5}), 41.4 (**C**^{10}), 56.95 (**C**^{3}), 61.7 (**C**^{8}), 101.7 (**C**^{6}), 105.42 (**C**^{2}), 114.22 (**C**^{14}), 115.2 (d, $^{2}J_{CF}$ = 21.3 Hz, (**C**$^{m-}$)), 131.3 (d, $^{3}J_{CF}$ = 7.8 Hz, (**C**$^{o-}$)), 132.3 (d, $^{4}J_{CF}$ = 3.1 Hz, (C^{i-})), 162.1(d, $^{1}J_{CF}$ = 245.4 Hz, (C^{p-})), 165.3 (**C**^{12}), 173.6 (**C**^{11}). ^{15}N (40.56 MHz, $CDCl_3$), δ: 137.7 (**N**), 141.1 (N**H**), 258.8 (C≡**N**).

26b: ^{1}H NMR (300.13 MHz, $CDCl_3$), δ: 1.31 (t, *J* = 7.1 Hz, 3H, (**H**^{9})), 1.42 (s, 3H, (**H**^{7})), 1.66 – 1.76 (m, 1H, (**H**^{4})), 1.75 (s, 3H, (**H**^{1})), 1.84 – 2.04 (m, 2H, (**H**^{4}, **H**^{5})), 2.10 – 2.25 (m, 1H, (**H**^{5})), 2.57 (d, *J* = 12.9 Hz, 1H, (**H**^{10})), 3.32 (d, *J* = 12.9 Hz, 1H, (**H**^{10})), 3.38 – 3.47 (m, 2H, , (**H**^{13})), 4.23 – 4.33 (m, 2H, (**H**^{8})), 6.88 – 7.05 (m, 4H, (**H**$^{m-}$, **H**$^{o-}$)), 8.23 (br.s., 1H, (N**H**)). ^{13}C NMR (75.48 MHz, $CDCl_3$), δ: 14.2 (**C**^{9}), 15.21 (**C**^{1}), 18.25 (**C**^{7}), 24.56 (**C**^{13}), 25.97 (**C**^{4}), 34.9 (**C**^{5}), 40.8 (**C**^{10}), 56.59 (**C**^{3}), 61.7 (**C**^{8}), 102.1 (**C**^{6}), 105.29 (**C**^{2}), 114.17

(**C14**), 115.2 (d, $^{2}J_{CF}$ = 21.3 Hz, (**C*m*-**)), 131.3 (d, $^{3}J_{CF}$ = 7.8 Hz, (**C*o*-**)), 132.3 (d, $^{4}J_{CF}$ = 3.1 Hz, (C*i*-)), 159.2 (**C12**), 162.1(d, $^{1}J_{CF}$ = 245.4 Hz, (C*p*-)), 171.9 (**C11**). ^{15}N (40.56 MHz, $CDCl_3$), δ: 260.0 (C≡**N**).

Anal. Calcd. for$C_{20}H_{24}FN_3O_5$: C, 59.25; H, 5.97; F, 4.69; N, 10.36. Found: C, 59.38; H, 6.09; F, 4.81; N, 10.45. HRMS (ESI-TOF): m/z $[M+Na]^+$: calculated for$[C_{20}H_{24}FN_3NaO_5]^+$: 428.1592; found: 428.1585.

Ethyl 2-benzyl-8-(2-cyanoacetamido)-1,5-dimethyl-6,7-dioxa-8-azabicyclo[3.2.1]octane-2-carboxylate, 27a + 27b

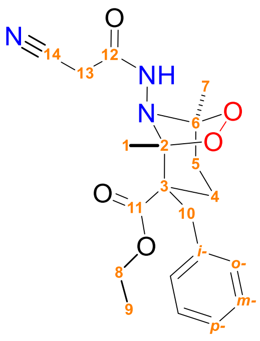

major
27a

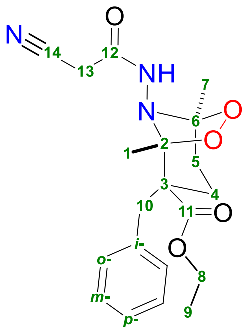

minor
27b

White crystals. Mp =149 – 151°C. **27a : 27b** = 92 : 8. R_f= 0.25 (TLC, PE : EA, 2 : 1).

27a: ^{1}H NMR (300.13 MHz, $CDCl_3$), δ: 1.18 (t, *J* = 7.2 Hz, 3H, (**H9**)), 1.53 (s, 3H, (**H7**)), 1.69 – 1.80 (m, 1H, (**H4**)), 1.83 (s, 3H, (**H1**)), 1.85 – 2.26 (m, 3H, (**H4**, **H5**)), 2.70 (d, *J* = 12.9 Hz, 1H, (**H10**)), 3.26 (d, *J* = 12.9 Hz, 1H, (**H10**)), 3.29 (d, *J* = 17.1 Hz, 1H, (**H13**)), 3.63 (d, *J* = 17.1 Hz, 1H, (**H13**)), 4.18 (q, *J* = 7.2 Hz, 2H, (**H8**)), 7.01 – 7.11 (m, 2H, (**H*o*-**)), 7.19 – 7.33 (m, 3H, (**H*m*-**, **H*p*-**)), 7.70 (br.s., 1H, (N**H**). ^{13}C NMR (75.48 MHz, $CDCl_3$), δ: 13.87 (**C9**), 15.46 (**C1**), 18.2 (**C7**), 24.2 (**C13**), 25.5 (**C4**), 34.9 (**C5**), 41.2 (**C10**), 57.0 (**C3**), 61.5 (**C8**), 101.6 (**C6**), 105.46 (**C2**), 114.2 (**C14**), 127.1 (**C*p*-**), 128.3 (**C*m*-**),130.0 (**C*o*-**), 135.5 (**C*i*-**), 165.5 (**C12**), 173.7 (**C11**). ^{15}N (40.56 MHz, $CDCl_3$), δ: 141.45 (**N**), 141.65 (NH), 252.4 (C≡**N**).

27b: ^{1}H NMR (300.13 MHz, $CDCl_3$), δ: 1.34 (t, *J* = 7.1 Hz, 3H, (**H9**)), 1.44 (s, 3H, (**H7**)), 1.69 – 1.80 (m, 1H, (**H4**)), 1.79 (s, 3H, (**H1**)), 1.85 – 2.26 (m, 3H, (**H4**, **H5**)), 2.62 (d, *J* = 12.9 Hz, 1H, (**H10**)), 3.49 (d, *J* = 17.1 Hz, 1H, (**H13**)), 4.26 – 4.35 (m, 2H, (**H8**)), 7.01 – 7.11 (m, 2H, (**H*o*-**)), 7.19 – 7.33 (m, 3H, (**H*m*-**, **H*p*-**)), 8.49 (br.s., 1H, (N**H**). ^{13}C NMR (75.48 MHz, $CDCl_3$), δ: 14.13 (**C9**), 15.19 (**C1**), 18.21 (**C7**), 24.56 (**C13**), 25.97 (**C4**), 34.9 (**C5**), 41.56 (**C10**), 56.55 (**C3**), 64.0 (**C8**), 102.1 (**C6**), 105.39 (**C2**), 114.36 (**C14**), 126.90 (**C*p*-**), 128.3 (**C*m*-**), 130.0 (**C*o*-**), 136.0 (**C*i*-**), 159.5 (**C12**), 172.0 (**C11**). ^{15}N (40.56 MHz, $CDCl_3$), δ: 136.1 (**N**), 138.3 (NH), 249.3 (C≡**N**). Anal. Calcd. for$C_{20}H_{25}N_3O_5$: C, 62.00; H, 6.50; N, 10.85. Found: C, 62.15; H, 6.68; N, 10.97. HRMS (ESI-TOF): m/z $[M+Na]^+$: calculated for$[C_{20}H_{25}N_3NaO_5]^+$: 410.1686; found: 410.1681.

Allyl 2-allyl-8-(2-cyanoacetamido)-1,5-dimethyl-6,7-dioxa-8-azabicyclo[3.2.1]octane-2-carboxylate, 28a + 28b

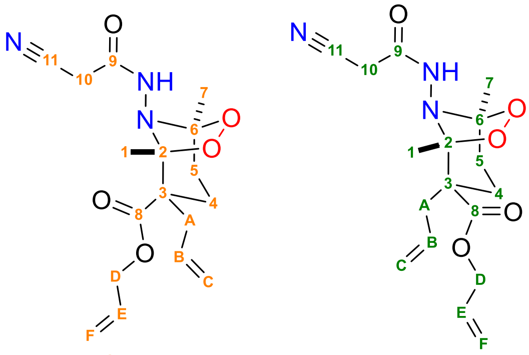

major **28a** minor **28b**

White crystals. Mp = 118 – 120 °C, **28a : 28b** = 92 : 8. R_f= 0.55 (TLC, PE : EA, 2 : 1).

28a: ^{1}H NMR (300.13 MHz, $CDCl_3$), δ: 1.49 (s, 3H, (**H^7**)), 1.67 (s, 3H, (**H^1**)), 1.84 – 2.06 (m, 2H, **H^4**, **H^5**)), 2.08 – 2.40 (m, 3H, **H^4**, **H^5**, **H^A**)), 2.48 – 2.64 (m, 1H, (**H^A**)), 3.28 (d, *J* = 17.4 Hz, 1H, (**H^{10}**)), 3.60 (d, *J* = 17.4 Hz, 1H, (**H^{10}**)), 4.57 – 4.71 (m, 2H, (**H^D**)), 5.06 – 5.17 (m, 2H, (**H^C**)), 5.29 – 5.46 (m, 2H, (**H^F**)), 5.57 – 5.76 (m, 1H, (**H^B**)), 5.86 – 6.05 (m, 1H, (**H^E**)), 7.34 (br.s., 1H, (N**H**)). ^{13}C NMR (75.48 MHz, $CDCl_3$), δ: 15.6 (**C^1**), 18.3 (**C^7**), 24.3 (**C^{10}**), 25.7 (**C^4**), 35.1 (**C^5**), 40.7 (**C^A**), 56.1 (**C^3**), 66.3 (**C^D**), 101.6 (**C^6**), 105.1 (**C^2**), 114.2 (**C^{11}**), 119.5 (**C^C**), 119.8 (**C^F**), 131.6 (**C^E**), 131.9 (**C^B**), 165.4 (**C^9**), 173.2 (**C^8**), ^{15}N (40.56 MHz, $CDCl_3$), δ: 141.1 (**N**), 141.6 (**N**H), 252.3 (C≡**N**).

28b: ^{1}H NMR (300.13 MHz, $CDCl_3$), δ: 1.41 (s, 3H, (**H^7**)), 1.65 (s, 3H, (**H^1**)), 1.84 – 2.06 (m, 2H, **H^4**, **H^5**)), 2.08 – 2.40 (m, 3H, **H^4**, **H^5**, **H^A**)), 2.48 – 2.64 (m, 1H, (**H^A**)), 3.44 (d, *J* = 17.4 Hz, 1H, (**H^{10}**)), 3.70 (d, *J* = 17.4 Hz, 1H, (**H^{10}**)), 4.57 – 4.71 (m, 2H, (**H^D**)), 5.06 – 5.17 (m, 2H, (**H^C**)), 5.29 – 5.46 (m, 2H, (**H^F**)), 5.57 – 5.76 (m, 1H, (**H^B**)), 5.86 – 6.05 (m, 1H, (**H^E**)), 8.22 (br.s., 1H, (N**H**)). ^{13}C NMR (75.48 MHz, $CDCl_3$), δ: 15.3 (**C^1**), 18.3 (**C^7**), 24.6 (**C^{10}**), 25.8 (**C^4**), 35.1 (**C^5**), 40.3 (**C^A**), 55.9 (**C^3**), 66.3 (**C^D**), 102.1 (**C^6**), 105.1 (**C^2**), 114.2 (**C^{11}**), 119.5 (**C^C**), 119.8 (**C^F**), 131.6 (**C^E**), 131.9 (**C^B**), 165.4 (**C^9**), 173.2 (**C^8**). ^{15}N (40.56 MHz, $CDCl_3$), δ:136.3 (**N**), 137.9 (**N**H), 253.8 (C≡**N**). Anal. Calcd. for$C_{17}H_{23}N_3O_5$: C, 58.44; H, 6.64; N, 12.03. Found: C, 58.60; H, 6.78; N, 12.21. HRMS (ESI-TOF): m/z [M+Na]$^+$: calculated for[$C_{17}H_{23}N_3NaO_5$]$^+$: 372.1530; found: 372.1525.

Synthesis of aminoperoxides 29, 30

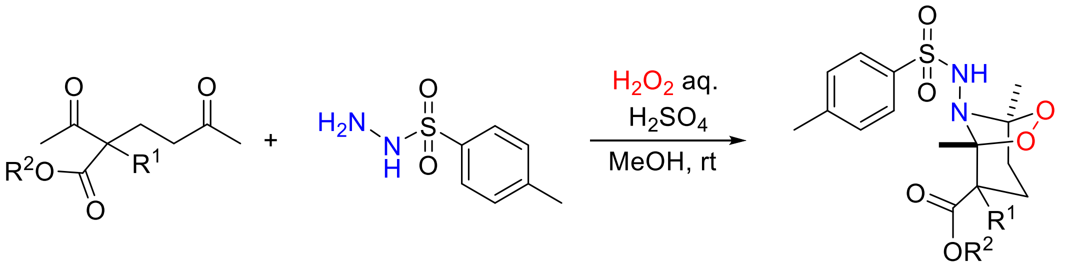

29: R^1 = 4-chlorobenzyl, R^2 = Et;
30: R^1 = 4-fluorobenzyl, R^2 = Et

Aminoperoxides **29, 30** were synthesized according to a known procedure.[10]

Aminoperoxide **29** is known compound.[10] Aminoperoxide **30** is new compound.

Procedure for the synthesis of aminoperoxides 29 and 30

A 98% H_2SO_4 (66 – 69 µL, 1.24 – 1.30 mmol, 2.0 mol of H_2SO_4 / 1.0 mol of **A**), 4-methylbenzenesulfonohydrazide **B** (115 – 121 mg, 0.62 – 0.65 mmol, 1.0 mol of **B** / 1.0 mol of **A**) and 35 % aq. solution of H_2O_2 (78 – 83 µL, 0.92 – 0.97 mmol, 1.5 mol of H_2O_2 / 1.0 mol of **A**) were successively added with stirring to a solution of 1,5-diketone **A** (0.200 g, 0.62 – 0.65 mmol) in MeOH (10mL). The reaction mixture was stirred at 20 - 25 ° C for 72h (in the case of aminoperoxide **29**) or 6 days (in the case of aminoperoxide 30). Then water (25 mL) was added, and the reaction mixture was stirred for another 15 minutes. The resulting precipitate was filtered and washed with water. Pure peroxides **29** and **30** were obtained. Aminoperoxide **29**: 0.160 g, 0.31 mmol, yield 52%. Aminoperoxide **30:** 0.099 g, 0.20 mmol, yield 31%.

Ethyl 2-(4-chlorobenzyl)-1,5-dimethyl-8-((4-methylphenyl)sulfonamido)-6,7-dioxa-8-azabicyclo[3.2.1]octane-2-carboxylate, 29[10]

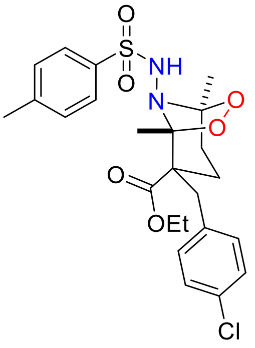

29

White crystals. Mp = 140 – 142 °C (Lit.[10] Mp = 140 – 142 °C).

29: ^{1}H NMR (300.13 MHz, $CDCl_3$), δ: 0.76 (s, 3H), 1.41 (t, *J* = 7.2 Hz, 3H), 1.46 – 1.53 (m, 1H), 1.59 – 1.69 (m, 1H), 1.70 – 1.81 (m, 1H), 1.81 (s, 3H), 2.01 – 2.10 (m, 1H), 2.43 (m, 3H), 2.58 (d, *J* = 12.8 Hz, 1H), 3.37 (d, *J* = 12.8 Hz, 1H), 4.28 – 4.40 (m, 2H), 6.38 (br.s., 1H), 6.97 (d, *J* = 8.3 Hz, 2H), 7.21 (d, *J* = 8.3 Hz, 2H), 7.30 (d, *J* = 8.3 Hz, 2H), 7.74 (d, *J* = 8.3 Hz, 1H). ^{13}C NMR (75.48 MHz, $CDCl_3$), δ: 14.2, 16.2, 17.8, 21.6, 26.2, 35.3, 40.8, 56.7, 61.5, 101.6, 105.7, 128.2, 128.6, 129.7, 131.2, 132.9, 134.9, 136.1, 144.3, 172.3. Anal. Calcd. for $C_{24}H_{29}ClN_2O_6S$: C, 56.63; H, 5.74; Cl, 6.96; N, 5.51; S, 6.31. Found: C, 56.79; H, 5.86; Cl, 7.11; N, 5.67; S, 6.52. HRMS (ESI-TOF): m/z $[M+Na]^+$: calculated for $[C_{24}H_{29}ClN_2NaO_6S]^+$: 531.1327; found: 531.1321.

Ethyl 2-(4-fluorobenzyl)-1,5-dimethyl-8-((4-methylphenyl)sulfonamido)-6,7-dioxa-8-azabicyclo[3.2.1]octane-2-carboxylate, 30

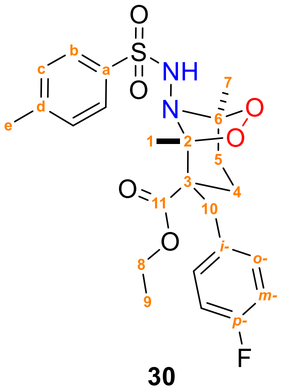

30

White crystals. Mp = 123-125 °C. R_f = 0.66 (TLC, PE : EA, 2 : 1).

30 :^{1}H NMR (300.13 MHz, $CDCl_3$), δ: 0.76 (s, 3H, (**H**^{7})), 1.41 (t, *J* = 7.2 Hz, 3H, (**H**^{9})), 1.45 – 1.57 (m, 1H, (**H**^{4})), 1.64 – 1.85 (m, 1H, (**H**^{4},**H**^{5})), 1.83 (s, 3H, (**H**^{1})), 2.05 – 2.20 (m, 1H, (**H**^{5})), 2.43 (m, 3H, (**H**^{e})), 2.58 (d, *J* = 12.8 Hz, 1H, (**H**^{10})), 3.38 (d, *J* = 12.8 Hz, 1H, (**H**^{10})), 4.25 – 4.44 (m, 2H, (**H**^{8})), 6.38 (br.s., 1H, N**H**), 6.94 – 7.10 (m, 4H, (**H**$^{o-}$, **H**$^{m-}$)), 7.31 (d, *J* = 8.3 Hz, 2H, (**H**^{c})), 7.73 (d, *J* = 8.3 Hz, 1H, (**H**^{b})). ^{13}C NMR (75.48 MHz, $CDCl_3$), δ: 14.2 (**C**^{9}), 16.2 (**C**^{1}), 18.1 (**C**^{7}), 21.8 (**C**^{e}), 26.3 (**C**^{4}), 35.4 (**C**^{5}), 40.9 (**C**^{10}), 56.9 (**C**^{3}), 61.6 (**C**^{8}), 101.6 (**C**^{6}), 105.6 (**C**^{2}), 115.3 (d, $^{2}J_{CF}$ = 21.3 Hz, (**C**$^{m-}$)), 128.2 (**C**^{b}), 129.7 (**C**^{c}), 131.3 (d, $^{3}J_{CF}$ = 7.9 Hz, (**C**$^{o-}$)), 132.0 (d, $^{4}J_{CF}$ = 3.8 Hz, (C^{i-})), 136.1 (**C**^{a}), 144.3 (**C**^{d}), 161.8 (d, $^{1}J_{CF}$ = 244.8 Hz, (C^{p-})), 172.2 (**C**^{11}). ^{15}N (40.56 MHz, $CDCl_3$), δ:126.7 (**NH**), 138.3 (**N**). Anal. Calcd. for $C_{24}H_{29}ClN_2O_6S$: C, 56.63; H, 5.74; Cl, 6.96; N, 5.50; S, 6.30. Found: C, 56.80; H, 5.88; Cl, 7.11; N, 5.67; S, 6.46. HRMS (ESI-TOF): m/z $[M+Na]^+$: calculated for $[C_{24}H_{29}ClN_2NaO_6S]^+$: 531.1327; found: 531.1315.

Synthesis of aminoperoxides 31a + 31b

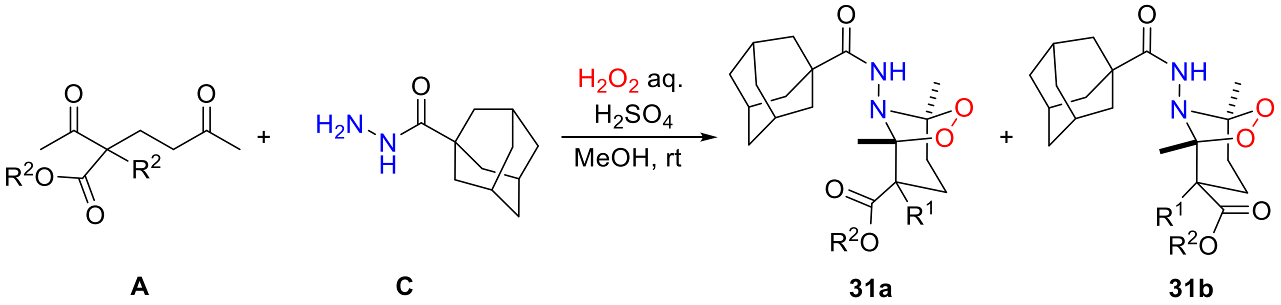

31a+31b: R^1 = 4-chlorobenzyl, R^2 = Et

Aminoperoxides **31a + 31b** were synthesized according to a known procedure.[10]

Aminoperoxides **31a + 31b** are known compounds.[10]

Ethyl 8-((3r,5r,7r)-adamantane-1-carboxamido)-2-(4-chlorobenzyl)-1,5-dimethyl-6,7-dioxa-8-azabicyclo[3.2.1]octane-2-carboxylate, 31a + 31b[10]

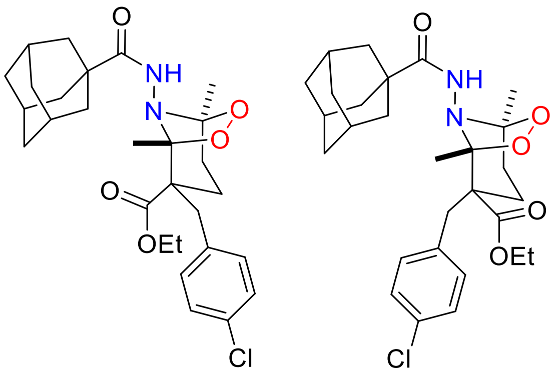

31a **31b**

White crystals. Mp = 127-129 °C (Lit.[10] Mp = 127 – 129 °C). **31a : 31b** = 90 : 10.

31a: ^{1}H NMR (300.13 MHz, $CDCl_3$), δ: 1.30 (t, *J* = 7.2 Hz, 3H), 1.37 (s, 2.7H), 1.45 (s, 0.3H), 1.50 (s, 0.3H), 1.52 – 1.57 (m, 1H), 1.66 – 1.84 (m, 11H), 1.69 (s, 2.7H), 2.01 – 2.12 (m, 6H), 2.26 – 2.37 (m, 1H), 2.57 (d, *J* = 12.7 Hz, 1H), 3.32 (d, *J* = 12.7 Hz, 1H), 4.24 – 4.36 (m, 2H), 6.94 (br.s., 1H), 6.97 (d, *J* = 8.1 Hz, 2H), 7.20 (d, *J* = 8.1 Hz, 2H). ^{13}C NMR (75.48 MHz, $CDCl_3$), δ: 14.2, 15.3, 18.5, 26.0, 28.1, 35.0, 36.5, 39.5, 41.0, 56.4, 61.6, 101.9, 105.2, 128.5, 131.2, 132.8, 134.7, 172.1, 174.8. Anal. Calcd. for $C_{28}H_{37}ClN_2O_5$: C, 65.04; H, 7.21; Cl, 6.86; N, 5.42. Found: C, 65.12; H, 7.35; Cl, 6.97; N, 5.50. HRMS (ESI-TOF): m/z $[M+Na]^+$: calculated for $[C_{28}H_{37}ClN_2NaO_5]^+$: 539.2283; found: 539.2272.

Synthesis of aminoperoxides 32 – 45

Azaperoxides **32 – 45** were synthesized according to a known procedure.[11] Aminoperoxides **32 – 45** are known compounds.[11]

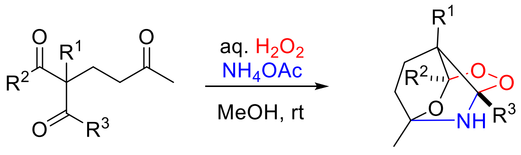

32: R^1 = allyl, R^2 = R^3 = CH_3
33: R^1 = hexyl^n, R^2 = R^3 = CH_3
34: R^1 = isopentyl, R^2 = R^3 = CH_3
35: R^1 = butyl, R^2 = R^3 = CH_3
36: R^1 = methyl, R^2 = R^3 = CH_3
37: R^1 = 4-bromobenzyl, R^2 = R^3 = CH_3
38: R^1 = benzyl, R^2 = R^3 = CH_3
39: R^1 =$CH_2CH_2C(O)OEt$, R^2 = R^3 = CH_3
40: R^1 = CH_2CH_2CN, R^2 = R^3 = CH_3
41: R^1 = ethyl, R^2 = R^3 = CH_3
42: R^1 = octyl^n, R^2 = R^3 = CH_3
43: R^1 = nonyl^n, R^2 = R^3 = CH_3
44: R^1 = 4-nitrobenzyl, R^2 = R^3 = CH_3
45: R^1 =H, R^2 = R^3 = ethyl

3a-Allyl-3,6,7a-trimethylhexahydro-3H-3,6-epoxy[1,2]dioxolo[3,4-b]pyridine, 32[11]

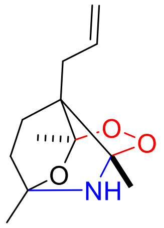

White crystals. Mp = 49 – 51°C. (Lit.[11] Mp = 49 – 51 °C).

^{1}H NMR (300.13 MHz, $CDCl_3$), δ: 1.31 (s, 3H), 1.38 (s, 3H), 1.42 (s, 3H), 1.58 – 1.73 (m, 4H), 2.24 – 2.31 (m, 2H), 2.66 (br.s, 1H), 5.02 – 5.11 (m, 2H), 5.84 – 6.01 (m, 1H). ^{13}C NMR (75.48 MHz, $CDCl_3$), δ: 17.0, 18.7, 20.1, 25.1, 32.9, 35.8, 51.8, 79.8, 95.6, 107.7, 118.1, 134.2. Anal. Calcd for $C_{12}H_{19}NO_3$: C, 63.98; H, 8.50; N, 6.22. Found: C, 64.06; H, 8.65; N, 6.30. HRMS (ESI-TOF): m/z $[M+H]^+$: calculated for $[C_{12}H_{20}NO_3]^+$: 226.1438; found: 226.1445.

3a-Hexyl-3,6,7a-trimethylhexahydro-3H-3,6-epoxy[1,2]dioxolo[3,4-b]pyridine, 33[11]

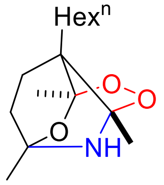

Slightly yellow oil.

^{1}H NMR (300.13 MHz, $CDCl_3$), δ: 0.80 – 0.91 (m, 3H), 1.18 – 1.48 (m, 10H), 1.31 (s, 3H), 1.38 (s, 3H), 1.41 (s, 3H), 1.53 – 1.74 (m, 4H), 2.65 (s, 1H). ^{13}C NMR (75.48 MHz, $CDCl_3$), δ: 14.1, 17.0, 18.7, 19.9, 22.8, 24.4, 25.2, 30.5, 31.3, 31.7, 32.9, 51.7, 79.8, 95.6, 107.9. Anal. Calcd for $C_{15}H_{27}NO_3$: C, 66.88; H, 10.10; N, 5.20.

Found: C, 66.95; H, 10.21; N, 5.26. HRMS (ESI-TOF): m/z $[M+H]^+$: calculated for $[C_{15}H_{28}NO_3]^+$: 270.2064; found: 270.2062.

3a-Isopentyl-3,6,7a-trimethylhexahydro-3H-3,6-epoxy[1,2]dioxolo[3,4-b]pyridine, 34[11]

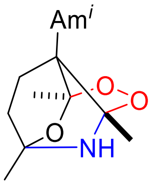

White crystals. Mp = 66 – 68°C. (Lit.[11] Mp = 66 – 68 °C).

^{1}H NMR (300.13 MHz, $CDCl_3$), δ: 0.88 (d, J = 6.6 Hz, 6H), 1.26 – 1.53 (m, 5H), 1.31 (s, 3H), 1.37 (s, 3H), 1.42 (s, 3H), 1.57 – 1.72 (m, 4H), 2.63 (s, 1H). ^{13}C NMR (75.48 MHz, $CDCl_3$), δ: 17.0, 18.8, 20.0, 22.6, 25.2, 29.0, 29.2, 33.0, 33.3, 51.6, 79.8, 95.7, 107.9. Anal. Calcd for $C_{14}H_{25}NO_3$: C, 65.85; H, 9.87; N, 5.49. Found: C, 65.99; H, 9.97; N, 5.60. HRMS (ESI-TOF): m/z $[M+H]^+$: calculated for $[C_{14}H_{26}NO_3]^+$: 256.1907; found: 256.1904.

3a-Butyl-3,6,7a-trimethylhexahydro-3H-3,6-epoxy[1,2]dioxolo[3,4-b]pyridine, 35[11]

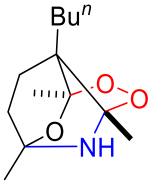

White crystals. Mp = 38 – 40°C. (Lit.[11] Mp = 38 – 40 °C).

^{1}H NMR (300.13 MHz, $CDCl_3$), δ: 0.88 (t, J = 7.6 Hz, 3H), 1.20 – 1.46 (m, 6H), 1.30 (s, 3H), 1.36 (s, 3H), 1.43 (s, 3H), 1.56 – 1.73 (m, 4H), 2.62 (br.s, 1H). ^{13}C NMR (75.48 MHz, $CDCl_3$), δ: 14.0, 17.0, 18.7, 19.9, 23.8, 25.2, 26.6, 31.0, 32.9, 51.7, 79.8, 95.6, 107.9. Anal. Calcd for $C_{13}H_{23}NO_3$: C, 64.70; H, 9.61; N, 5.80. Found: C, 64.80; H, 9.69; N, 5.88. HRMS (ESI-TOF): m/z $[M+H]^+$: calculated for $[C_{13}H_{24}NO_3]^+$: 242.1751; found: 242.1749.

3,3a,6,7a-Tetramethylhexahydro-3H-3,6-epoxy[1,2]dioxolo[3,4-b]pyridine, 36[11]

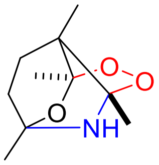

White crystals. Mp = 70 – 72°C. (Lit.[11] Mp = 70 – 72 °C).

^{1}H NMR (300.13 MHz, $CDCl_3$), δ: 0.98 (s, 3H), 1.30 (s, 3H), 1.31 (s, 3H), 1.37 (s, 3H), 1.58 – 1.68 (m, 4H), 2.67 (br.s, 1H). ^{13}C NMR (75.48 MHz, $CDCl_3$), δ: 16.3, 16.8, 17.9, 22.2, 25.2, 33.6, 49.4, 80.1, 95.0, 107.5.

Anal. Calcd for $C_{10}H_{17}NO_3$: C, 60.28; H, 8.60; N, 7.03. Found: C, 60.36; H, 8.72; N, 7.14. HRMS (ESI-TOF): m/z $[M+H]^+$: calculated for $[C_{10}H_{18}NO_3]^+$: 200.1281; found: 200.1280.

3a-(4-Bromobenzyl)-3,6,7a-trimethylhexahydro-3H-3,6-epoxy[1,2]dioxolo[3,4-b]pyridine, 37[11]

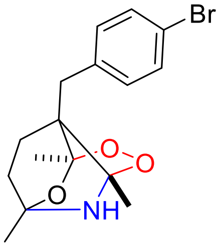

White crystals. Mp = 134 – 136 °C. (Lit.[11] Mp = 134 – 136 °C).

^{1}H NMR (300.13 MHz, $CDCl_3$), δ: 1.31 (s, 3H), 1.35 (s, 3H), 1.37 (s, 3H), 1.58 – 1.68 (m, 2H), 1.73 – 1.86 (m, 2H), 2.70 (br. s, 1H), 2.84 (s, 2H), 7.12 (d, J = 8.2 Hz, 2H), 7.39 (d, J = 8.2 Hz, 2H). ^{13}C NMR (75.48 MHz, $CDCl_3$), δ: 17.7, 19.5, 19.9, 25.1, 32.9, 36.0, 52.8, 79.5, 96.0, 108.0, 120.8, 131.2, 133.1, 136.3. Anal. Calcd for $C_{16}H_{20}BrNO_3$: C, 54.25; H, 5.69; Br, 22.56; N, 3.95. Found: C, 54.37; H, 5.75; Br, 22.63; N, 3.99. HRMS (ESI-TOF): m/z $[M+H]^+$: calculated for $[C_{16}H_{21}BrNO_3]^+$: 354.0699, 356.0680; found: 354.0698, 356.0679.

3a-Benzyl-3,6,7a-trimethylhexahydro-3H-3,6-epoxy[1,2]dioxolo[3,4-b]pyridine, 38[11]

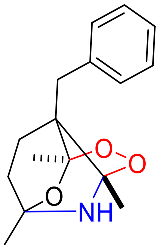

White crystals. Mp = 129 – 131°C. (Lit.[11] Mp = 129 – 131 °C).

^{1}H NMR (300.13 MHz, $CDCl_3$), δ: 1.32 (s, 3H), 1.38 (s, 3H), 1.41 (s, 3H), 1.58 – 1.69 (m, 2H), 1.78 – 1.91 (m, 2H), 2.72 (br.s, 1H), 2.90 (s, 2H). ^{13}C NMR (75.48 MHz, $CDCl_3$), δ: 17.7, 19.4, 19.8, 25.1, 33.0, 36.5, 52.9, 79.4, 96.1, 108.2, 126.7, 128.1, 131.4, 137.3. Anal. Calcd for $C_{16}H_{21}NO_3$: C, 69.79; H, 7.69; N, 5.09. Found: C, 69.84; H, 7.75; N, 5.12. HRMS (ESI-TOF): m/z $[M+H]^+$: calculated for $[C_{16}H_{22}NO_3]^+$: 276.1594; found: 276.1600.

Ethyl 3-(3,6,7a-trimethyltetrahydro-3H-3,6-epoxy[1,2]dioxolo[3,4-b]pyridin-3a(4H)-yl)propanoate, 39[11]

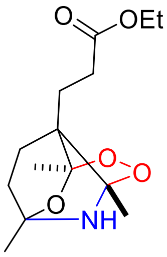

White crystals. Mp = 61 – 63°C. (Lit.[11] Mp = 61 – 63 °C).

^{1}H NMR (300.13 MHz, CDCl$_3$), δ: 1.20 (t, *J* = 7.2 Hz, 3H), 1.28 (s, 3H), 1.35 (s, 3H), 1.40 (s, 3H), 1.50 – 1.66 (m, 4H), 1.72 – 1.85 (m, 2H), 2.45 – 2.57 (m, 2H), 2.65 (br.s, 1H), 4.07 (q, *J* = 7.2 Hz, 1H). ^{13}C NMR (75.48 MHz, CDCl$_3$), δ: 14.2, 16.6, 18.3, 20.9, 25.0, 26.6, 29.7, 32.8, 51.3, 60.5, 79.8, 95.2, 107.3, 173.6. Anal. Calcd for $C_{14}H_{23}NO_5$: C, 58.93; H, 8.13; N, 4.91. Found: C, 59.15; H, 8.32; N, 5.08. HRMS (ESI-TOF): m/z [M+H]$^+$: calculated for [$C_{14}H_{24}NO_5$]$^+$: 286.1649; found: 286.1645.

3-(-3,6,7a-Trimethyltetrahydro-3H-3,6-epoxy[1,2]dioxolo[3,4-b]pyridin-3a(4H)-yl)propanenitrile, 40[11]

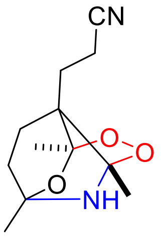

White crystals. Mp = 116 – 118 °C. (Lit.[11] Mp = 116 – 118 °C).

^{1}H NMR (300.13 MHz, CDCl$_3$), δ: 1.30 (s, 3H), 1.38 (s, 3H), 1.42 (s, 3H), 1.56 – 1.72 (m, 4H), 1.80 – 1.89 (m, 2H), 2.58 – 2.68 (m, 2H), 2.72 (br.s, 1H). ^{13}C NMR (75.48 MHz, CDCl$_3$), δ: 13.1, 16.5, 18.1, 21.4, 24.9, 28.3, 32.6, 51.3, 80.0, 95.0, 106.8, 119.9. Anal. Calcd for $C_{12}H_{18}N_2O_3$: C, 60.49; H, 7.61; N, 11.76. Found: C, 60.57; H, 7.72; N, 11.85. HRMS (ESI-TOF): m/z [M+H]$^+$: calculated for [$C_{12}H_{19}N_2O_3$]$^+$: 239.1390; found: 239.1391.

3a-Ethyl-3,6,7a-trimethylhexahydro-3H-3,6-epoxy[1,2]dioxolo[3,4-b]pyridine, 41[11]

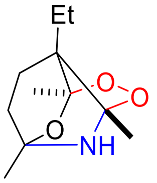

White crystals. Mp = 47 – 49°C. (Lit.[11] Mp = 47 – 49 °C).

^{1}H NMR (300.13 MHz, CDCl$_3$), δ: 0.99 (t, *J* = 7.4 Hz, 3H), 1.30 (s, 3H), 1.37 (s, 3H), 1.41 (s, 3H), 1.48 – 1.72 (m, 6H), 2.63 (br.s, 1H). ^{13}C NMR (75.48 MHz, CDCl$_3$), δ: 9.3, 16.9, 18.6, 19.6, 23.7, 25.2, 32.9, 51.6, 79.8, 95.6, 107.9. Anal. Calcd for $C_{11}H_{19}NO_3$: C, 61.95; H, 8.98; N, 6.57. Found: C, 62.06; H, 9.08; N, 6.68. HRMS (ESI-TOF): m/z [M+H]$^+$: calculated for [$C_{11}H_{20}NO_3$]$^+$: 214.1438; found: 214.1440.

3,6,7a-Trimethyl-3a-octylhexahydro-3H-3,6-epoxy[1,2]dioxolo[3,4-b]pyridine, 42[11]

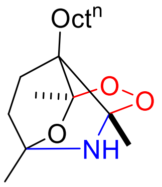

White crystals. Mp = 41 – 43°C. (Lit.[11] Mp = 41 – 43 °C).

^{1}H NMR (300.13 MHz, $CDCl_3$), δ: 0.80 – 0.93 (m, 3H), 1.13 – 1.50 (m, 10H), 1.31 (s, 3H), 1.37 (s, 3H), 1.41 (m, 4H), (s, 3H), 1.56 – 1.75 (m, 4H), 2.63 (br.s, 1H). ^{13}C NMR (75.48 MHz, $CDCl_3$), δ: 14.2, 17.0, 18.7, 19.9, 22.7, 24.4, 25.2, 29.4, 29.5, 30.8, 31.3, 31.9, 32.9, 51.7, 79.8, 95.6, 107.9. Anal. Calcd for $C_{17}H_{31}NO_3$: C, 68.65; H, 10.51; N, 4.71. Found: C, 68.75; H, 10.60; N, 4.81. HRMS (ESI-TOF): m/z $[M+H]^+$: calculated for $[C_{17}H_{32}NO_3]^+$: 298.2377; found: 298.2380.

3,6,7a-Trimethyl-3a-nonylhexahydro-3H-3,6-epoxy[1,2]dioxolo[3,4-b]pyridine, 43[11]

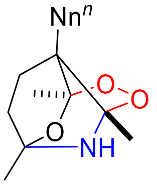

Slightly yellow oil.

^{1}H NMR (300.13 MHz, $CDCl_3$), δ: 0.80 – 0.91 (m, 3H), 1.14 – 1.49 (m, 16H), 1.31 (s, 3H), 1.39 (s, 3H), 1.41 (s, 3H), 1.55 – 1.79 (m, 4H), 2.65 (br.s, 1H). ^{13}C NMR (75.48 MHz, $CDCl_3$), δ: 14.2, 17.0, 18.7, 19.9, 22.8, 24.5, 25.2, 29.4, 29.5, 29.7, 30.8, 31.3, 32.0, 32.9, 51.7, 79.8, 95.6, 107.9. Anal. Calcd for $C_{18}H_{33}NO_3$: C, 69.41; H, 10.68; N, 4.50. Found: C, 69.60; H, 10.75; N, 4.62. HRMS (ESI-TOF): m/z $[M+H]^+$: calculated for $[C_{18}H_{34}NO_3]^+$: 312.2533; found: 312.2539.

3,6,7a-Trimethyl-3a-(4-nitrobenzyl)hexahydro-3H-3,6-epoxy[1,2]dioxolo[3,4-b]pyridine, 44[11]

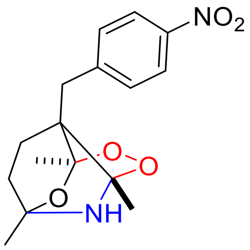

White crystals. Mp = 143 – 145°C. (Lit.[11] Mp = 143 – 145 °C).

^{1}H NMR (300.13 MHz, $CDCl_3$), δ: 1.32 (s, 3H), 1.35 (s, 3H), 1.38 (s, 3H), 1.59 – 1.72 (m, 2H), 1.77 – 1.86 (m, 2H), 2.74 (br. s, 1H), 2.95 – 3.03 (m, 2H), 7.43 (d, J = 8.3 Hz, 2H), 8.13 (d, J = 8.3 Hz, 2H). ^{13}C NMR (75.48 MHz, $CDCl_3$), δ: 17.8, 19.5, 20.0, 25.0, 32.9, 36.6, 53.1, 79.5, 95.9, 107.9, 123.3, 132.2, 145.4, 147.0. Anal. Calcd for $C_{16}H_{20}N_2O_3$: C, 59.99; H, 6.29; N, 8.74. Found: C, 60.15; H, 6.35; N, 8.82. HRMS (ESI-TOF): m/z $[M+H]^+$: calculated for $[C_{16}H_{21}N_2O_3]^+$: 321.1445; found: 321.1448.

3,7a-Diethyl-6-methylhexahydro-3H-3,6-epoxy[1,2]dioxolo[3,4-b]pyridine, 45[11]

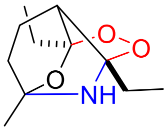

Slightly yellow oil.

^{1}H NMR (300.13 MHz, $CDCl_3$), δ: 0.89 – 1.06 (m, 6H), 1.34 (s, 3H), 1.49 – 1.61 (m, 2H), 1.65 – 1.99 (m, 6H), 2.33 – 2.42 (m, 1H), 2.57 (br. s, 1H), ^{13}C NMR (75.48 MHz, $CDCl_3$), δ: 7.8, 7.9, 14.0, 25.3, 25.9, 27.6, 31.6, 46.1, 80.9, 95.0, 107.0. Anal. Calcd for $C_{11}H_{19}NO_3$: C, 61.95; H, 8.98; N, 6.57. Found: C, 62.12; H, 9.12; N, 6.63. HRMS (ESI-TOF): m/z $[M+H]^+$: calculated for $[C_{11}H_{20}NO_3]^+$: 214.1438; found: 214.1435.

Synthesis of ozonides 46 – 52

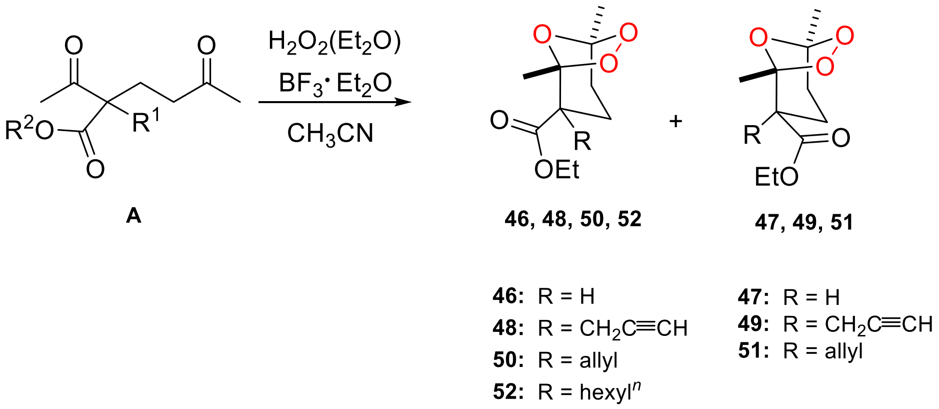

Ozonides **46 – 52** were synthesized according to a known procedure.[12]

Ozonides **46 – 52** are known compounds.[12]

Ethyl (1*R**,2*S**,5*S**)-1,5-dimethyl-6,7,8-trioxabicyclo[3.2.1]octane-2-carboxylate, 46[12]

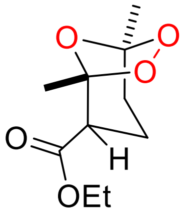

Colorless oil. ^{1}H NMR (300.13 MHz, $CDCl_3$), δ: 1.27 (t, J = 7.1 Hz, 3H), 1.51 (s, 3H), 1.61 (s, 3H), 1.74 (dd, J = 13.3, 6.0 Hz, 1H), 1.90 (dd, J = 5.8, 13.3 Hz, 1H), 2.06 – 2.22 (m, 1H), 2.29 – 2.46 (m, 1H), 2.74 (d, J = 6.1 Hz, 1H), 4.16 (q, J = 7.1 Hz, 2H). ^{13}C NMR (75.48 MHz, $CDCl_3$), δ: 14.3, 20.5, 21.0, 21.1, 31.1, 46.8, 60.9, 108.1, 110.0, 171.3. Anal. Calcd. for $C_{10}H_{16}O_5$: C, 55.55; H, 7.46. Found: C, 55.70; H, 7.55. HRMS (ESI-TOF): m/z $[M+Na]^+$: calculated for $[C_{10}H_{16}NaO_5]^+$: 239.0890; found: 239.0893.

Ethyl (1*S**,2*S**,5*R**)-1,5-dimethyl-6,7,8-trioxabicyclo[3.2.1]octane-2-carboxylate, 47[12]

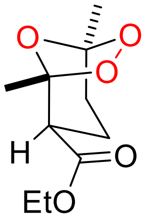

White crystals. Mp = 50 – 51 °C (Lit.[12] Mp = 49 – 50 °C). ^{1}H NMR (300.13 MHz, $CDCl_3$), δ: 1.26 (t, *J* = 7.1 Hz, 3H), 1.51 (s, 3H), 1.57 (s, 3H), 1.72 – 1.97 (m, 3H), 2.36 – 2.58 (m, 1H), 2.77 (dd, *J* = 12.3, 4.9 Hz, 1H), 4.16 (q, *J* = 7.1 Hz, 2H). ^{13}C NMR (75.48 MHz, $CDCl_3$), δ: 14.3, 20.4, 21.0, 21.3, 33.4, 49.3, 60.9, 107.7, 108.7, 171.6. Anal. Calcd. for $C_{10}H_{16}O_5$: C, 55.55; H, 7.46. Found: C, 55.69; H, 7.62. HRMS (ESI-TOF): m/z $[M+Na]^+$: calculated for $[C_{10}H_{16}NaO_5]^+$: 239.0890; found: 239.0892.

Ethyl (1*R,2*R**,5*S**)-1,5-dimethyl-2-(prop-2-yn-1-yl)-6,7,8-trioxabicyclo[3.2.1]octane-2-carboxylate, 48**[12]

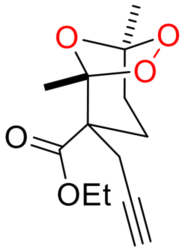

Slightly yellow oil. ^{1}H NMR (300.13 MHz, $CDCl_3$), δ: 1.25 (t, *J* = 7.1 Hz, 3H), 1.46 (s, 3H), 1.58 (s, 3H), 1.79 (dd, *J* = 12.7, 5.2 Hz, 1H), 1.88 – 2.00 (m, 2H), 2.10 – 2.36 (m, 3H), 2.75 (dd, *J* = 16.2, 2.6 Hz, 1H), 4.19 (q, *J* = 7.1 Hz, 2H). ^{13}C NMR (75.48 MHz, $CDCl_3$), δ: 14.1, 18.3, 20.5, 25.6, 26.2, 32.9, 52.3, 61.3, 71.1, 79.0, 109.7, 110.0, 171.4. Anal. Calcd. for $C_{13}H_{18}O_5$:C, 61.41; H, 7.14. Found: C, 61.60; H, 7.25. HRMS (ESI-TOF): m/z $[M+Na]^+$: calculated for $[C_{13}H_{18}NaO_5]^+$: 277.1046; found: 277.1049.

Ethyl (1*S,2*R**,5*R**)-1,5-dimethyl-2-(prop-2-yn-1-yl)-6,7,8-trioxabicyclo[3.2.1]octane-2-carboxylate, 49**[12]

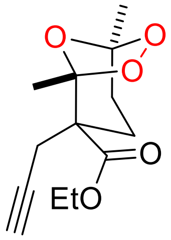

White crystals. Mp = 61 – 63 °C Lit.[12] Mp = 62 – 64 °C). ^{1}H NMR (300.13 MHz, $CDCl_3$), δ: 1.27 (t, *J* = 7.1 Hz, 3H), 1.49 (s, 3H), 1.52 (s, 3H), 1.77 – 1.86 (m, 2H), 1.96 – 2.06 (m, 2H), 2.64 (dd, *J* = 16.7, 2.7 Hz, 1H), 2.70 – 2.89 (m, 2H), 4.21 (q, *J* = 7.1 Hz, 2H). ^{13}C NMR (75.48 MHz, $CDCl_3$), δ: 14.2, 18.6, 20.6, 22.5, 23.5, 30.8, 52.7, 61.5, 71.2, 80.2, 109.3, 109.9, 171.6. Anal. Calcd. for $C_{13}H_{18}O_5$:C, 61.41; H, 7.14. Found: C, 61.58; H, 7.33. HRMS (ESI-TOF): m/z $[M+Na]^+$: calculated for $[C_{13}H_{18}NaO_5]^+$: 277.1046; found: 277.1040.

Ethyl (1*R,2*R**,5*S**)-2-allyl-1,5-dimethyl-6,7,8-trioxabicyclo[3.2.1]octane-2-carboxylate, 50**[12]

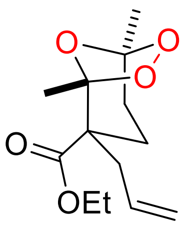

Slightly yellow oil. ^{1}H NMR (300.13 MHz, $CDCl_3$), δ: 1.26 (t, J = 7.1 Hz, 3H), 1.48 (s, 3H), 1.69 (s, 3H), 1.74 – 1.83 (m, 1H), 1.91 – 2.23 (m, 4H), 2.63 (dd, J = 13.2, 6.8 Hz, 1H), 4.19 (q, J = 7.1 Hz, 2H), 5.00 – 5.17 (m, 2H), 5.52 – 5.68 (m, 1H).^{13}C NMR (75.48 MHz, $CDCl_3$), δ: 14.3, 18.7, 20.7, 25.9, 32.9, 39.8, 53.1, 61.1, 109.8, 110.9, 118.9, 132.4, 172.4. Anal. Calcd. for $C_{13}H_{20}O_5$: C, 60.92; H, 7.87. Found: C, 61.08; H, 7.94. HRMS (ESI-TOF): m/z $[M+Na]^+$: calculated for $[C_{13}H_{20}NaO_5]^+$: 279.1203; found: 279.1215.

Ethyl (1*S,2*R**,5*R**)-2-allyl-1,5-dimethyl-6,7,8-trioxabicyclo[3.2.1]octane-2-carboxylate, 51**[12]

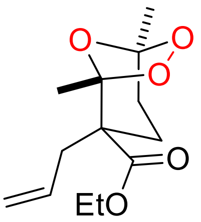

Slightly yellow oil. ^{1}H NMR (300.13 MHz, $CDCl_3$), δ: 1.26 (t, J = 7.1 Hz, 3H), 1.50 (s, 3H), 1.57 (s, 3H), 1.61 – 1.81 (m, 3H), 2.47 (dd, J = 13.9, 8.7 Hz, 1H), 2.58 – 2.75 (m, 2H), 4.16 (q, J = 7.1 Hz, 2H), 5.05 – 5.14 (m, 2H), 5.56 –5.72 (m, 1H). ^{13}C NMR (75.48 MHz, $CDCl_3$), δ: 14.3, 18.8, 20.8, 22.7, 30.8, 36.4, 52.8, 61.2, 109.0, 110.9, 118.7, 133.7, 172.6. Anal. Calcd. for $C_{13}H_{20}O_5$: C, 60.92; H, 7.87. Found: C, 61.11; H, 8.00. HRMS (ESI-TOF): m/z $[M+Na]^+$: calculated for $[C_{13}H_{20}NaO_5]^+$: 279.1203; found: 279.1209.

Ethyl (1*R,2*S**,5*S**)-2-hexyl-1,5-dimethyl-6,7,8-trioxabicyclo[3.2.1]octane-2-carboxylate, 52**[12]

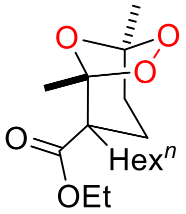

Slightly yellow oil. ^{1}H NMR (300.13 MHz, $CDCl_3$), δ: 0.86 (t, J = 7.1 Hz, 3H), 0.90 – 1.09 (m, 1H), 1.17 – 1.35 (m, 10H), 1.37 – 1.52 (m, 1H), 1.47 (s, 3H), 1.68 (s, 3H), 1.71 – 1.98 (m, 3H), 2.05 – 2.20 (m, 2H), 4.14 – 4.24 (m, 2H). ^{13}C NMR (75.48 MHz, $CDCl_3$), δ: 14.1, 14.3, 18.7, 18.8, 18.8, 20.6, 20.7, 20.7, 22.7, 23.8, 25.7, 29.7, 29.8, 29.8, 31.7, 31.7, 31.7, 33.0, 35.2, 53.4, 60.9, 109.6, 111.4, 173.1. Anal. Calcd. for $C_{16}H_{28}O_5$: C, 63.97; H, 9.40. Found: C, 64.11; H, 9.59. HRMS (ESI-TOF): m/z $[M+Na]^+$: calculated for $[C_{16}H_{28}NaO_5]^+$: 323.1829; found: 323.1832.

NMR spectra of peroxides 1a – 5a + 1b – 5b, 6, 7, 8a' + 8a'' + 8b' + 8b'', 9a – 11a, 12a + 12b, 13a, 14a, 15a-23a + 15b – 23b, 24, 25a – 28a + 25b – 28b, 29, 30, 31a + 31b, 32 – 45, 46 – 52

^{1}H NMR (300.13 MHz, $CDCl_3$). Ethyl 2-(4-fluorobenzyl)-1,5-dimethyl-6,7-dioxa-8-azabicyclo[3.2.1]octane-2-carboxylate, 1a+1b

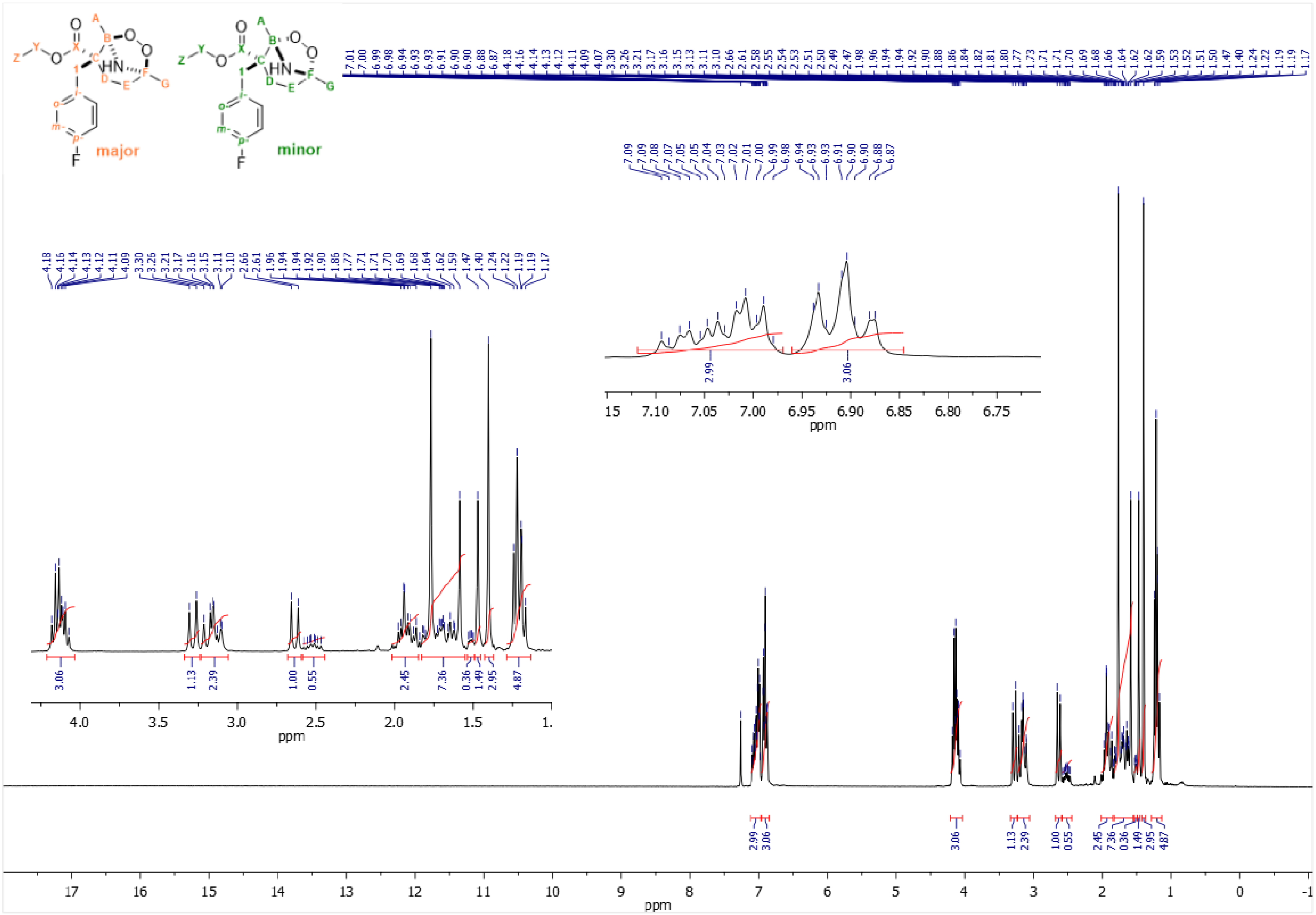

^{1}H NMR (300.13 MHz, CDCl3). Ethyl 2-(4-fluorobenzyl)-1,5-dimethyl-6,7-dioxa-8-azabicyclo[3.2.1]octane-2-carboxylate, 1a+1b

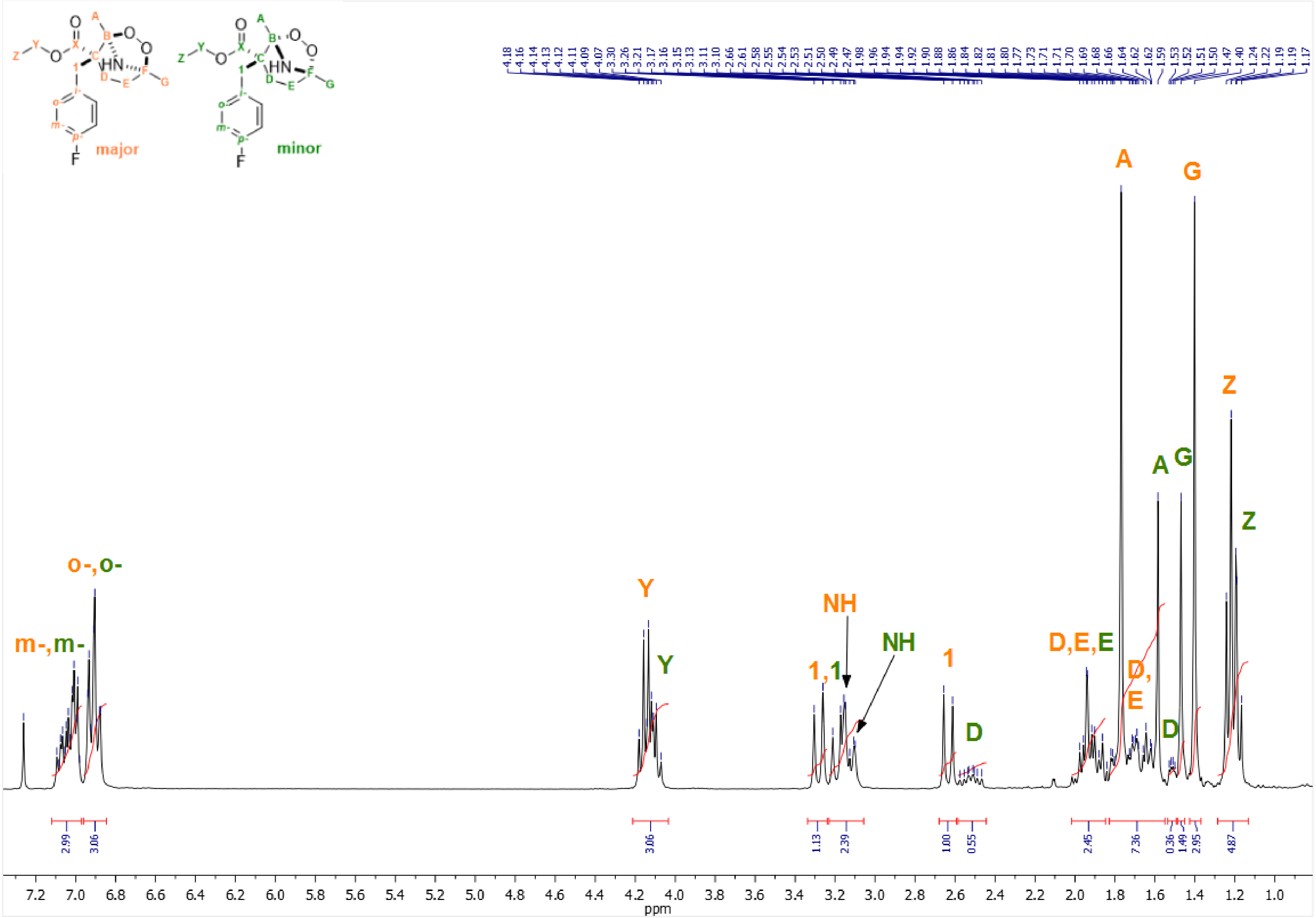

^{13}C NMR (75.48 MHz, CDCl3). Ethyl 2-(4-fluorobenzyl)-1,5-dimethyl-6,7-dioxa-8-azabicyclo[3.2.1]octane-2-carboxylate, 1a+1b

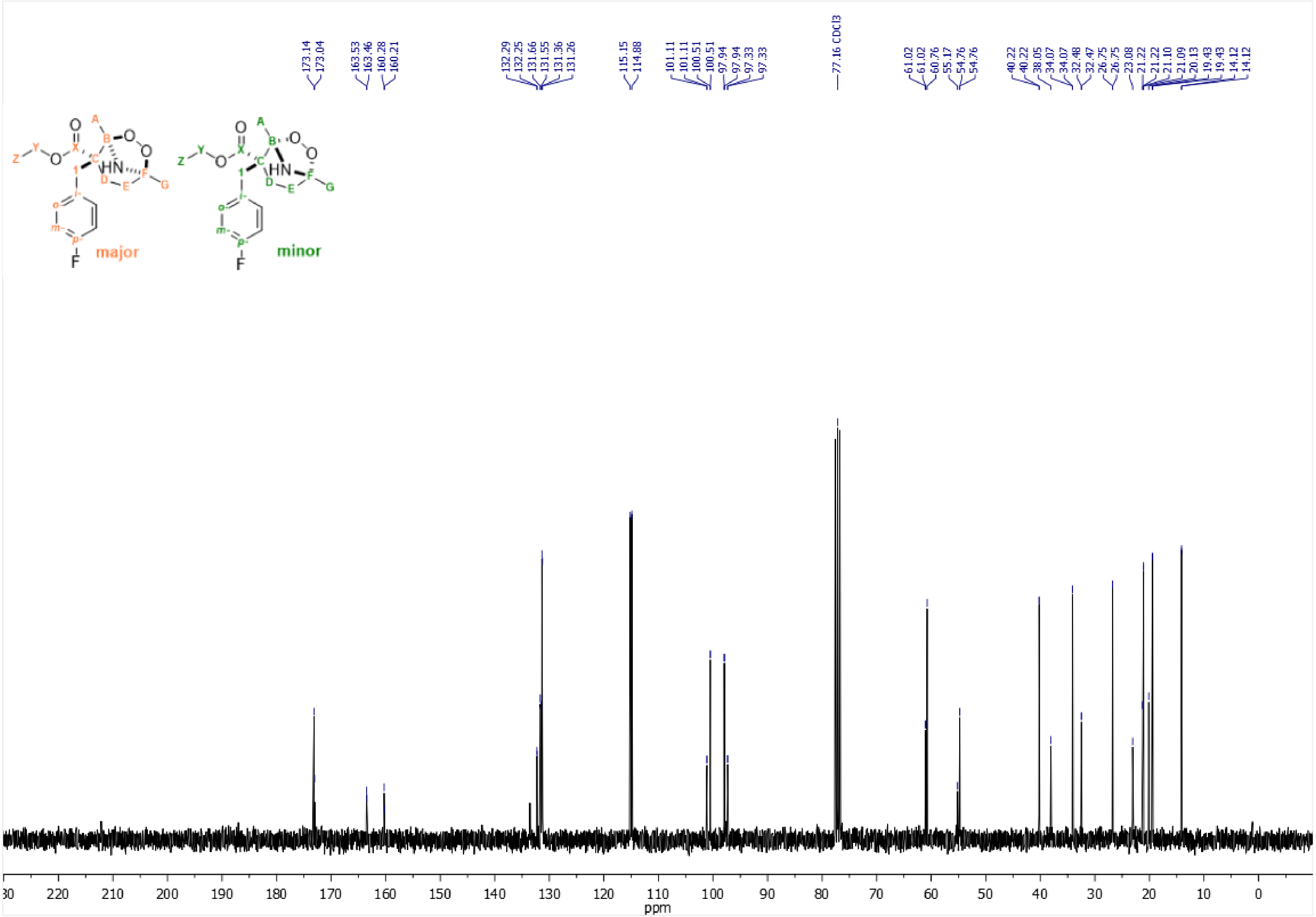

^{13}C NMR (75.48 MHz, $CDCl_3$). Ethyl 2-(4-fluorobenzyl)-1,5-dimethyl-6,7-dioxa-8-azabicyclo[3.2.1]octane-2-carboxylate, 1a+1b

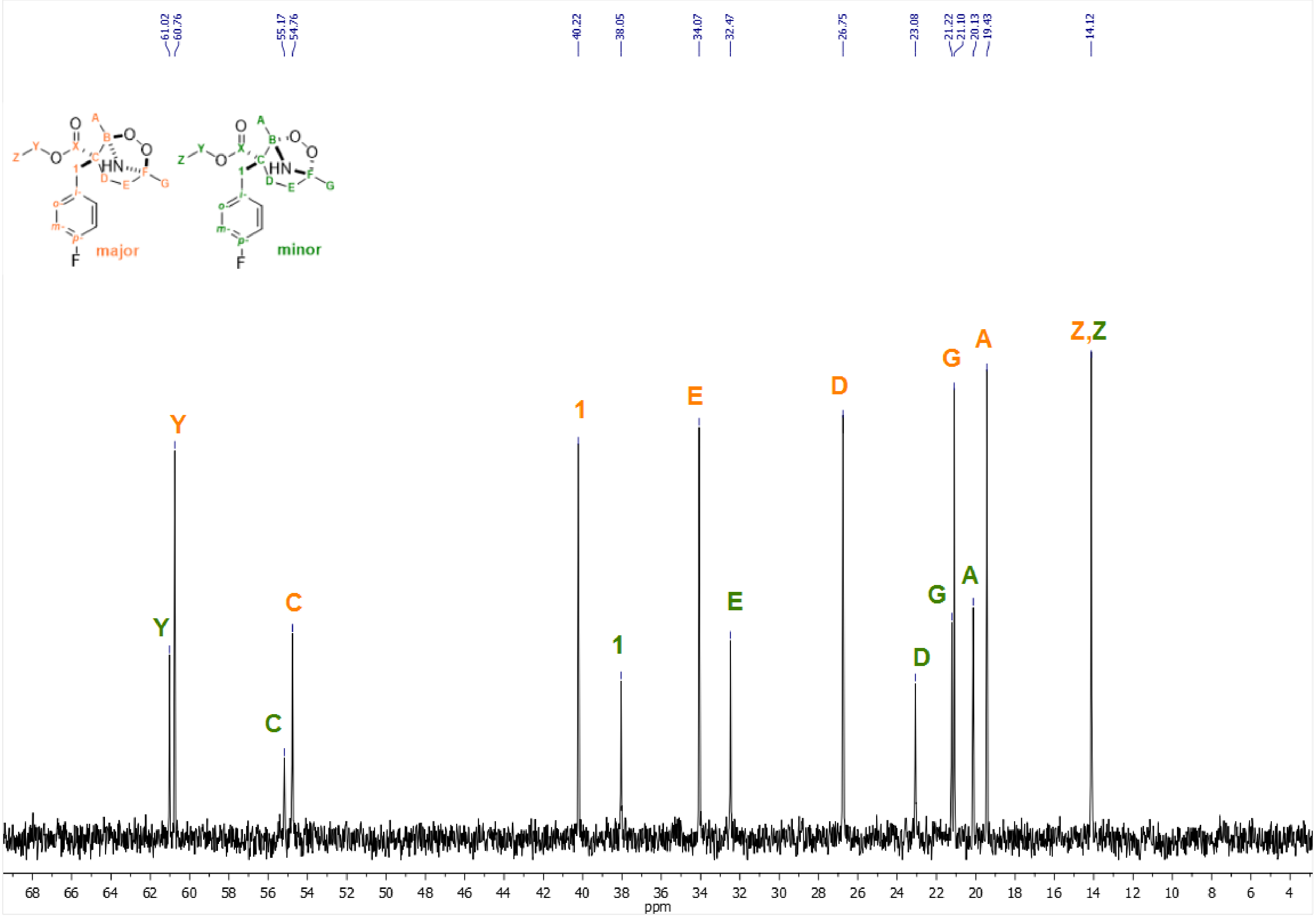

^{13}C NMR (75.48 MHz, CDCl$_3$). Ethyl 2-(4-fluorobenzyl)-1,5-dimethyl-6,7-dioxa-8-azabicyclo[3.2.1]octane-2-carboxylate, 1a+1b

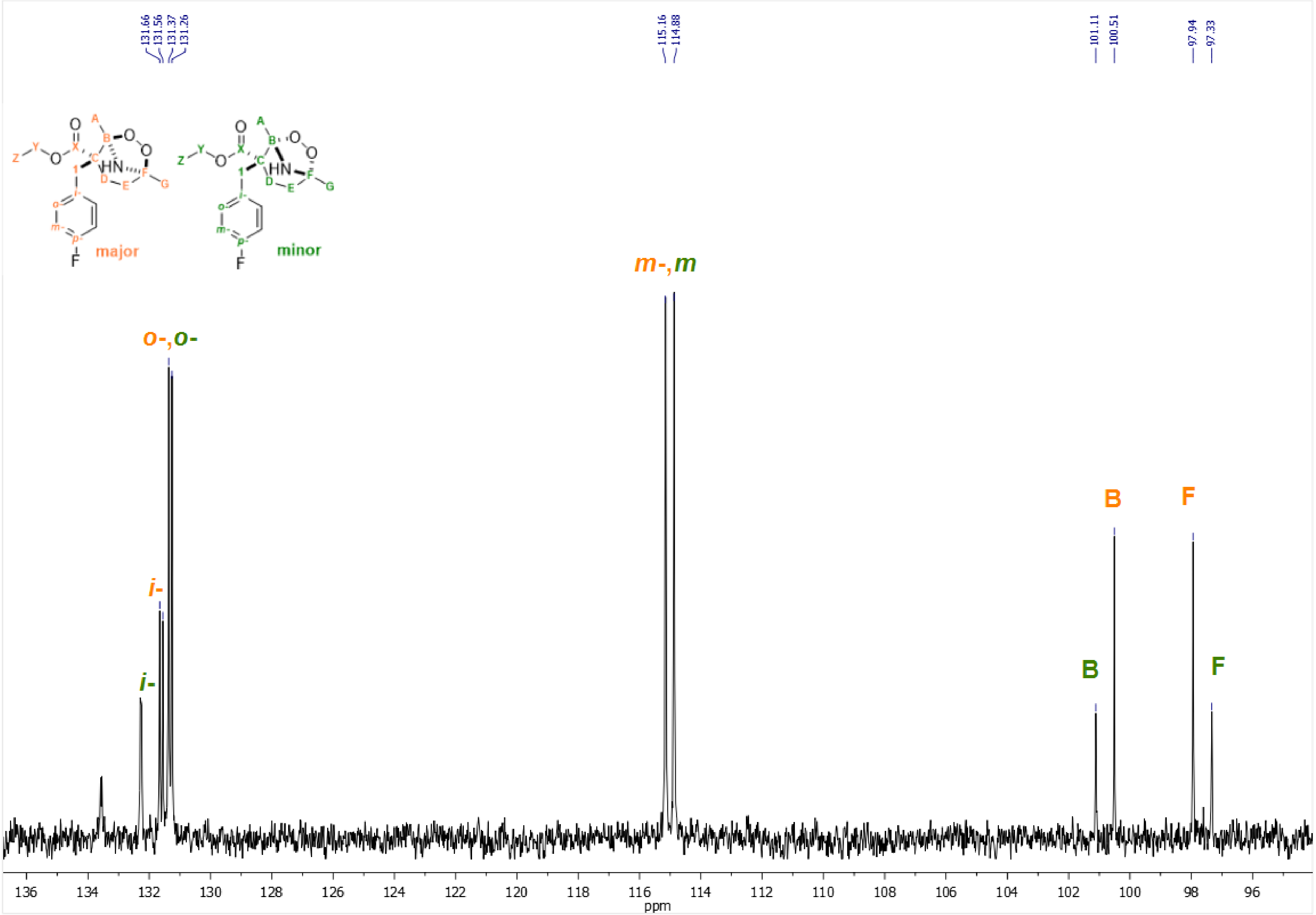

^{13}C NMR (75.48 MHz, CDCl3). Ethyl 2-(4-fluorobenzyl)-1,5-dimethyl-6,7-dioxa-8-azabicyclo[3.2.1]octane-2-carboxylate, 1a+1b

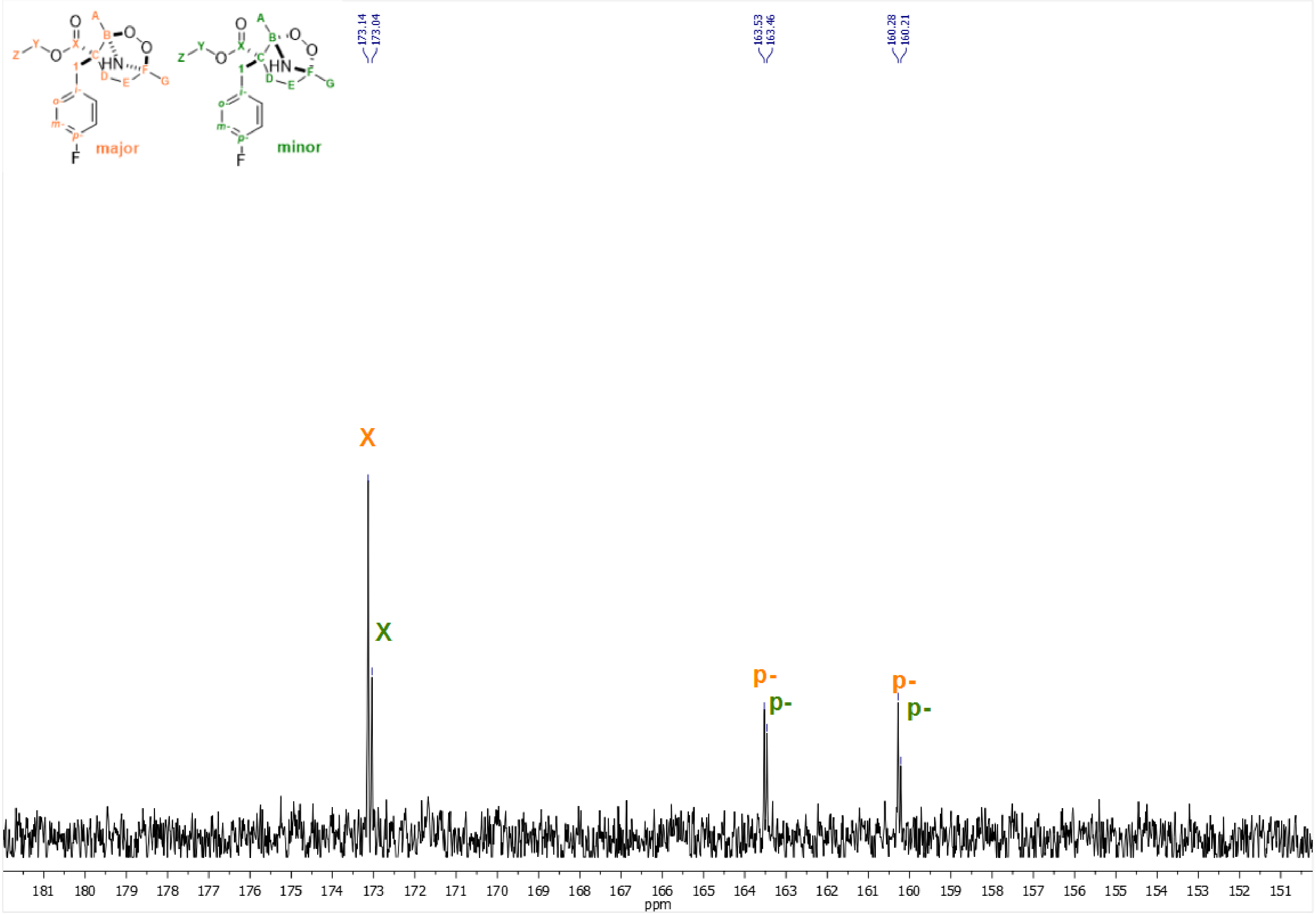

^{1}H NMR (300.13 MHz, CDCl3). Ethyl 2-decyl-1,5-dimethyl-6,7-dioxa-8-azabicyclo[3.2.1]octane-2-carboxylate, 2a + 2b

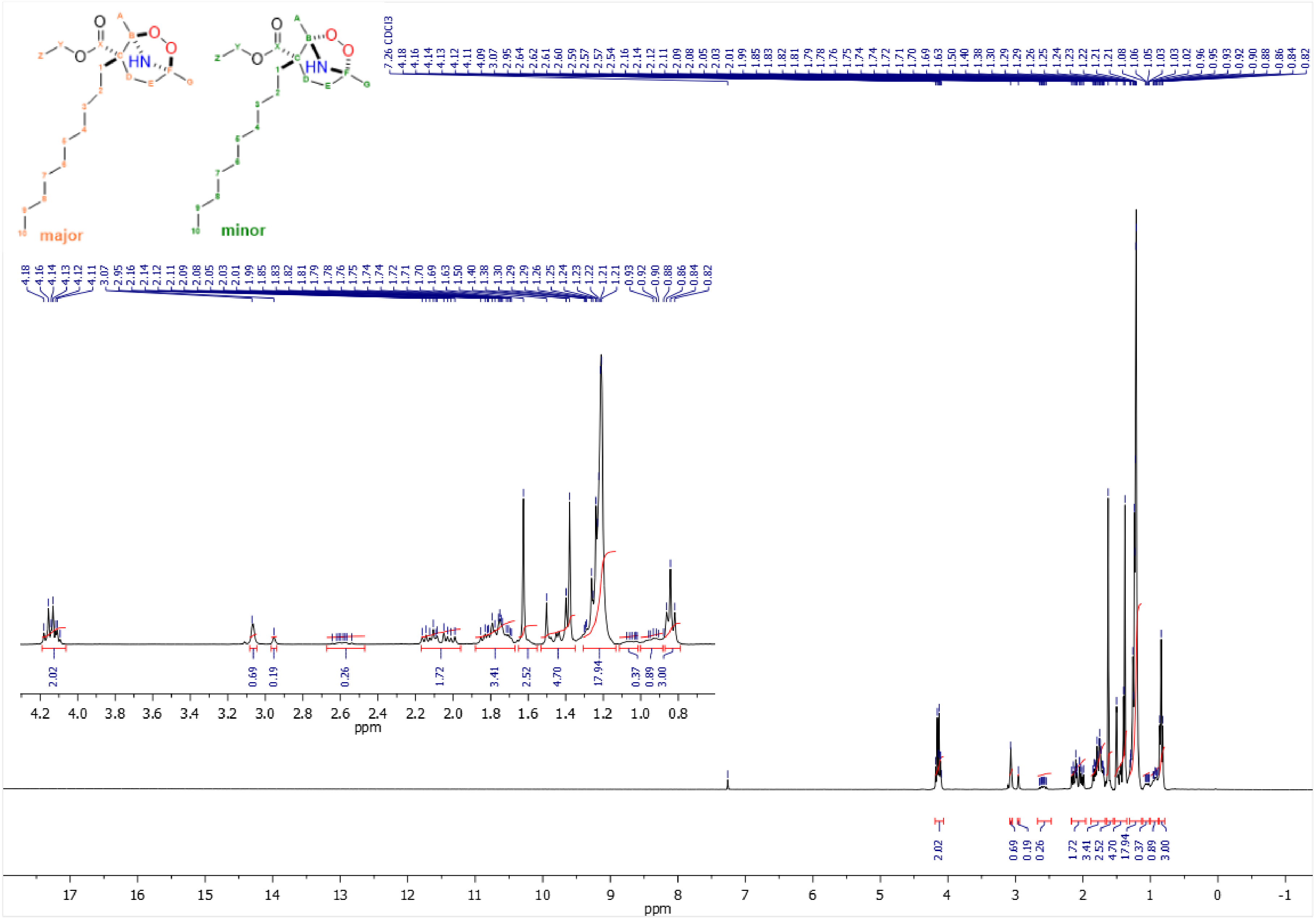

^{1}H NMR (300.13 MHz, $CDCl_3$). Ethyl 2-decyl-1,5-dimethyl-6,7-dioxa-8-azabicyclo[3.2.1]octane-2-carboxylate, 2a + 2b

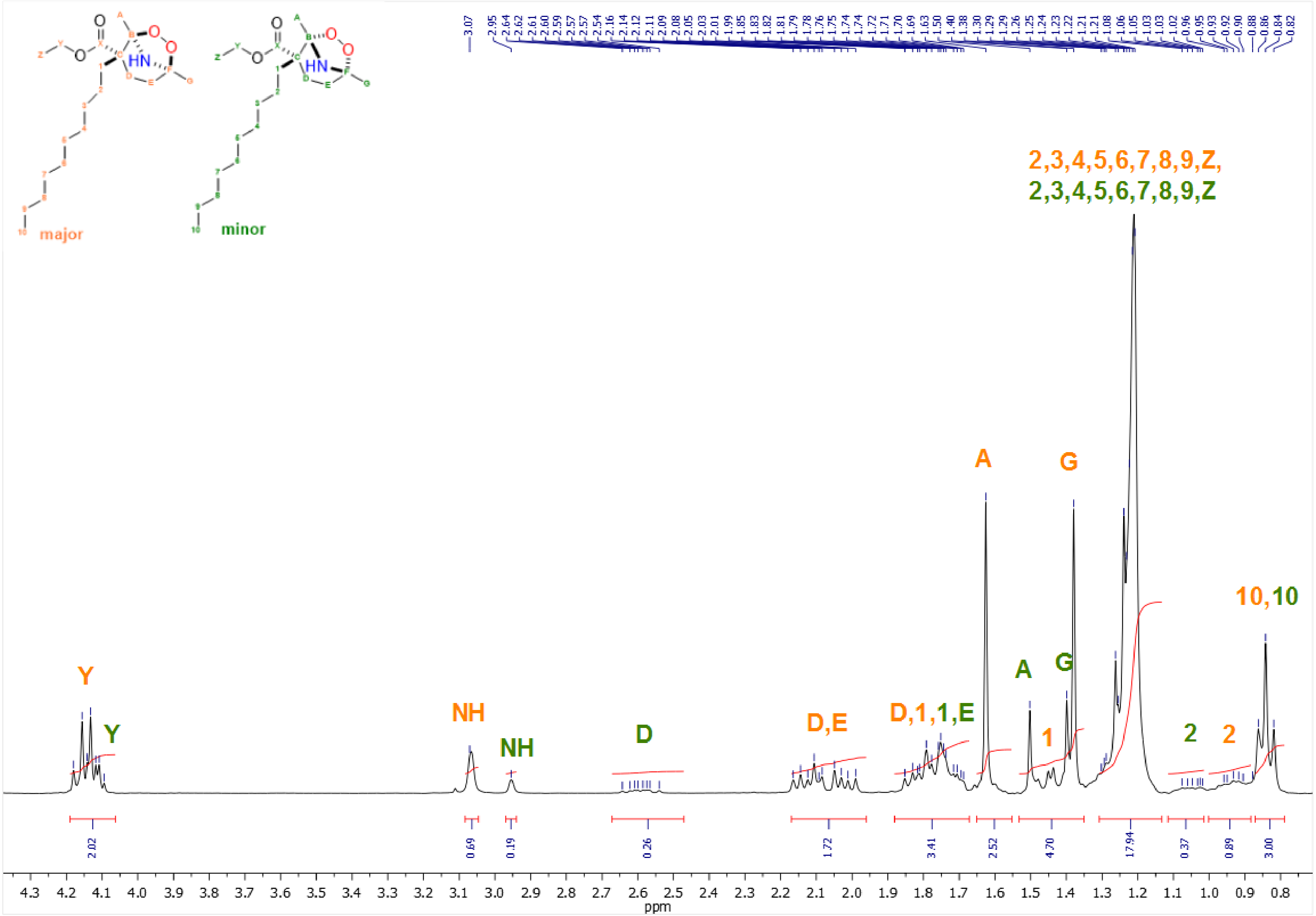

^{13}C NMR (75.48 MHz, CDCl3). Ethyl 2-decyl-1,5-dimethyl-6,7-dioxa-8-azabicyclo[3.2.1]octane-2-carboxylate, 2a + 2b

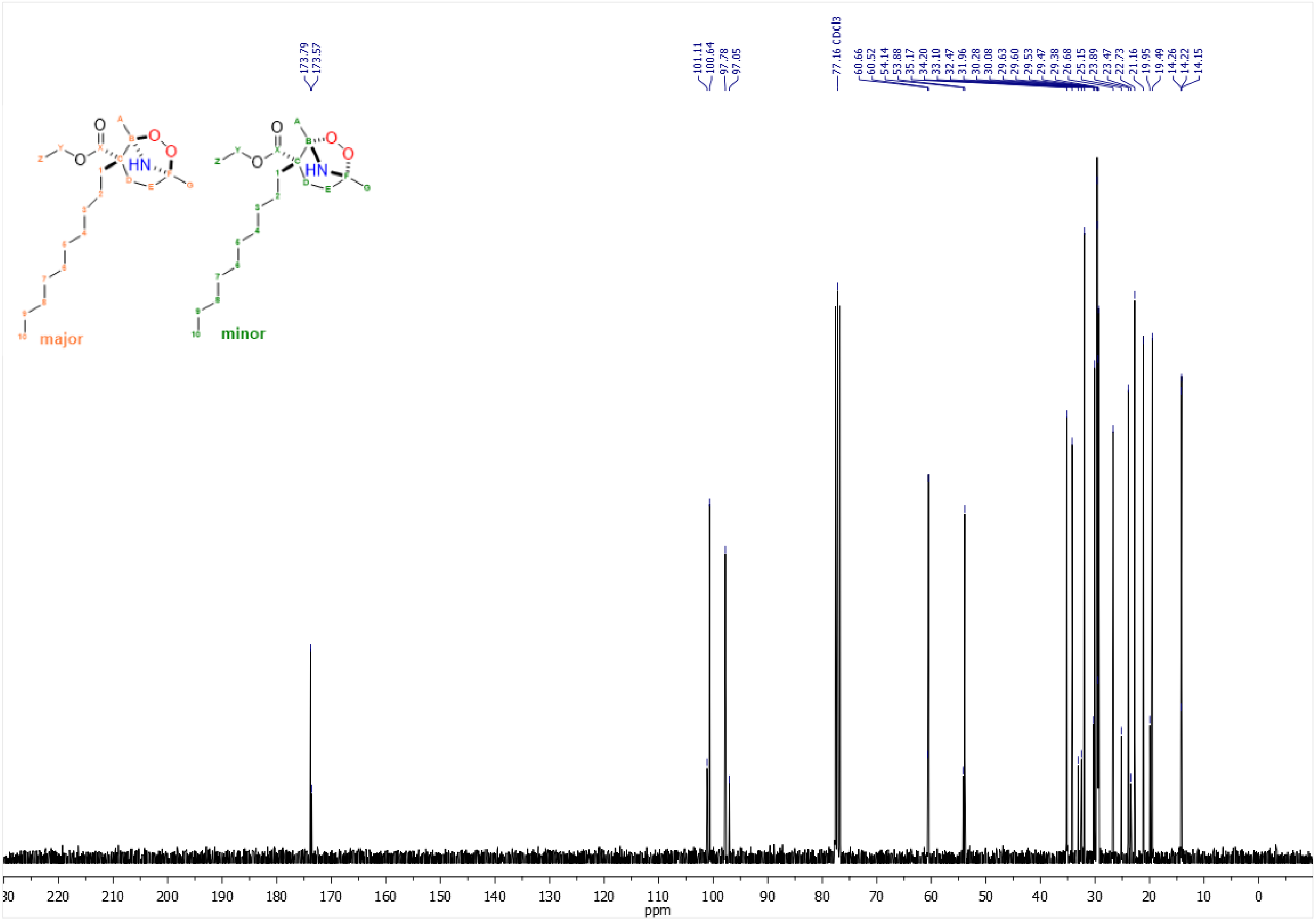

^{13}C NMR (75.48 MHz, CDCl$_3$). Ethyl 2-decyl-1,5-dimethyl-6,7-dioxa-8-azabicyclo[3.2.1]octane-2-carboxylate, 2a + 2b

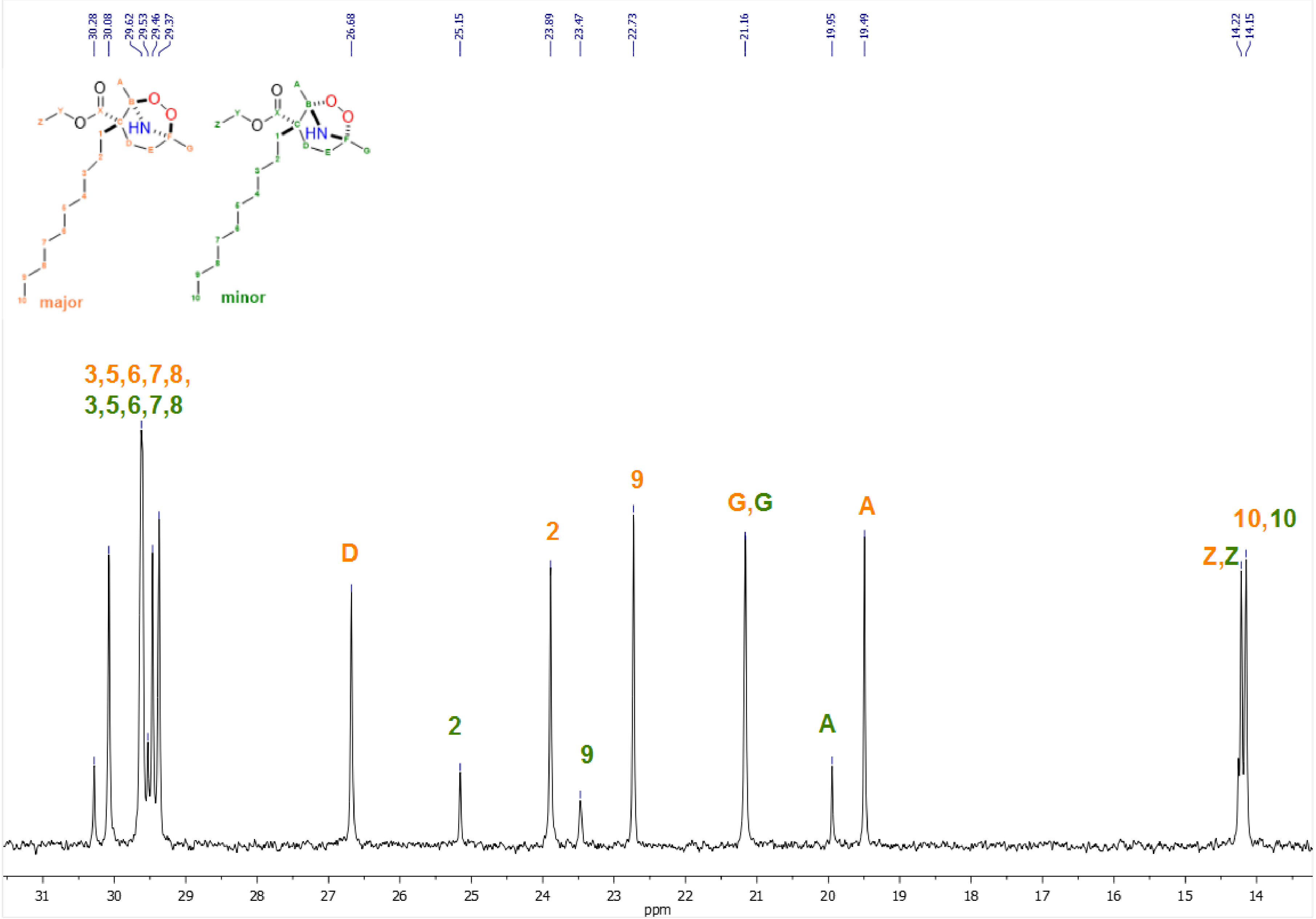

^{13}C NMR (75.48 MHz, $CDCl_3$). Ethyl 2-decyl-1,5-dimethyl-6,7-dioxa-8-azabicyclo[3.2.1]octane-2-carboxylate, 2a + 2b

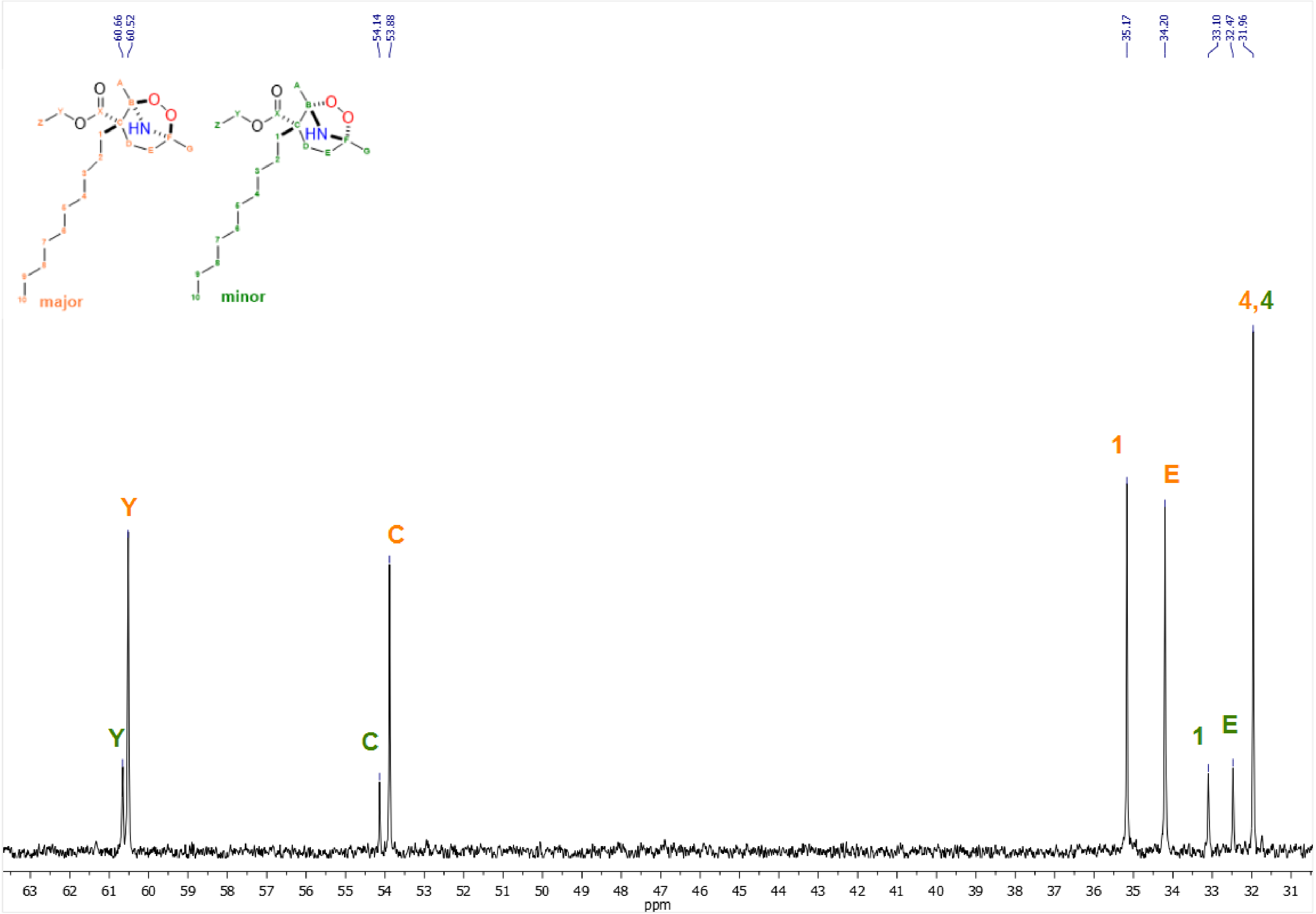

^{13}C NMR (75.48 MHz, CDCl3). Ethyl 2-decyl-1,5-dimethyl-6,7-dioxa-8-azabicyclo[3.2.1]octane-2-carboxylate, 2a + 2b

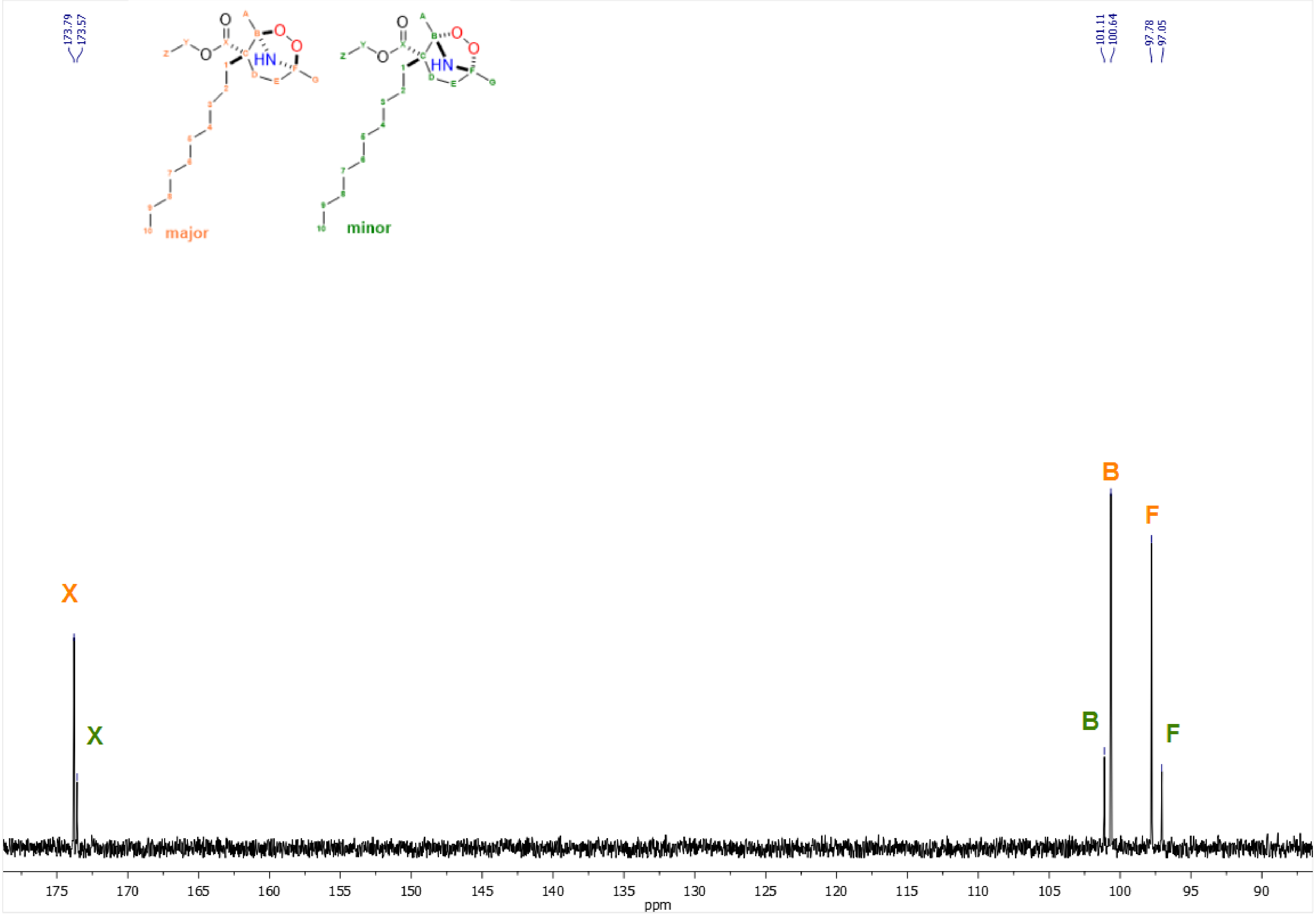

^{1}H NMR (300.13 MHz, $CDCl_3$). Benzyl 2-allyl-1,5-dimethyl-6,7-dioxa-8-azabicyclo[3.2.1]octane-2-carboxylate, 3a + 3b

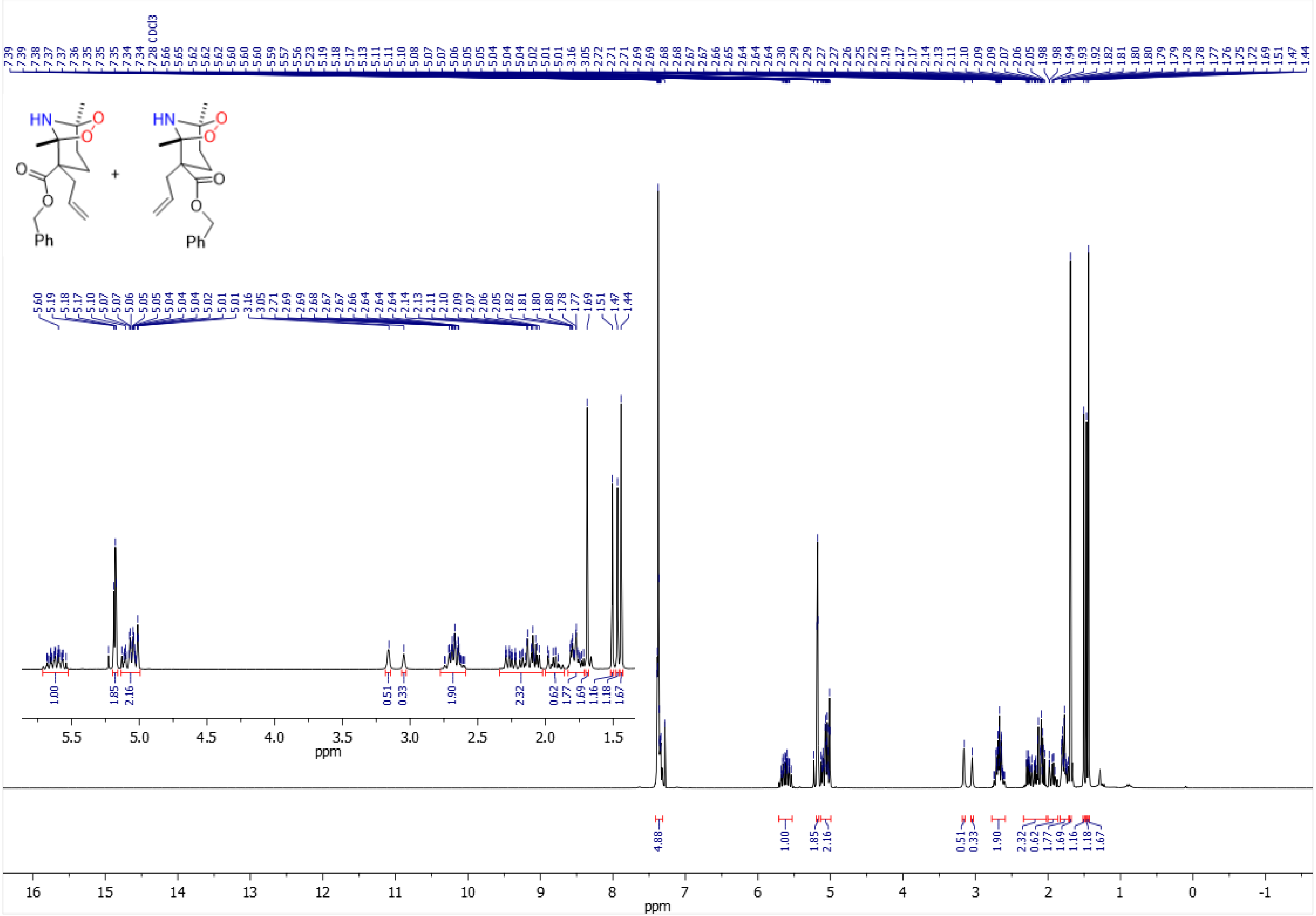

^{13}C NMR (75.48 MHz, CDCl$_3$). Benzyl 2-allyl-1,5-dimethyl-6,7-dioxa-8-azabicyclo[3.2.1]octane-2-carboxylate, 3a + 3b

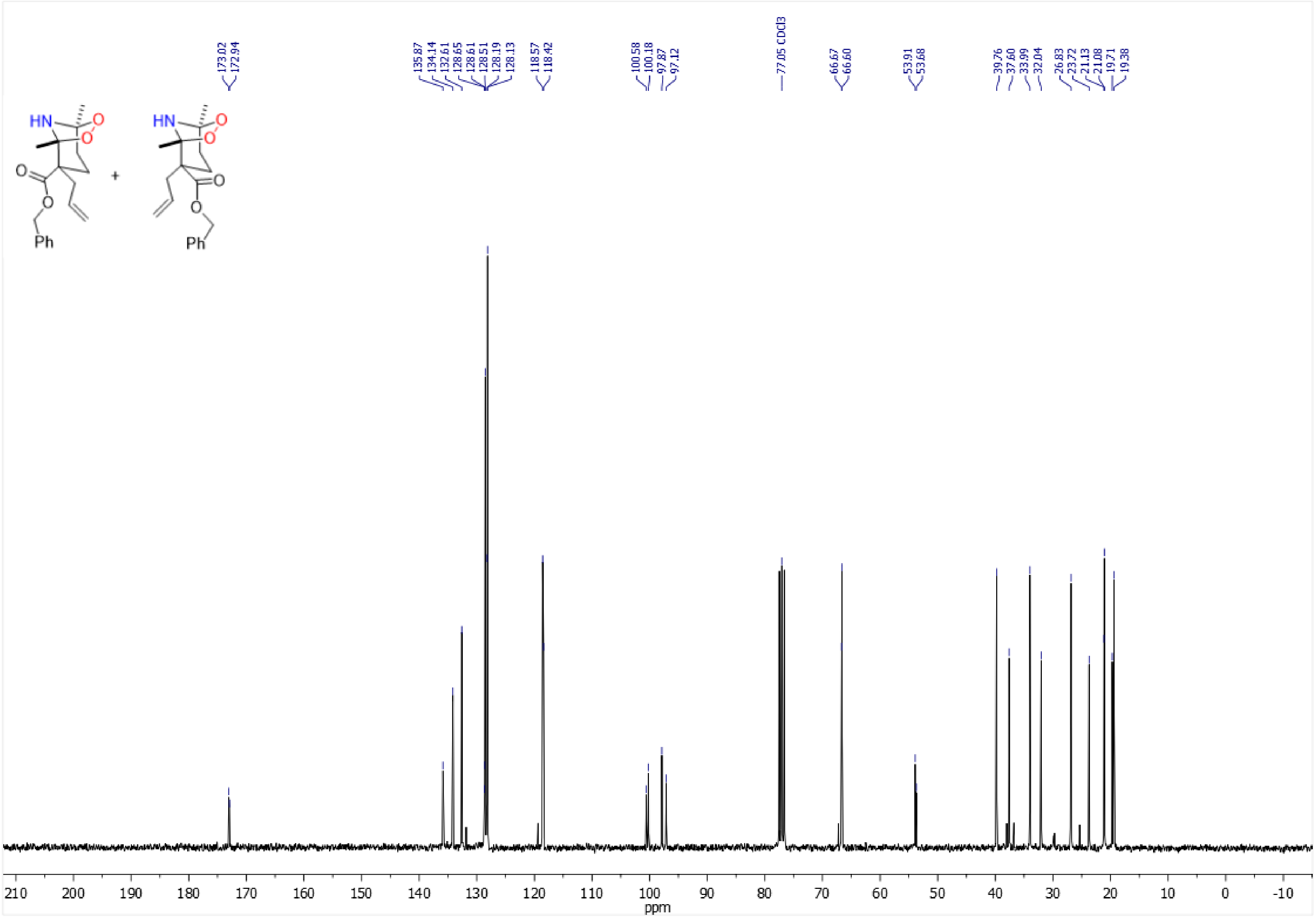

^{1}H NMR (300.13 MHz, $CDCl_3$). Adamantan-1-yl 2-allyl-1,5-dimethyl-6,7-dioxa-8-azabicyclo[3.2.1]octane-2-carboxylate, 4a +4b

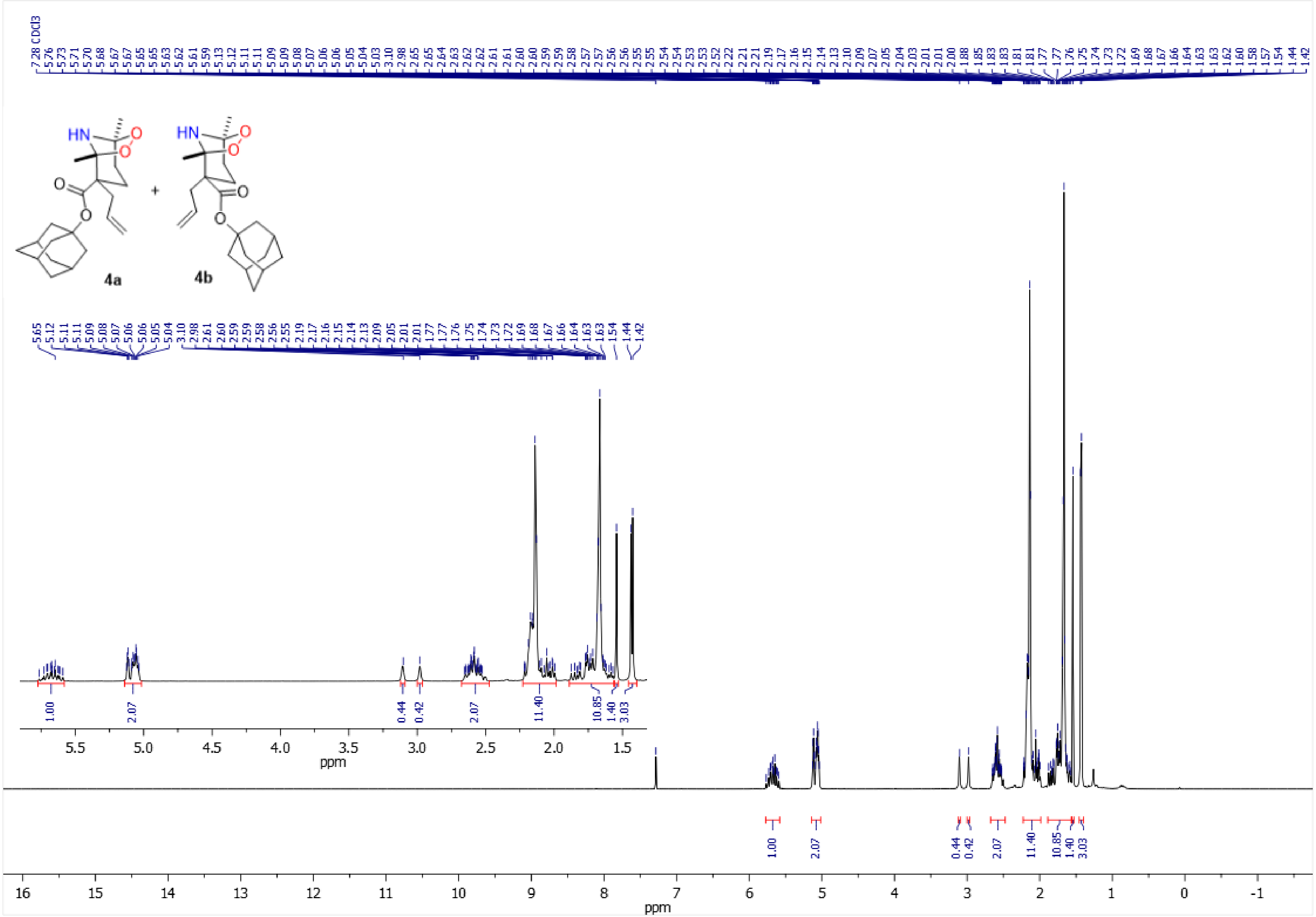

^{13}C NMR (75.48 MHz, $CDCl_3$). Adamantan-1-yl 2-allyl-1,5-dimethyl-6,7-dioxa-8-azabicyclo[3.2.1]octane-2-carboxylate, 4a +4b

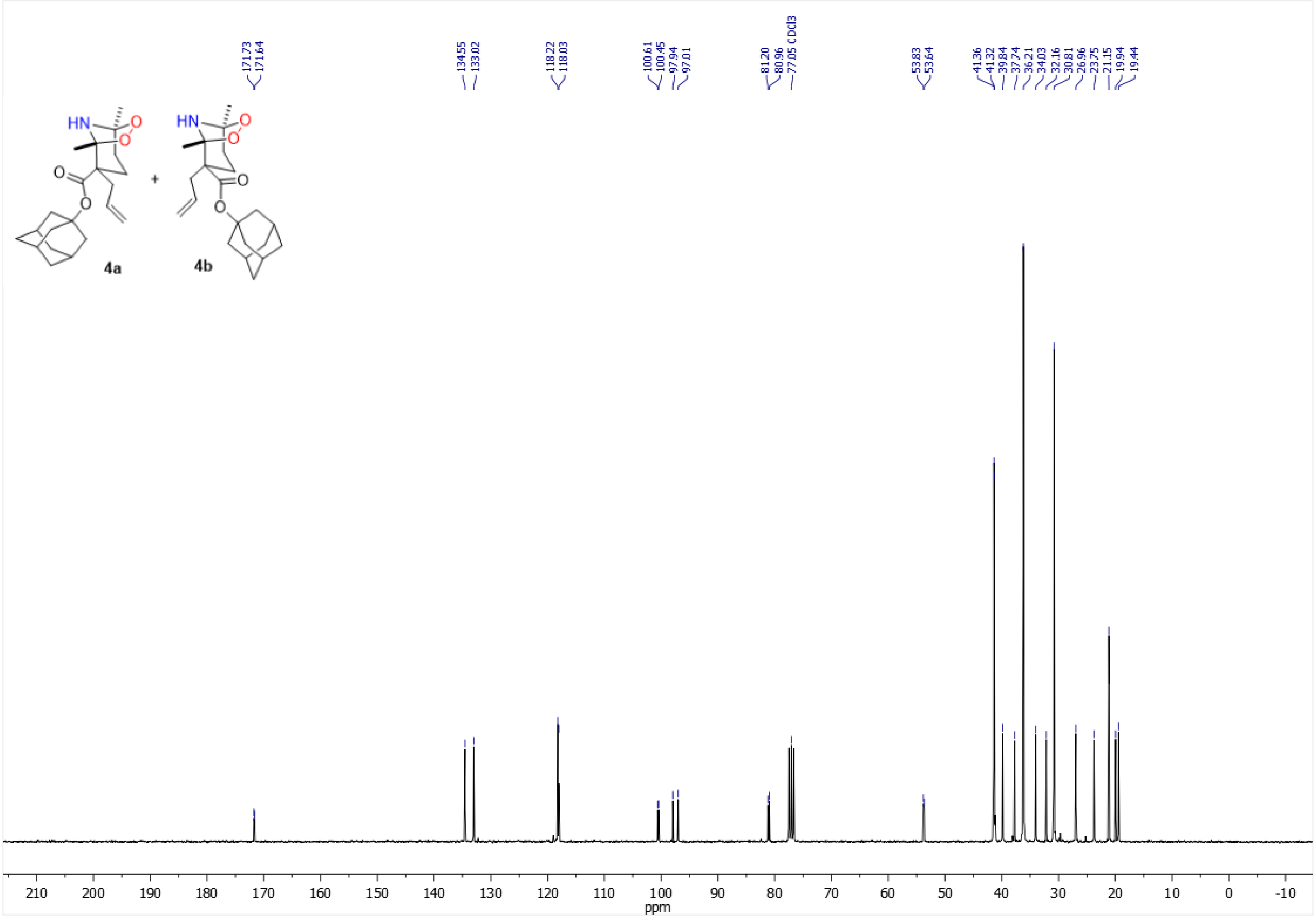

^{1}H NMR (300.13 MHz, CDCl3). Allyl 2-allyl-1,5-dimethyl-6,7-dioxa-8-azabicyclo[3.2.1]octane-2-carboxylate, 5a + 5b

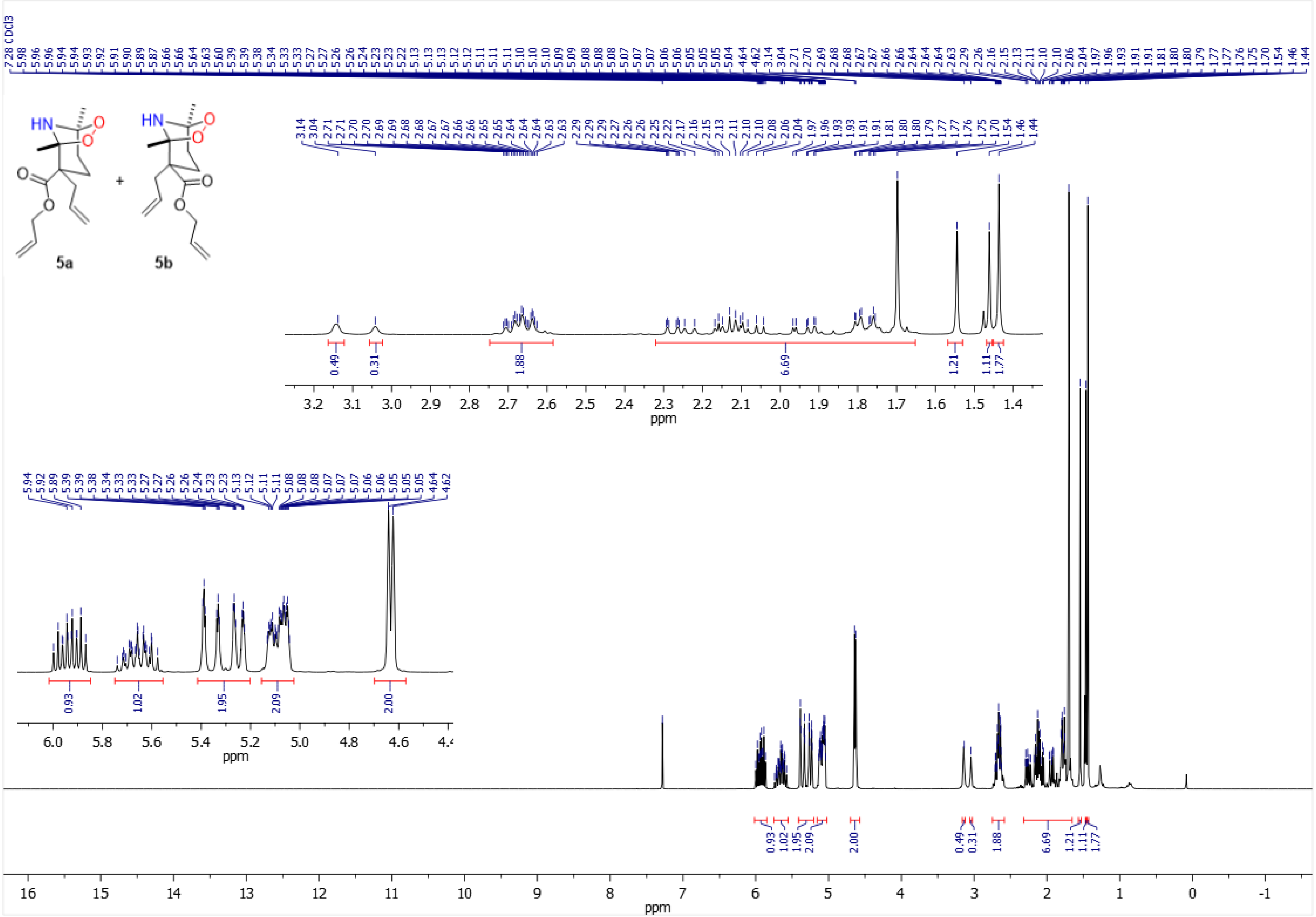

^{13}C NMR (75.48 MHz, CDCl3). Allyl 2-allyl-1,5-dimethyl-6,7-dioxa-8-azabicyclo[3.2.1]octane-2-carboxylate, 5a + 5b

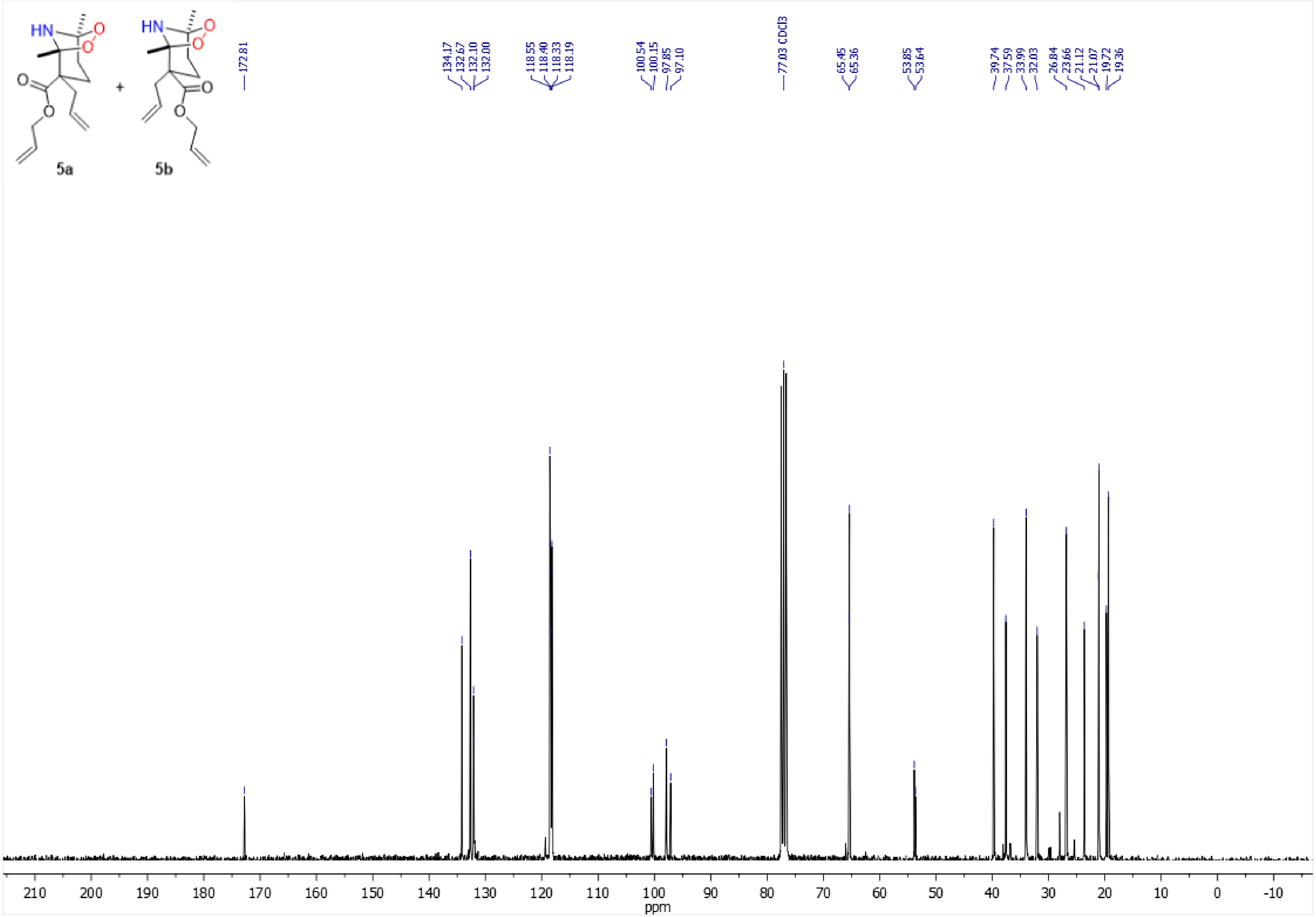

^{1}H NMR (300.13 MHz, $CDCl_3$). (1R,5S)-1,5-dimethyl-3-(4-nitrophenyl)-6,7-dioxa-8-azabicyclo[3.2.1]octane, 6

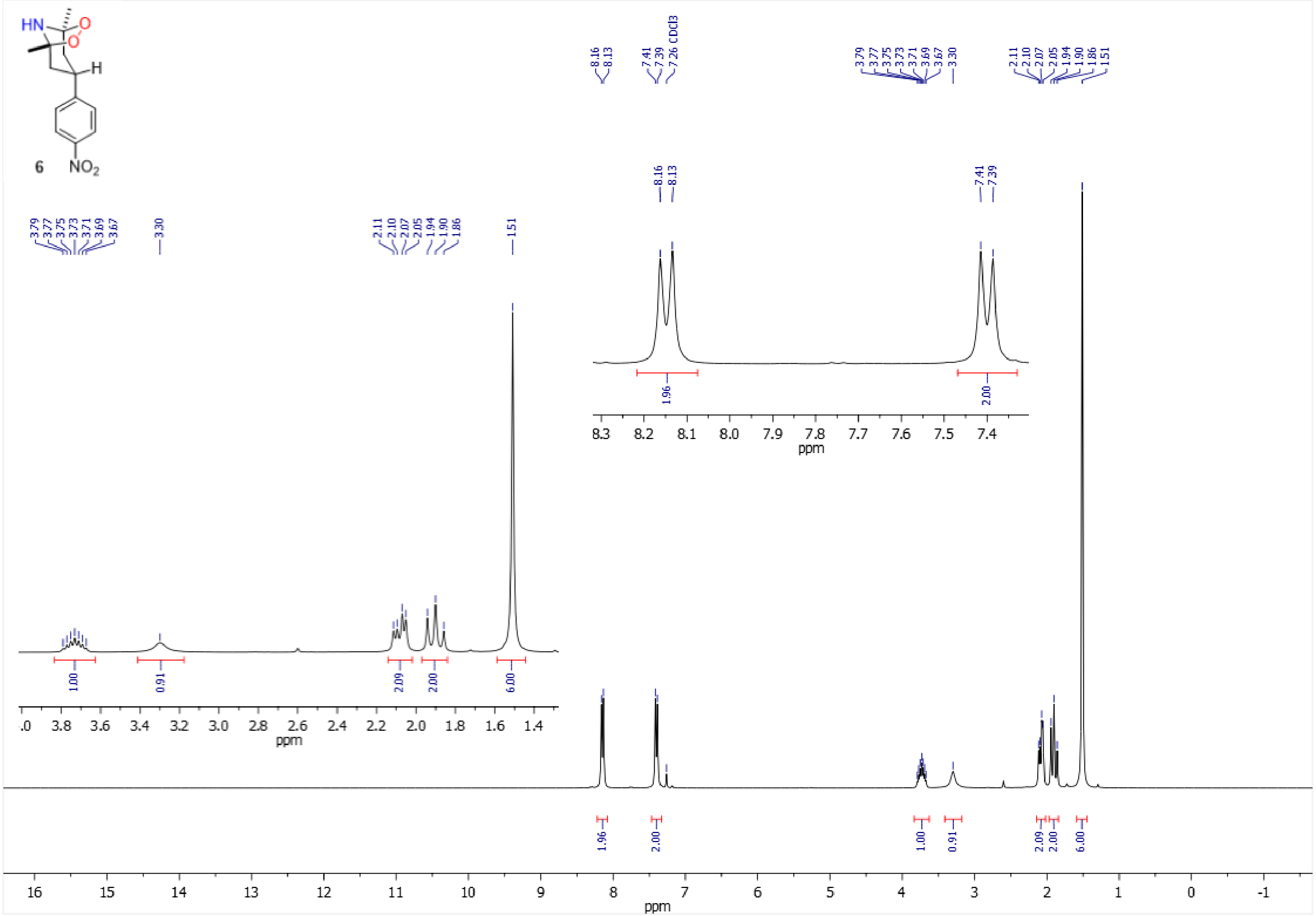

^{13}C NMR (75.48 MHz, $CDCl_3$). (1R,5S)-1,5-dimethyl-3-(4-nitrophenyl)-6,7-dioxa-8-azabicyclo[3.2.1]octane, 6

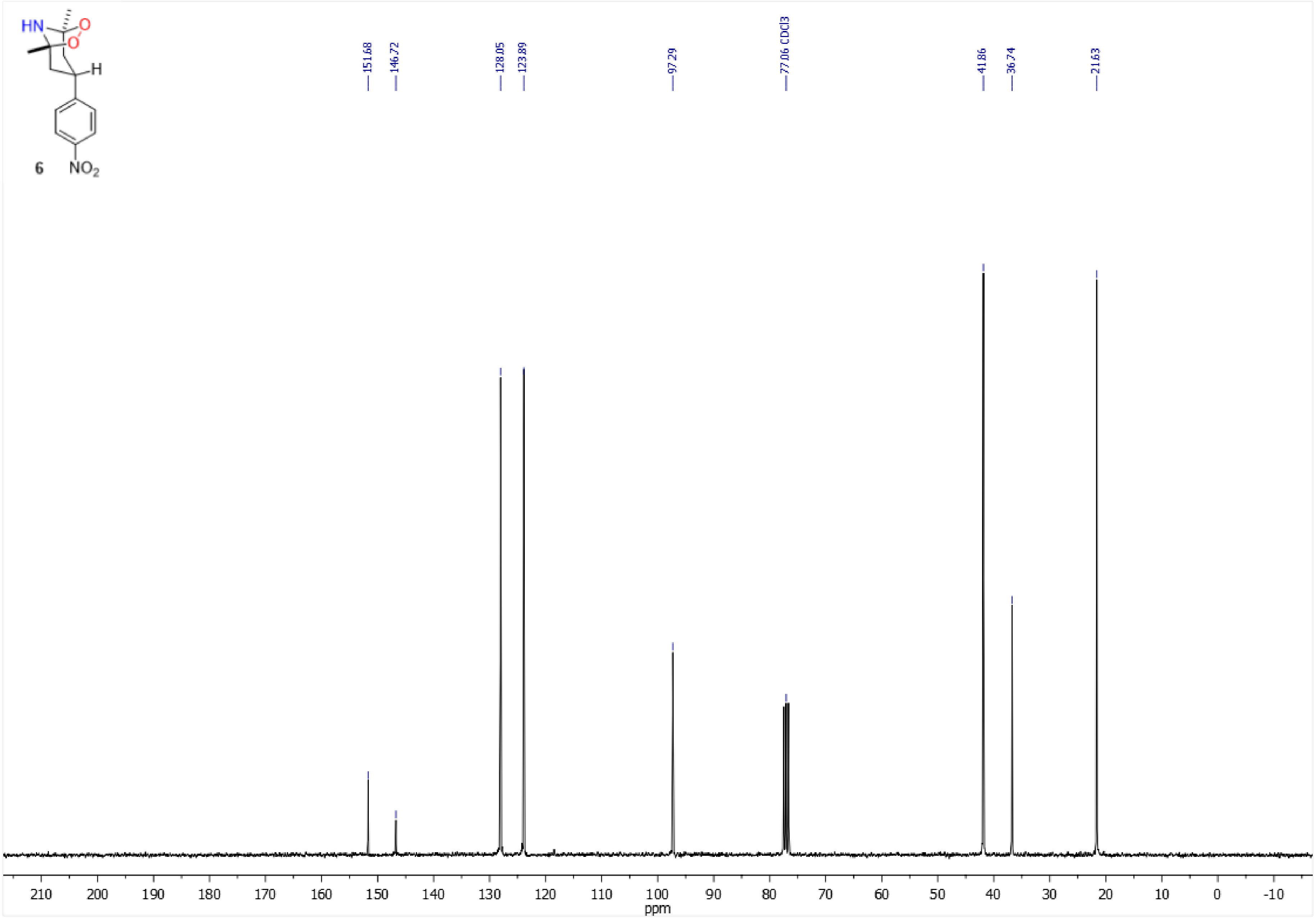

^{1}H NMR (300.13 MHz, $CDCl_3$). (1R,5S)-3-(4-chlorophenyl)-1,5-dimethyl-6,7-dioxa-8-azabicyclo[3.2.1]octane, 7

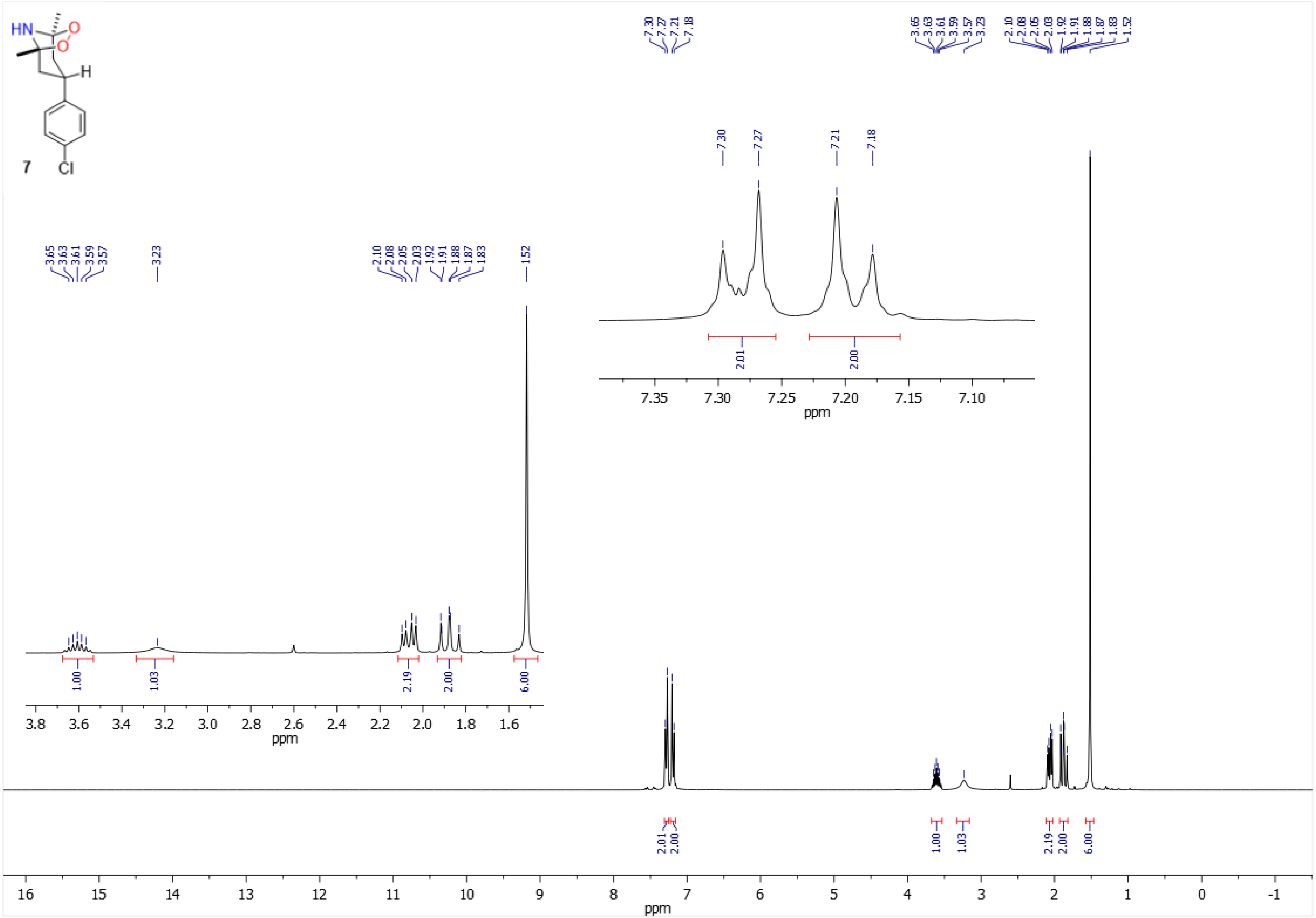

^{13}C NMR (75.48 MHz, $CDCl_3$). (1R,5S)-3-(4-chlorophenyl)-1,5-dimethyl-6,7-dioxa-8-azabicyclo[3.2.1]octane, 7

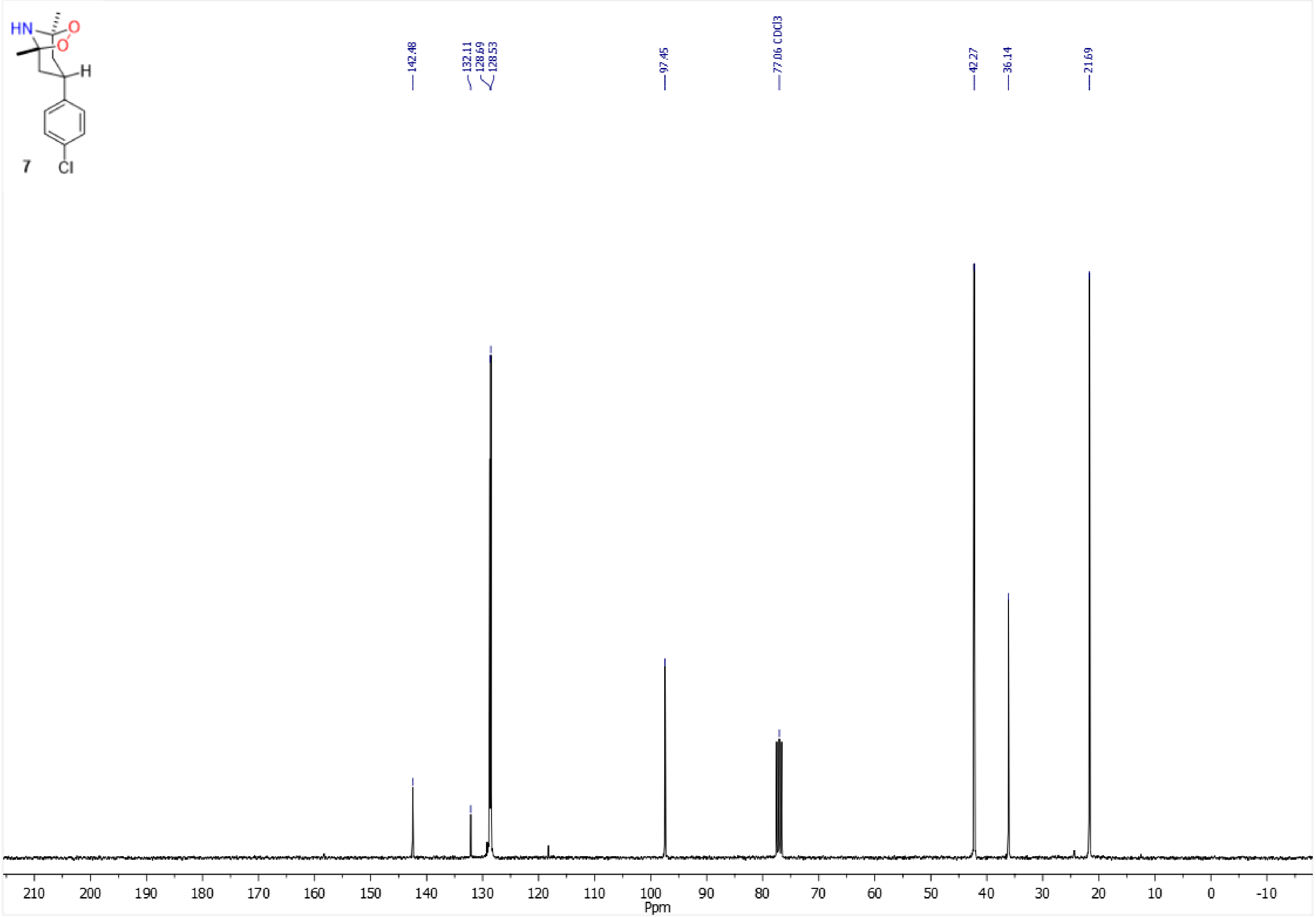

^{1}H NMR (300.13 MHz, CDCl3). 1-ethoxy-1-oxopropan-2-yl -2-allyl-1,5-dimethyl-6,7-dioxa-8-azabicyclo[3.2.1]octane-2-carboxylate, 8a', 8a'', 8b', 8b"

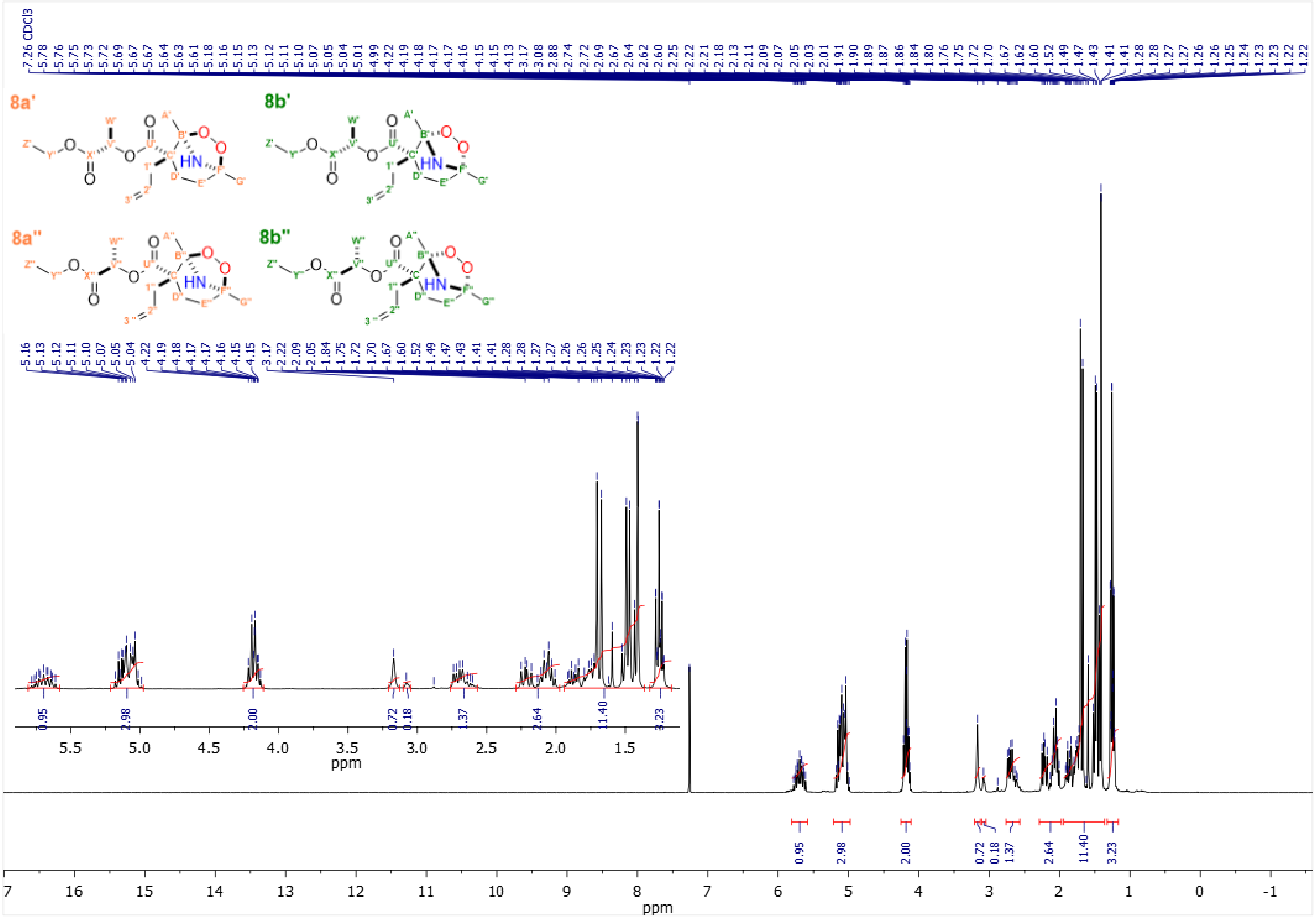

^{1}H NMR (300.13 MHz, CDCl3). 1-ethoxy-1-oxopropan-2-yl -2-allyl-1,5-dimethyl-6,7-dioxa-8-azabicyclo[3.2.1]octane-2-carboxylate, 8a’, 8a’’, 8b’, 8b”

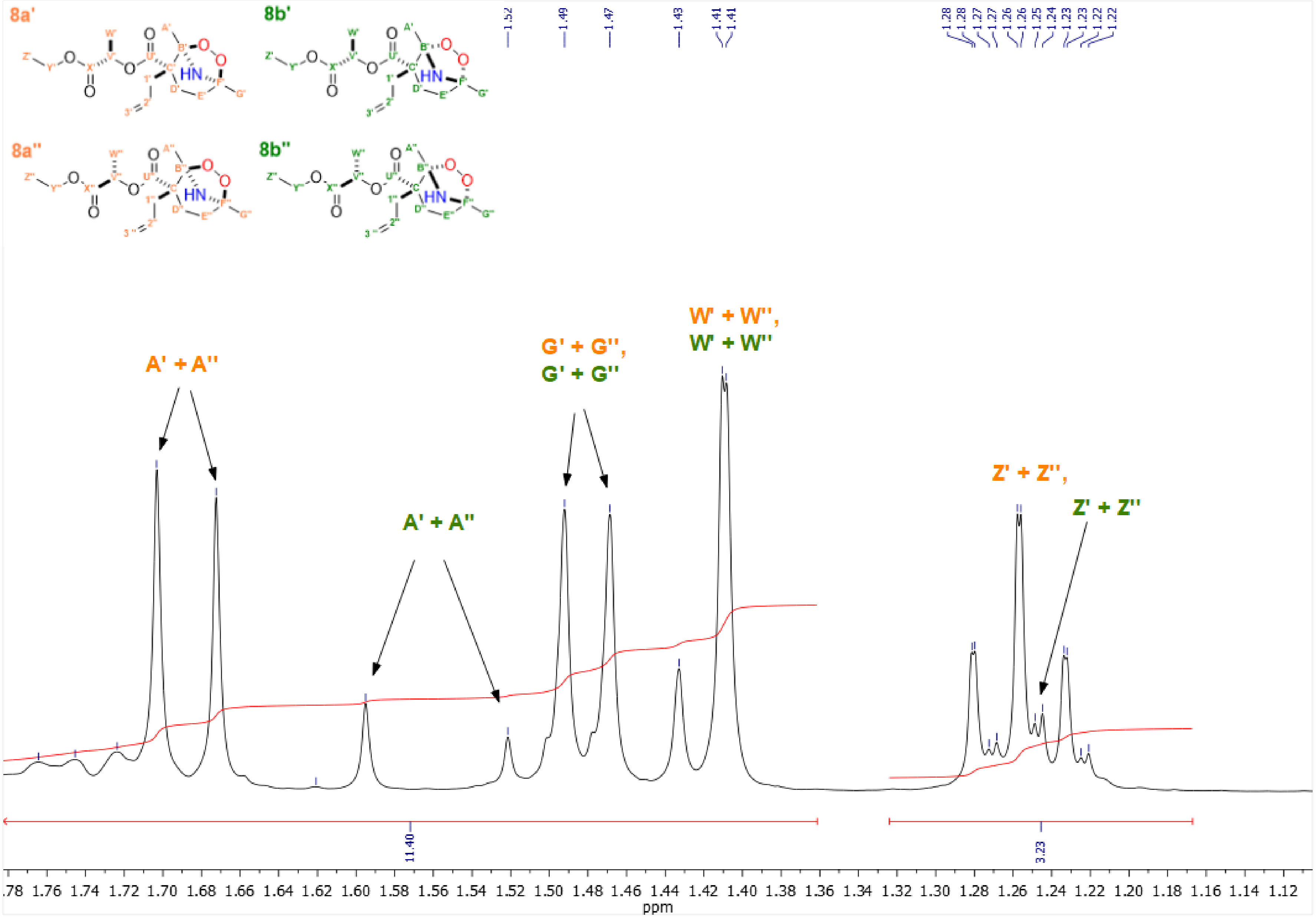

^{1}H NMR (300.13 MHz, CDCl3). 1-ethoxy-1-oxopropan-2-yl -2-allyl-1,5-dimethyl-6,7-dioxa-8-azabicyclo[3.2.1]octane-2-carboxylate, 8a', 8a'', 8b', 8b''

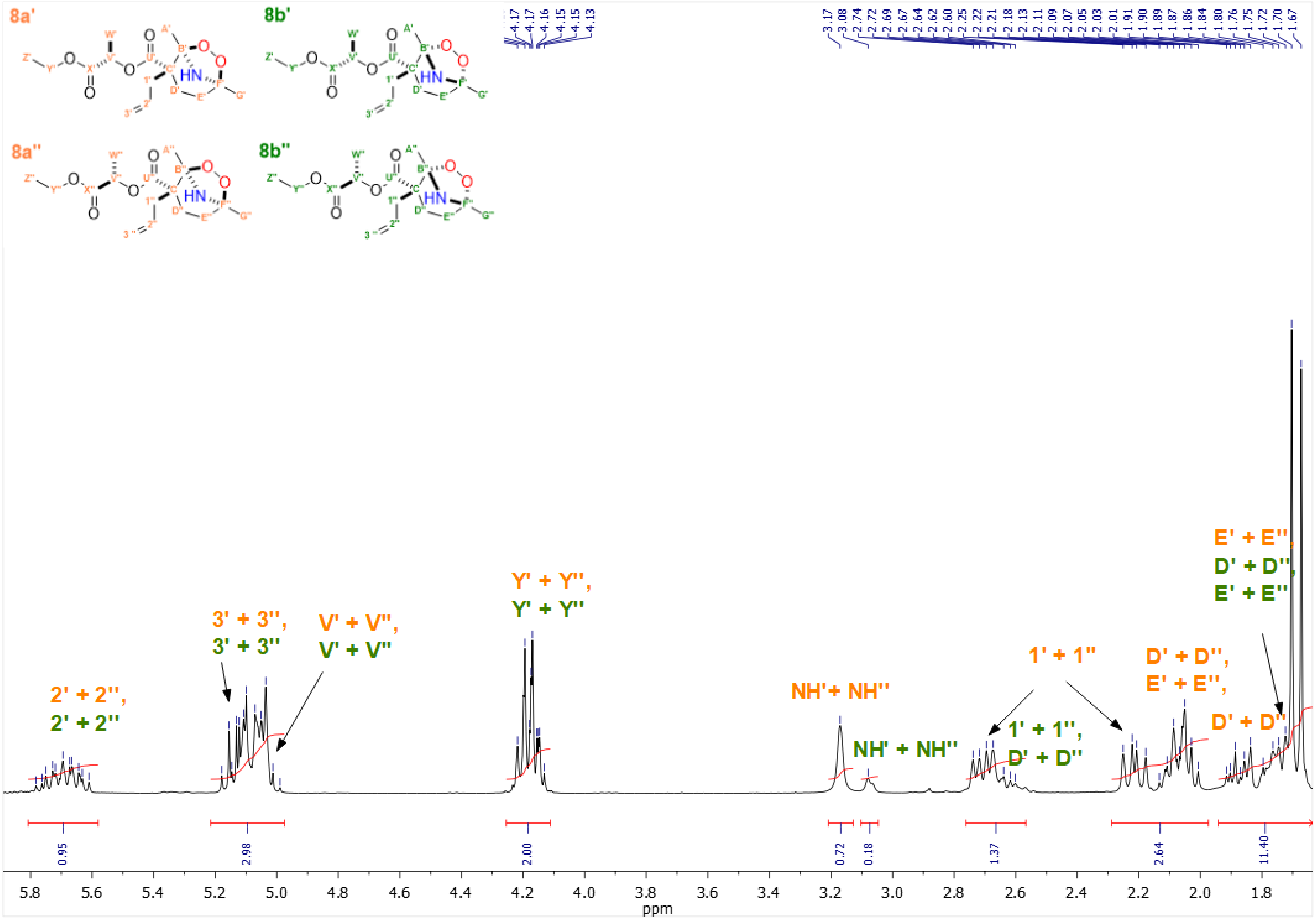

^{13}C NMR (75.48 MHz, $CDCl_3$). 1-ethoxy-1-oxopropan-2-yl -2-allyl-1,5-dimethyl-6,7-dioxa-8-azabicyclo[3.2.1]octane-2-carboxylate, 8a’, 8a’’, 8b’, 8b”

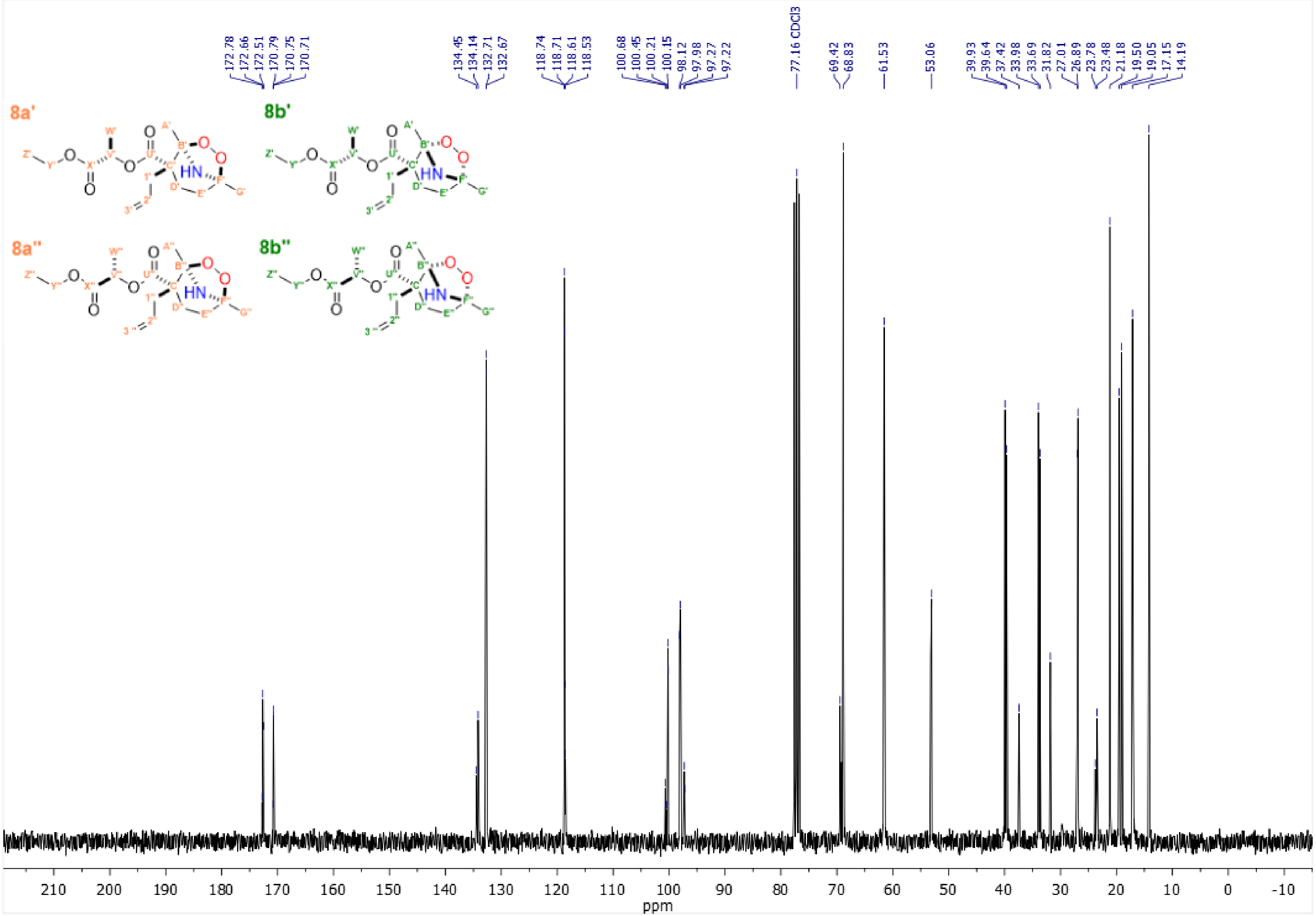

^{13}C NMR (75.48 MHz, CDCl3). 1-ethoxy-1-oxopropan-2-yl -2-allyl-1,5-dimethyl-6,7-dioxa-8-azabicyclo[3.2.1]octane-2-carboxylate, 8a’, 8a’’, 8b’, 8b”

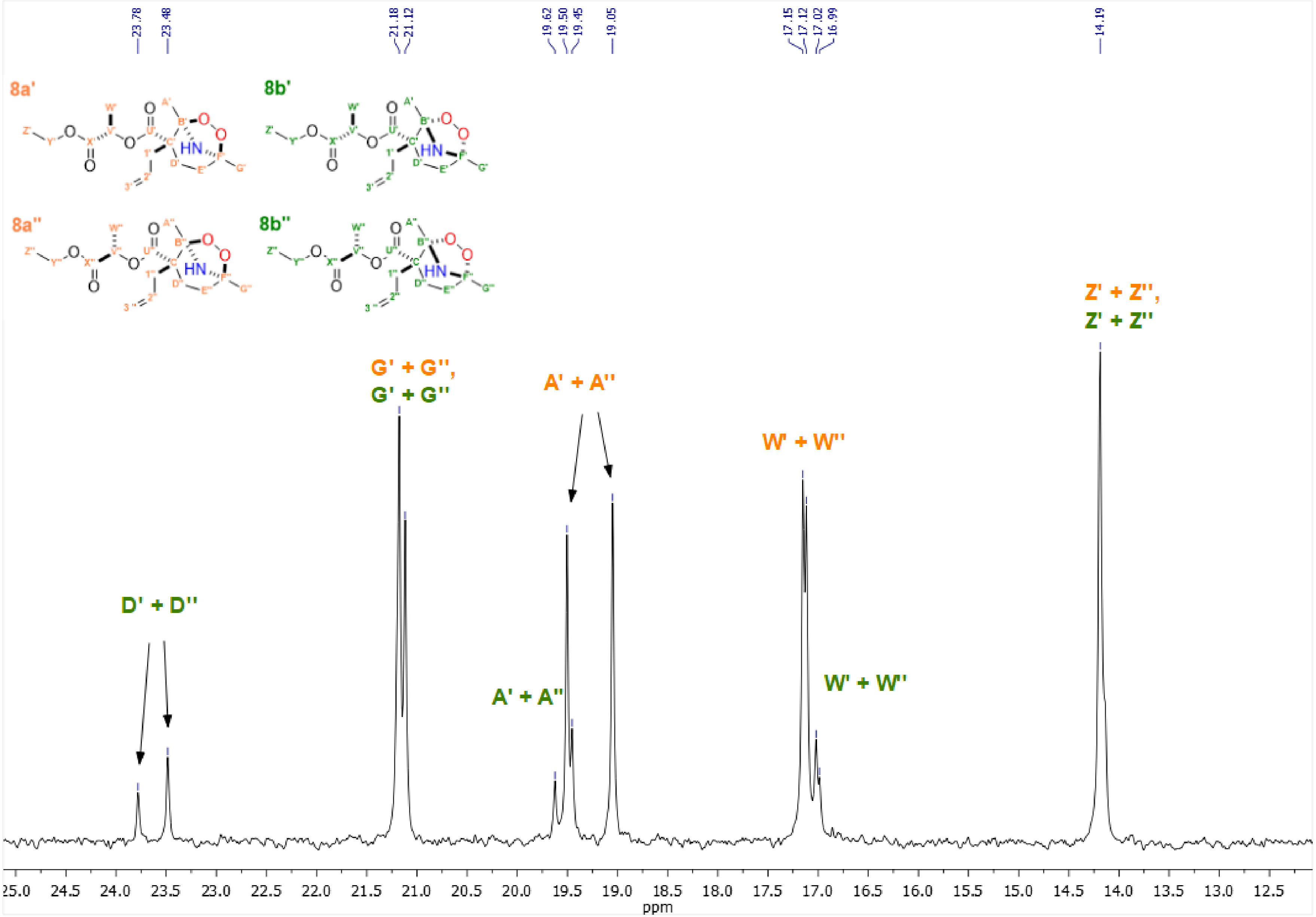

^{13}C NMR (75.48 MHz, $CDCl_3$). 1-ethoxy-1-oxopropan-2-yl -2-allyl-1,5-dimethyl-6,7-dioxa-8-azabicyclo[3.2.1]octane-2-carboxylate, 8a', 8a'', 8b', 8b''

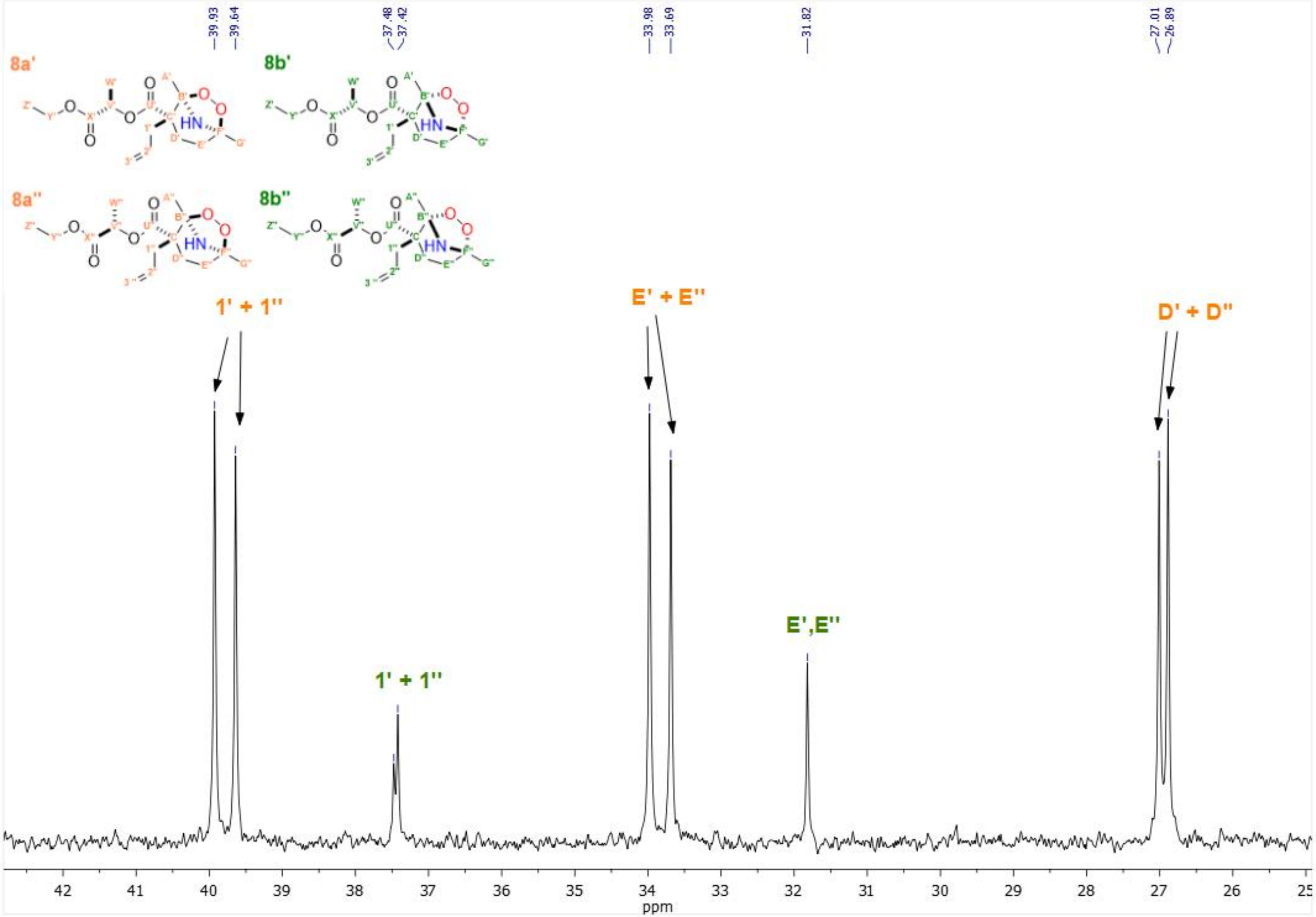

^{13}C NMR (75.48 MHz, CDCl$_3$). 1-ethoxy-1-oxopropan-2-yl -2-allyl-1,5-dimethyl-6,7-dioxa-8-azabicyclo[3.2.1]octane-2-carboxylate, 8a', 8a'', 8b', 8b''

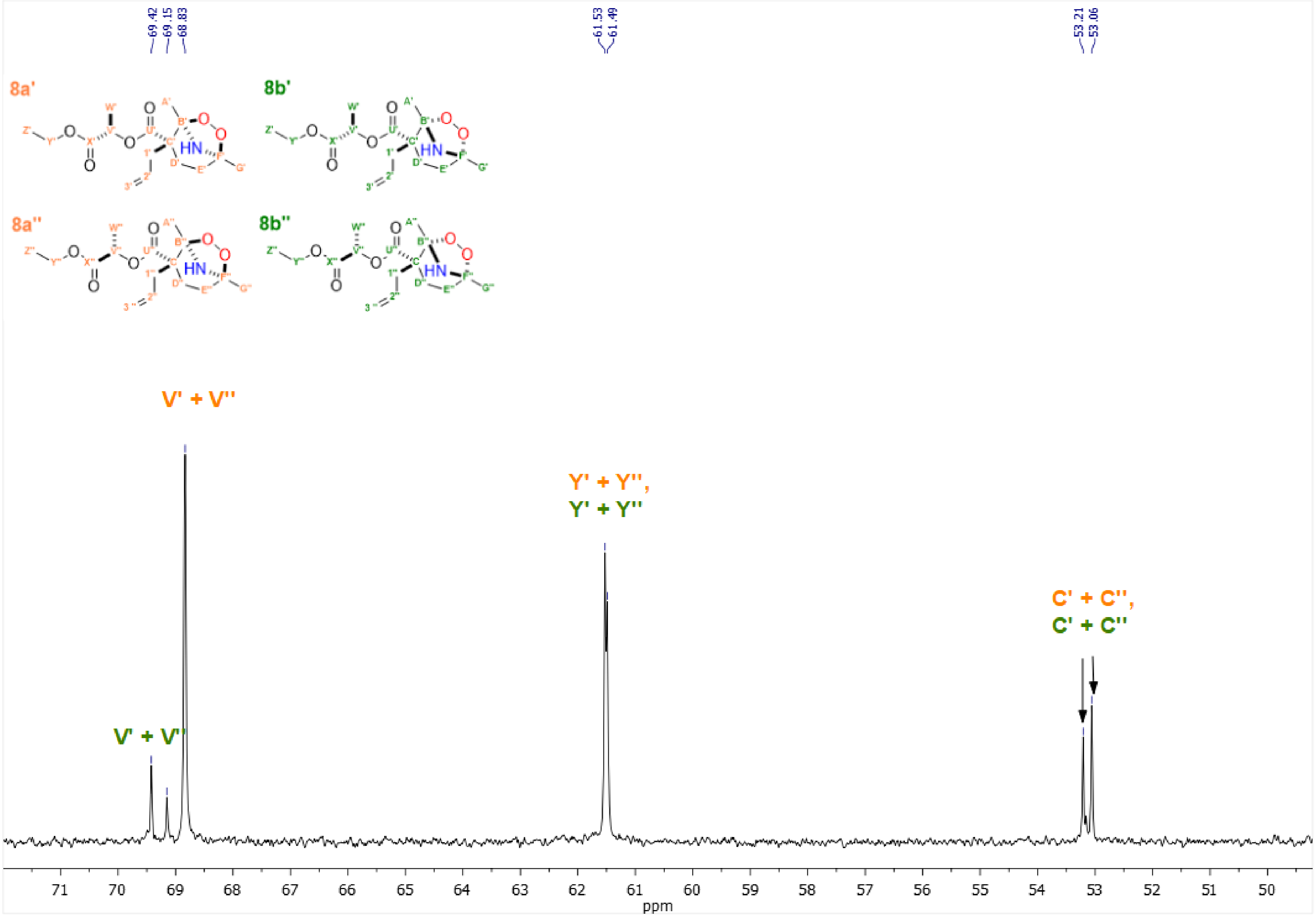

^{13}C NMR (75.48 MHz, $CDCl_3$). 1-ethoxy-1-oxopropan-2-yl -2-allyl-1,5-dimethyl-6,7-dioxa-8-azabicyclo[3.2.1]octane-2-carboxylate, 8a’, 8a’’, 8b’, 8b”

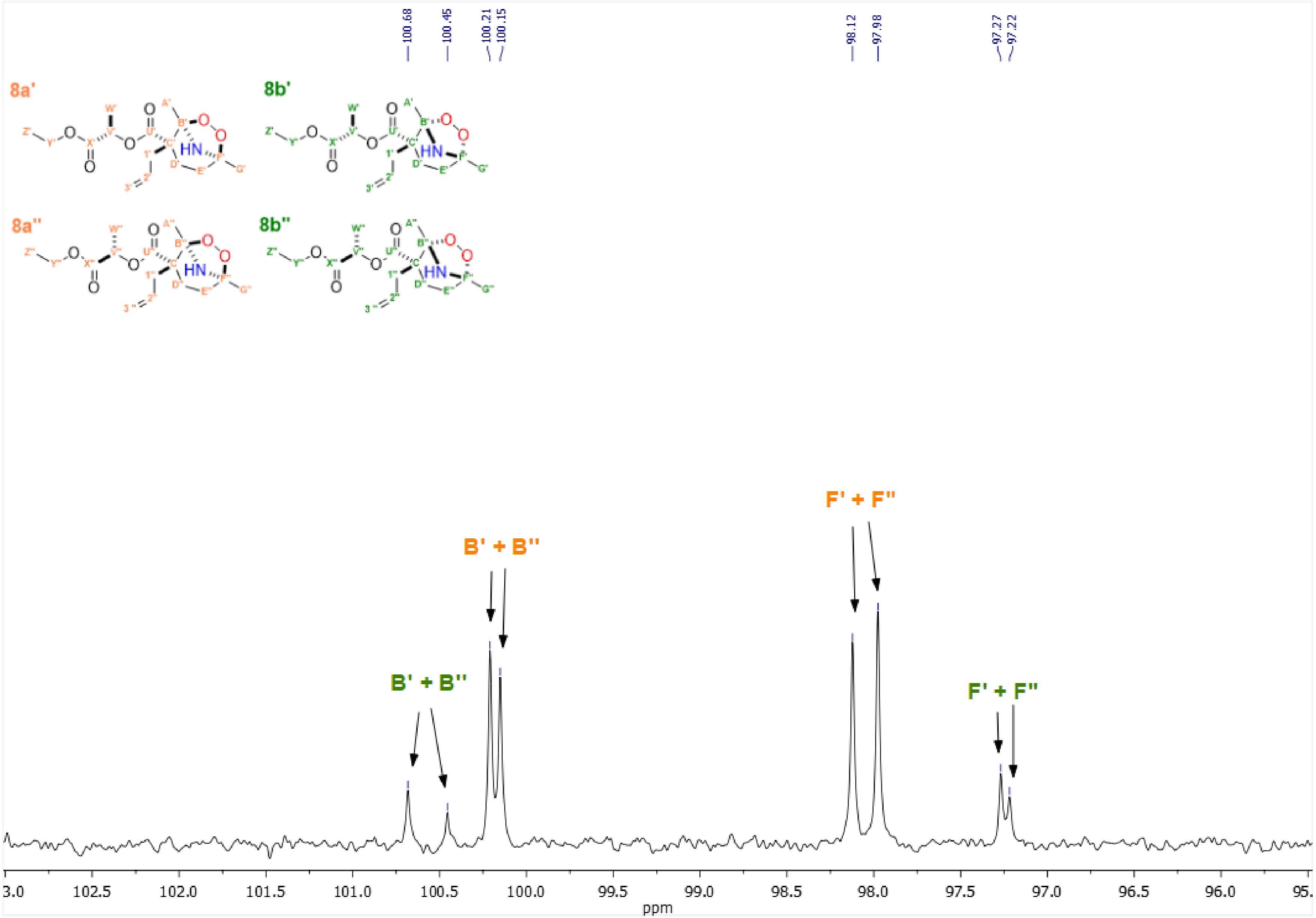

^{13}C NMR (75.48 MHz, CDCl$_3$). 1-ethoxy-1-oxopropan-2-yl -2-allyl-1,5-dimethyl-6,7-dioxa-8-azabicyclo[3.2.1]octane-2-carboxylate, 8a', 8a'', 8b', 8b''

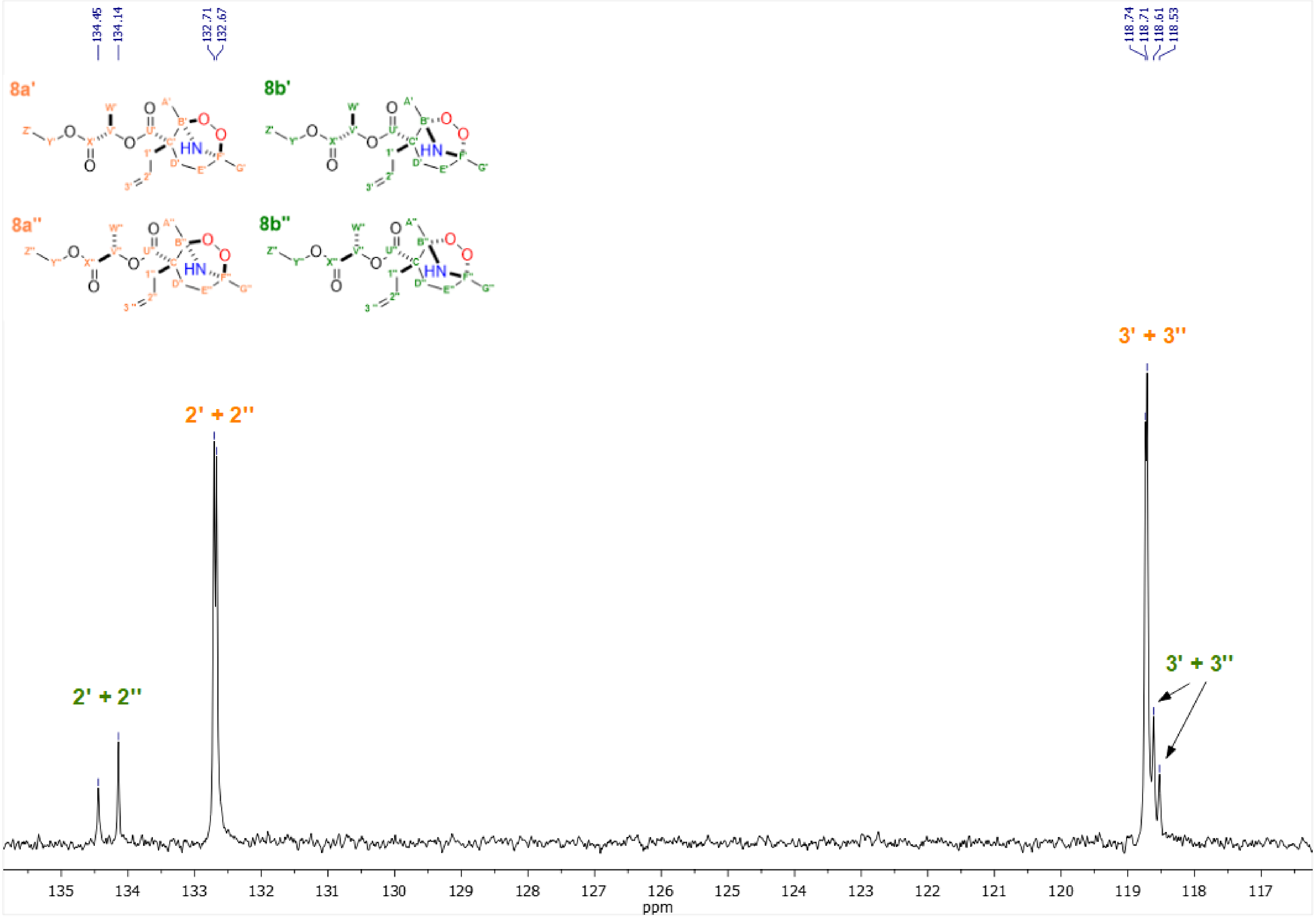

^{13}C NMR (75.48 MHz, CDCl$_3$). 1-ethoxy-1-oxopropan-2-yl -2-allyl-1,5-dimethyl-6,7-dioxa-8-azabicyclo[3.2.1]octane-2-carboxylate, 8a', 8a'', 8b', 8b''

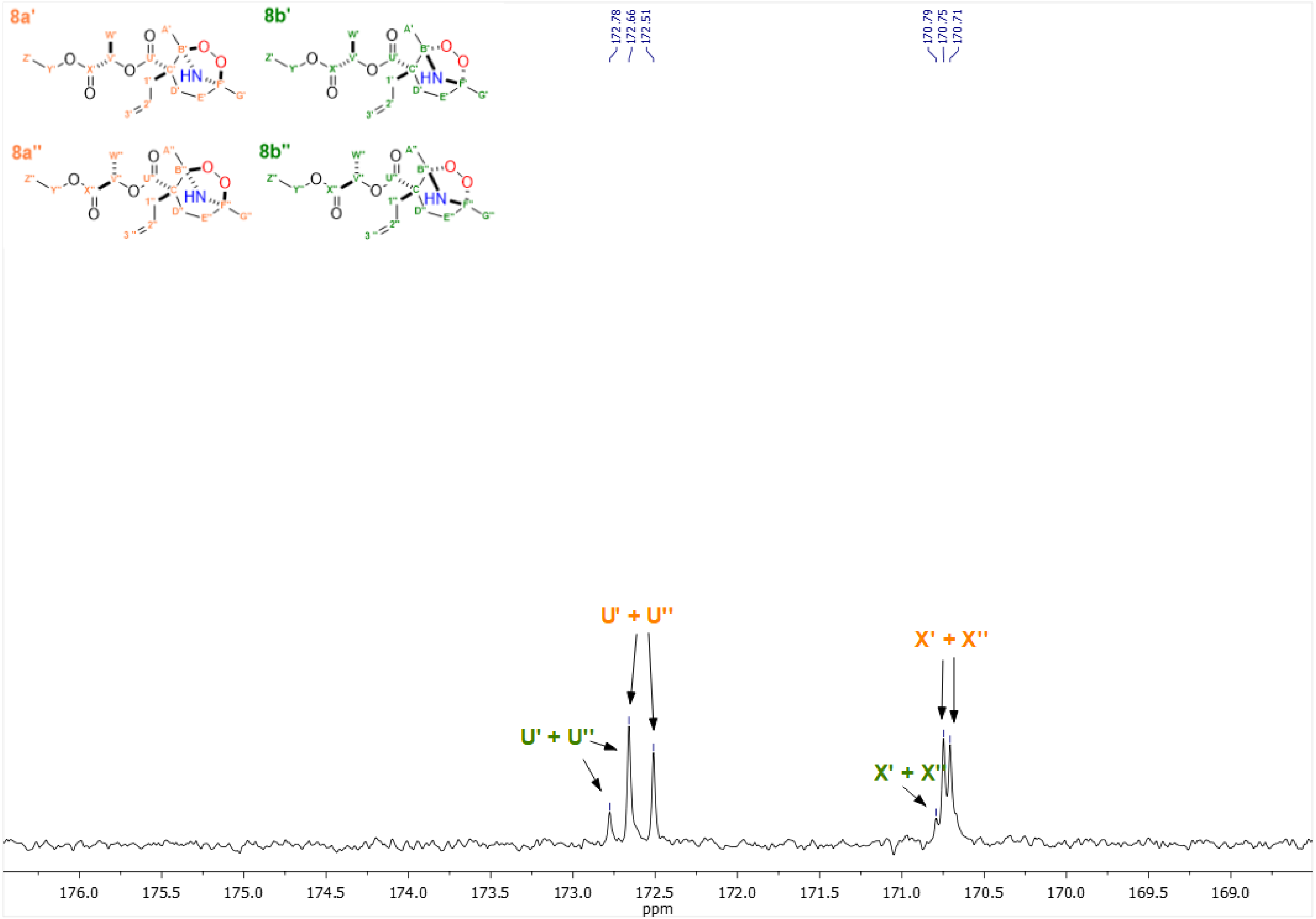

^{15}N (40.56 MHz, $CDCl_3$). 1-ethoxy-1-oxopropan-2-yl -2-allyl-1,5-dimethyl-6,7-dioxa-8-azabicyclo[3.2.1]octane-2-carboxylate, 8a', 8a'', 8b', 8b''

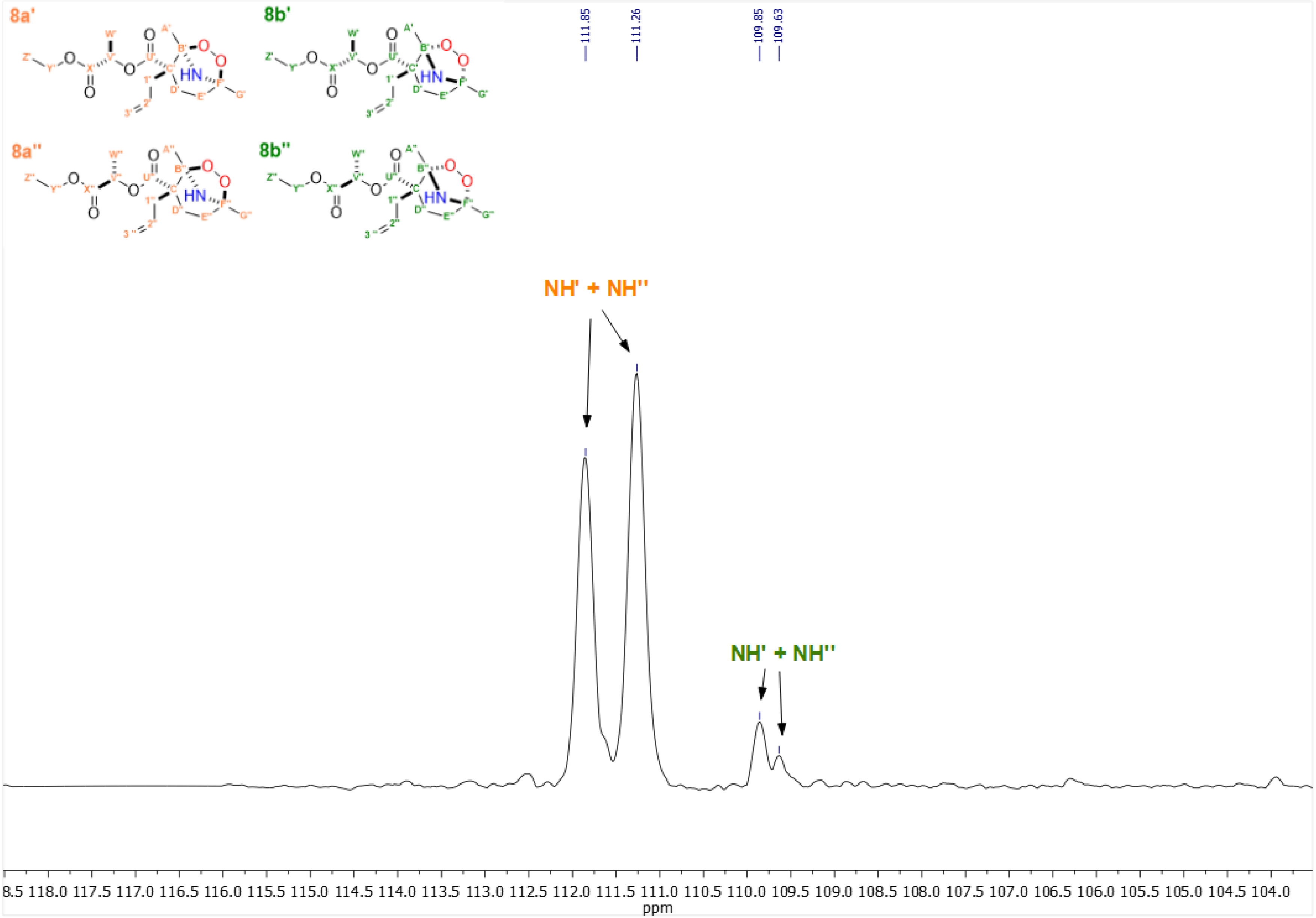

^{13}C NMR (75.48 MHz, CDCl$_3$). 1-ethoxy-1-oxopropan-2-yl -2-allyl-1,5-dimethyl-6,7-dioxa-8-azabicyclo[3.2.1]octane-2-carboxylate, 8a', 8a'', 8b', 8b''

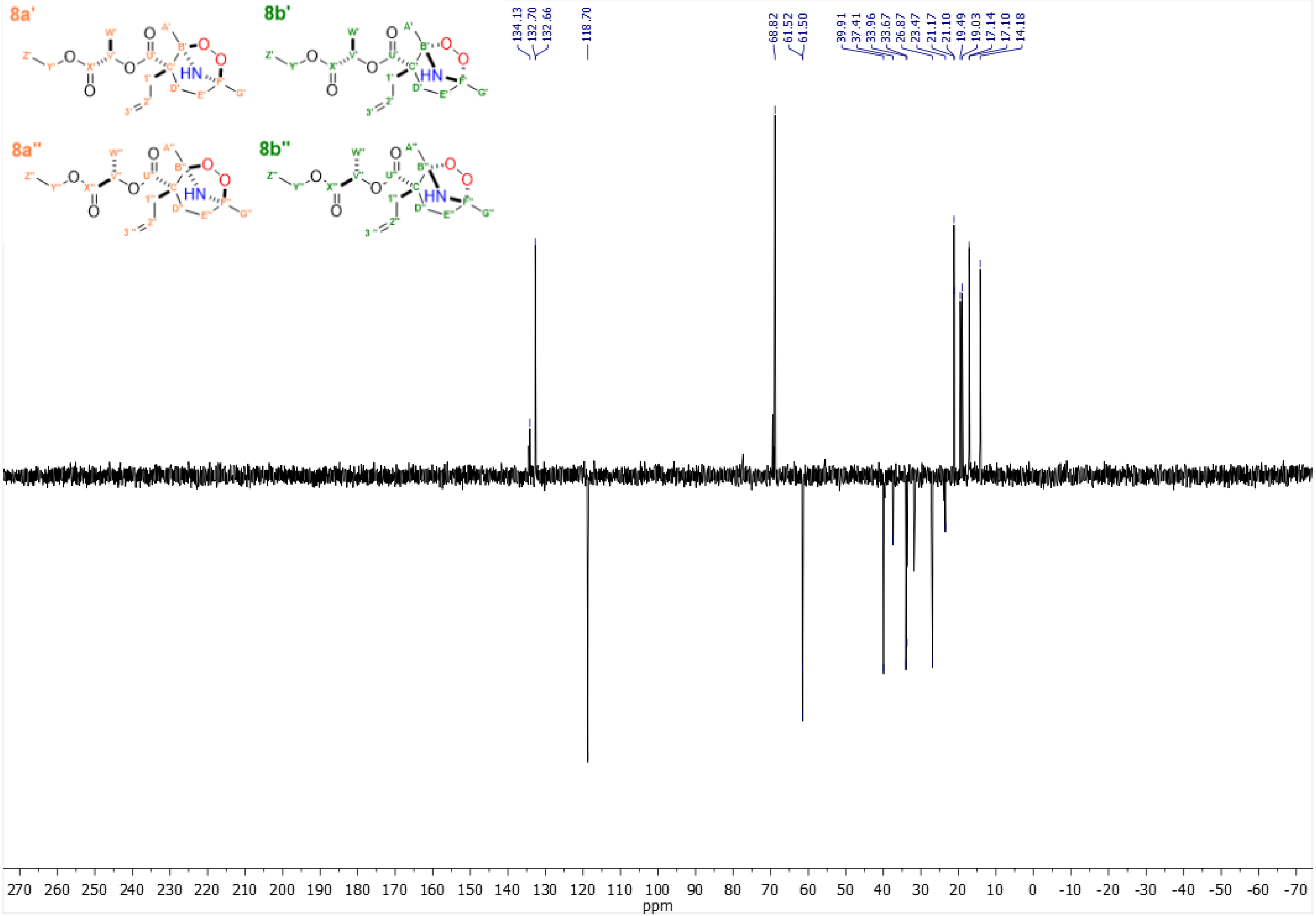

1-ethoxy-1-oxopropan-2-yl -2-allyl-1,5-dimethyl-6,7-dioxa-8-azabicyclo[3.2.1]octane-2-carboxylate, 8a', 8a'', 8b', 8b''

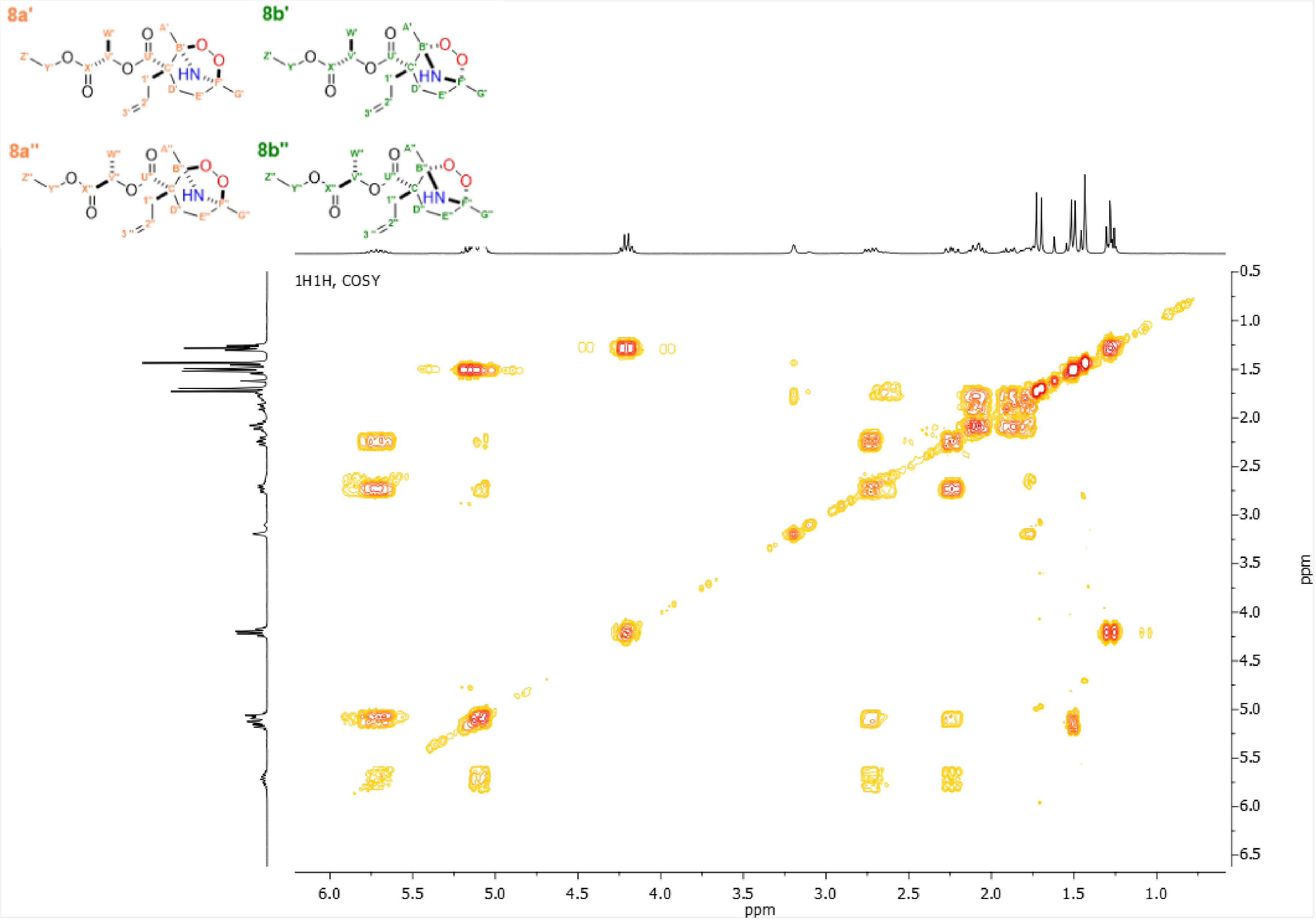

1-ethoxy-1-oxopropan-2-yl -2-allyl-1,5-dimethyl-6,7-dioxa-8-azabicyclo[3.2.1]octane-2-carboxylate, 8a’, 8a’’, 8b’, 8b”

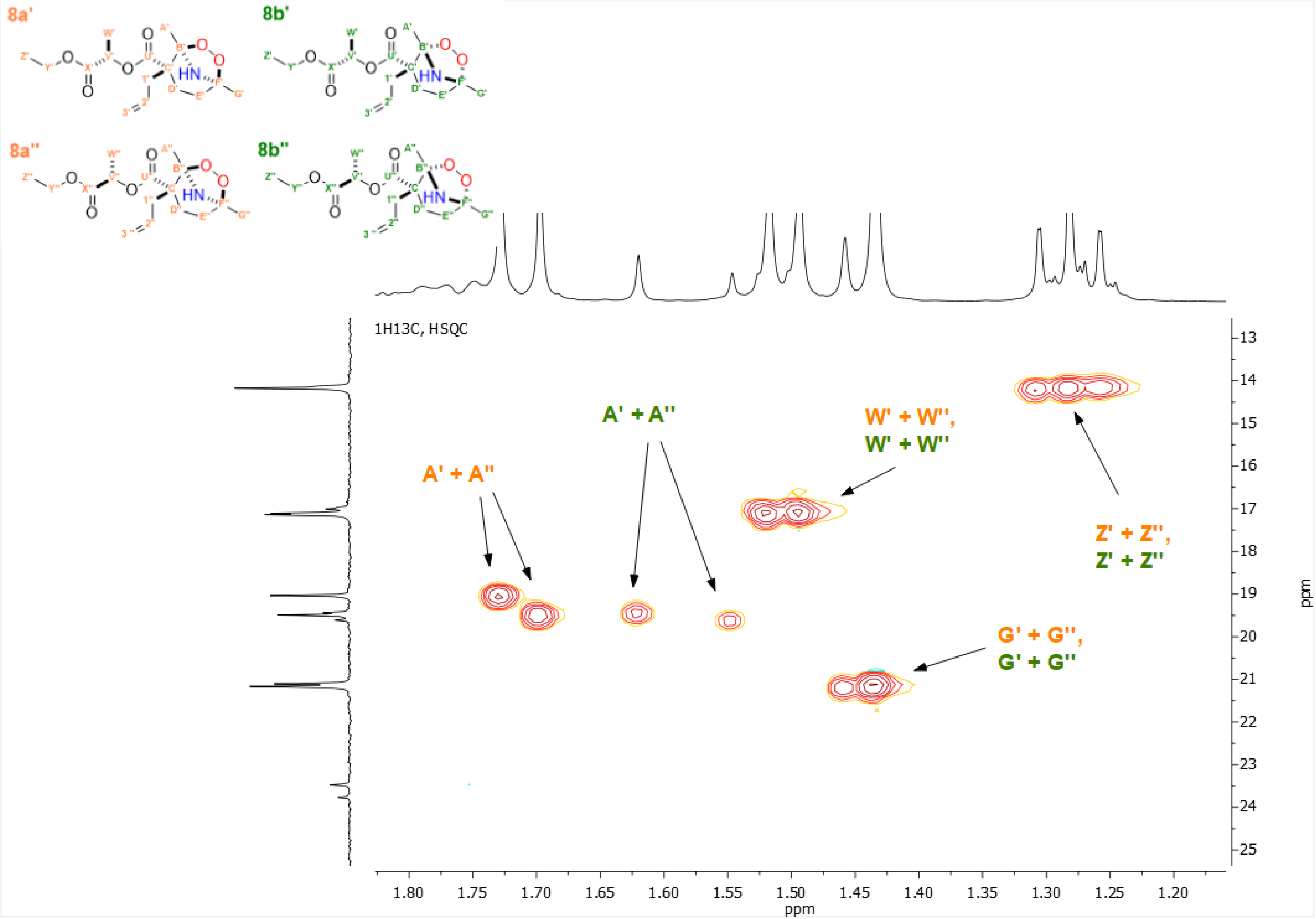

1-ethoxy-1-oxopropan-2-yl -2-allyl-1,5-dimethyl-6,7-dioxa-8-azabicyclo[3.2.1]octane-2-carboxylate, 8a’, 8a’’, 8b’, 8b”

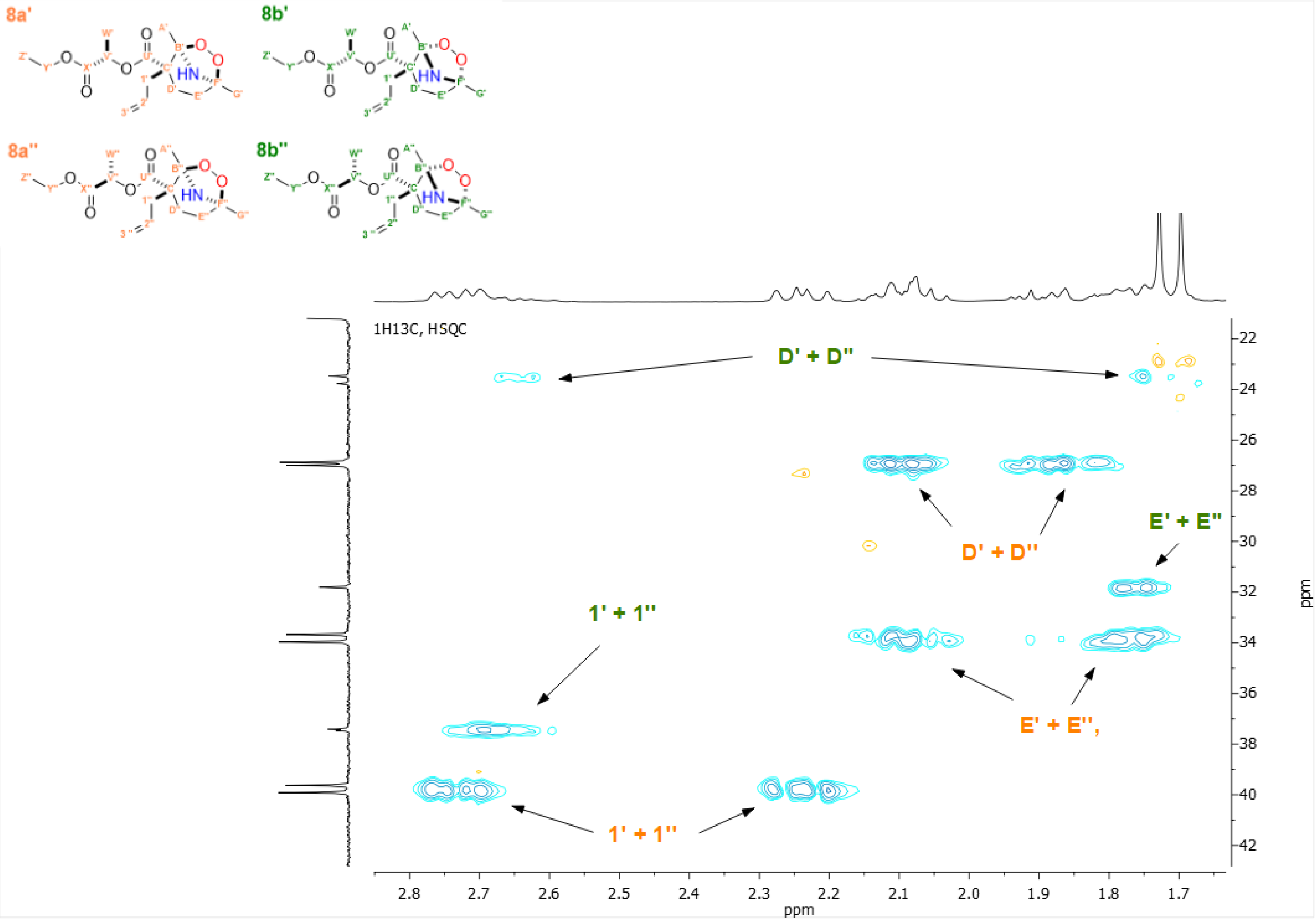

1-ethoxy-1-oxopropan-2-yl -2-allyl-1,5-dimethyl-6,7-dioxa-8-azabicyclo[3.2.1]octane-2-carboxylate, 8a', 8a'', 8b', 8b"

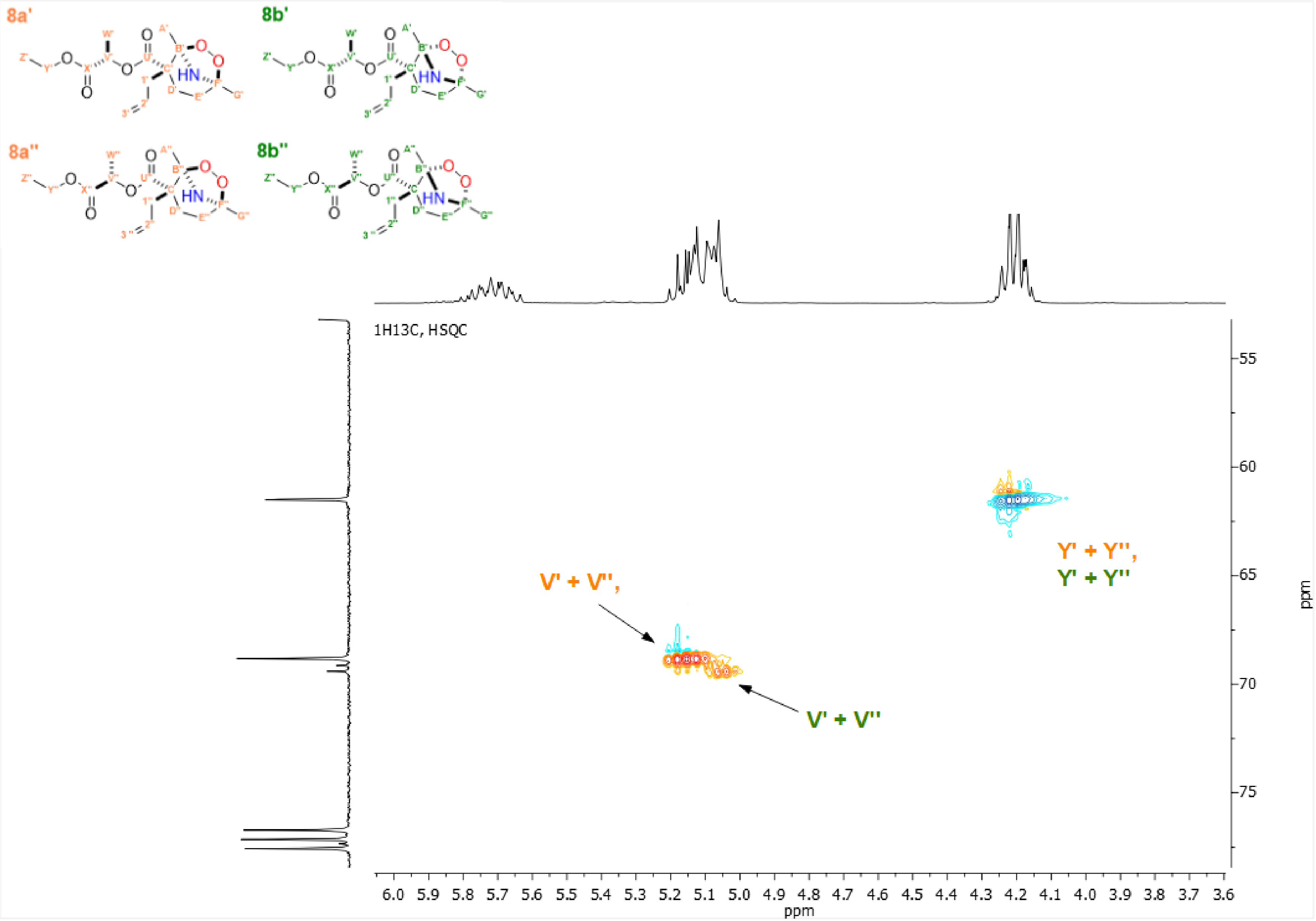

1-ethoxy-1-oxopropan-2-yl -2-allyl-1,5-dimethyl-6,7-dioxa-8-azabicyclo[3.2.1]octane-2-carboxylate, 8a', 8a'', 8b', 8b''

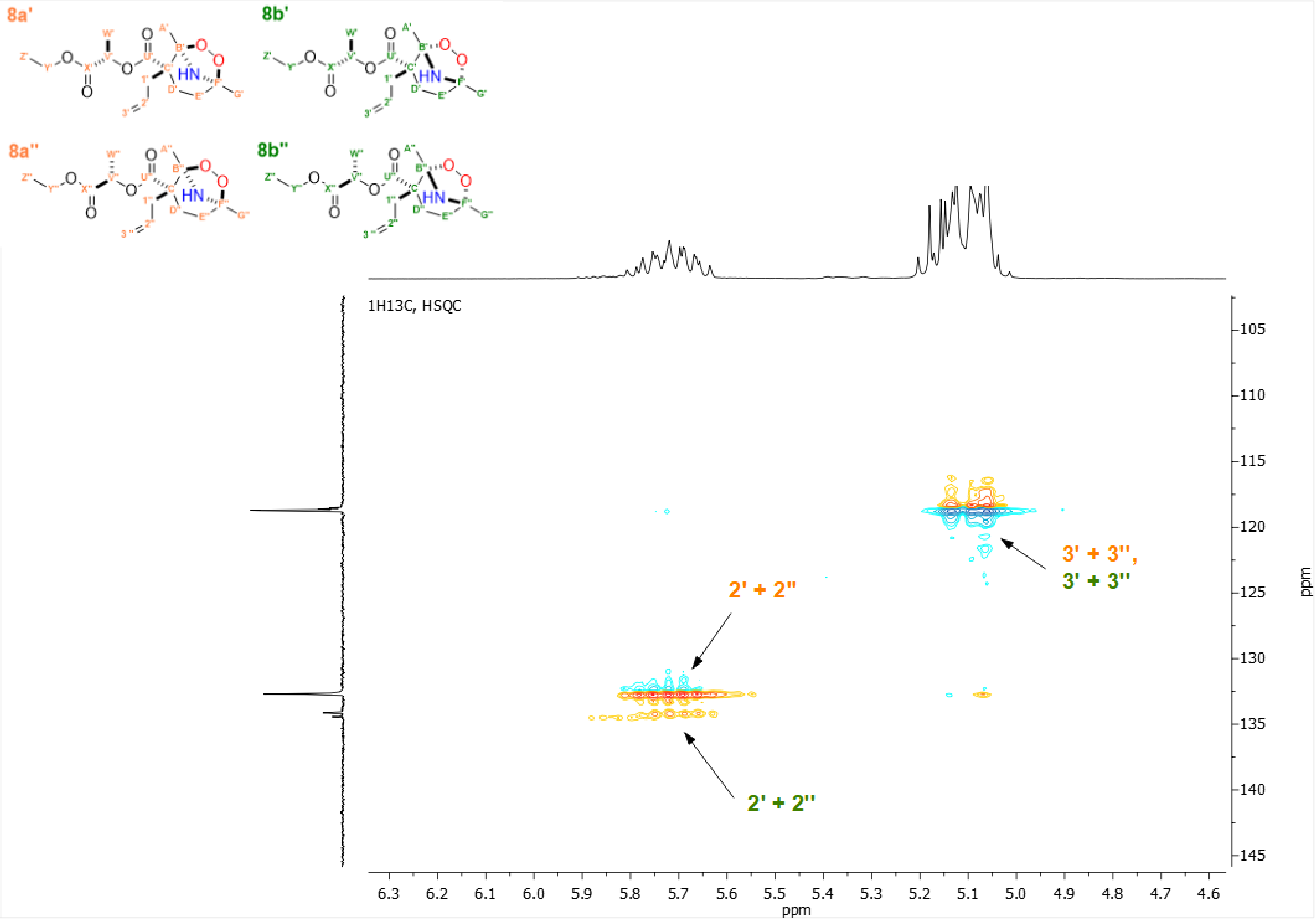

1-ethoxy-1-oxopropan-2-yl -2-allyl-1,5-dimethyl-6,7-dioxa-8-azabicyclo[3.2.1]octane-2-carboxylate, 8a', 8a'', 8b', 8b''

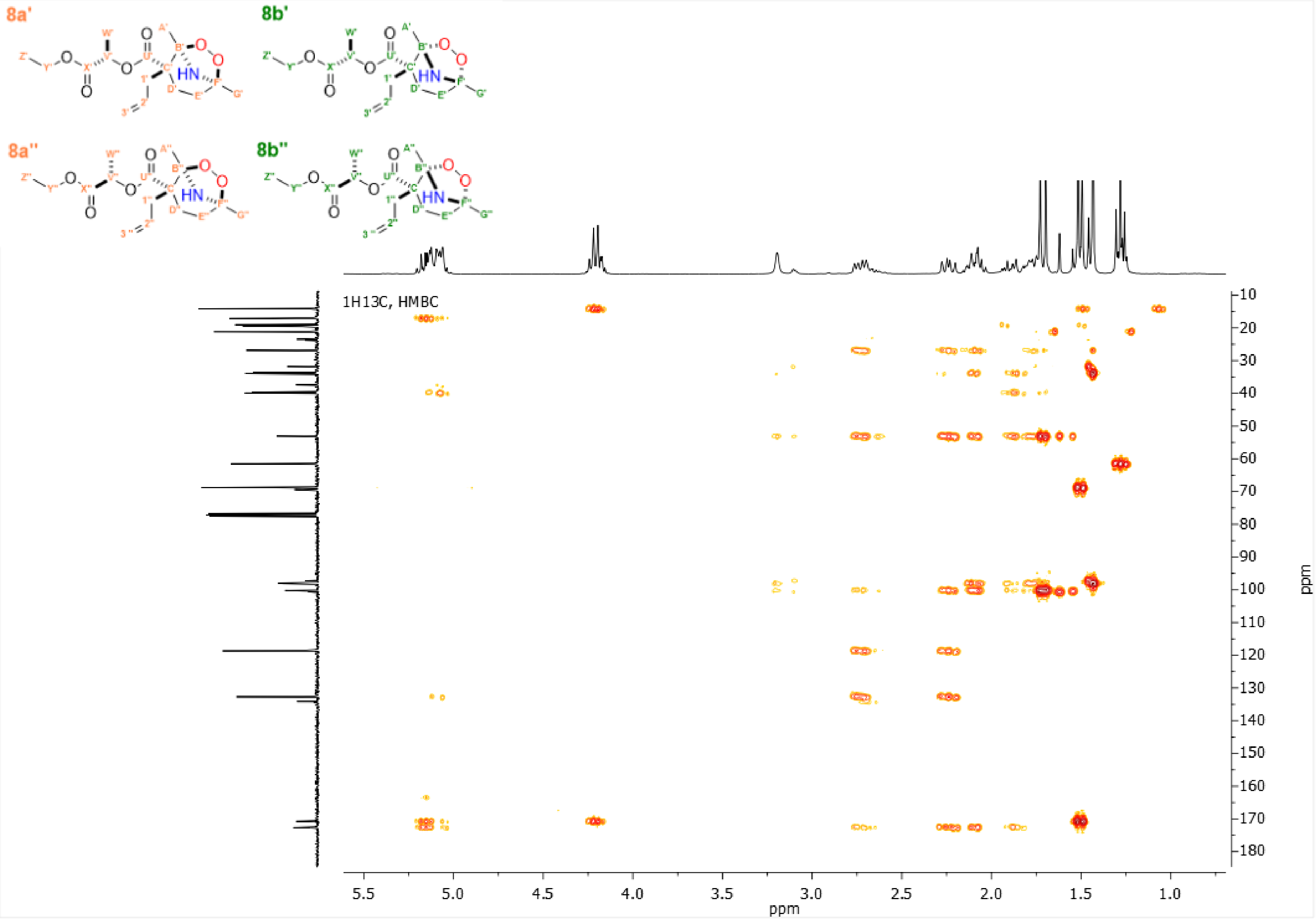

1-ethoxy-1-oxopropan-2-yl -2-allyl-1,5-dimethyl-6,7-dioxa-8-azabicyclo[3.2.1]octane-2-carboxylate, 8a’, 8a’’, 8b’, 8b”

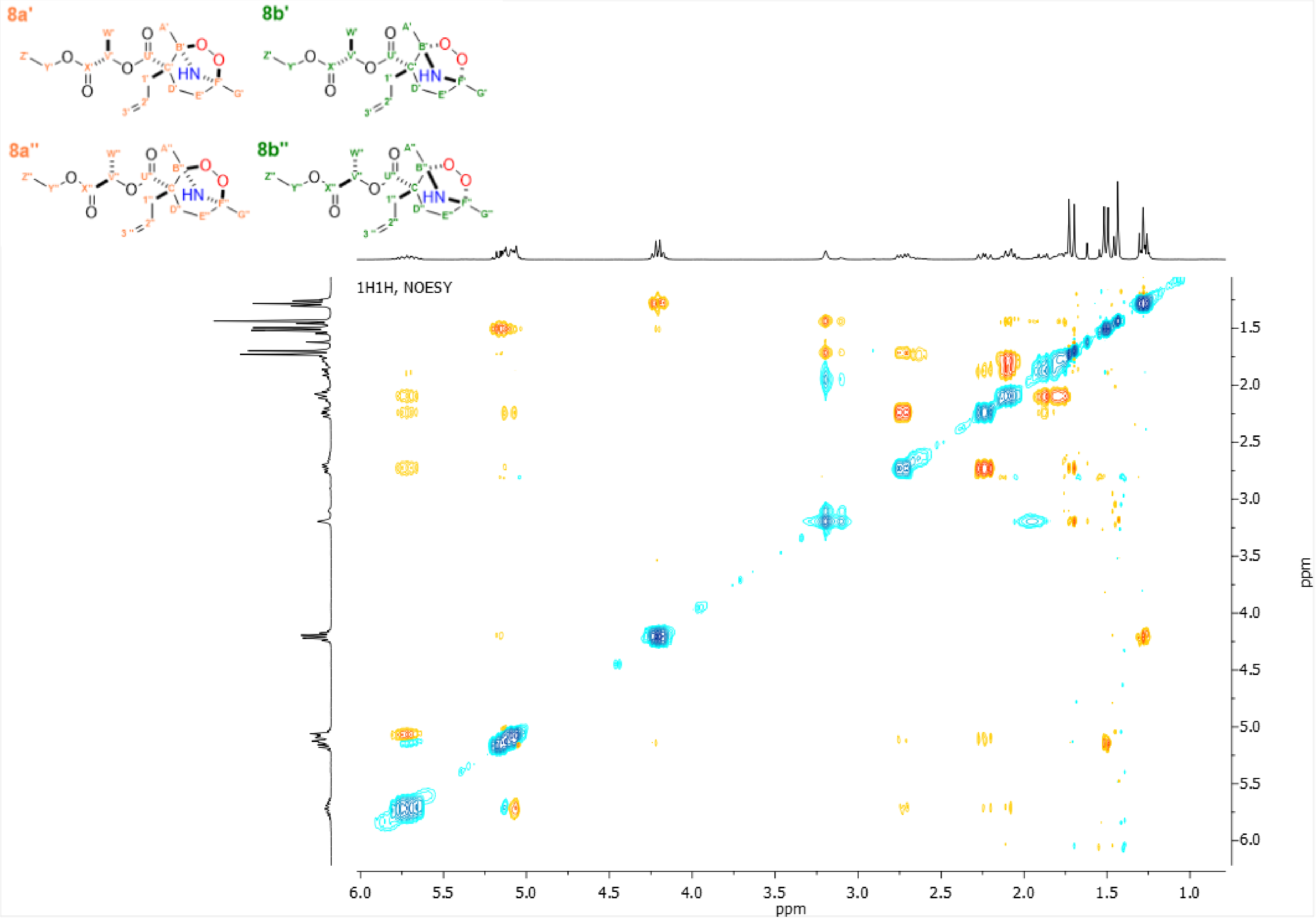

1-ethoxy-1-oxopropan-2-yl -2-allyl-1,5-dimethyl-6,7-dioxa-8-azabicyclo[3.2.1]octane-2-carboxylate, 8a’, 8a’’, 8b’, 8b”

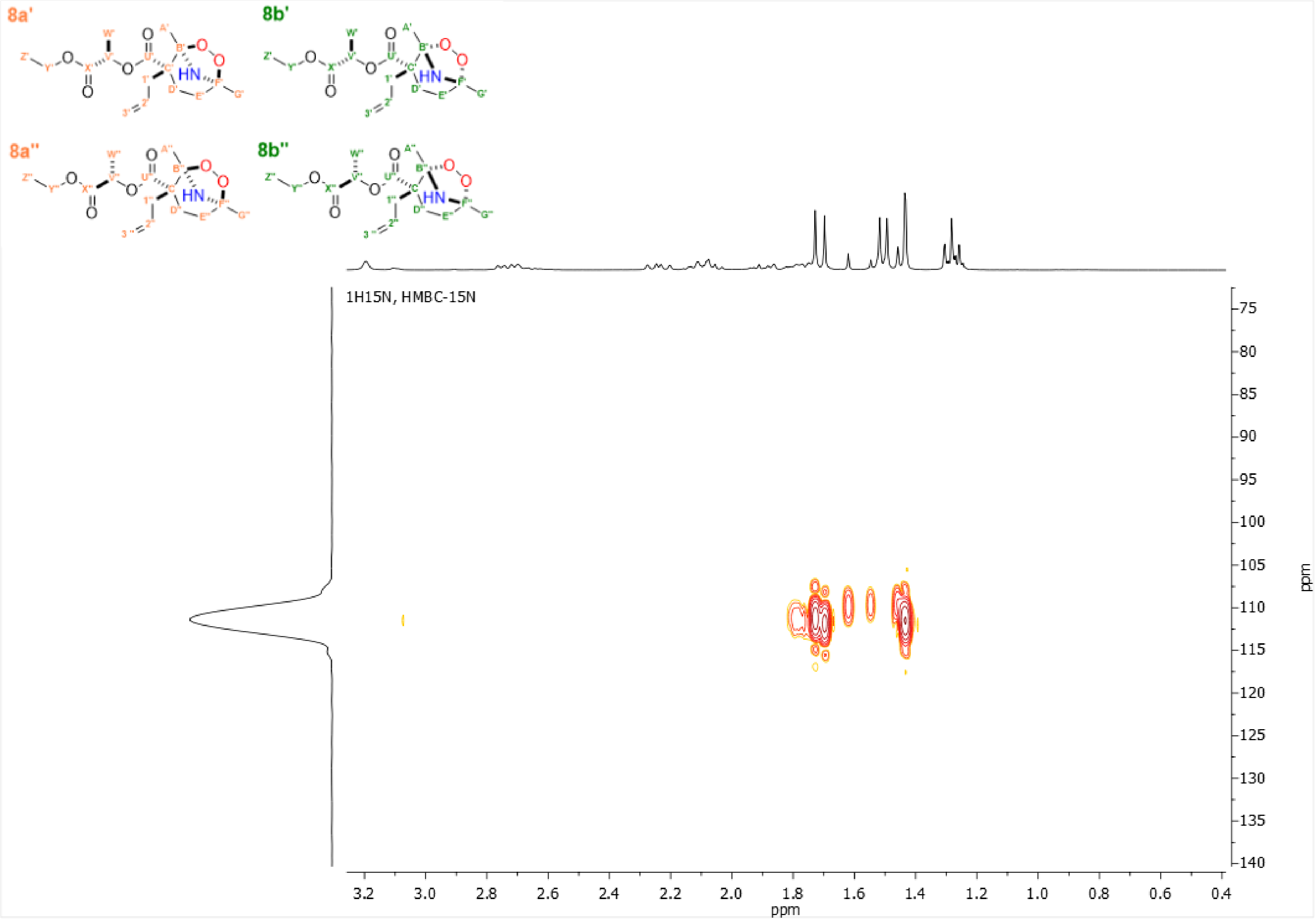

1-ethoxy-1-oxopropan-2-yl -2-allyl-1,5-dimethyl-6,7-dioxa-8-azabicyclo[3.2.1]octane-2-carboxylate, 8a’, 8a’’, 8b’, 8b”

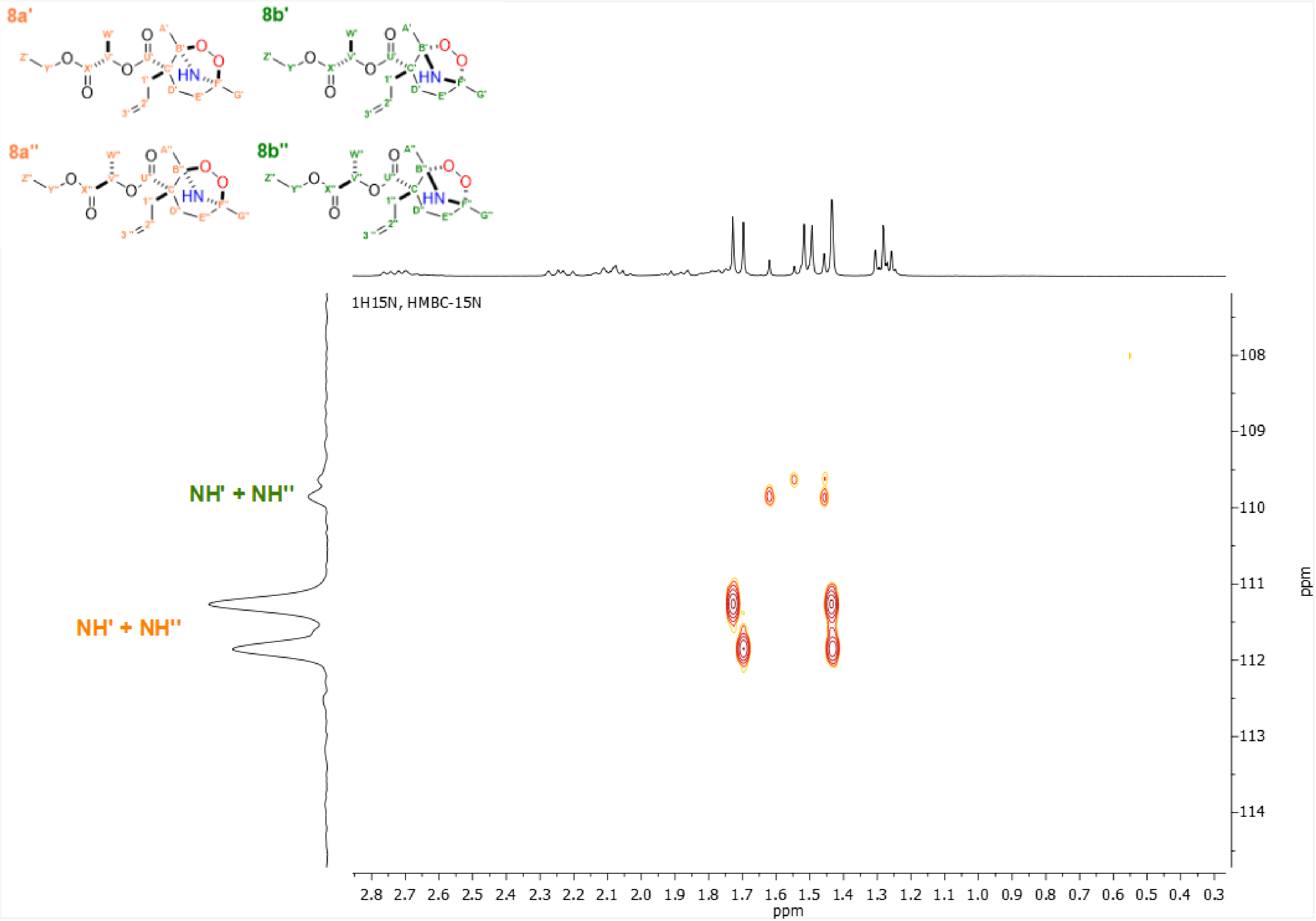

1-ethoxy-1-oxopropan-2-yl -2-allyl-1,5-dimethyl-6,7-dioxa-8-azabicyclo[3.2.1]octane-2-carboxylate, 8a', 8a'', 8b', 8b''

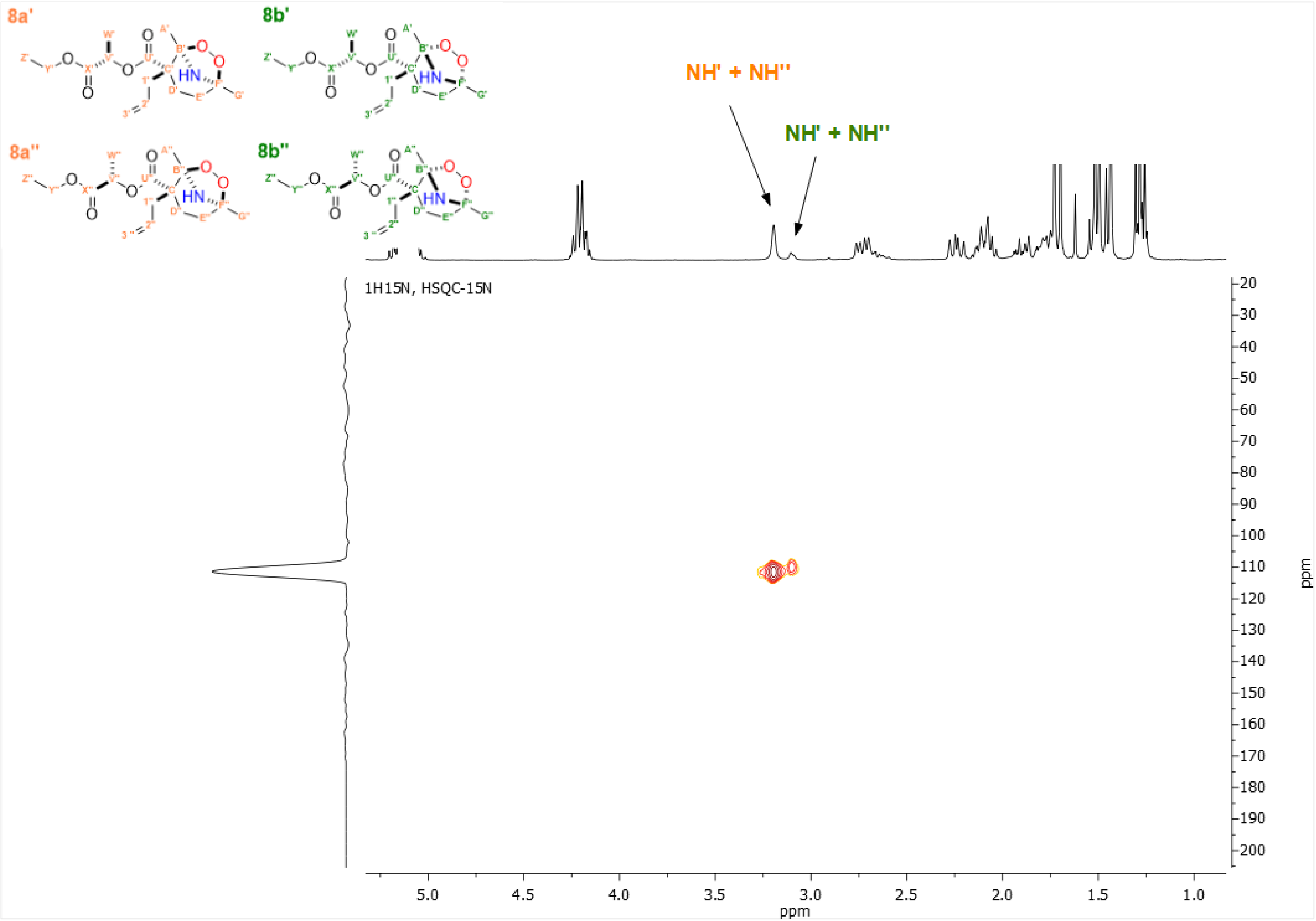

^{1}H NMR (300.13 MHz, CDCl3). Ethyl 2-(4-chlorobenzyl)-1,5-dimethyl-8-ureido-6,7-dioxa-8-azabicyclo[3.2.1]octane-2-carboxylate, 9a

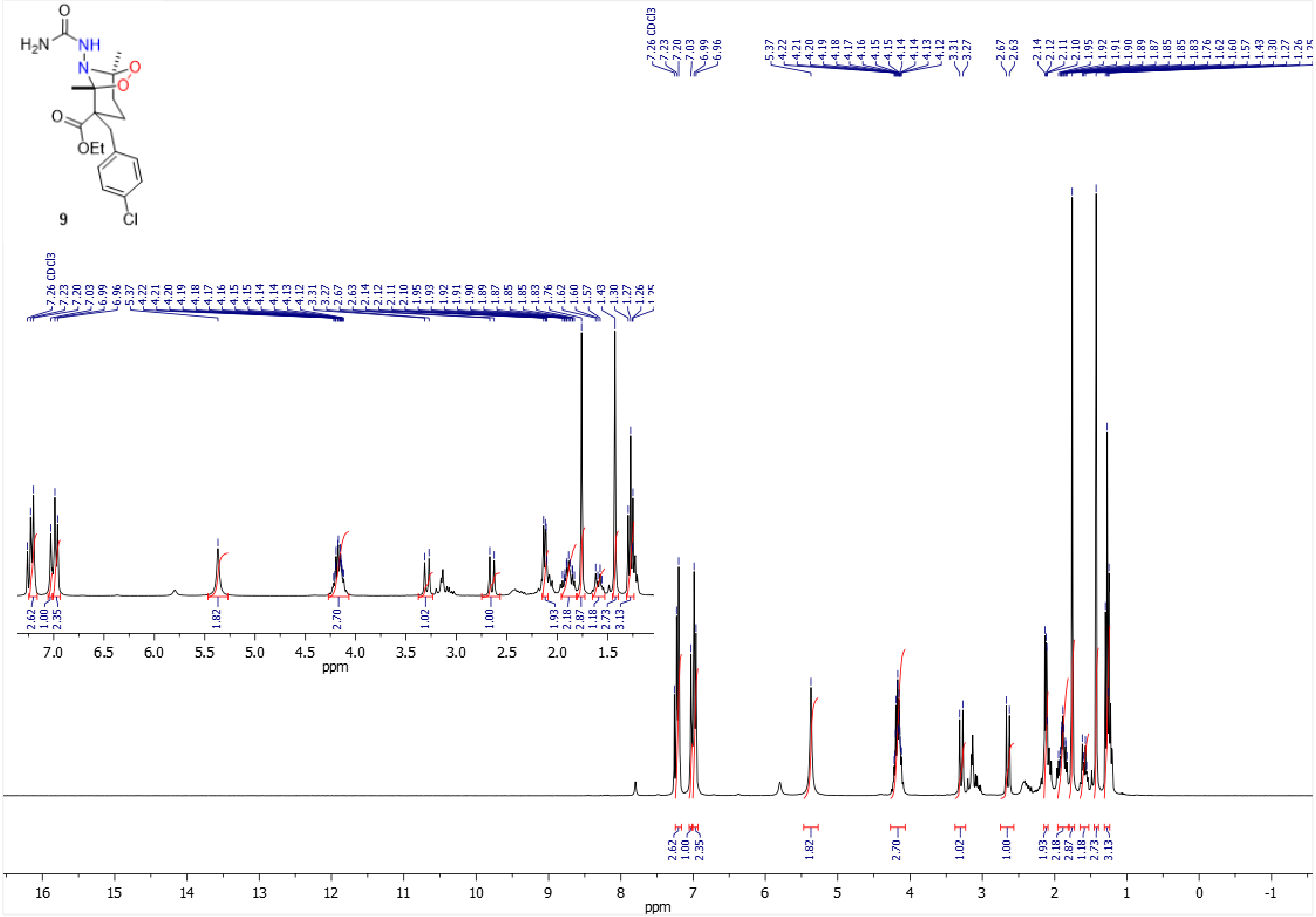

^{13}C NMR (75.48 MHz, CDCl₃). Ethyl 2-(4-chlorobenzyl)-1,5-dimethyl-8-ureido-6,7-dioxa-8-azabicyclo[3.2.1]octane-2-carboxylate, 9a

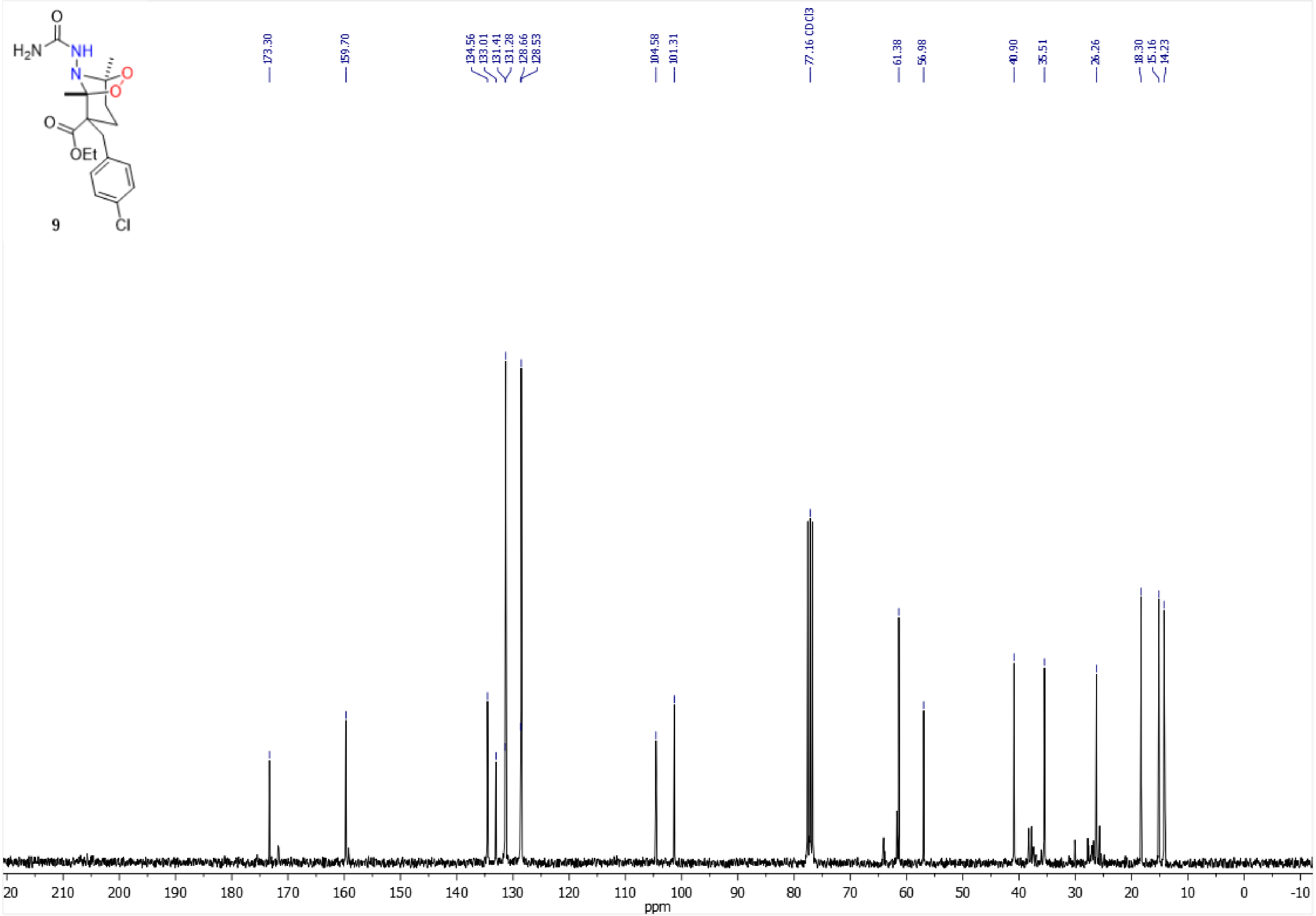

^{1}H NMR (300.13 MHz, $CDCl_3$). Ethyl 2-butyl-1,5-dimethyl-8-ureido-6,7-dioxa-8-azabicyclo[3.2.1]octane-2-carboxylate, 10a

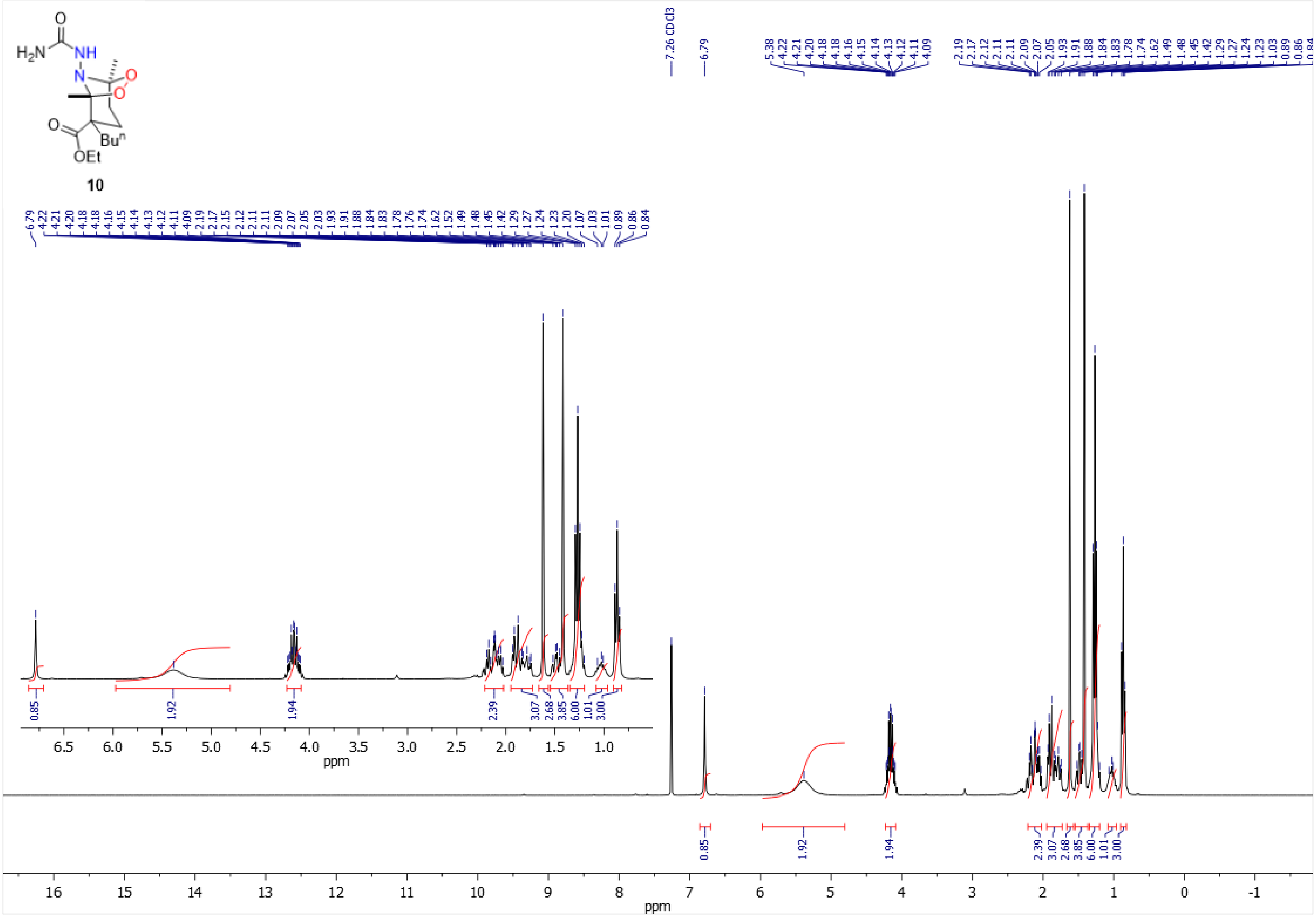

^{13}C NMR (75.48 MHz, $CDCl_3$). Ethyl 2-butyl-1,5-dimethyl-8-ureido-6,7-dioxa-8-azabicyclo[3.2.1]octane-2-carboxylate, 10a

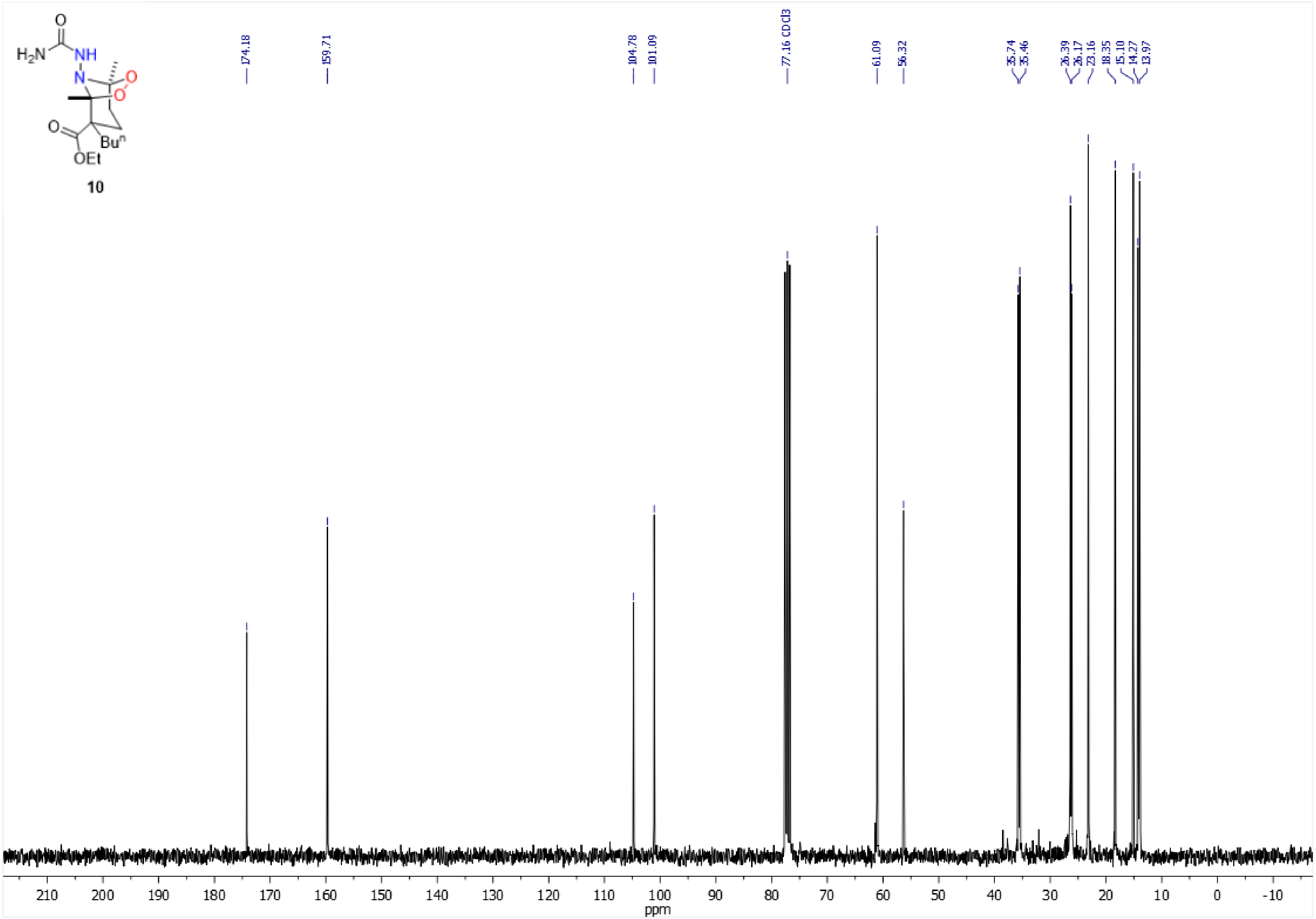

^{1}H NMR (300.13 MHz, $CDCl_3$). Ethyl 2-(3-ethoxy-3-oxopropyl)-1,5-dimethyl-8-ureido-6,7-dioxa-8-azabicyclo[3.2.1]octane-2-carboxylate, 11a

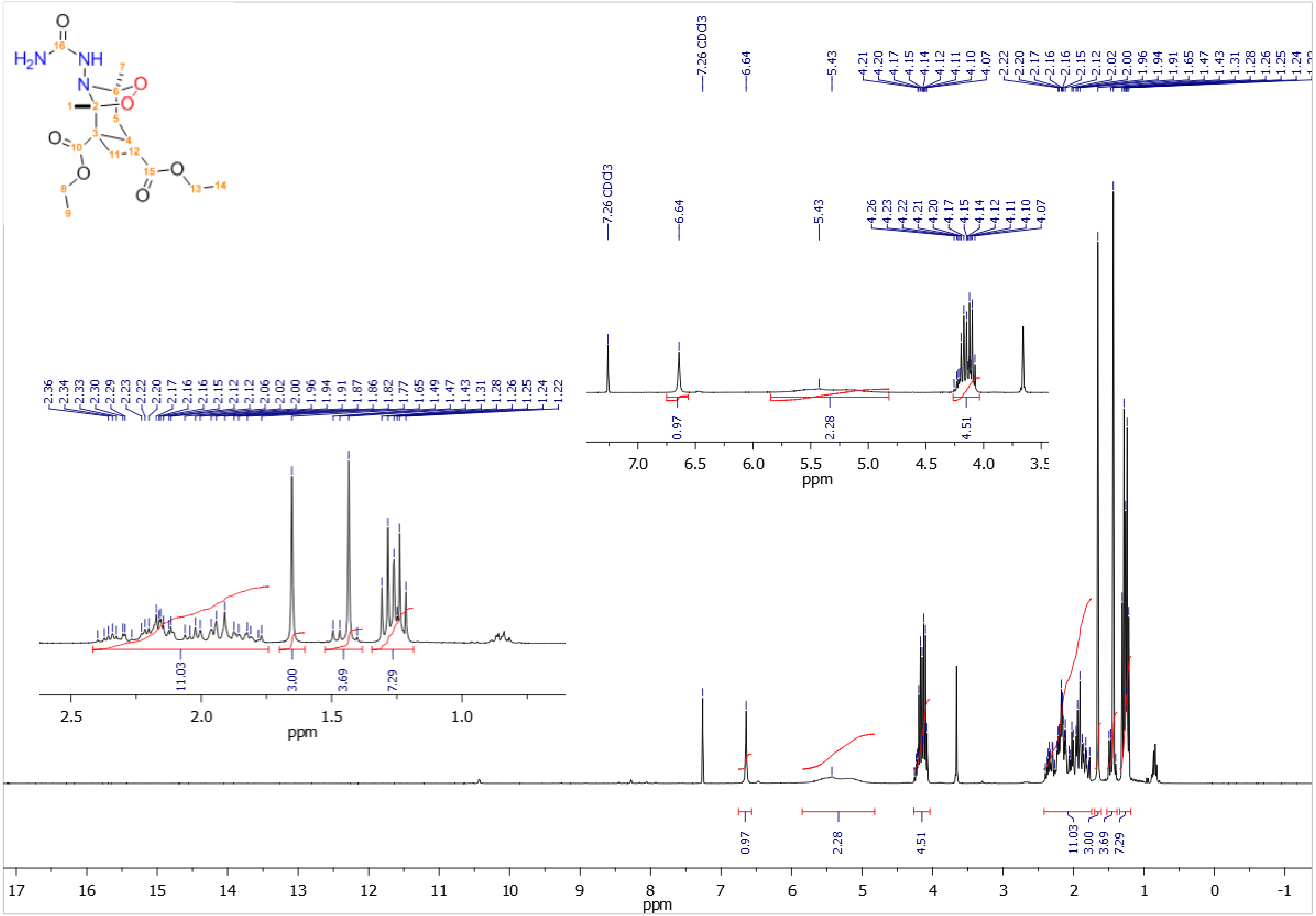

^{1}H NMR (300.13 MHz, CDCl3). Ethyl 2-(3-ethoxy-3-oxopropyl)-1,5-dimethyl-8-ureido-6,7-dioxa-8-azabicyclo[3.2.1]octane-2-carboxylate, 11a

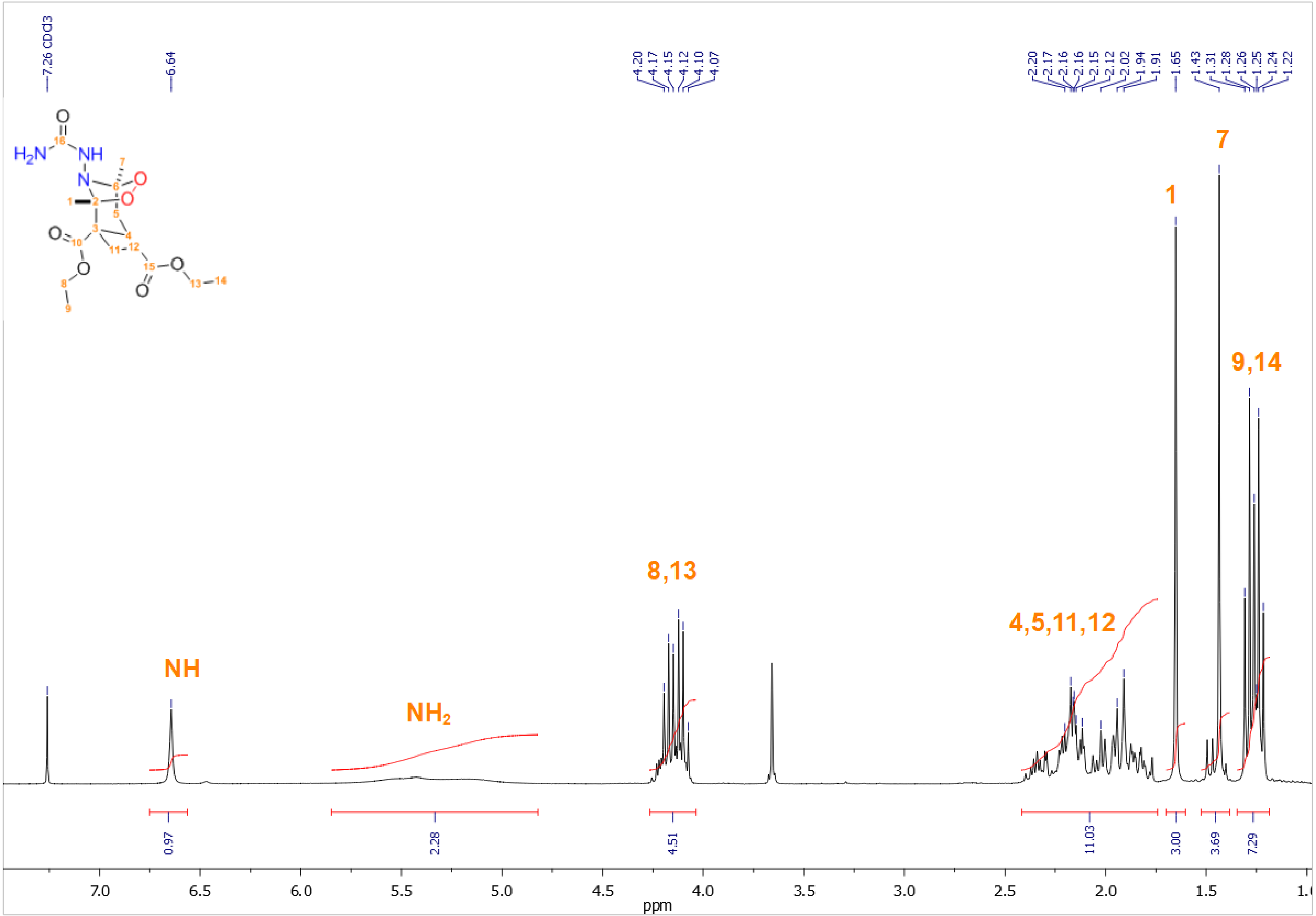

^{13}C NMR (75.48 MHz, $CDCl_3$). Ethyl 2-(3-ethoxy-3-oxopropyl)-1,5-dimethyl-8-ureido-6,7-dioxa-8-azabicyclo[3.2.1]octane-2-carboxylate, 11a

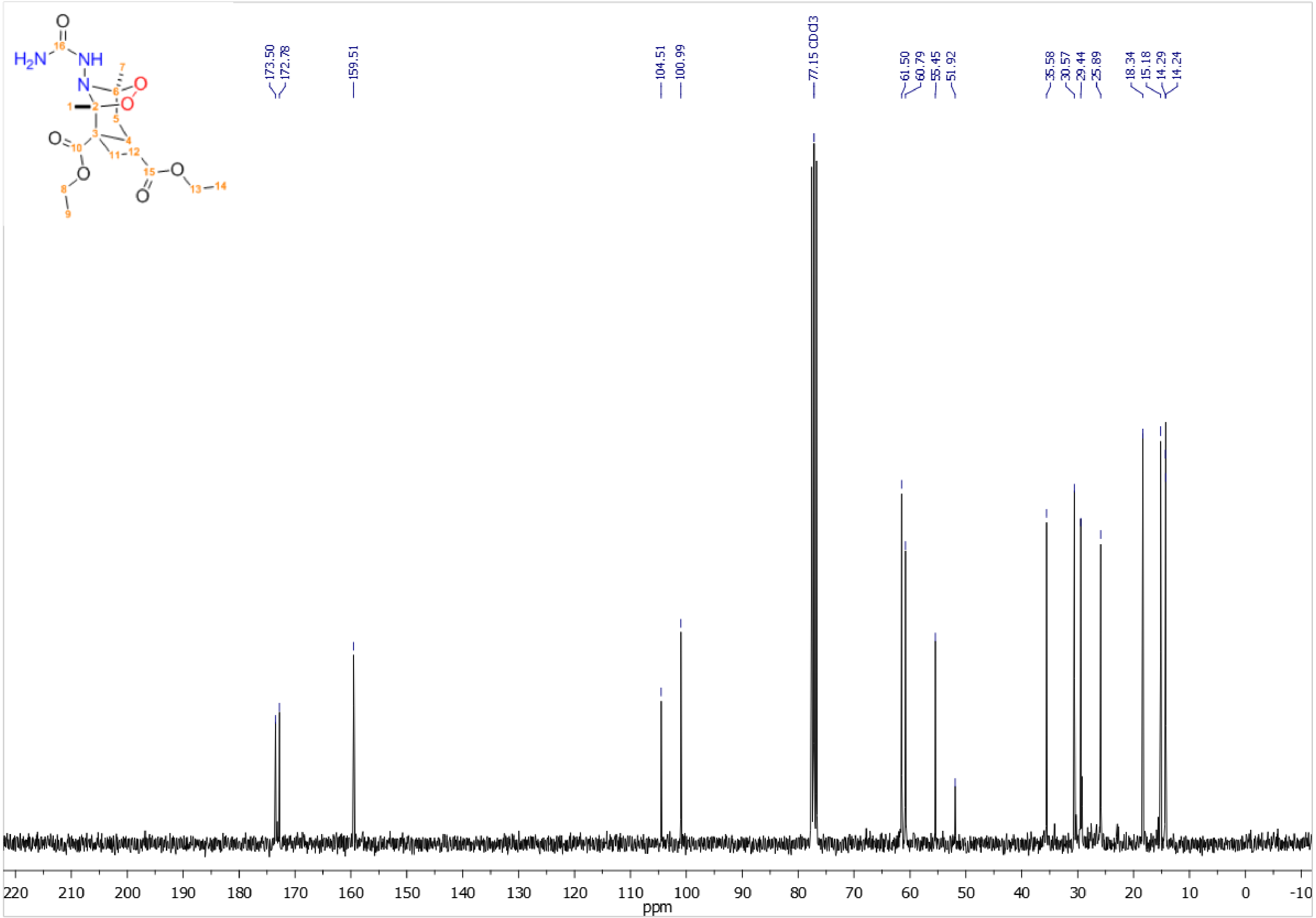

^{13}C NMR (75.48 MHz, $CDCl_3$). Ethyl 2-(3-ethoxy-3-oxopropyl)-1,5-dimethyl-8-ureido-6,7-dioxa-8-azabicyclo[3.2.1]octane-2-carboxylate, 11a

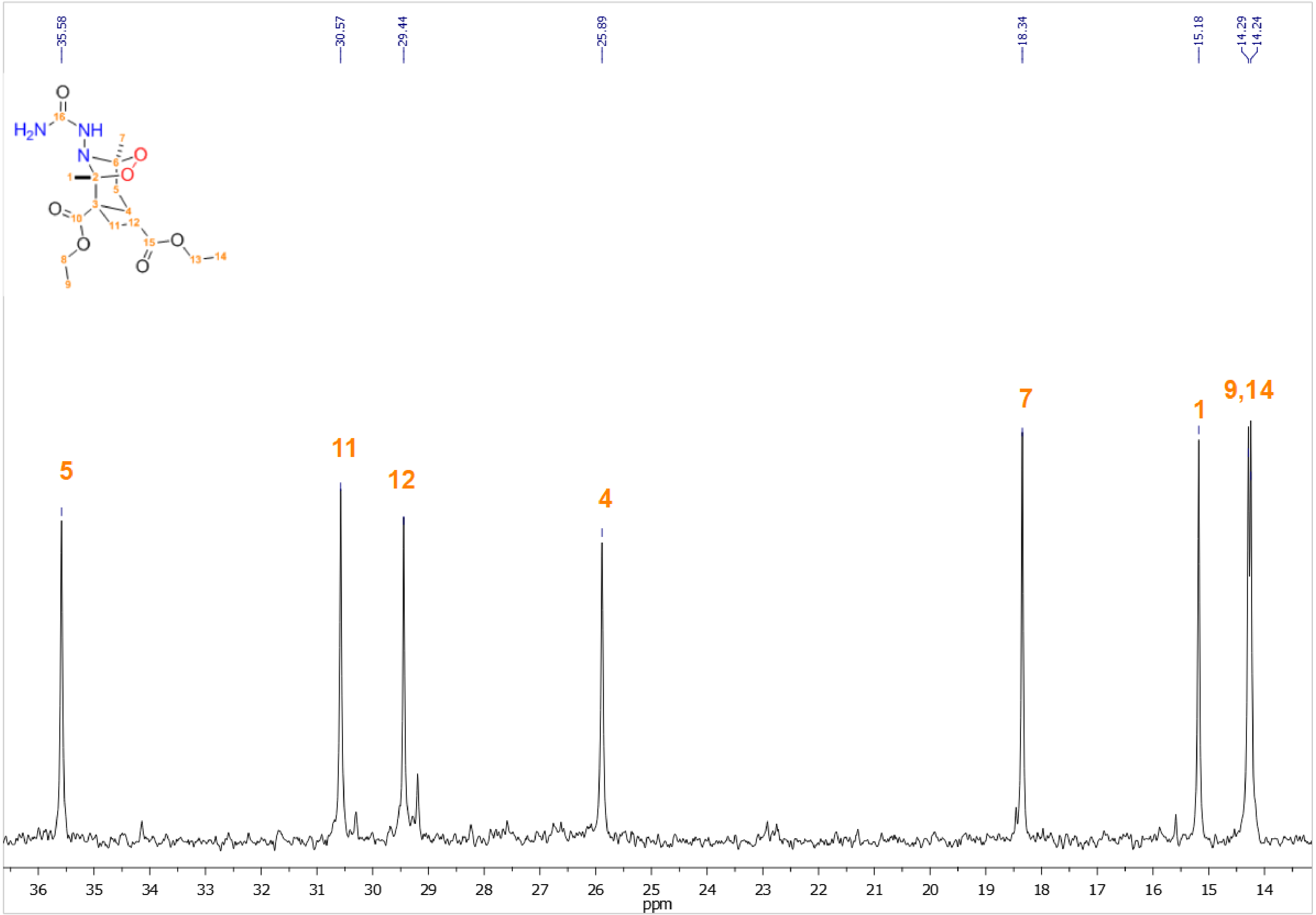

^{13}C NMR (75.48 MHz, CDCl3). Ethyl 2-(3-ethoxy-3-oxopropyl)-1,5-dimethyl-8-ureido-6,7-dioxa-8-azabicyclo[3.2.1]octane-2-carboxylate, 11a

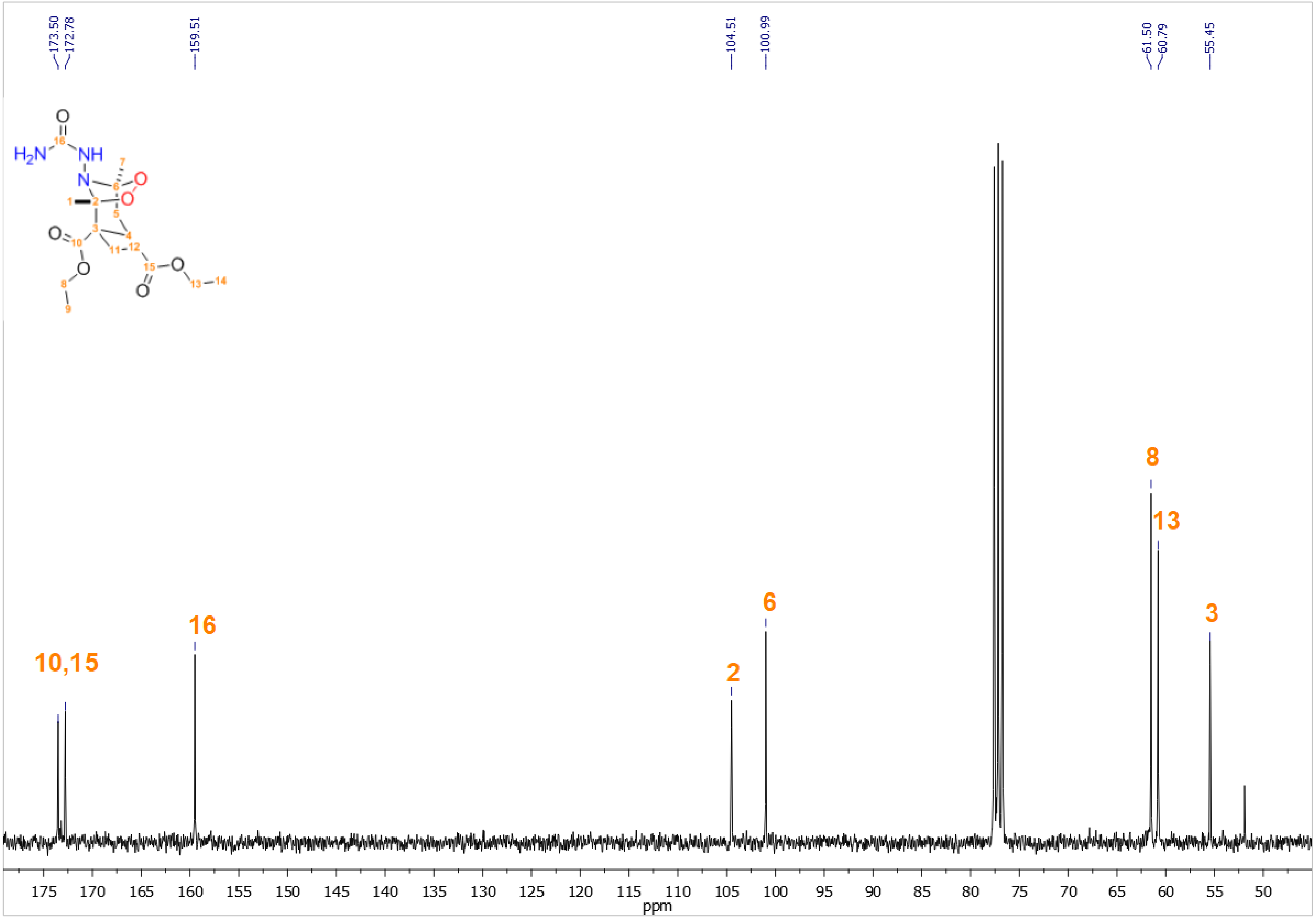

^{15}N (40.56 MHz, CDCl3). Ethyl 2-(3-ethoxy-3-oxopropyl)-1,5-dimethyl-8-ureido-6,7-dioxa-8-azabicyclo[3.2.1]octane-2-carboxylate, 11a

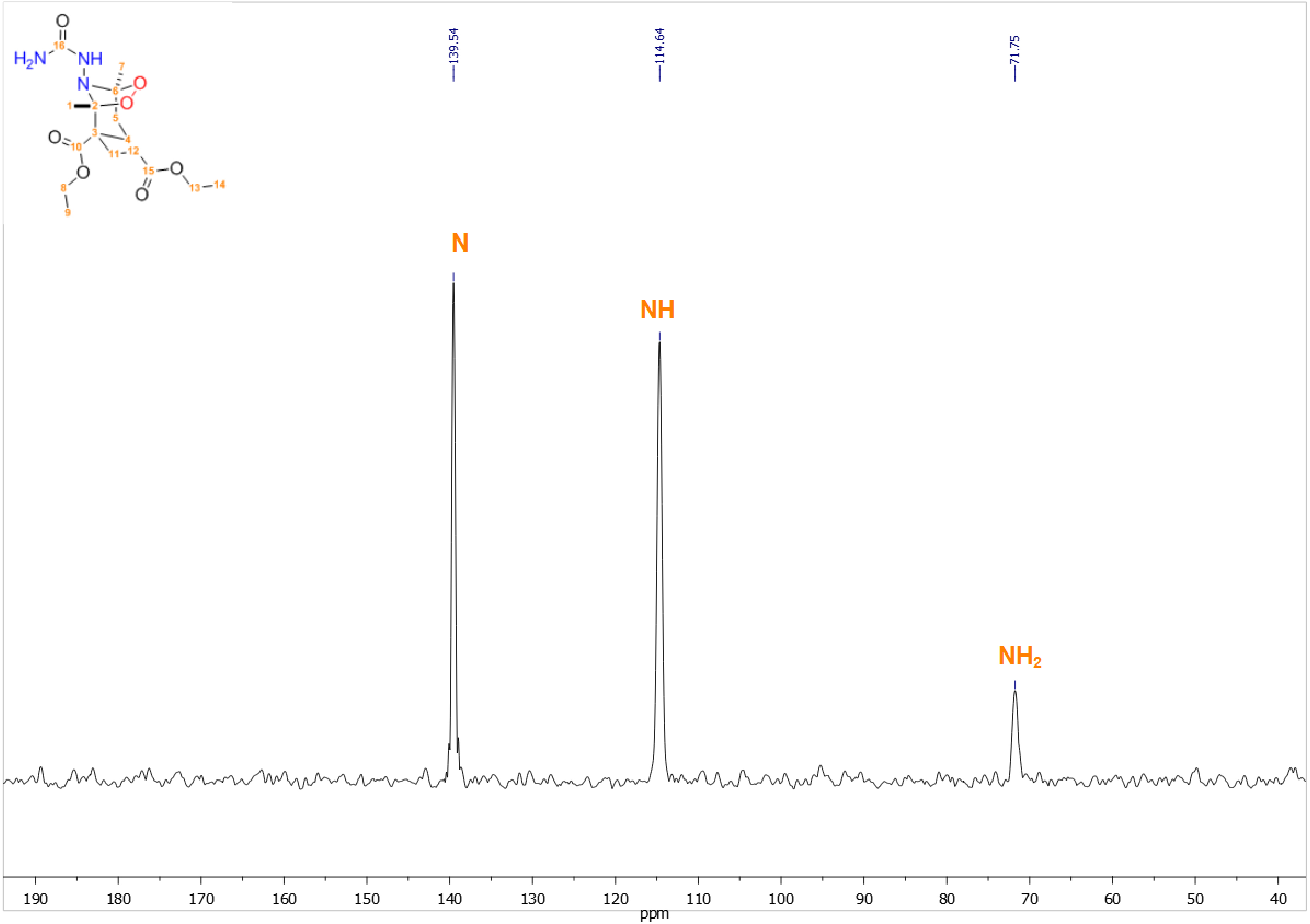

^{13}C NMR (75.48 MHz, $CDCl_3$). Ethyl 2-(3-ethoxy-3-oxopropyl)-1,5-dimethyl-8-ureido-6,7-dioxa-8-azabicyclo[3.2.1]octane-2-carboxylate, 11a

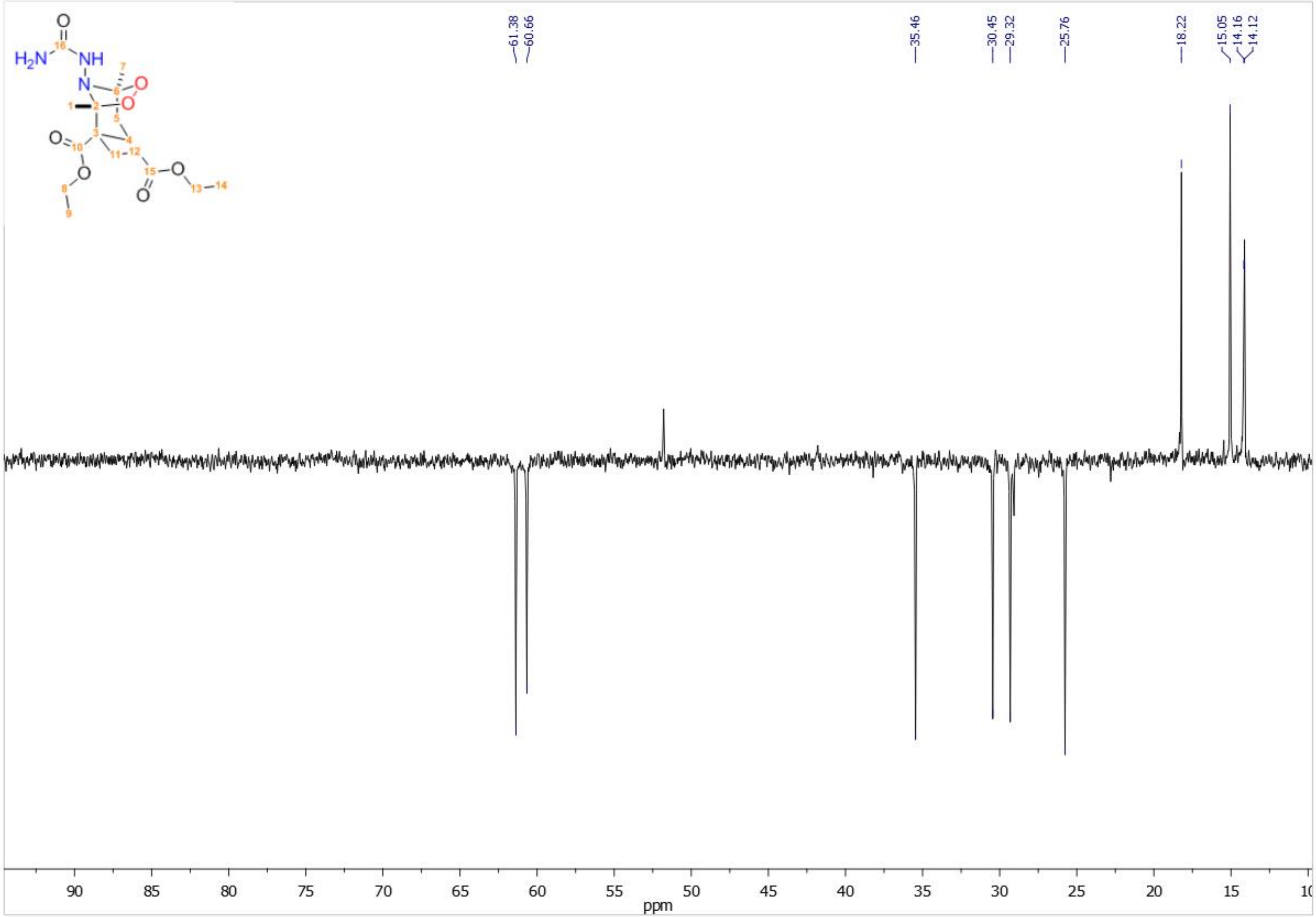

Ethyl 2-(3-ethoxy-3-oxopropyl)-1,5-dimethyl-8-ureido-6,7-dioxa-8-azabicyclo[3.2.1]octane-2-carboxylate, 11a

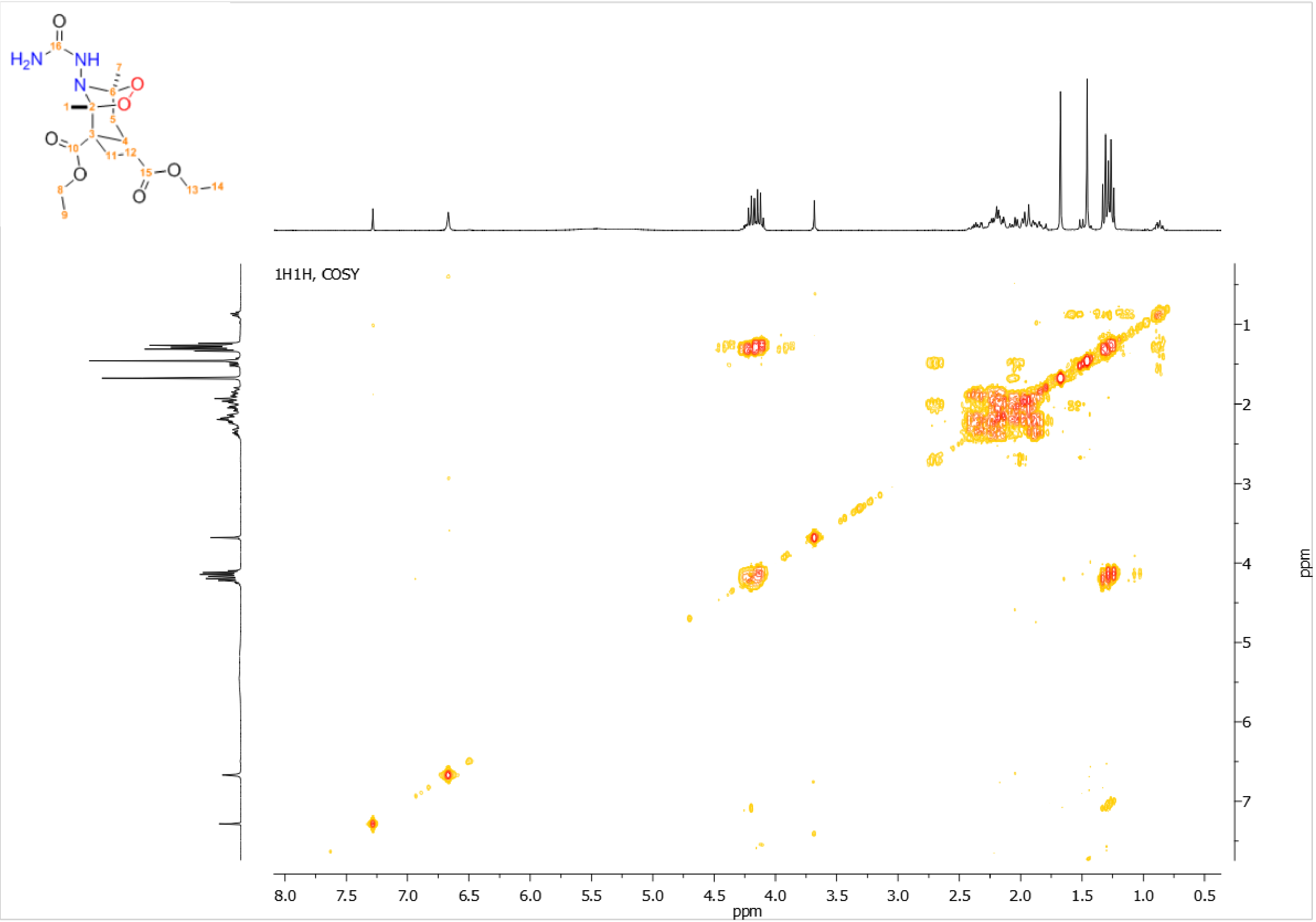

Ethyl 2-(3-ethoxy-3-oxopropyl)-1,5-dimethyl-8-ureido-6,7-dioxa-8-azabicyclo[3.2.1]octane-2-carboxylate, 11a

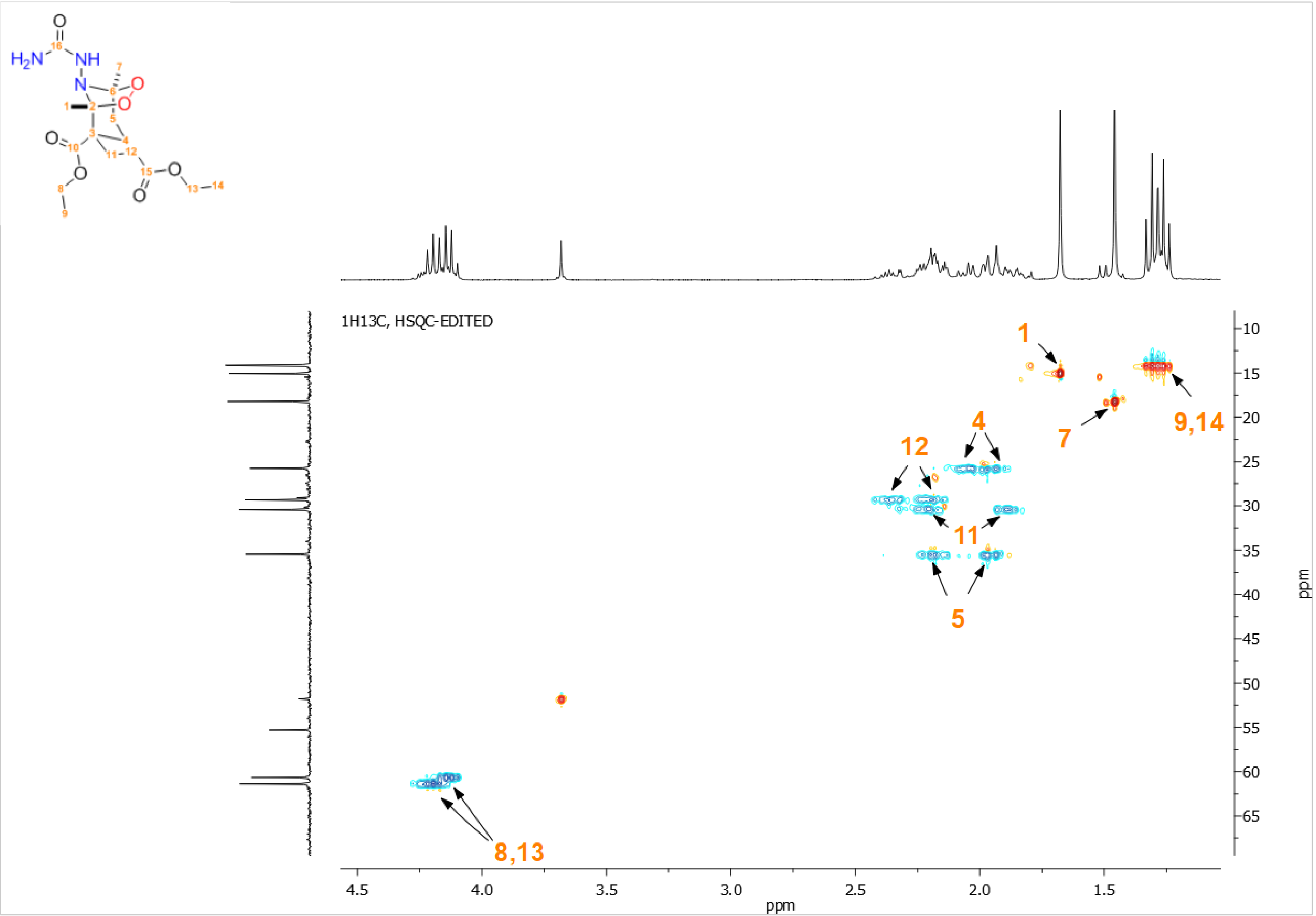

Ethyl 2-(3-ethoxy-3-oxopropyl)-1,5-dimethyl-8-ureido-6,7-dioxa-8-azabicyclo[3.2.1]octane-2-carboxylate, 11a

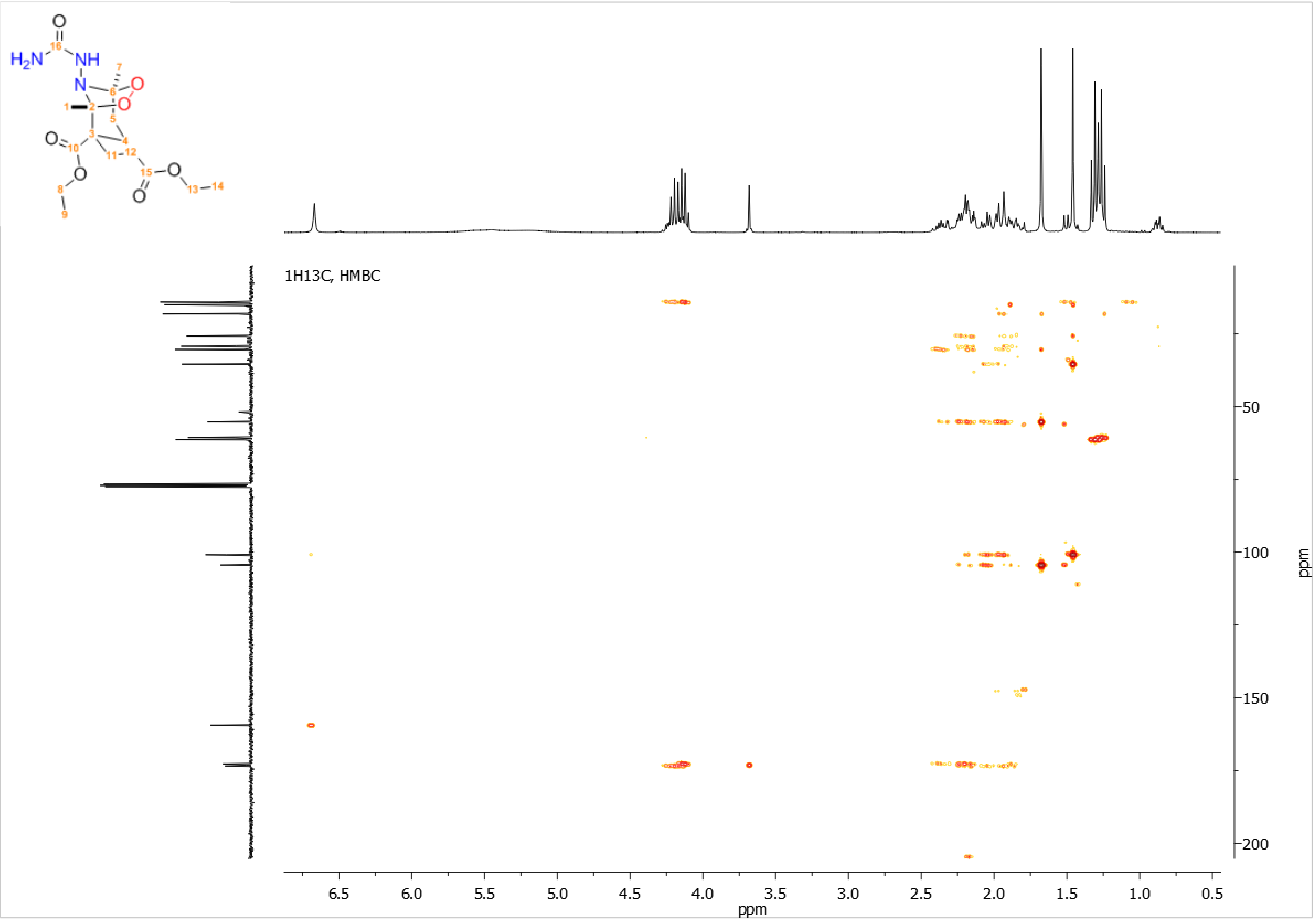

Ethyl 2-(3-ethoxy-3-oxopropyl)-1,5-dimethyl-8-ureido-6,7-dioxa-8-azabicyclo[3.2.1]octane-2-carboxylate, 11a

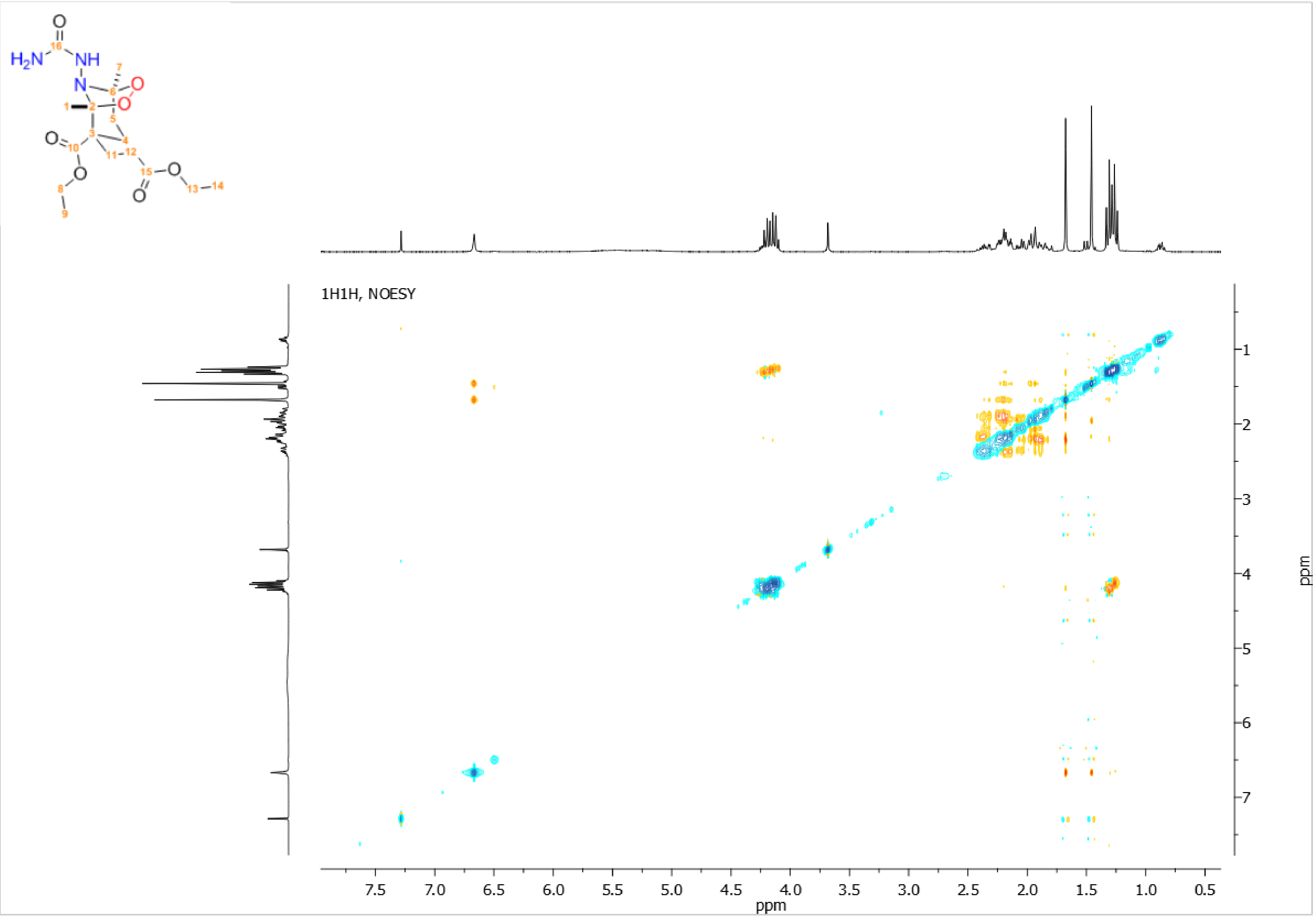

Ethyl 2-(3-ethoxy-3-oxopropyl)-1,5-dimethyl-8-ureido-6,7-dioxa-8-azabicyclo[3.2.1]octane-2-carboxylate, 11a

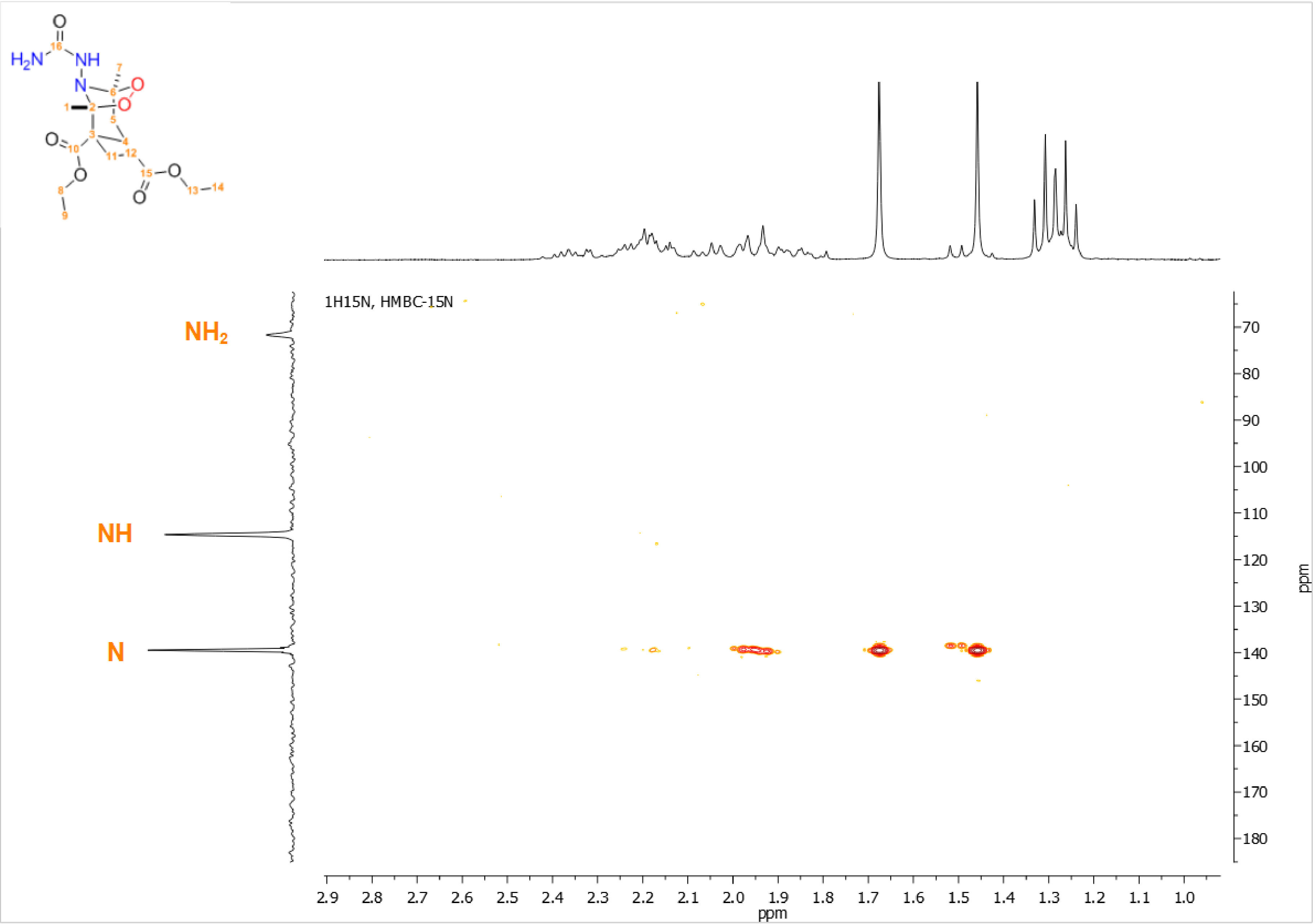

Ethyl 2-(3-ethoxy-3-oxopropyl)-1,5-dimethyl-8-ureido-6,7-dioxa-8-azabicyclo[3.2.1]octane-2-carboxylate, 11a

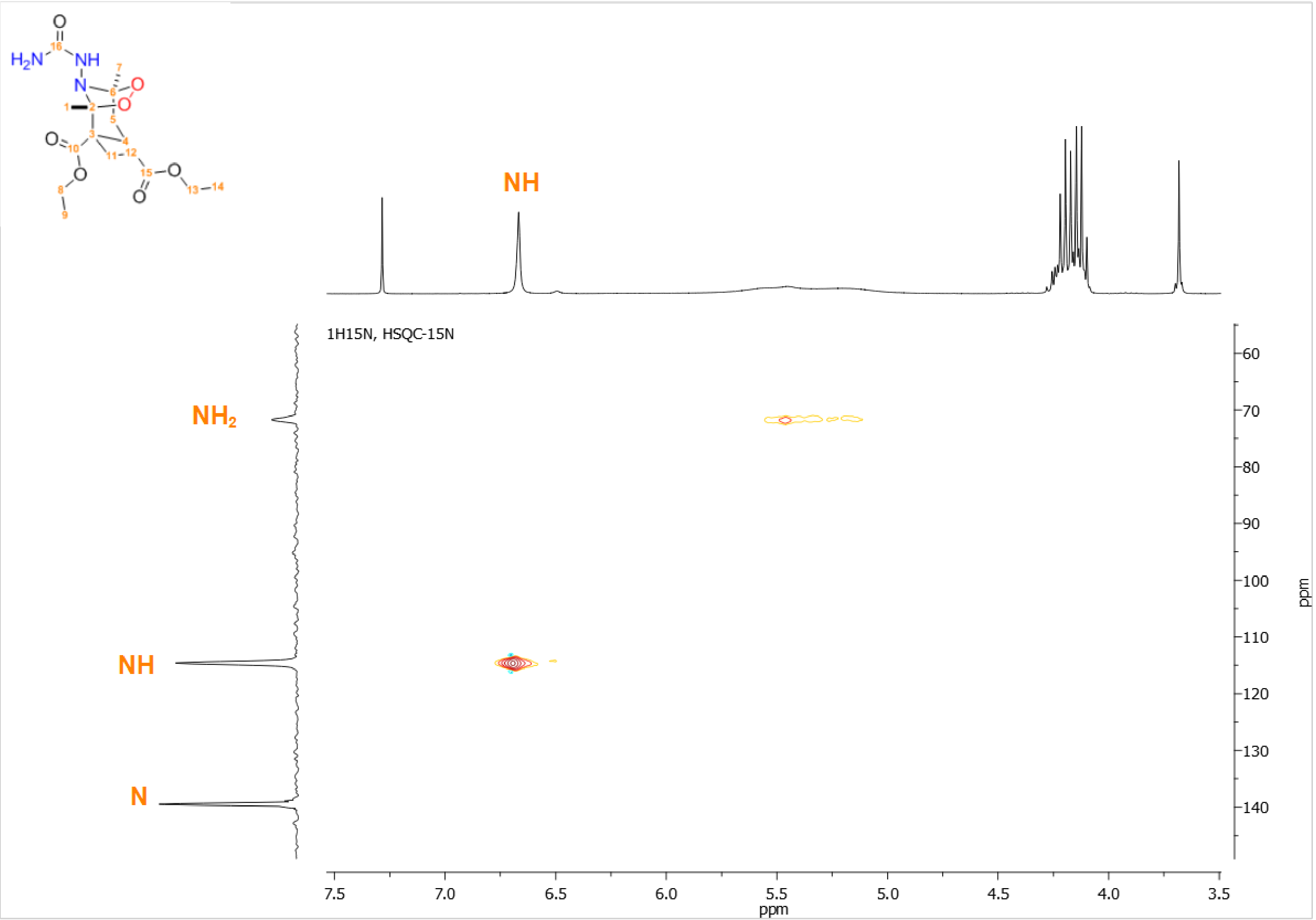

^{1}H NMR (300.13 MHz, $CDCl_3$). Ethyl 2-(4-fluorobenzyl)-1,5-dimethyl-8-ureido-6,7-dioxa-8-azabicyclo[3.2.1]octane-2-carboxylate, 12a + 12b

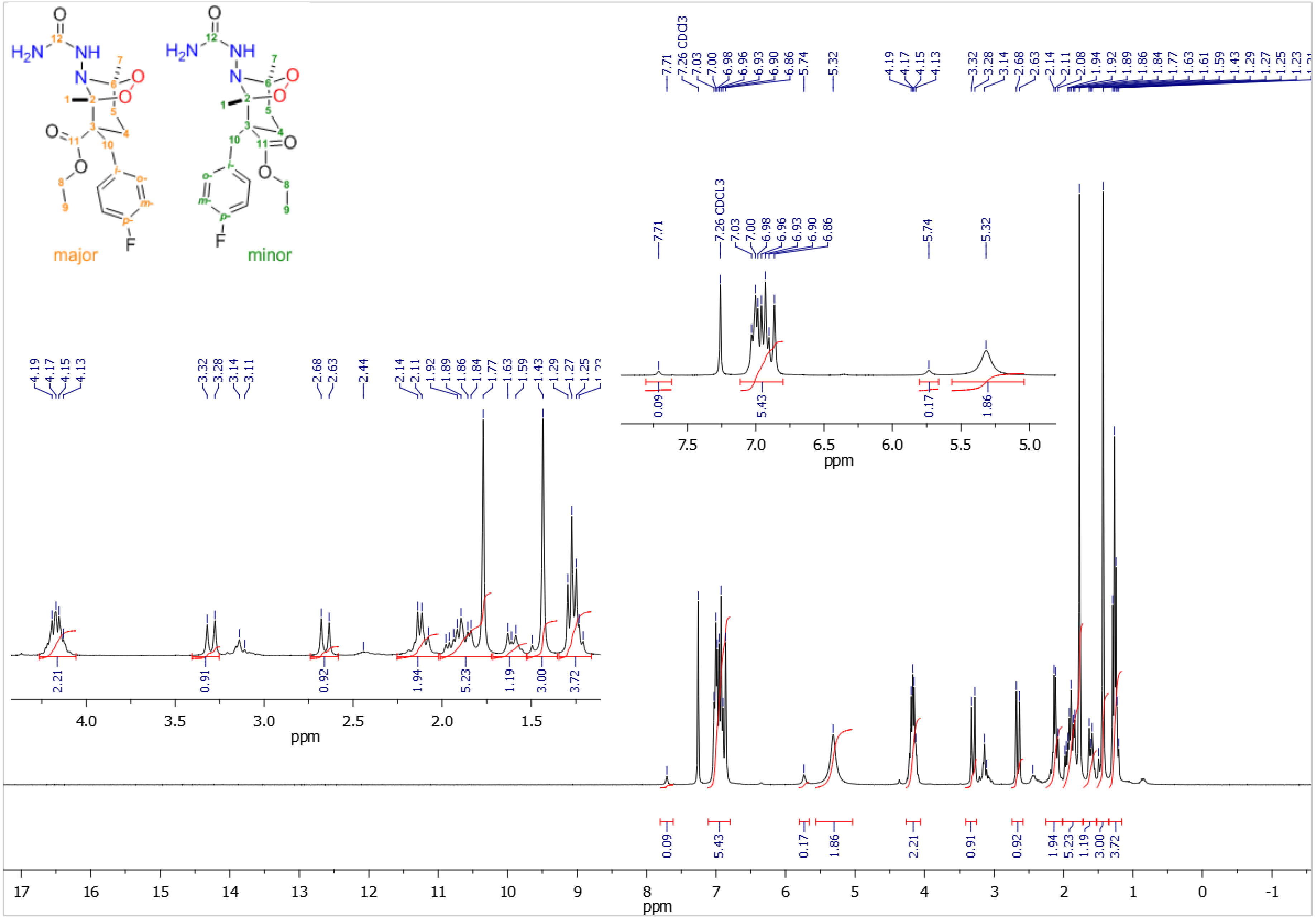

^{1}H NMR (300.13 MHz, $CDCl_3$). Ethyl 2-(4-fluorobenzyl)-1,5-dimethyl-8-ureido-6,7-dioxa-8-azabicyclo[3.2.1]octane-2-carboxylate, 12a + 12b

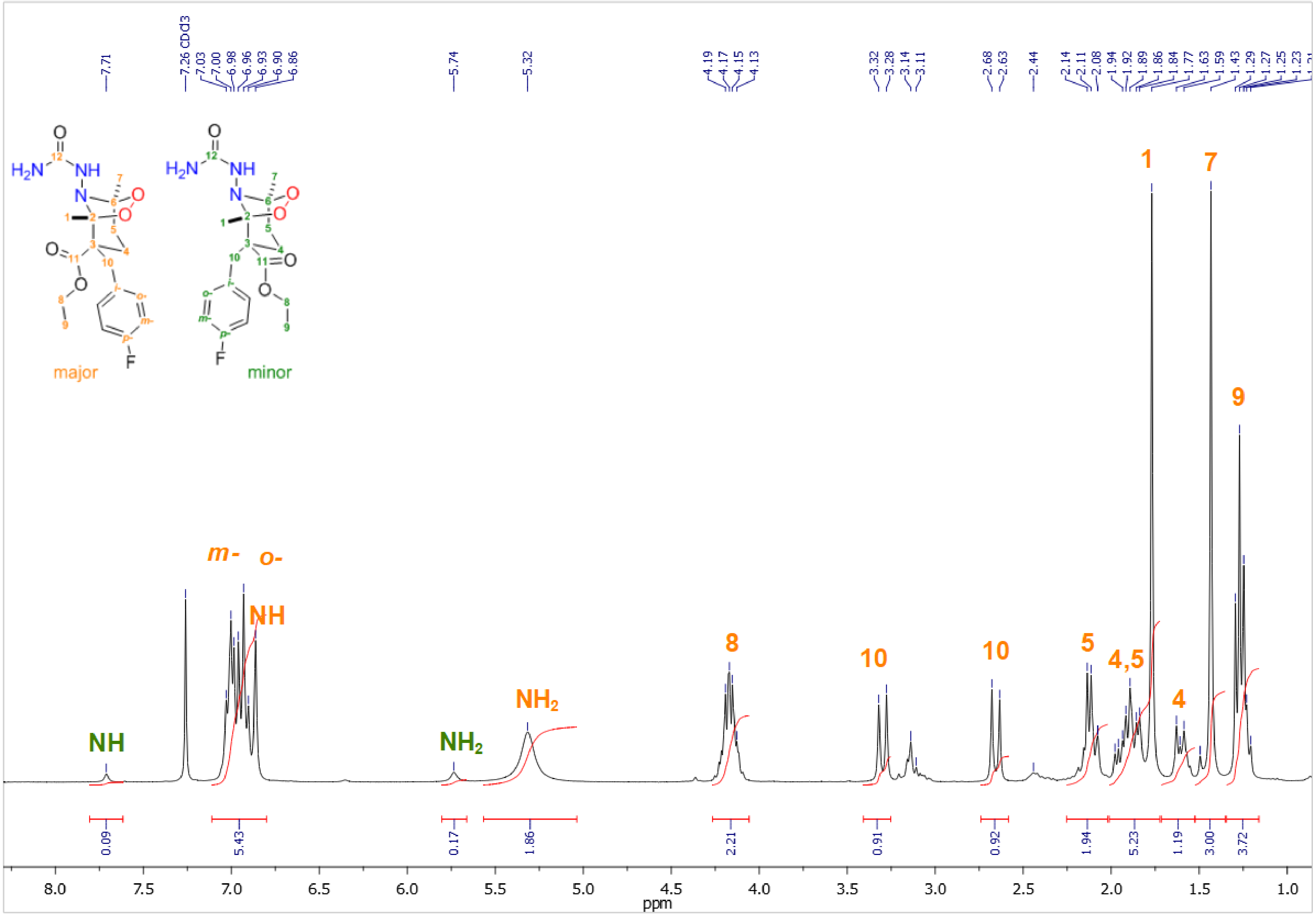

^{13}C NMR (75.48 MHz, CDCl3). Ethyl 2-(4-fluorobenzyl)-1,5-dimethyl-8-ureido-6,7-dioxa-8-azabicyclo[3.2.1]octane-2-carboxylate, 12a + 12b

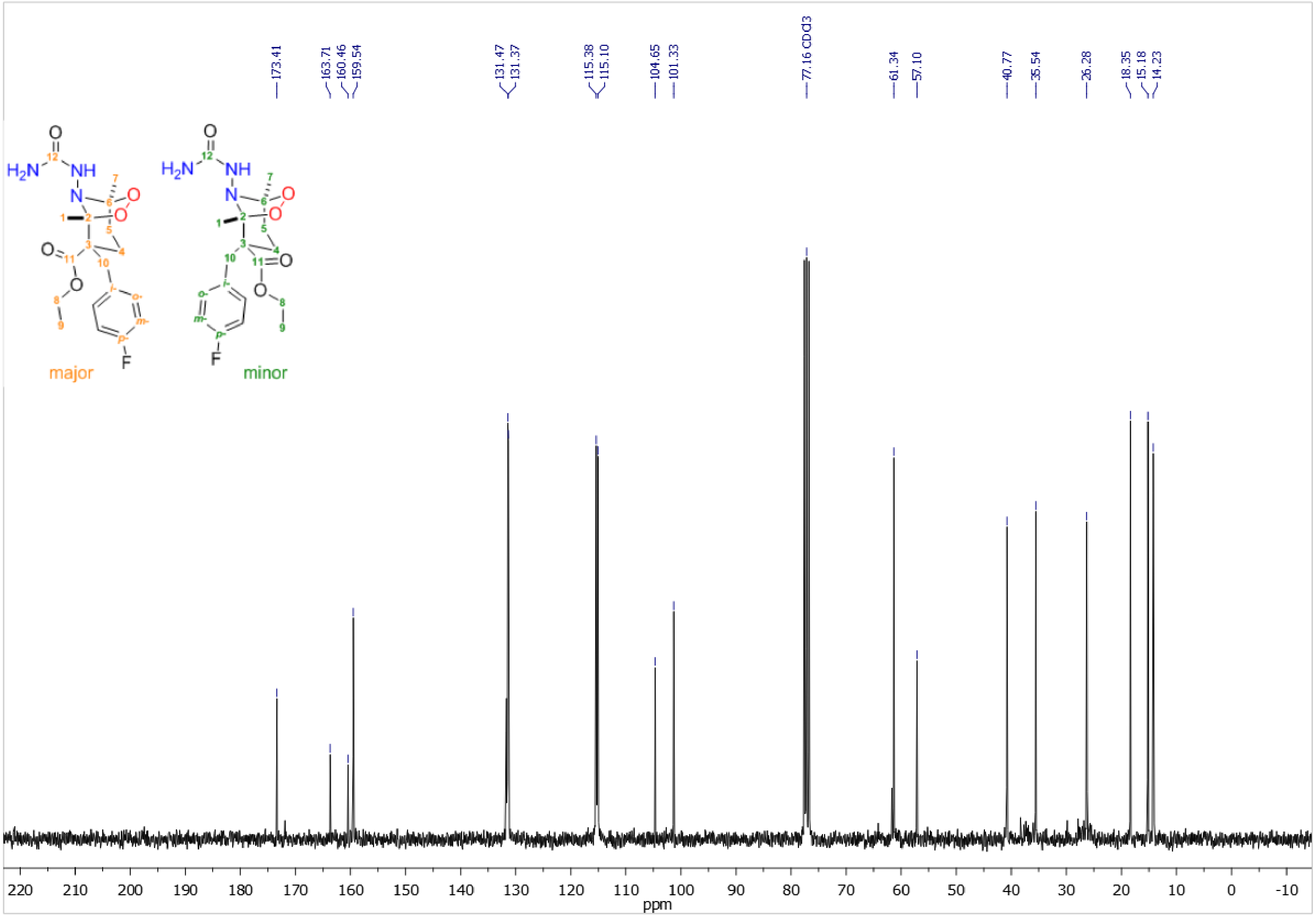

^{13}C NMR (75.48 MHz, CDCl3). Ethyl 2-(4-fluorobenzyl)-1,5-dimethyl-8-ureido-6,7-dioxa-8-azabicyclo[3.2.1]octane-2-carboxylate, 12a + 12b

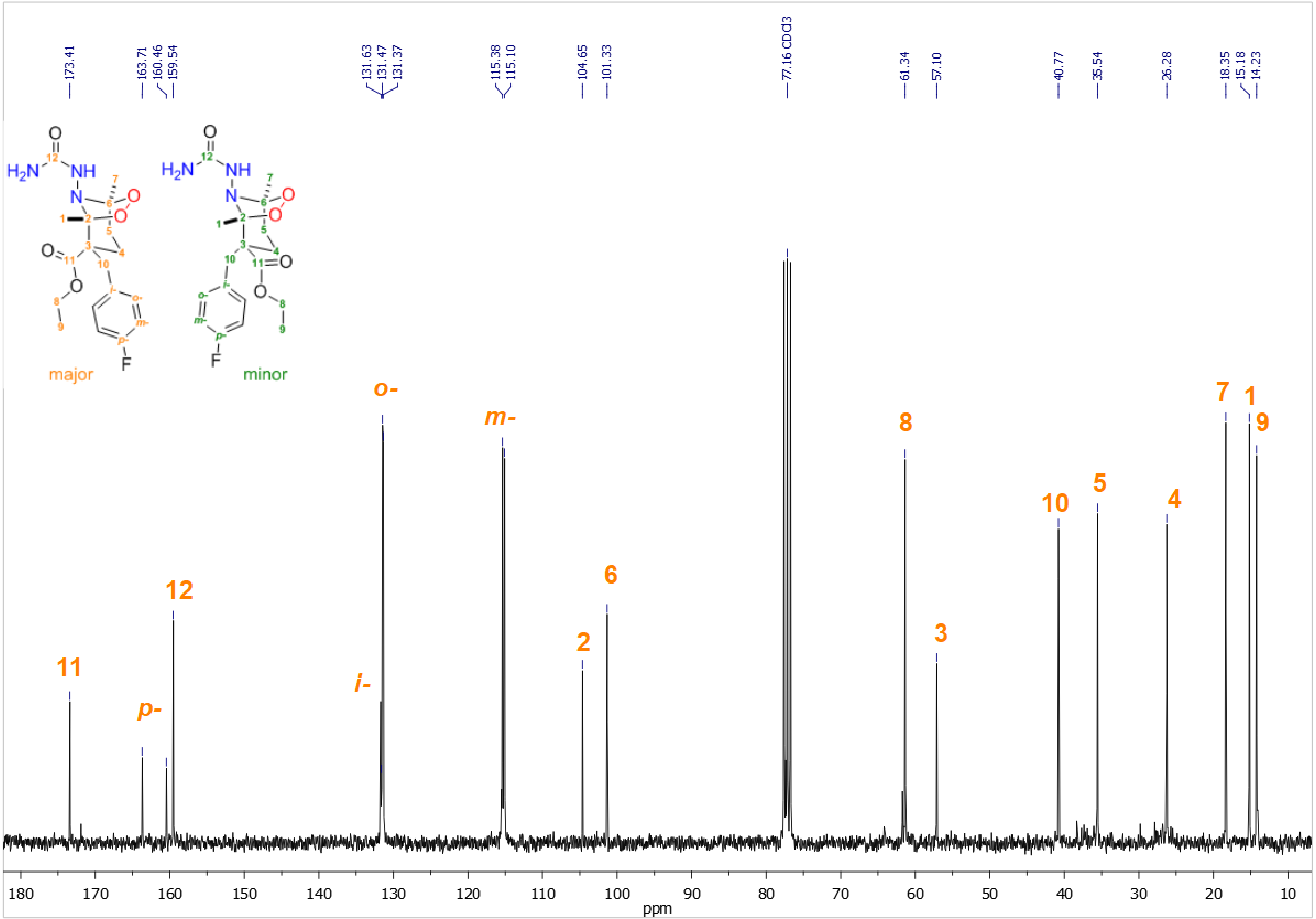

^{15}N (40.56 MHz, $CDCl_3$). Ethyl 2-(4-fluorobenzyl)-1,5-dimethyl-8-ureido-6,7-dioxa-8-azabicyclo[3.2.1]octane-2-carboxylate, 12a + 12b

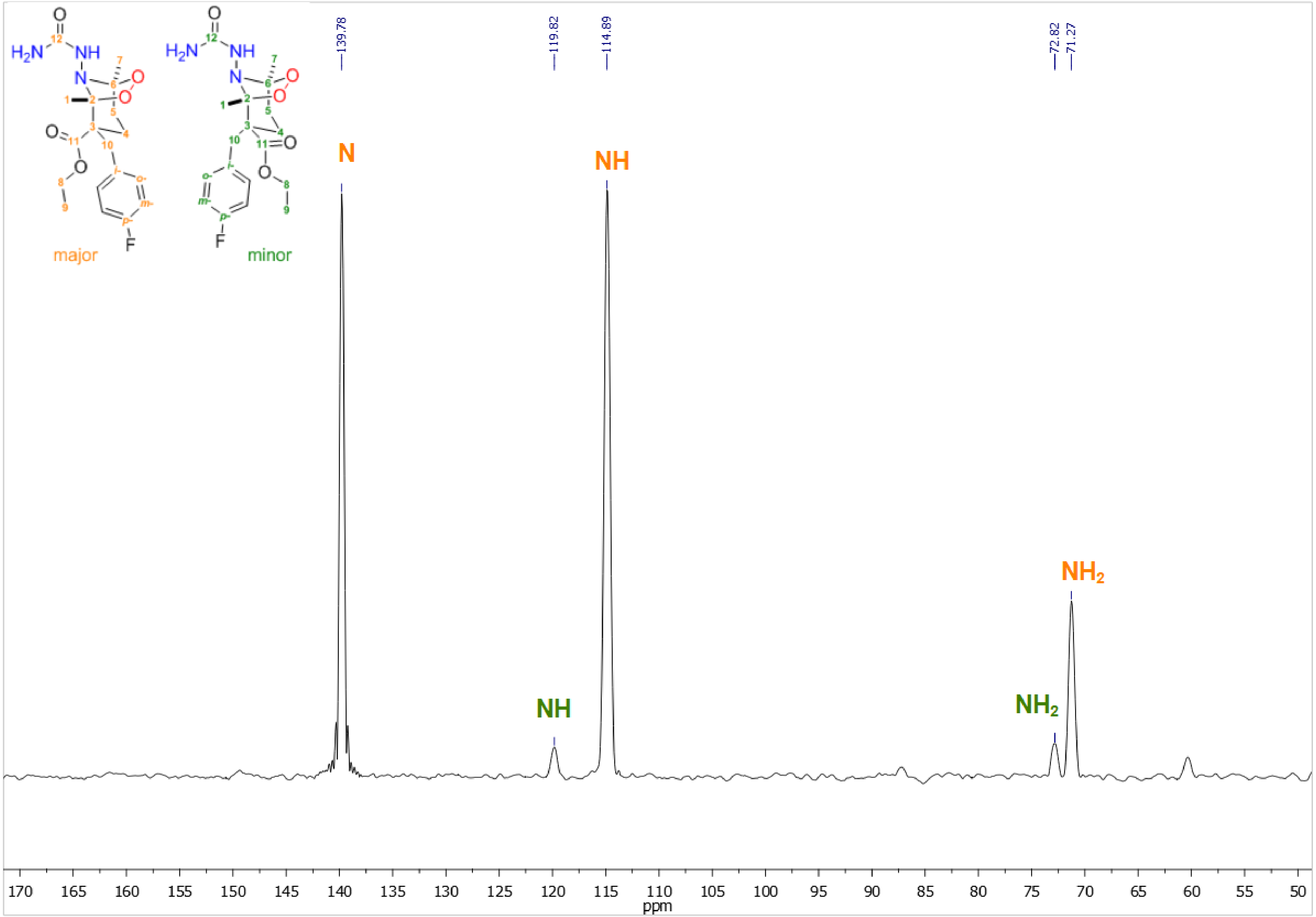

^{13}C NMR (75.48 MHz, $CDCl_3$). Ethyl 2-(4-fluorobenzyl)-1,5-dimethyl-8-ureido-6,7-dioxa-8-azabicyclo[3.2.1]octane-2-carboxylate, 12a + 12b

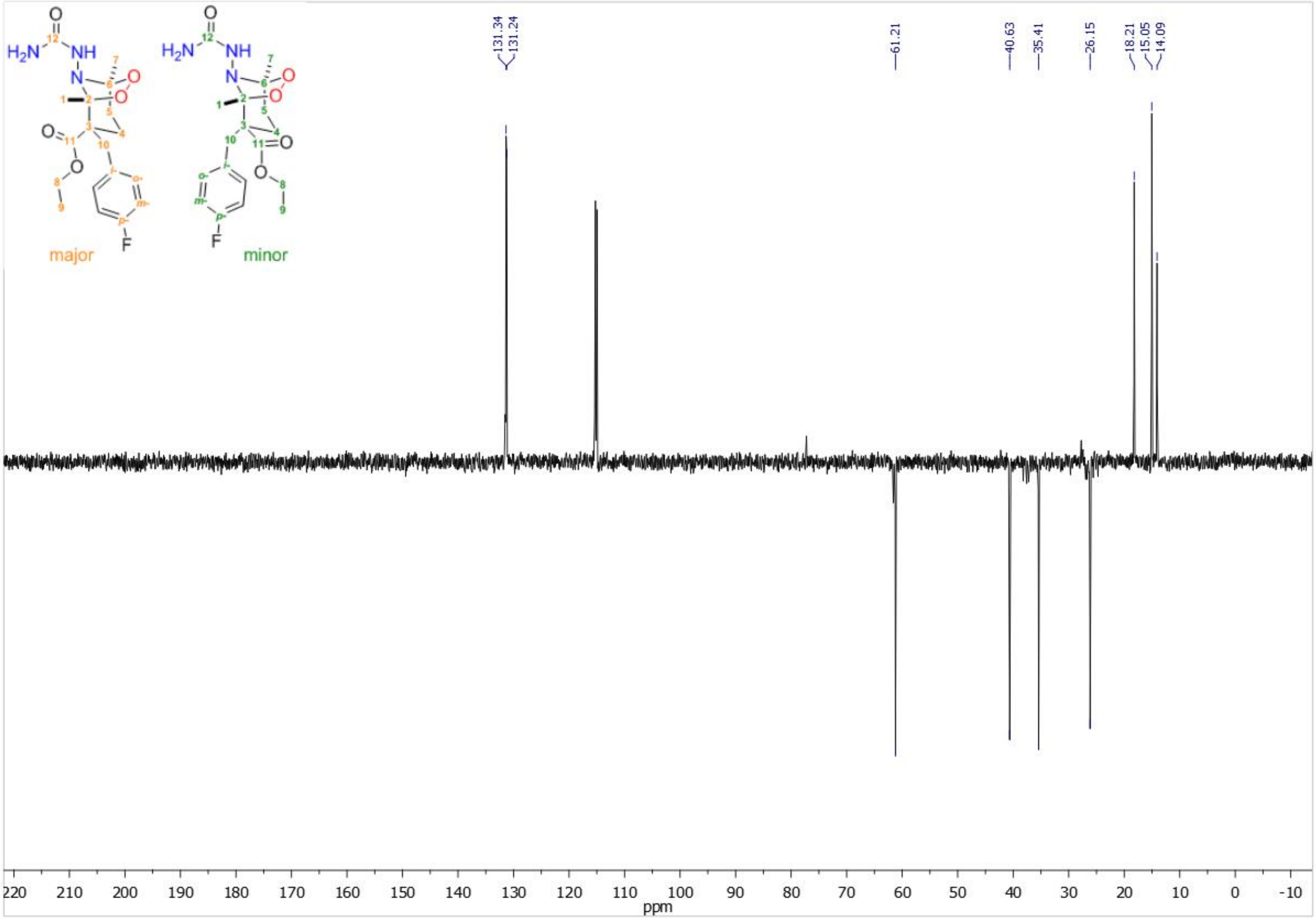

Ethyl 2-(4-fluorobenzyl)-1,5-dimethyl-8-ureido-6,7-dioxa-8-azabicyclo[3.2.1]octane-2-carboxylate, 12a + 12b

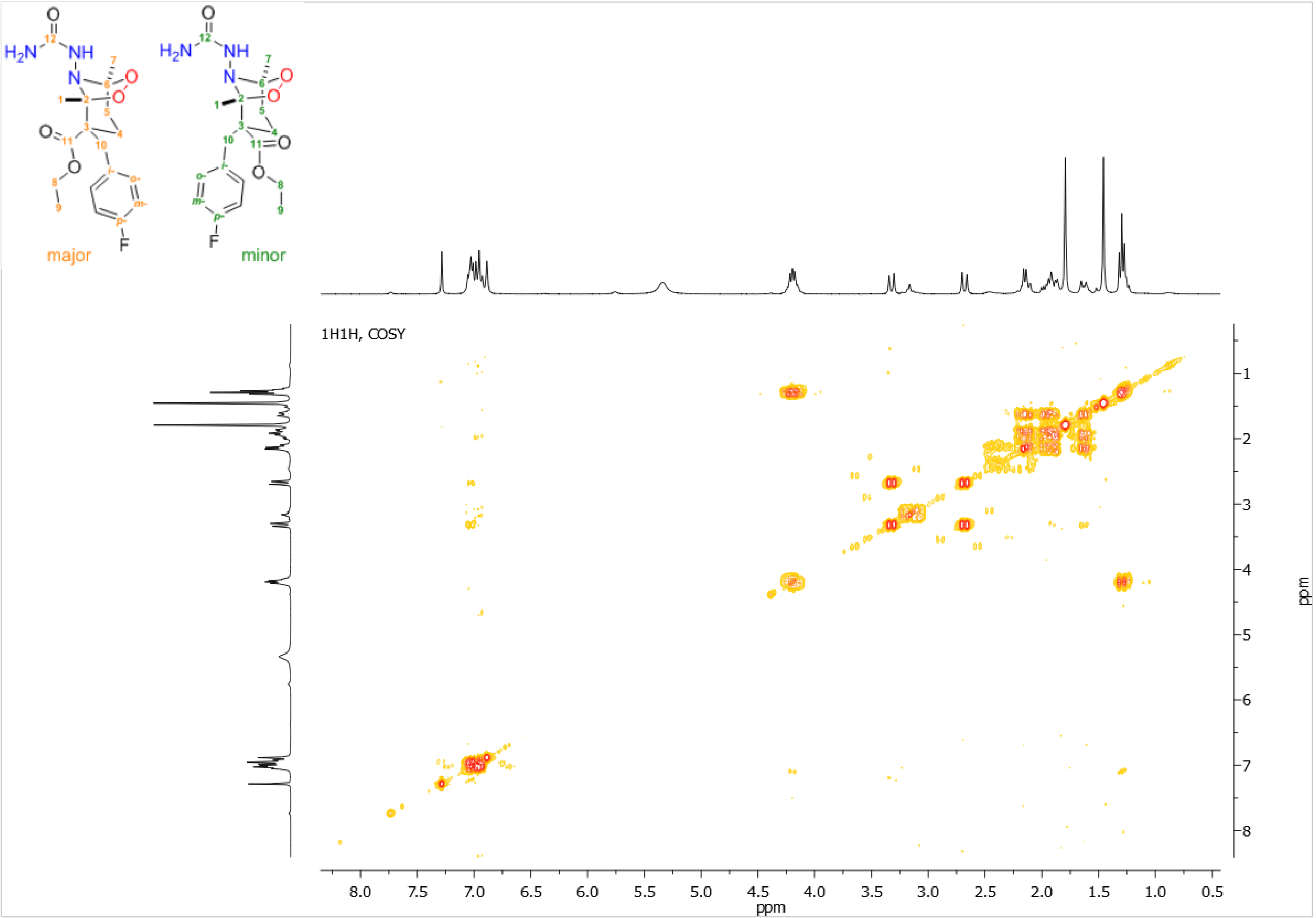

Ethyl 2-(4-fluorobenzyl)-1,5-dimethyl-8-ureido-6,7-dioxa-8-azabicyclo[3.2.1]octane-2-carboxylate, 12a + 12b

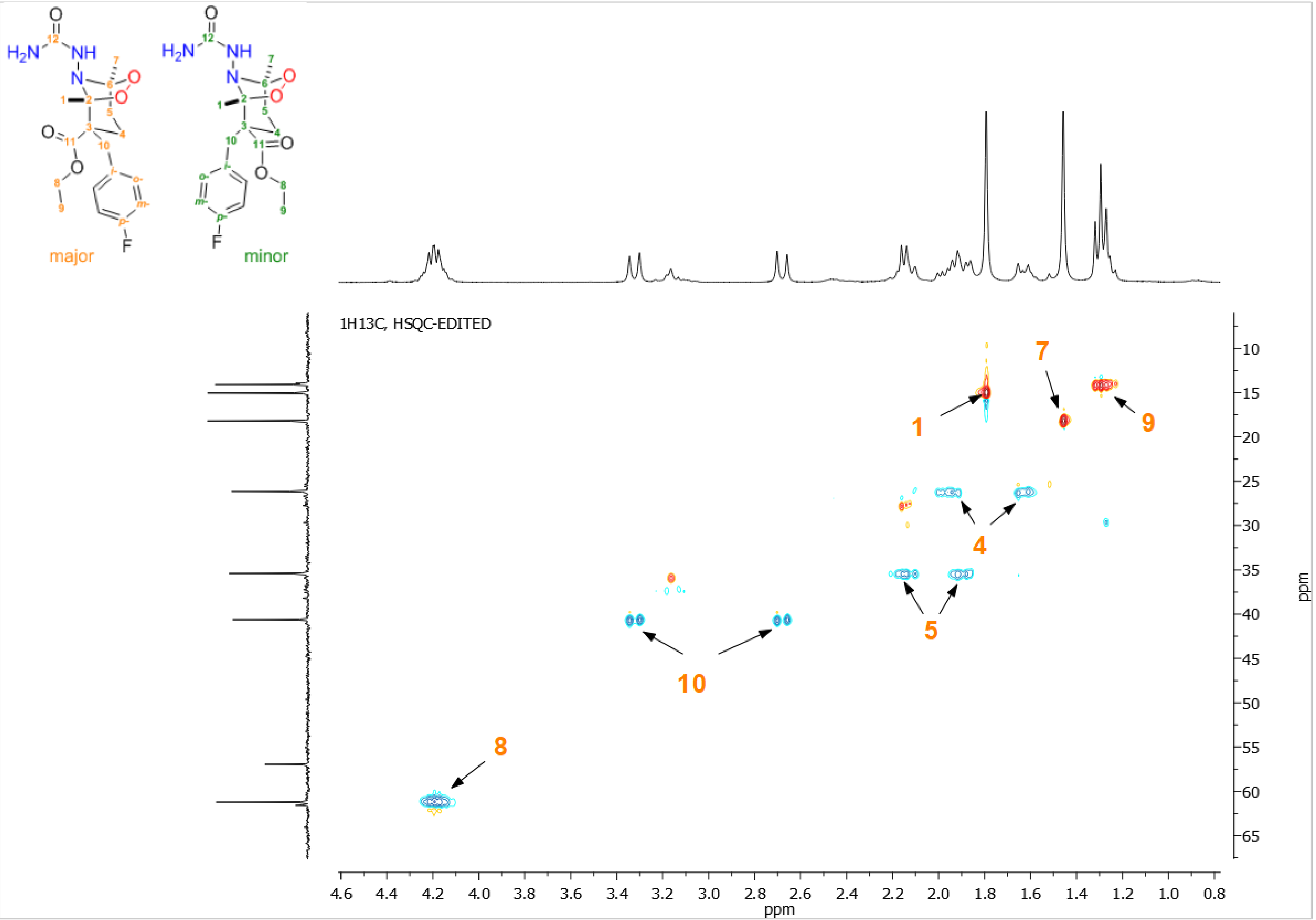

Ethyl 2-(4-fluorobenzyl)-1,5-dimethyl-8-ureido-6,7-dioxa-8-azabicyclo[3.2.1]octane-2-carboxylate, 12a + 12b

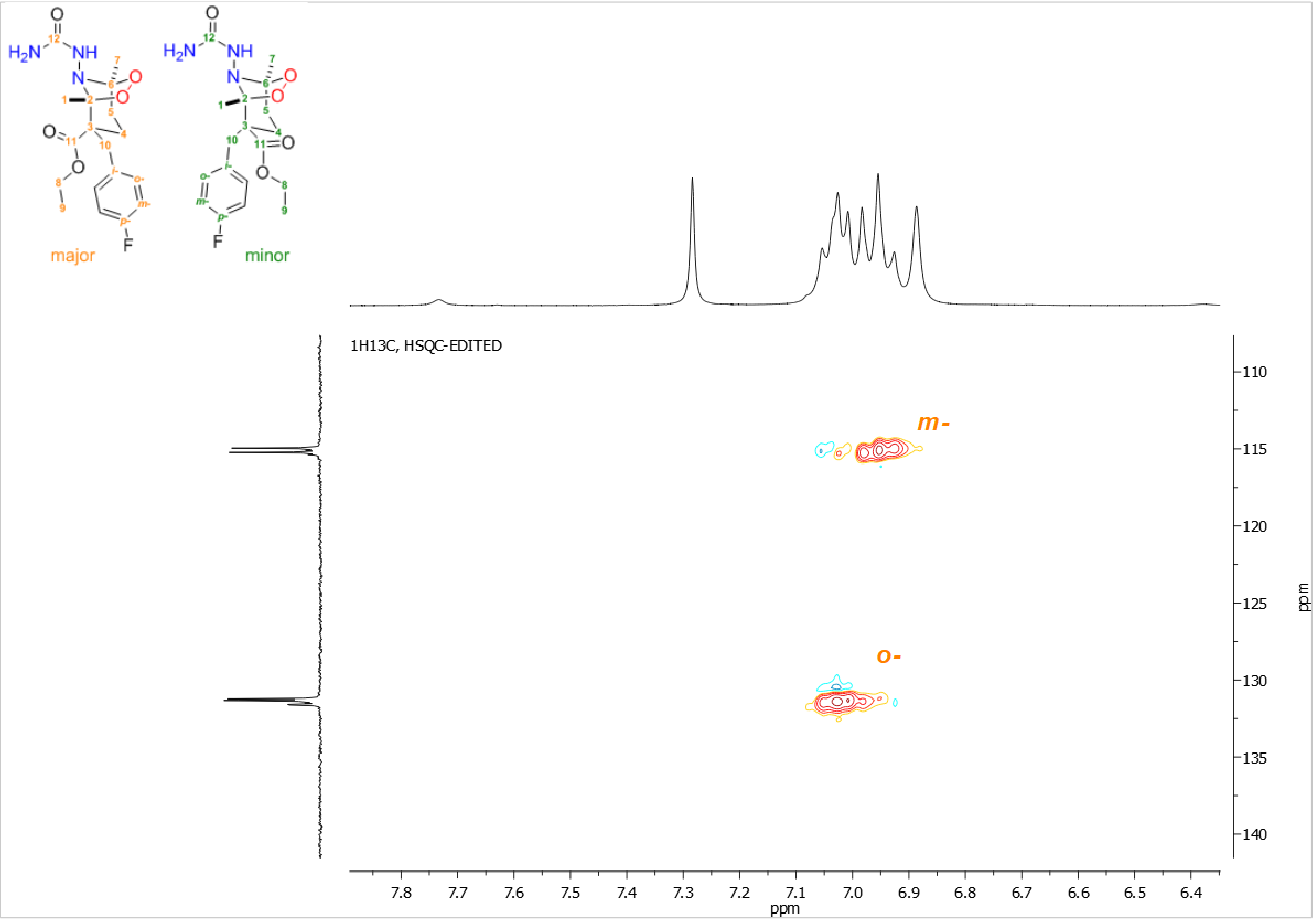

Ethyl 2-(4-fluorobenzyl)-1,5-dimethyl-8-ureido-6,7-dioxa-8-azabicyclo[3.2.1]octane-2-carboxylate, 12a + 12b

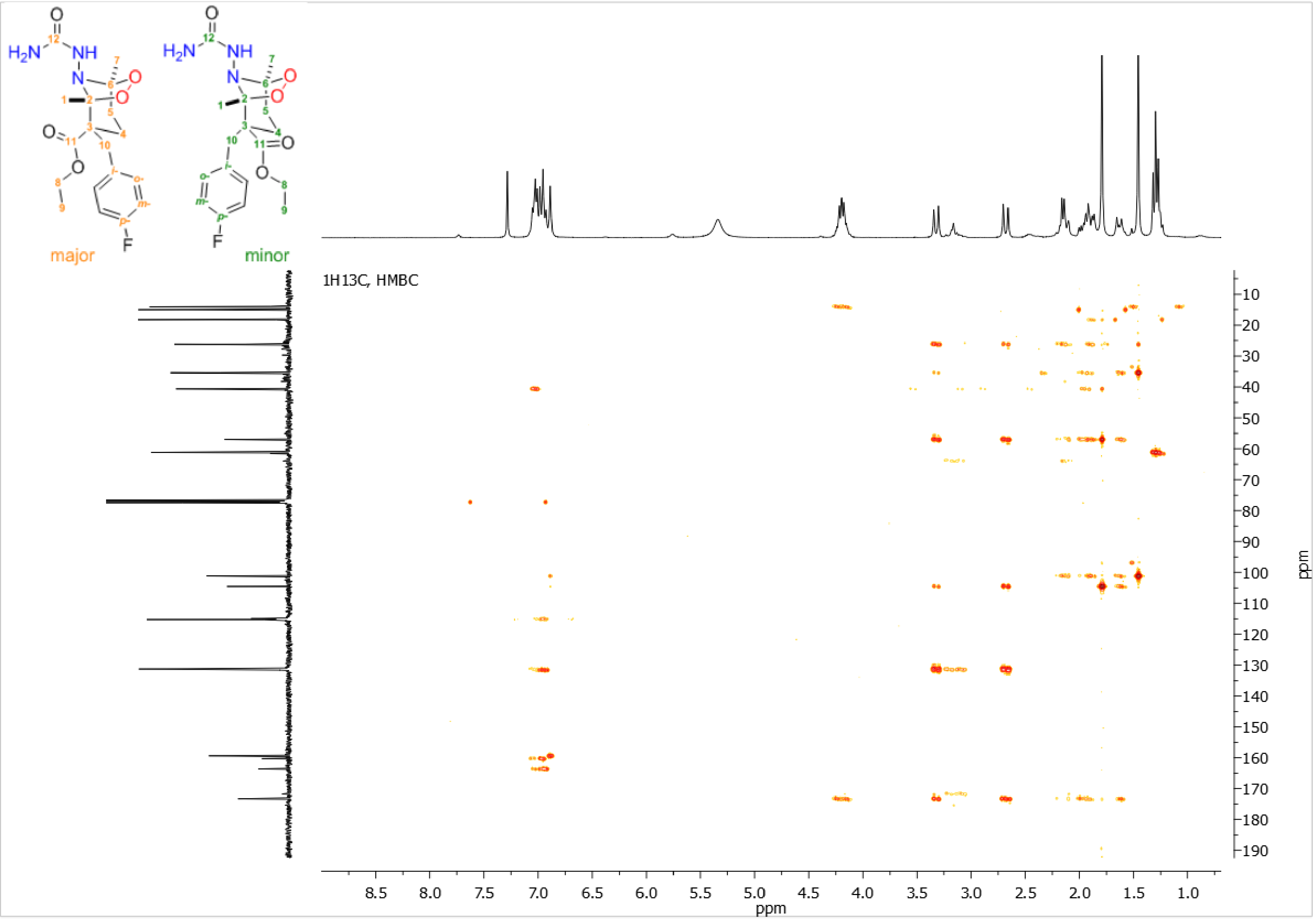

Ethyl 2-(4-fluorobenzyl)-1,5-dimethyl-8-ureido-6,7-dioxa-8-azabicyclo[3.2.1]octane-2-carboxylate, 12a + 12b

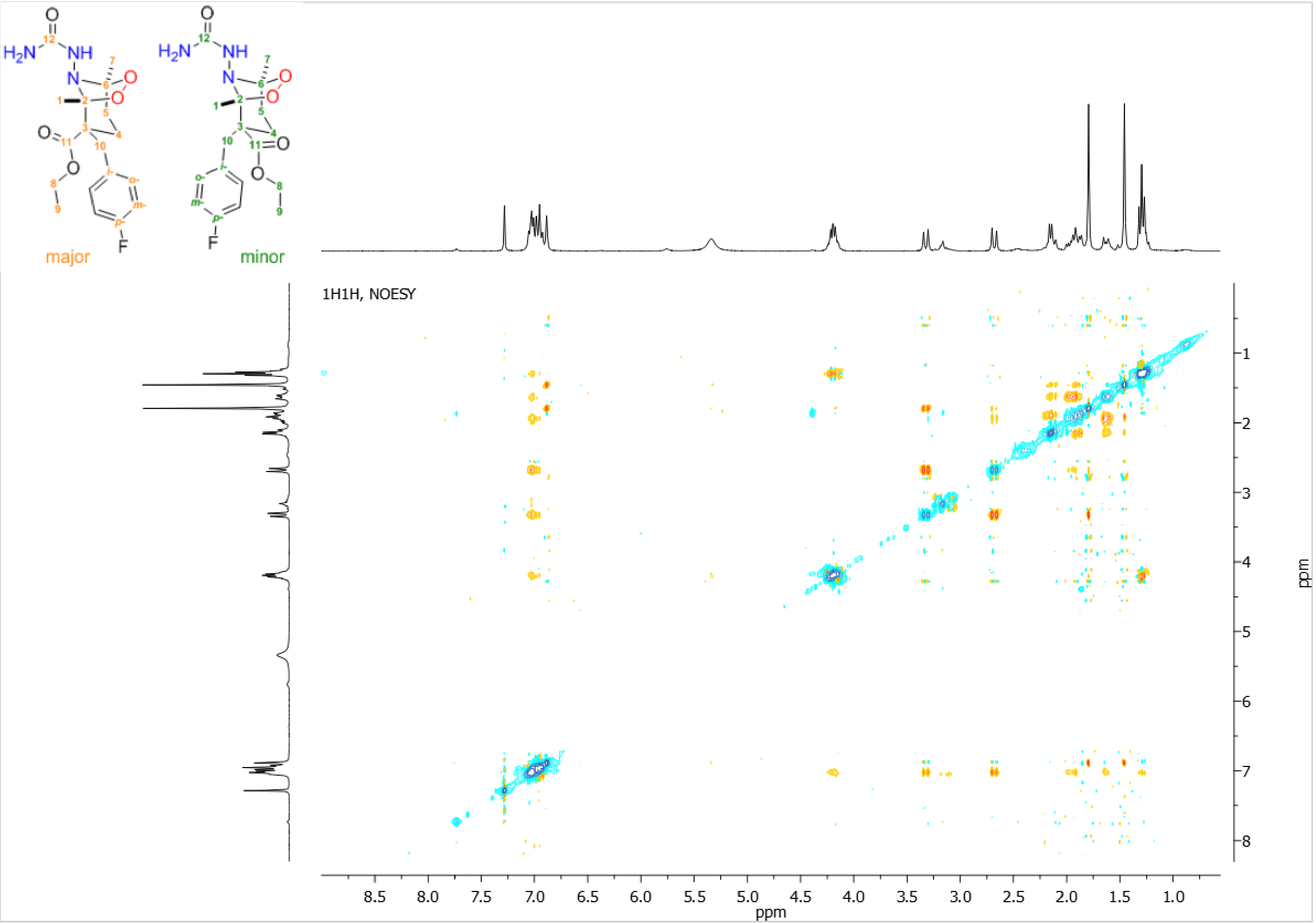

Ethyl 2-(4-fluorobenzyl)-1,5-dimethyl-8-ureido-6,7-dioxa-8-azabicyclo[3.2.1]octane-2-carboxylate, 12a + 12b

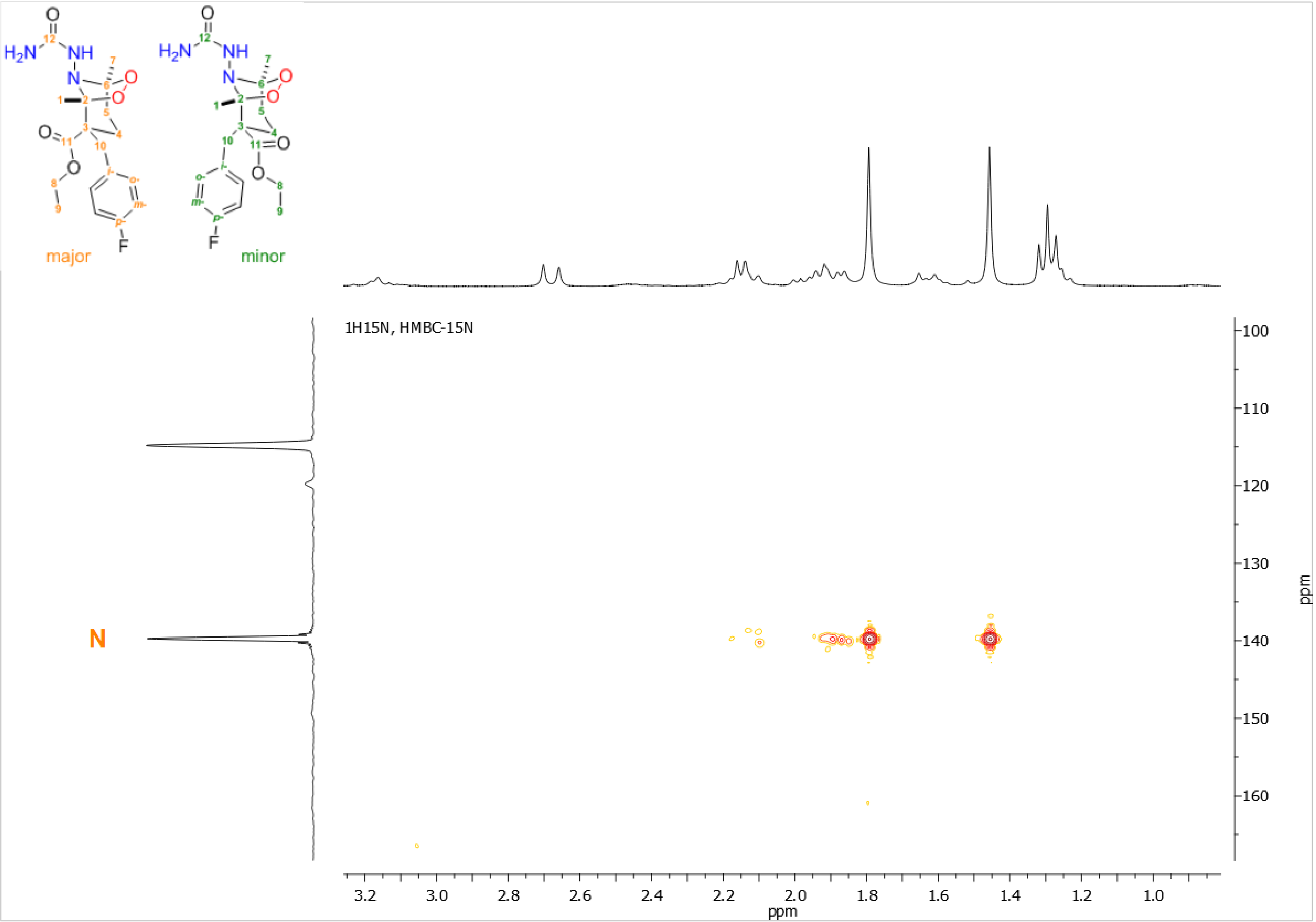

Ethyl 2-(4-fluorobenzyl)-1,5-dimethyl-8-ureido-6,7-dioxa-8-azabicyclo[3.2.1]octane-2-carboxylate, 12a + 12b

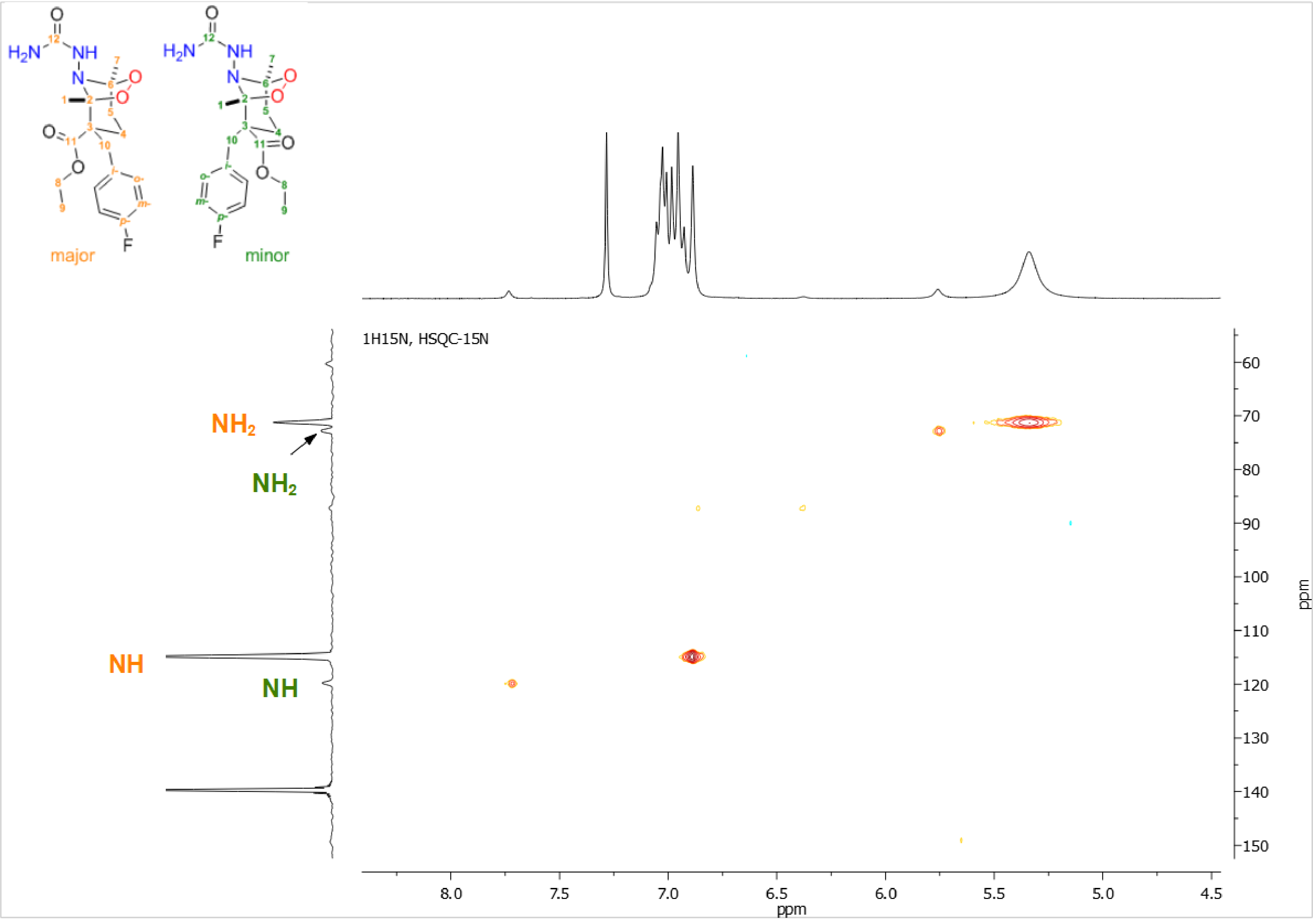

^{1}H NMR (300.13 MHz, $CDCl_3$). Allyl 2-allyl-1,5-dimethyl-8-ureido-6,7-dioxa-8-azabicyclo[3.2.1]octane-2-carboxylate, 13a

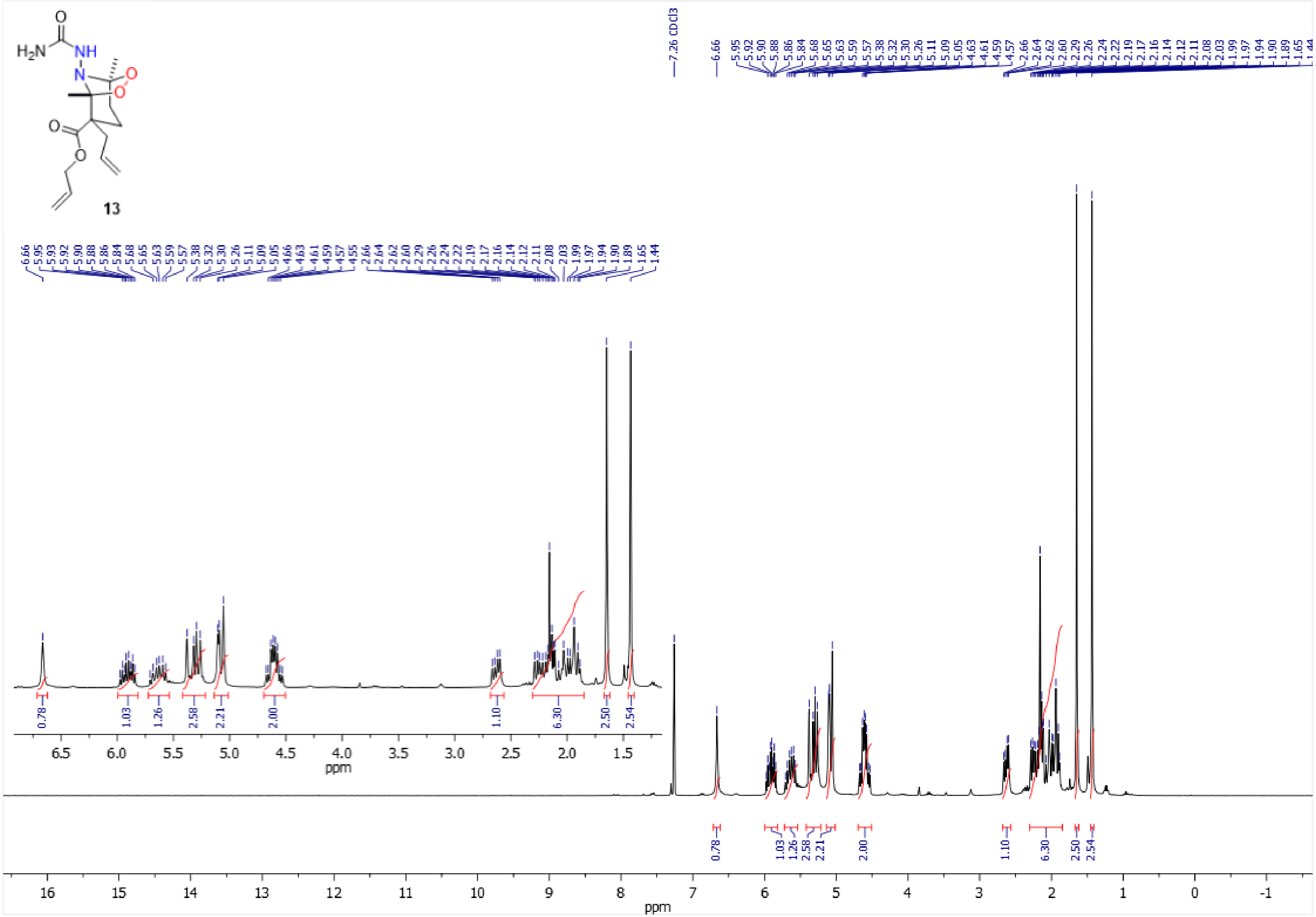

^{13}C NMR (75.48 MHz, CDCl$_3$). Allyl 2-allyl-1,5-dimethyl-8-ureido-6,7-dioxa-8-azabicyclo[3.2.1]octane-2-carboxylate, 13a

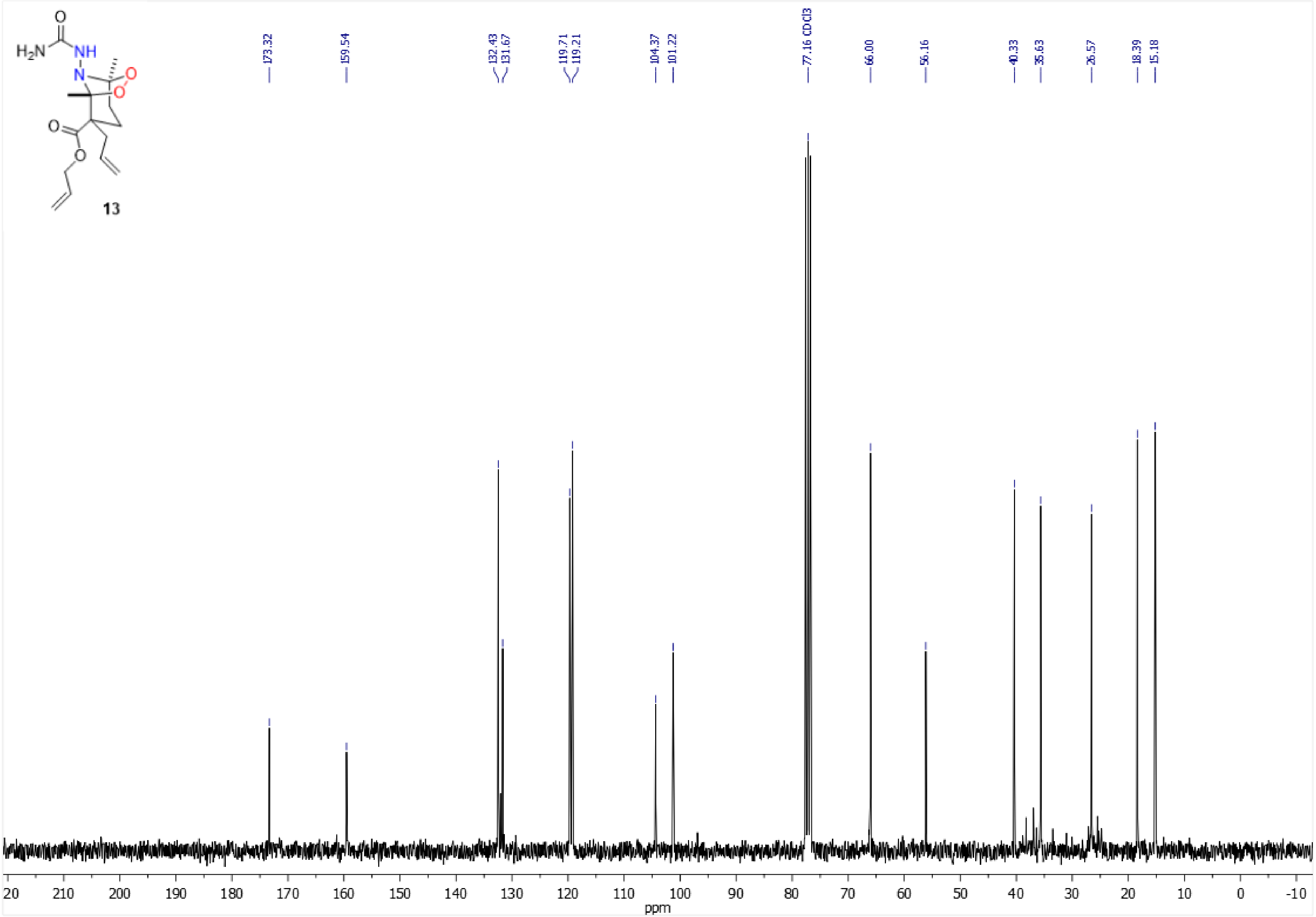

^{1}H NMR (300.13 MHz, CDCl3). Ethyl 2-benzyl-1,5-dimethyl-8-ureido-6,7-dioxa-8-azabicyclo[3.2.1]octane-2-carboxylate, 14a

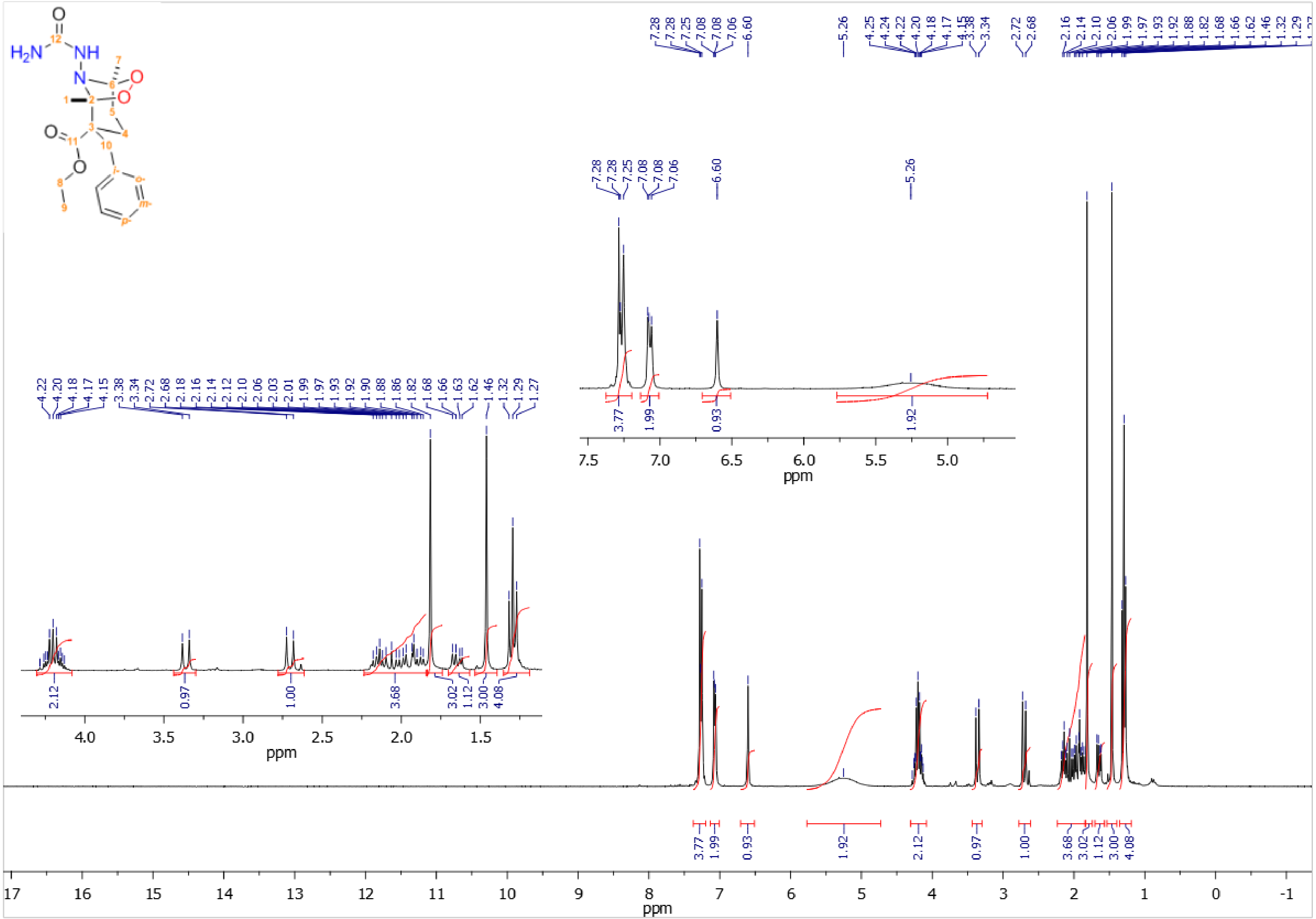

^{1}H NMR (300.13 MHz, CDCl3). Ethyl 2-benzyl-1,5-dimethyl-8-ureido-6,7-dioxa-8-azabicyclo[3.2.1]octane-2-carboxylate, 14a

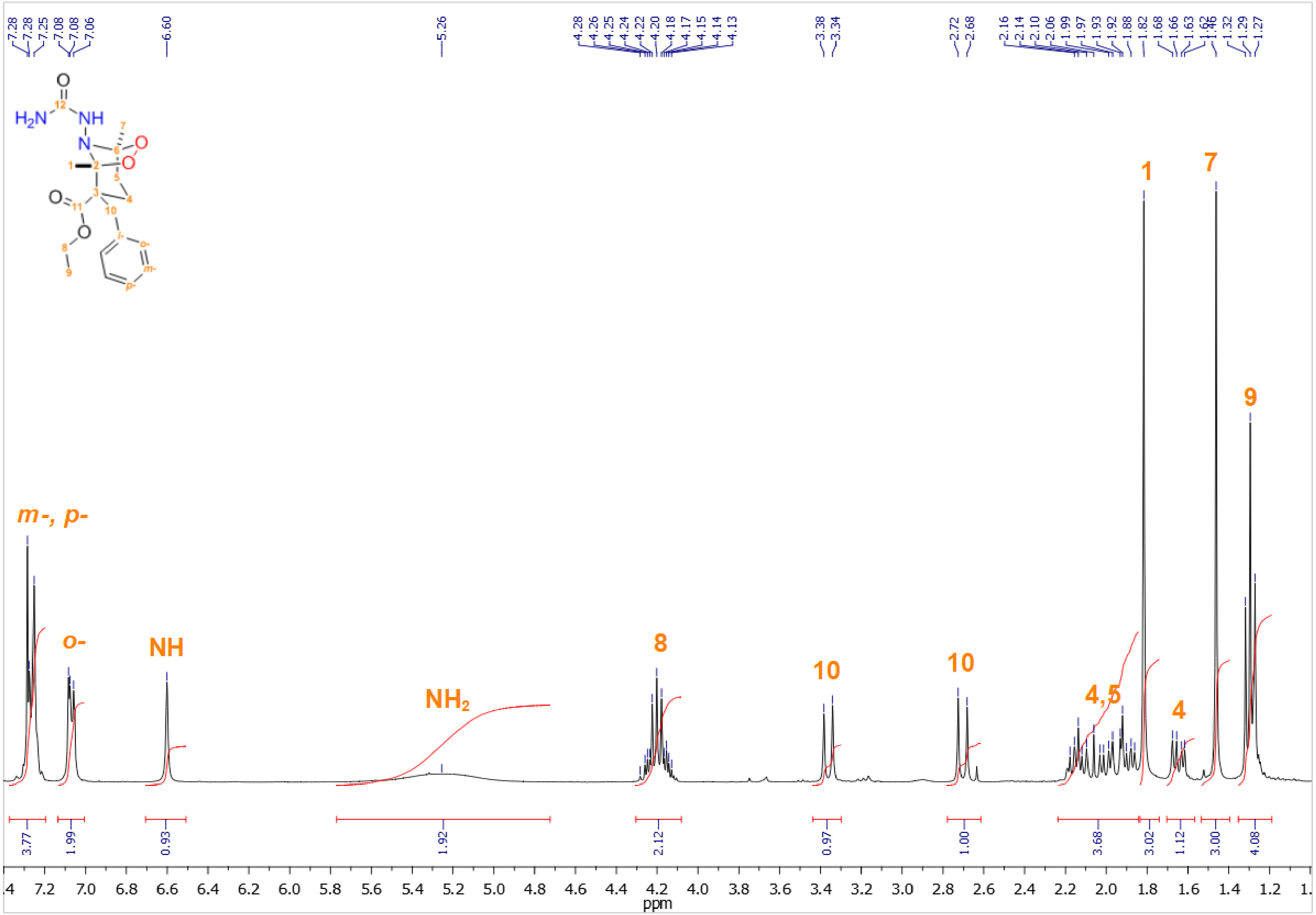

^{13}C NMR (75.48 MHz, CDCl$_3$).Ethyl 2-benzyl-1,5-dimethyl-8-ureido-6,7-dioxa-8-azabicyclo[3.2.1]octane-2-carboxylate, 14a

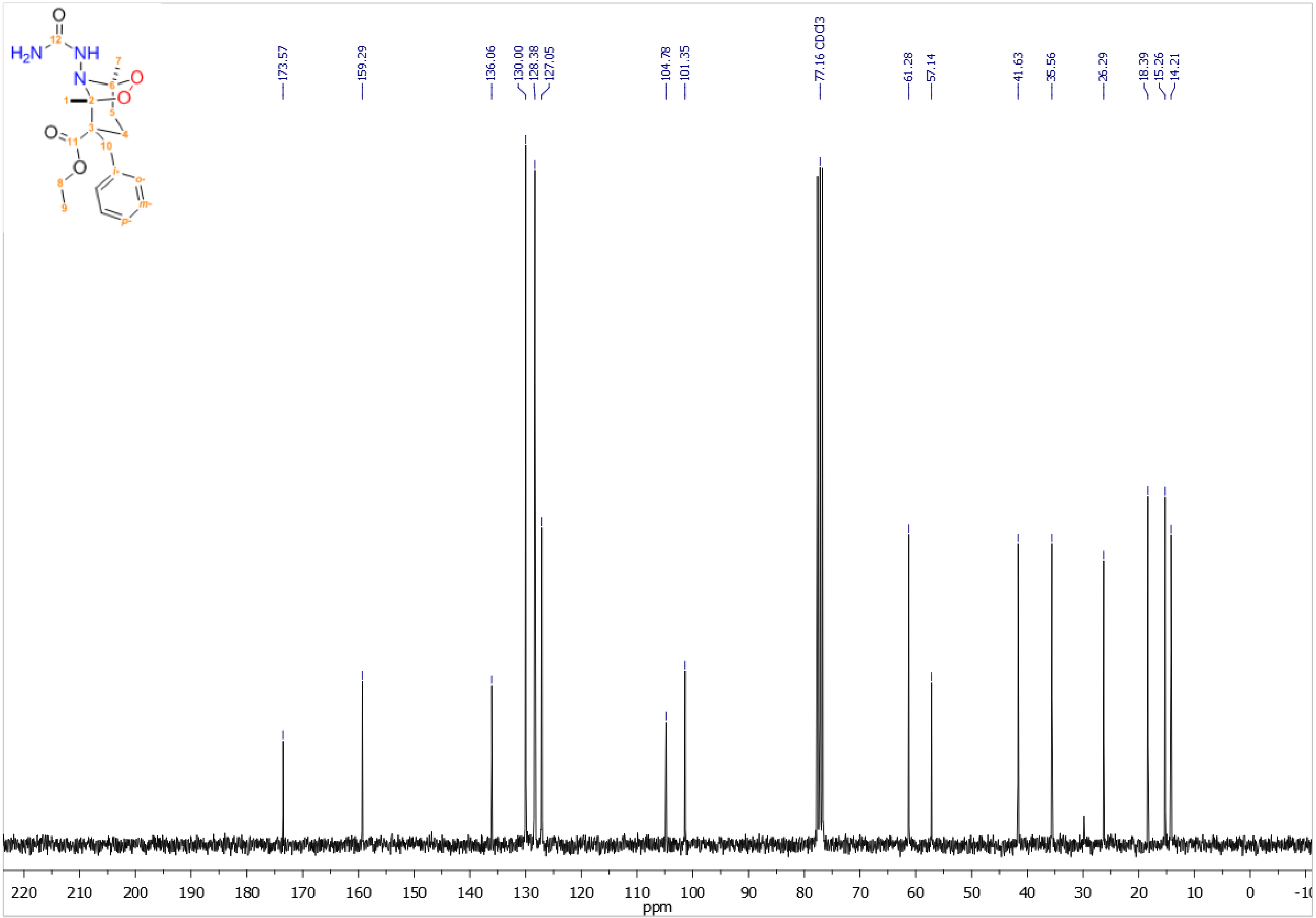

^{13}C NMR (75.48 MHz, $CDCl_3$).Ethyl 2-benzyl-1,5-dimethyl-8-ureido-6,7-dioxa-8-azabicyclo[3.2.1]octane-2-carboxylate, 14a

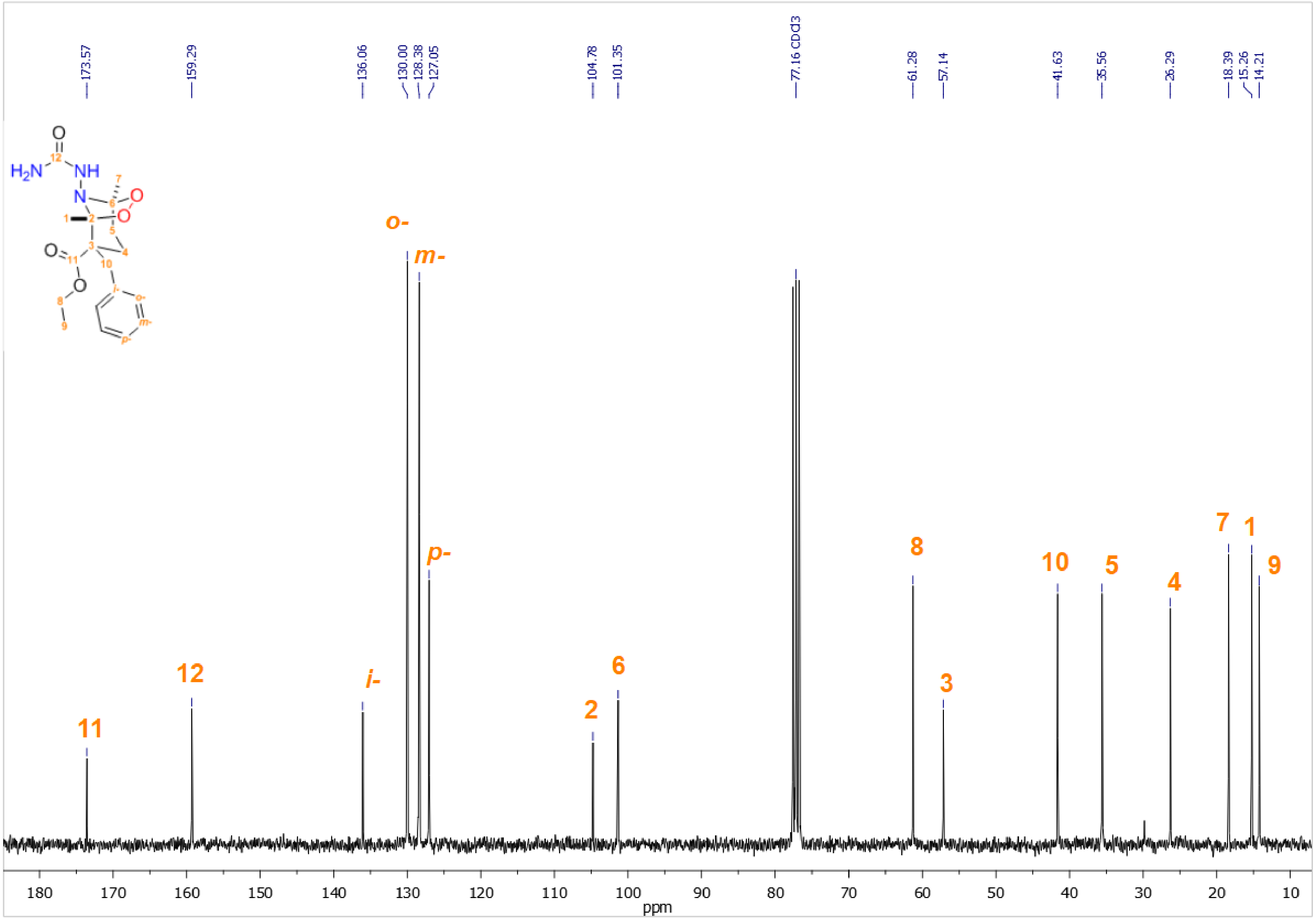

^{15}N (40.56 MHz, $CDCl_3$). Ethyl 2-benzyl-1,5-dimethyl-8-ureido-6,7-dioxa-8-azabicyclo[3.2.1]octane-2-carboxylate, 14a

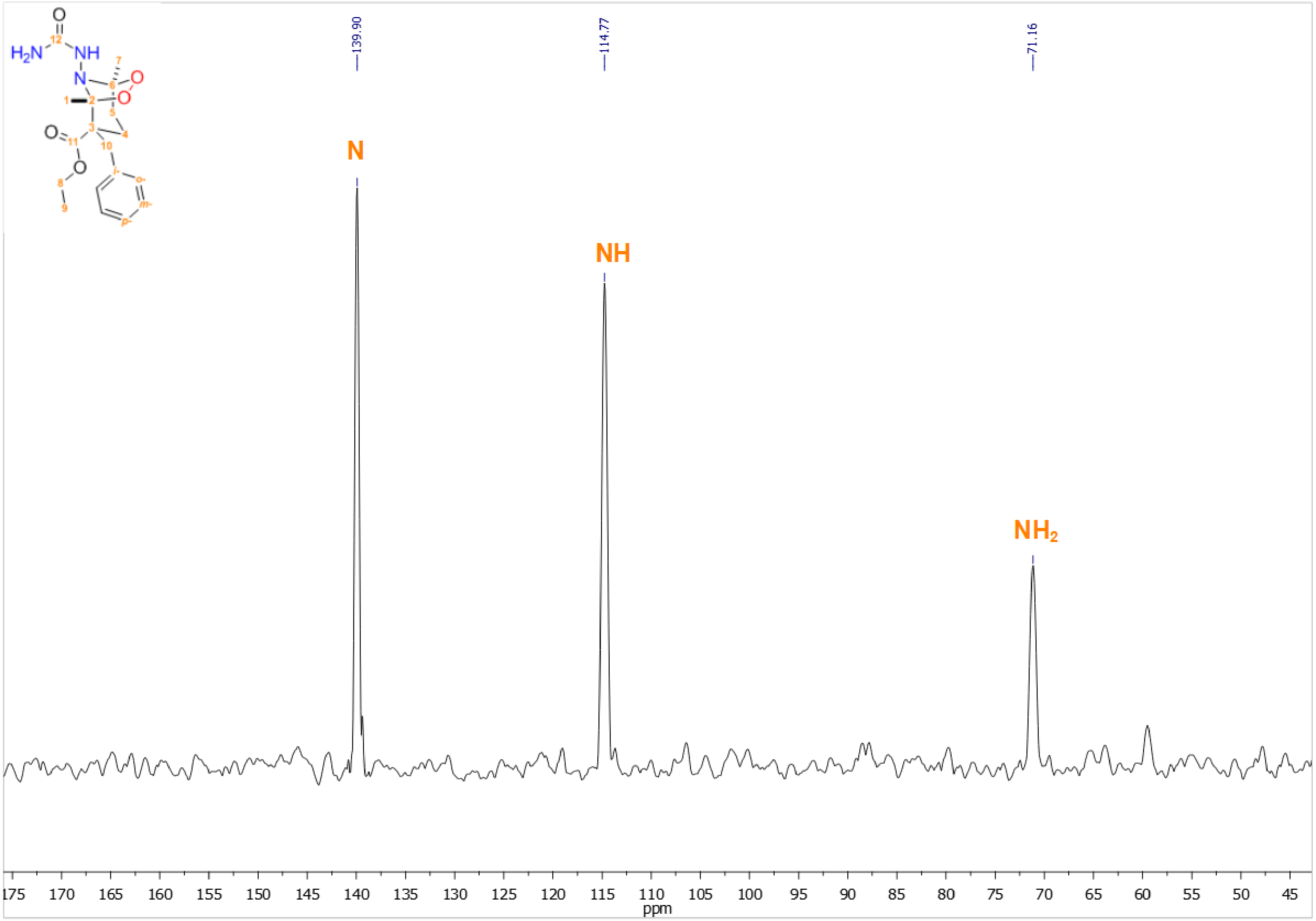

^{13}C NMR (75.48 MHz, $CDCl_3$).Ethyl 2-benzyl-1,5-dimethyl-8-ureido-6,7-dioxa-8-azabicyclo[3.2.1]octane-2-carboxylate, 14a

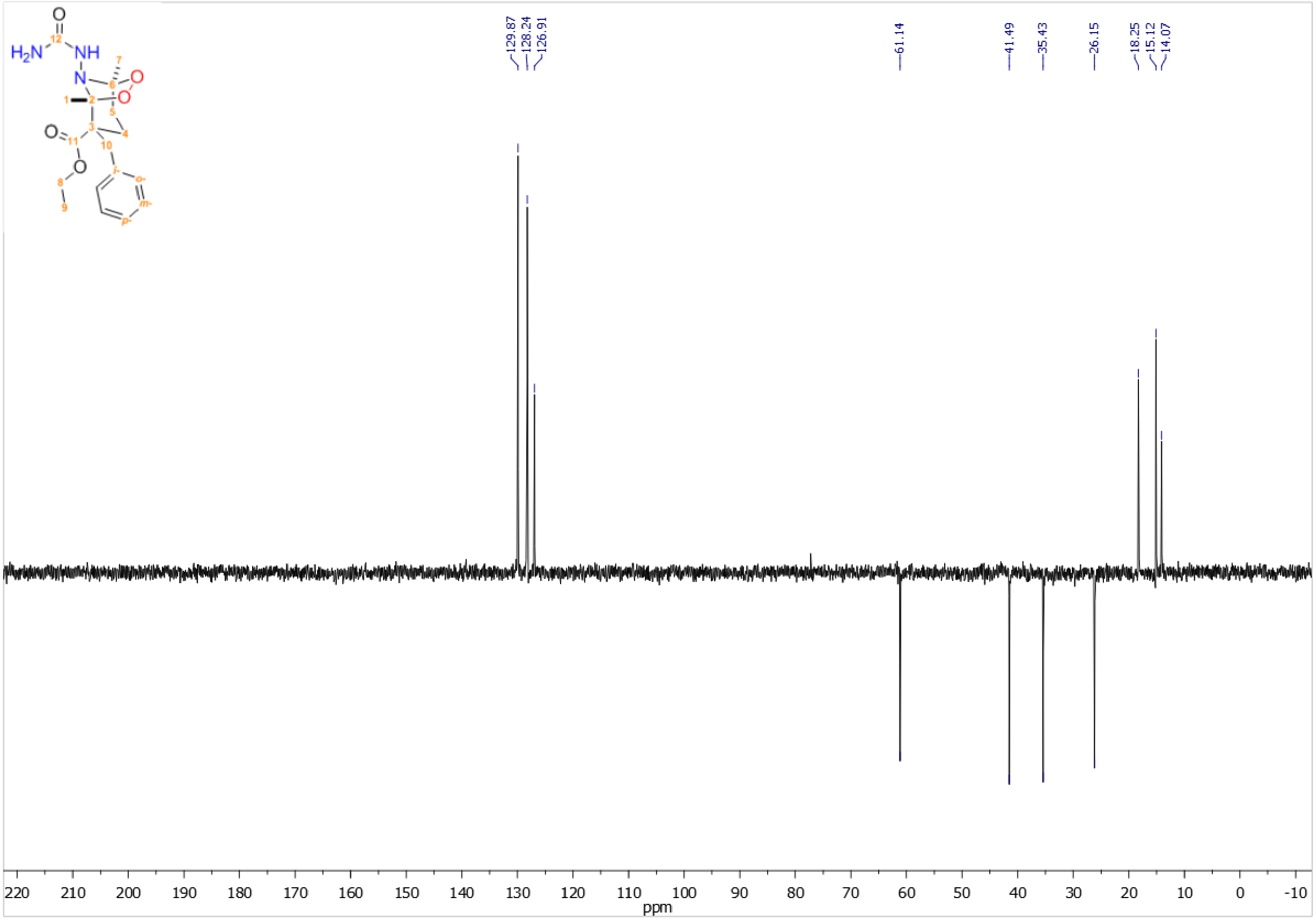

Ethyl 2-benzyl-1,5-dimethyl-8-ureido-6,7-dioxa-8-azabicyclo[3.2.1]octane-2-carboxylate, 14a

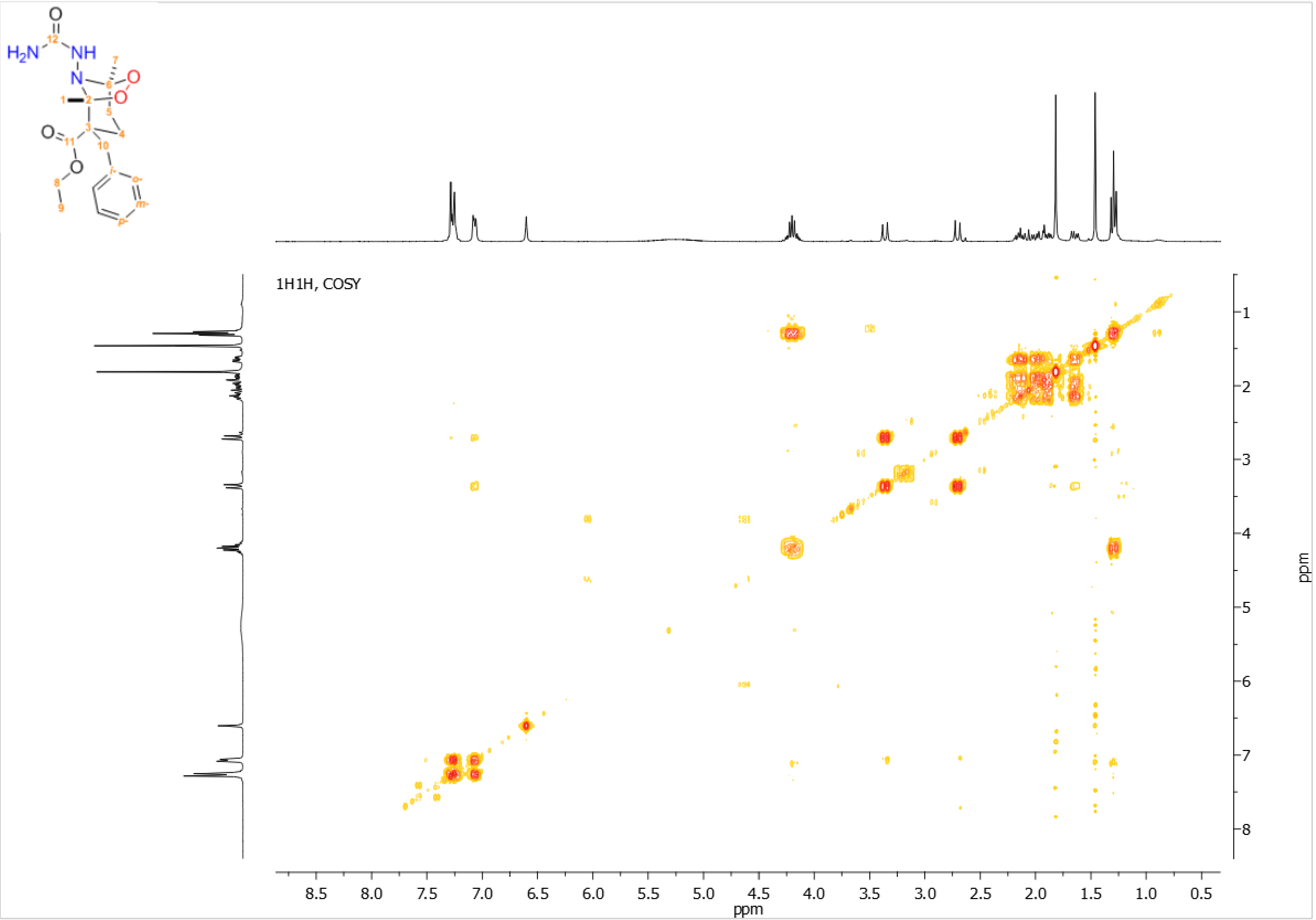

Ethyl 2-benzyl-1,5-dimethyl-8-ureido-6,7-dioxa-8-azabicyclo[3.2.1]octane-2-carboxylate, 14a

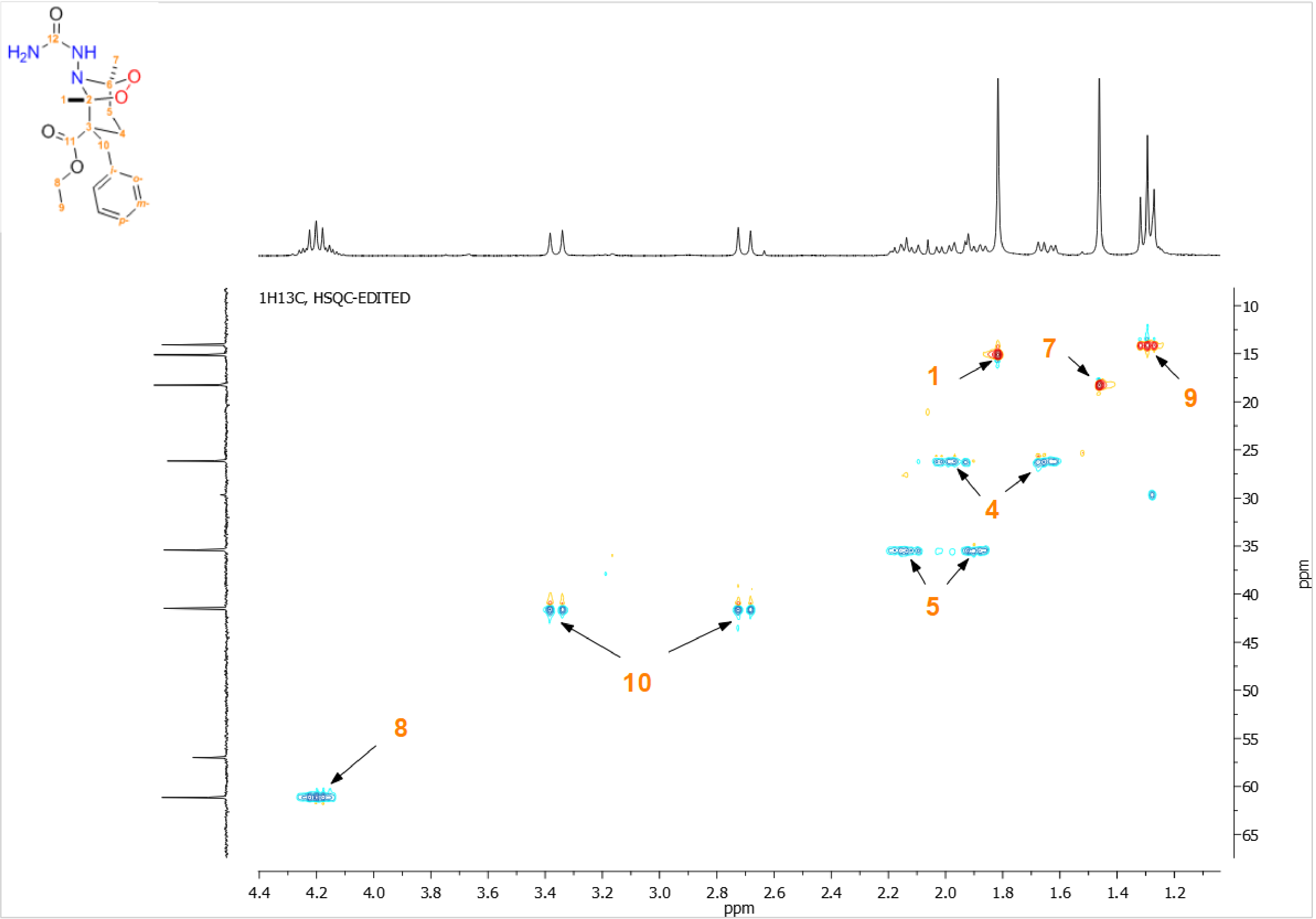

Ethyl 2-benzyl-1,5-dimethyl-8-ureido-6,7-dioxa-8-azabicyclo[3.2.1]octane-2-carboxylate, 14a

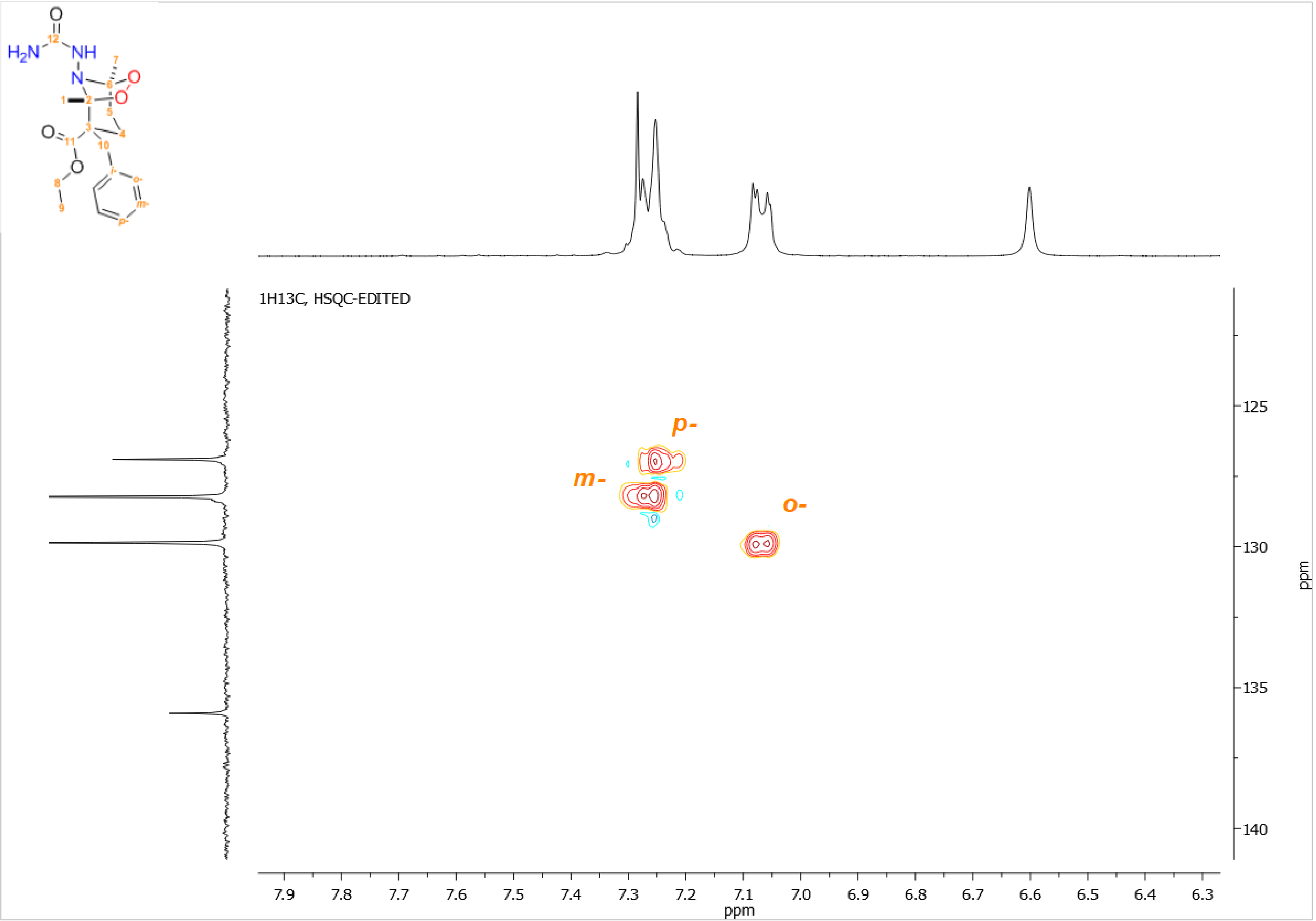

Ethyl 2-benzyl-1,5-dimethyl-8-ureido-6,7-dioxa-8-azabicyclo[3.2.1]octane-2-carboxylate, 14a

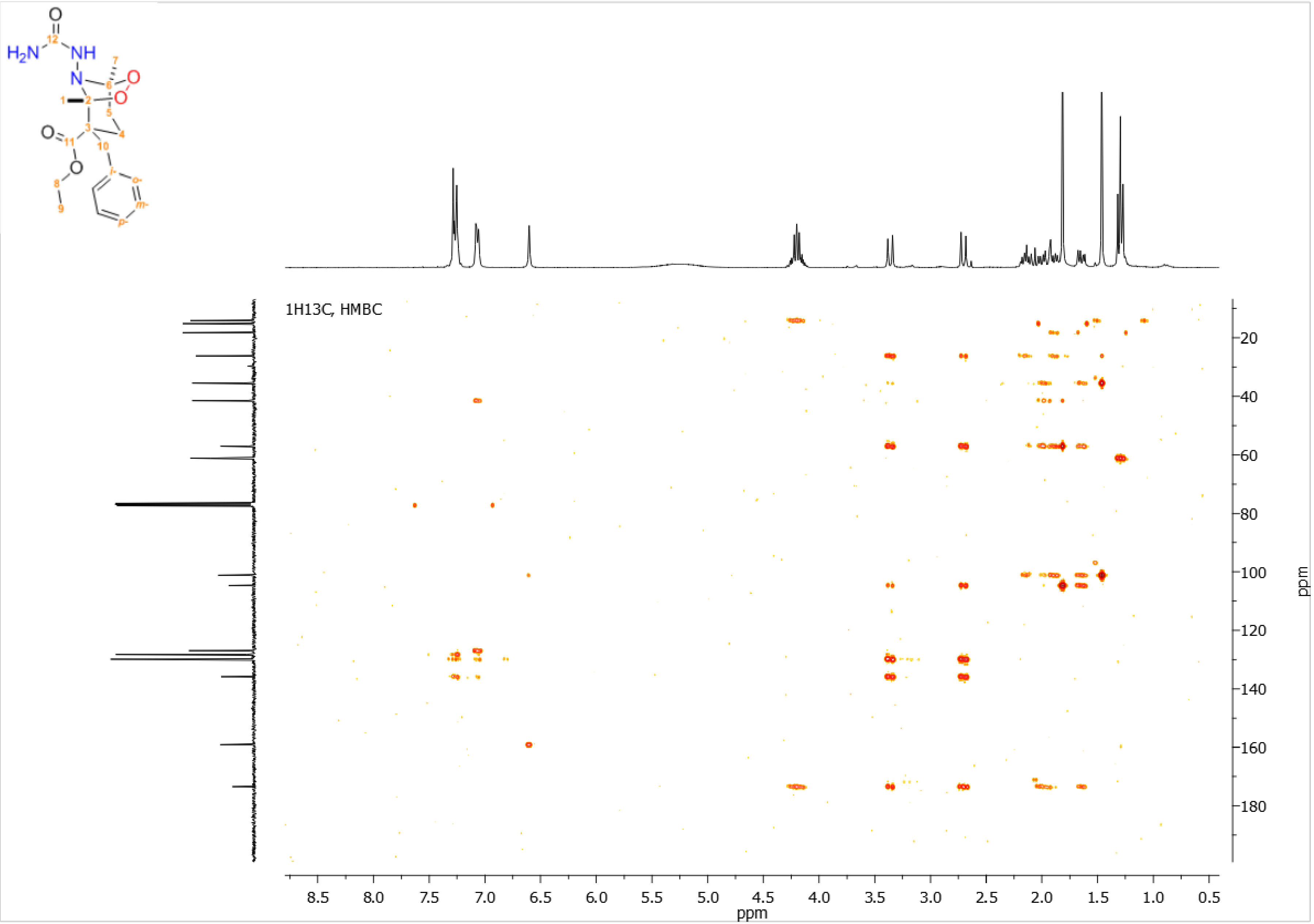

Ethyl 2-benzyl-1,5-dimethyl-8-ureido-6,7-dioxa-8-azabicyclo[3.2.1]octane-2-carboxylate, 14a

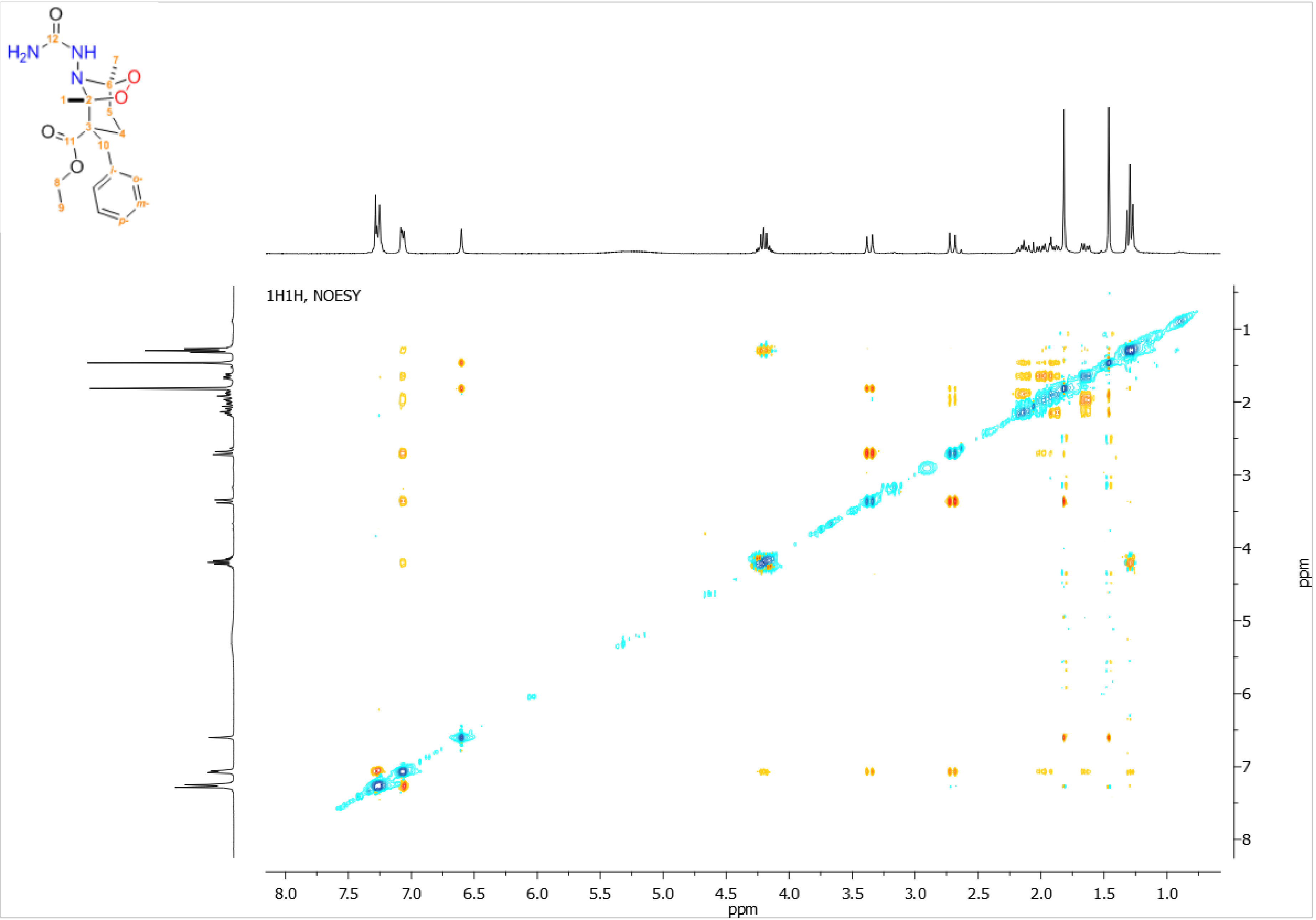

Ethyl 2-benzyl-1,5-dimethyl-8-ureido-6,7-dioxa-8-azabicyclo[3.2.1]octane-2-carboxylate, 14a

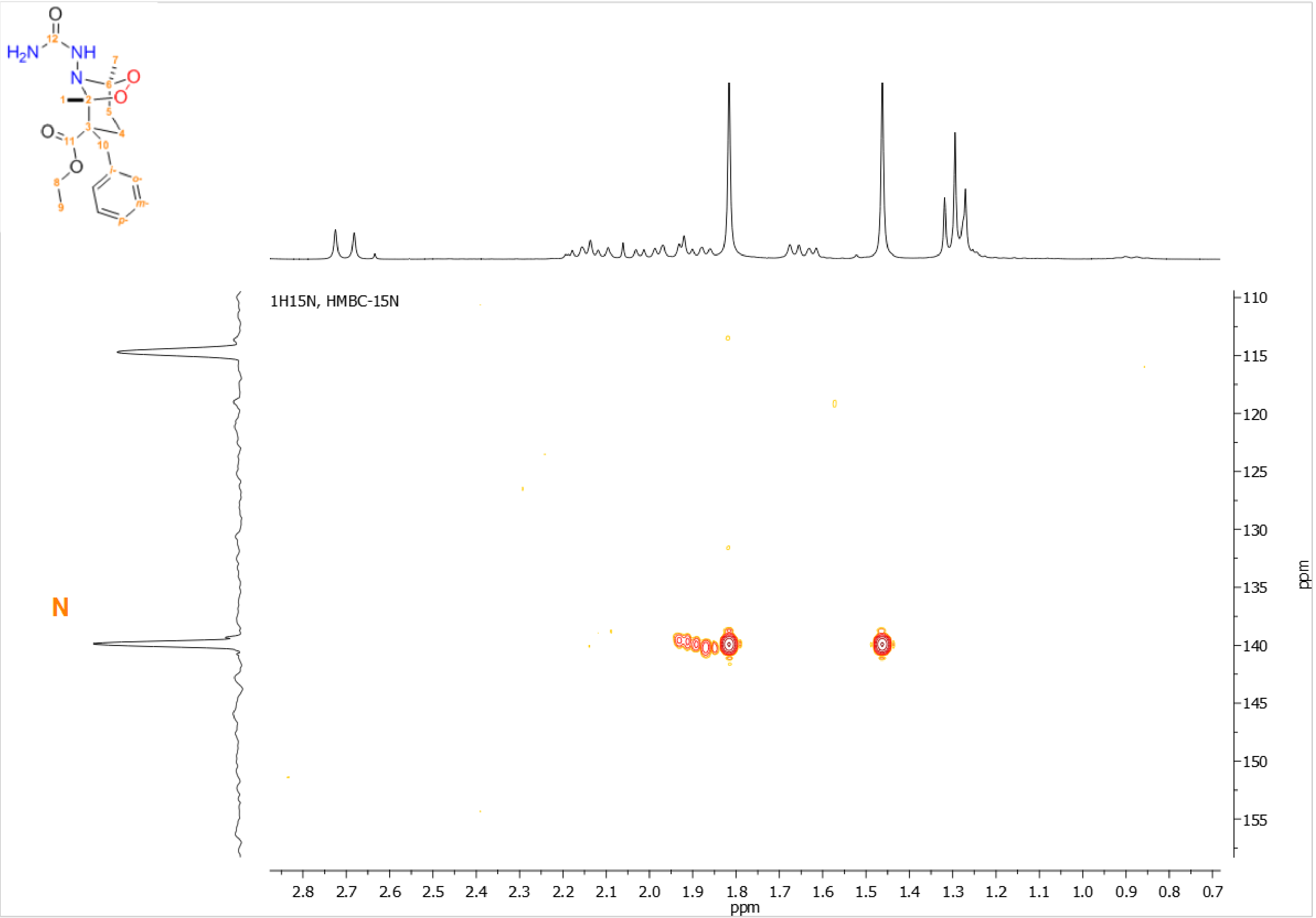

Ethyl 2-benzyl-1,5-dimethyl-8-ureido-6,7-dioxa-8-azabicyclo[3.2.1]octane-2-carboxylate, 14a

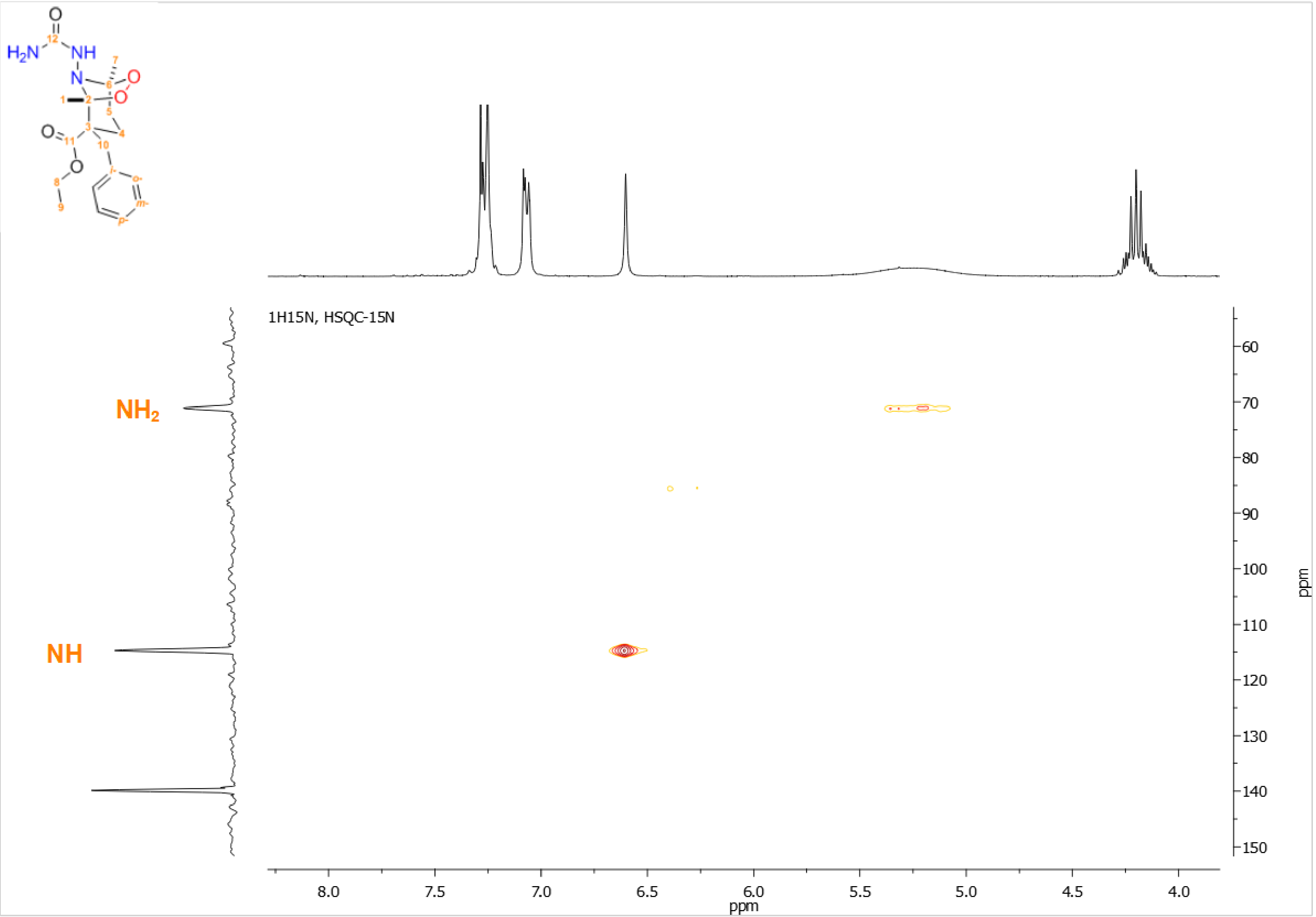

^{1}H NMR (300.13 MHz, CDCl3). Ethyl 8-(2-bromobenzamido)-2-(4-chlorobenzyl)-1,5-dimethyl-6,7-dioxa-8-azabicyclo[3.2.1]octane-2-carboxylate, 15a+15b

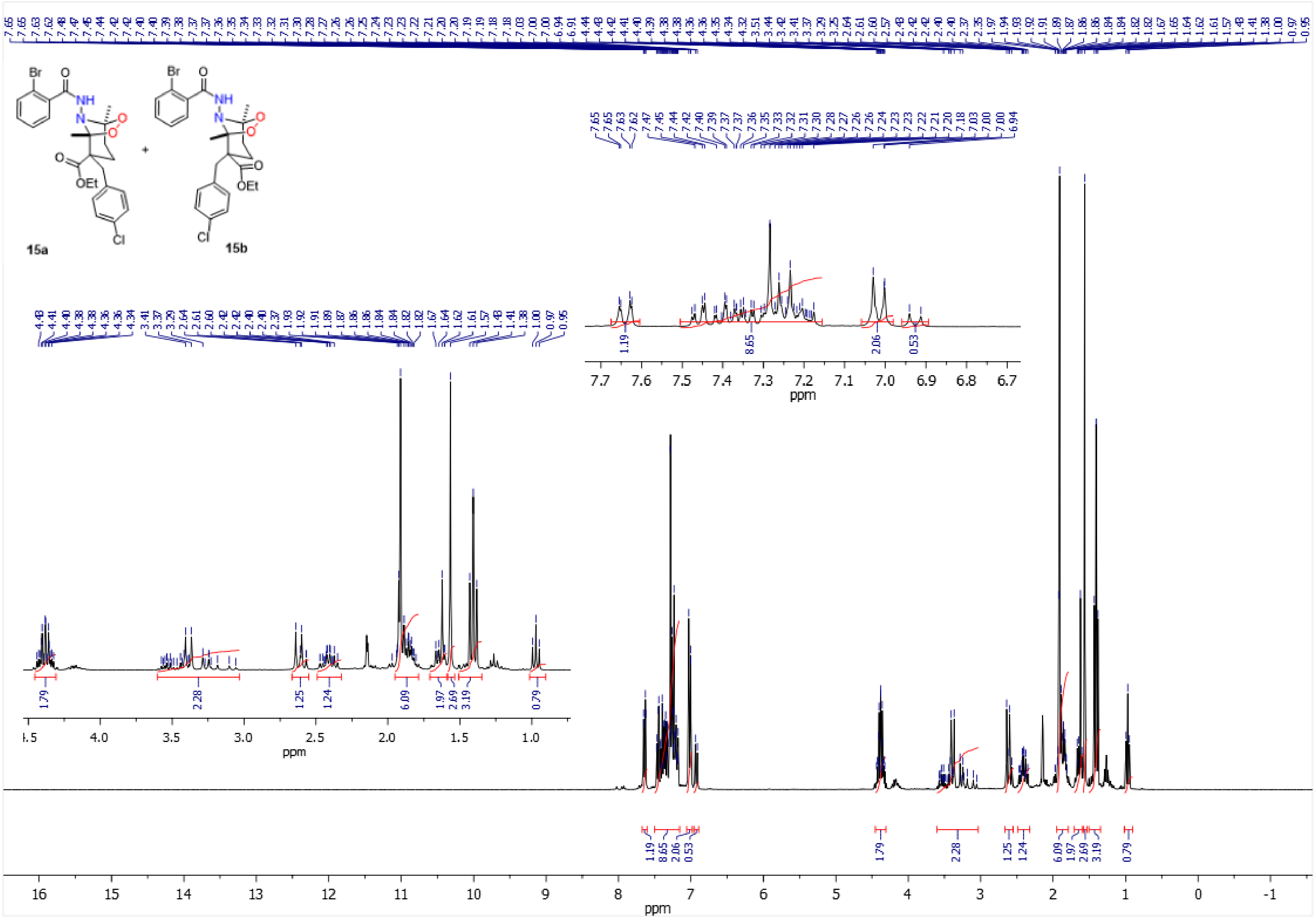

^{13}C NMR (75.48 MHz, $CDCl_3$). Ethyl 8-(2-bromobenzamido)-2-(4-chlorobenzyl)-1,5-dimethyl-6,7-dioxa-8-azabicyclo[3.2.1]octane-2-carboxylate, 15a+15b

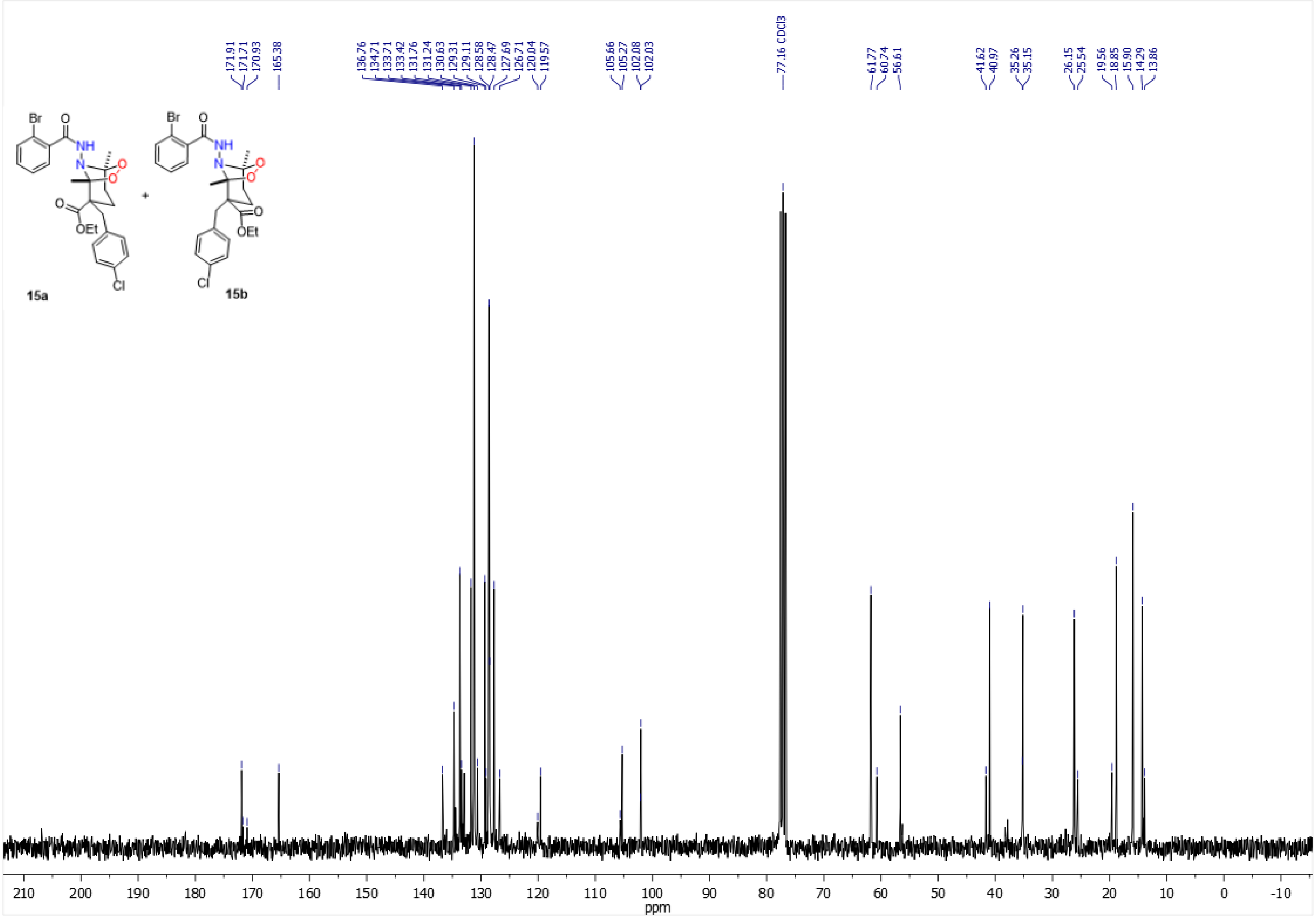

^{1}H NMR (300.13 MHz, CDCl₃). Ethyl 8-(2-bromobenzamido)-2-ethyl-1,5-dimethyl-6,7-dioxa-8-azabicyclo[3.2.1]octane-2-carboxylate, 16a + 16b

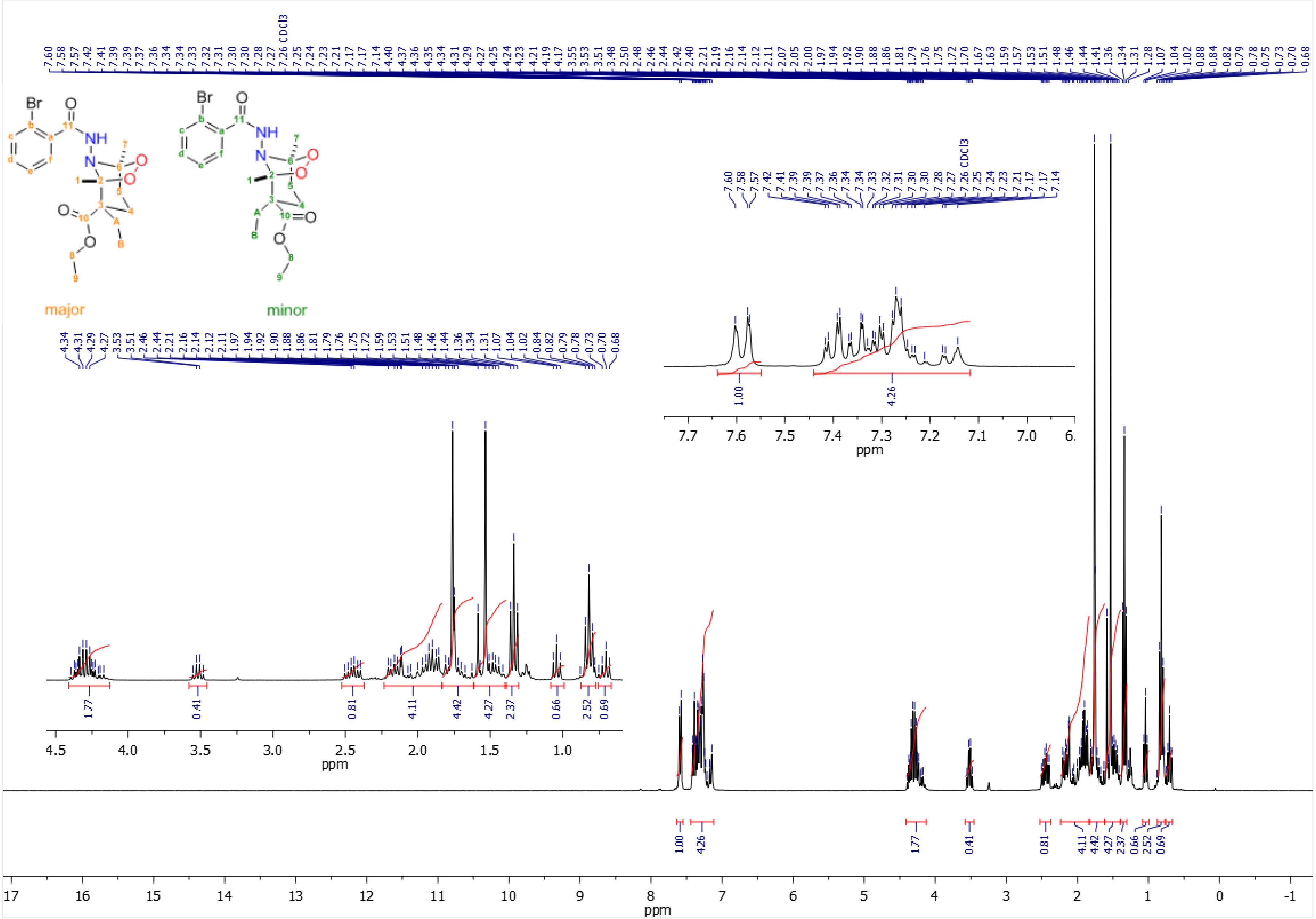

^{1}H NMR (300.13 MHz, CDCl3). Ethyl 8-(2-bromobenzamido)-2-ethyl-1,5-dimethyl-6,7-dioxa-8-azabicyclo[3.2.1]octane-2-carboxylate, 16a + 16b

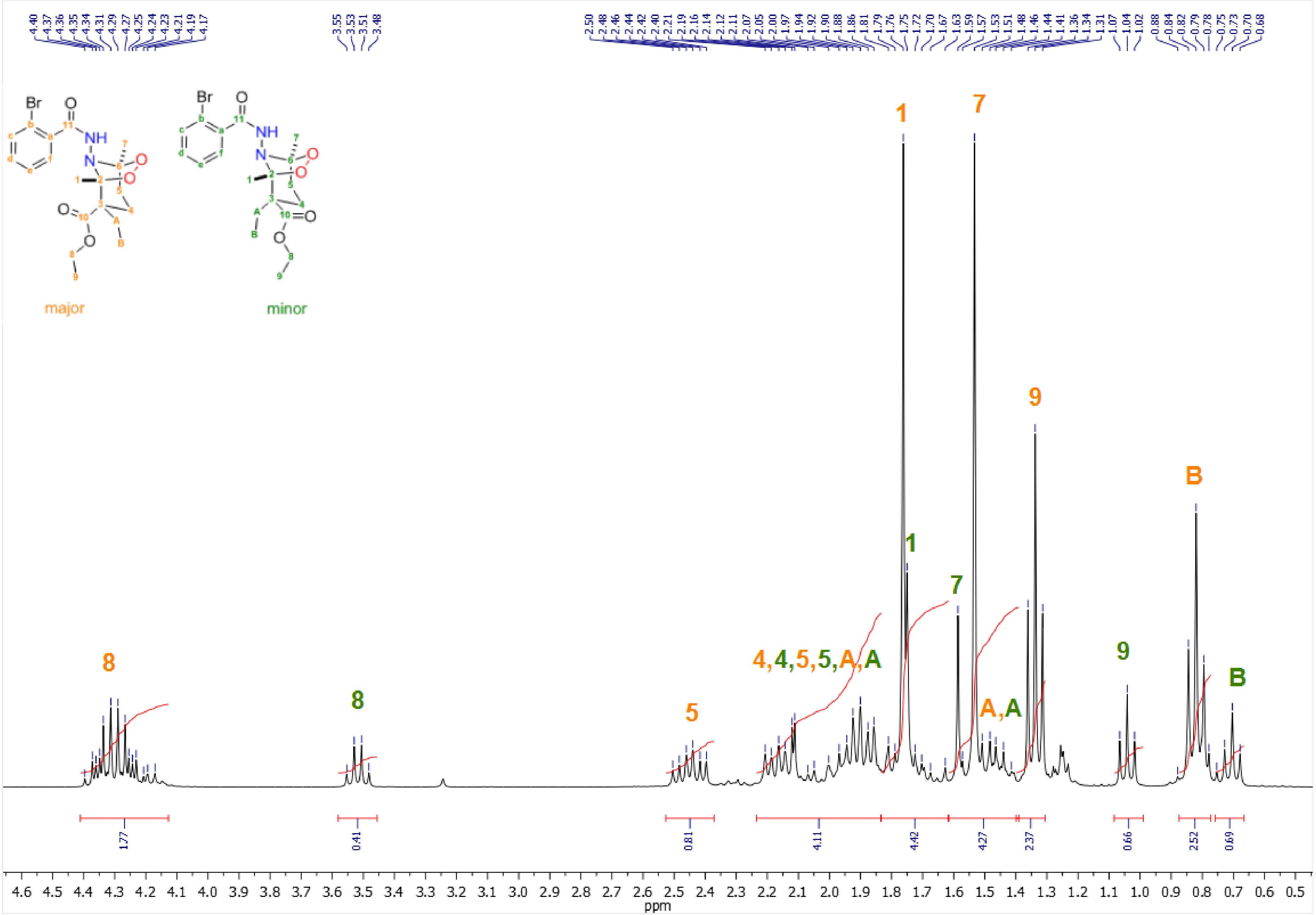

^{1}H NMR (300.13 MHz, CDCl3). Ethyl 8-(2-bromobenzamido)-2-ethyl-1,5-dimethyl-6,7-dioxa-8-azabicyclo[3.2.1]octane-2-carboxylate, 16a + 16b

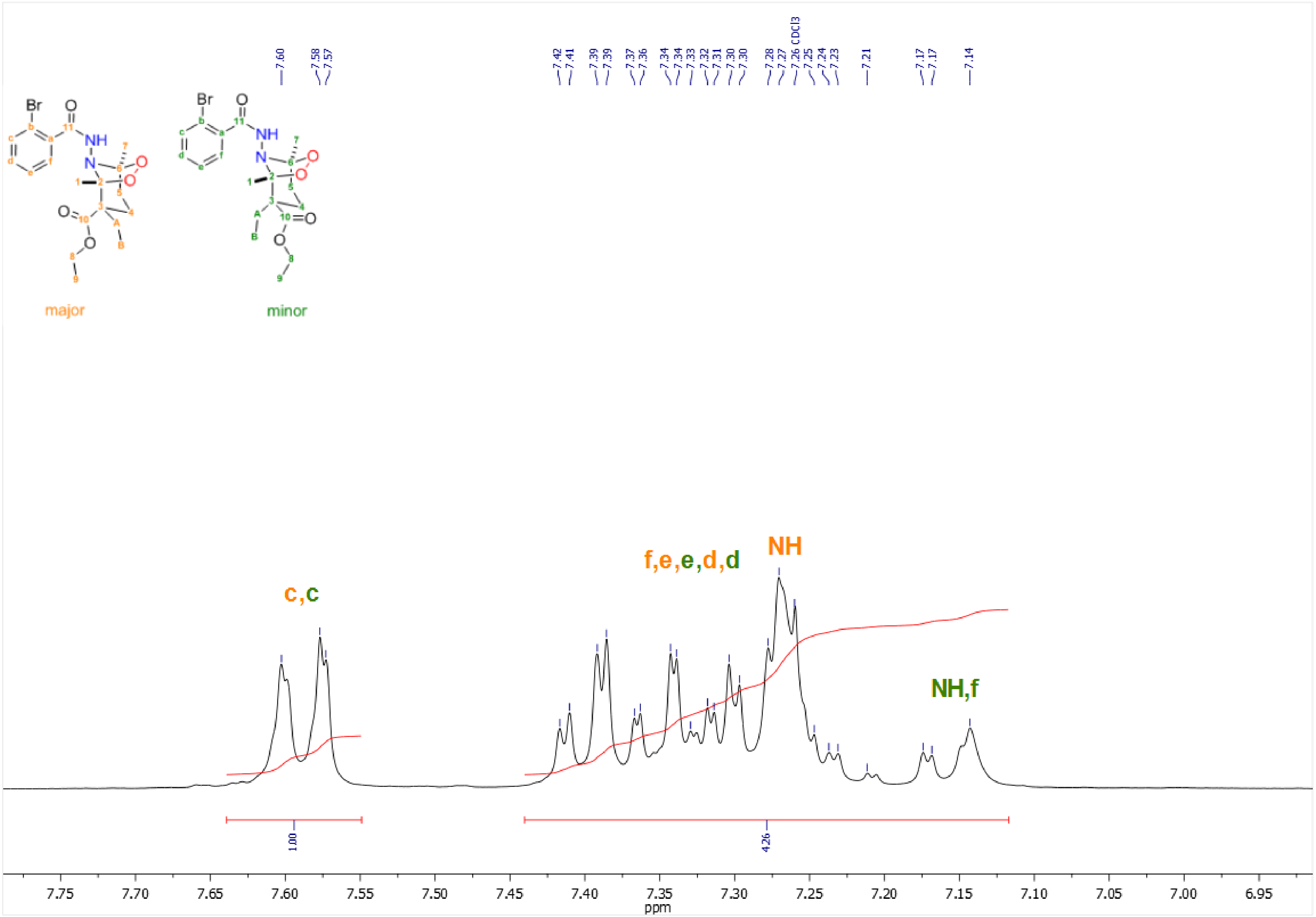

^{13}C NMR (75.48 MHz, CDCl3). Ethyl 8-(2-bromobenzamido)-2-ethyl-1,5-dimethyl-6,7-dioxa-8-azabicyclo[3.2.1]octane-2-carboxylate, 16a + 16b

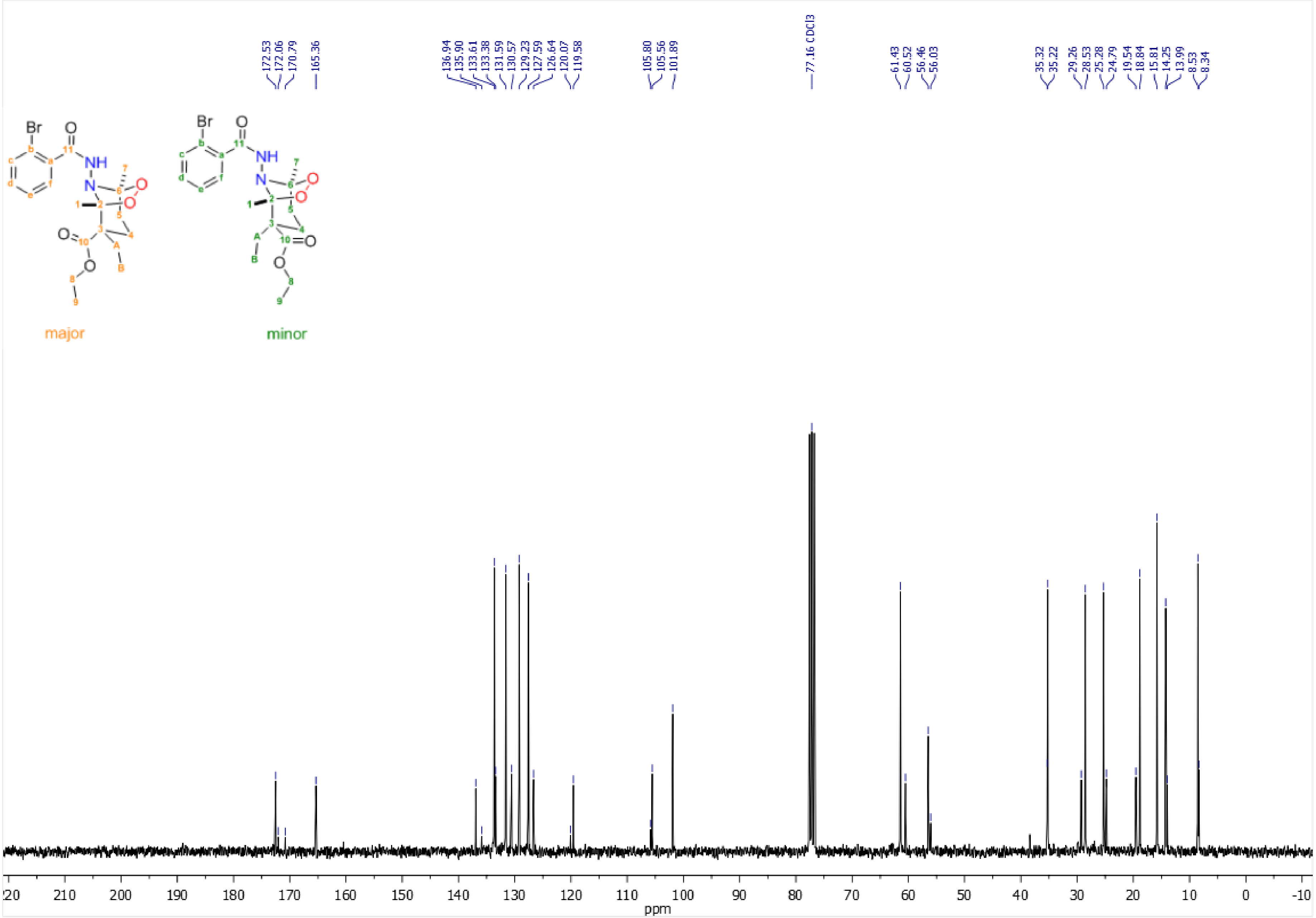

^{13}C NMR (75.48 MHz, CDCl3). Ethyl 8-(2-bromobenzamido)-2-ethyl-1,5-dimethyl-6,7-dioxa-8-azabicyclo[3.2.1]octane-2-carboxylate, 16a + 16b

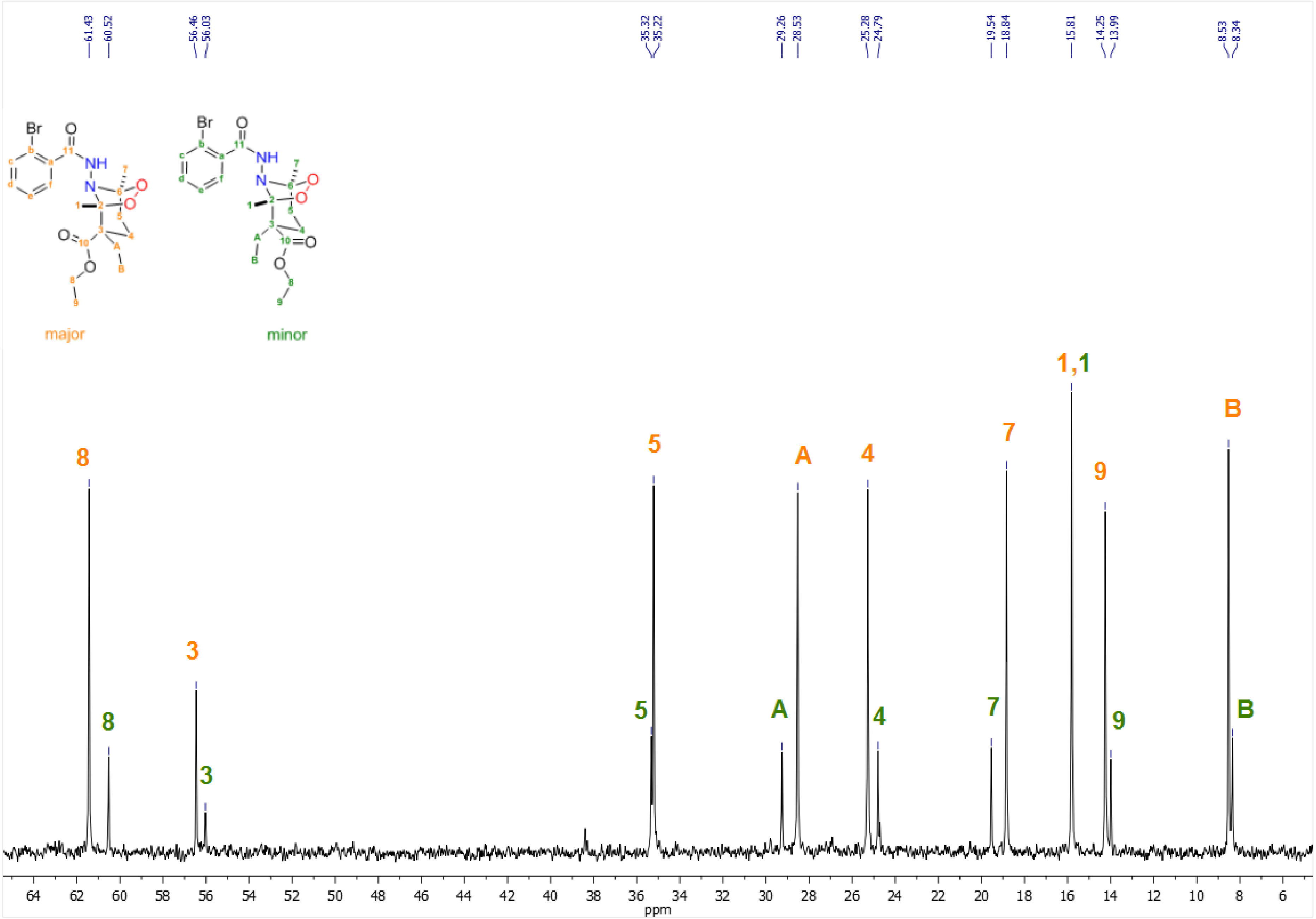

^{13}C NMR (75.48 MHz, CDCl$_3$). Ethyl 8-(2-bromobenzamido)-2-ethyl-1,5-dimethyl-6,7-dioxa-8-azabicyclo[3.2.1]octane-2-carboxylate, 16a + 16b

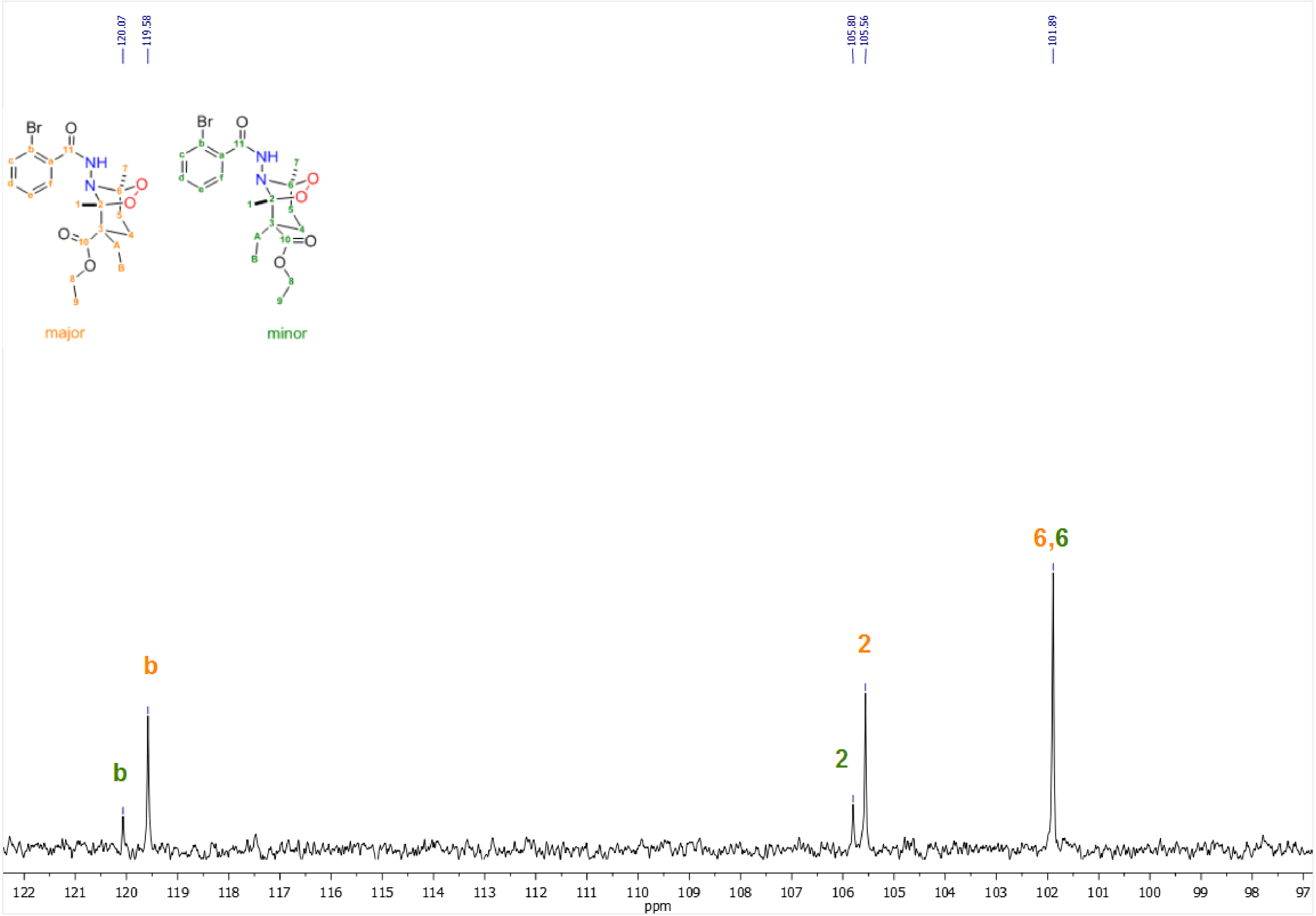

^{13}C NMR (75.48 MHz, $CDCl_3$). Ethyl 8-(2-bromobenzamido)-2-ethyl-1,5-dimethyl-6,7-dioxa-8-azabicyclo[3.2.1]octane-2-carboxylate, 16a + 16b

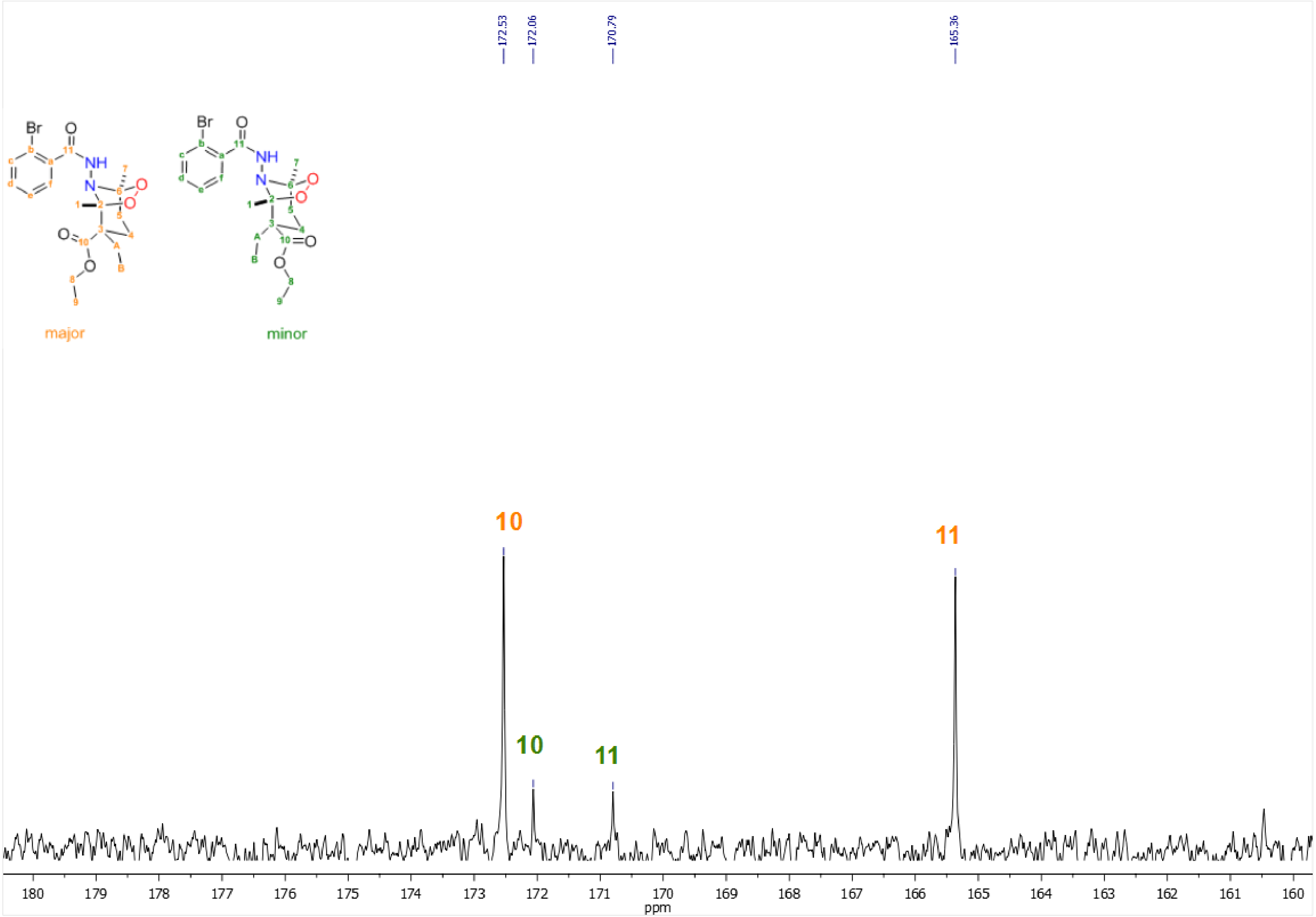

^{15}N (40.56 MHz, $CDCl_3$). Ethyl 8-(2-bromobenzamido)-2-ethyl-1,5-dimethyl-6,7-dioxa-8-azabicyclo[3.2.1]octane-2-carboxylate, 16a + 16b

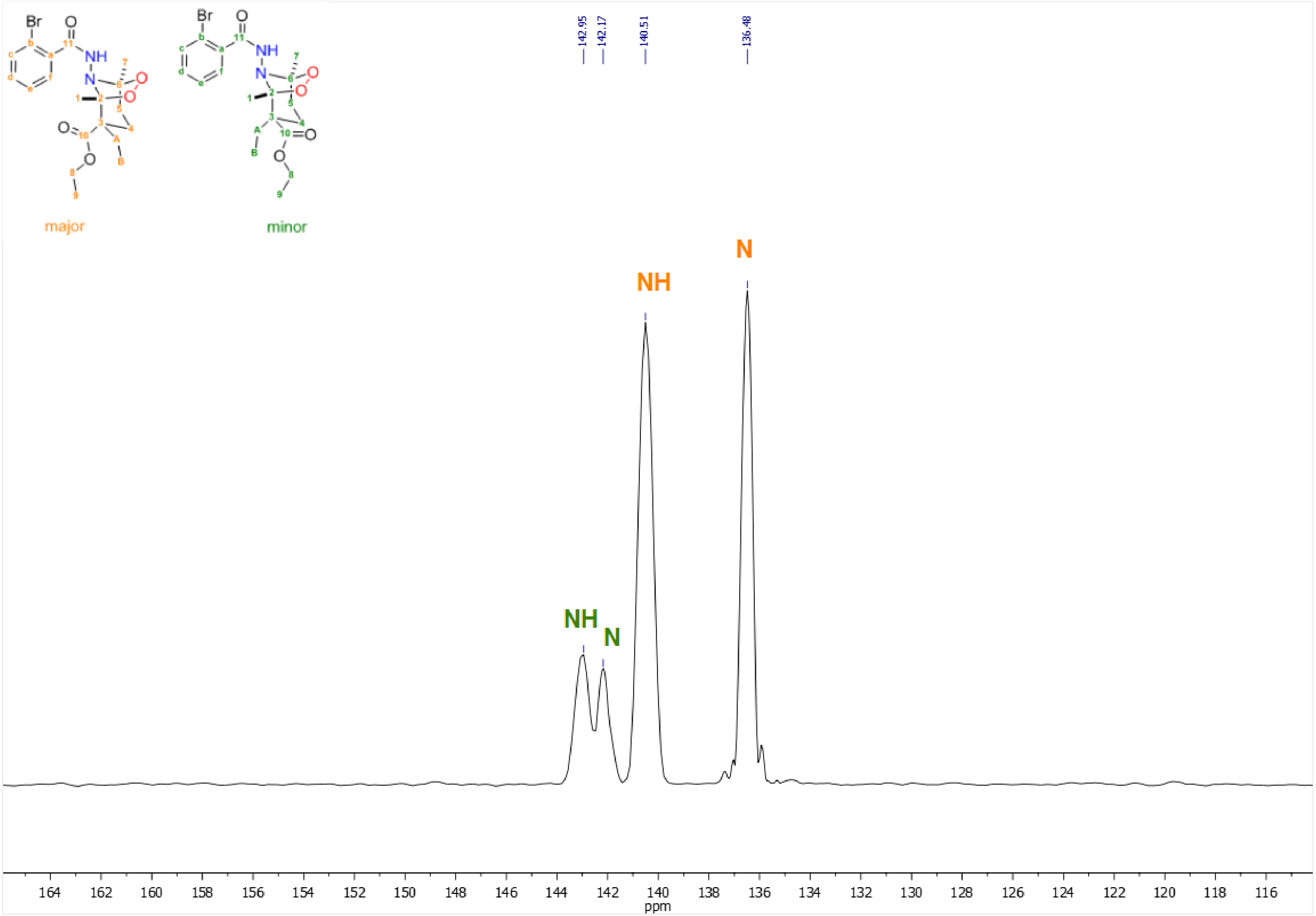

^{13}C NMR (75.48 MHz, $CDCl_3$). Ethyl 8-(2-bromobenzamido)-2-ethyl-1,5-dimethyl-6,7-dioxa-8-azabicyclo[3.2.1]octane-2-carboxylate, 16a + 16b

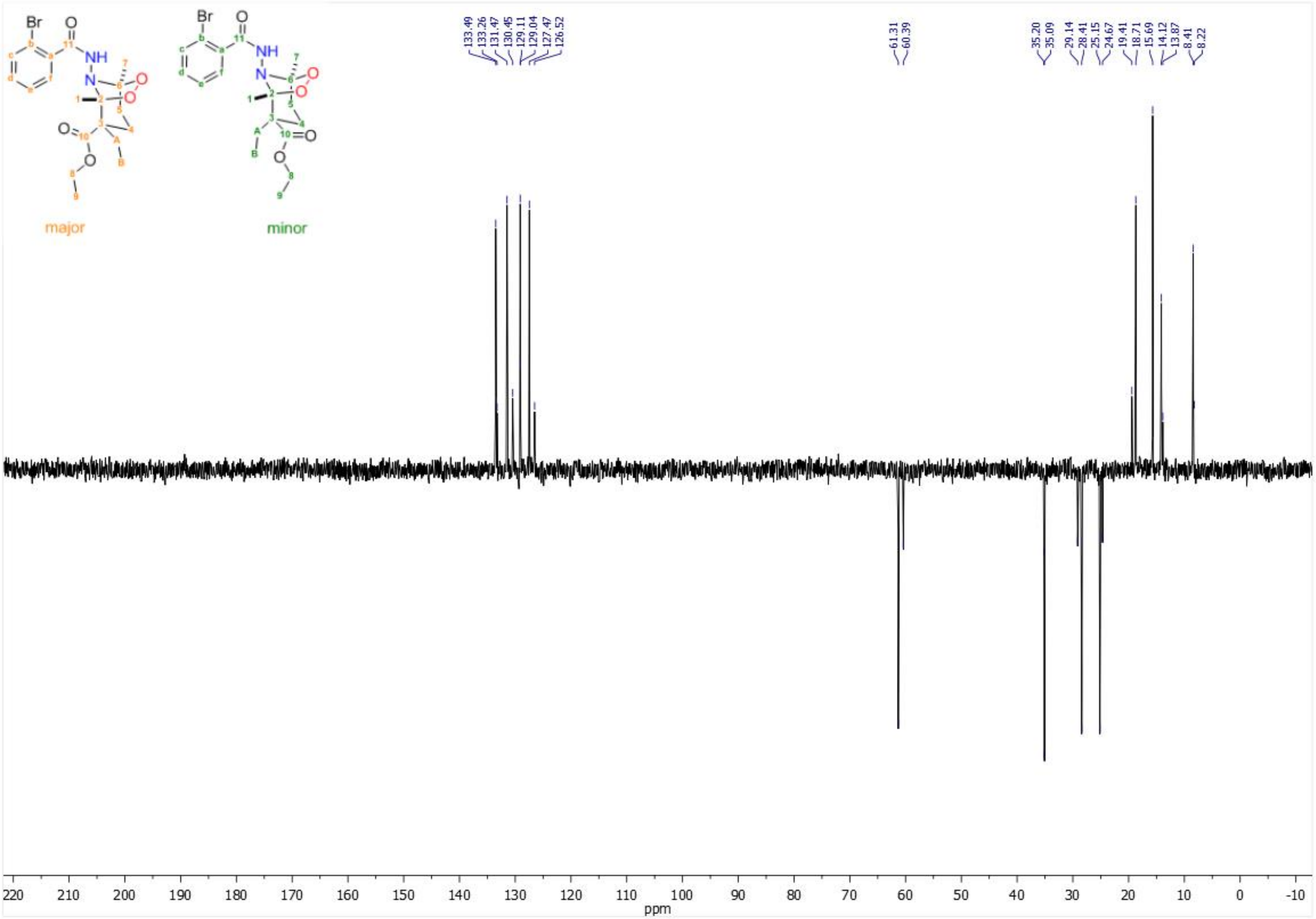

Ethyl 8-(2-bromobenzamido)-2-ethyl-1,5-dimethyl-6,7-dioxa-8-azabicyclo[3.2.1]octane-2-carboxylate, 16a + 16b

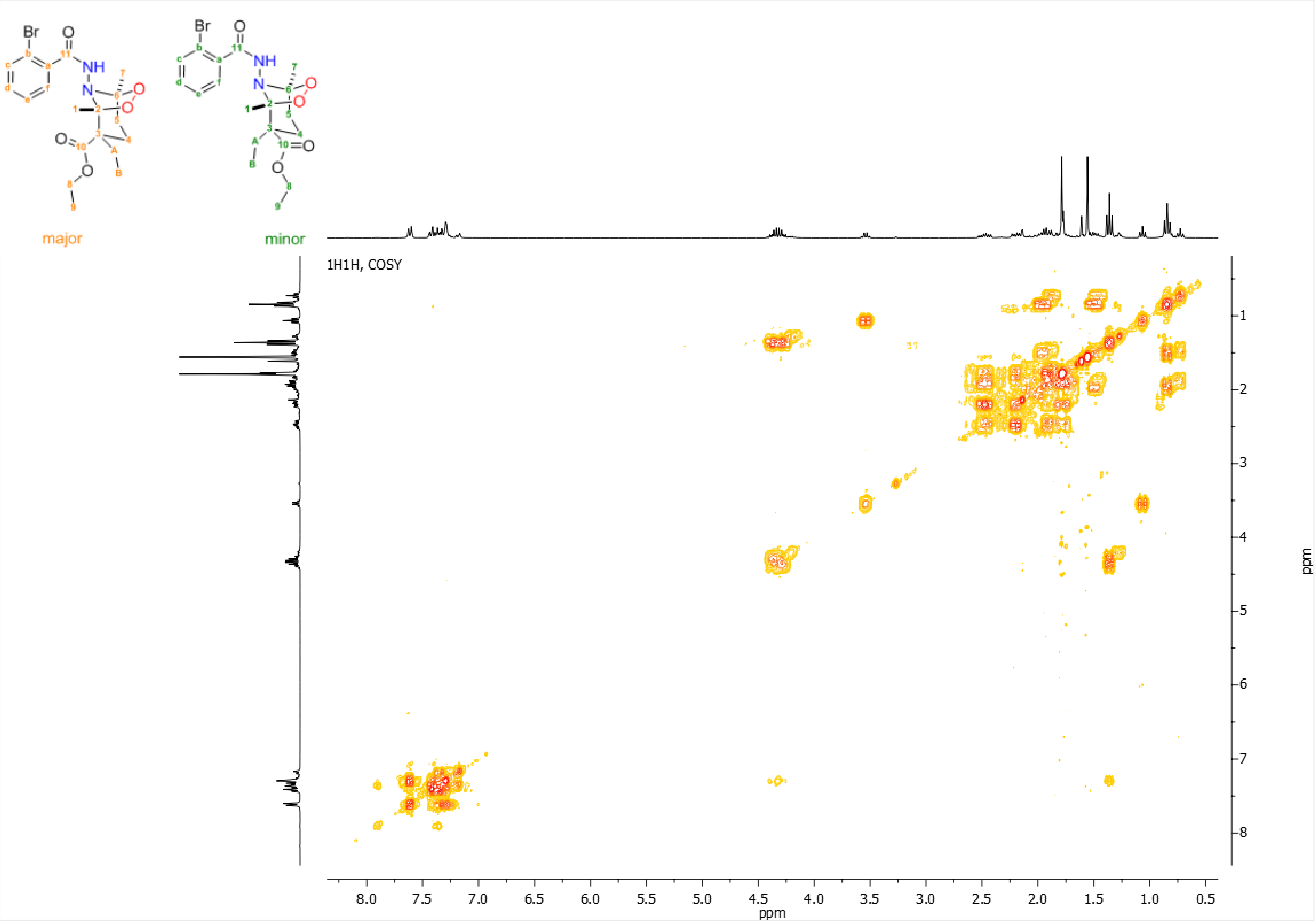

Ethyl 8-(2-bromobenzamido)-2-ethyl-1,5-dimethyl-6,7-dioxa-8-azabicyclo[3.2.1]octane-2-carboxylate, 16a + 16b

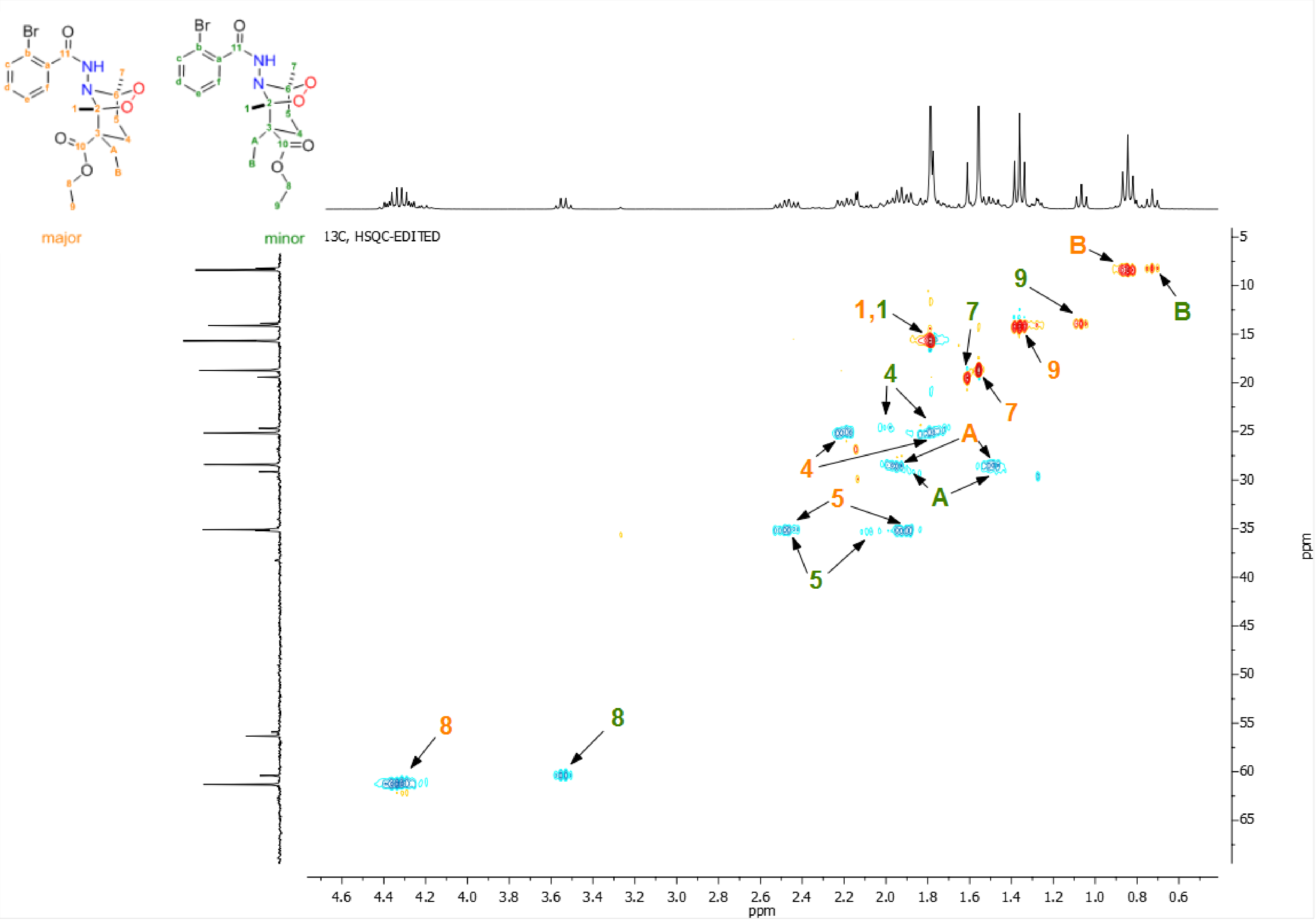

Ethyl 8-(2-bromobenzamido)-2-ethyl-1,5-dimethyl-6,7-dioxa-8-azabicyclo[3.2.1]octane-2-carboxylate, 16a + 16b

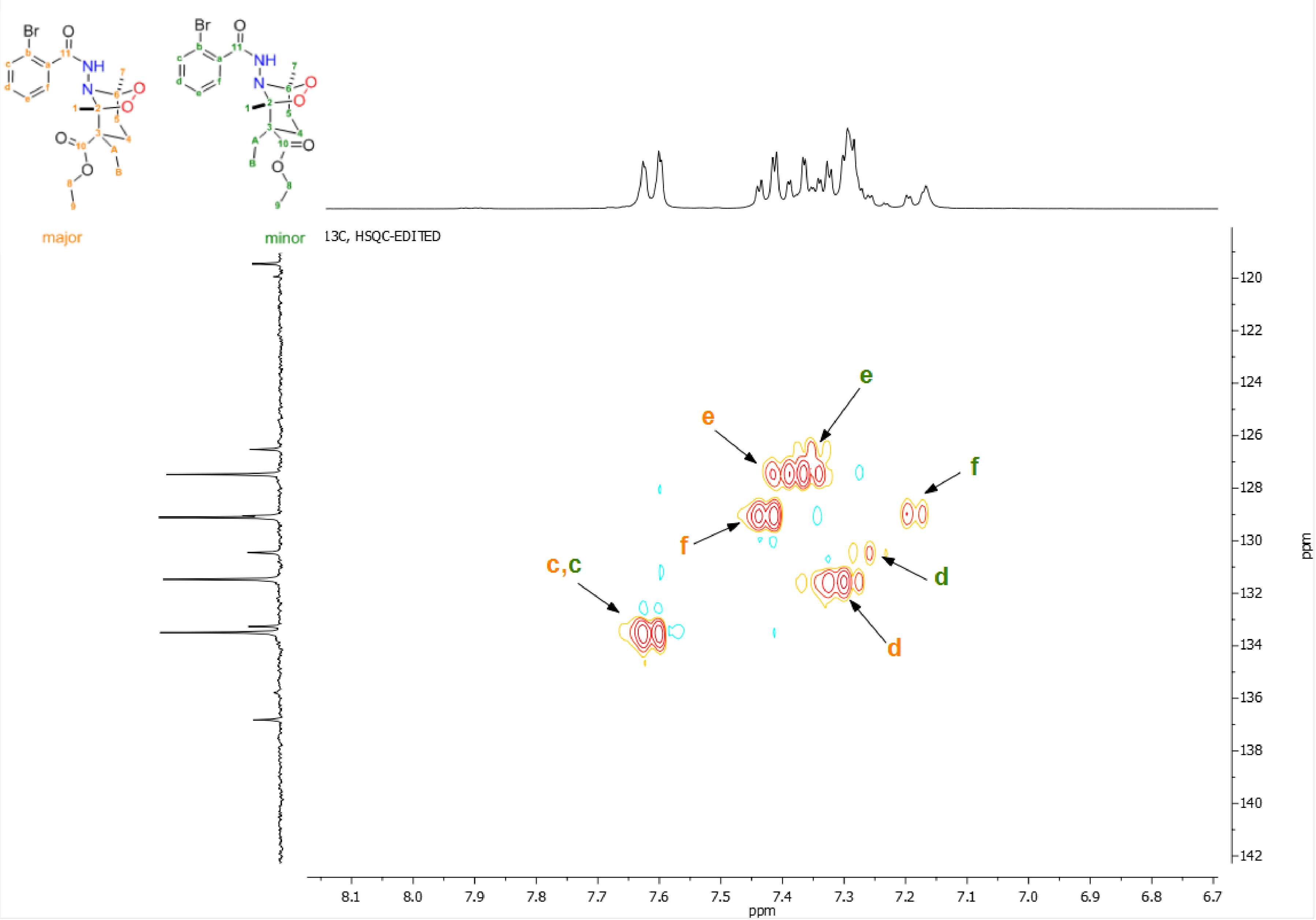

Ethyl 8-(2-bromobenzamido)-2-ethyl-1,5-dimethyl-6,7-dioxa-8-azabicyclo[3.2.1]octane-2-carboxylate, 16a + 16b

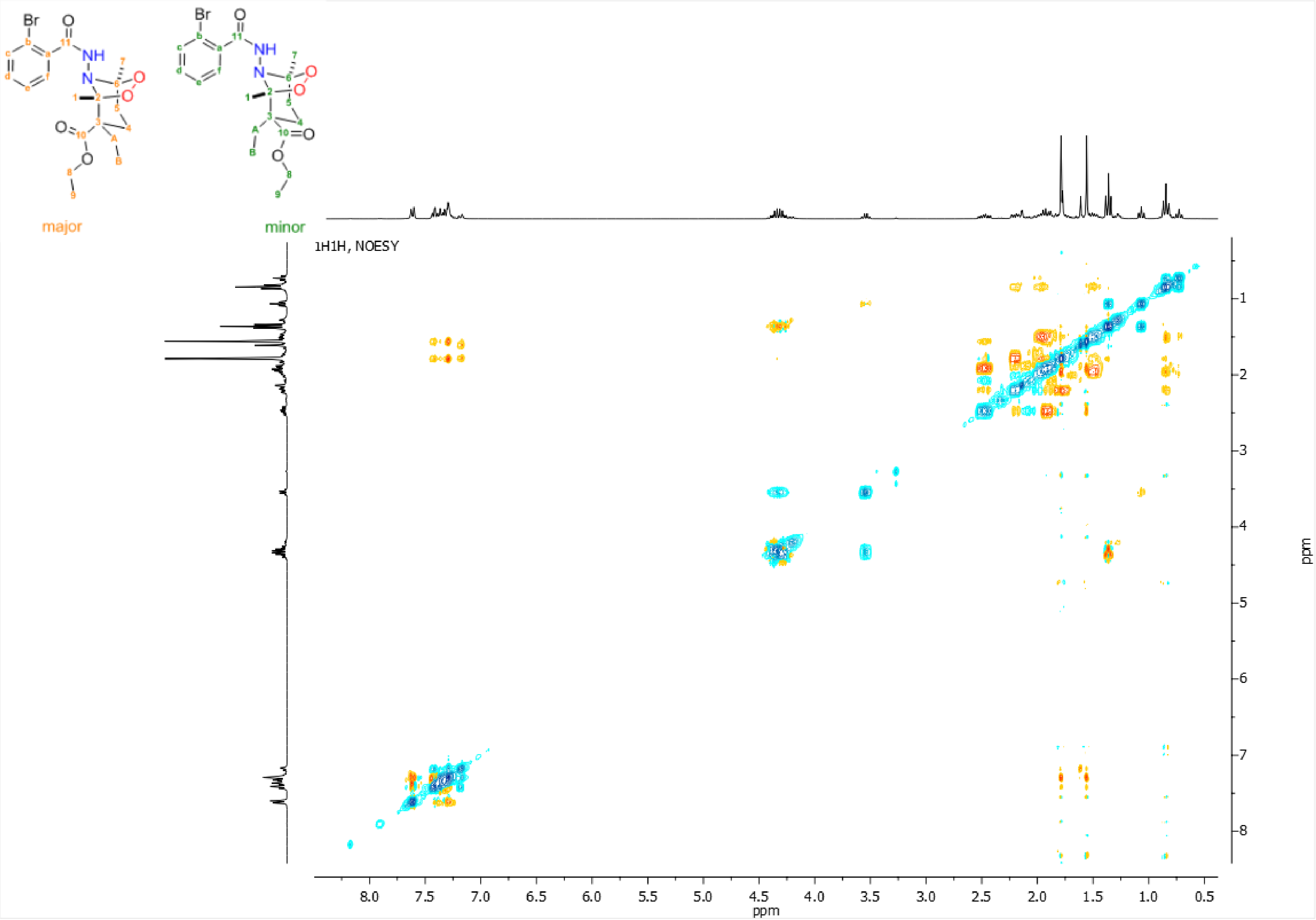

Ethyl 8-(2-bromobenzamido)-2-ethyl-1,5-dimethyl-6,7-dioxa-8-azabicyclo[3.2.1]octane-2-carboxylate, 16a + 16b

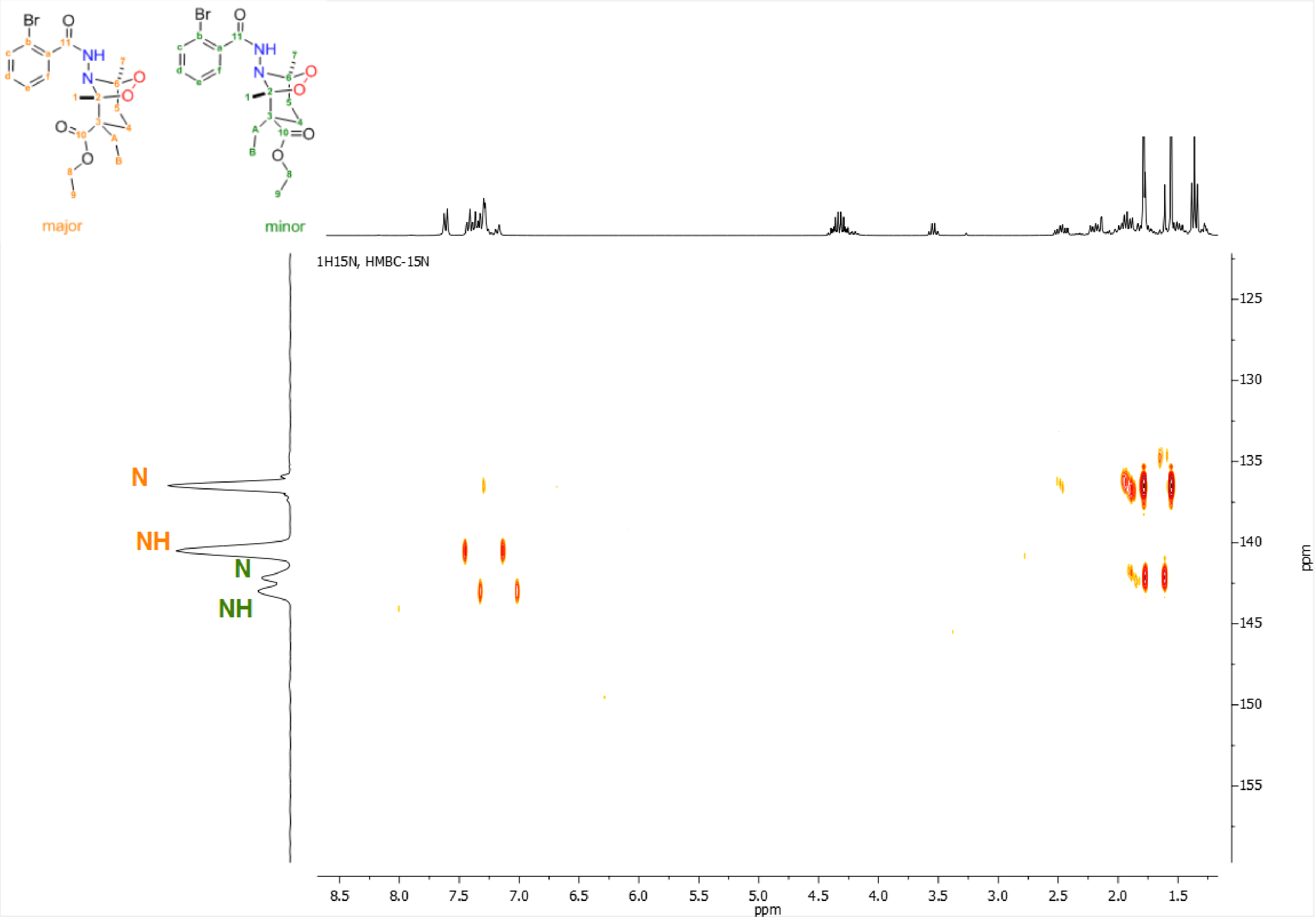

Ethyl 8-(2-bromobenzamido)-2-ethyl-1,5-dimethyl-6,7-dioxa-8-azabicyclo[3.2.1]octane-2-carboxylate, 16a + 16b

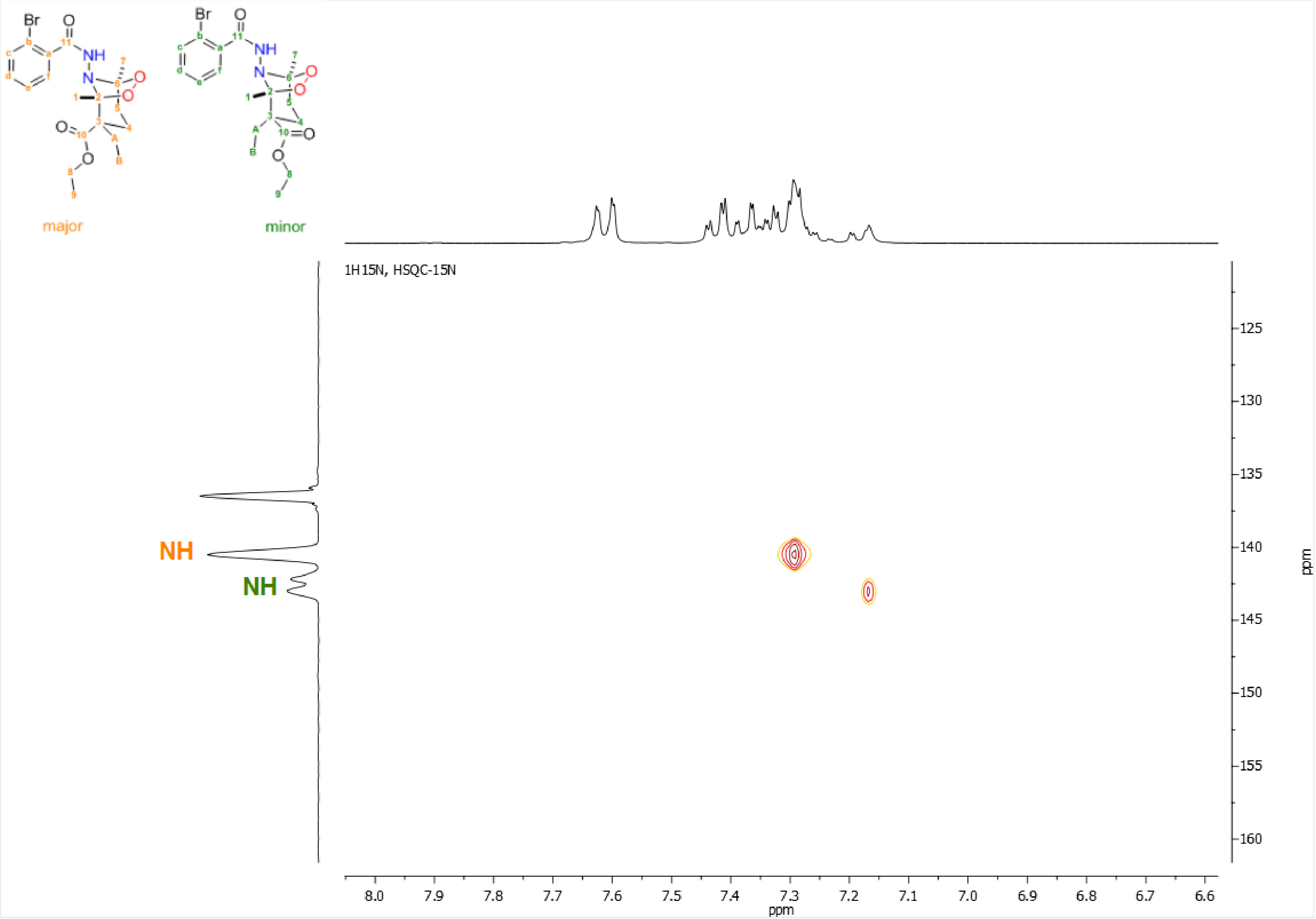

^{1}H NMR (300.13 MHz, CDCl3). Allyl 2-allyl-8-(2-bromobenzamido)-1,5-dimethyl-6,7-dioxa-8-azabicyclo[3.2.1]octane-2-carboxylate, 17a + 17b

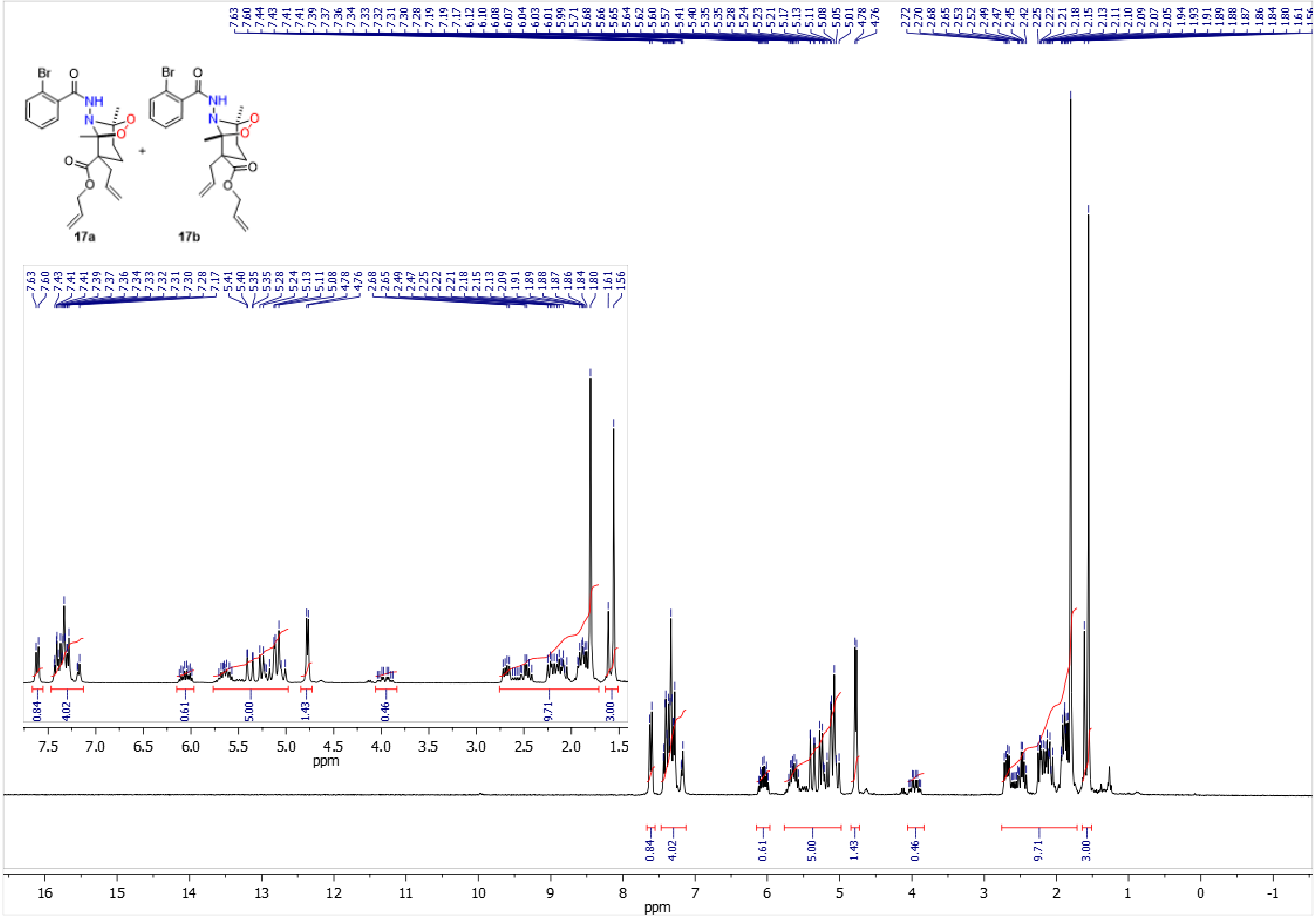

^{13}C NMR (75.48 MHz, CDCl$_3$). Allyl 2-allyl-8-(2-bromobenzamido)-1,5-dimethyl-6,7-dioxa-8-azabicyclo[3.2.1]octane-2-carboxylate, 17a + 17b

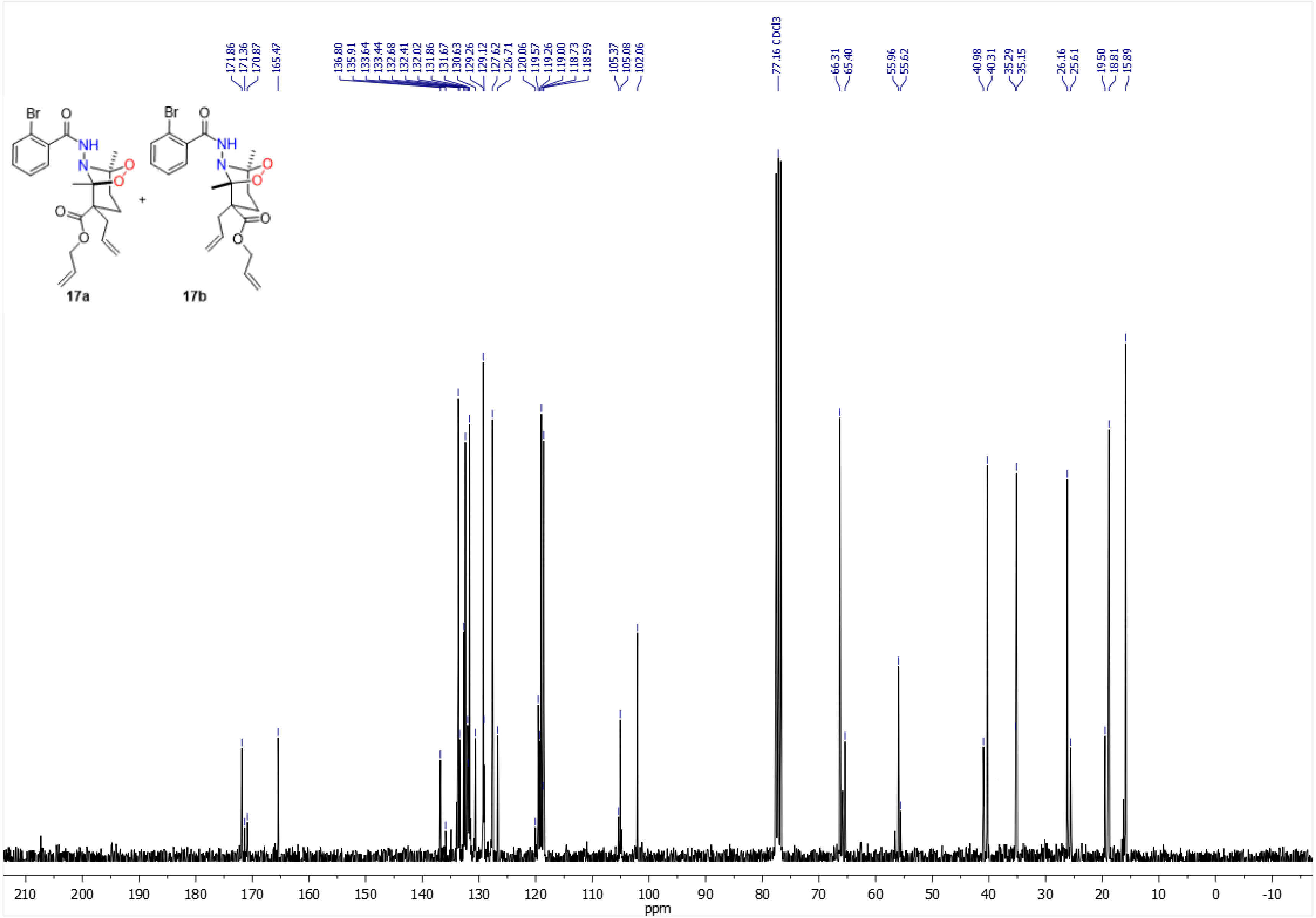

^{1}H NMR (300.13 MHz, CDCl3). *tert*-Butyl 8-(2-bromobenzamido)-2-(4-bromobenzyl)-1,5-dimethyl-6,7-dioxa-8-azabicyclo[3.2.1]octane-2-carboxylate, **18a**

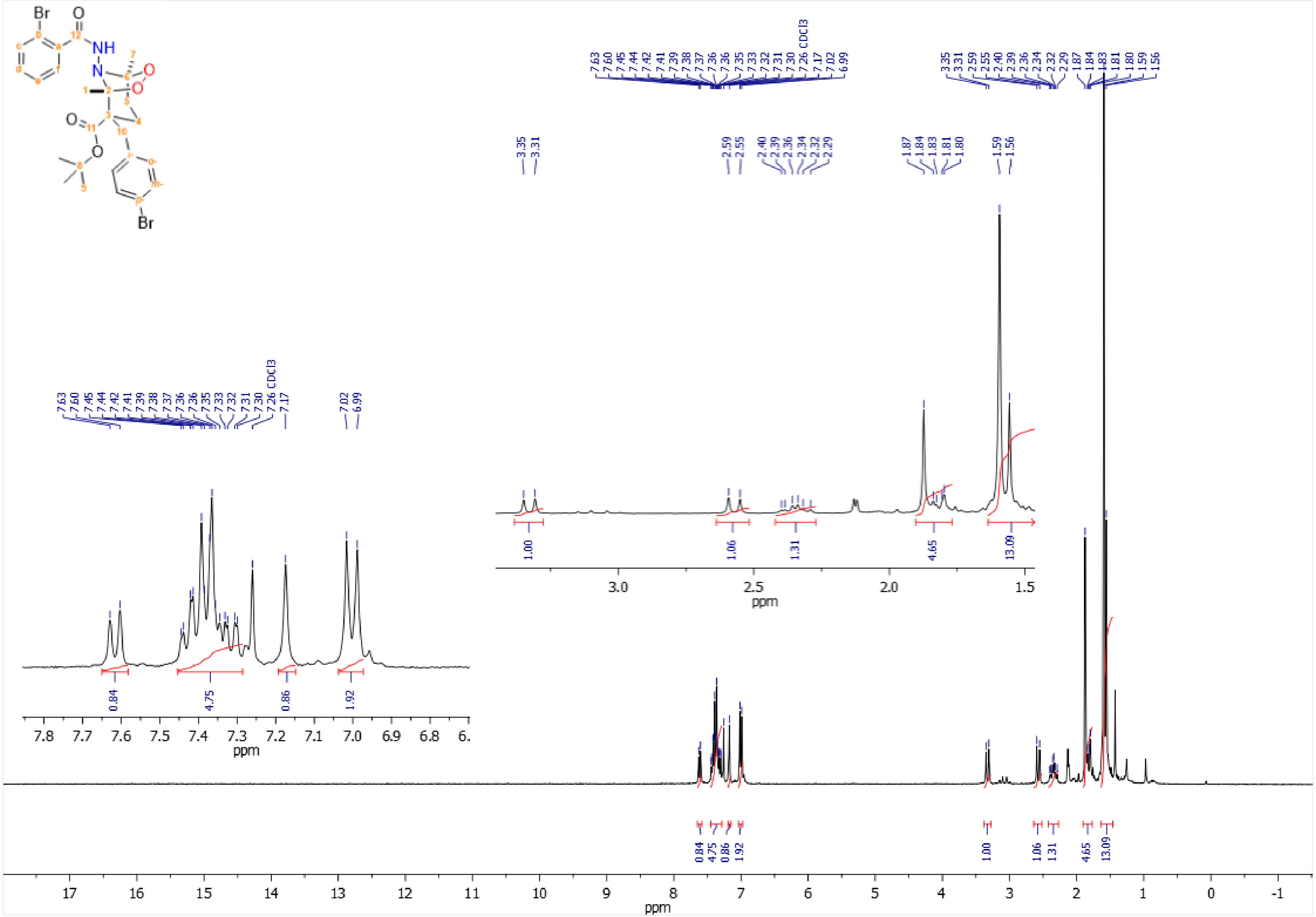

^{1}H NMR (300.13 MHz, $CDCl_3$). *tert*-Butyl 8-(2-bromobenzamido)-2-(4-bromobenzyl)-1,5-dimethyl-6,7-dioxa-8-azabicyclo[3.2.1]octane-2-carboxylate, 18a

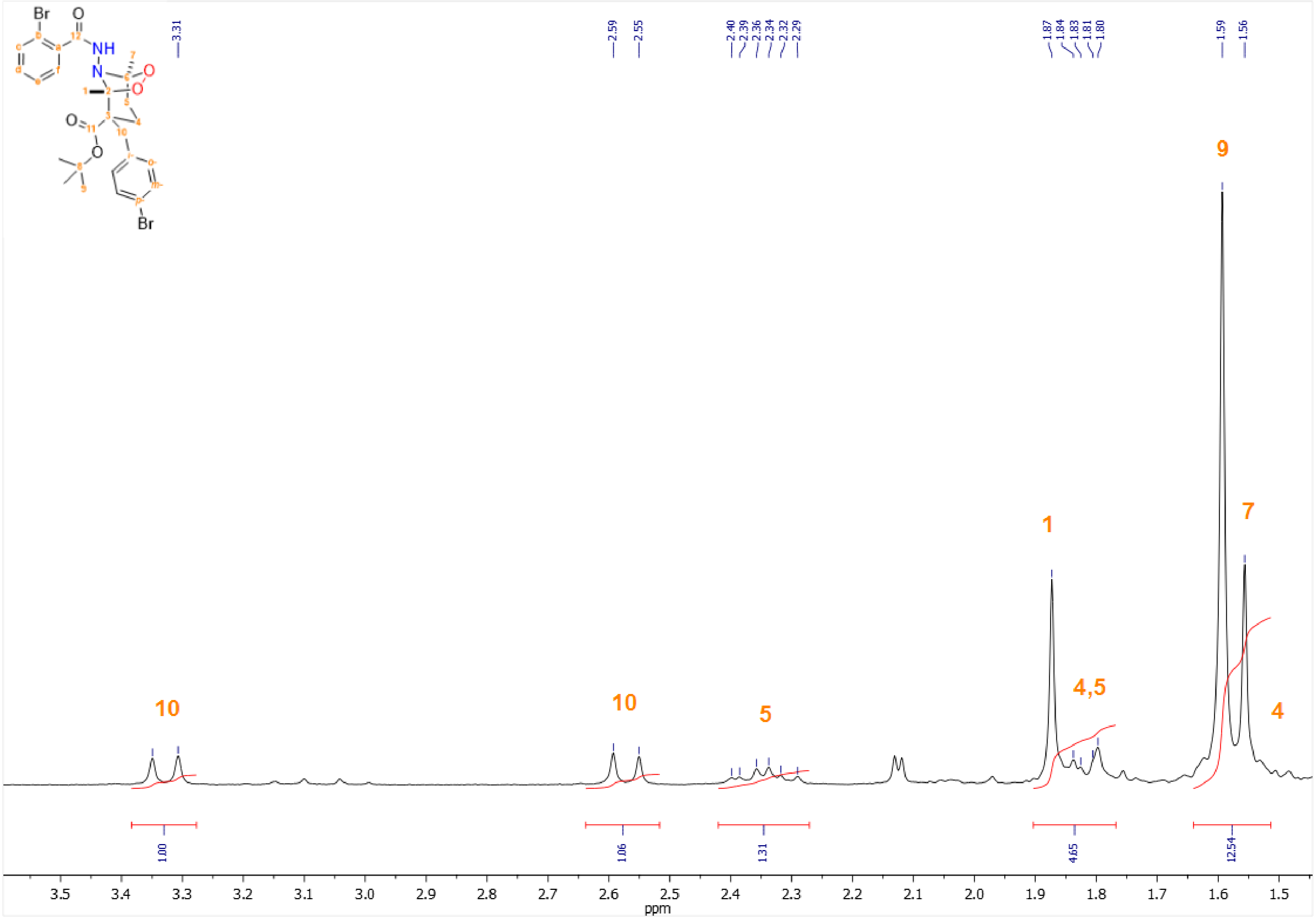

^{1}H NMR (300.13 MHz, CDCl3). *tert*-Butyl 8-(2-bromobenzamido)-2-(4-bromobenzyl)-1,5-dimethyl-6,7-dioxa-8-azabicyclo[3.2.1]octane-2-carboxylate, 18a

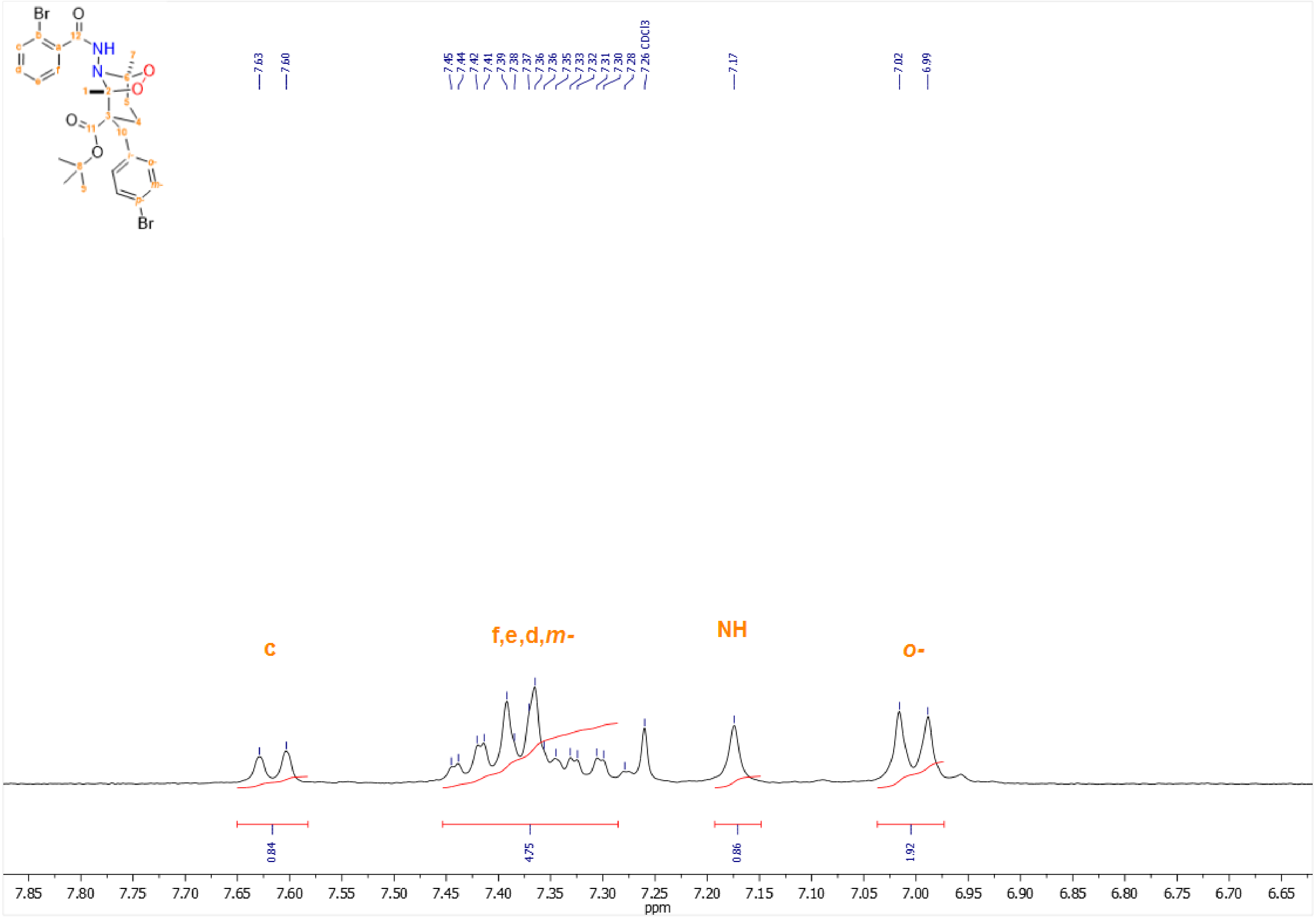

^{13}C NMR (75.48 MHz, $CDCl_3$). *tert*-Butyl 8-(2-bromobenzamido)-2-(4-bromobenzyl)-1,5-dimethyl-6,7-dioxa-8-azabicyclo[3.2.1]octane-2-carboxylate, 18a

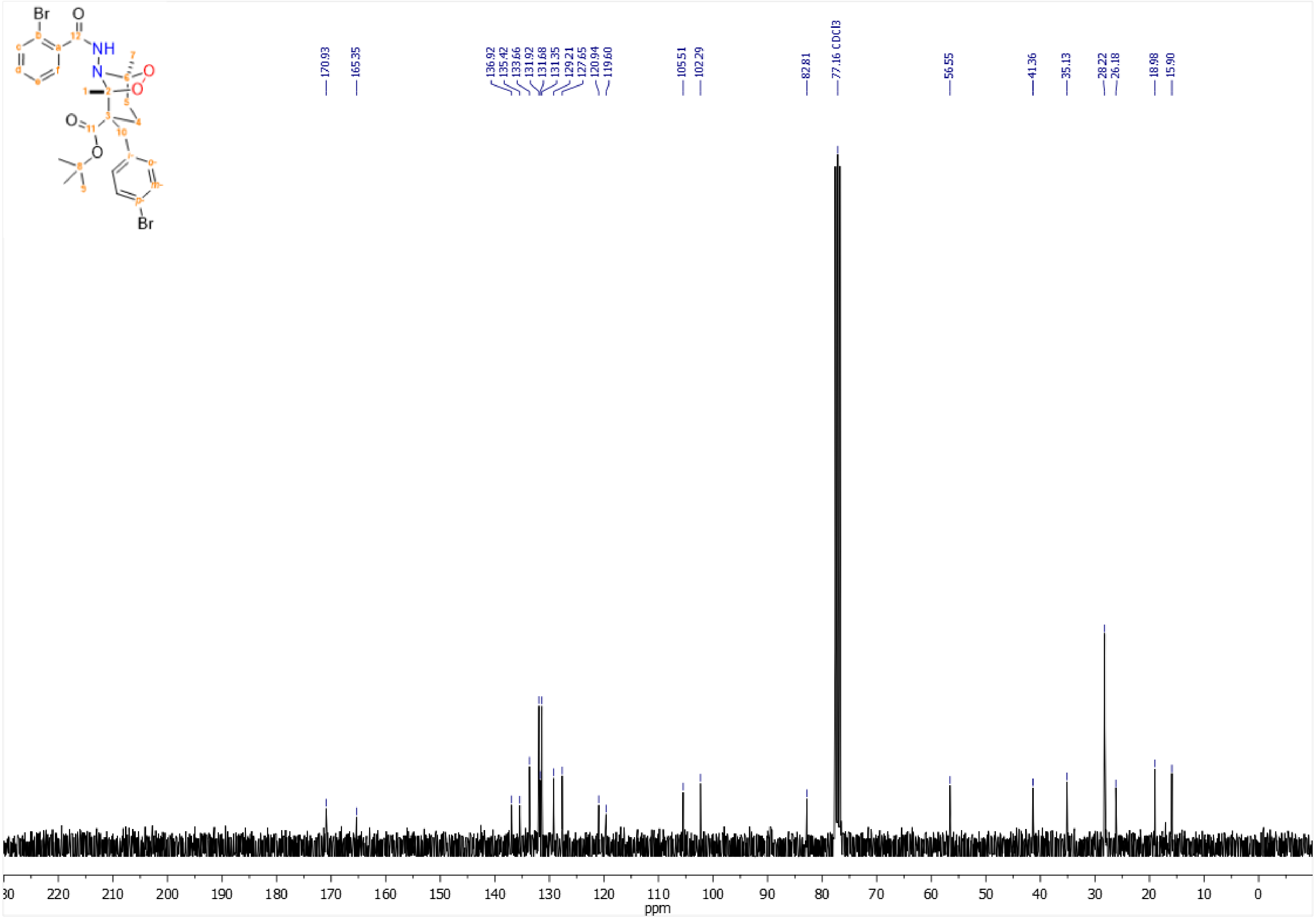

^{13}C NMR (75.48 MHz, CDCl$_3$). *tert*-Butyl 8-(2-bromobenzamido)-2-(4-bromobenzyl)-1,5-dimethyl-6,7-dioxa-8-azabicyclo[3.2.1]octane-2-carboxylate, 18a

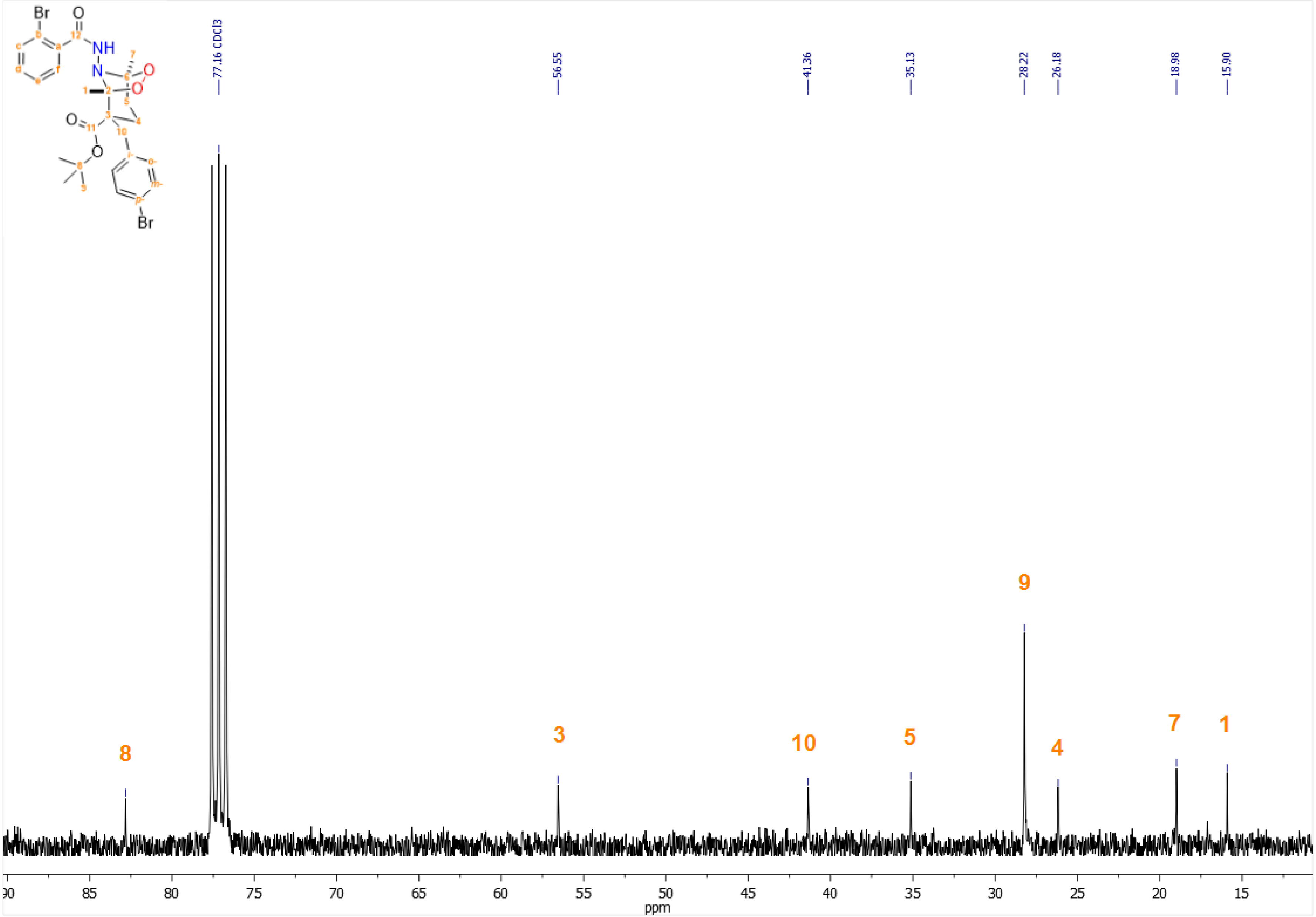

^{13}C NMR (75.48 MHz, $CDCl_3$). *tert*-Butyl 8-(2-bromobenzamido)-2-(4-bromobenzyl)-1,5-dimethyl-6,7-dioxa-8-azabicyclo[3.2.1]octane-2-carboxylate, 18a

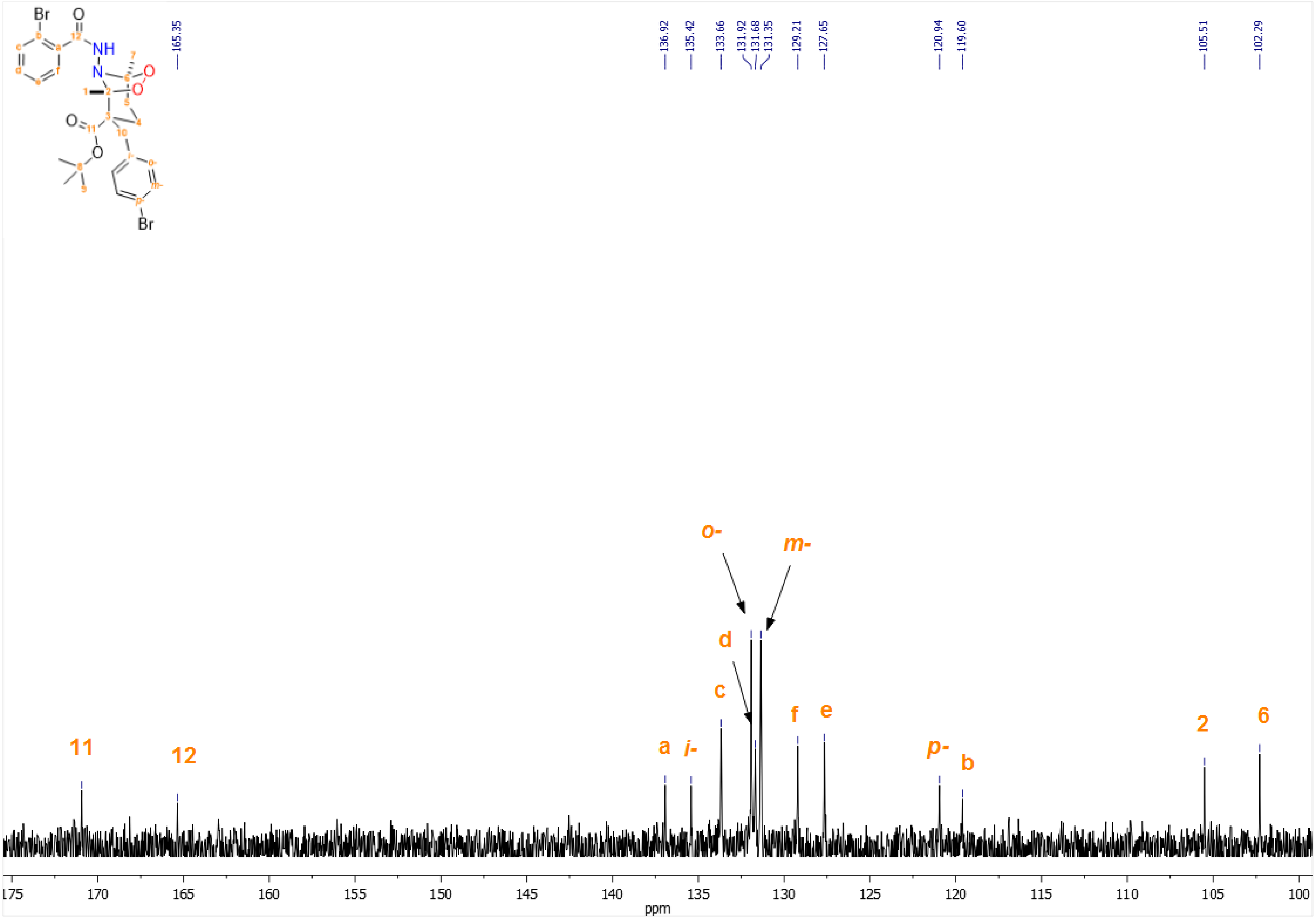

^{15}N (40.56 MHz, $CDCl_3$). *tert*-Butyl 8-(2-bromobenzamido)-2-(4-bromobenzyl)-1,5-dimethyl-6,7-dioxa-8-azabicyclo[3.2.1]octane-2-carboxylate, 18a

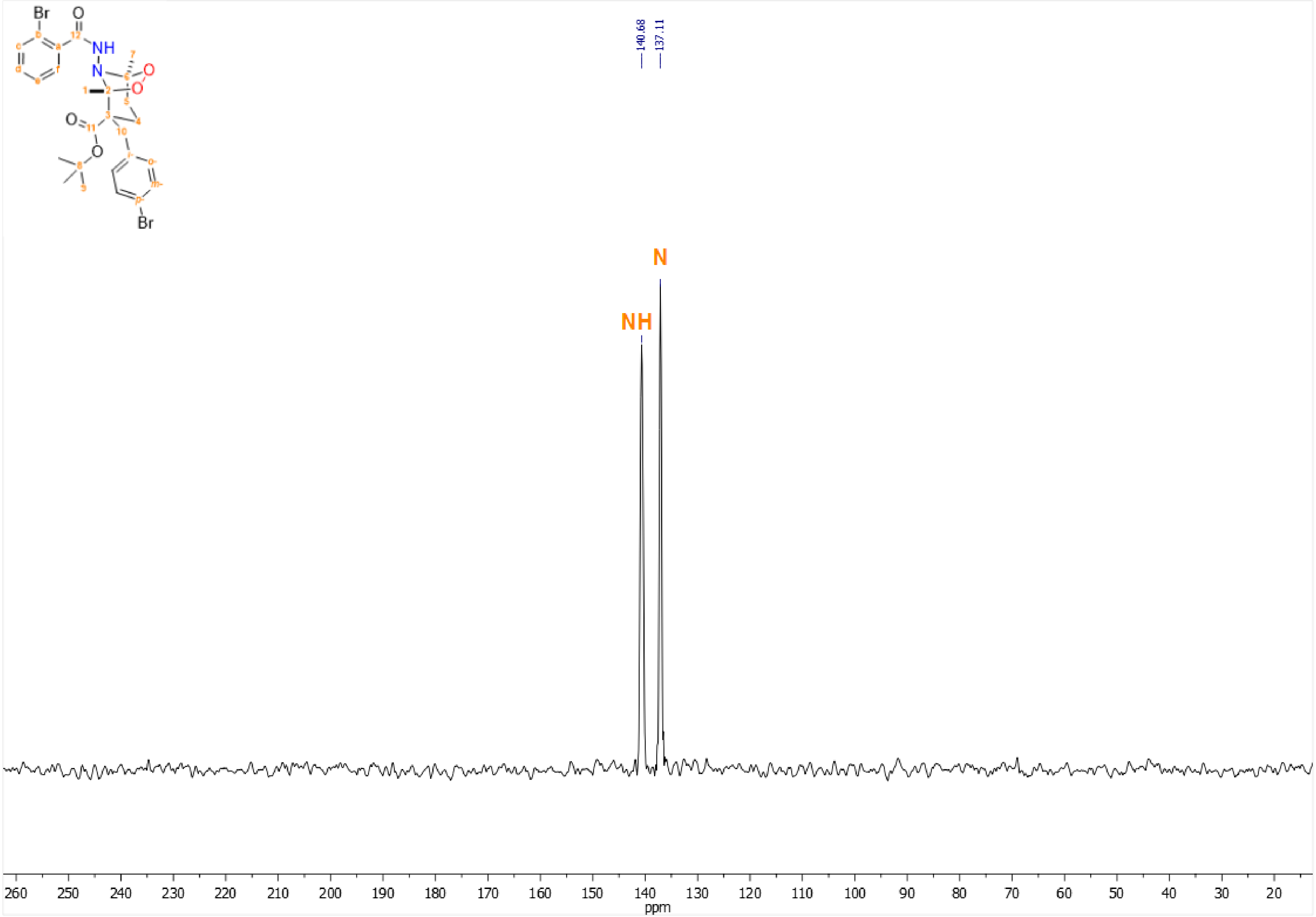

***tert*-Butyl 8-(2-bromobenzamido)-2-(4-bromobenzyl)-1,5-dimethyl-6,7-dioxa-8-azabicyclo[3.2.1]octane-2-carboxylate, 18a**

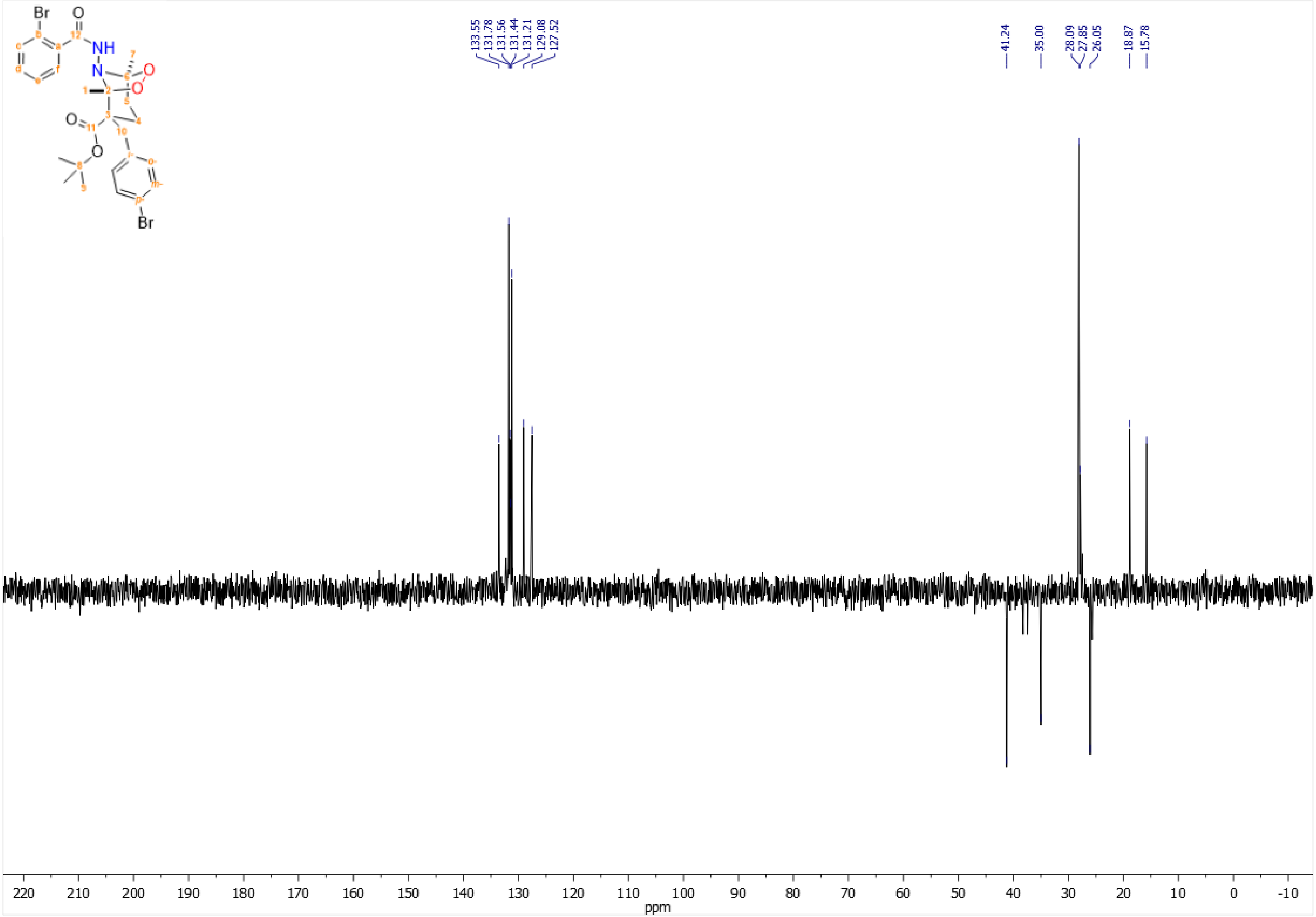

***tert*-Butyl 8-(2-bromobenzamido)-2-(4-bromobenzyl)-1,5-dimethyl-6,7-dioxa-8-azabicyclo[3.2.1]octane-2-carboxylate, 18a**

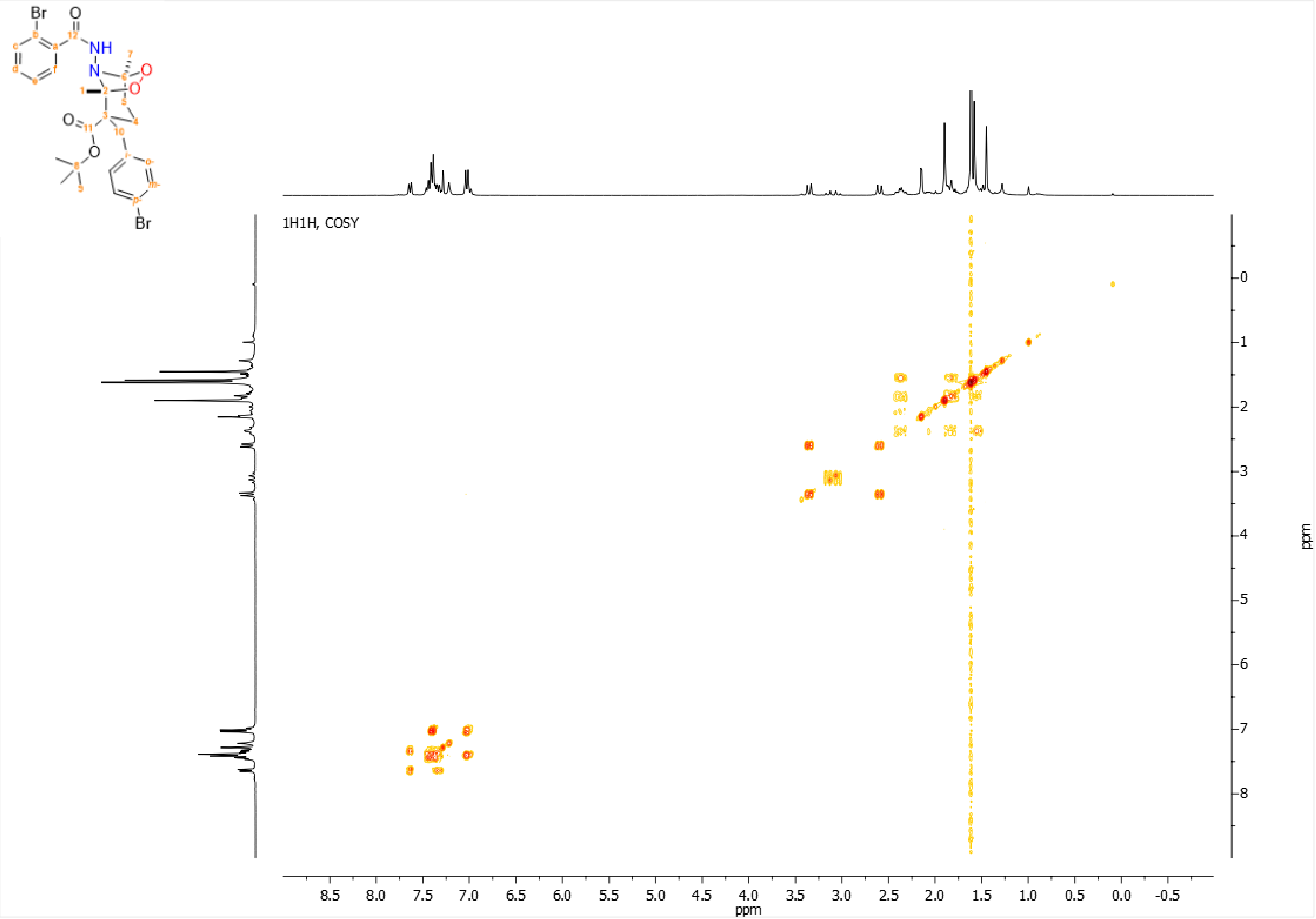

tert-Butyl 8-(2-bromobenzamido)-2-(4-bromobenzyl)-1,5-dimethyl-6,7-dioxa-8-azabicyclo[3.2.1]octane-2-carboxylate, 18a

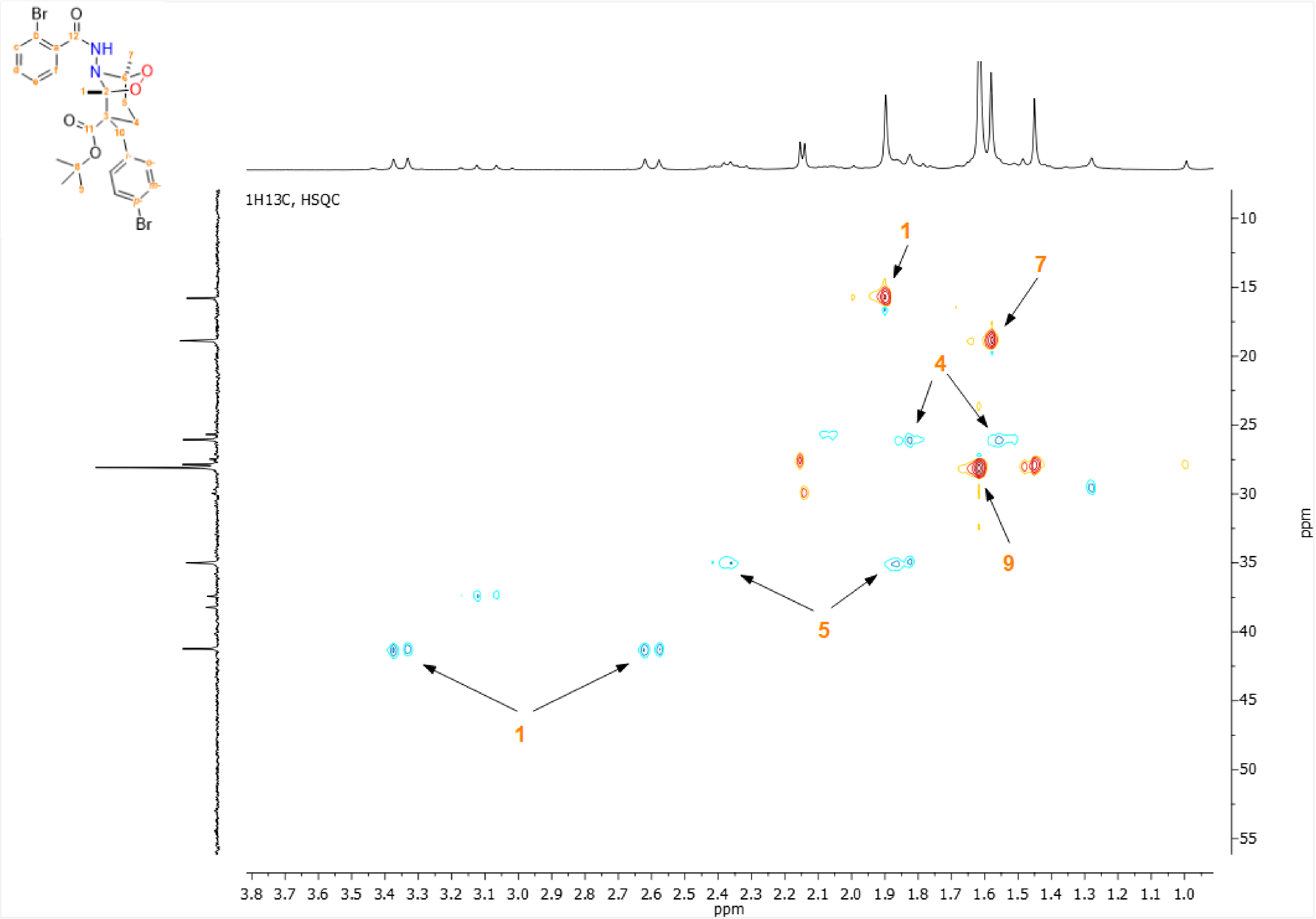

tert-Butyl 8-(2-bromobenzamido)-2-(4-bromobenzyl)-1,5-dimethyl-6,7-dioxa-8-azabicyclo[3.2.1]octane-2-carboxylate, 18a

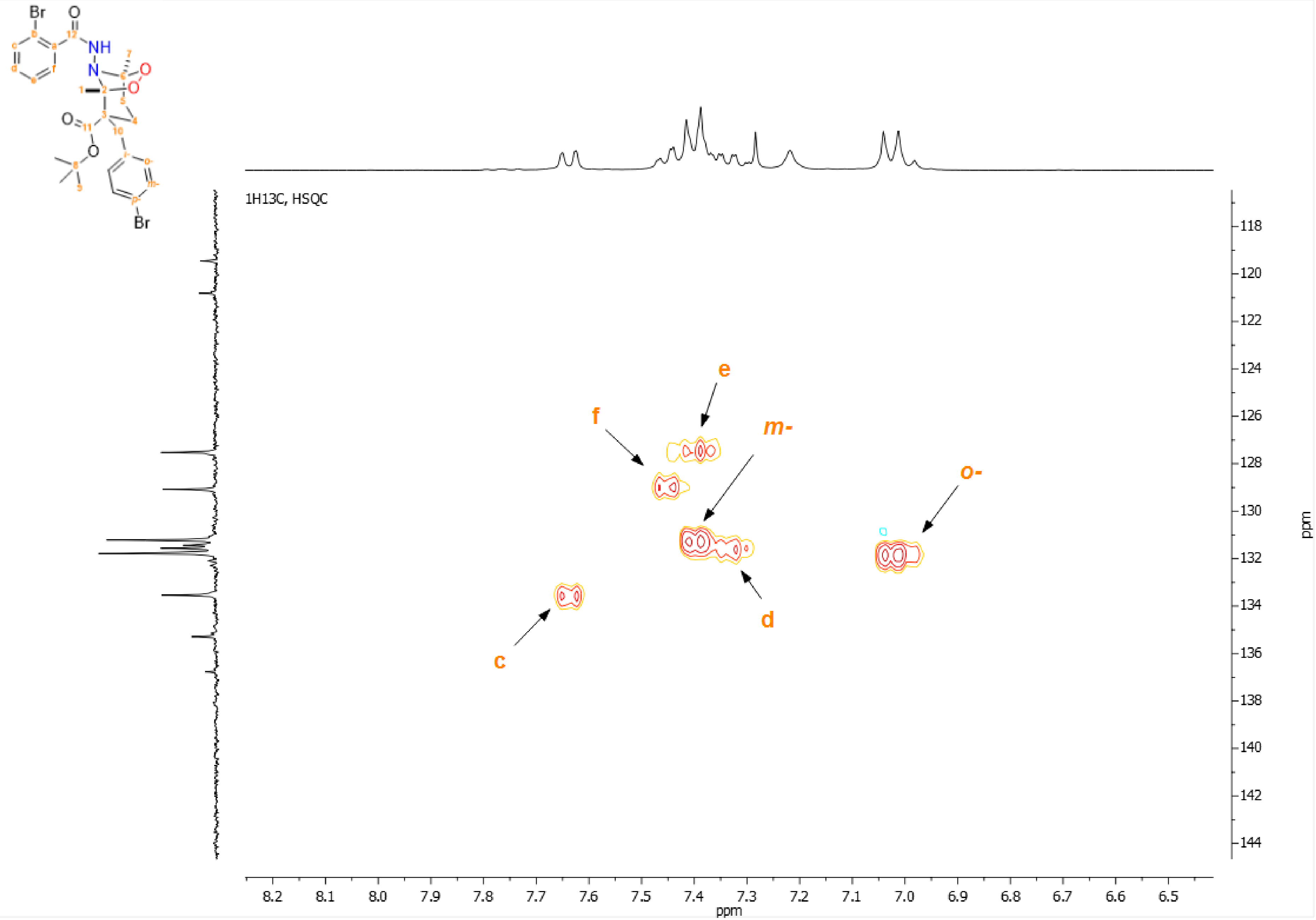

tert-Butyl 8-(2-bromobenzamido)-2-(4-bromobenzyl)-1,5-dimethyl-6,7-dioxa-8-azabicyclo[3.2.1]octane-2-carboxylate, 18a

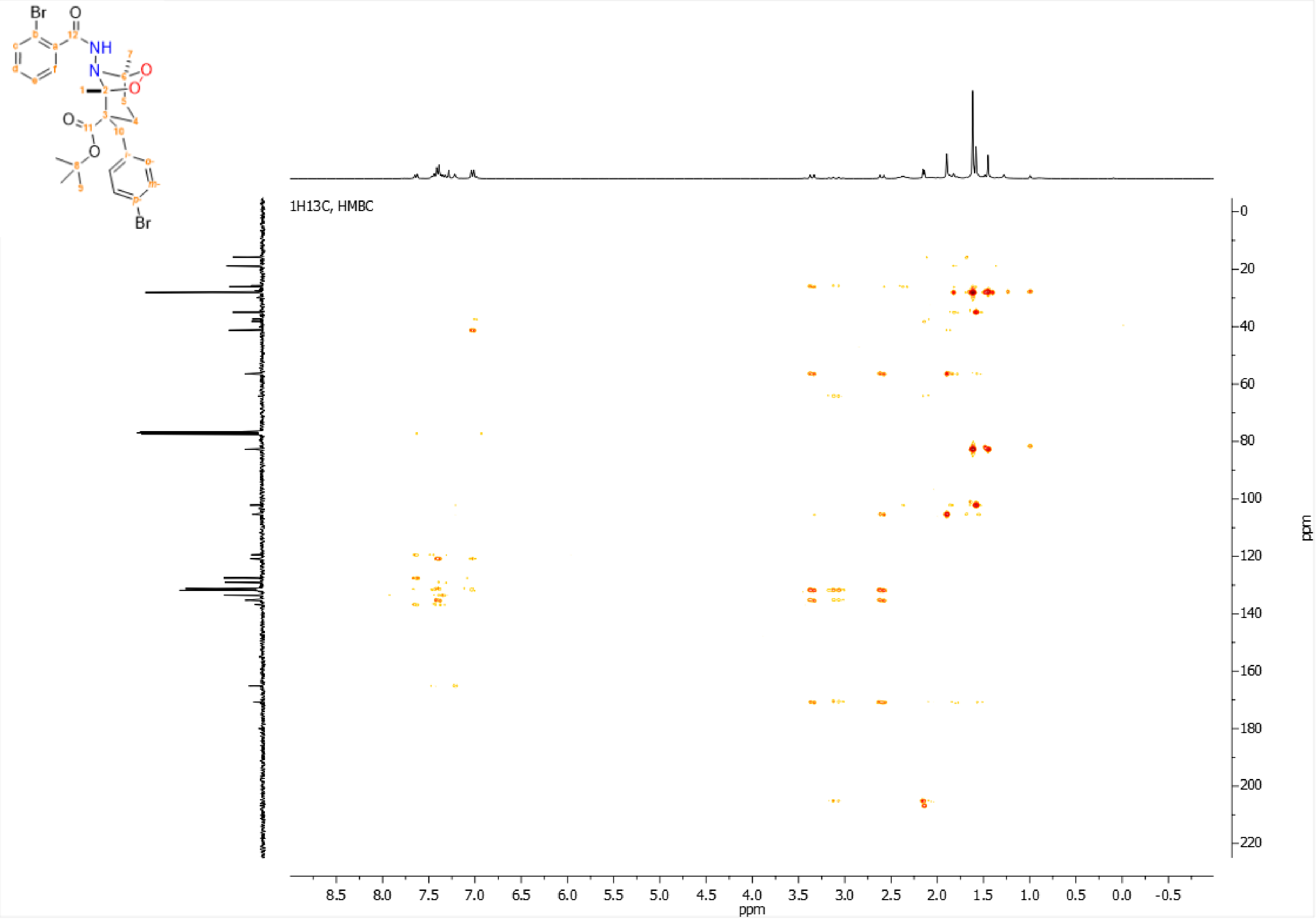

***tert*-Butyl 8-(2-bromobenzamido)-2-(4-bromobenzyl)-1,5-dimethyl-6,7-dioxa-8-azabicyclo[3.2.1]octane-2-carboxylate, 18a**

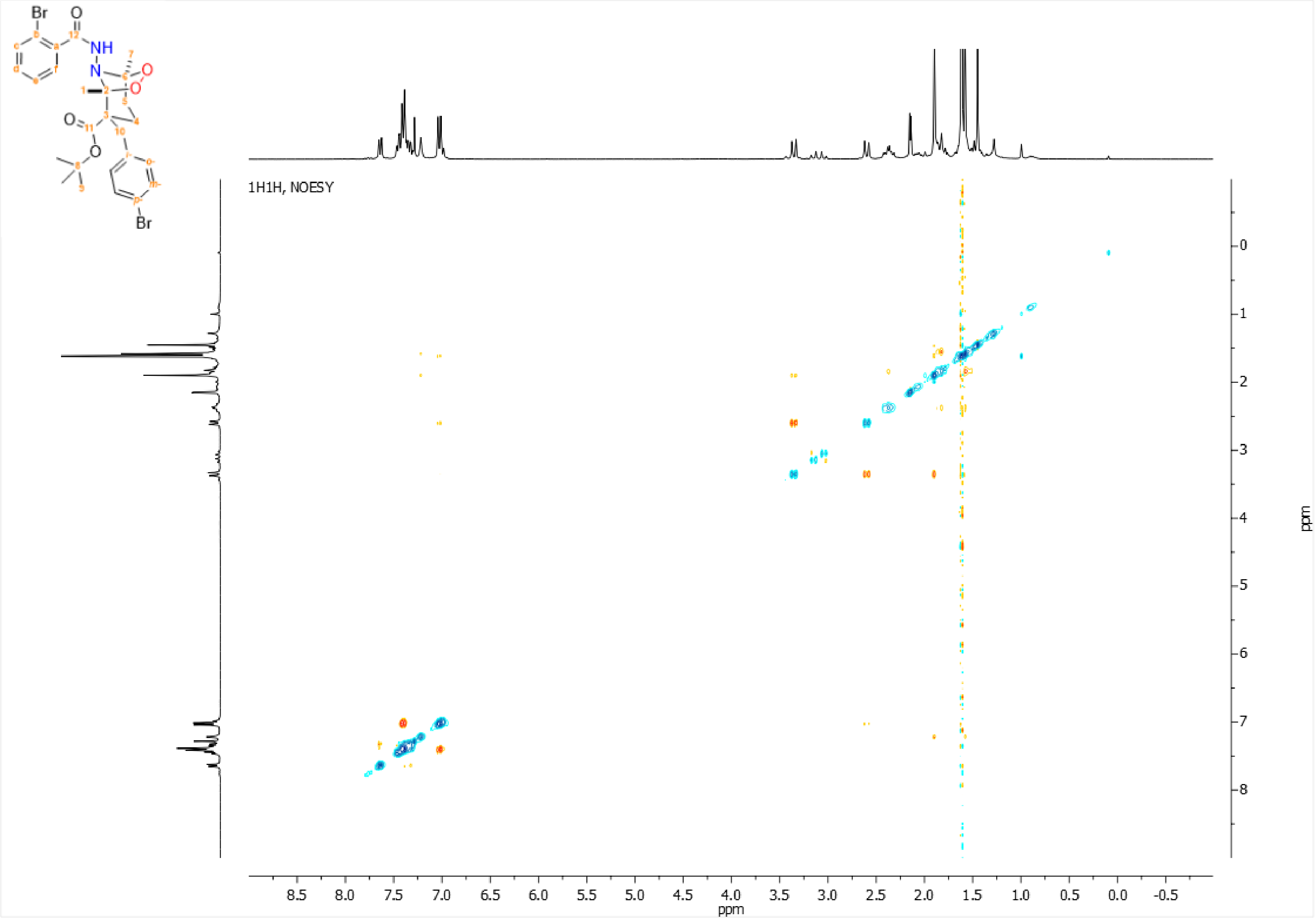

tert-Butyl 8-(2-bromobenzamido)-2-(4-bromobenzyl)-1,5-dimethyl-6,7-dioxa-8-azabicyclo[3.2.1]octane-2-carboxylate, 18a

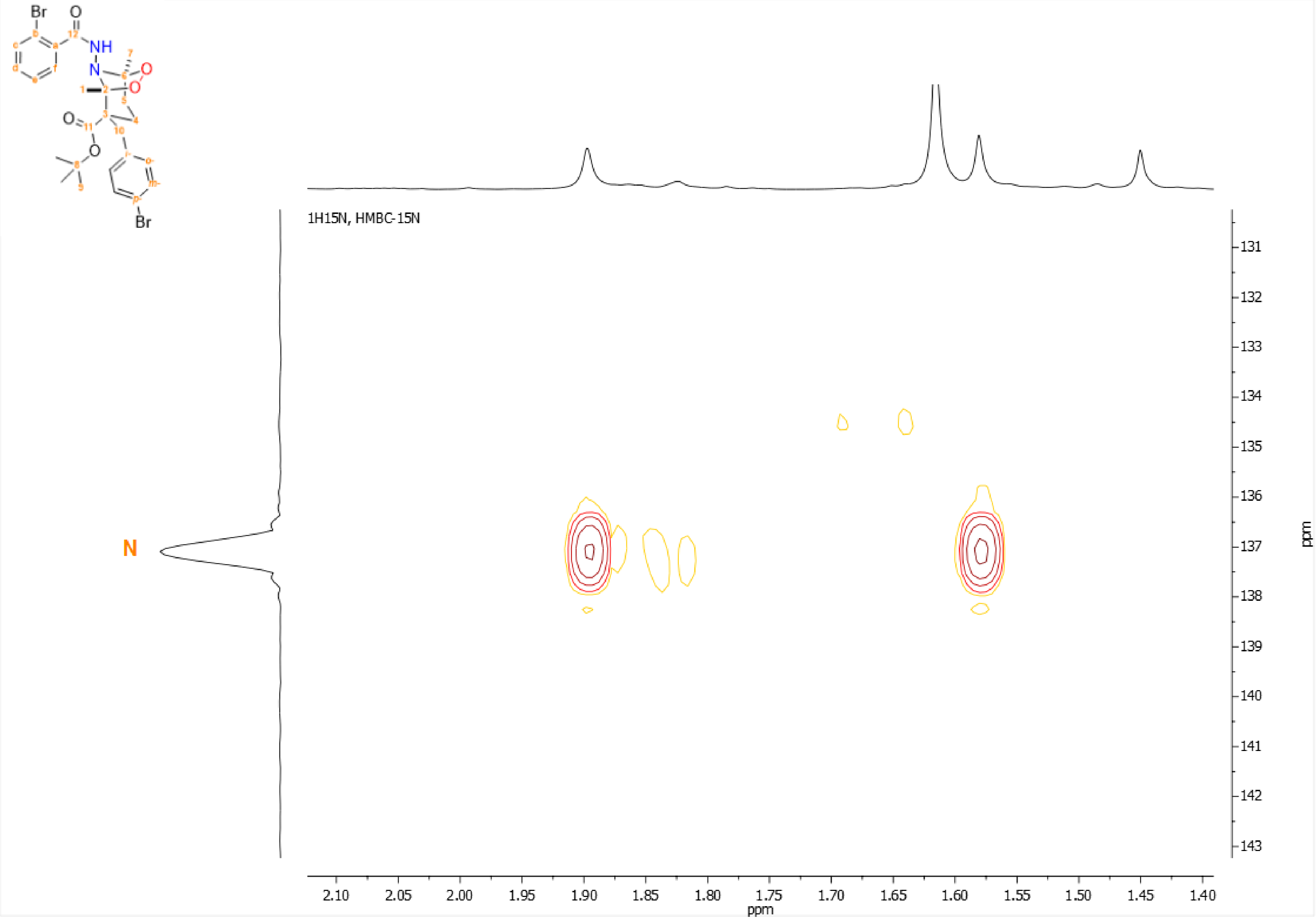

tert-Butyl 8-(2-bromobenzamido)-2-(4-bromobenzyl)-1,5-dimethyl-6,7-dioxa-8-azabicyclo[3.2.1]octane-2-carboxylate, 18a

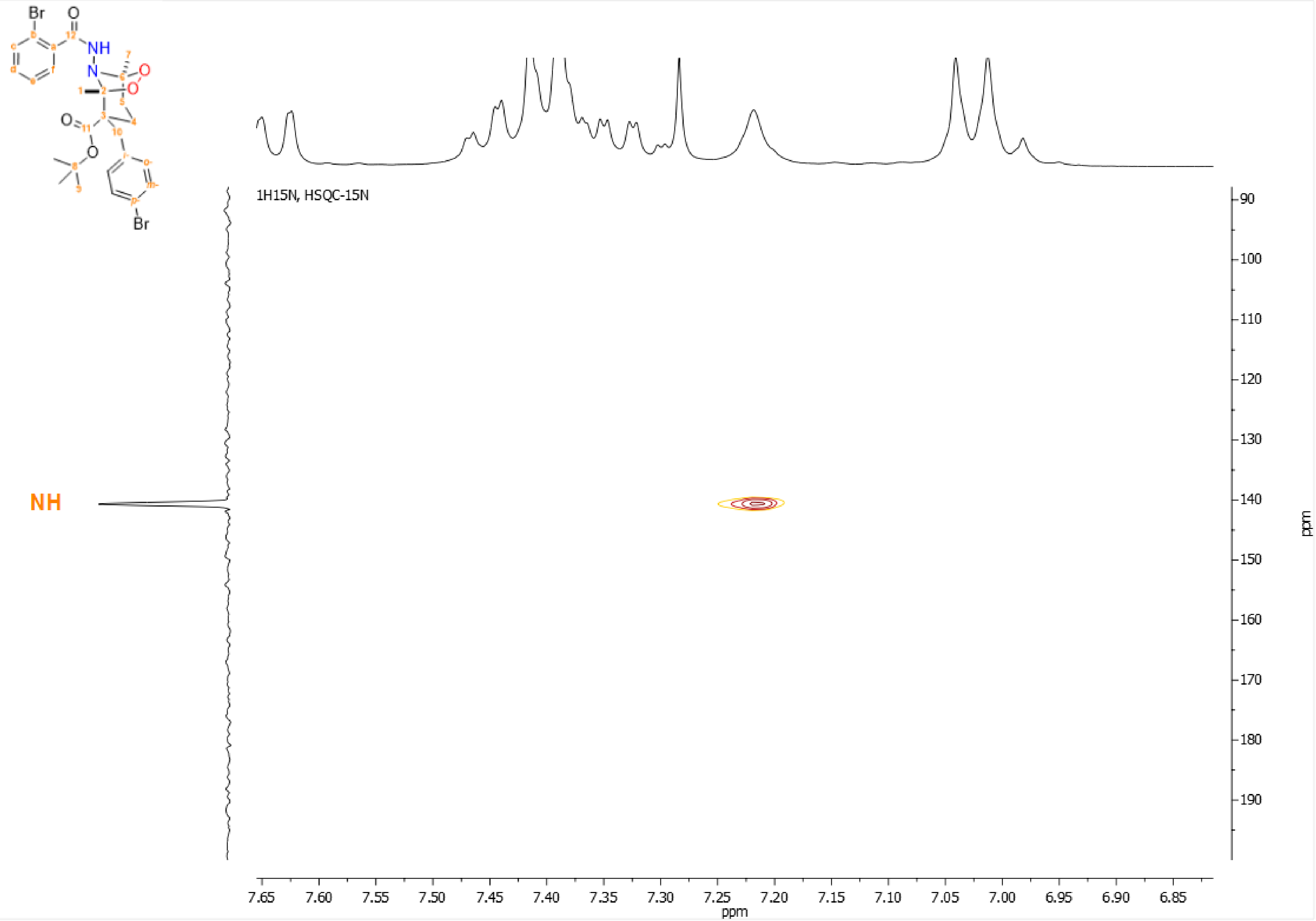

^{1}H NMR (300.13 MHz, CDCl3). Ethyl 8-(2-bromobenzamido)-2-(4-fluorobenzyl)-1,5-dimethyl-6,7-dioxa-8-azabicyclo[3.2.1]octane-2-carboxylate, 19a + 19b

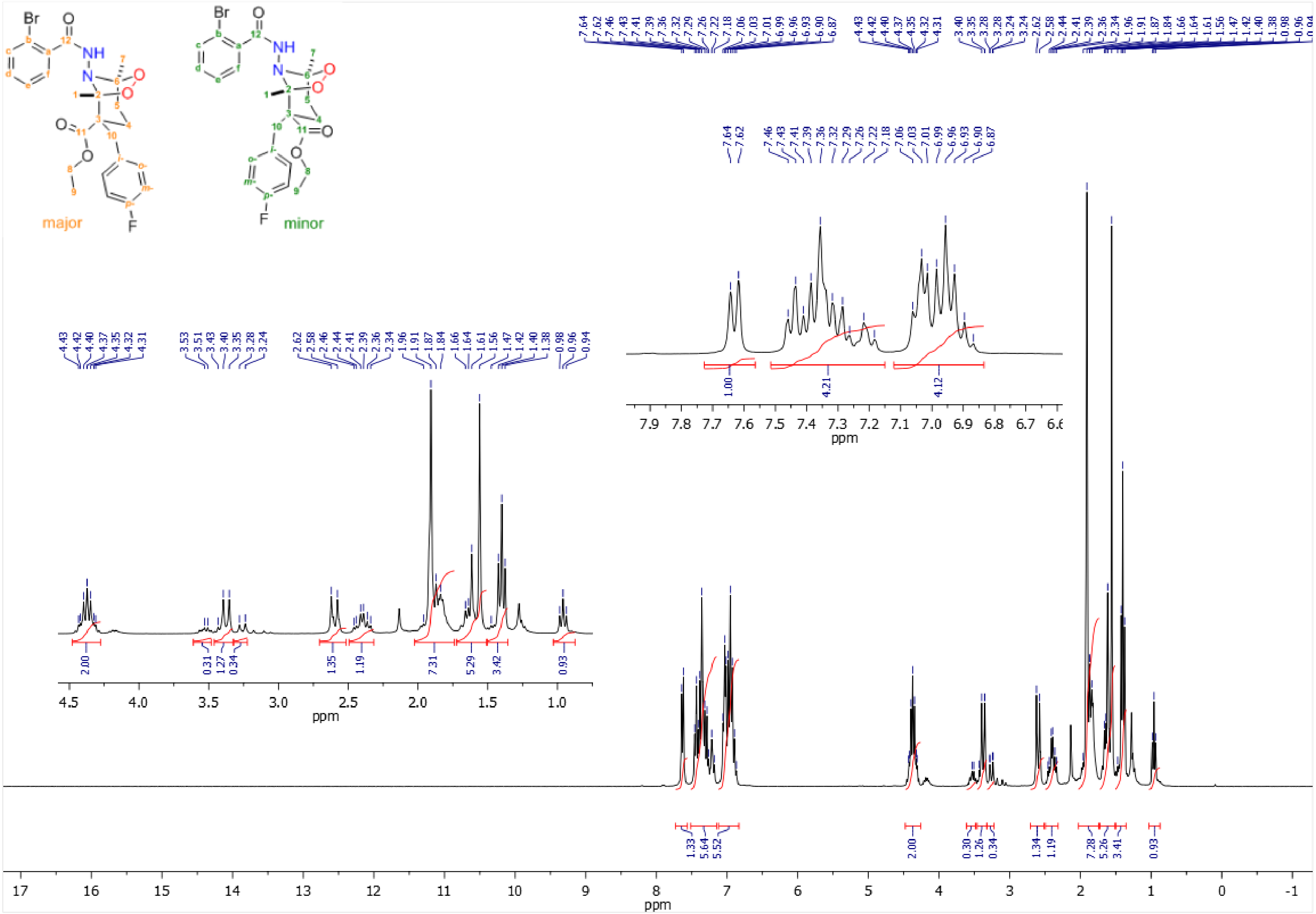

^{1}H NMR (300.13 MHz, $CDCl_3$). Ethyl 8-(2-bromobenzamido)-2-(4-fluorobenzyl)-1,5-dimethyl-6,7-dioxa-8-azabicyclo[3.2.1]octane-2-carboxylate, 19a + 19b

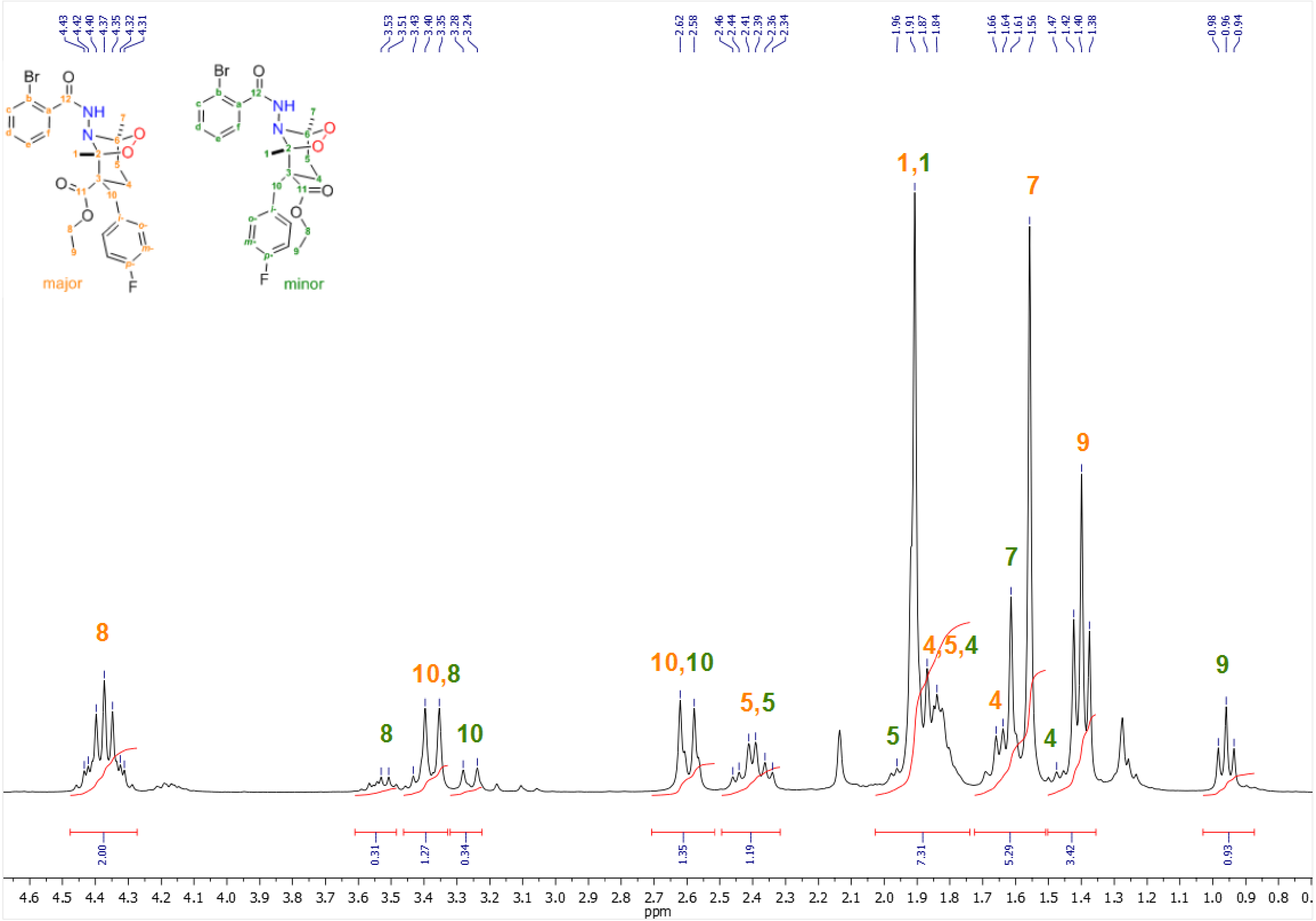

^{1}H NMR (300.13 MHz, CDCl3). Ethyl 8-(2-bromobenzamido)-2-(4-fluorobenzyl)-1,5-dimethyl-6,7-dioxa-8-azabicyclo[3.2.1]octane-2-carboxylate, 19a + 19b

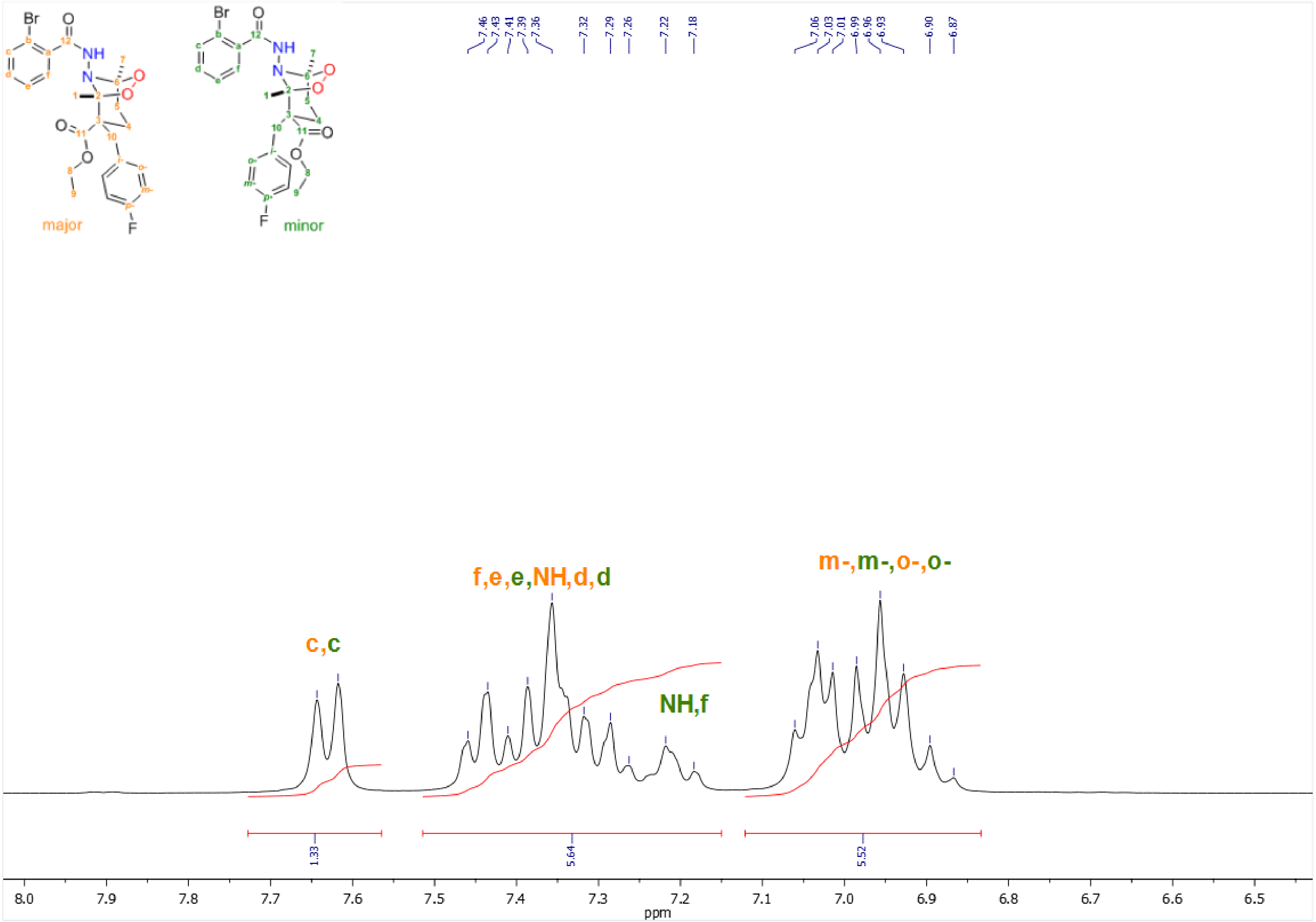

^{13}C NMR (75.48 MHz, $CDCl_3$). Ethyl 8-(2-bromobenzamido)-2-(4-fluorobenzyl)-1,5-dimethyl-6,7-dioxa-8-azabicyclo[3.2.1]octane-2-carboxylate, 19a + 19b

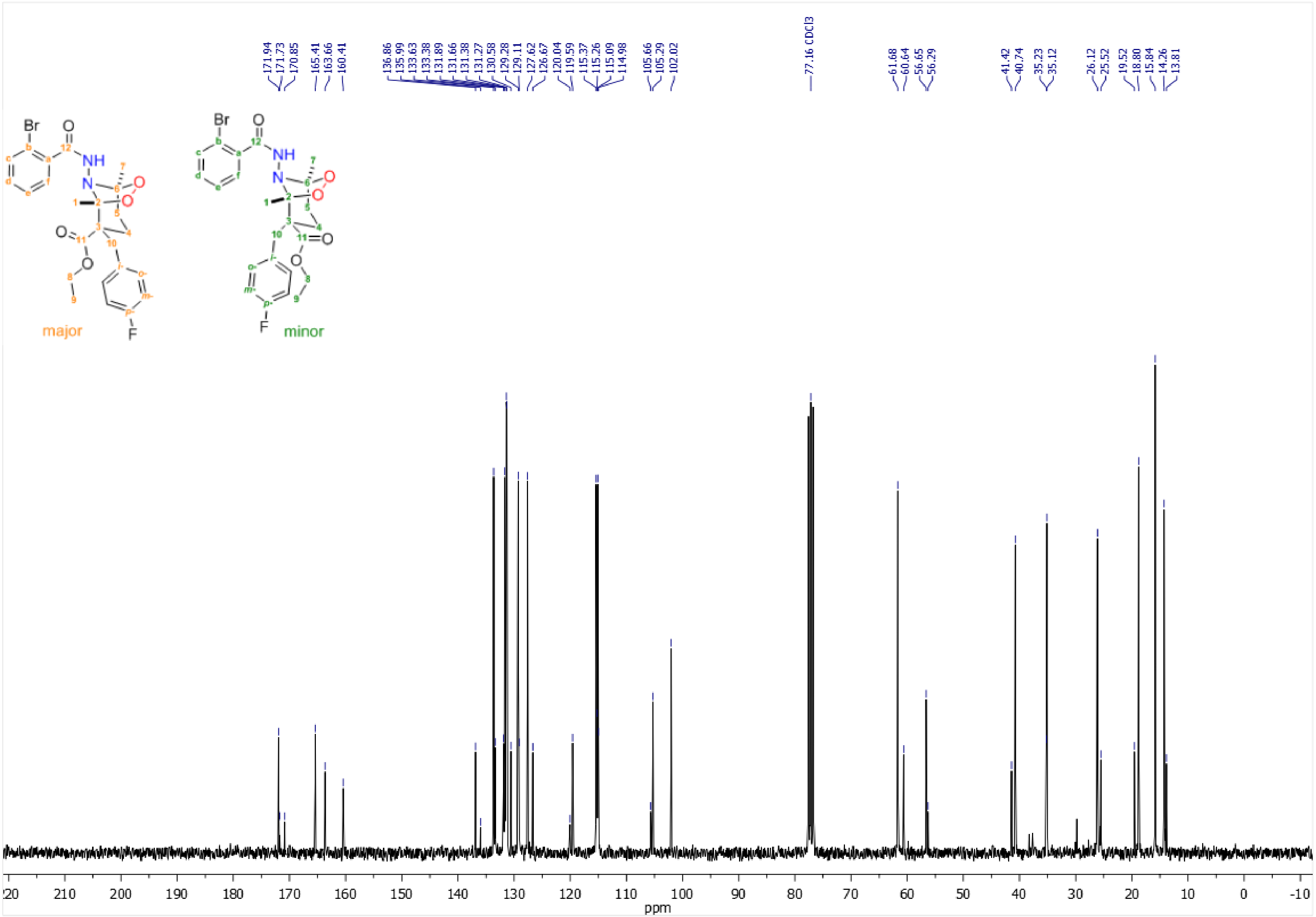

^{13}C NMR (75.48 MHz, CDCl3). Ethyl 8-(2-bromobenzamido)-2-(4-fluorobenzyl)-1,5-dimethyl-6,7-dioxa-8-azabicyclo[3.2.1]octane-2-carboxylate, 19a + 19b

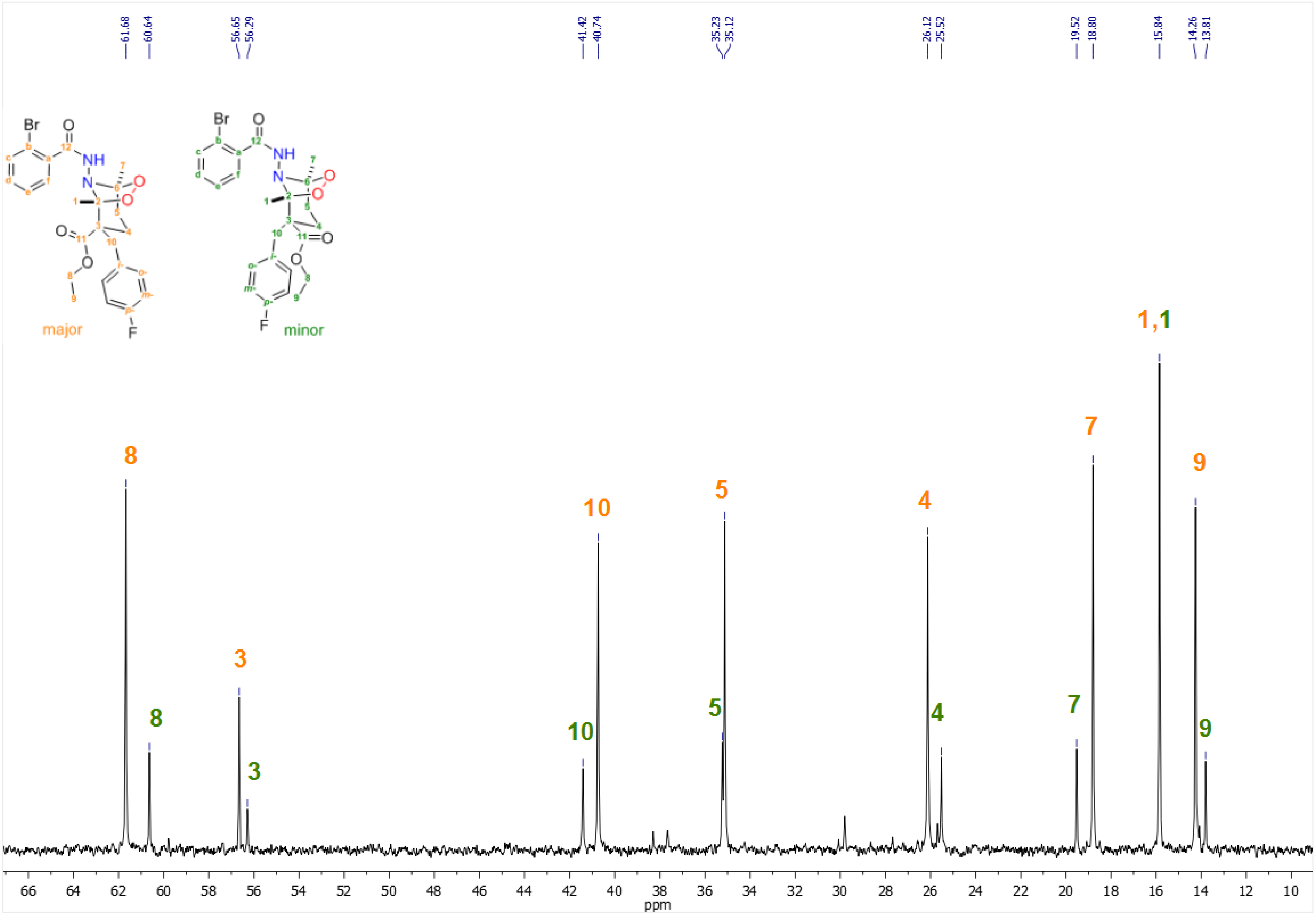

^{13}C NMR (75.48 MHz, CDCl$_3$). Ethyl 8-(2-bromobenzamido)-2-(4-fluorobenzyl)-1,5-dimethyl-6,7-dioxa-8-azabicyclo[3.2.1]octane-2-carboxylate, 19a + 19b

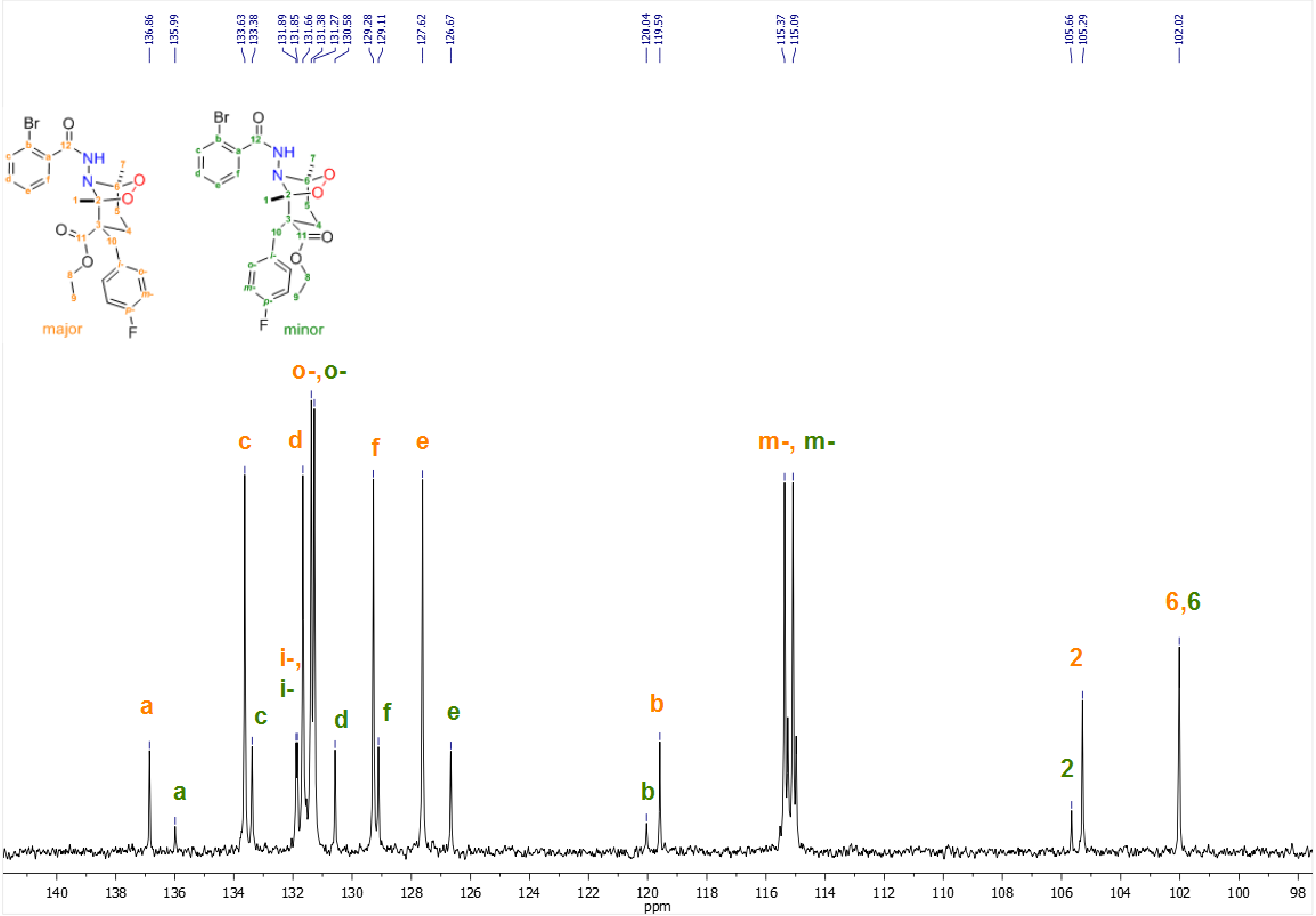

^{13}C NMR (75.48 MHz, CDCl$_3$). Ethyl 8-(2-bromobenzamido)-2-(4-fluorobenzyl)-1,5-dimethyl-6,7-dioxa-8-azabicyclo[3.2.1]octane-2-carboxylate, 19a + 19b

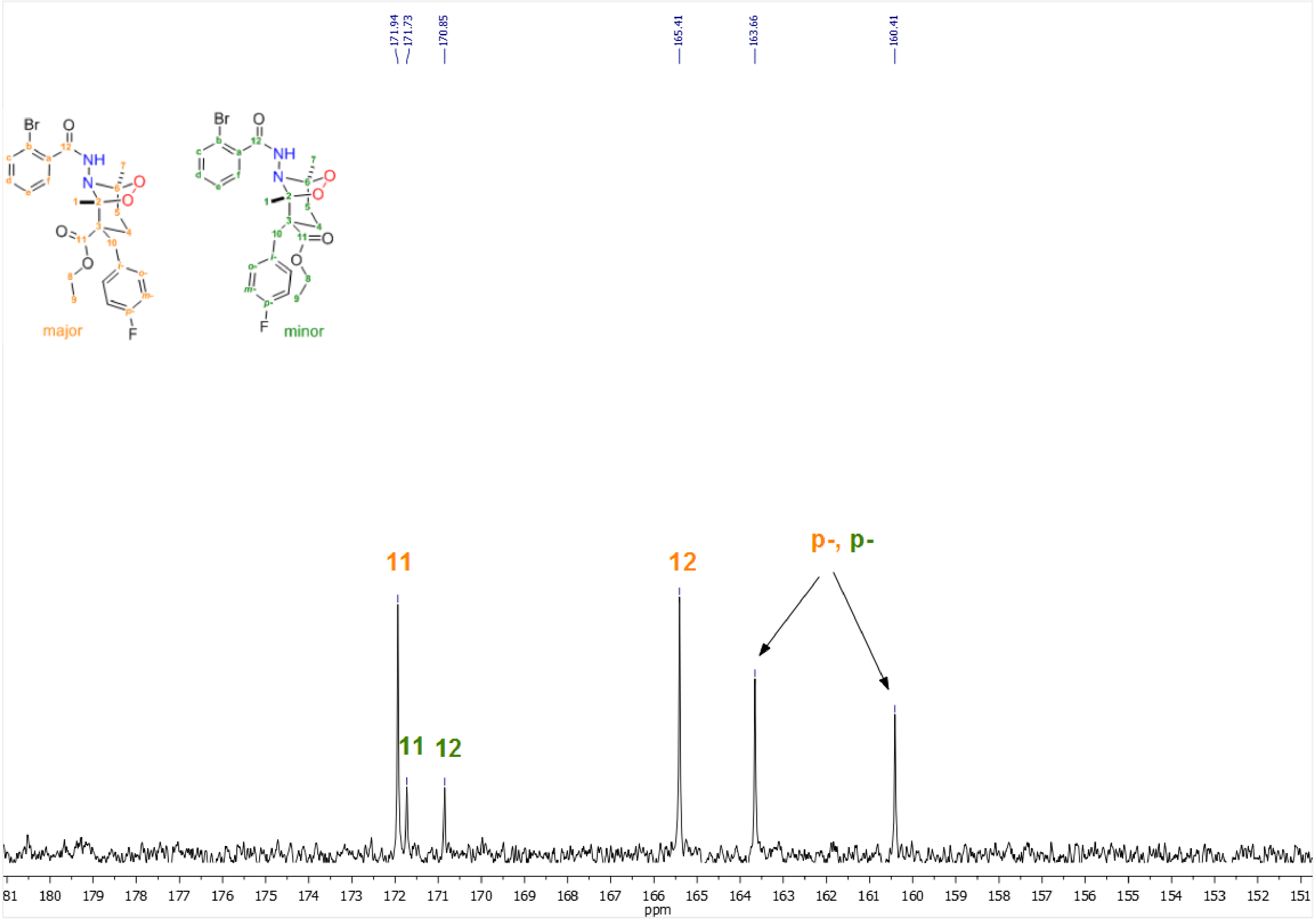

^{15}N (40.56 MHz, $CDCl_3$).Ethyl 8-(2-bromobenzamido)-2-(4-fluorobenzyl)-1,5-dimethyl-6,7-dioxa-8-azabicyclo[3.2.1]octane-2-carboxylate, 19a + 19b

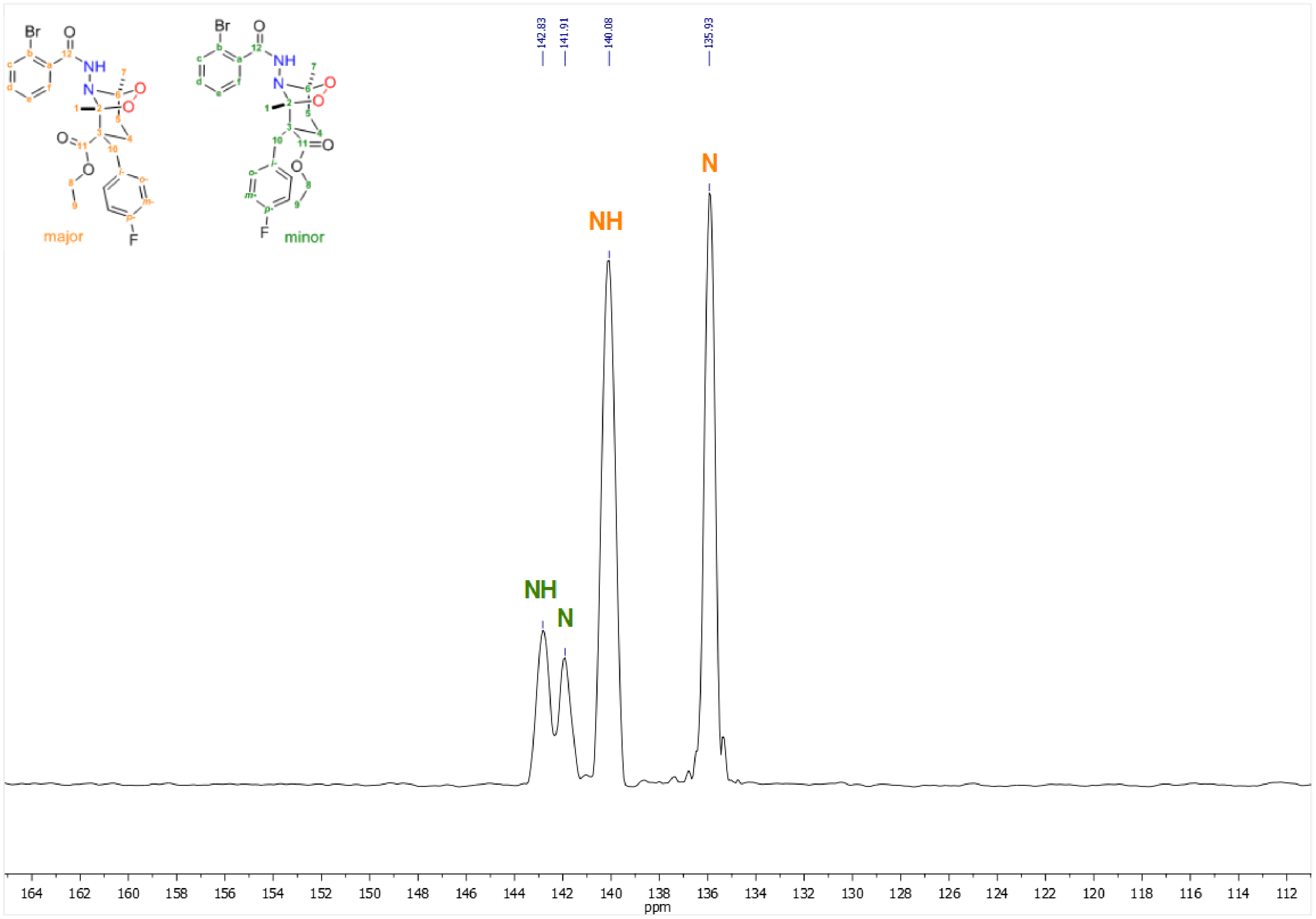

^{13}C NMR (75.48 MHz, CDCl3). Ethyl 8-(2-bromobenzamido)-2-(4-fluorobenzyl)-1,5-dimethyl-6,7-dioxa-8-azabicyclo[3.2.1]octane-2-carboxylate, 19a + 19b

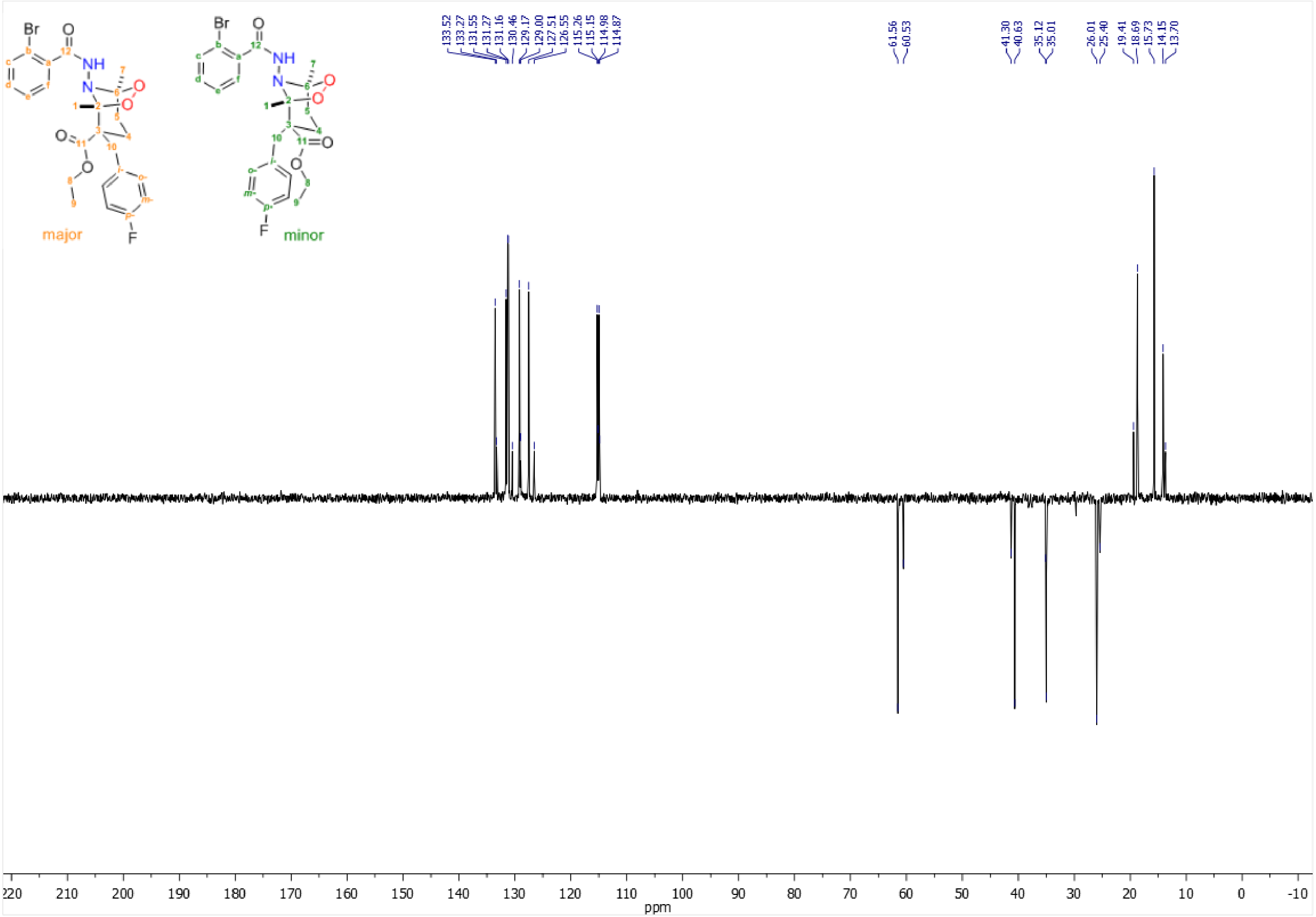

Ethyl 8-(2-bromobenzamido)-2-(4-fluorobenzyl)-1,5-dimethyl-6,7-dioxa-8-azabicyclo[3.2.1]octane-2-carboxylate, 19a + 19b

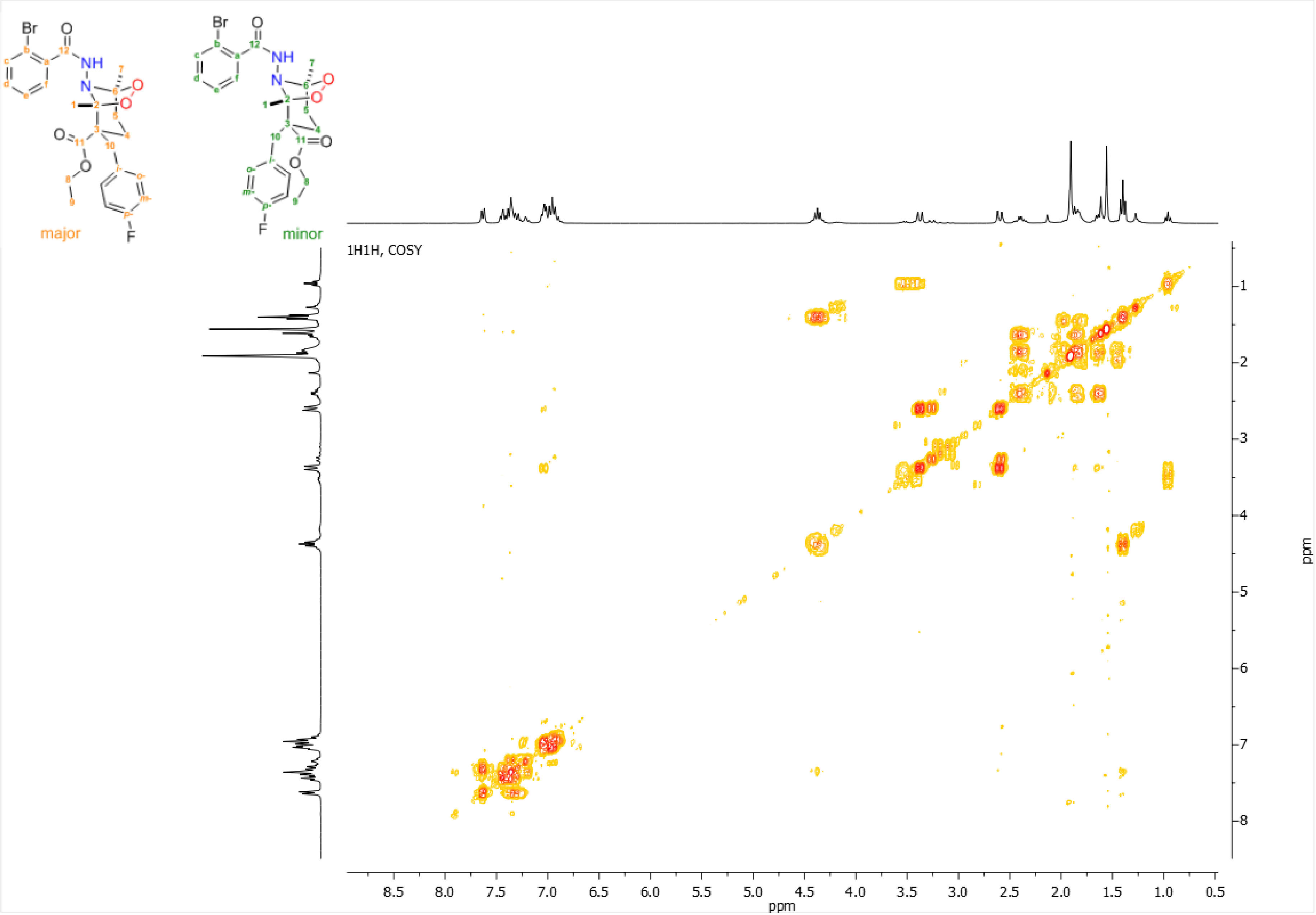

Ethyl 8-(2-bromobenzamido)-2-(4-fluorobenzyl)-1,5-dimethyl-6,7-dioxa-8-azabicyclo[3.2.1]octane-2-carboxylate, 19a + 19b

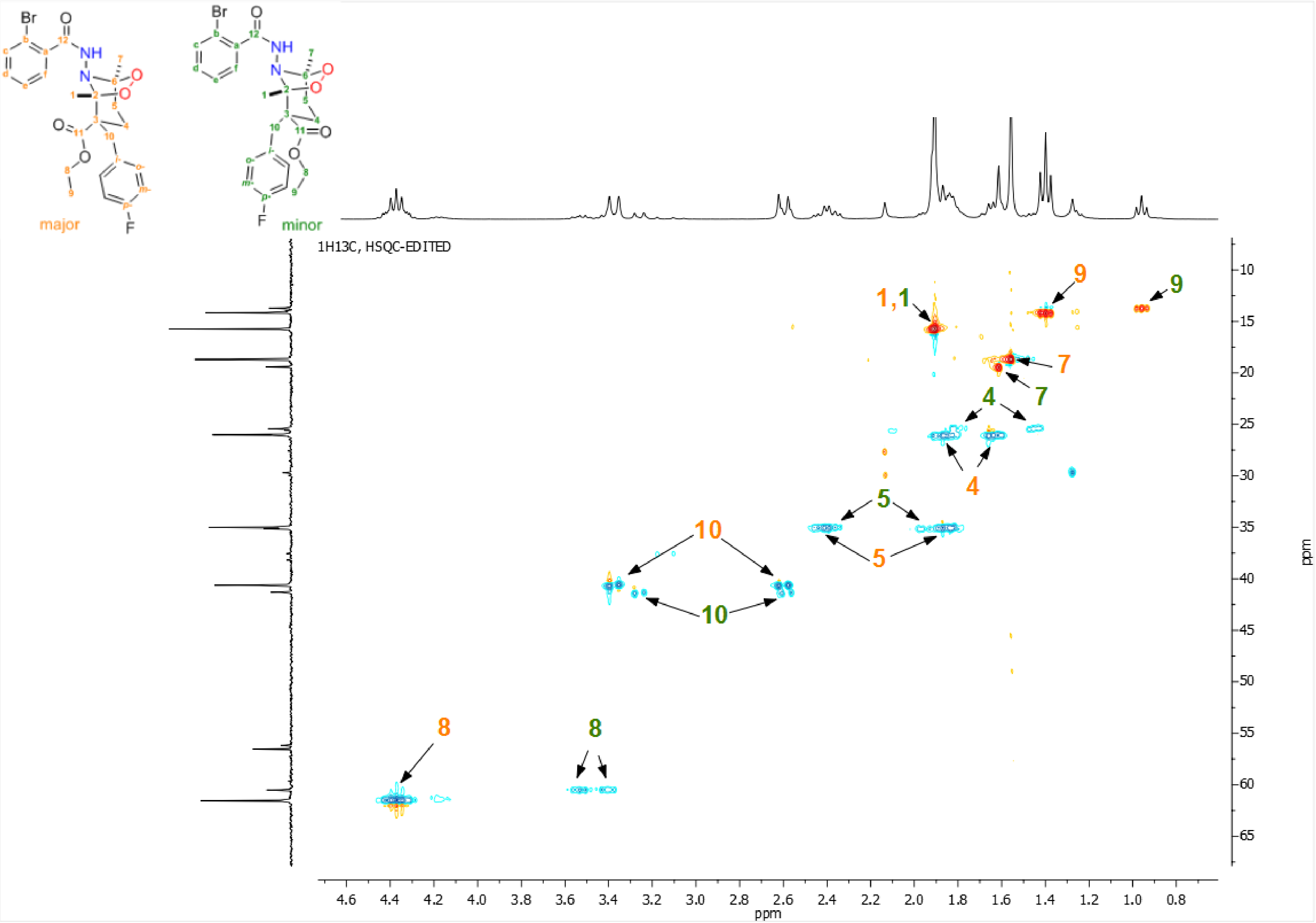

Ethyl 8-(2-bromobenzamido)-2-(4-fluorobenzyl)-1,5-dimethyl-6,7-dioxa-8-azabicyclo[3.2.1]octane-2-carboxylate, 19a + 19b

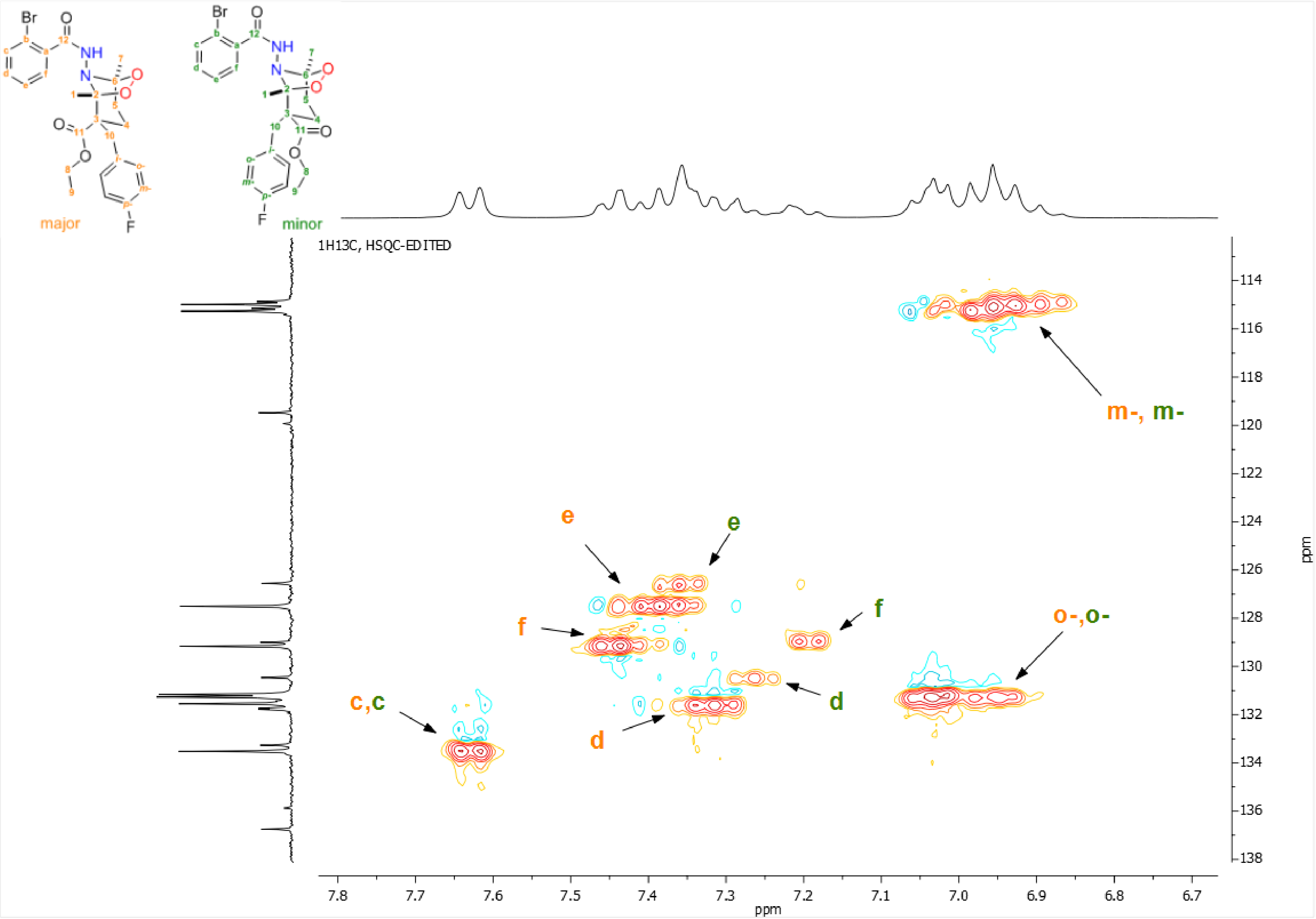

Ethyl 8-(2-bromobenzamido)-2-(4-fluorobenzyl)-1,5-dimethyl-6,7-dioxa-8-azabicyclo[3.2.1]octane-2-carboxylate, 19a + 19b

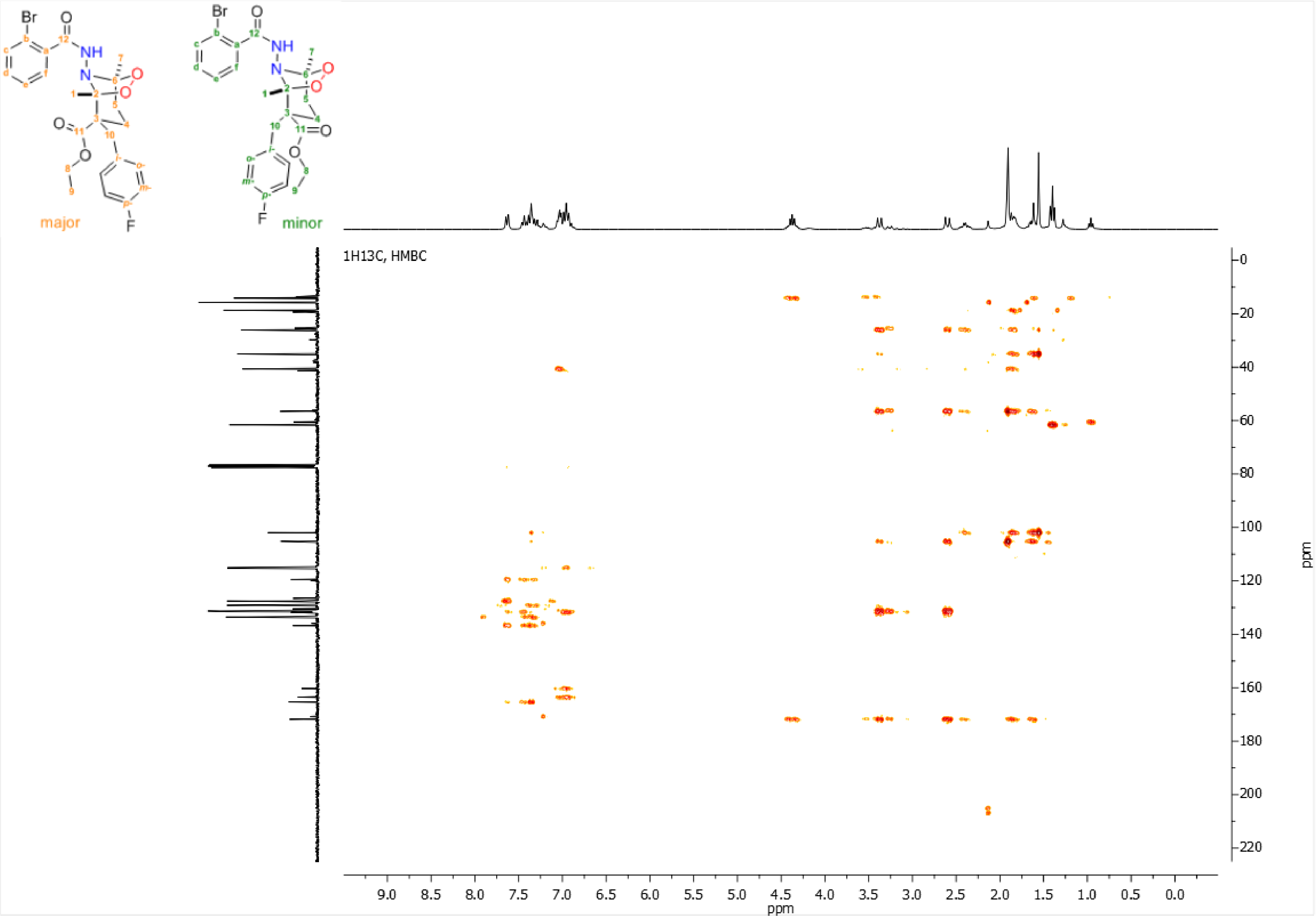

Ethyl 8-(2-bromobenzamido)-2-(4-fluorobenzyl)-1,5-dimethyl-6,7-dioxa-8-azabicyclo[3.2.1]octane-2-carboxylate, 19a + 19b

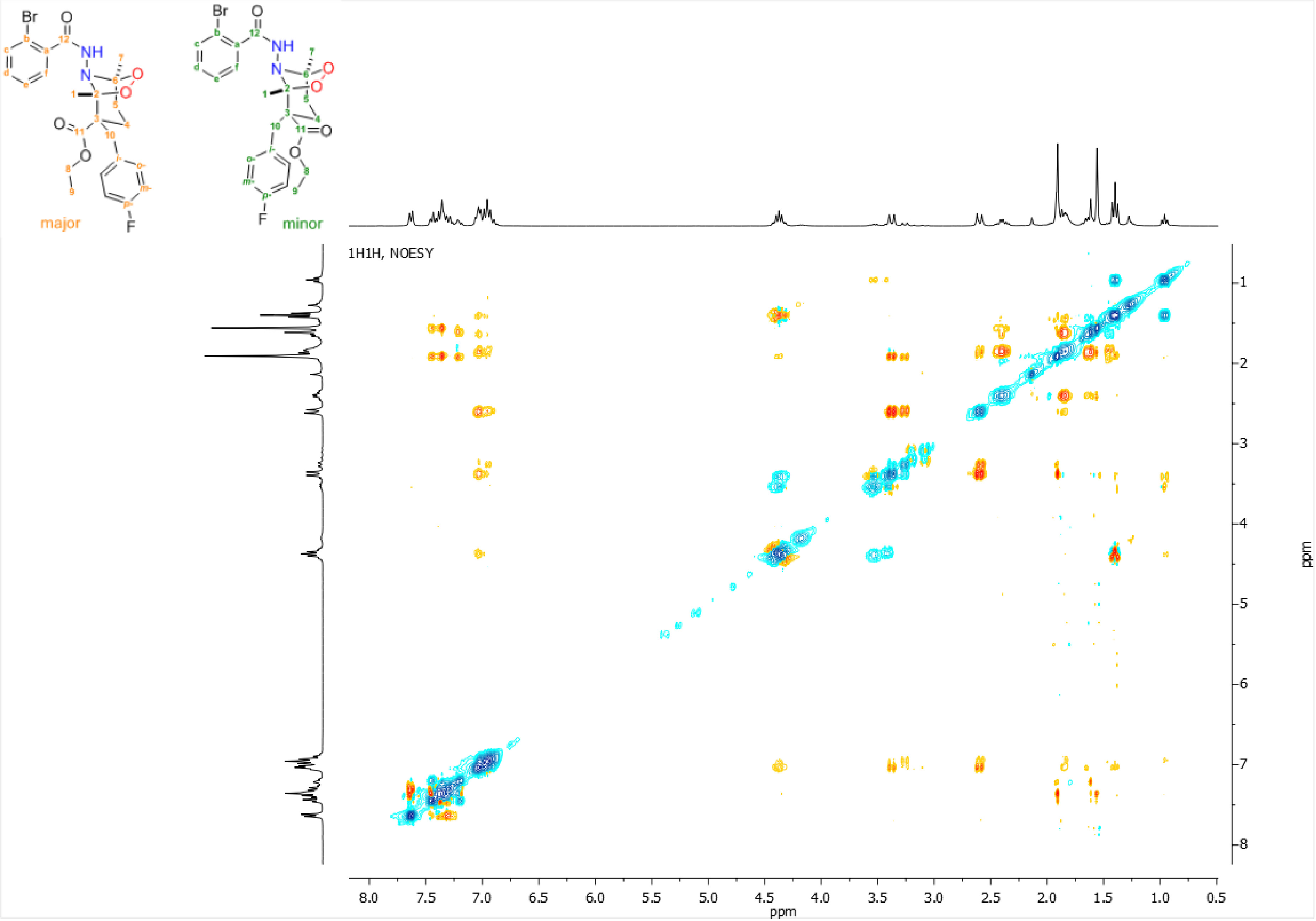

Ethyl 8-(2-bromobenzamido)-2-(4-fluorobenzyl)-1,5-dimethyl-6,7-dioxa-8-azabicyclo[3.2.1]octane-2-carboxylate, 19a + 19b

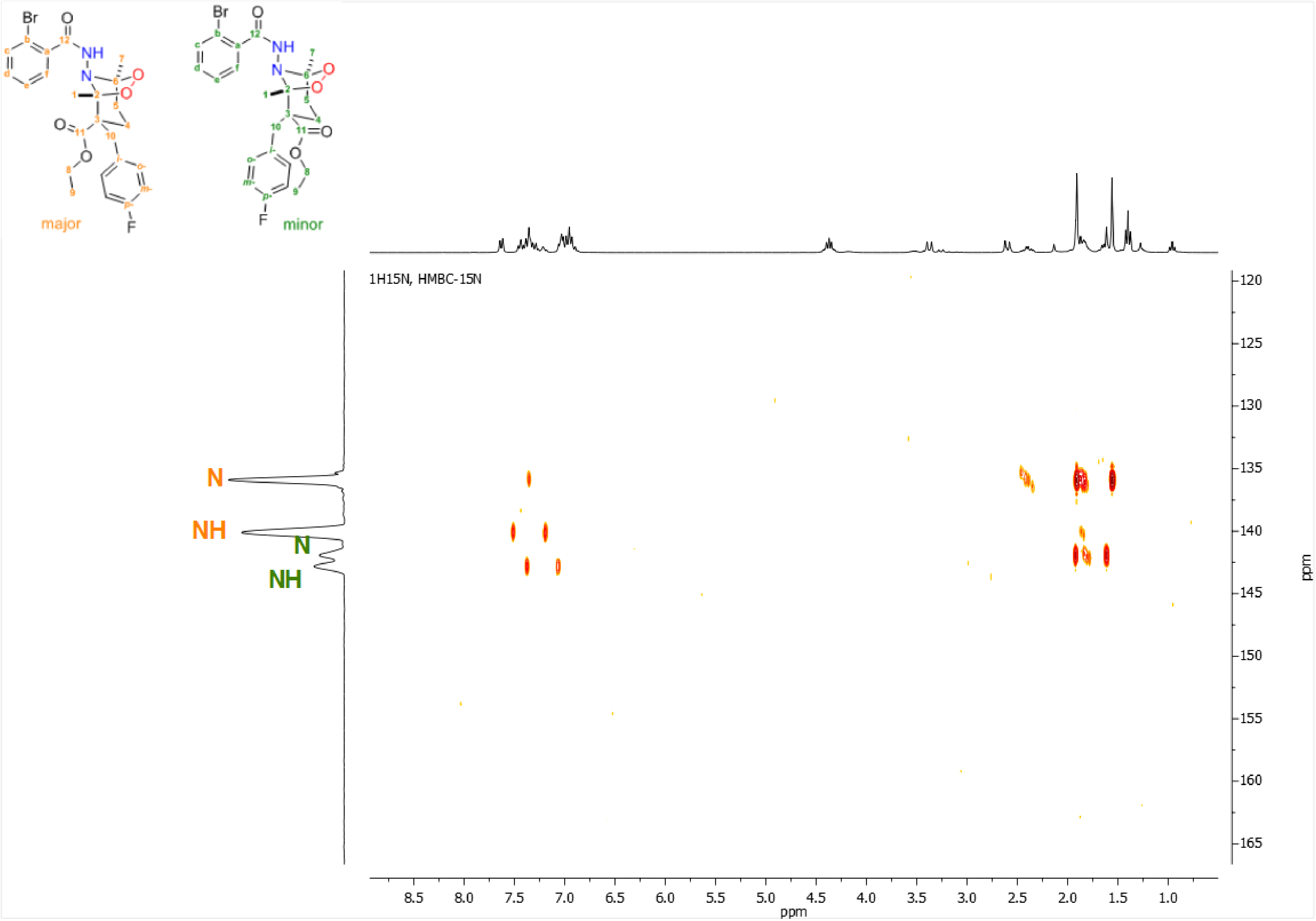

Ethyl 8-(2-bromobenzamido)-2-(4-fluorobenzyl)-1,5-dimethyl-6,7-dioxa-8-azabicyclo[3.2.1]octane-2-carboxylate, 19a + 19b

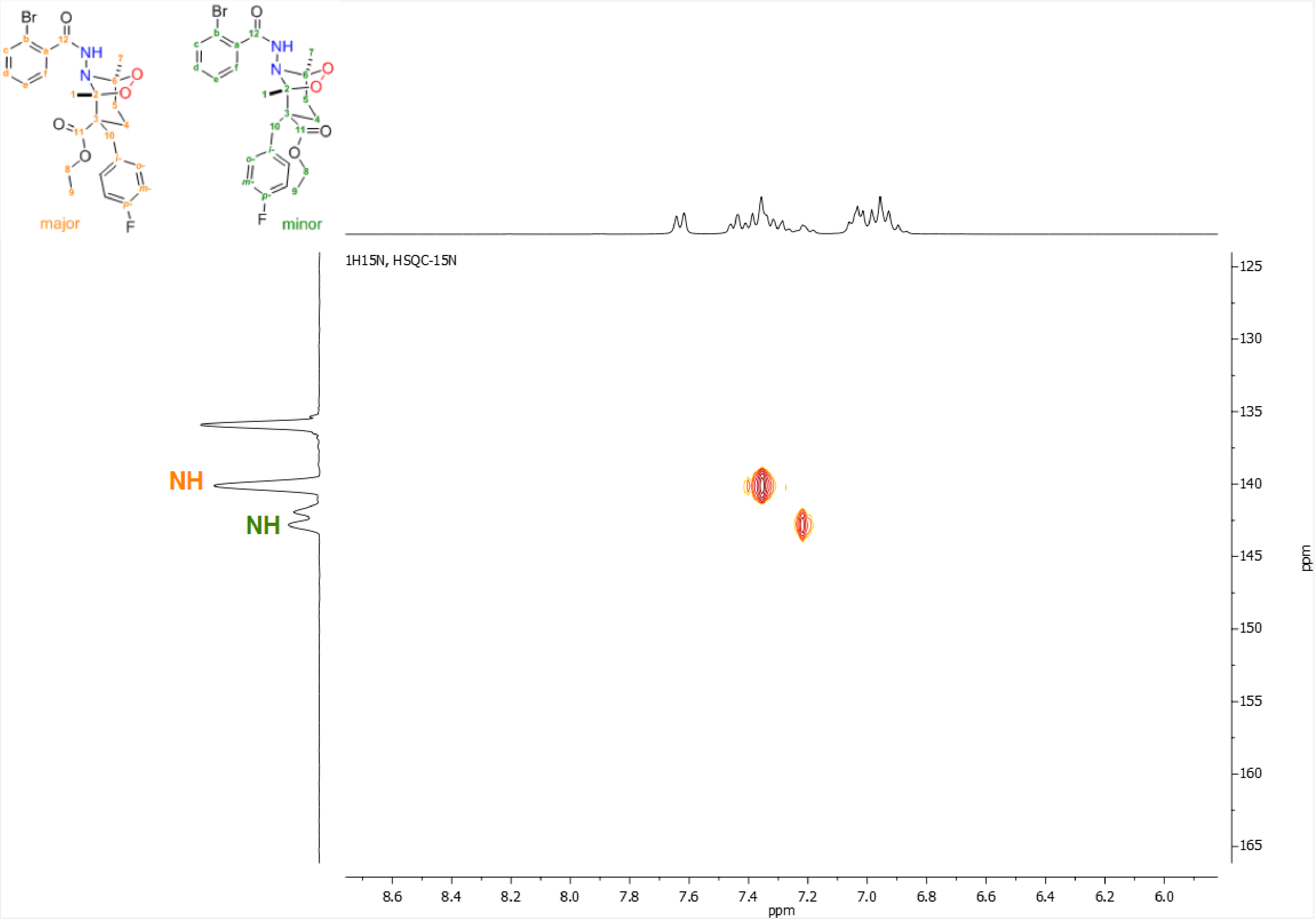

^{1}H NMR (300.13 MHz, $CDCl_3$). Ethyl 2-benzyl-8-(2-bromobenzamido)-1,5-dimethyl-6,7-dioxa-8-azabicyclo[3.2.1]octane-2-carboxylate, 20a + 20b

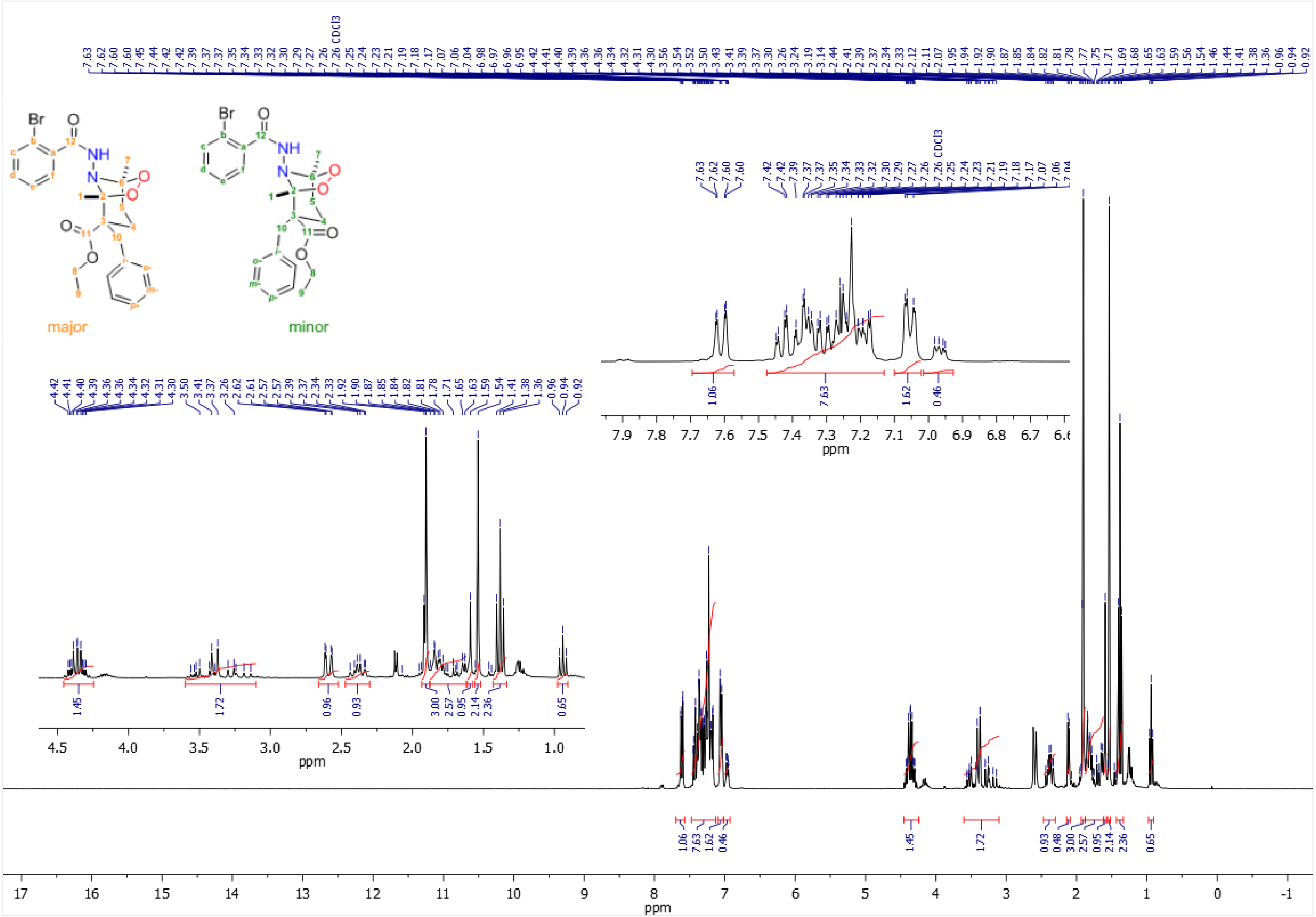

^{1}H NMR (300.13 MHz, $CDCl_3$). Ethyl 2-benzyl-8-(2-bromobenzamido)-1,5-dimethyl-6,7-dioxa-8-azabicyclo[3.2.1]octane-2-carboxylate, 20a + 20b

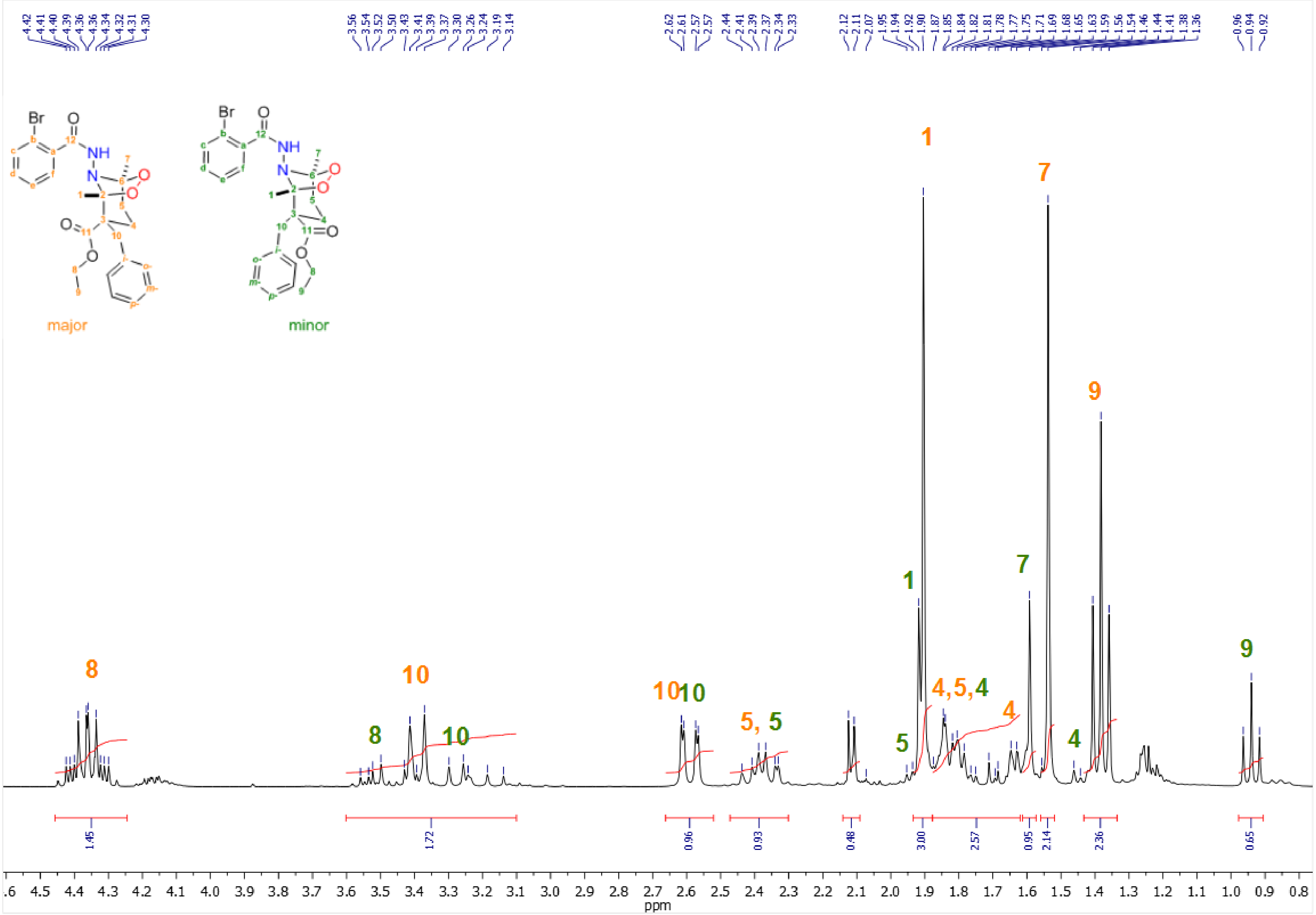

^{1}H NMR (300.13 MHz, $CDCl_3$). Ethyl 2-benzyl-8-(2-bromobenzamido)-1,5-dimethyl-6,7-dioxa-8-azabicyclo[3.2.1]octane-2-carboxylate, 20a + 20b

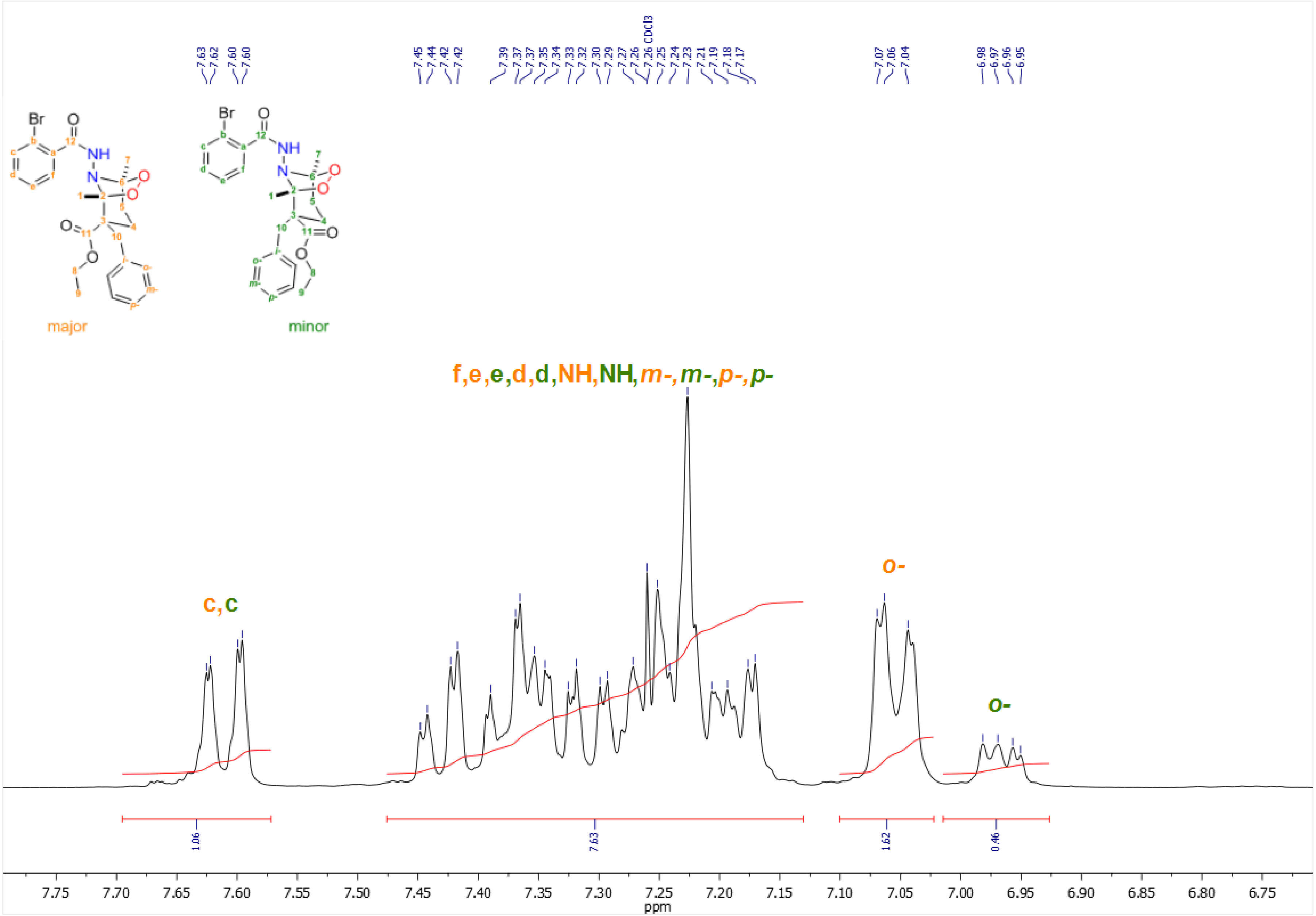

^{13}C NMR (75.48 MHz, CDCl3). Ethyl 2-benzyl-8-(2-bromobenzamido)-1,5-dimethyl-6,7-dioxa-8-azabicyclo[3.2.1]octane-2-carboxylate, 20a + 20b

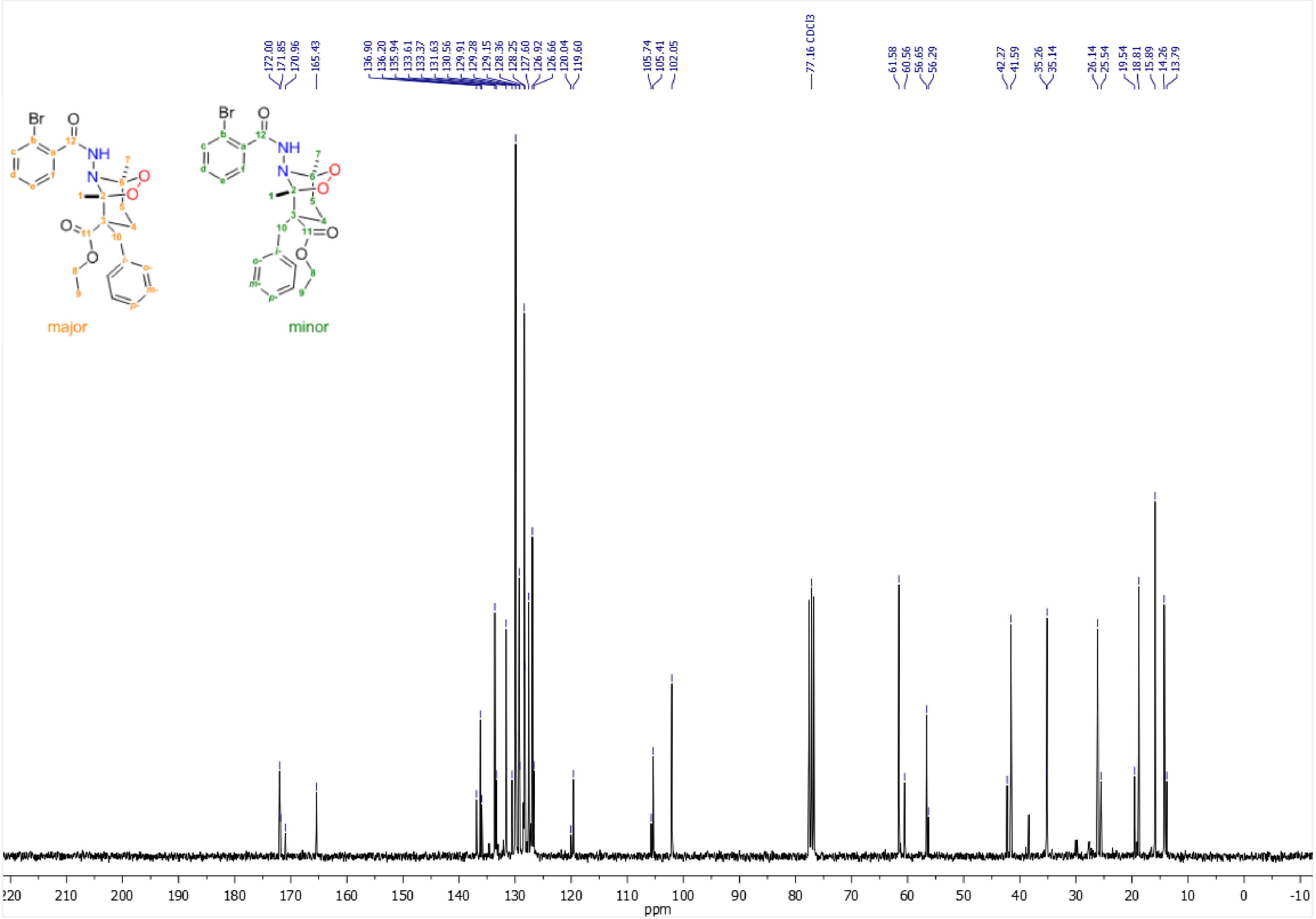

^{13}C NMR (75.48 MHz, $CDCl_3$). Ethyl 2-benzyl-8-(2-bromobenzamido)-1,5-dimethyl-6,7-dioxa-8-azabicyclo[3.2.1]octane-2-carboxylate, 20a + 20b

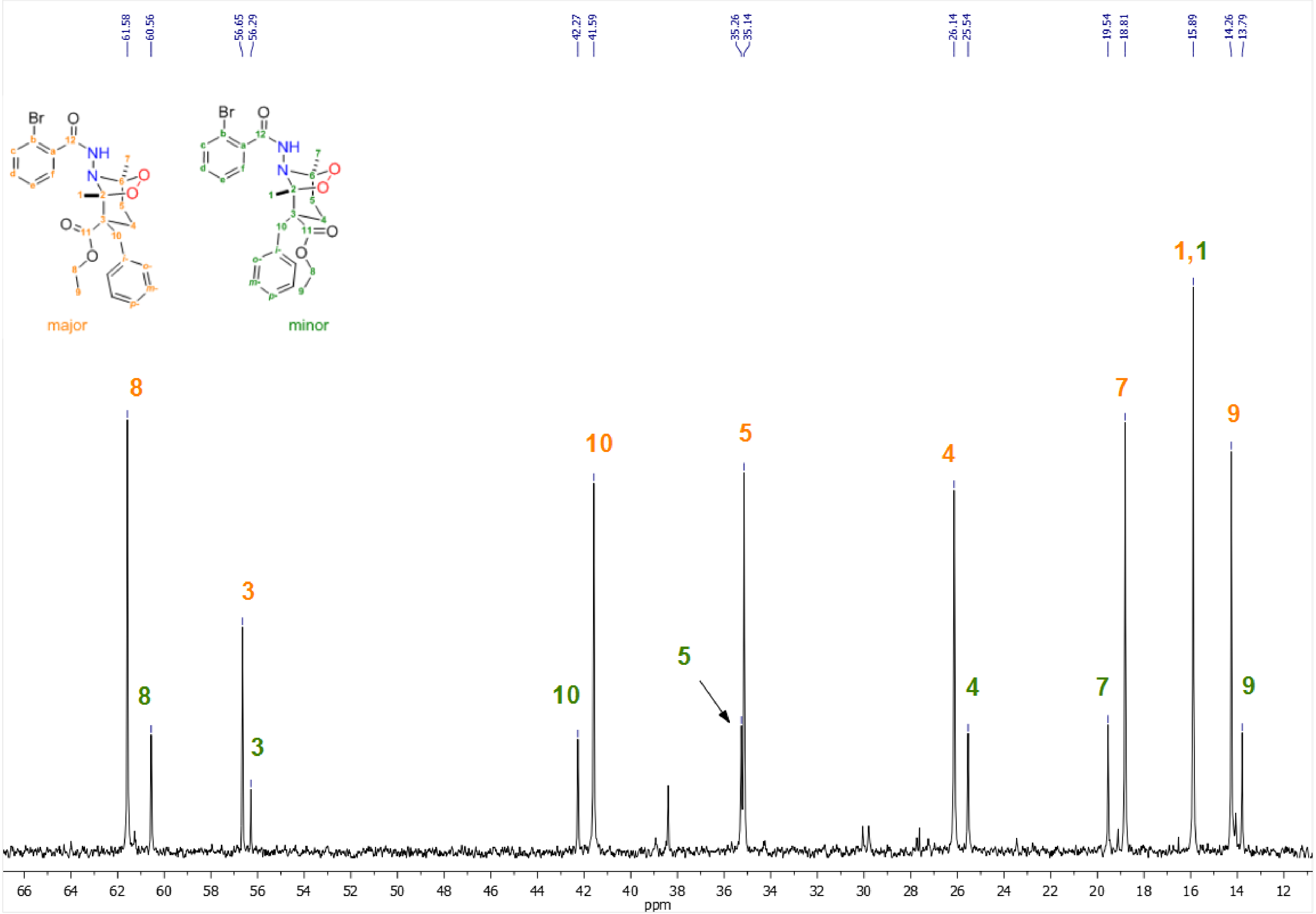

^{13}C NMR (75.48 MHz, CDCl3). Ethyl 2-benzyl-8-(2-bromobenzamido)-1,5-dimethyl-6,7-dioxa-8-azabicyclo[3.2.1]octane-2-carboxylate, 20a + 20b

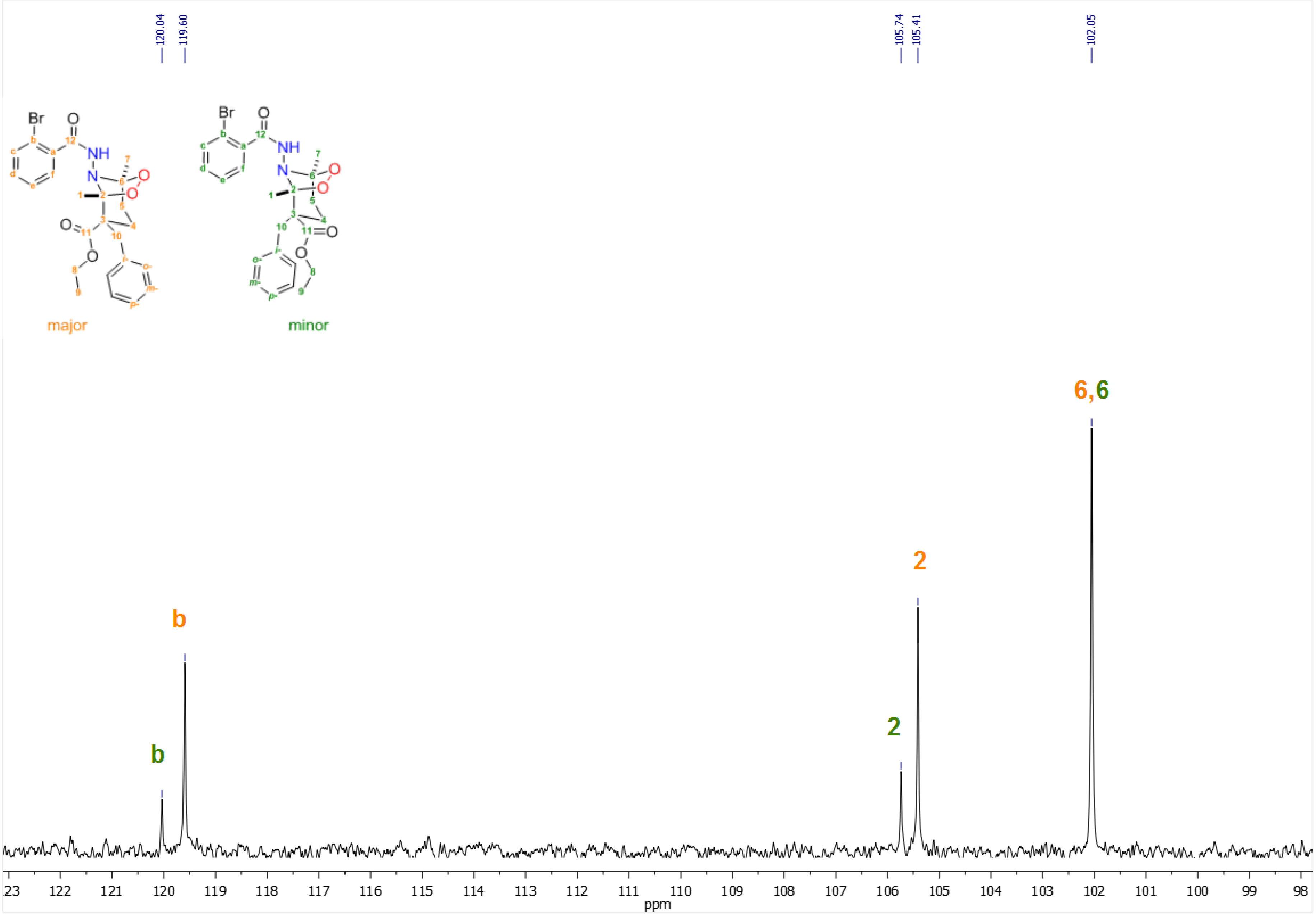

^{13}C NMR (75.48 MHz, $CDCl_3$). Ethyl 2-benzyl-8-(2-bromobenzamido)-1,5-dimethyl-6,7-dioxa-8-azabicyclo[3.2.1]octane-2-carboxylate, 20a + 20b

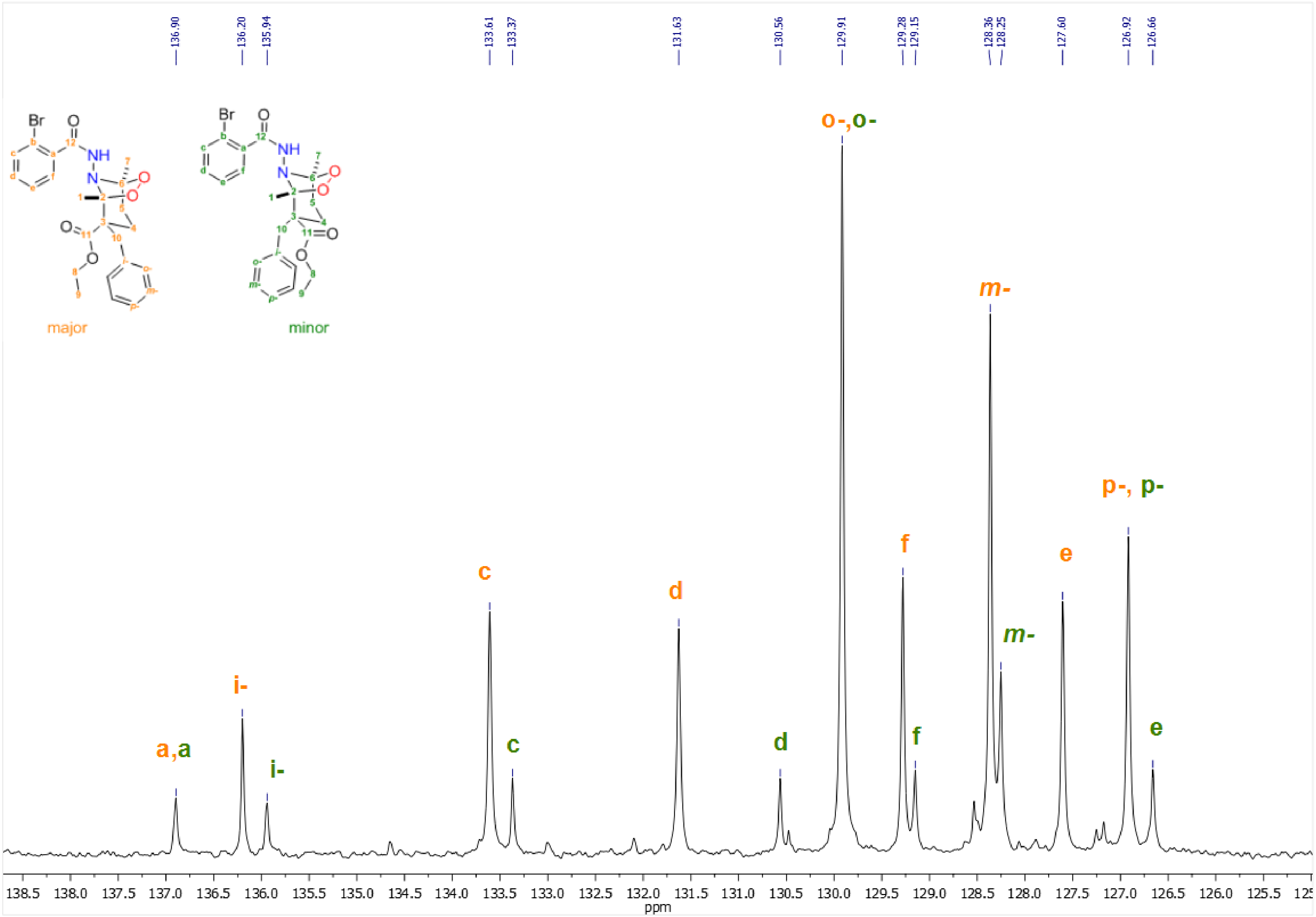

^{13}C NMR (75.48 MHz, CDCl$_3$). Ethyl 2-benzyl-8-(2-bromobenzamido)-1,5-dimethyl-6,7-dioxa-8-azabicyclo[3.2.1]octane-2-carboxylate, 20a + 20b

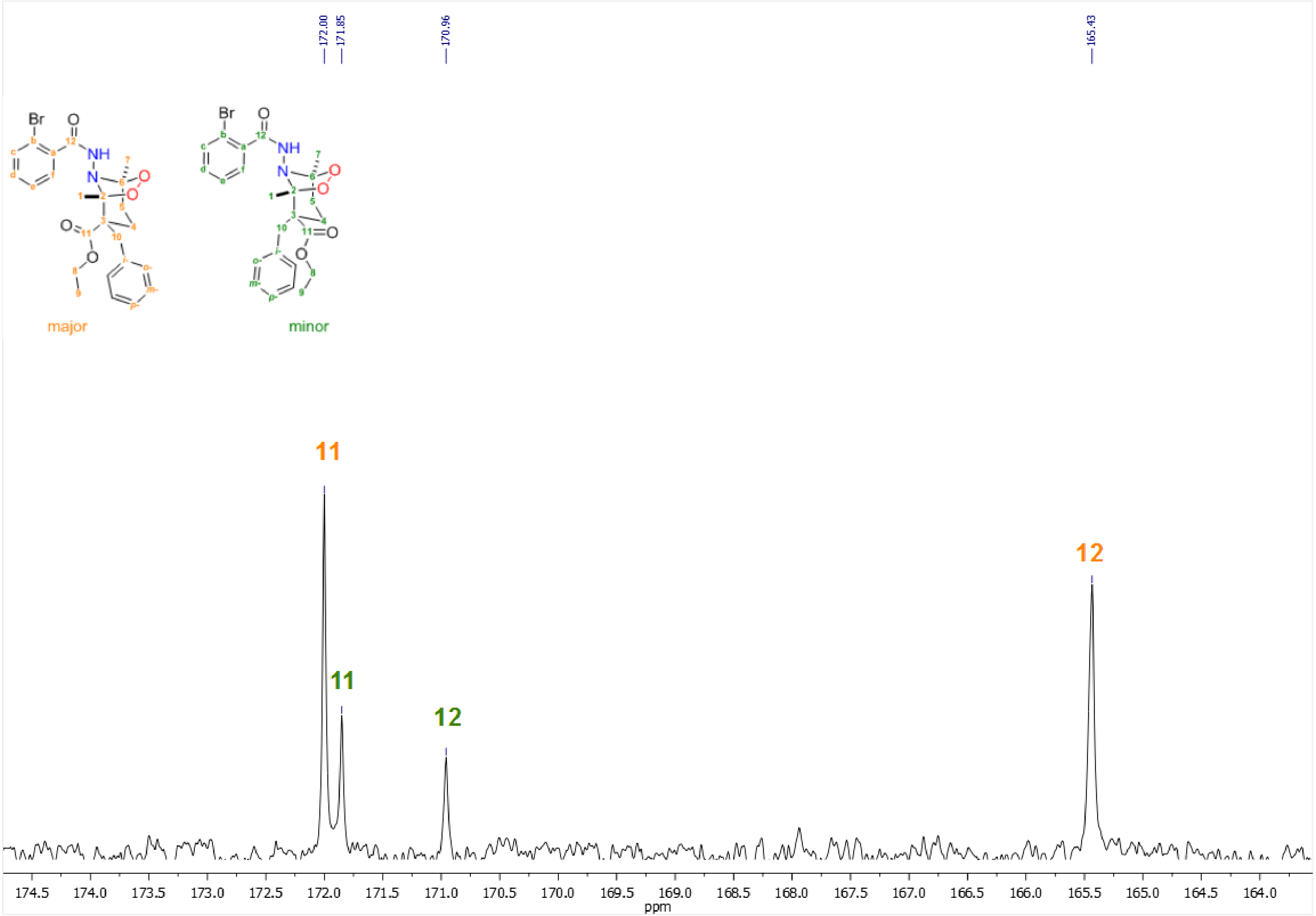

^{15}N (40.56 MHz, $CDCl_3$). Ethyl 2-benzyl-8-(2-bromobenzamido)-1,5-dimethyl-6,7-dioxa-8-azabicyclo[3.2.1]octane-2-carboxylate, 20a + 20b

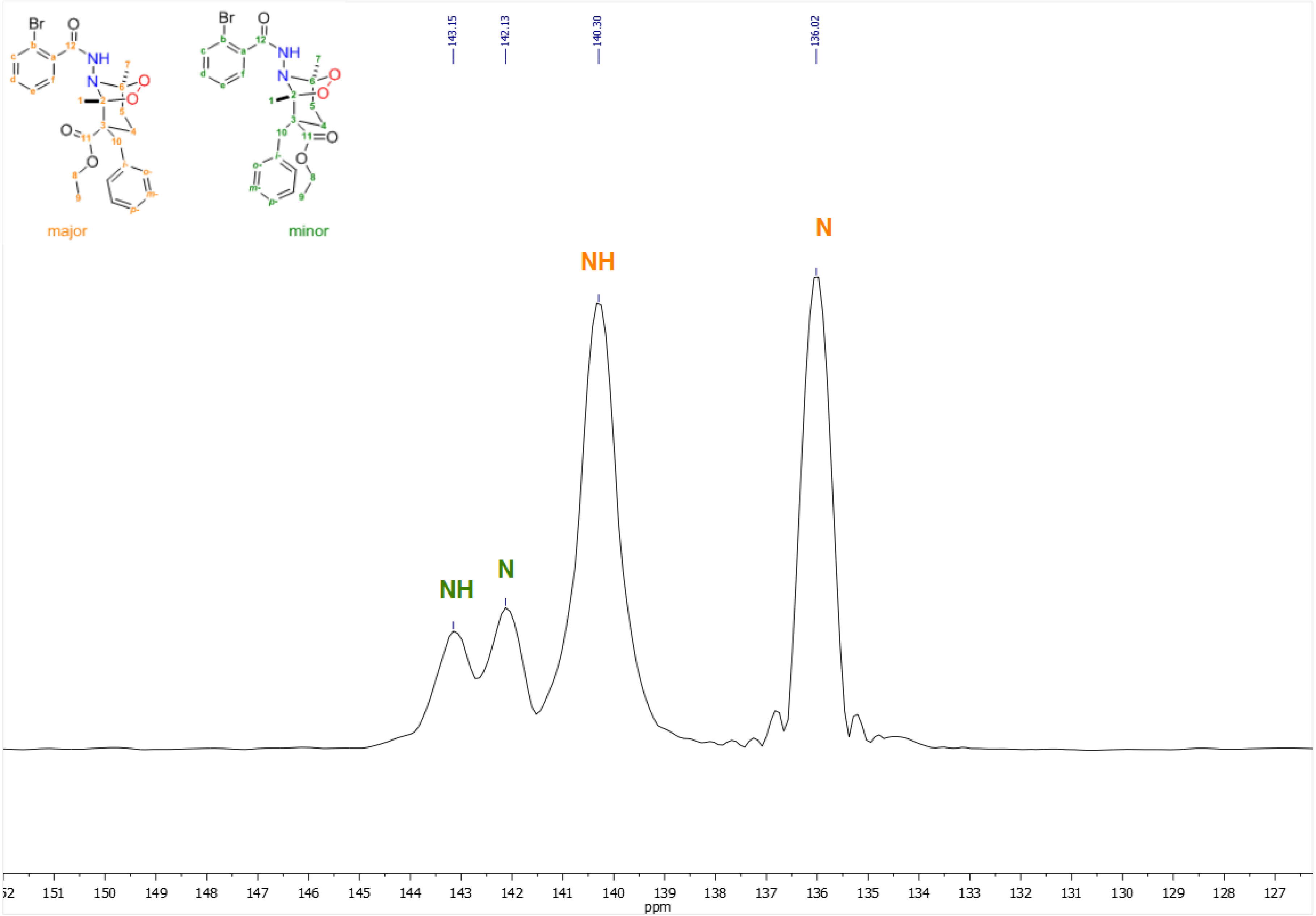

^{13}C NMR (75.48 MHz, CDCl$_3$). Ethyl 2-benzyl-8-(2-bromobenzamido)-1,5-dimethyl-6,7-dioxa-8-azabicyclo[3.2.1]octane-2-carboxylate, 20a + 20b

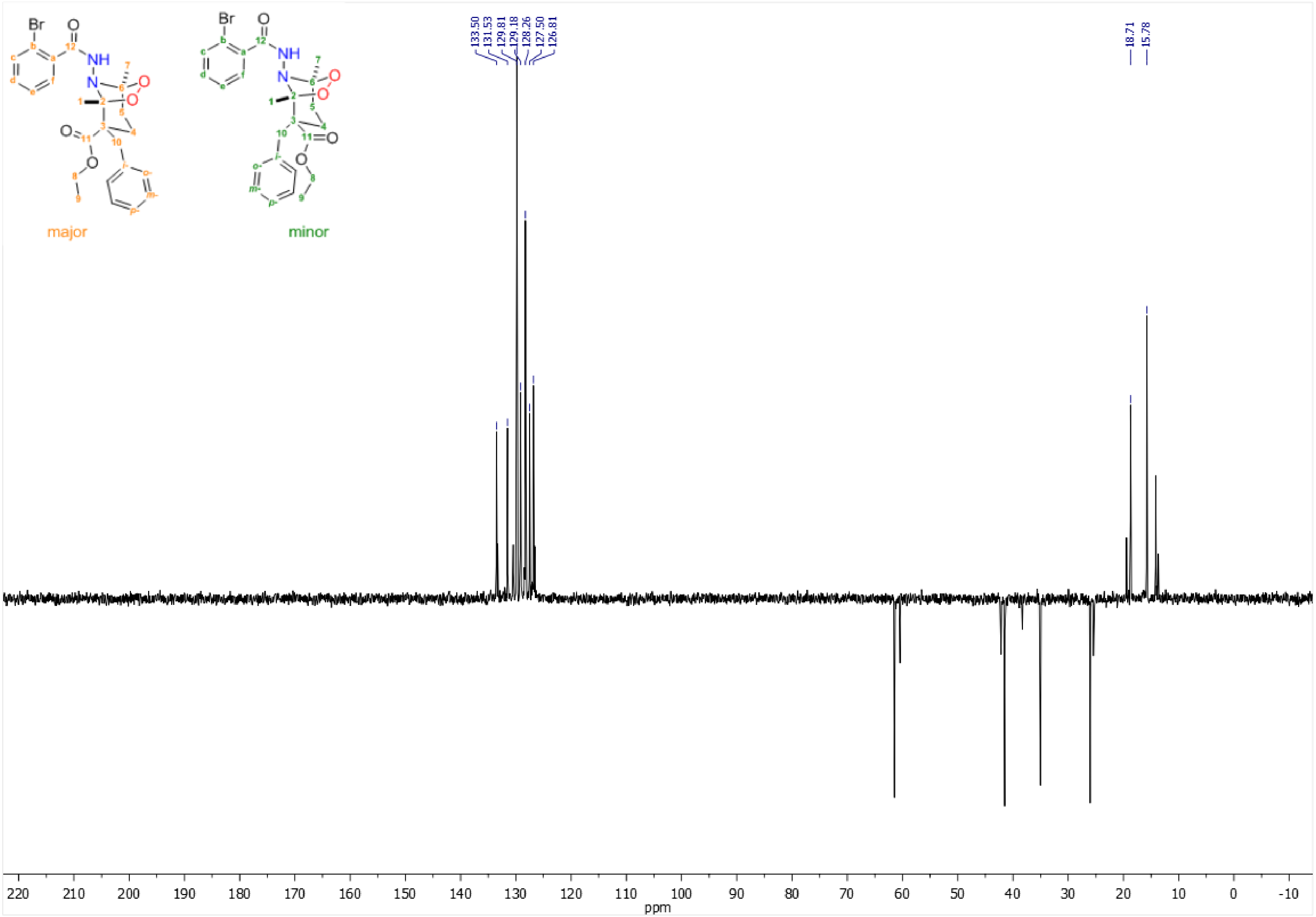

Ethyl 2-benzyl-8-(2-bromobenzamido)-1,5-dimethyl-6,7-dioxa-8-azabicyclo[3.2.1]octane-2-carboxylate, 20a + 20b

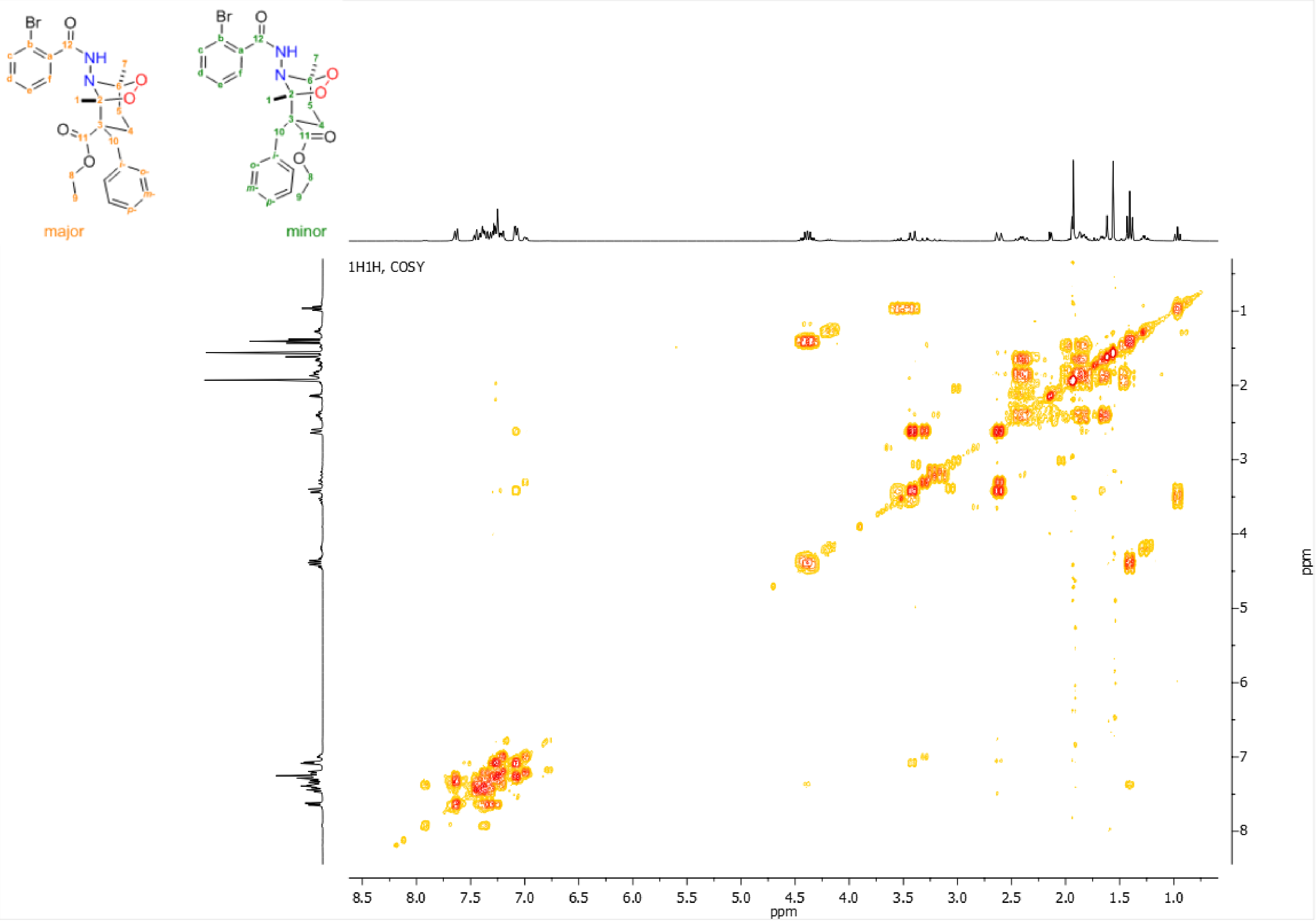

Ethyl 2-benzyl-8-(2-bromobenzamido)-1,5-dimethyl-6,7-dioxa-8-azabicyclo[3.2.1]octane-2-carboxylate, 20a + 20b

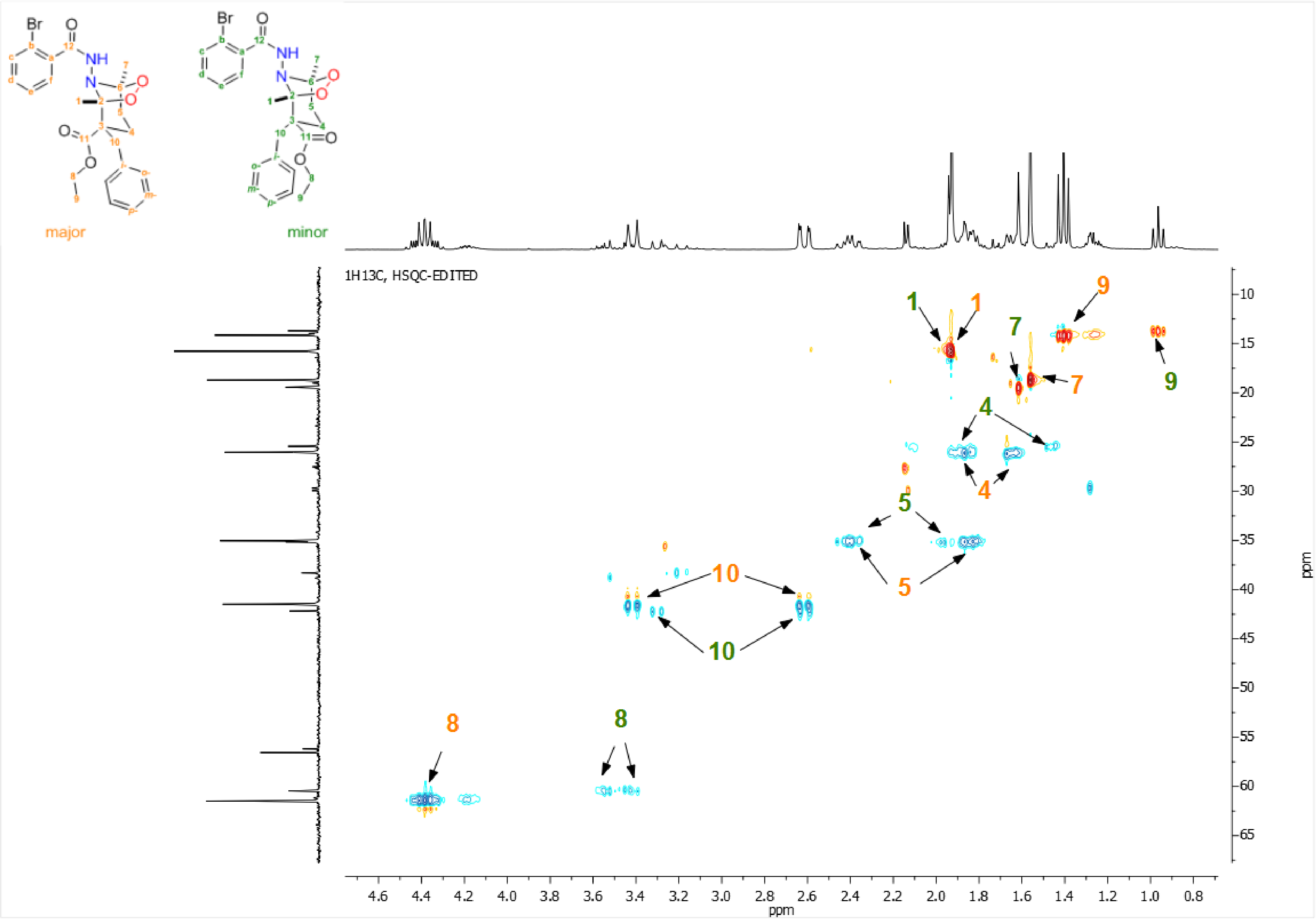

Ethyl 2-benzyl-8-(2-bromobenzamido)-1,5-dimethyl-6,7-dioxa-8-azabicyclo[3.2.1]octane-2-carboxylate, 20a + 20b

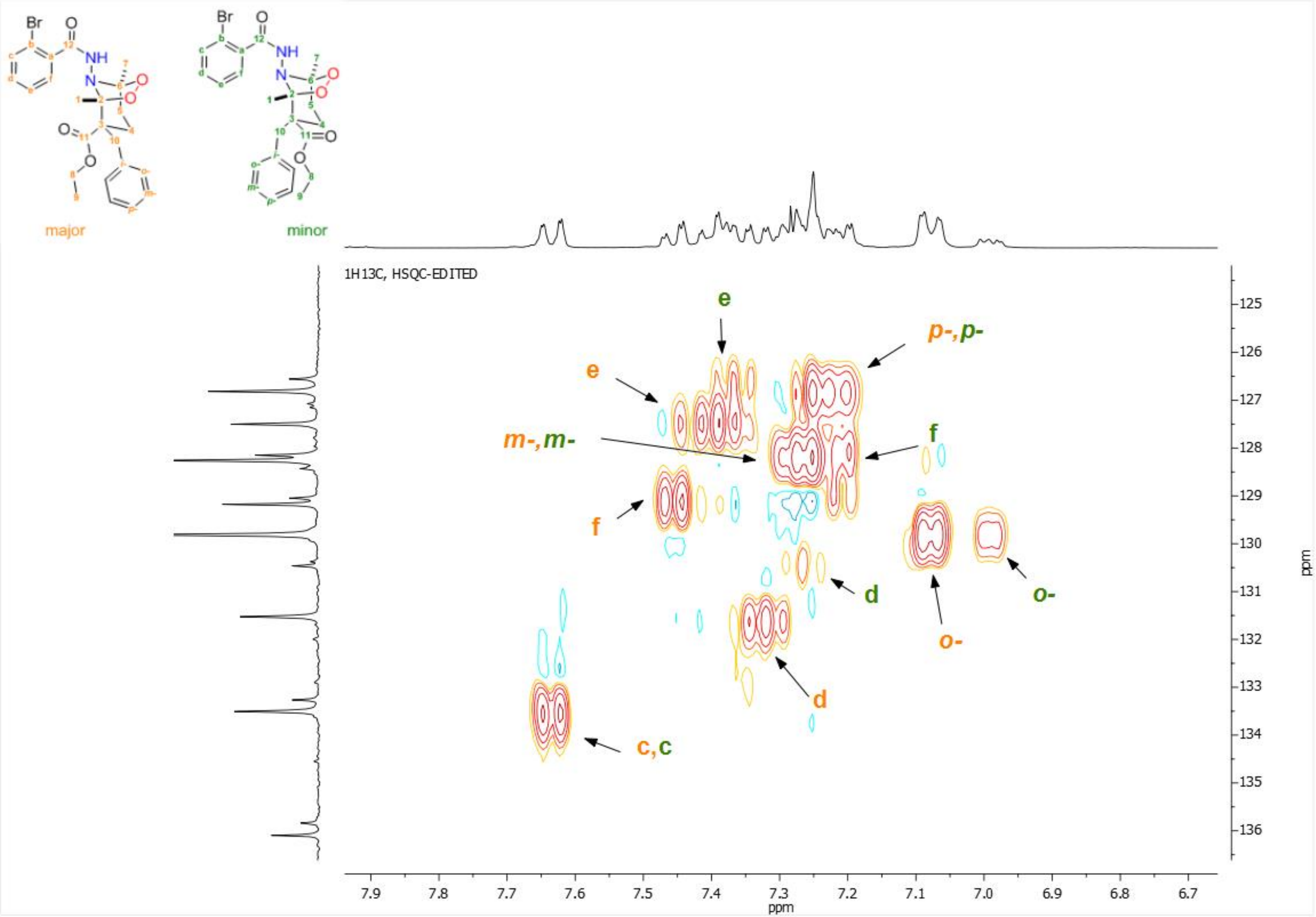

Ethyl 2-benzyl-8-(2-bromobenzamido)-1,5-dimethyl-6,7-dioxa-8-azabicyclo[3.2.1]octane-2-carboxylate, 20a + 20b

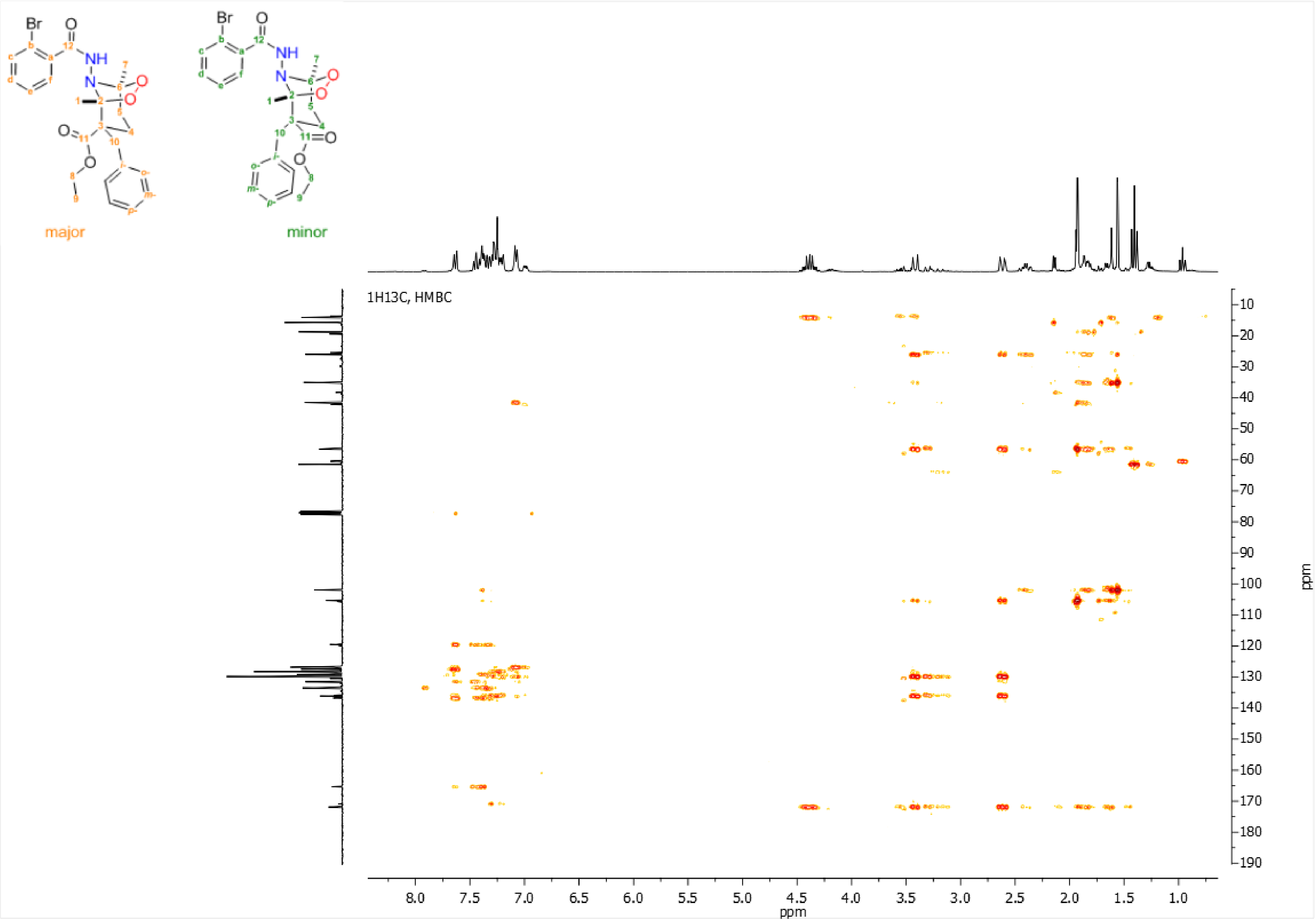

Ethyl 2-benzyl-8-(2-bromobenzamido)-1,5-dimethyl-6,7-dioxa-8-azabicyclo[3.2.1]octane-2-carboxylate, 20a + 20b

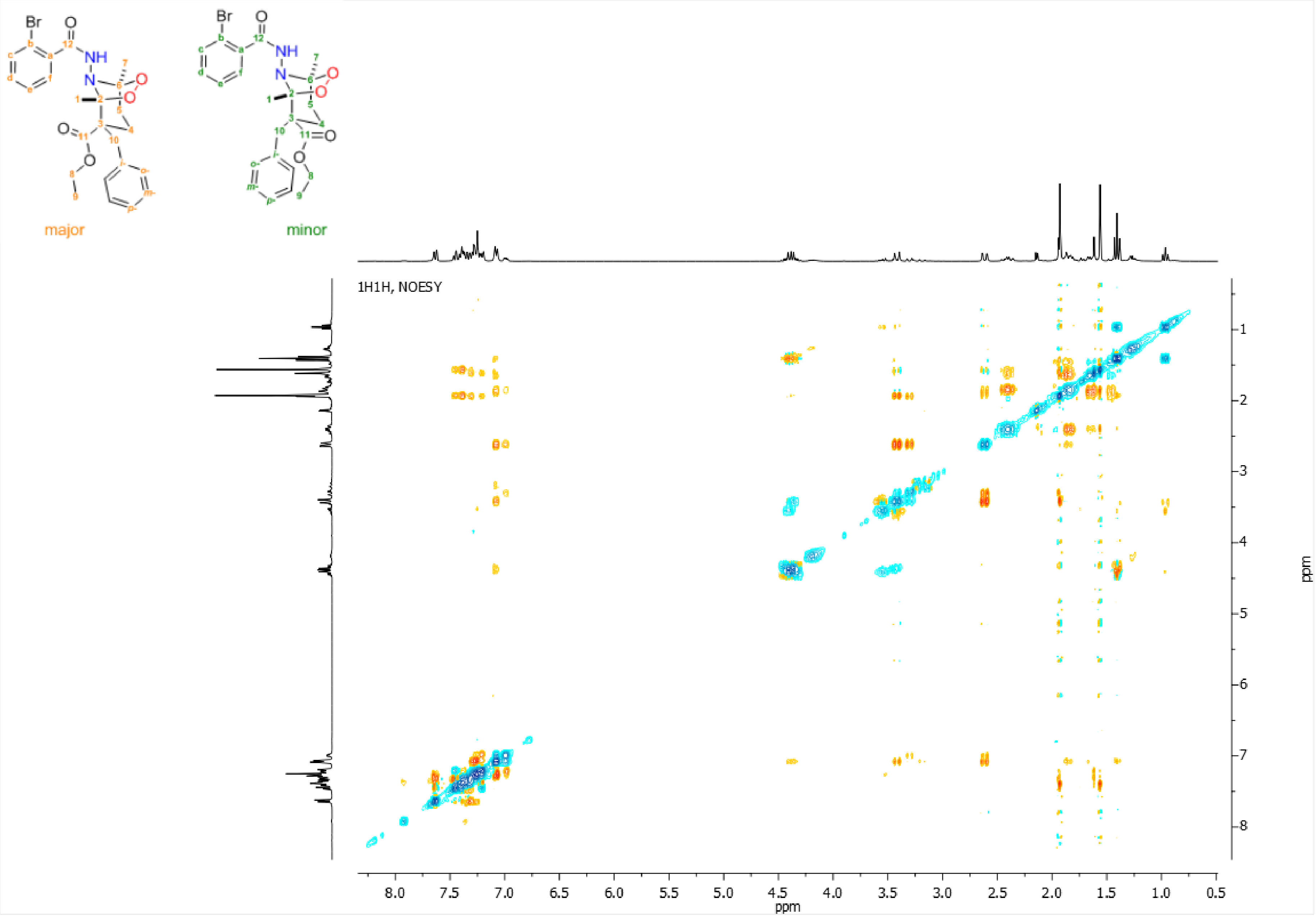

Ethyl 2-benzyl-8-(2-bromobenzamido)-1,5-dimethyl-6,7-dioxa-8-azabicyclo[3.2.1]octane-2-carboxylate, 20a + 20b

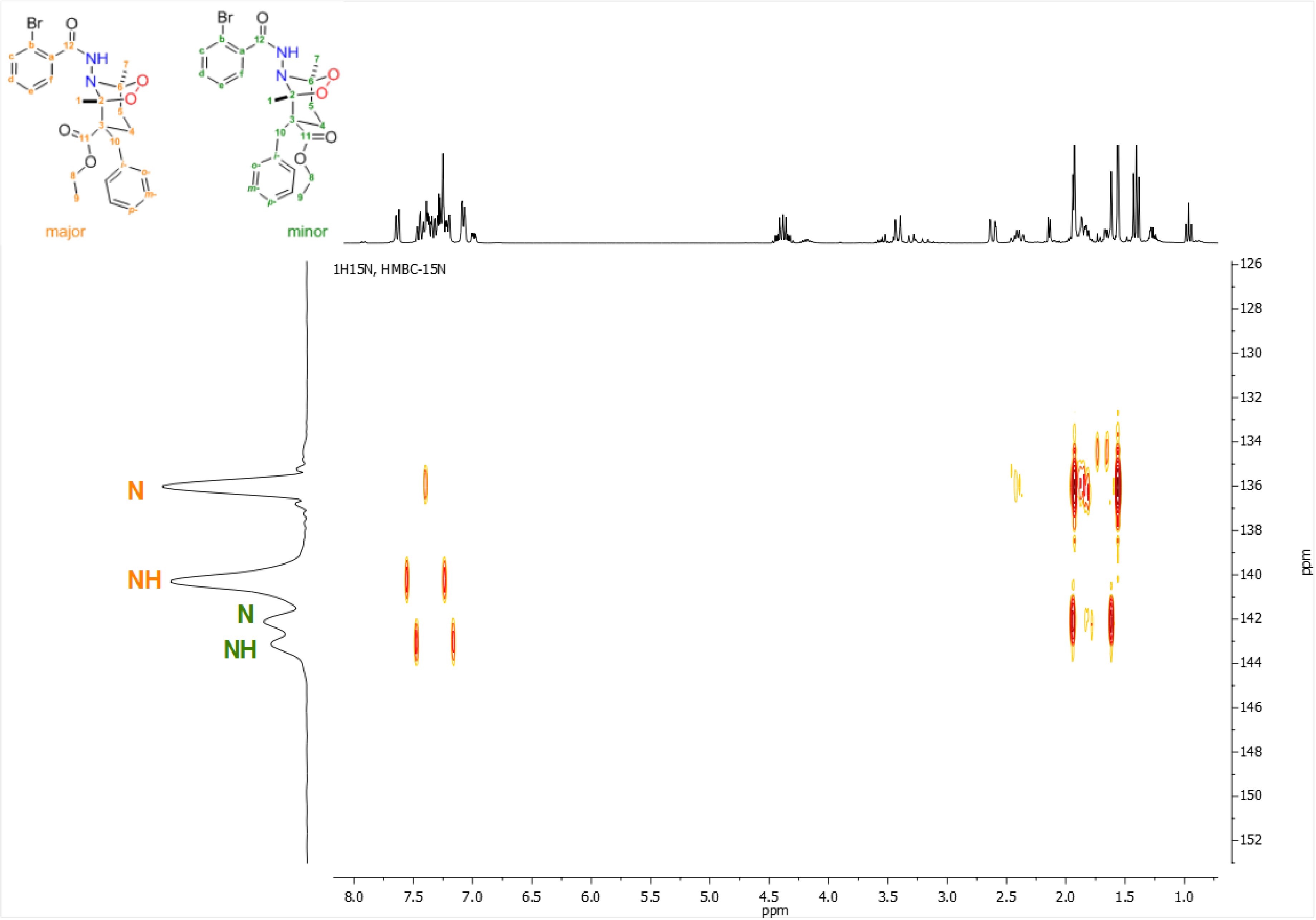

Ethyl 2-benzyl-8-(2-bromobenzamido)-1,5-dimethyl-6,7-dioxa-8-azabicyclo[3.2.1]octane-2-carboxylate, 20a + 20b

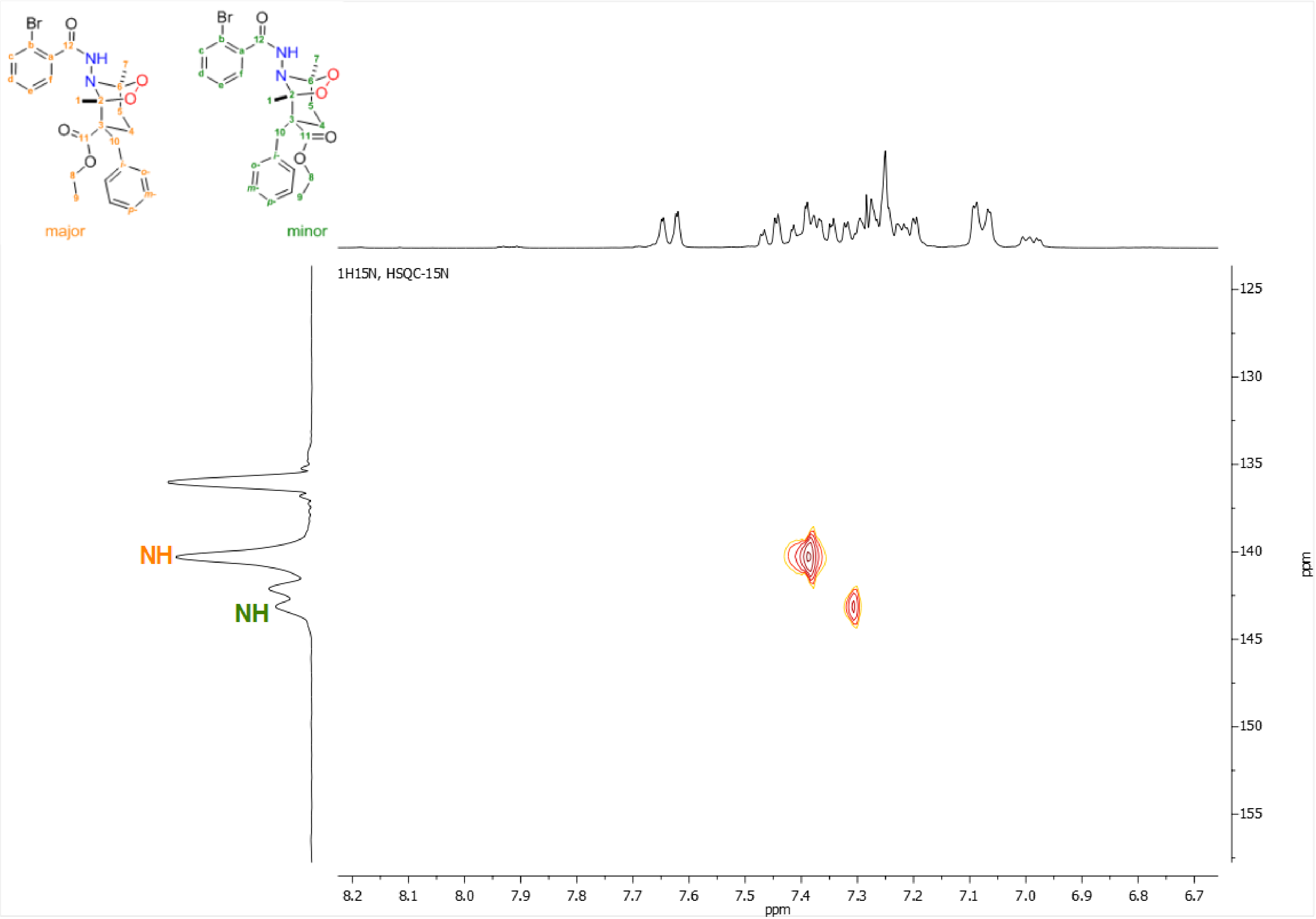

^{1}H NMR (300.13 MHz, $CDCl_3$). Benzyl 2-allyl-8-(2-bromobenzamido)-1,5-dimethyl-6,7-dioxa-8-azabicyclo[3.2.1]octane-2-carboxylate, 21a+21b

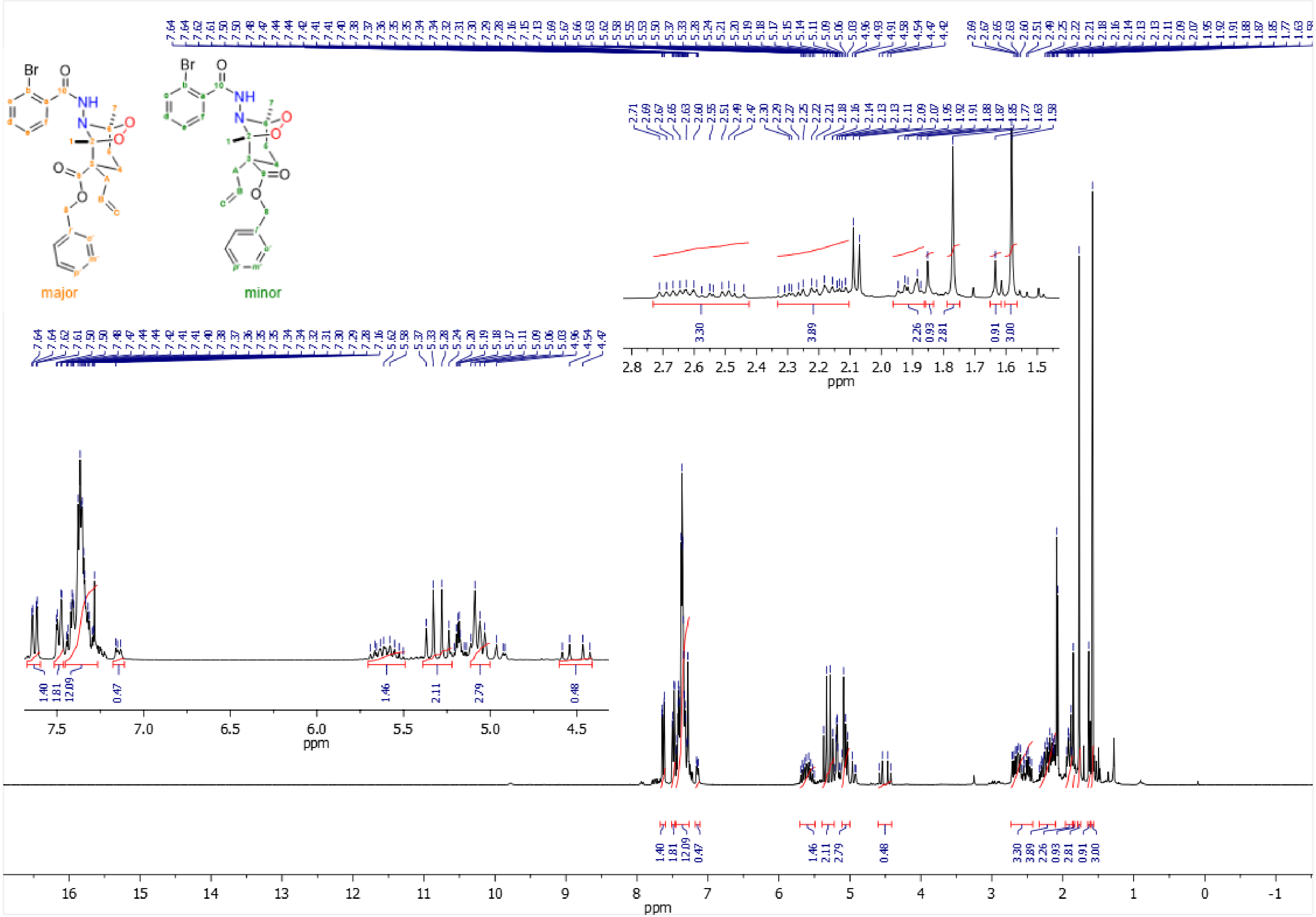

^{1}H NMR (300.13 MHz, CDCl3). Benzyl 2-allyl-8-(2-bromobenzamido)-1,5-dimethyl-6,7-dioxa-8-azabicyclo[3.2.1]octane-2-carboxylate, 21a+21b

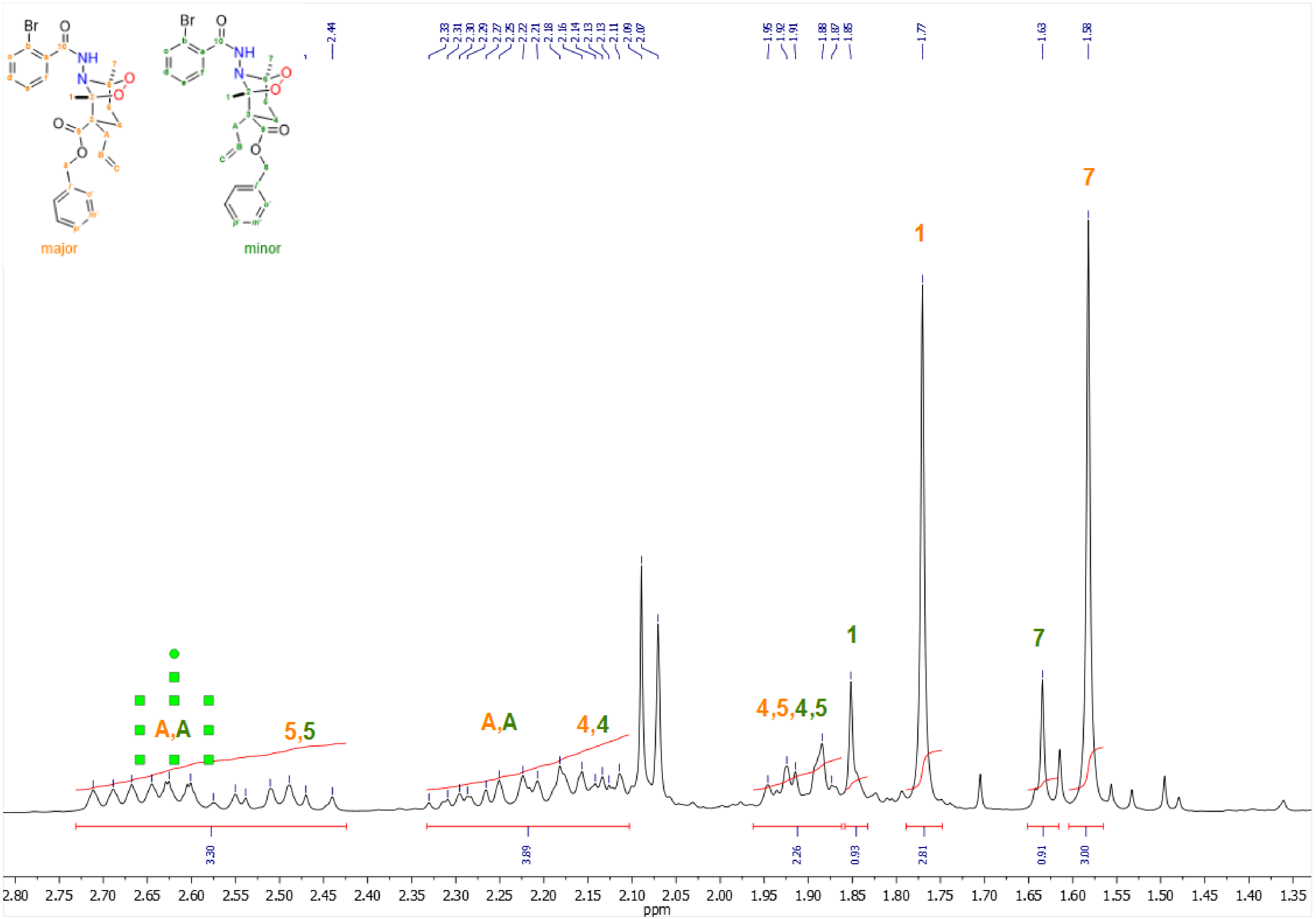

^{1}H NMR (300.13 MHz, CDCl3). Benzyl 2-allyl-8-(2-bromobenzamido)-1,5-dimethyl-6,7-dioxa-8-azabicyclo[3.2.1]octane-2-carboxylate, 21a+21b

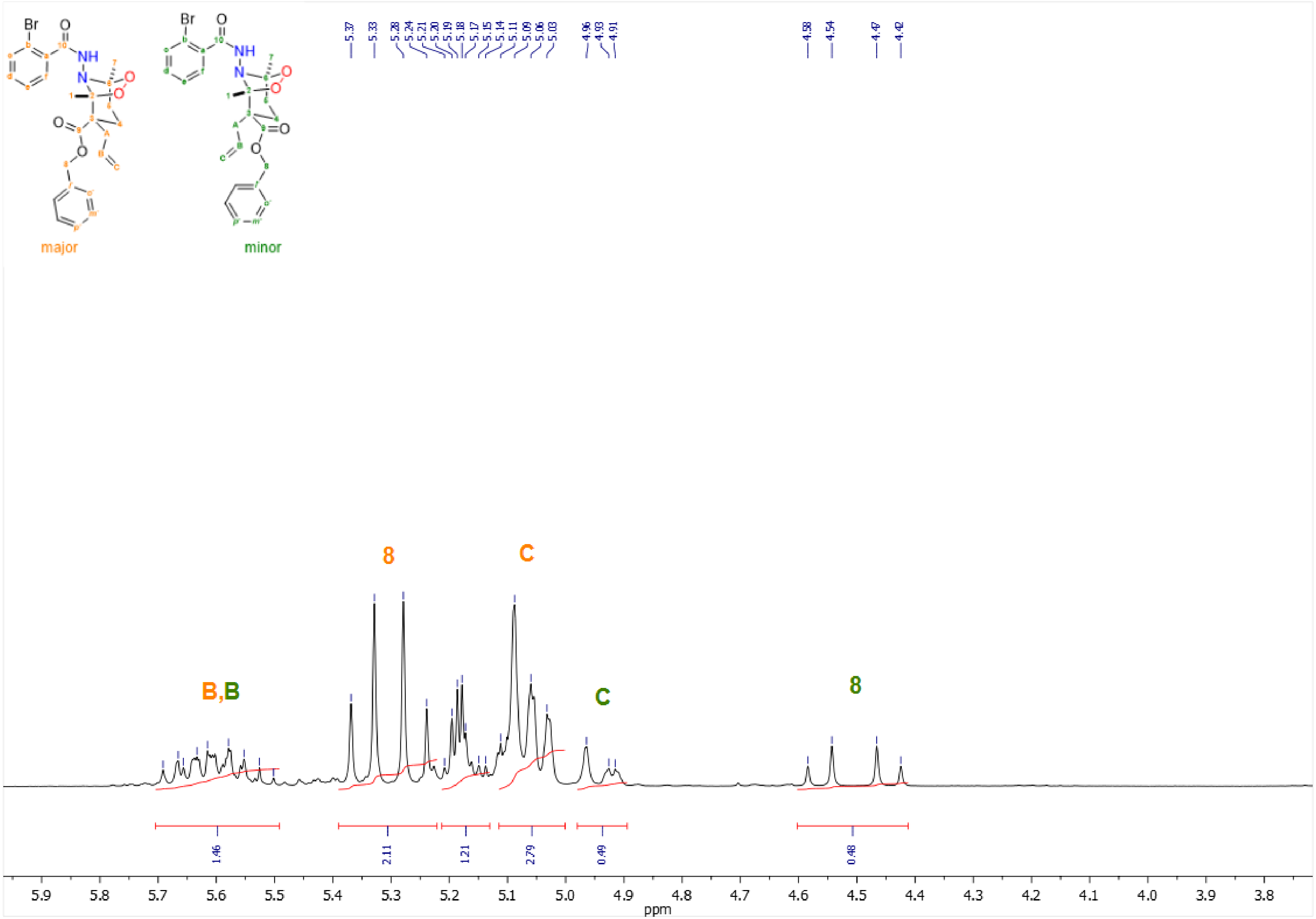

^{1}H NMR (300.13 MHz, $CDCl_3$). Benzyl 2-allyl-8-(2-bromobenzamido)-1,5-dimethyl-6,7-dioxa-8-azabicyclo[3.2.1]octane-2-carboxylate, 21a+21b

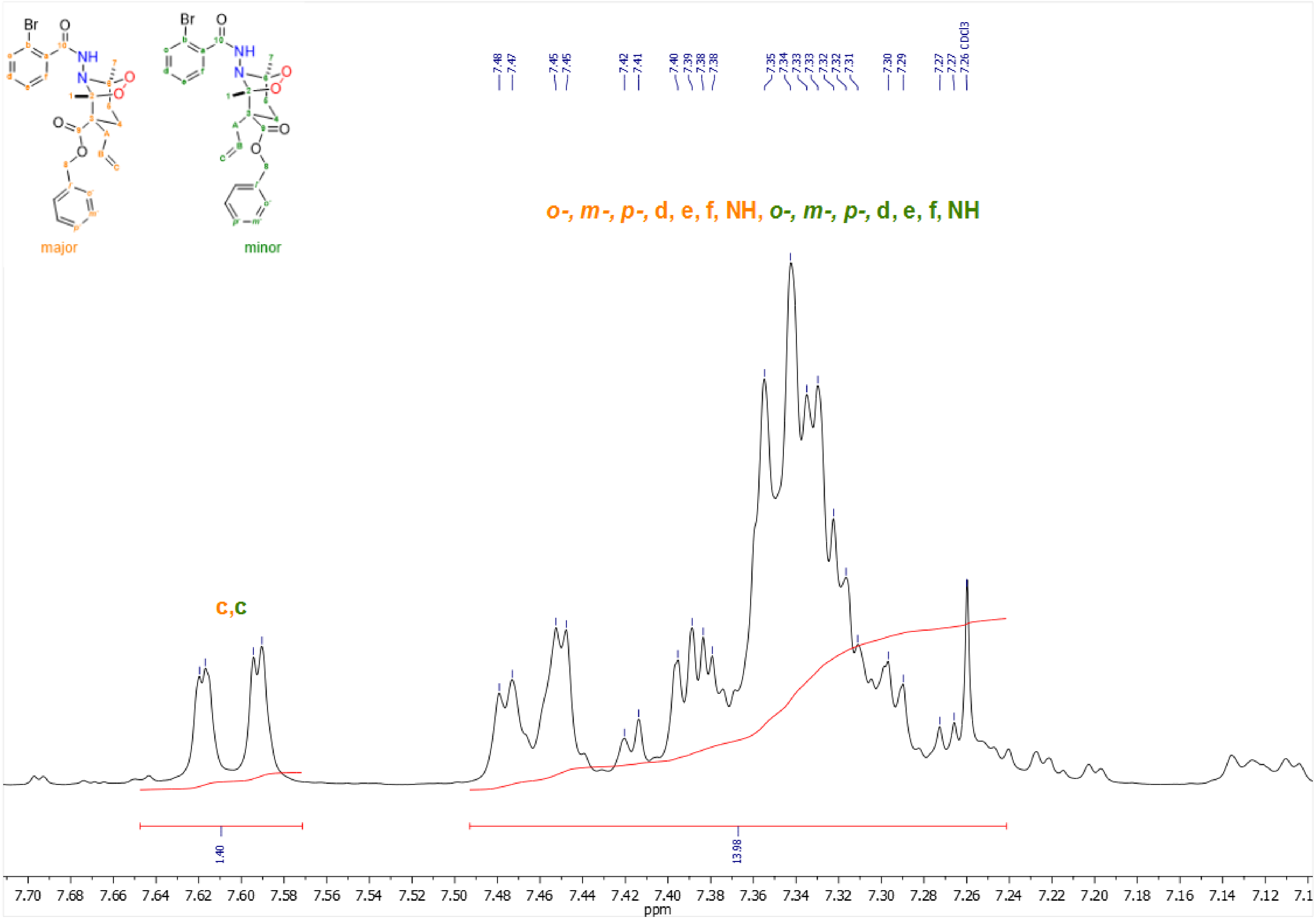

^{13}C NMR (75.48 MHz, CDCl$_3$). Benzyl 2-allyl-8-(2-bromobenzamido)-1,5-dimethyl-6,7-dioxa-8-azabicyclo[3.2.1]octane-2-carboxylate, 21a+21b

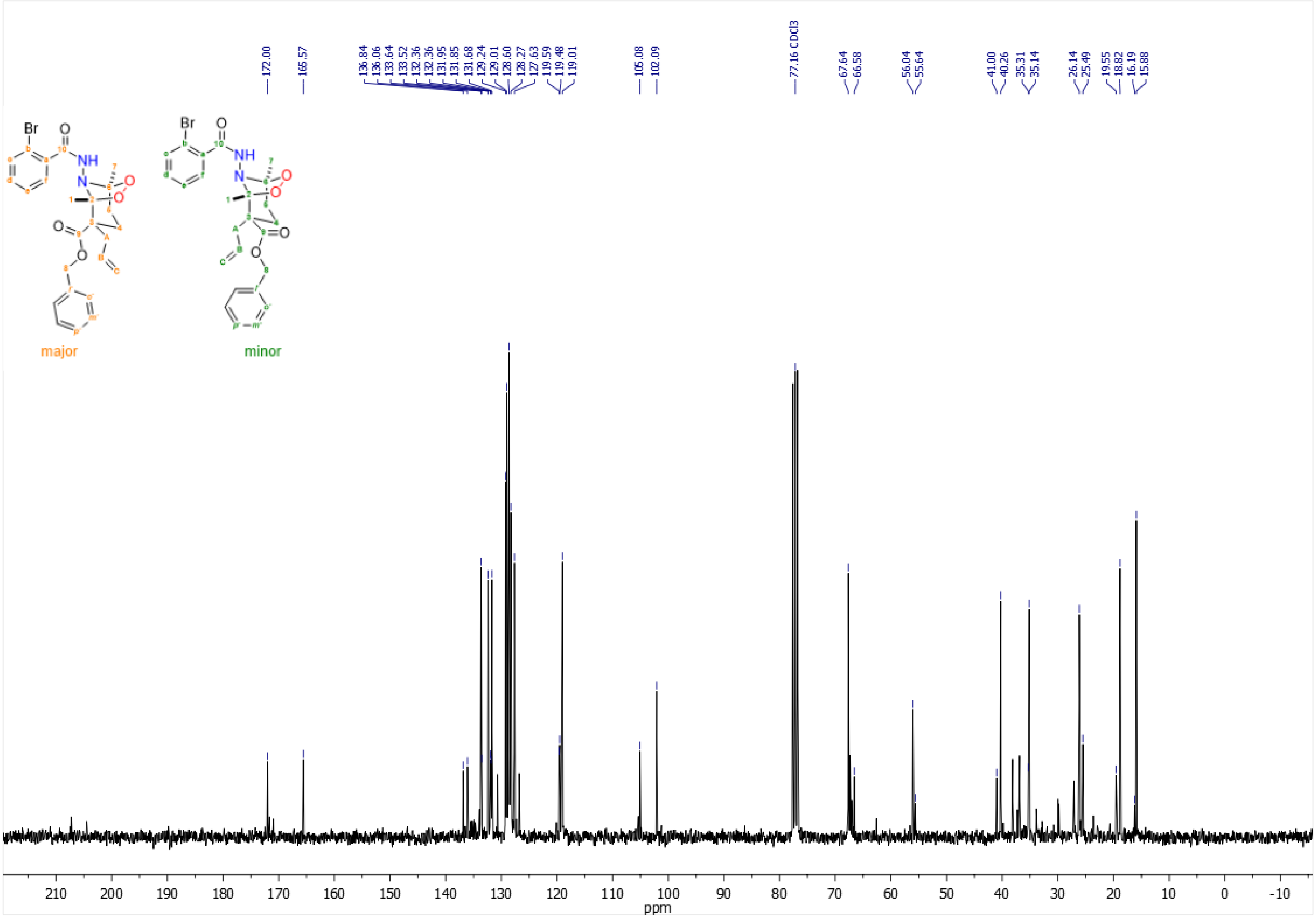

^{13}C NMR (75.48 MHz, $CDCl_3$). Benzyl 2-allyl-8-(2-bromobenzamido)-1,5-dimethyl-6,7-dioxa-8-azabicyclo[3.2.1]octane-2-carboxylate, 21a+21b

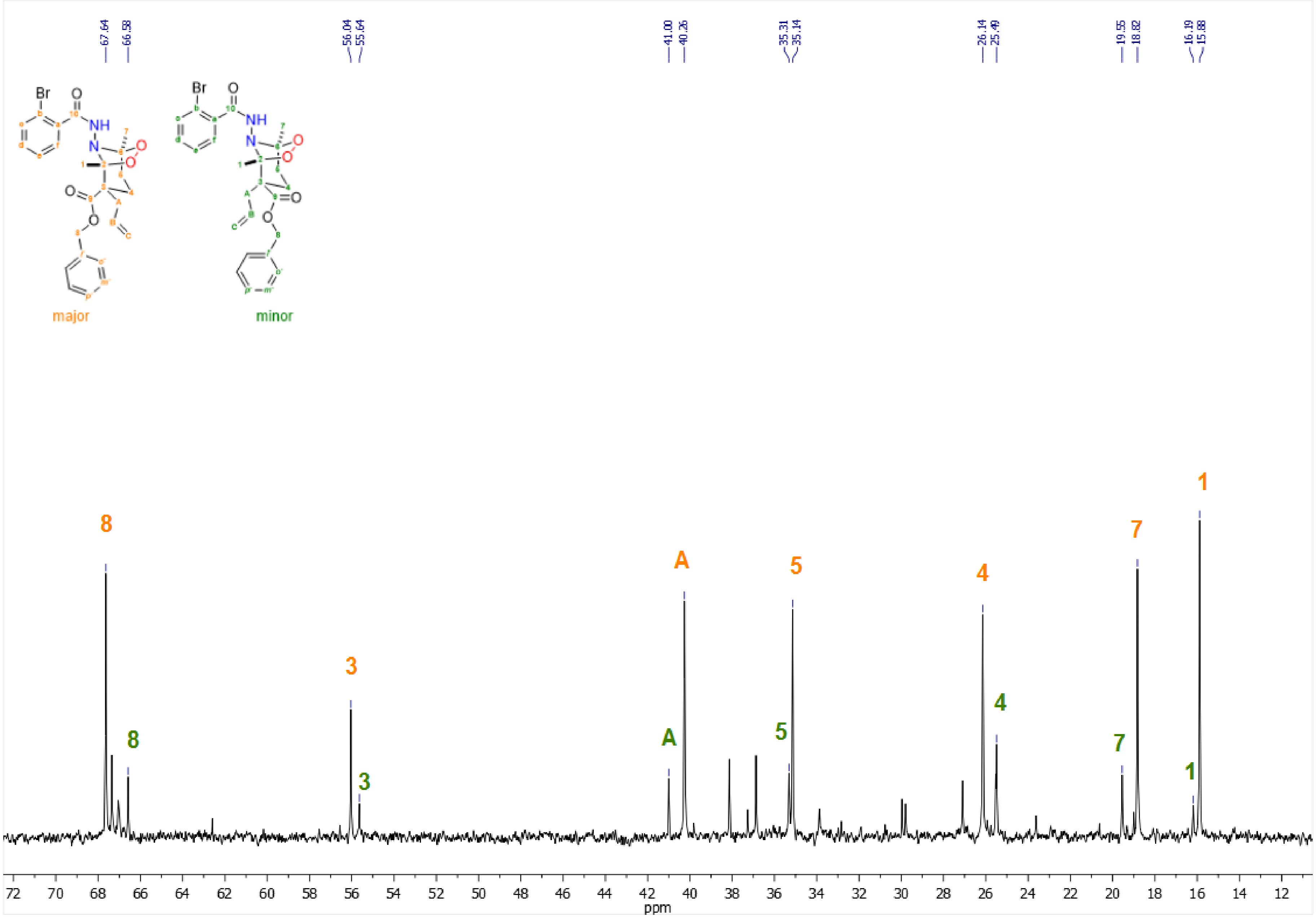

^{13}C NMR (75.48 MHz, $CDCl_3$). Benzyl 2-allyl-8-(2-bromobenzamido)-1,5-dimethyl-6,7-dioxa-8-azabicyclo[3.2.1]octane-2-carboxylate, 21a+21b

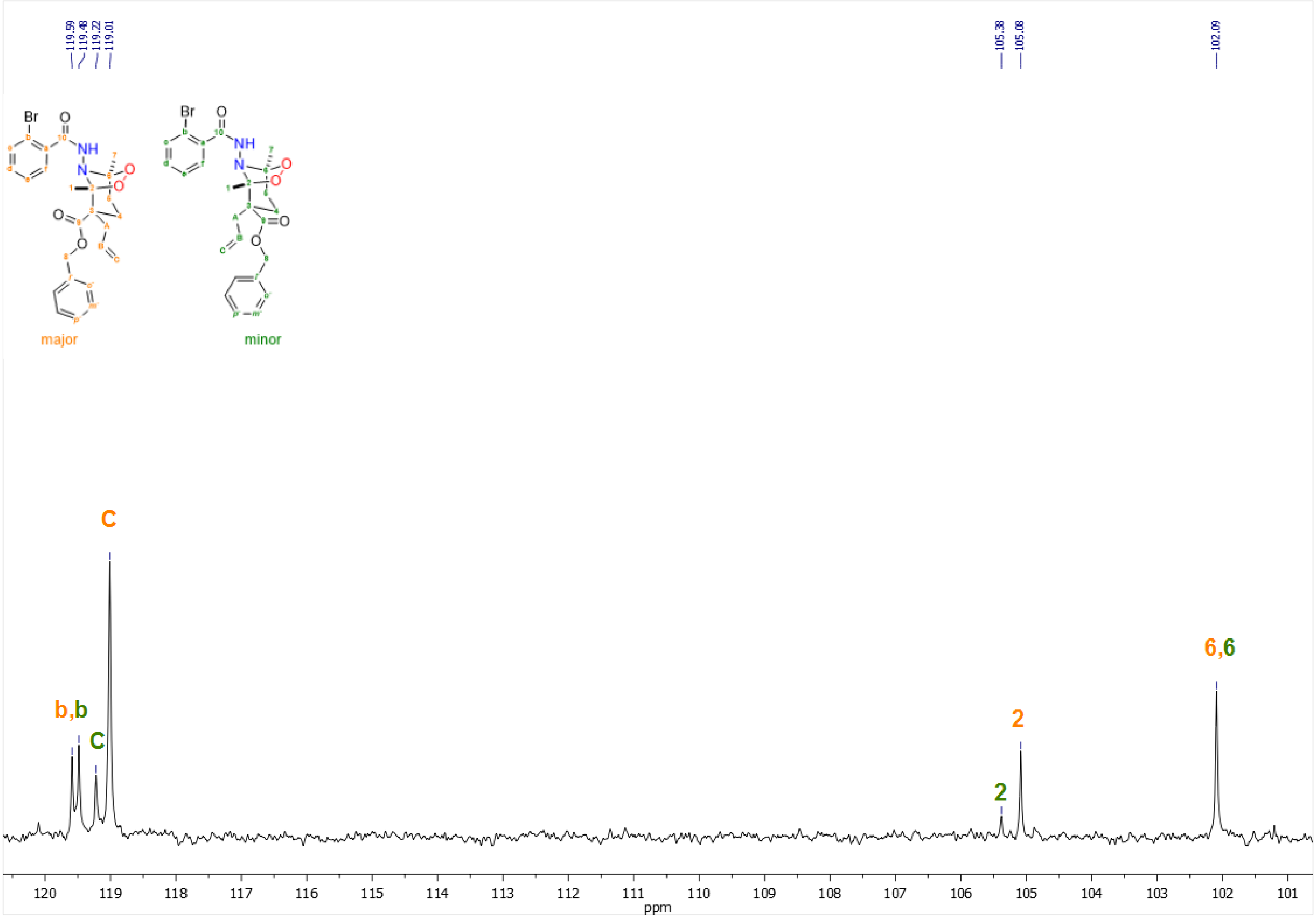

^{13}C NMR (75.48 MHz, CDCl3). Benzyl 2-allyl-8-(2-bromobenzamido)-1,5-dimethyl-6,7-dioxa-8-azabicyclo[3.2.1]octane-2-carboxylate, 21a+21b

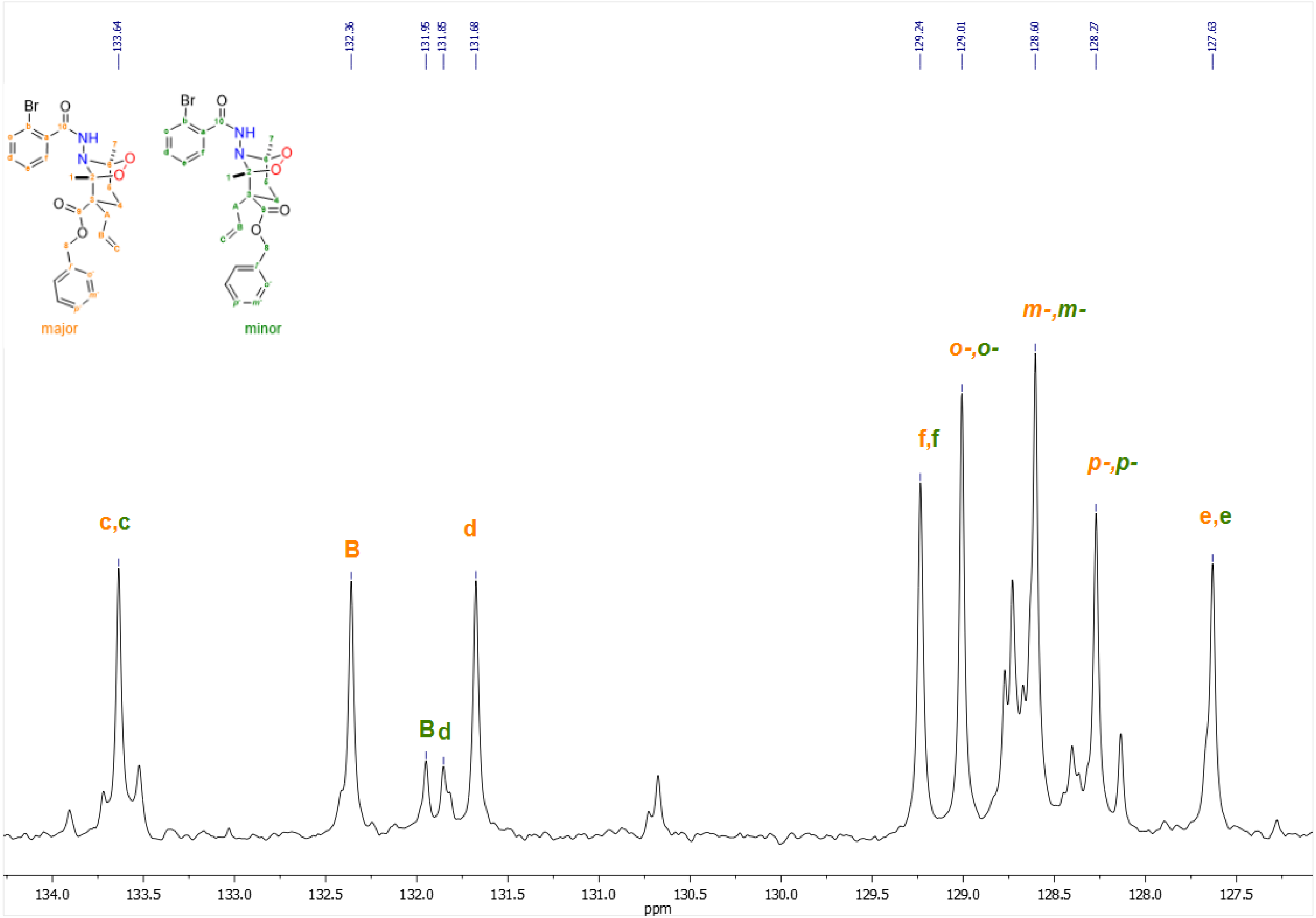

^{13}C NMR (75.48 MHz, CDCl3). Benzyl 2-allyl-8-(2-bromobenzamido)-1,5-dimethyl-6,7-dioxa-8-azabicyclo[3.2.1]octane-2-carboxylate, 21a+21b

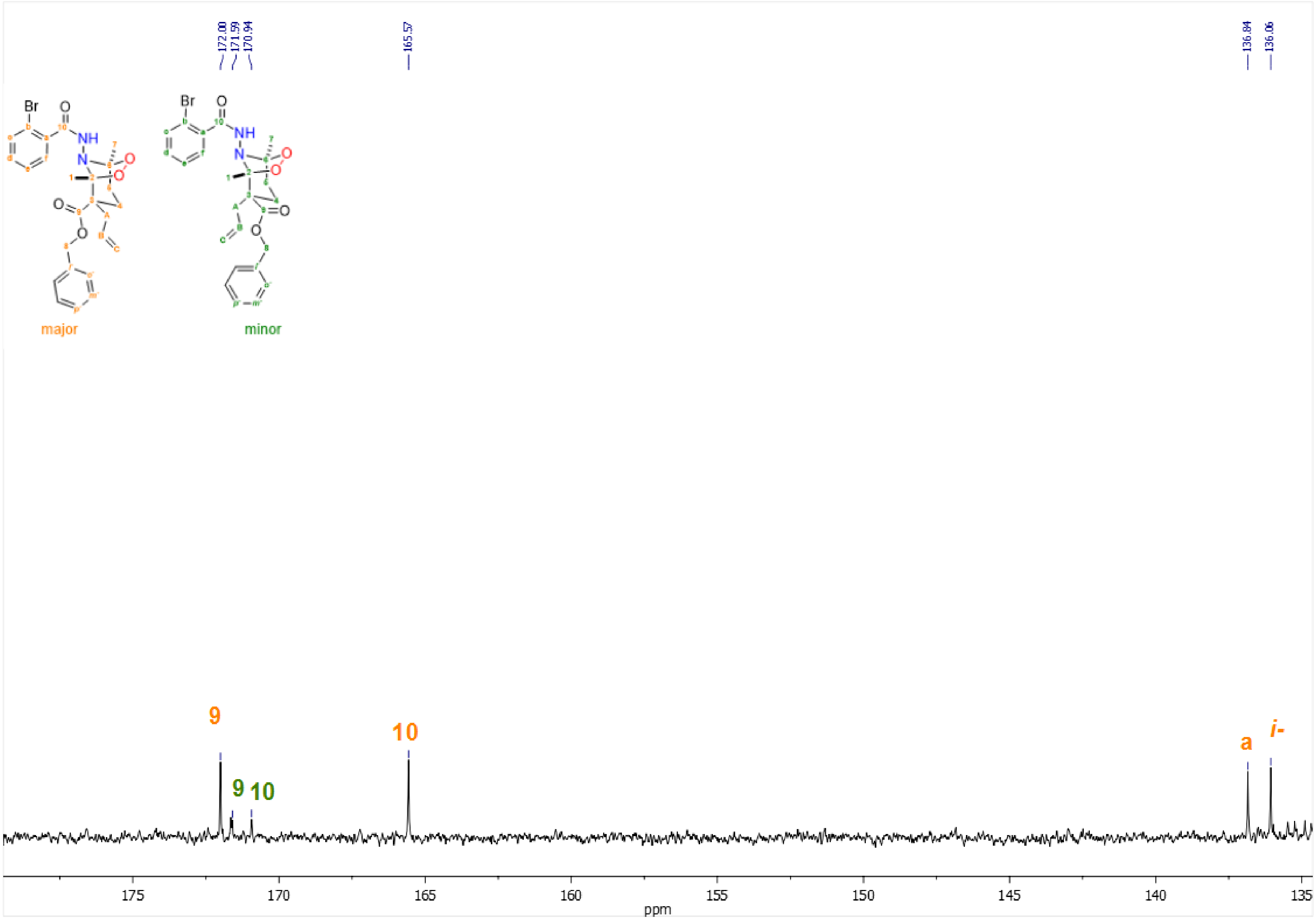

^{15}N (40.56 MHz, CDCl$_3$). Benzyl 2-allyl-8-(2-bromobenzamido)-1,5-dimethyl-6,7-dioxa-8-azabicyclo[3.2.1]octane-2-carboxylate, 21a+21b

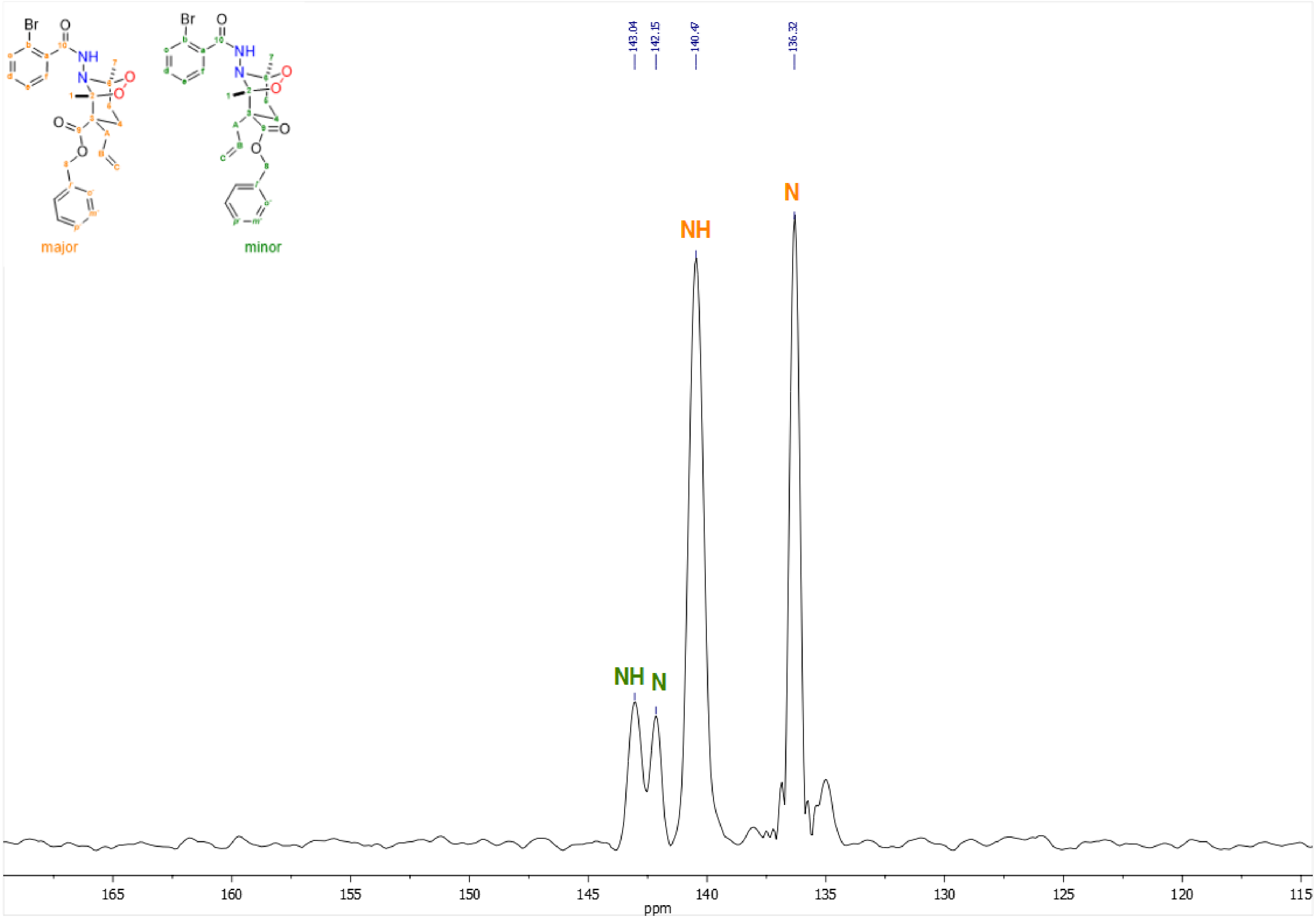

Benzyl 2-allyl-8-(2-bromobenzamido)-1,5-dimethyl-6,7-dioxa-8-azabicyclo[3.2.1]octane-2-carboxylate, 21a+21b

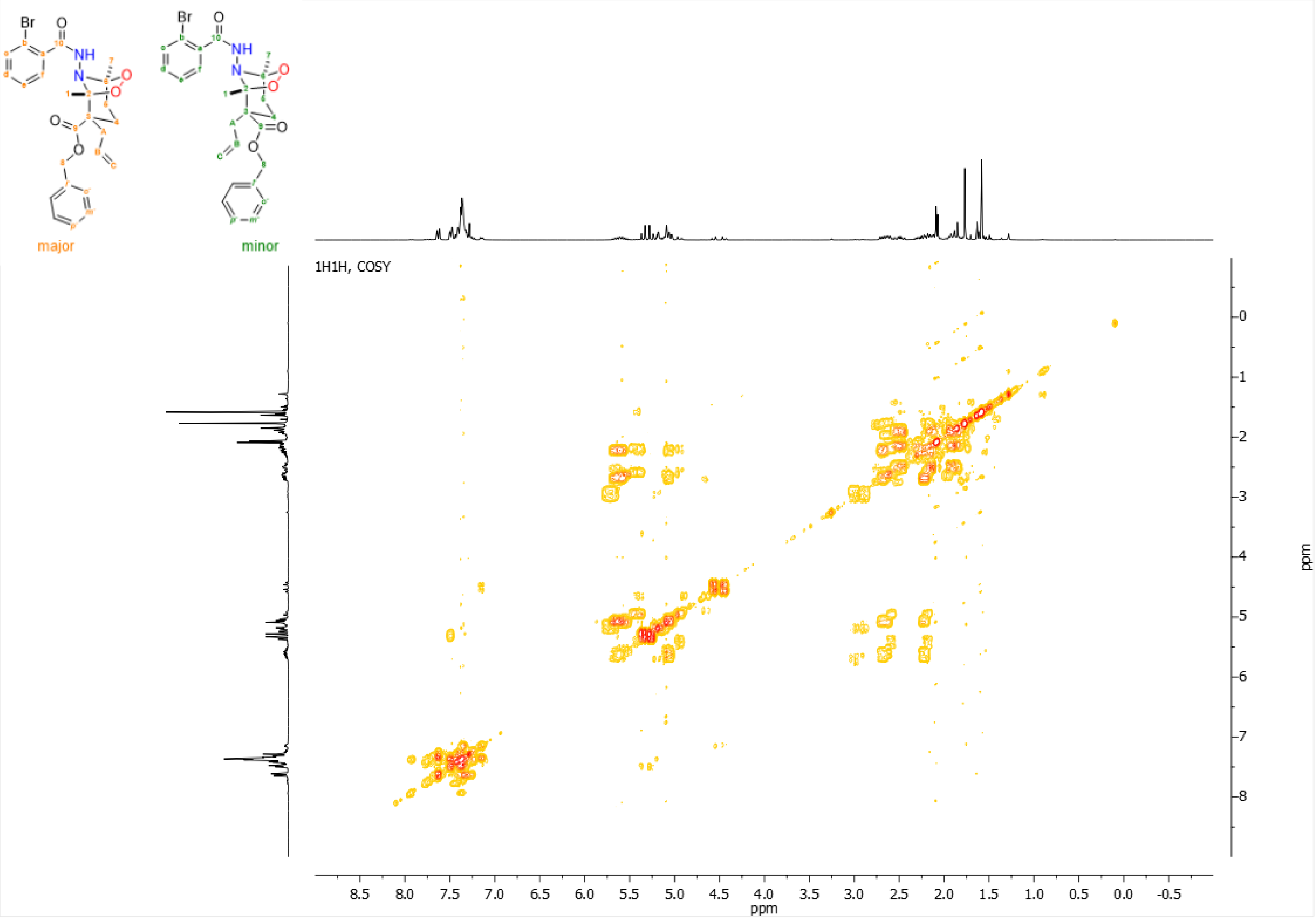

Benzyl 2-allyl-8-(2-bromobenzamido)-1,5-dimethyl-6,7-dioxa-8-azabicyclo[3.2.1]octane-2-carboxylate, 21a+21b

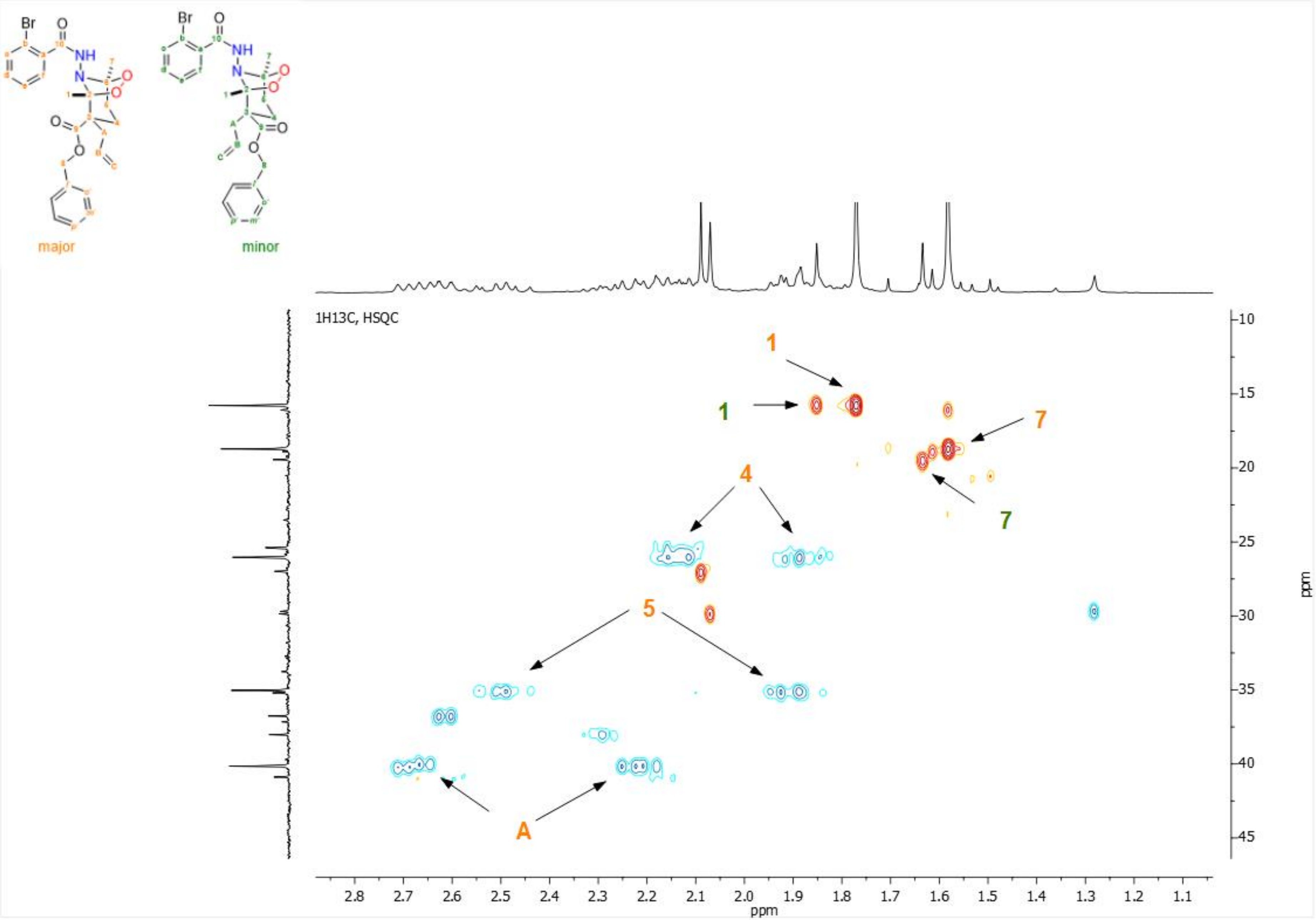

Benzyl 2-allyl-8-(2-bromobenzamido)-1,5-dimethyl-6,7-dioxa-8-azabicyclo[3.2.1]octane-2-carboxylate, 21a+21b

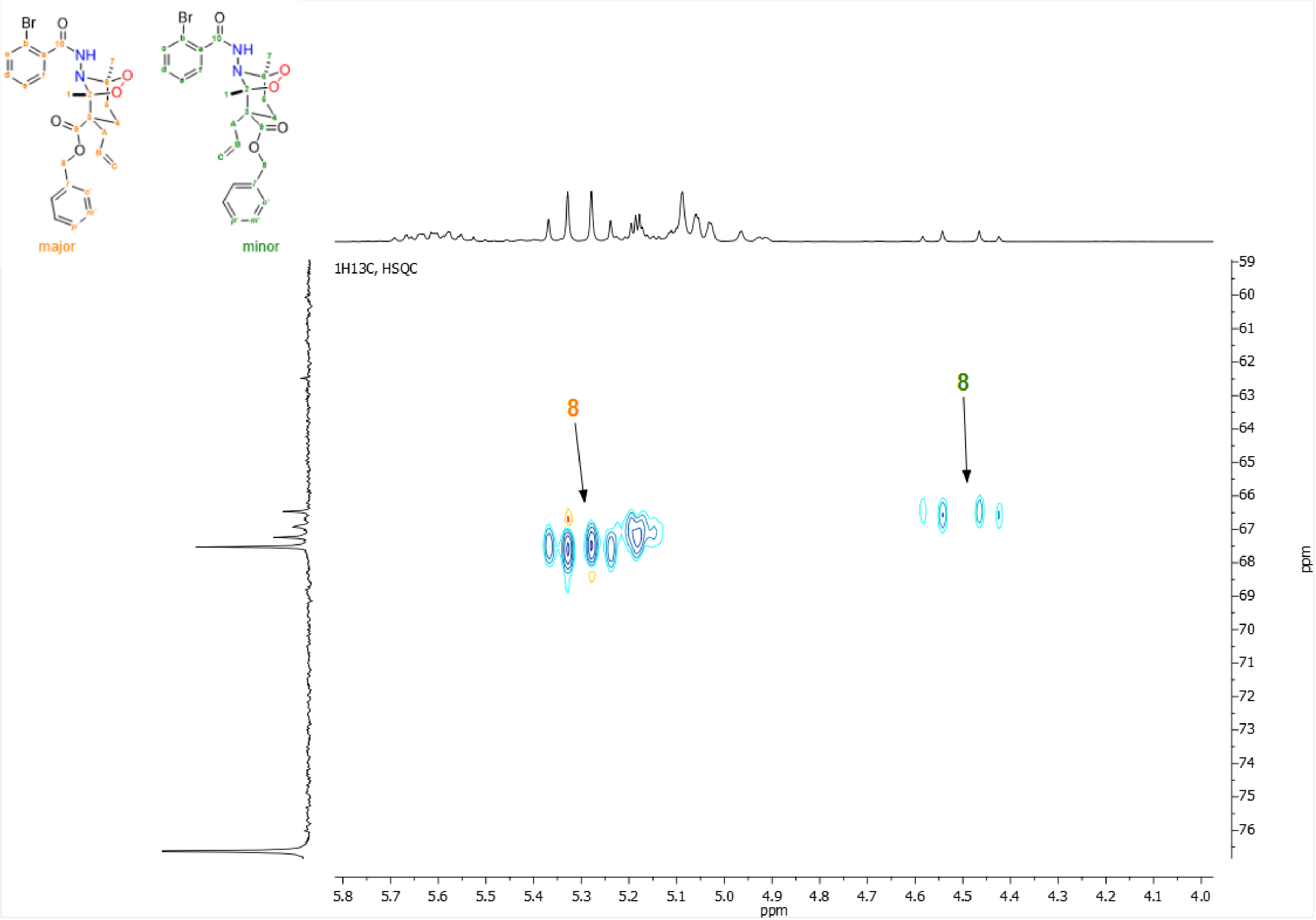

Benzyl 2-allyl-8-(2-bromobenzamido)-1,5-dimethyl-6,7-dioxa-8-azabicyclo[3.2.1]octane-2-carboxylate, 21a+21b

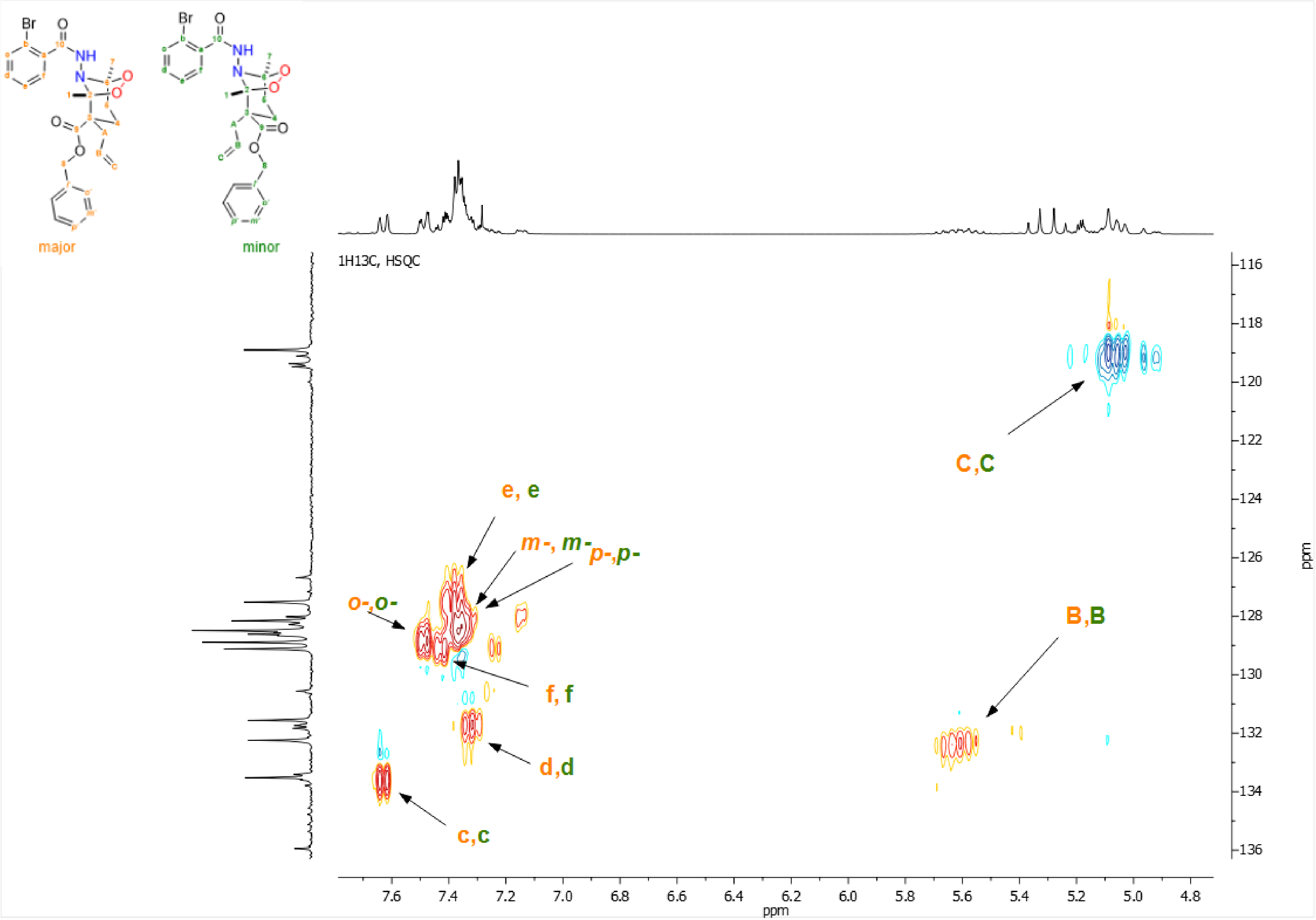

Benzyl 2-allyl-8-(2-bromobenzamido)-1,5-dimethyl-6,7-dioxa-8-azabicyclo[3.2.1]octane-2-carboxylate, 21a+21b

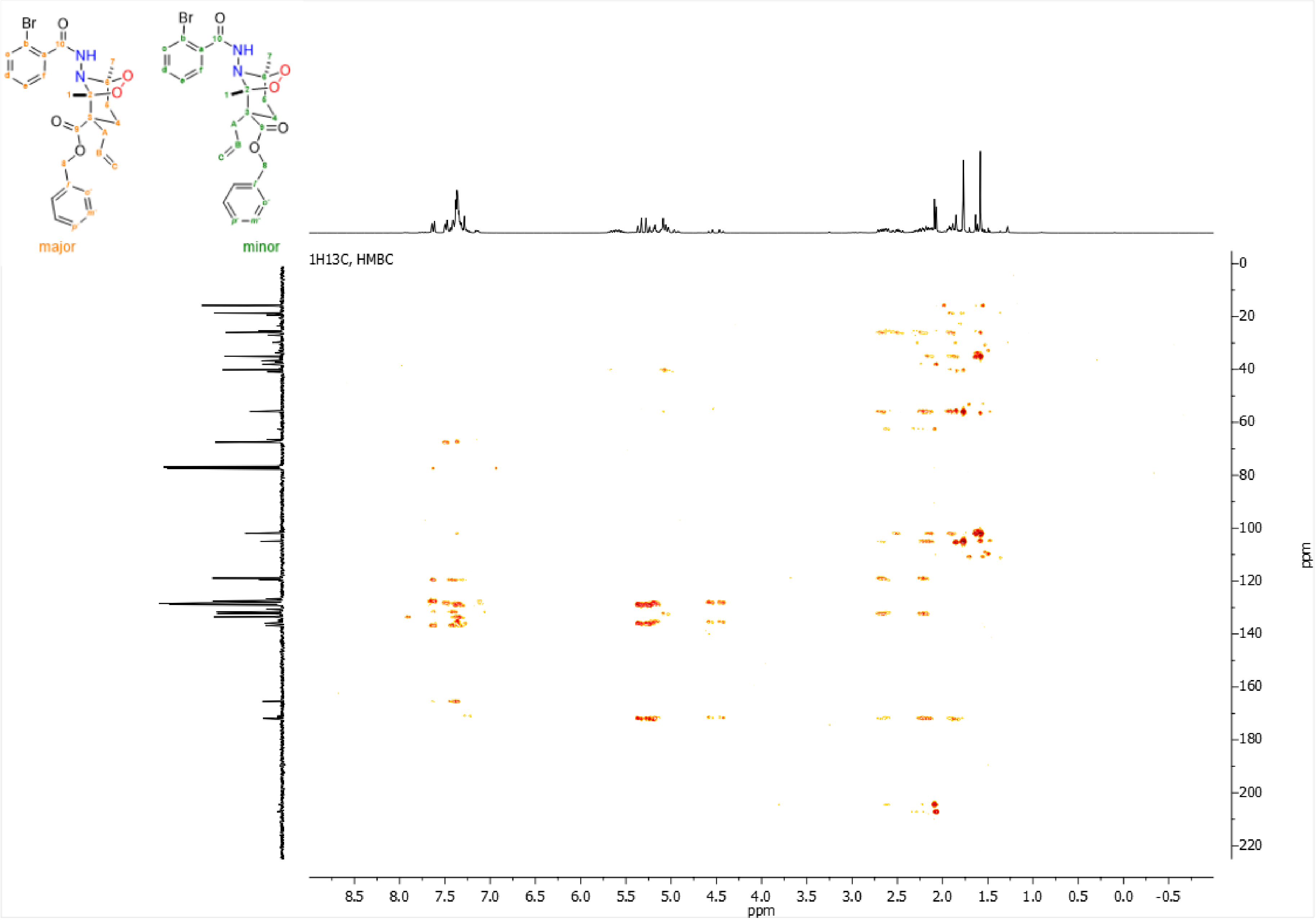

Benzyl 2-allyl-8-(2-bromobenzamido)-1,5-dimethyl-6,7-dioxa-8-azabicyclo[3.2.1]octane-2-carboxylate, 21a+21b

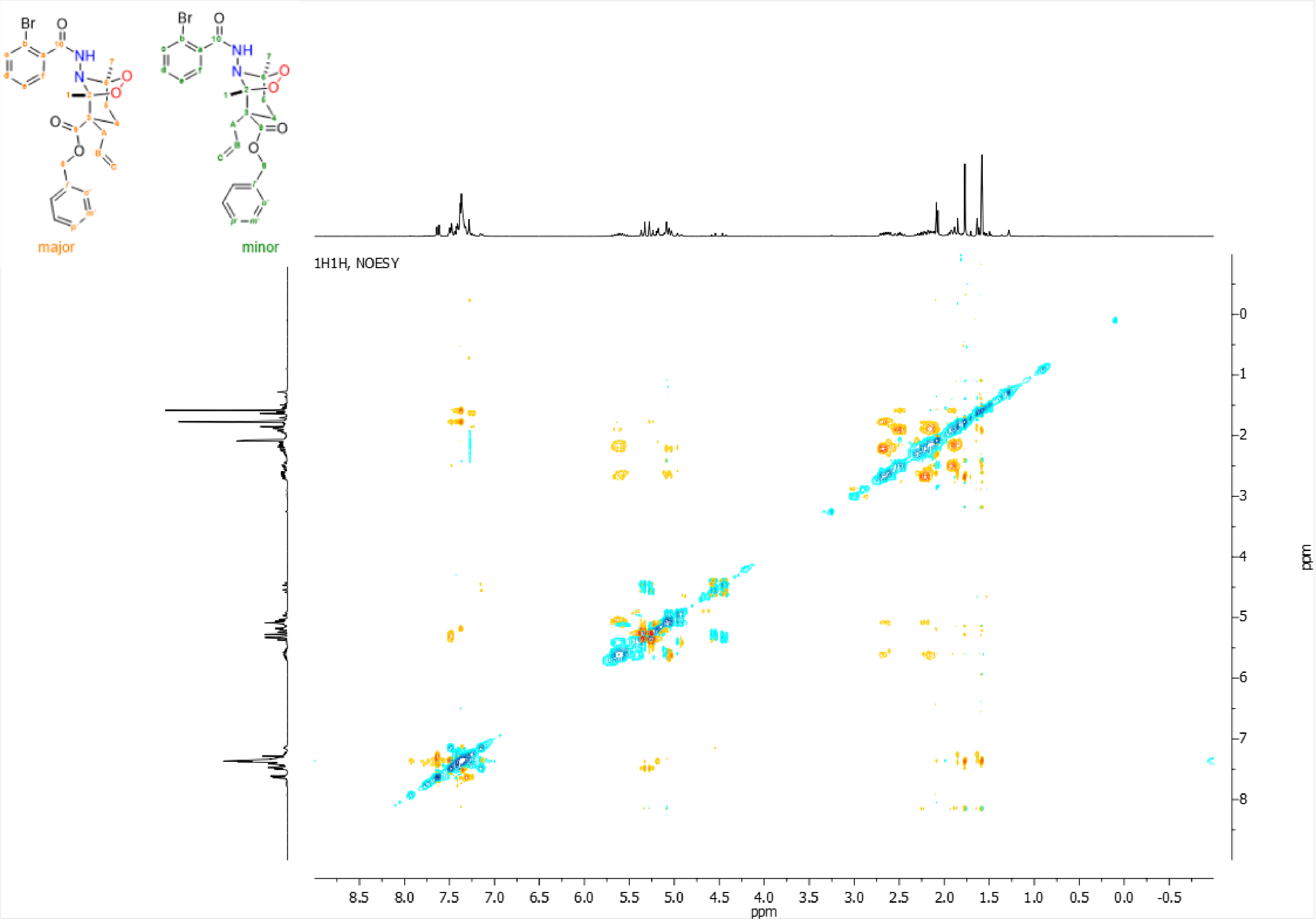

Benzyl 2-allyl-8-(2-bromobenzamido)-1,5-dimethyl-6,7-dioxa-8-azabicyclo[3.2.1]octane-2-carboxylate, 21a+21b

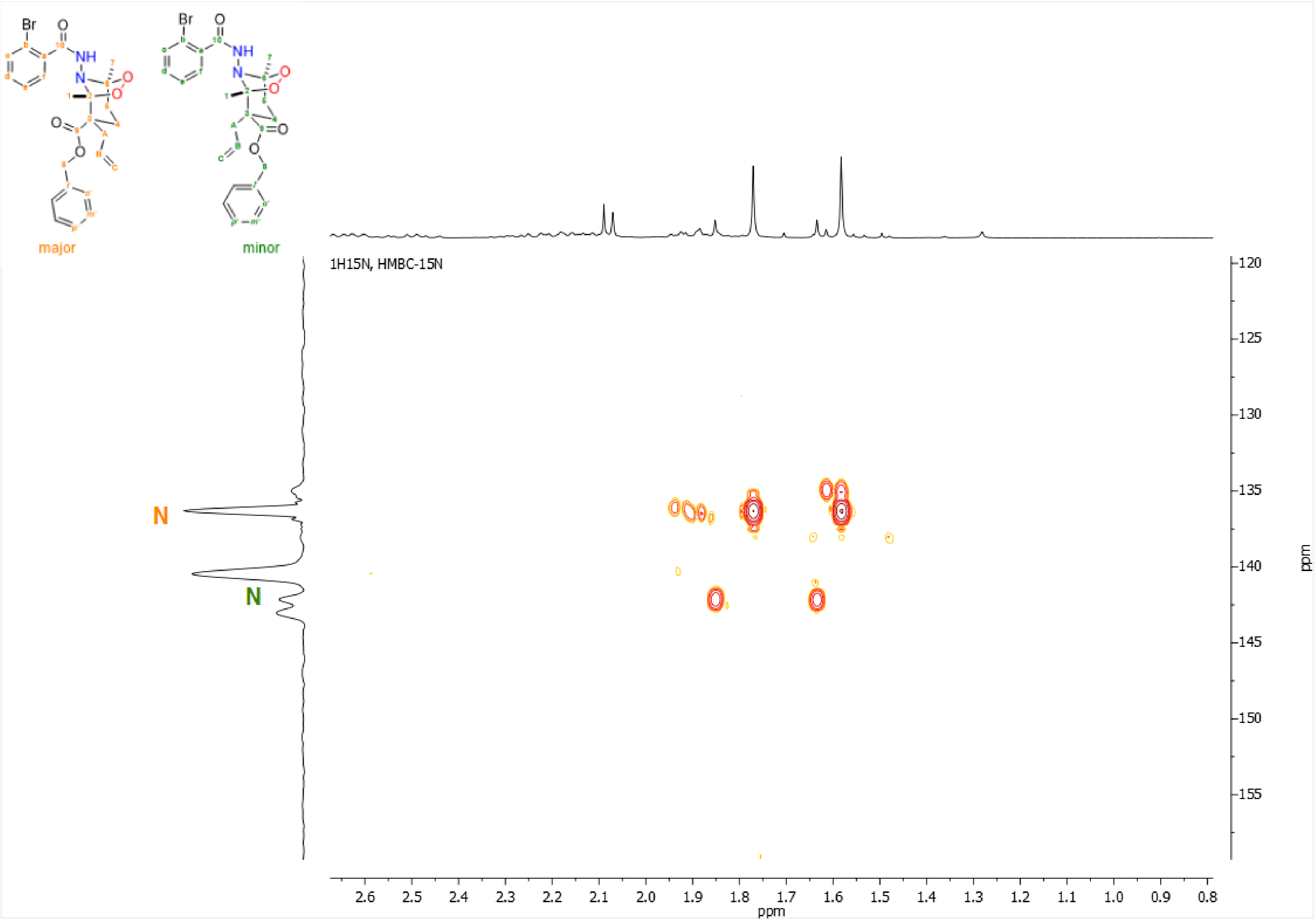

Benzyl 2-allyl-8-(2-bromobenzamido)-1,5-dimethyl-6,7-dioxa-8-azabicyclo[3.2.1]octane-2-carboxylate, 21a+21b

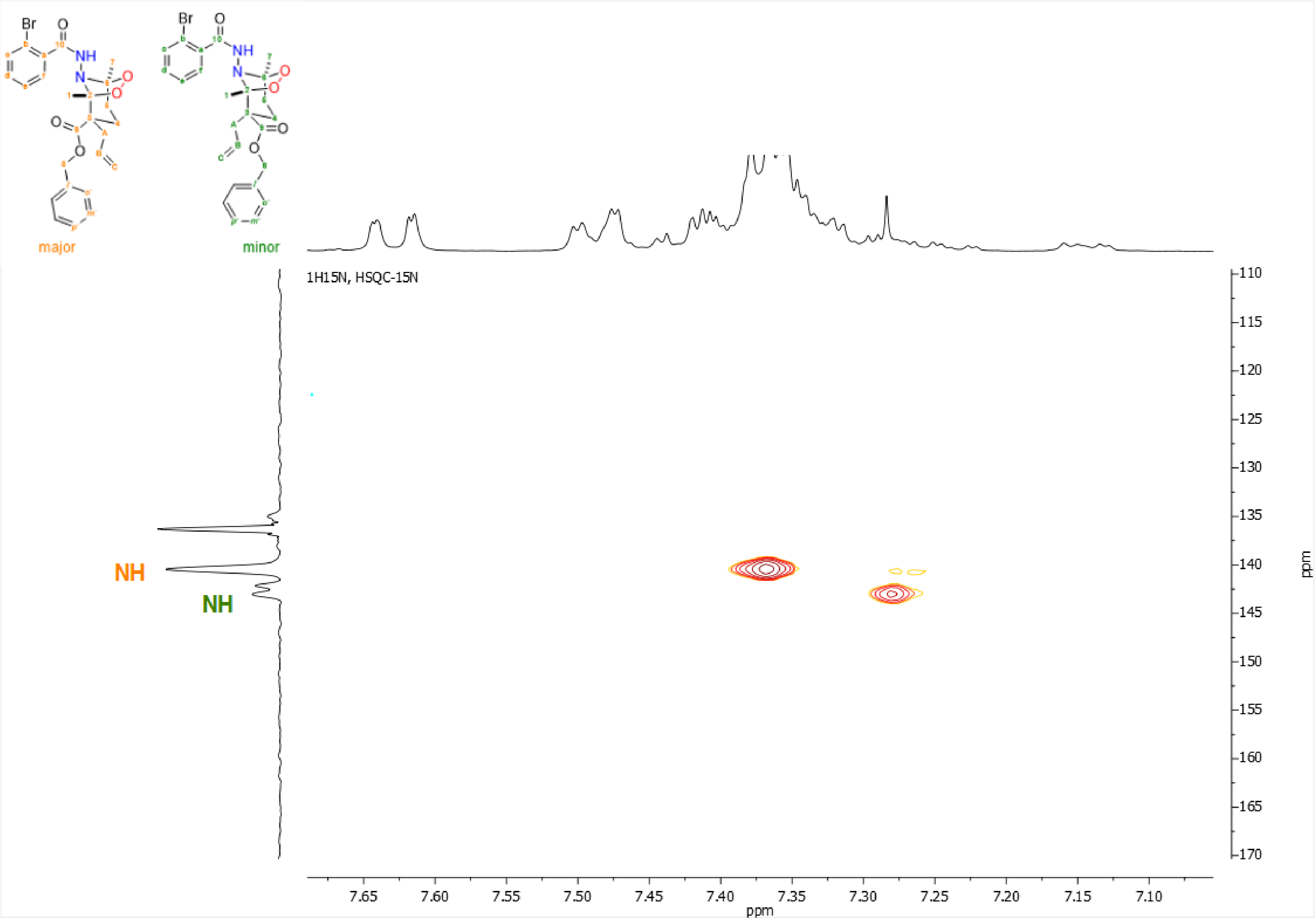

^{1}H NMR (300.13 MHz, $CDCl_3$). Ethyl 8-(2-bromobenzamido)-2-butyl-1,5-dimethyl-6,7-dioxa-8-azabicyclo[3.2.1]octane-2-carboxylate, 22a+22b

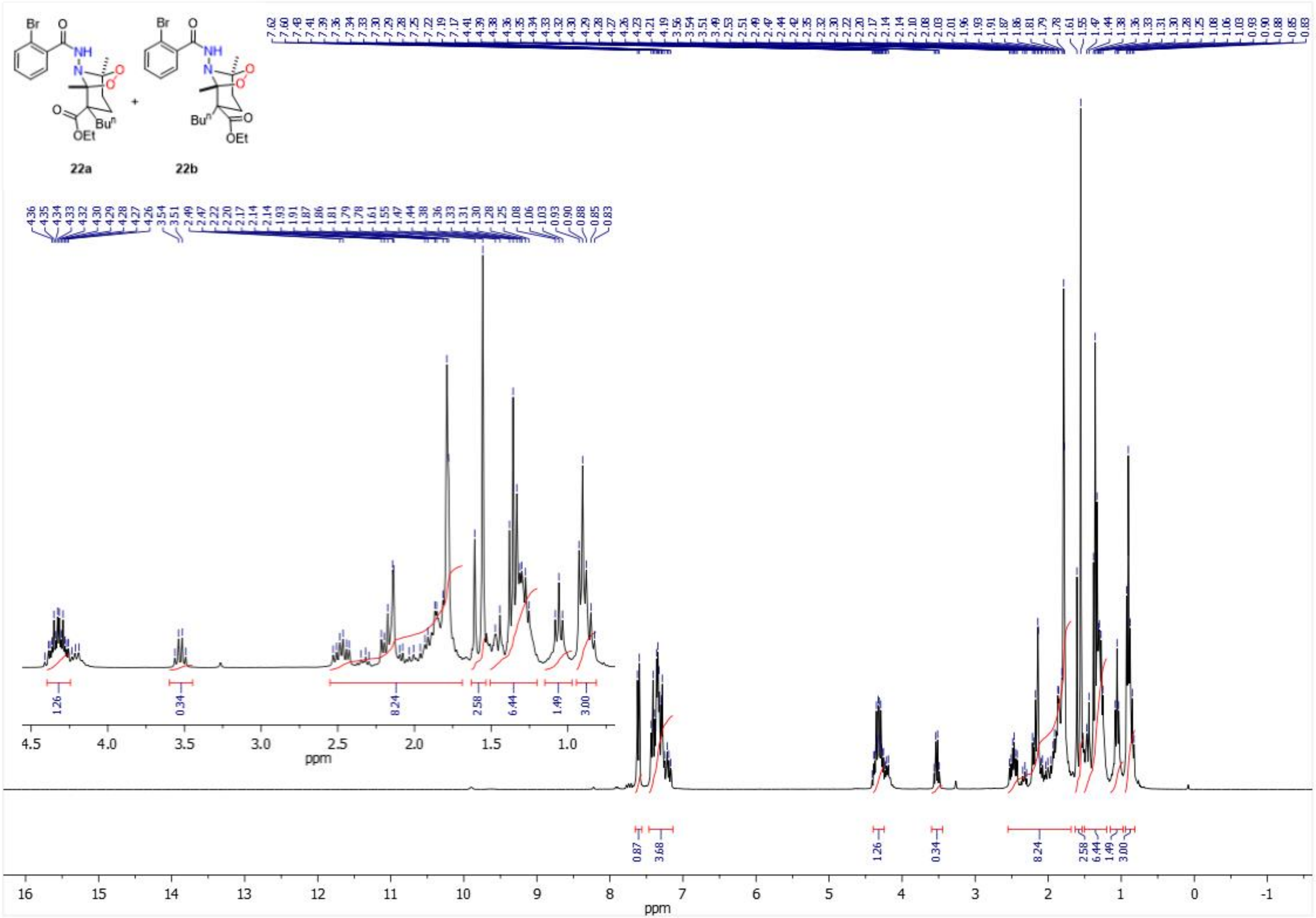

^{13}C NMR (75.48 MHz, CDCl$_3$). Ethyl 8-(2-bromobenzamido)-2-butyl-1,5-dimethyl-6,7-dioxa-8-azabicyclo[3.2.1]octane-2-carboxylate, 22a+22b

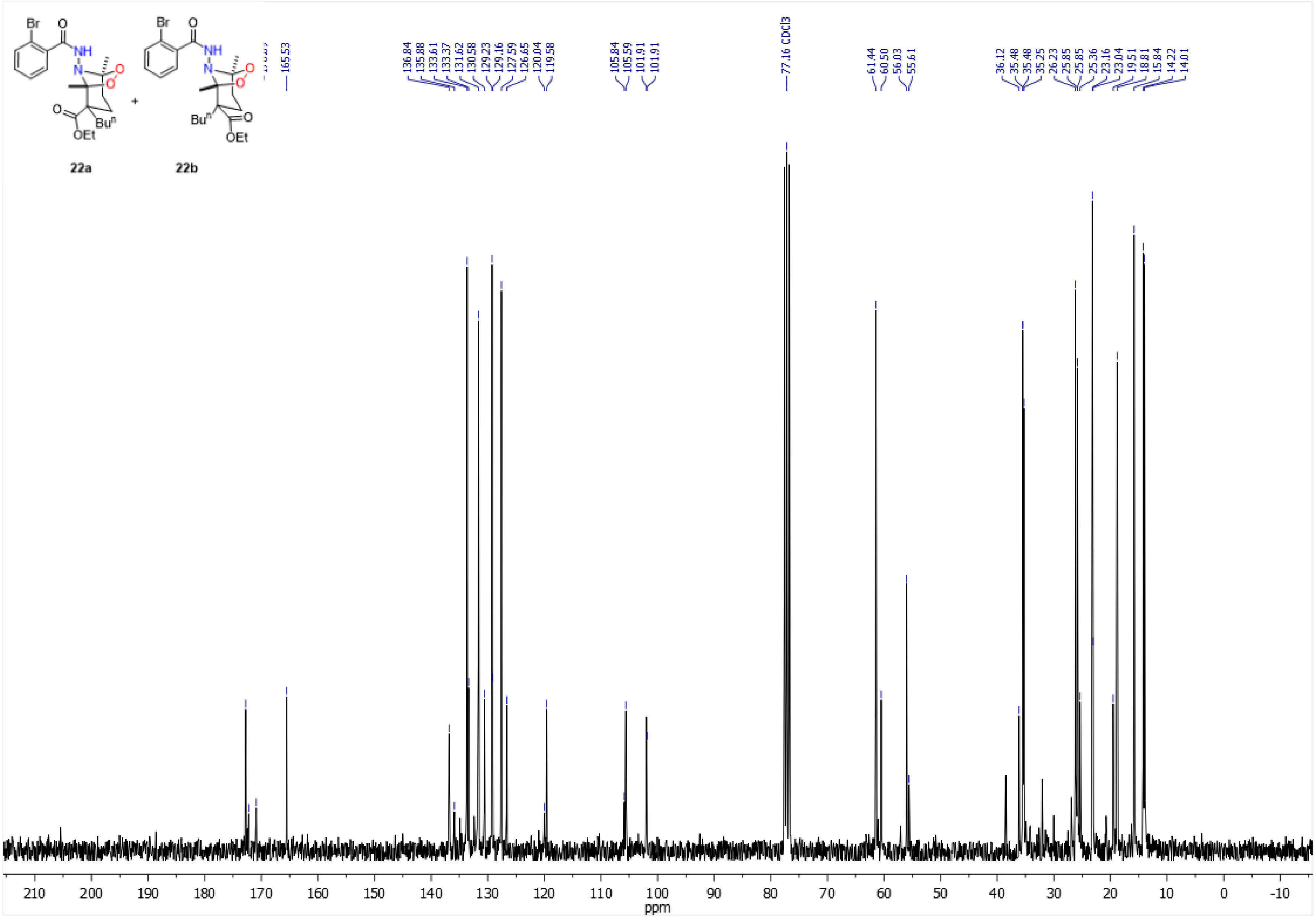

^{1}H NMR (300.13 MHz, CDCl3). Ethyl 2-allyl-8-(2-bromobenzamido)-1,5-dimethyl-6,7-dioxa-8-azabicyclo[3.2.1]octane-2-carboxylate, 23a + 23b

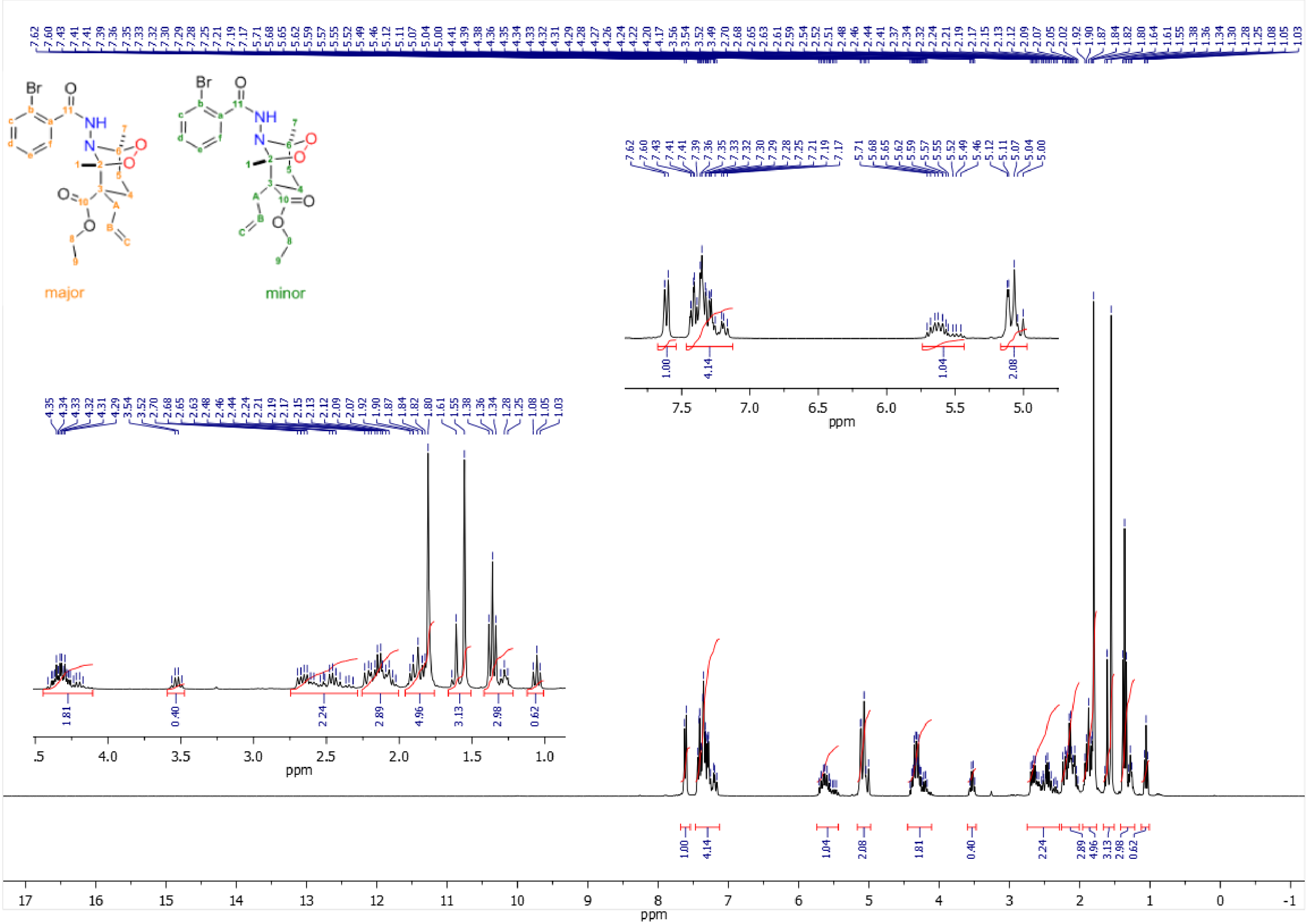

^{1}H NMR (300.13 MHz, $CDCl_3$). Ethyl 2-allyl-8-(2-bromobenzamido)-1,5-dimethyl-6,7-dioxa-8-azabicyclo[3.2.1]octane-2-carboxylate, 23a + 23b

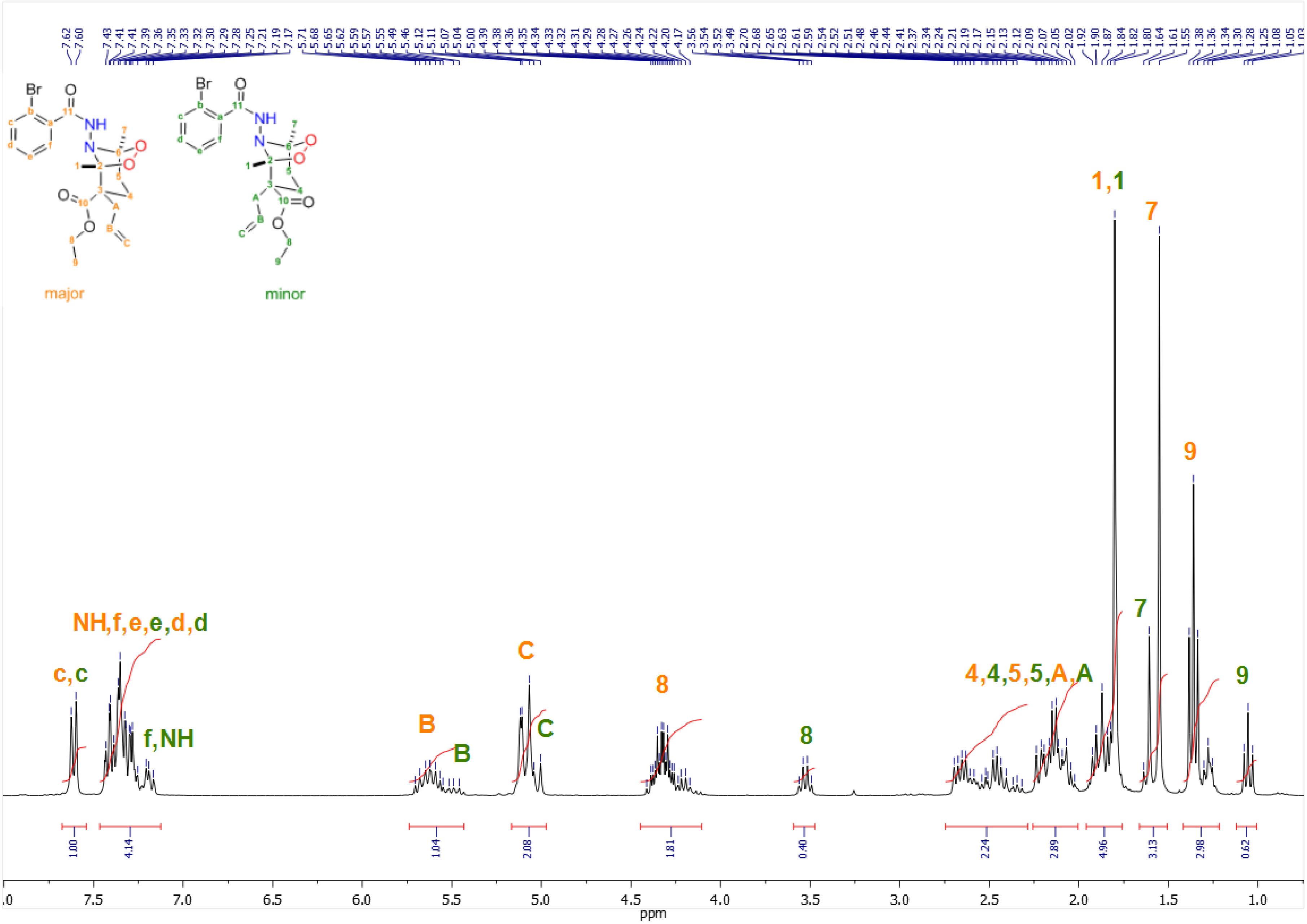

^{13}C NMR (75.48 MHz, $CDCl_3$). Ethyl 2-allyl-8-(2-bromobenzamido)-1,5-dimethyl-6,7-dioxa-8-azabicyclo[3.2.1]octane-2-carboxylate, 23a + 23b

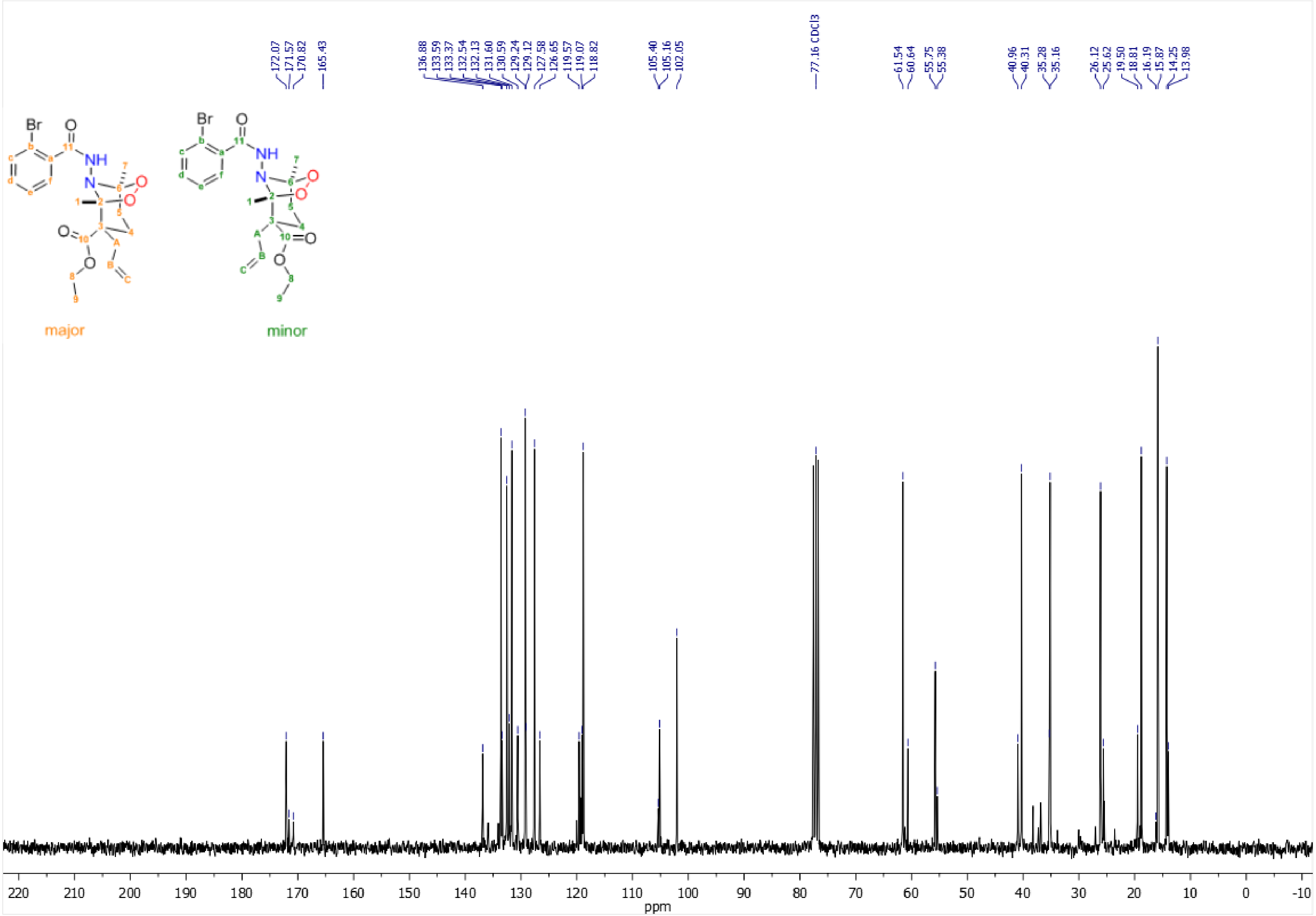

^{13}C NMR (75.48 MHz, CDCl3). Ethyl 2-allyl-8-(2-bromobenzamido)-1,5-dimethyl-6,7-dioxa-8-azabicyclo[3.2.1]octane-2-carboxylate, 23a + 23b

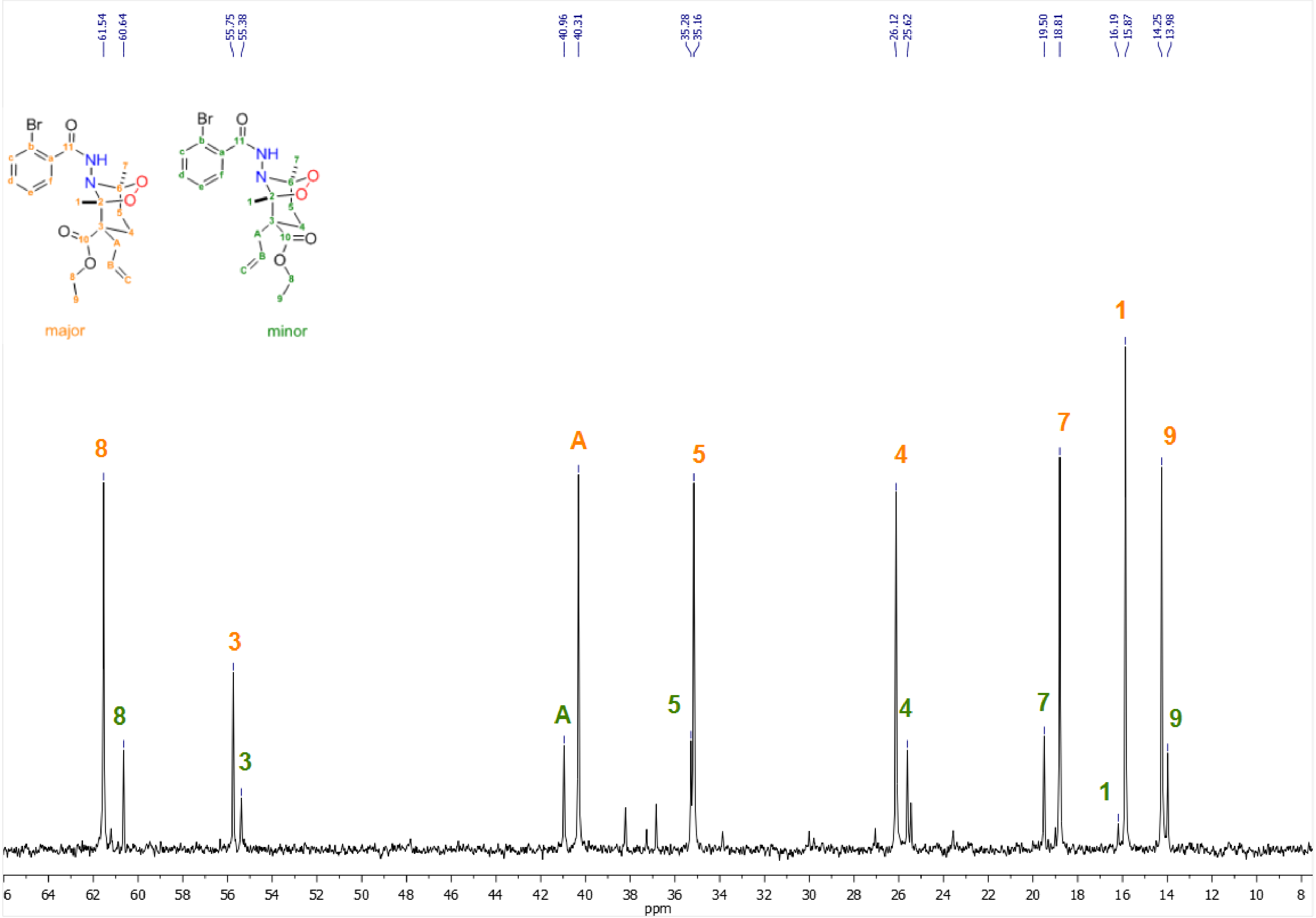

^{13}C NMR (75.48 MHz, $CDCl_3$). Ethyl 2-allyl-8-(2-bromobenzamido)-1,5-dimethyl-6,7-dioxa-8-azabicyclo[3.2.1]octane-2-carboxylate, 23a + 23b

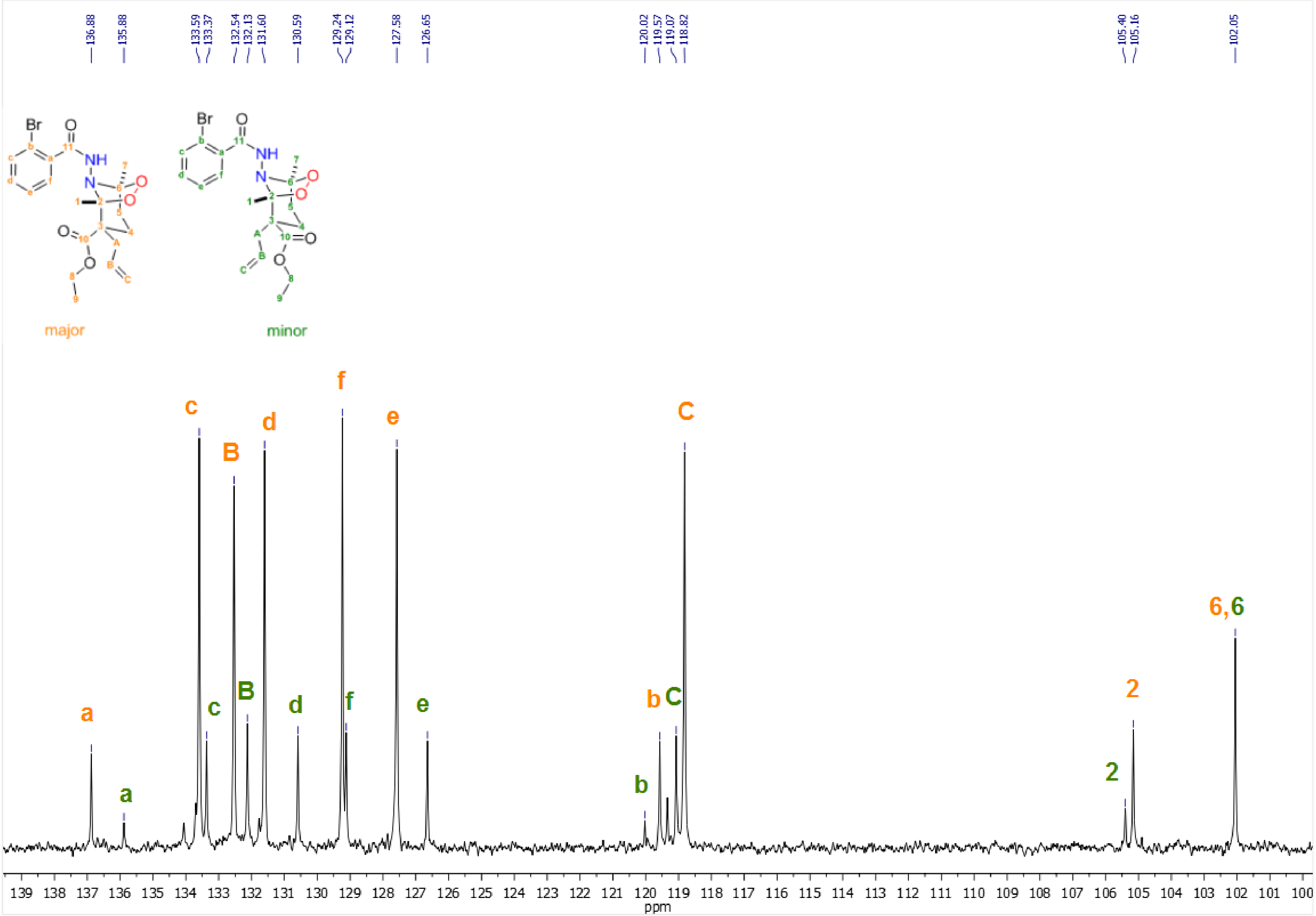

^{13}C NMR (75.48 MHz, CDCl$_3$). Ethyl 2-allyl-8-(2-bromobenzamido)-1,5-dimethyl-6,7-dioxa-8-azabicyclo[3.2.1]octane-2-carboxylate, 23a + 23b

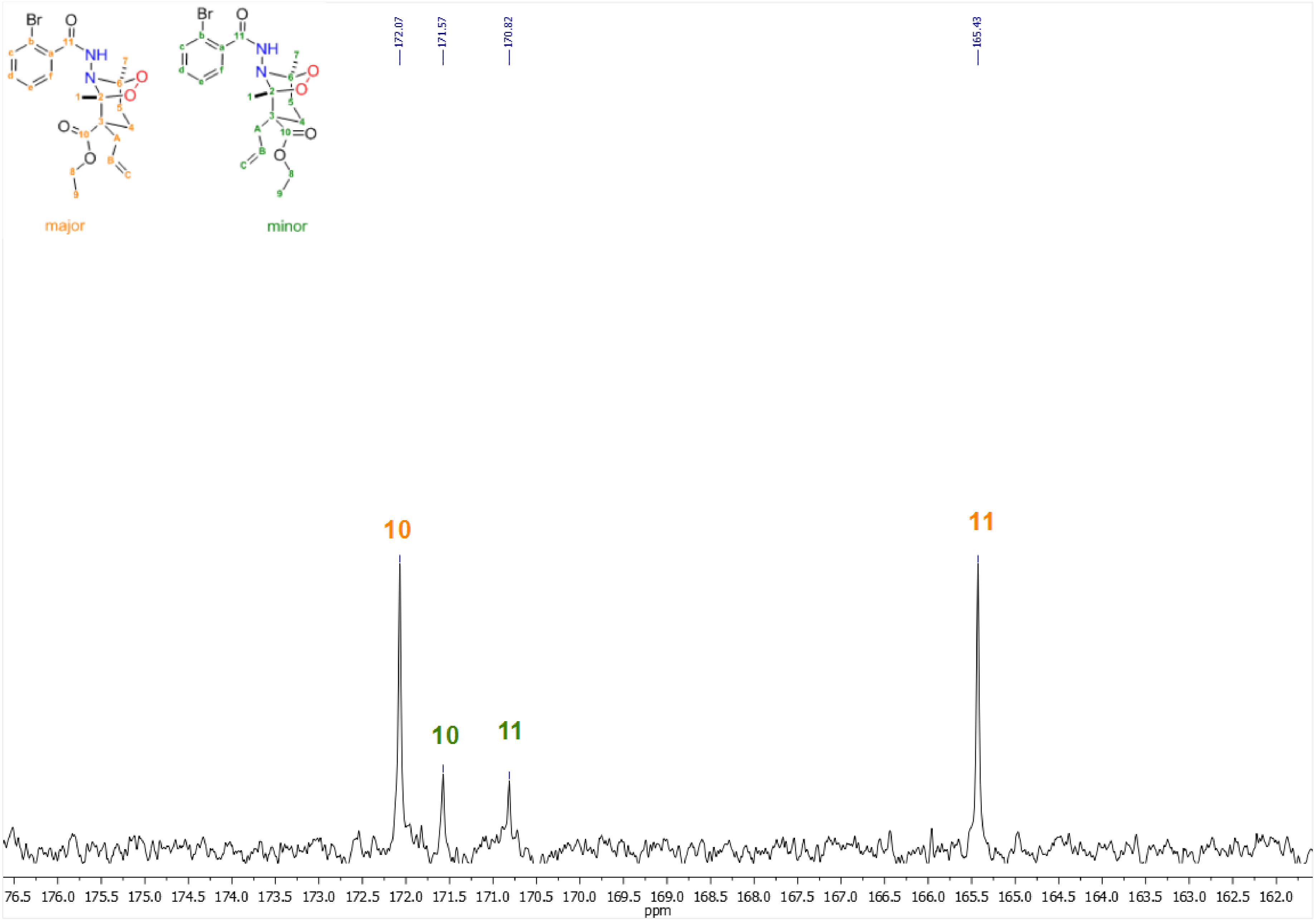

^{15}N (40.56 MHz, CDCl$_3$). Ethyl 2-allyl-8-(2-bromobenzamido)-1,5-dimethyl-6,7-dioxa-8-azabicyclo[3.2.1]octane-2-carboxylate, 23a + 23b

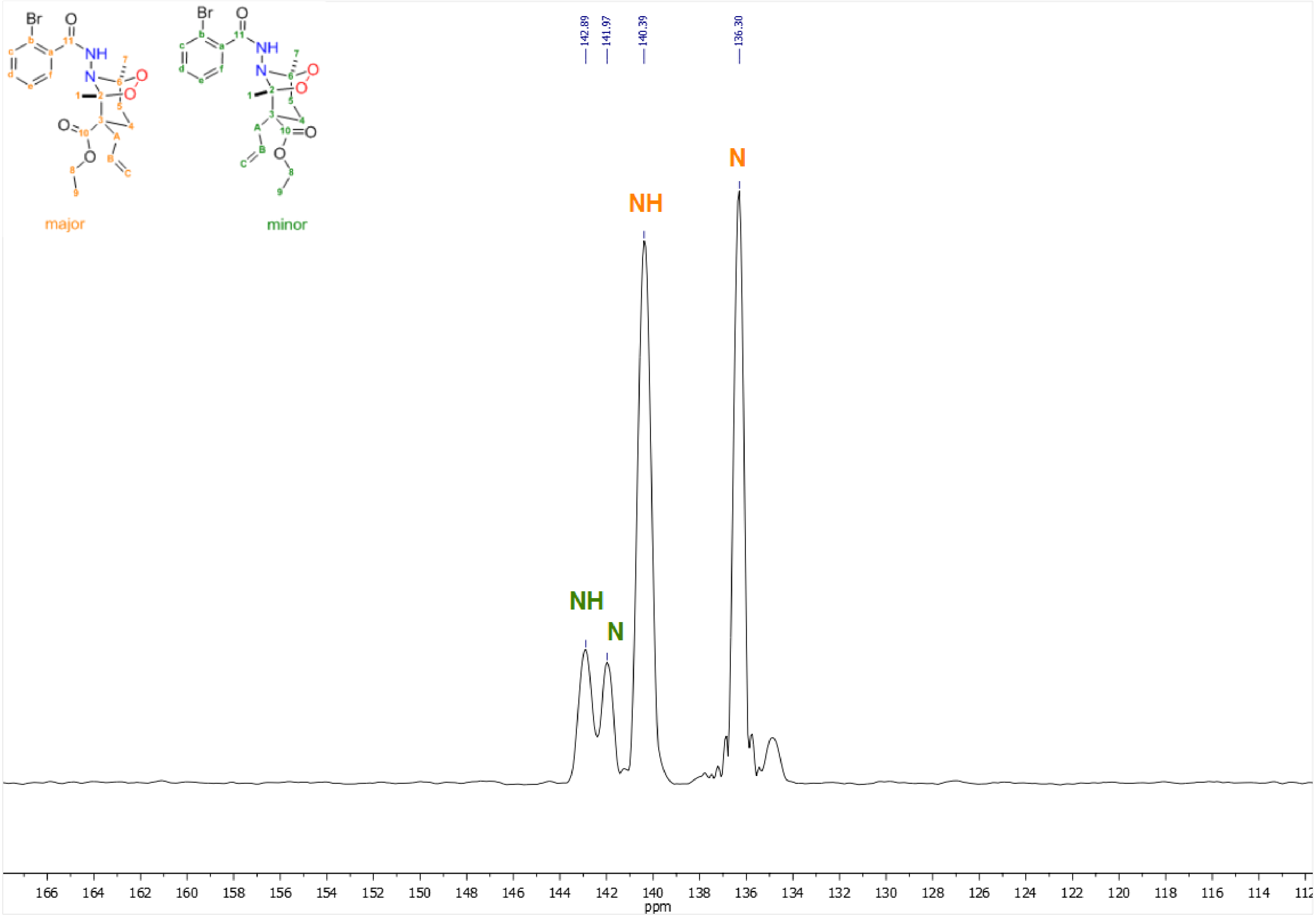

^{13}C NMR (75.48 MHz, CDCl3). Ethyl 2-allyl-8-(2-bromobenzamido)-1,5-dimethyl-6,7-dioxa-8-azabicyclo[3.2.1]octane-2-carboxylate, 23a + 23b

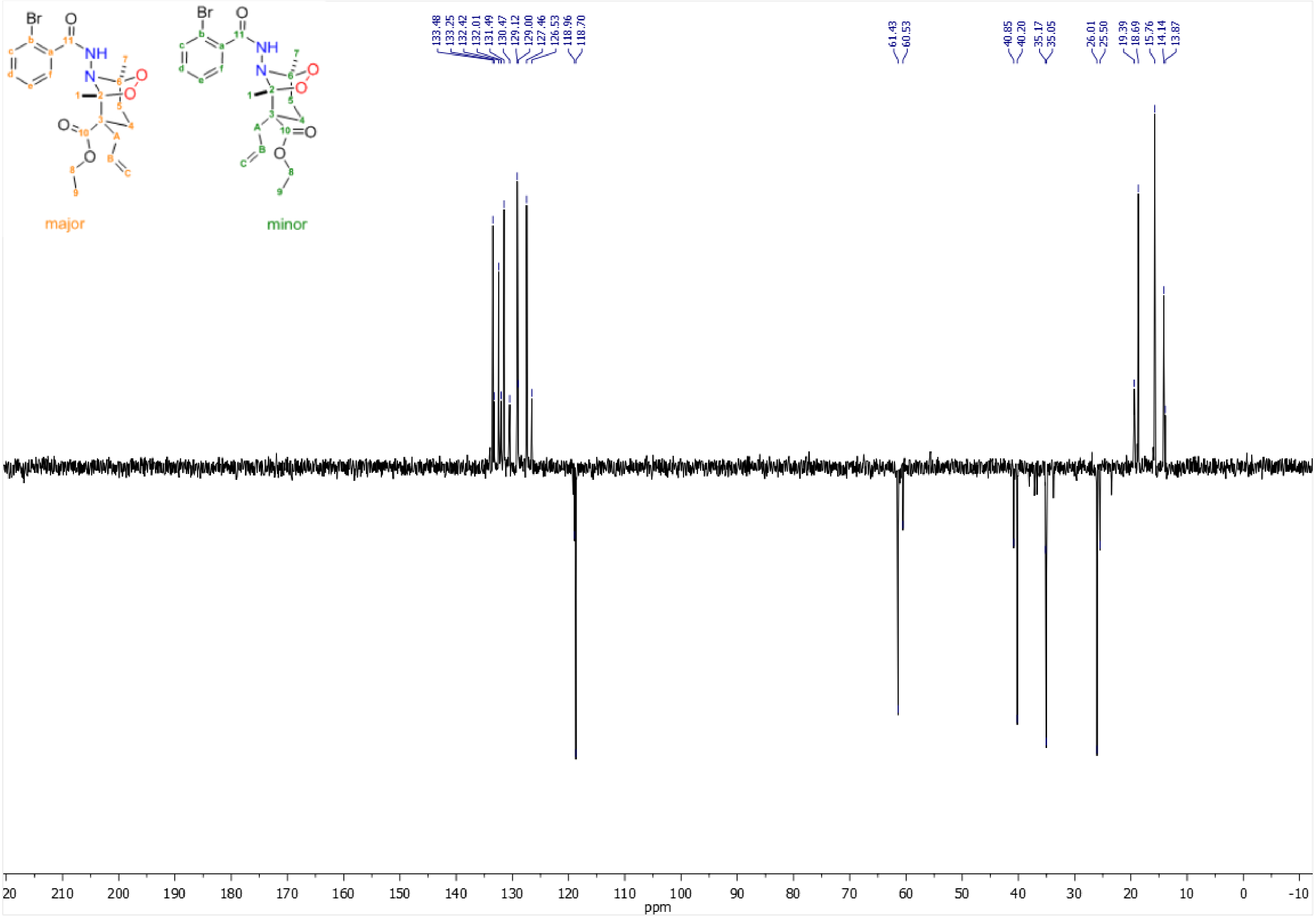

Ethyl 2-allyl-8-(2-bromobenzamido)-1,5-dimethyl-6,7-dioxa-8-azabicyclo[3.2.1]octane-2-carboxylate, 23a + 23b

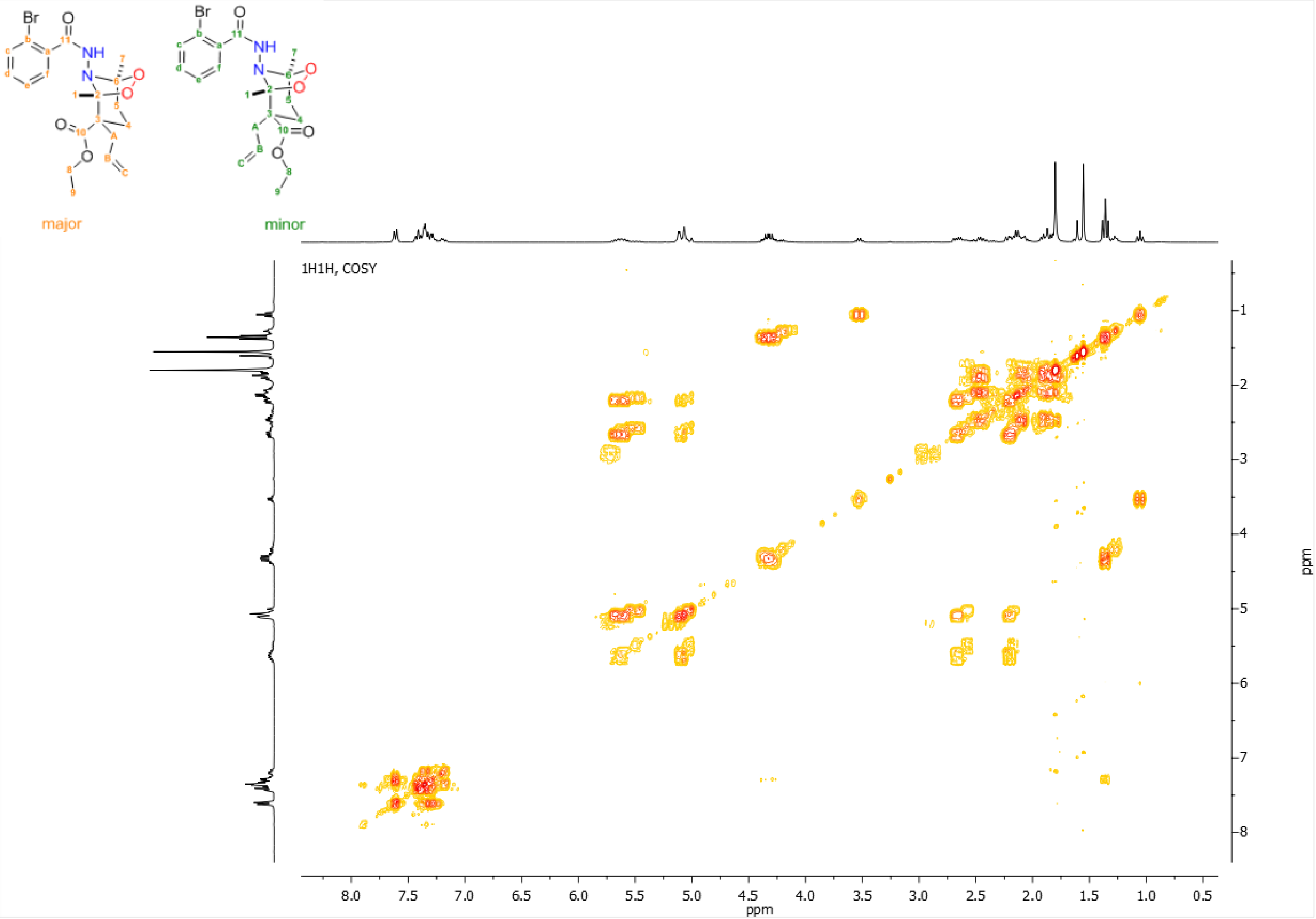

Ethyl 2-allyl-8-(2-bromobenzamido)-1,5-dimethyl-6,7-dioxa-8-azabicyclo[3.2.1]octane-2-carboxylate, 23a + 23b

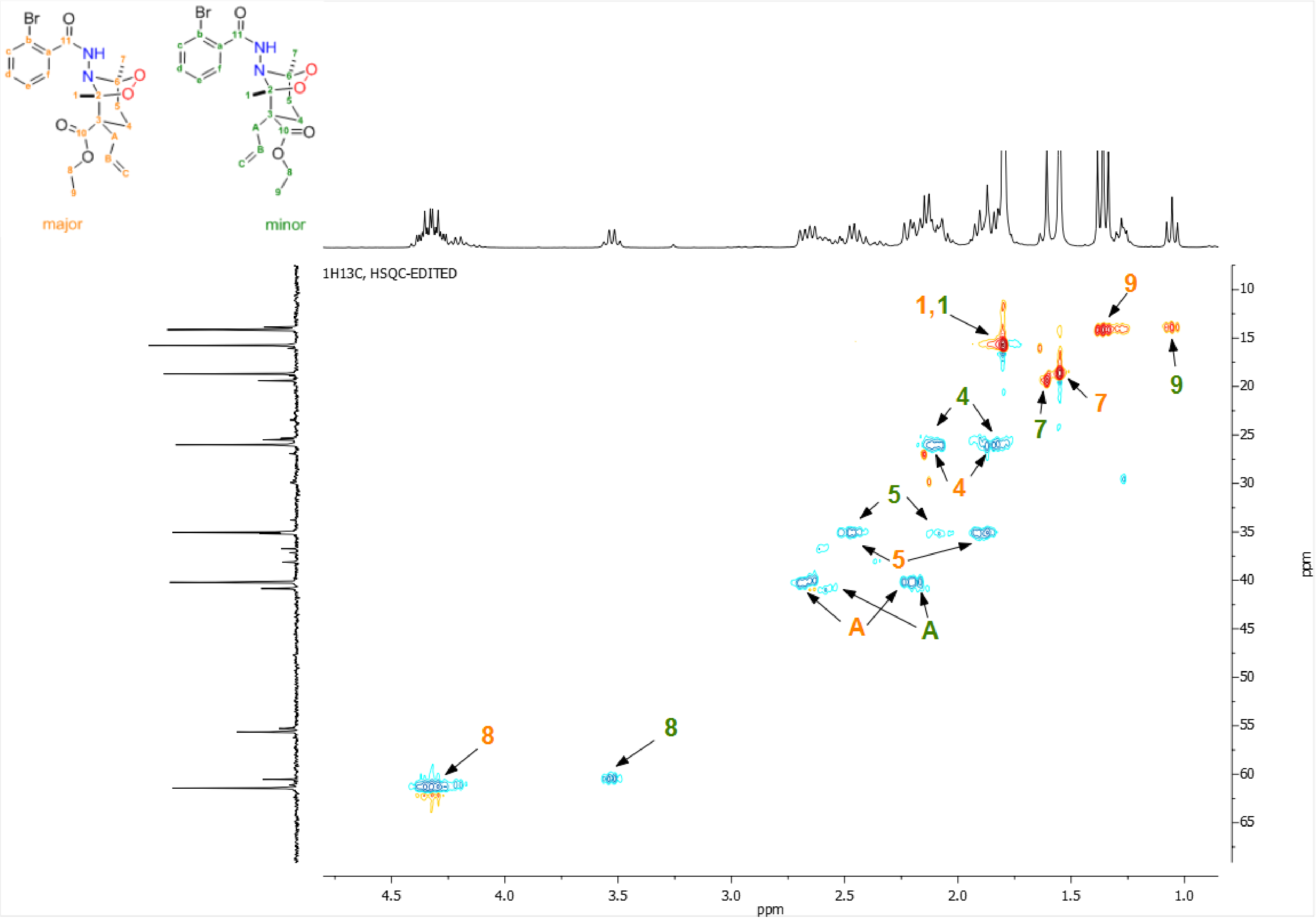

Ethyl 2-allyl-8-(2-bromobenzamido)-1,5-dimethyl-6,7-dioxa-8-azabicyclo[3.2.1]octane-2-carboxylate, 23a + 23b

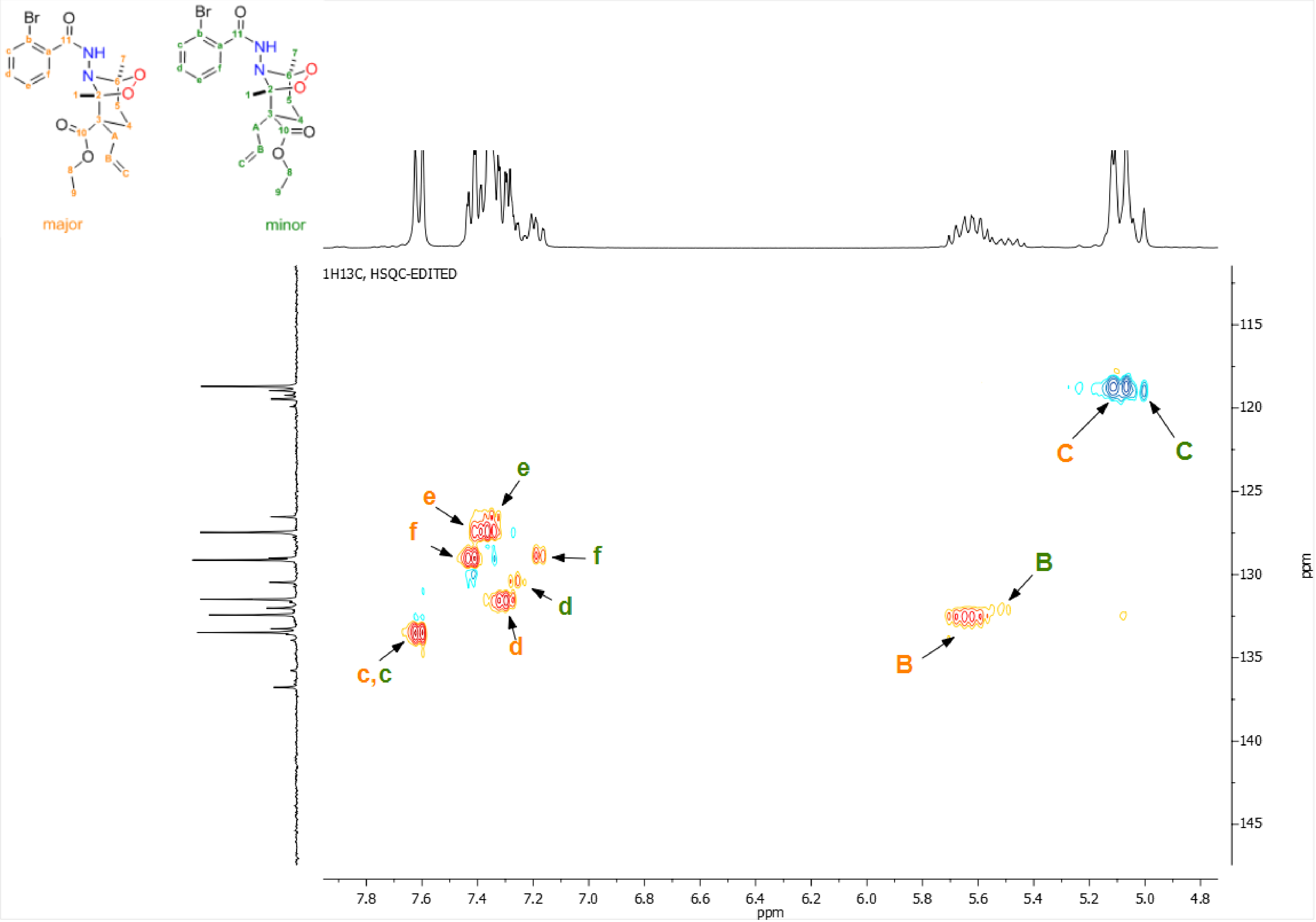

Ethyl 2-allyl-8-(2-bromobenzamido)-1,5-dimethyl-6,7-dioxa-8-azabicyclo[3.2.1]octane-2-carboxylate, 23a + 23b

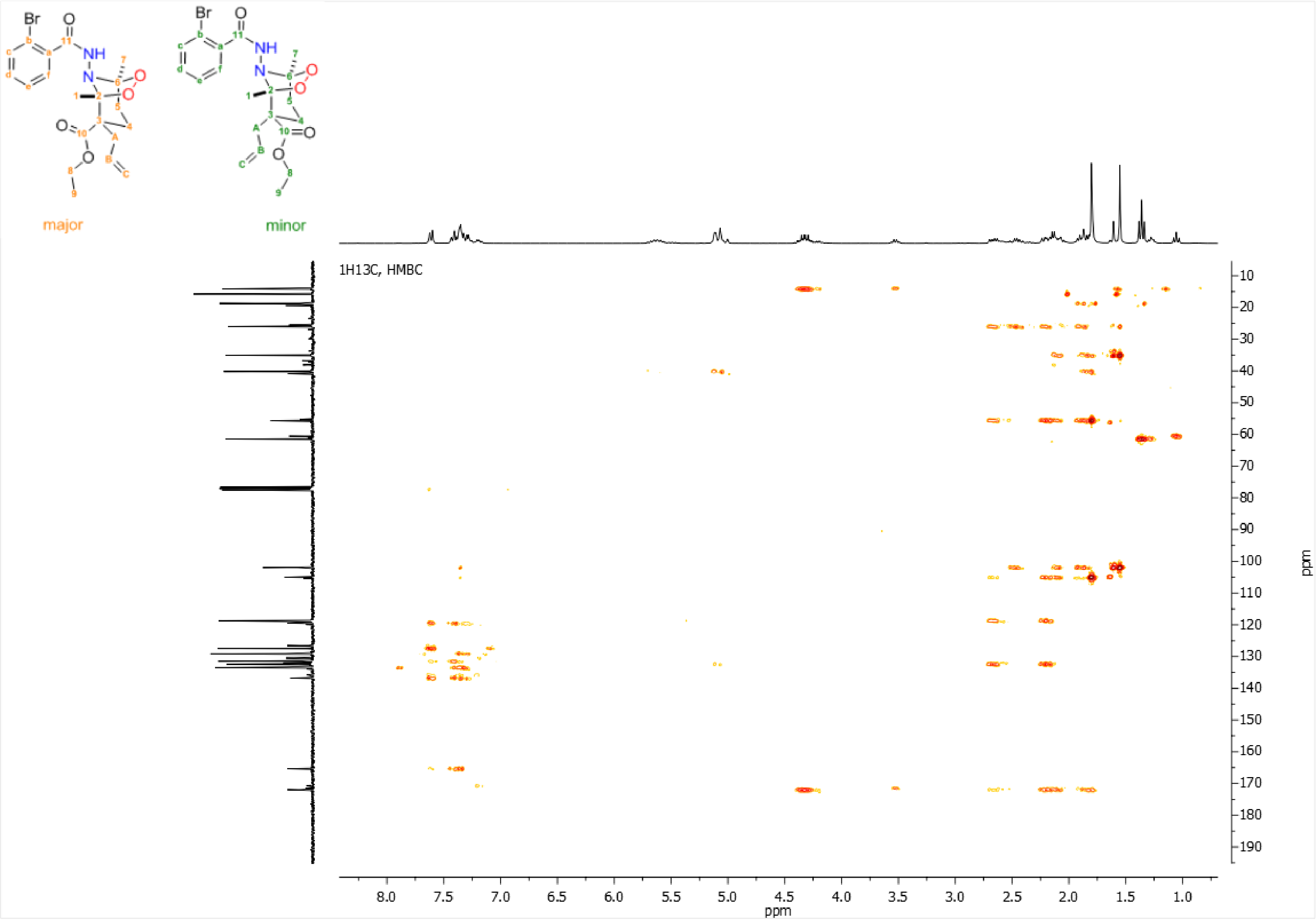

Ethyl 2-allyl-8-(2-bromobenzamido)-1,5-dimethyl-6,7-dioxa-8-azabicyclo[3.2.1]octane-2-carboxylate, 23a + 23b

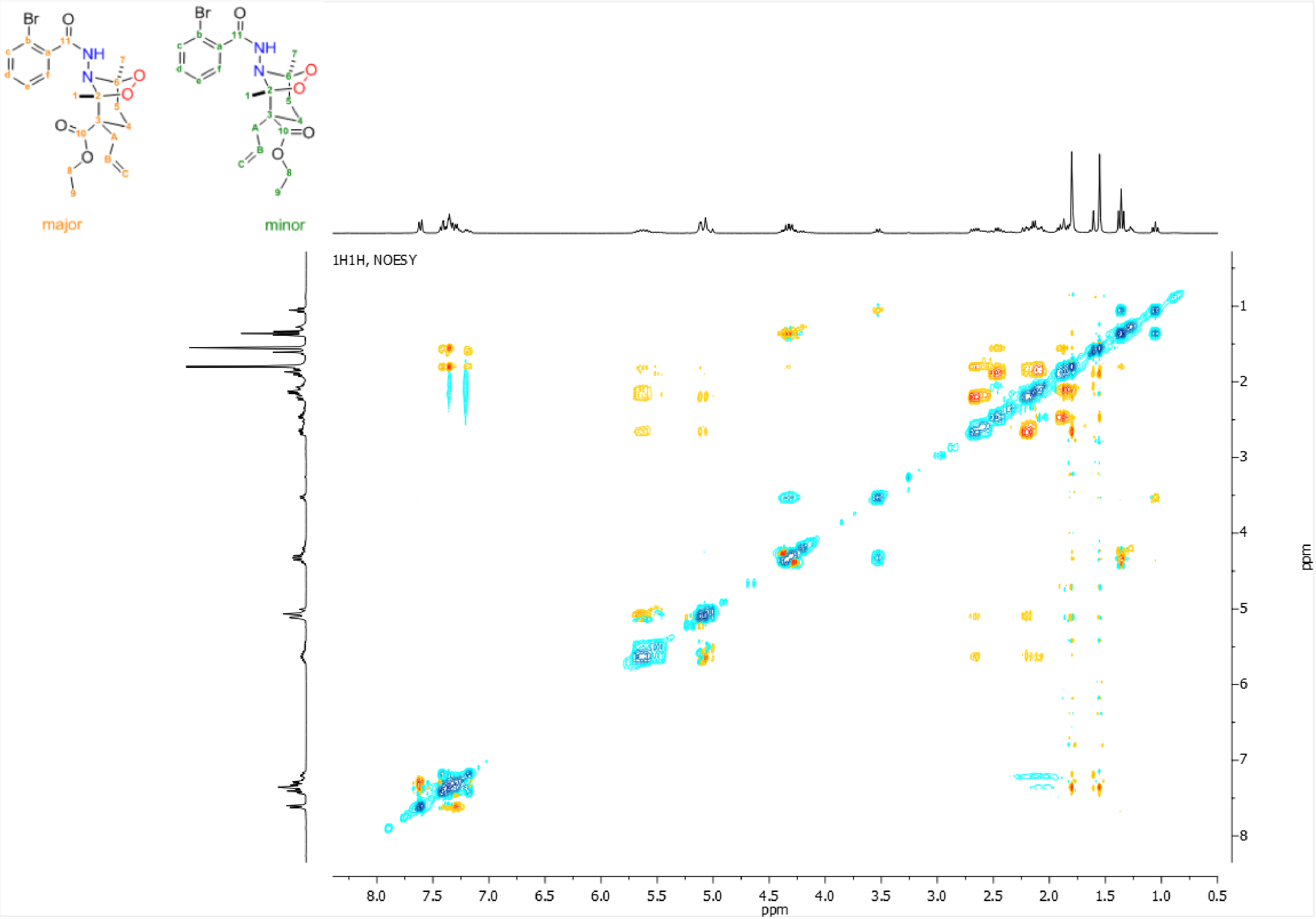

Ethyl 2-allyl-8-(2-bromobenzamido)-1,5-dimethyl-6,7-dioxa-8-azabicyclo[3.2.1]octane-2-carboxylate, 23a + 23b

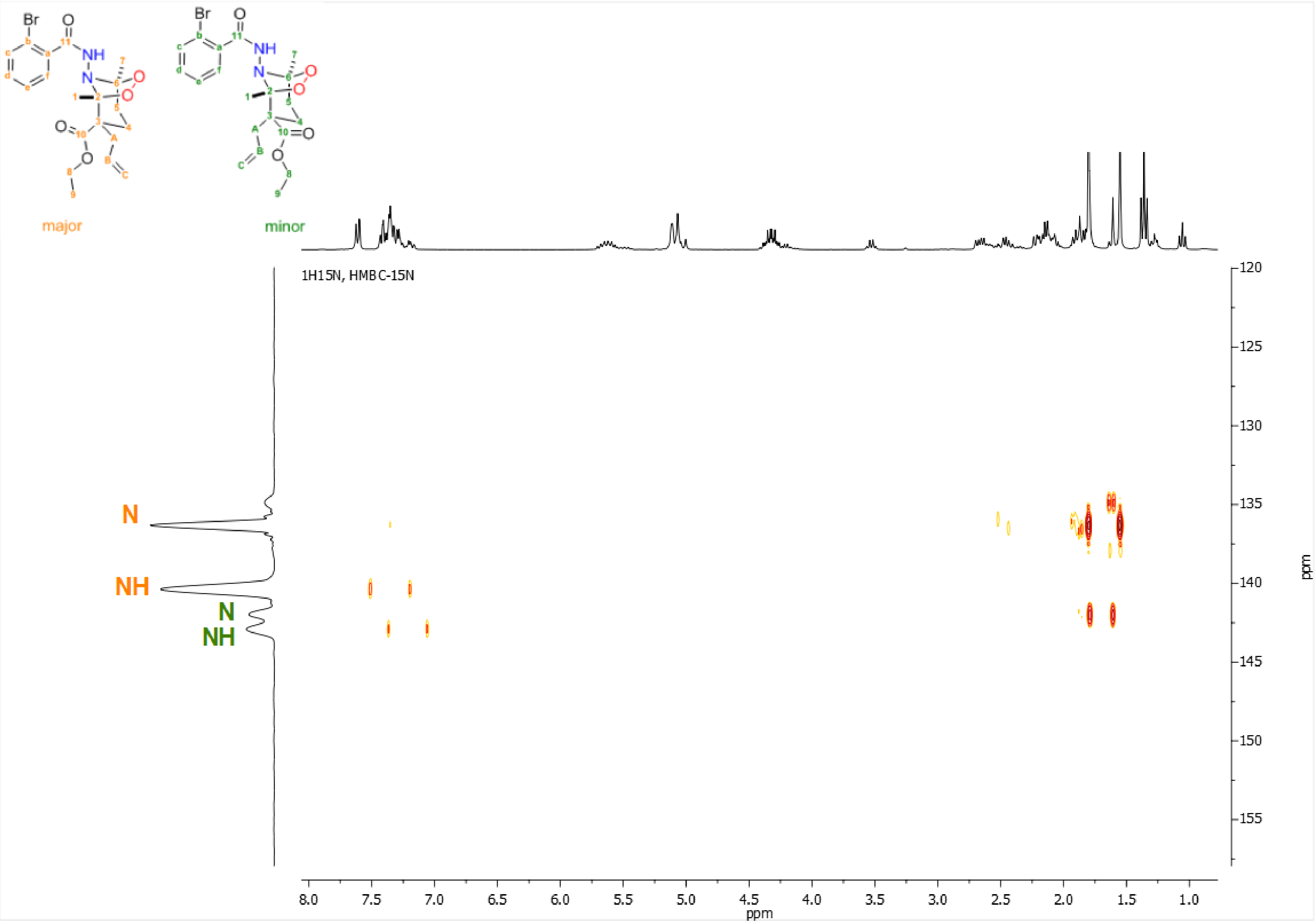

Ethyl 2-allyl-8-(2-bromobenzamido)-1,5-dimethyl-6,7-dioxa-8-azabicyclo[3.2.1]octane-2-carboxylate, 23a + 23b

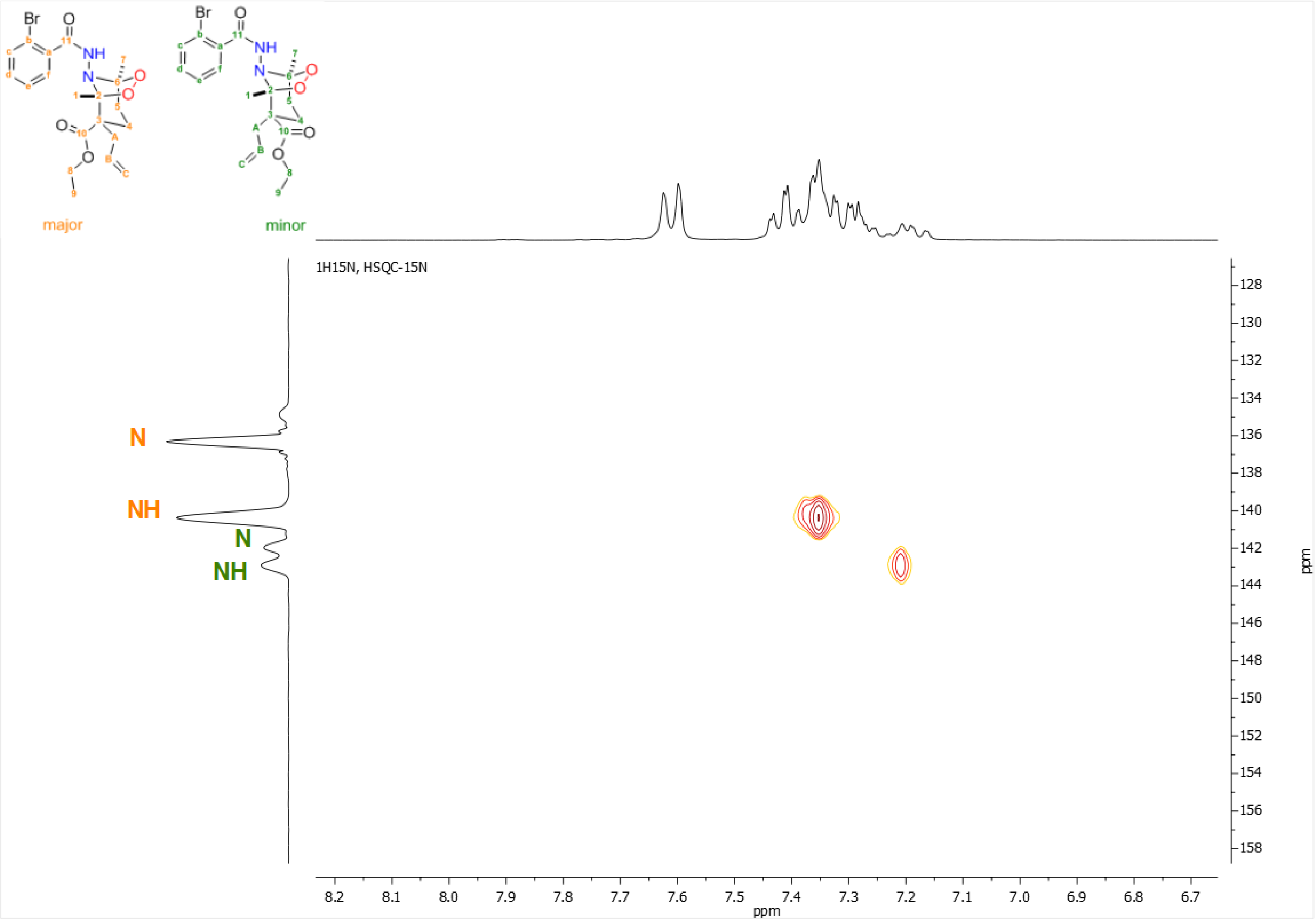

^{1}H NMR (300.13 MHz, CDCl3). 2-Bromo-N-(1,5-dimethyl-3-(4-nitrophenyl)-6,7-dioxa-8-azabicyclo[3.2.1]octan-8-yl)benzamide, 24

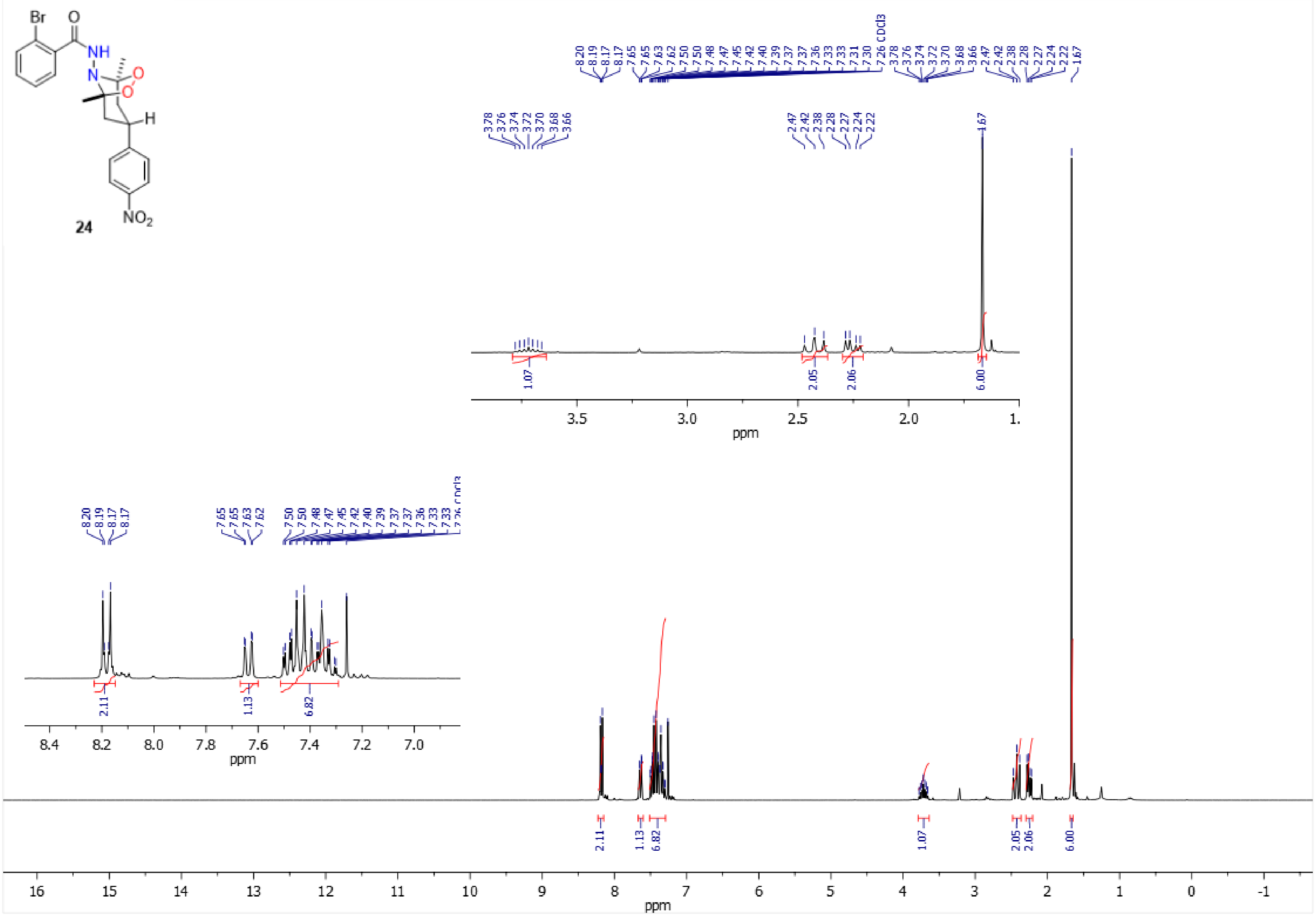

^{13}C NMR (75.48 MHz, CDCl$_3$). 2-Bromo-N-(1,5-dimethyl-3-(4-nitrophenyl)-6,7-dioxa-8-azabicyclo[3.2.1]octan-8-yl)benzamide, 24

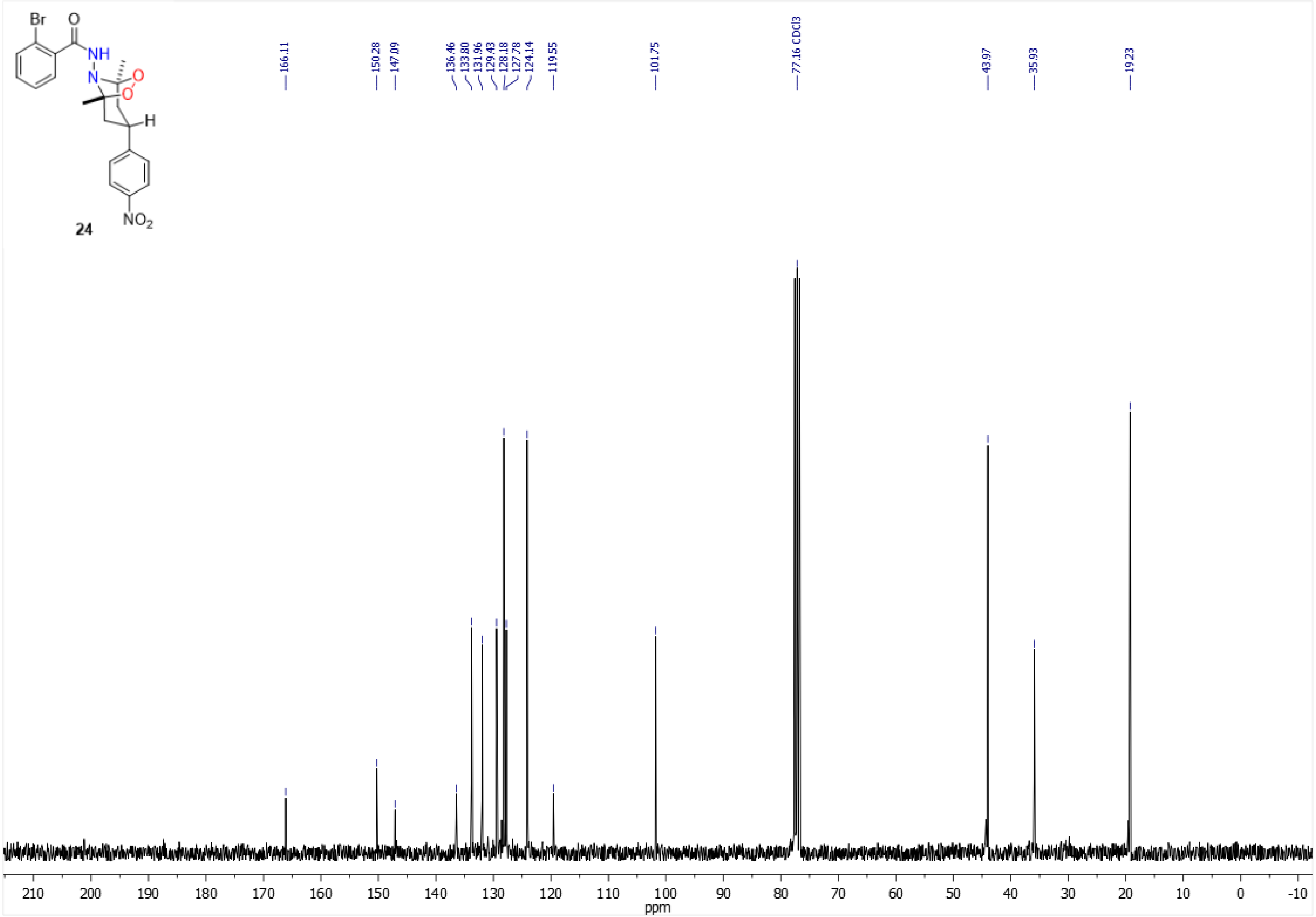

^{1}H NMR (300.13 MHz, $CDCl_3$). Ethyl 2-(4-chlorobenzyl)-8-(2-cyanoacetamido)-1,5-dimethyl-6,7-dioxa-8-azabicyclo[3.2.1]octane-2-carboxylate, 25a + 25b

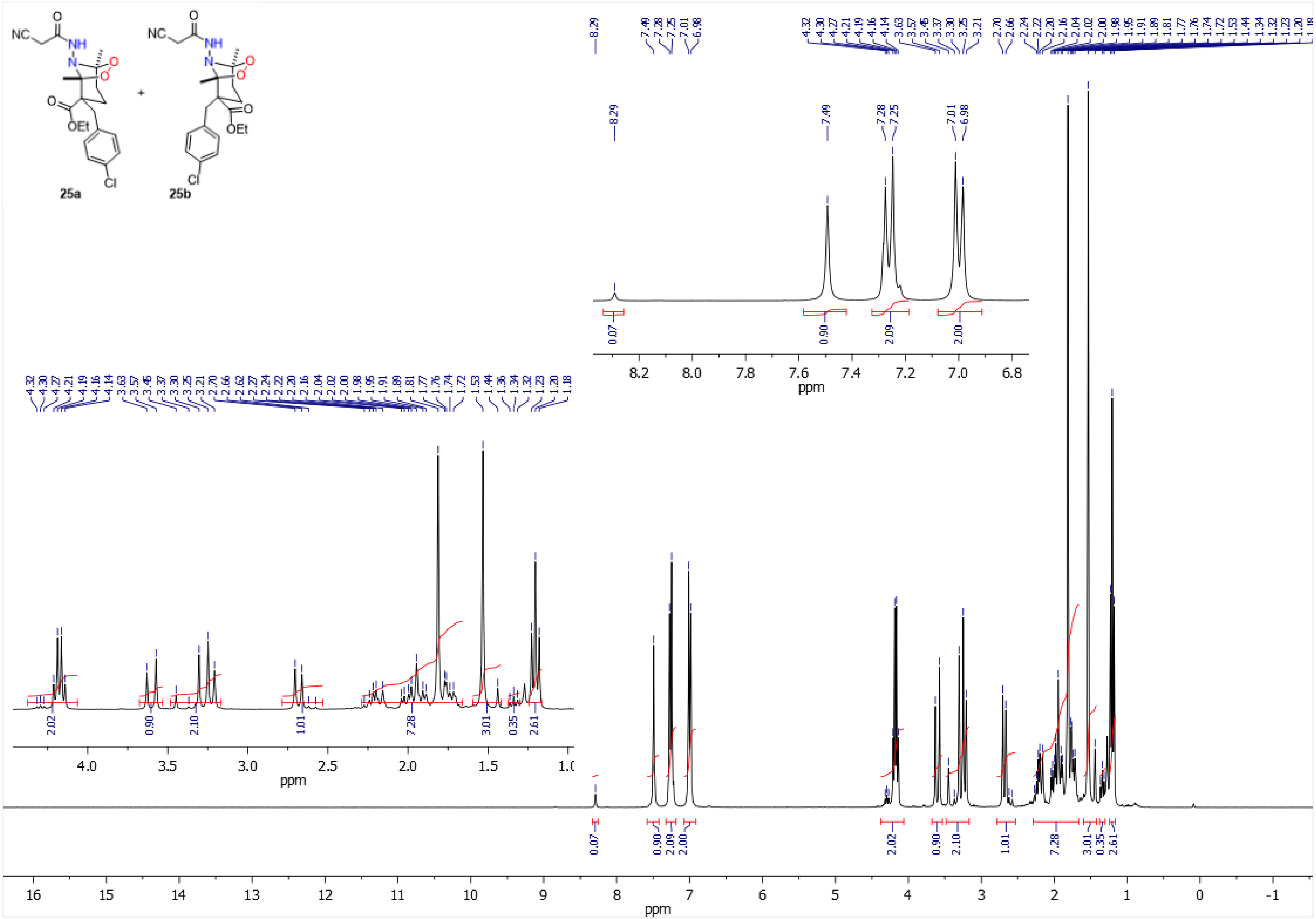

^{13}C NMR (75.48 MHz, $CDCl_3$). Ethyl 2-(4-chlorobenzyl)-8-(2-cyanoacetamido)-1,5-dimethyl-6,7-dioxa-8-azabicyclo[3.2.1]octane-2-carboxylate, 25a + 25b

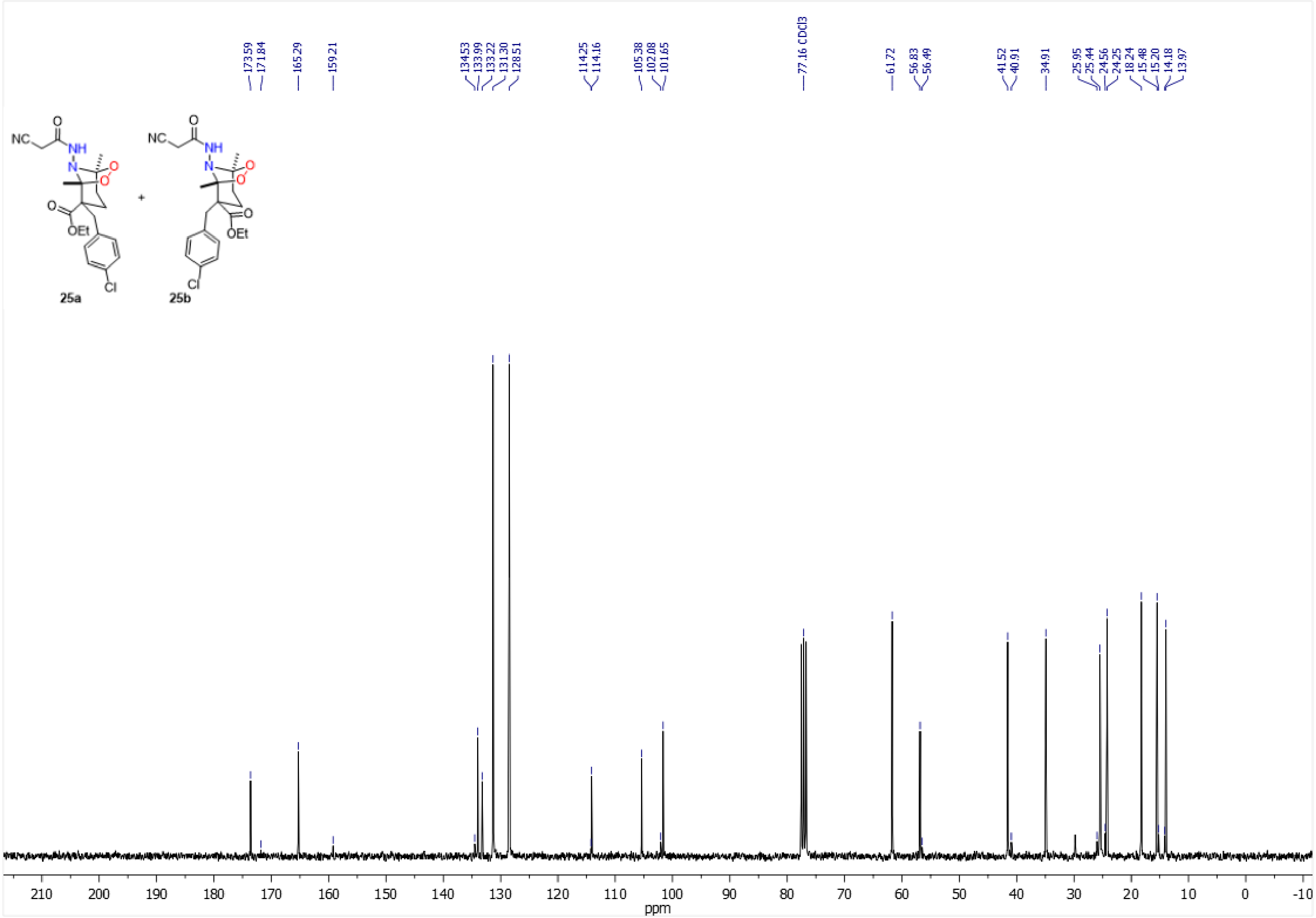

^{1}H NMR (300.13 MHz, $CDCl_3$). Ethyl 8-(2-cyanoacetamido)-2-(4-fluorobenzyl)-1,5-dimethyl-6,7-dioxa-8-azabicyclo[3.2.1]octane-2-carboxylate, 26a + 26b

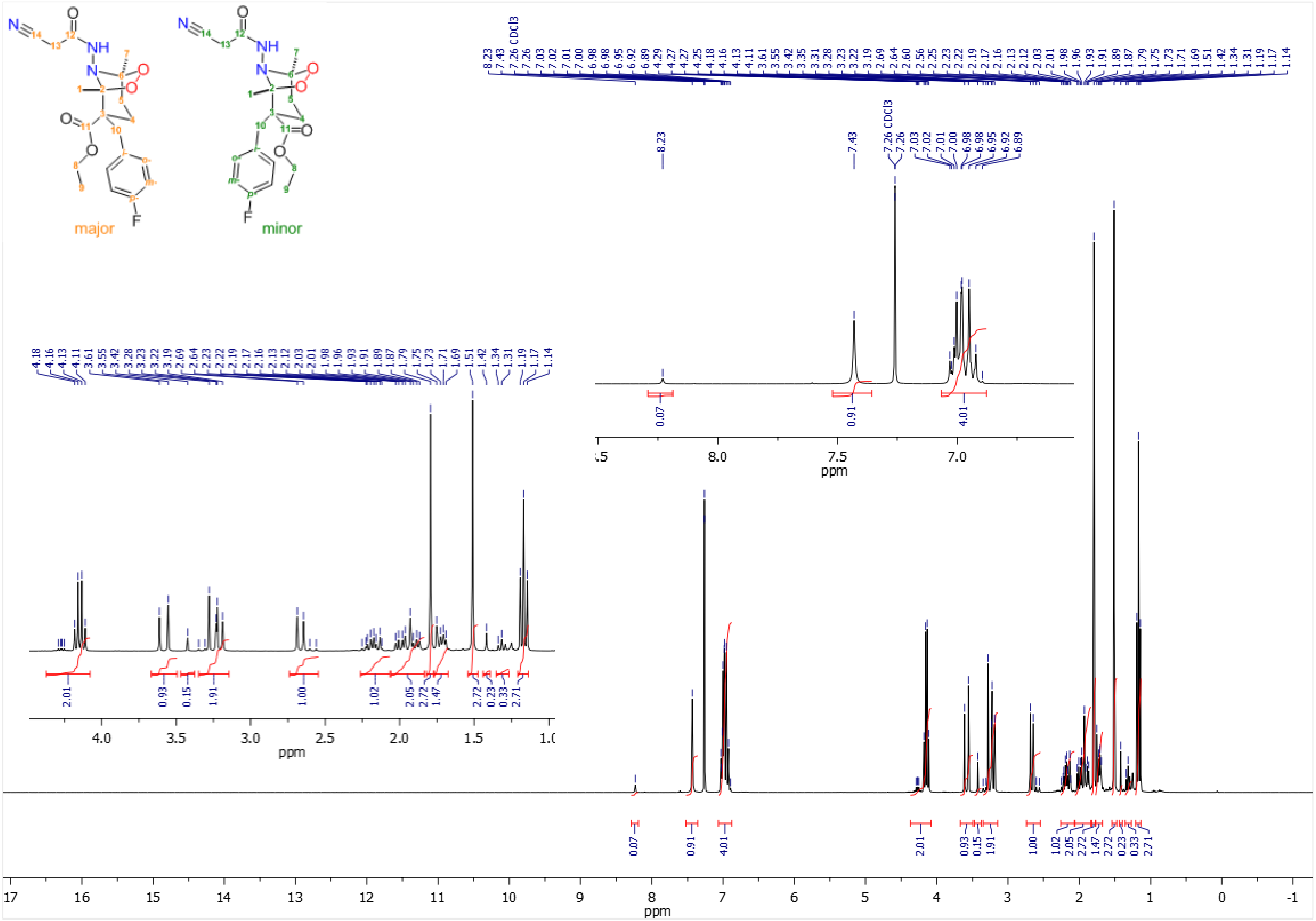

^{1}H NMR (300.13 MHz, CDCl3). Ethyl 8-(2-cyanoacetamido)-2-(4-fluorobenzyl)-1,5-dimethyl-6,7-dioxa-8-azabicyclo[3.2.1]octane-2-carboxylate, 26a + 26b

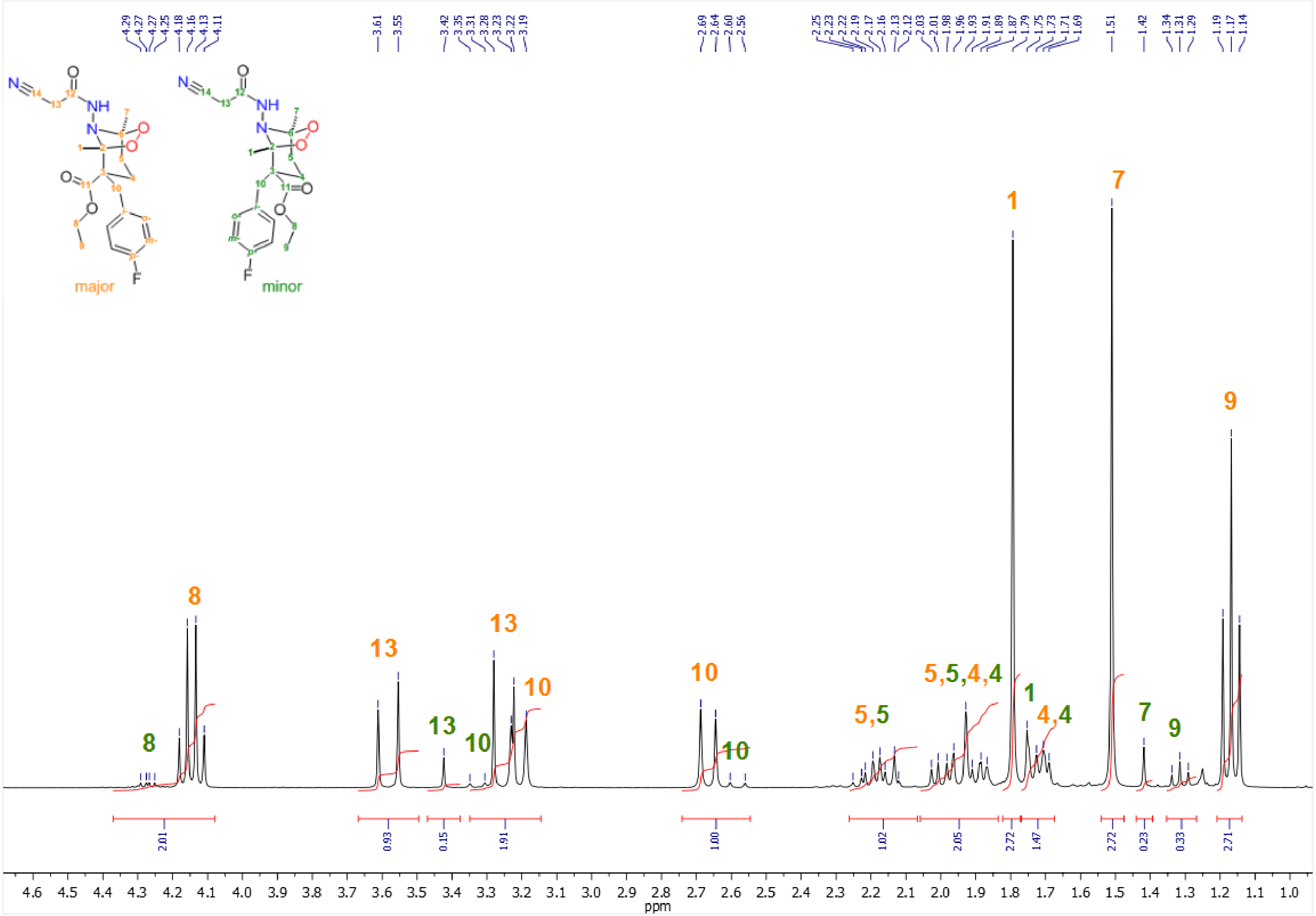

^{1}H NMR (300.13 MHz, CDCl$_3$). Ethyl 8-(2-cyanoacetamido)-2-(4-fluorobenzyl)-1,5-dimethyl-6,7-dioxa-8-azabicyclo[3.2.1]octane-2-carboxylate, 26a + 26b

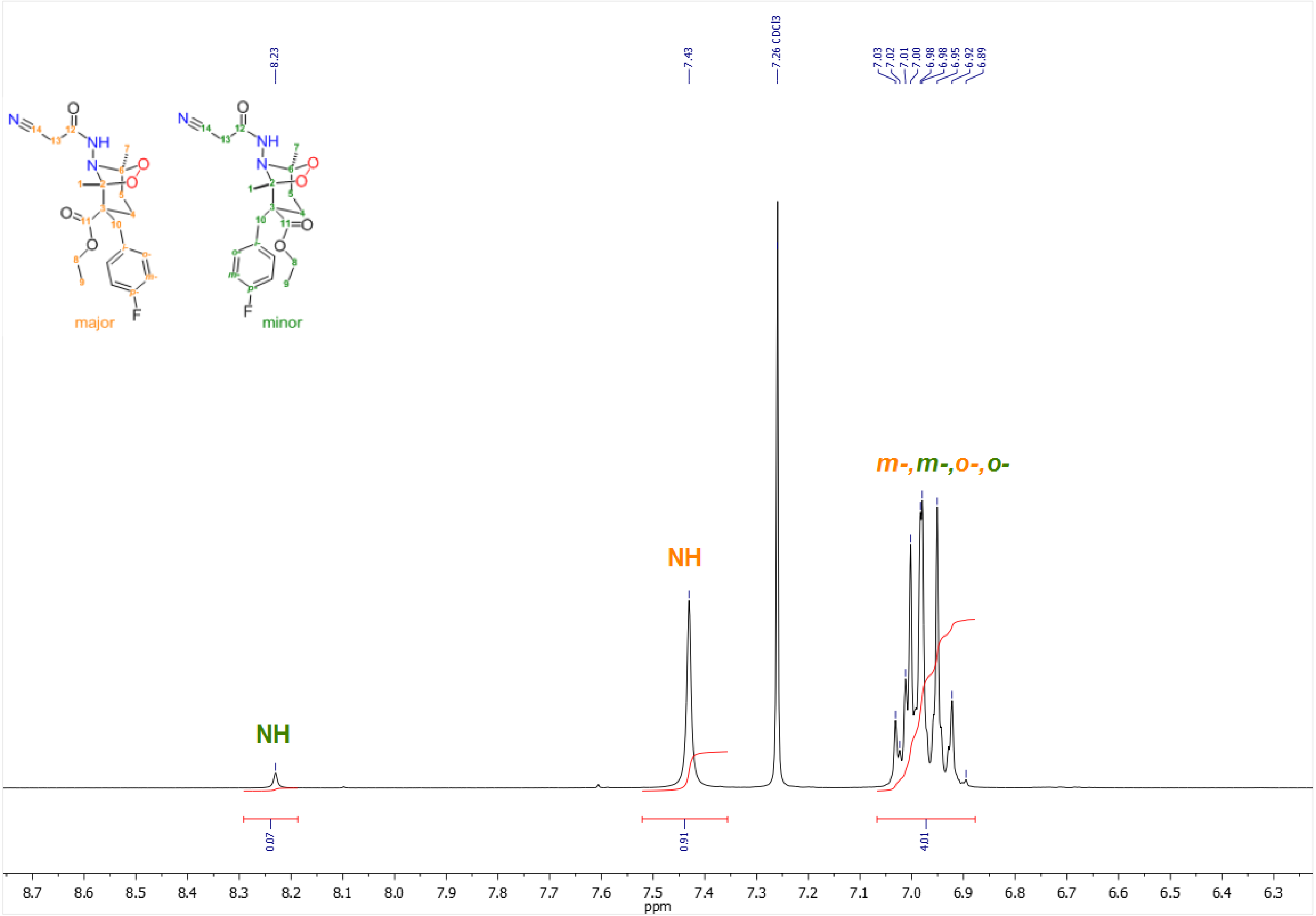

^{13}C NMR (75.48 MHz, $CDCl_3$). Ethyl 8-(2-cyanoacetamido)-2-(4-fluorobenzyl)-1,5-dimethyl-6,7-dioxa-8-azabicyclo[3.2.1]octane-2-carboxylate, 26a + 26b

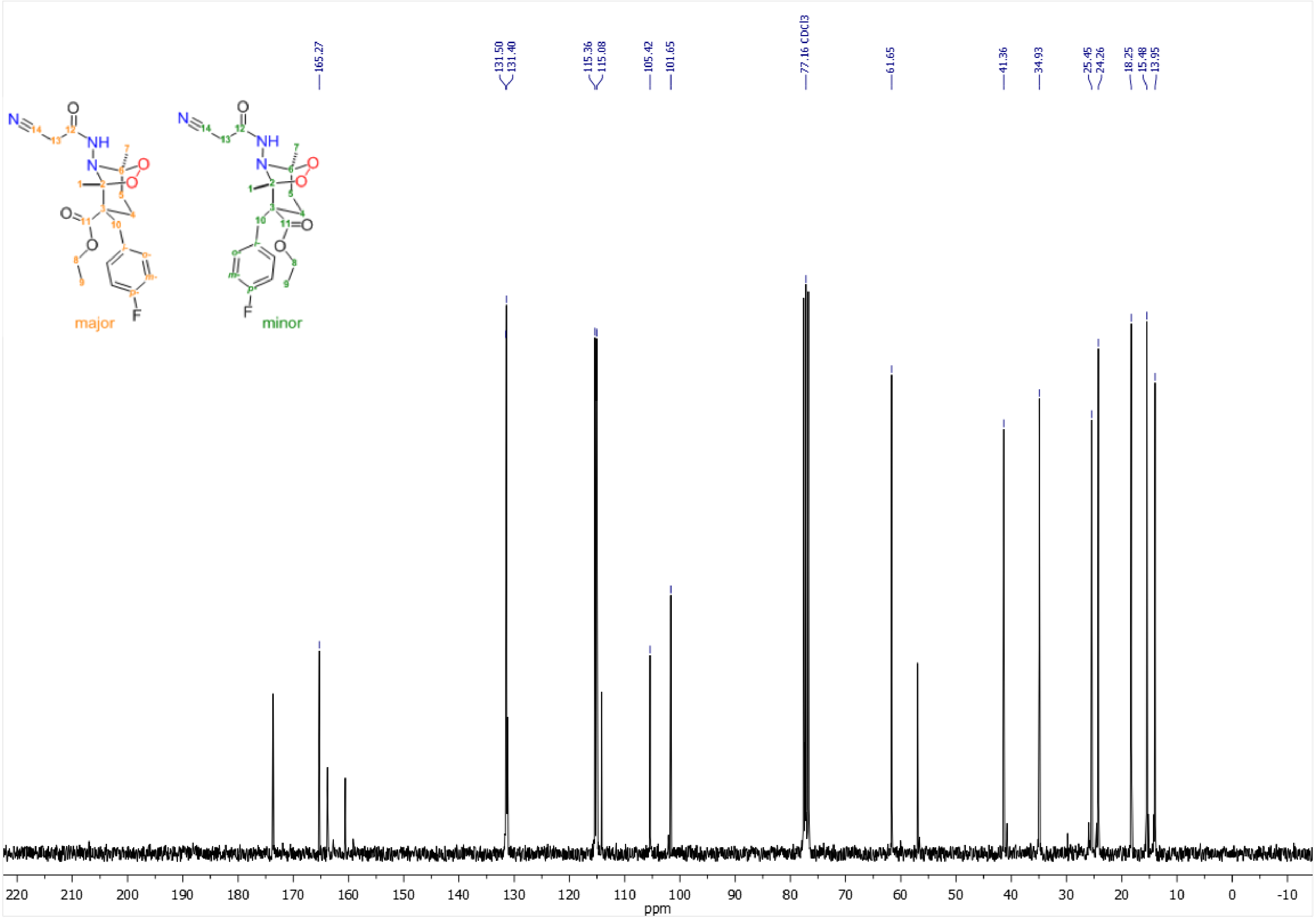

^{13}C NMR (75.48 MHz, CDCl3). Ethyl 8-(2-cyanoacetamido)-2-(4-fluorobenzyl)-1,5-dimethyl-6,7-dioxa-8-azabicyclo[3.2.1]octane-2-carboxylate, 26a + 26b

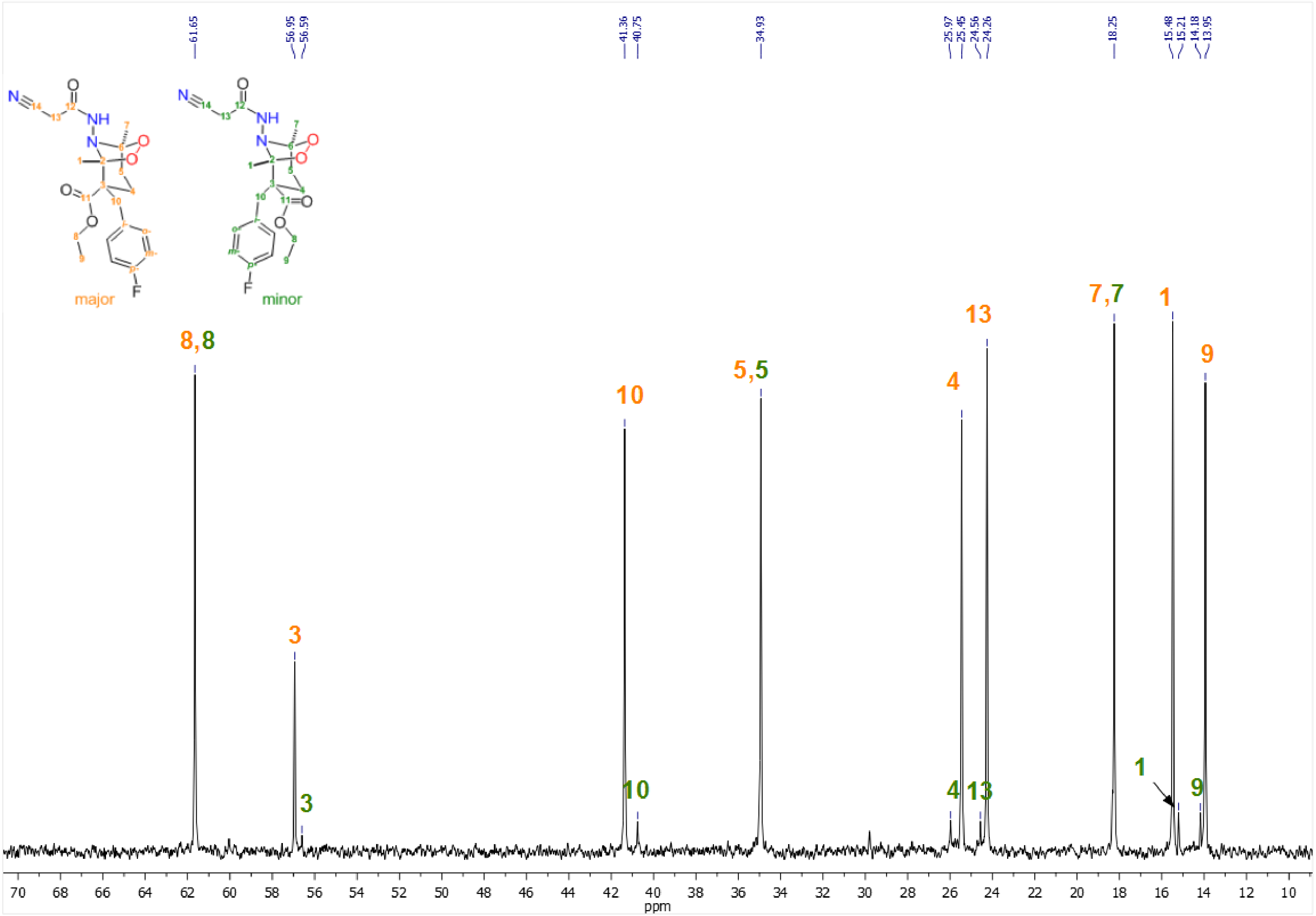

^{13}C NMR (75.48 MHz, CDCl3). Ethyl 8-(2-cyanoacetamido)-2-(4-fluorobenzyl)-1,5-dimethyl-6,7-dioxa-8-azabicyclo[3.2.1]octane-2-carboxylate, 26a + 26b

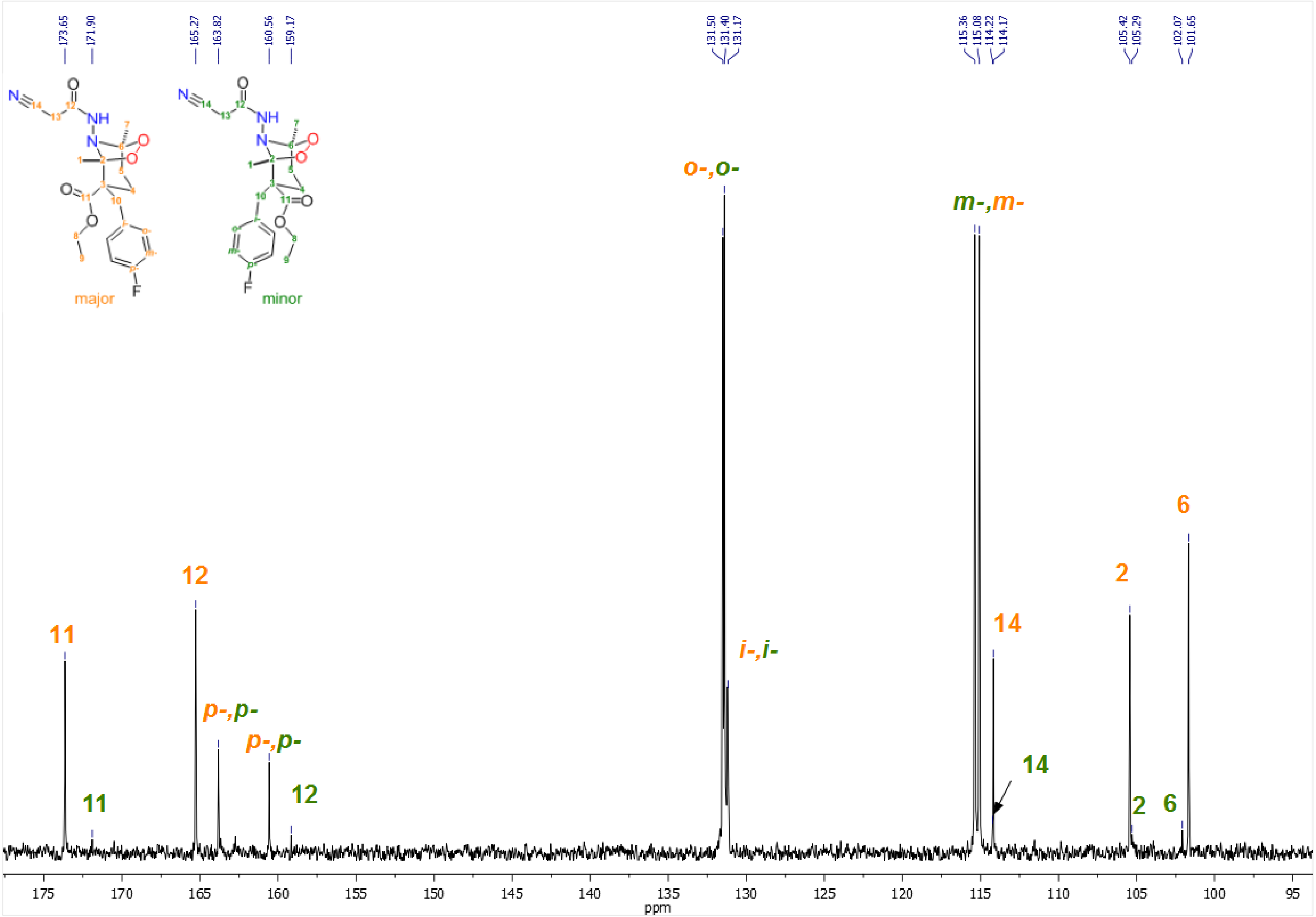

^{13}C NMR (75.48 MHz, CDCl₃). Ethyl 8-(2-cyanoacetamido)-2-(4-fluorobenzyl)-1,5-dimethyl-6,7-dioxa-8-azabicyclo[3.2.1]octane-2-carboxylate, 26a + 26b

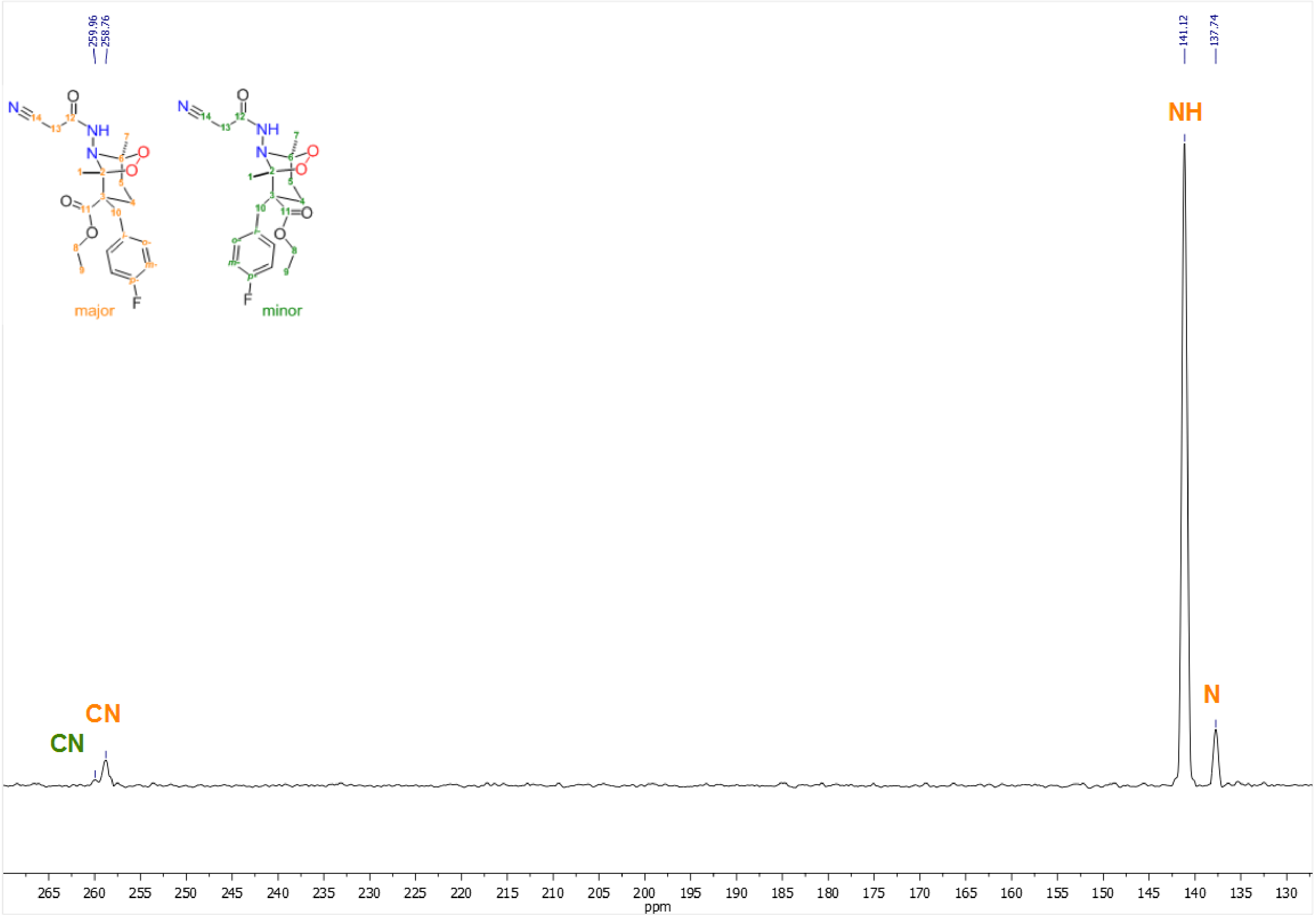

^{15}N (40.56 MHz, CDCl3). Ethyl 8-(2-cyanoacetamido)-2-(4-fluorobenzyl)-1,5-dimethyl-6,7-dioxa-8-azabicyclo[3.2.1]octane-2-carboxylate, 26a + 26b

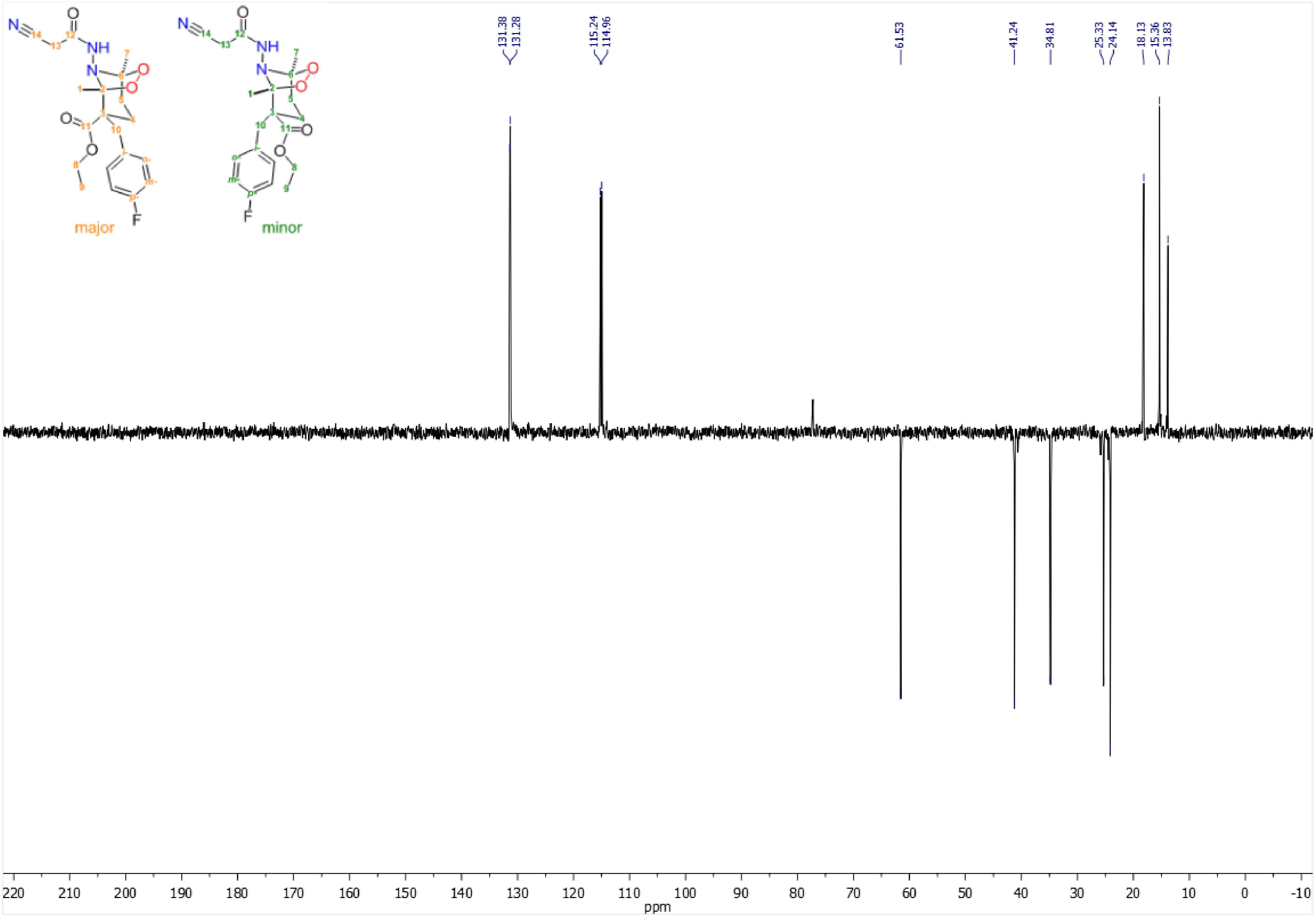

^{13}C NMR (75.48 MHz, CDCl$_3$). Ethyl 8-(2-cyanoacetamido)-2-(4-fluorobenzyl)-1,5-dimethyl-6,7-dioxa-8-azabicyclo[3.2.1]octane-2-carboxylate, 26a + 26b

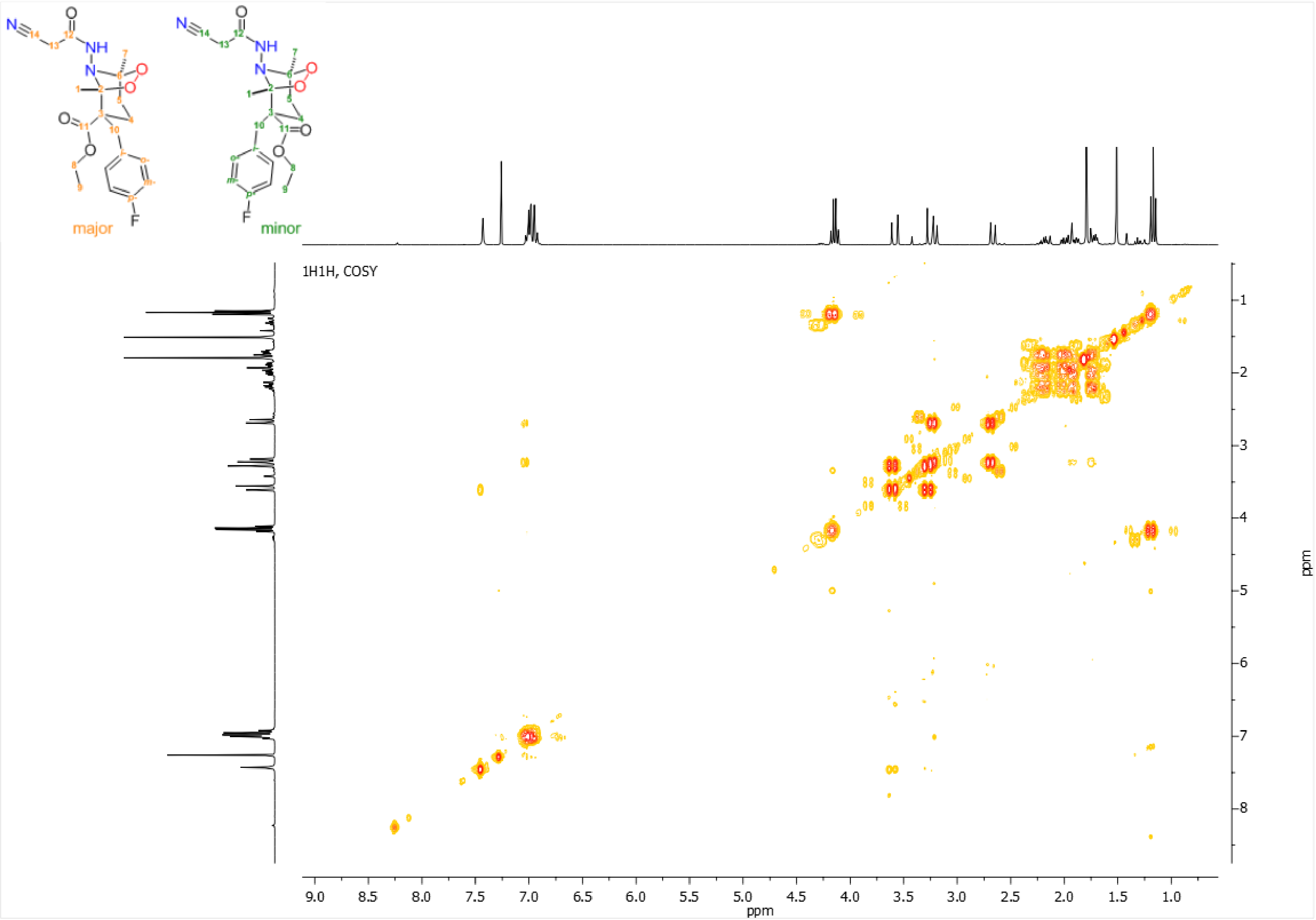

Ethyl 8-(2-cyanoacetamido)-2-(4-fluorobenzyl)-1,5-dimethyl-6,7-dioxa-8-azabicyclo[3.2.1]octane-2-carboxylate, 26a + 26b

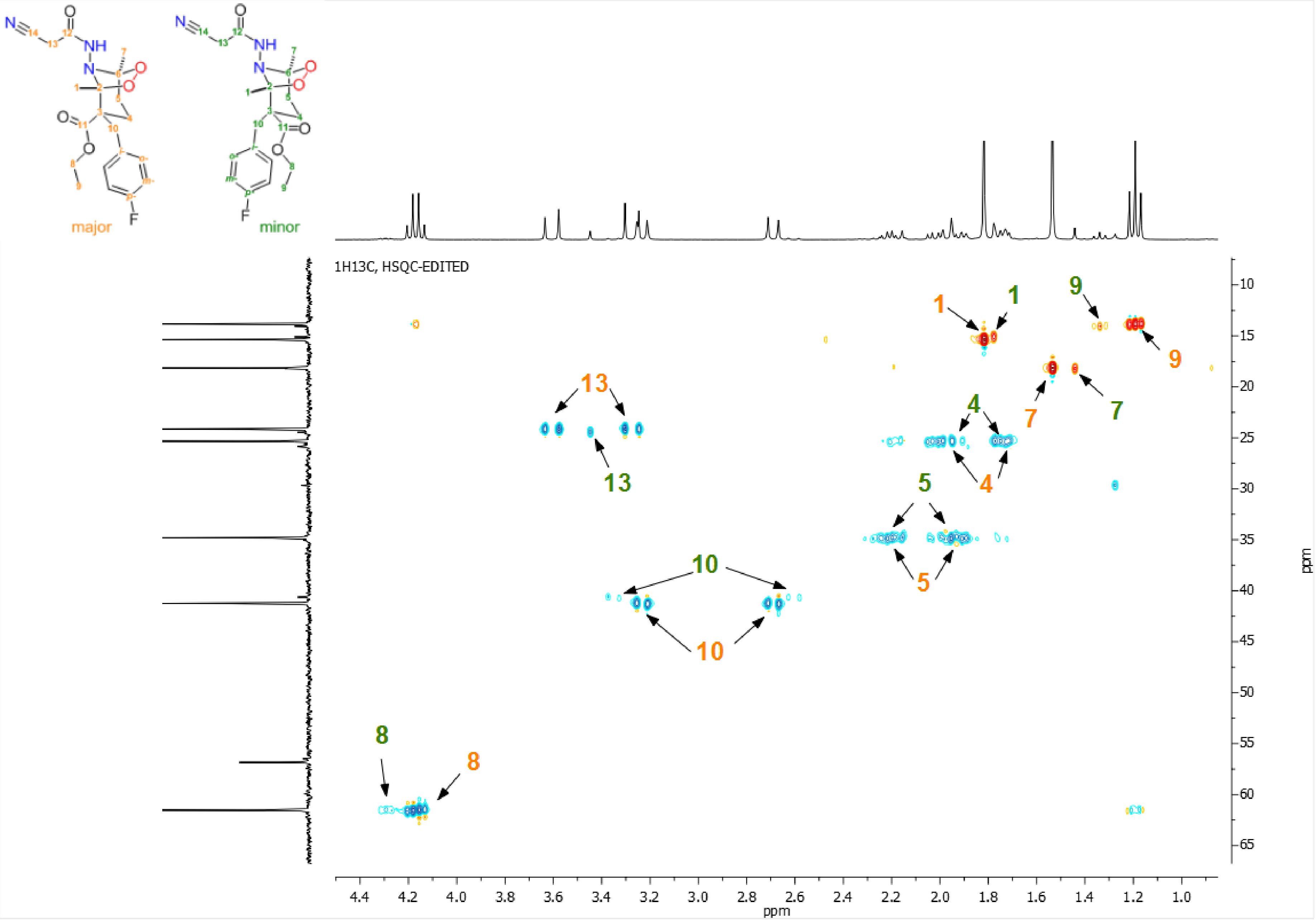

Ethyl 8-(2-cyanoacetamido)-2-(4-fluorobenzyl)-1,5-dimethyl-6,7-dioxa-8-azabicyclo[3.2.1]octane-2-carboxylate, 26a + 26b

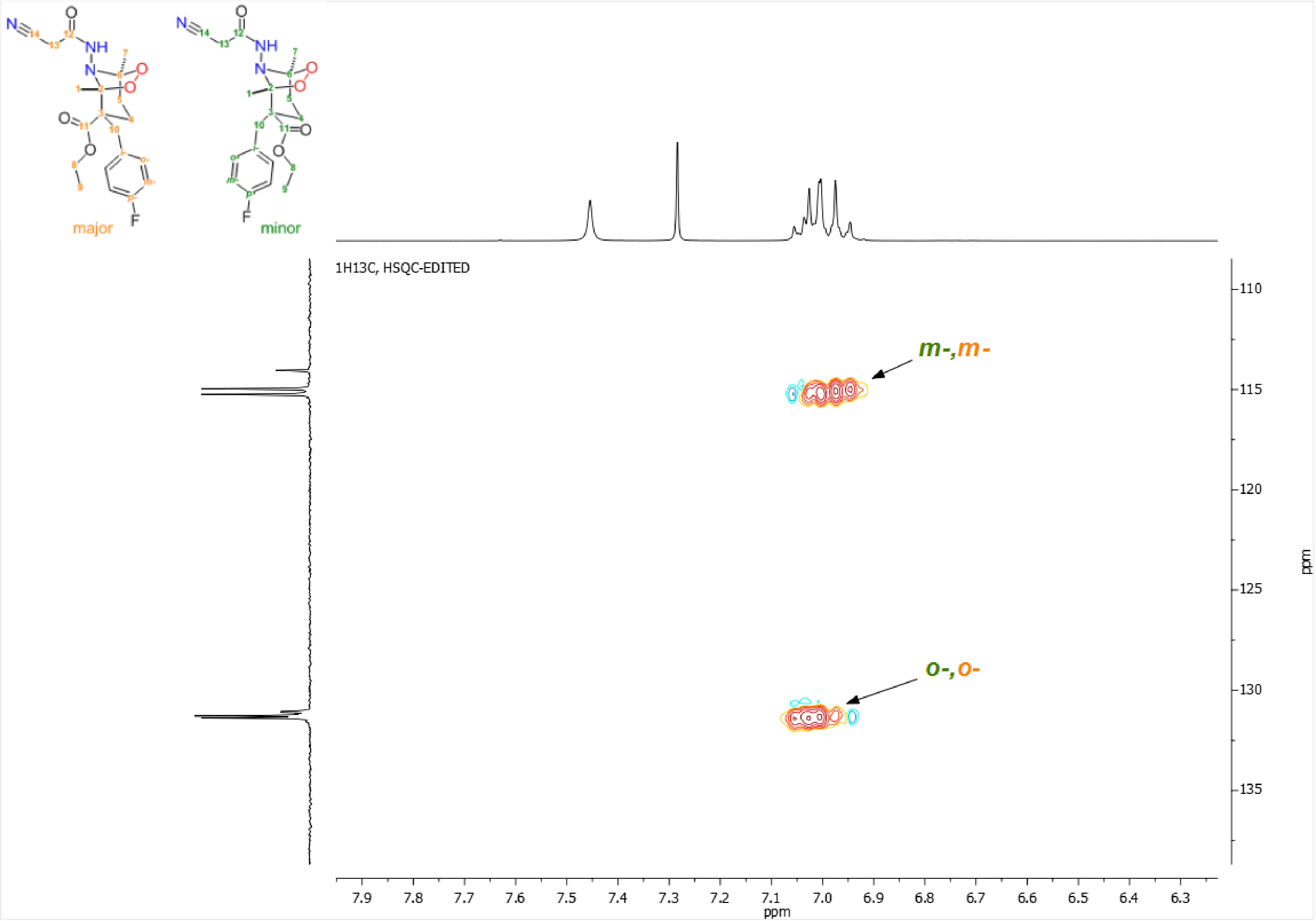

Ethyl 8-(2-cyanoacetamido)-2-(4-fluorobenzyl)-1,5-dimethyl-6,7-dioxa-8-azabicyclo[3.2.1]octane-2-carboxylate, 26a + 26b

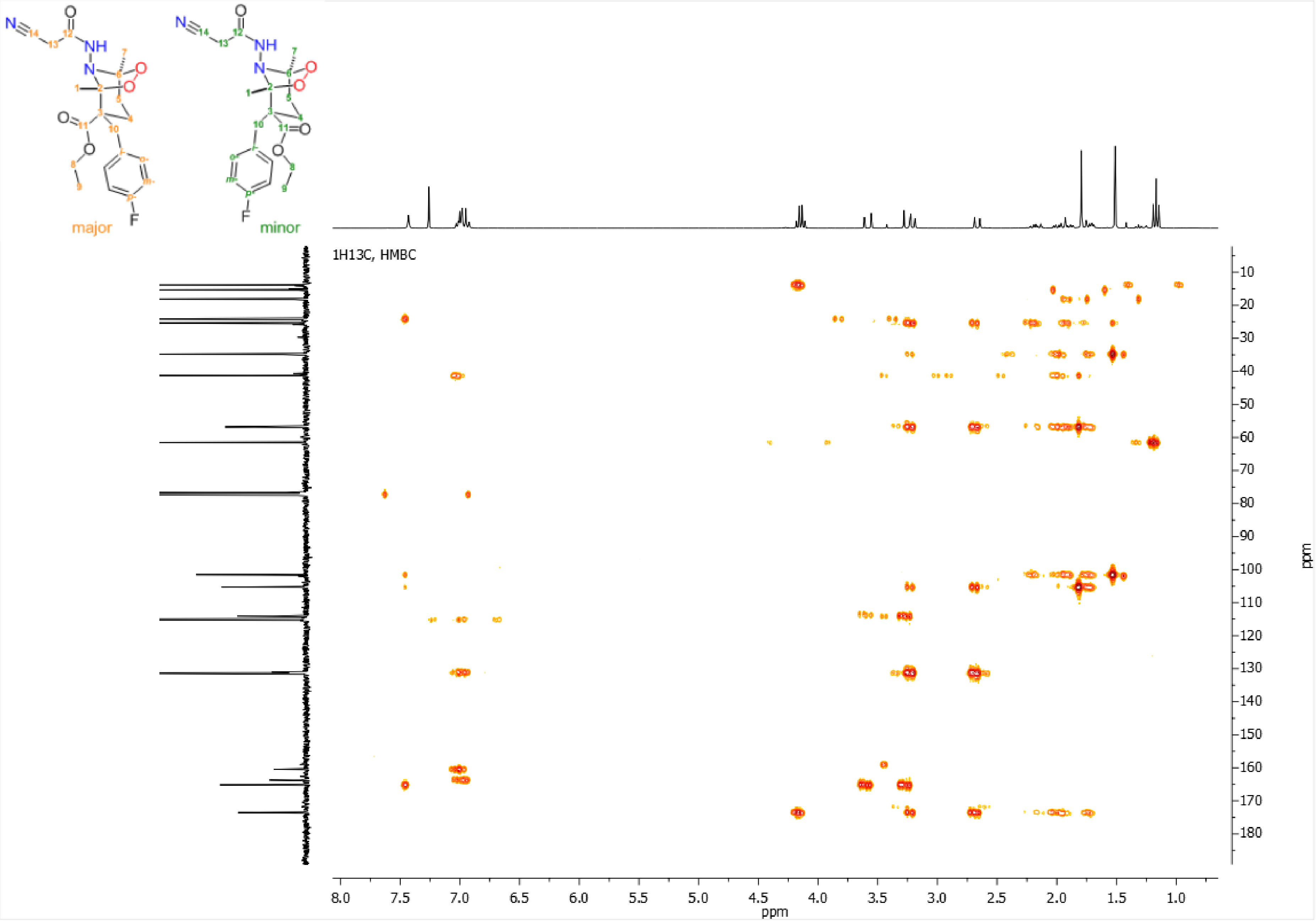

Ethyl 8-(2-cyanoacetamido)-2-(4-fluorobenzyl)-1,5-dimethyl-6,7-dioxa-8-azabicyclo[3.2.1]octane-2-carboxylate, 26a + 26b

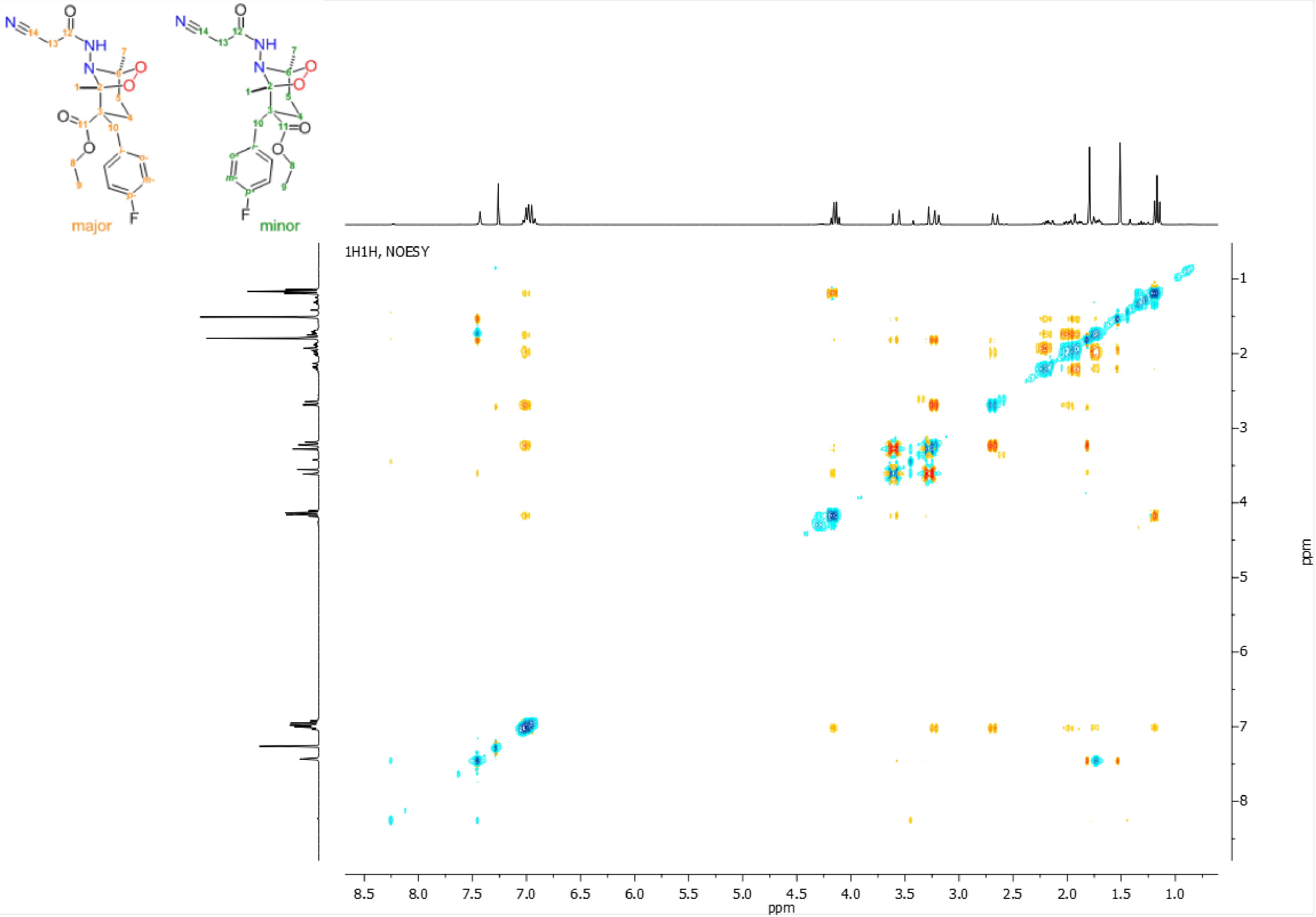

Ethyl 8-(2-cyanoacetamido)-2-(4-fluorobenzyl)-1,5-dimethyl-6,7-dioxa-8-azabicyclo[3.2.1]octane-2-carboxylate, 26a + 26b

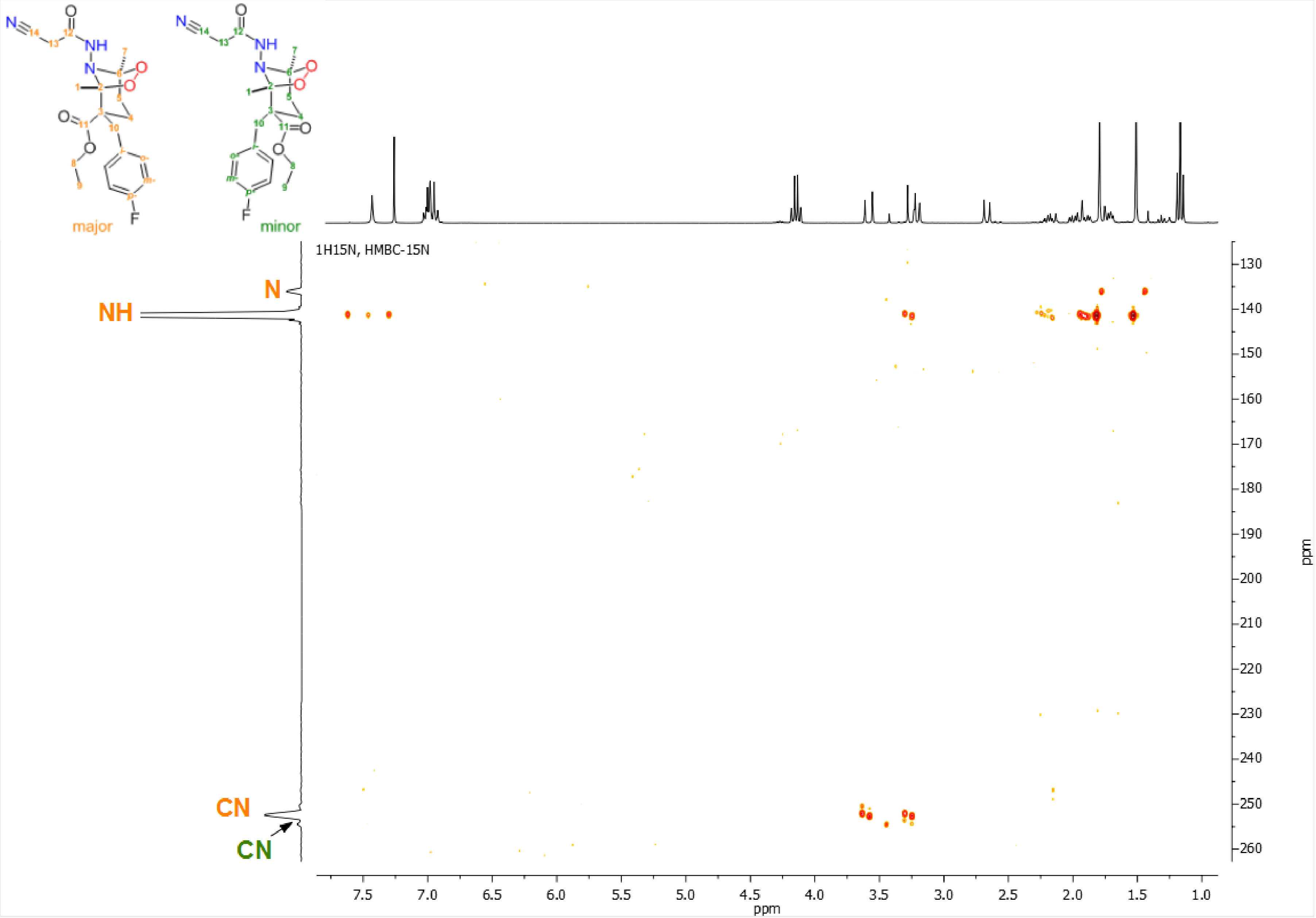

Ethyl 8-(2-cyanoacetamido)-2-(4-fluorobenzyl)-1,5-dimethyl-6,7-dioxa-8-azabicyclo[3.2.1]octane-2-carboxylate, 26a + 26b

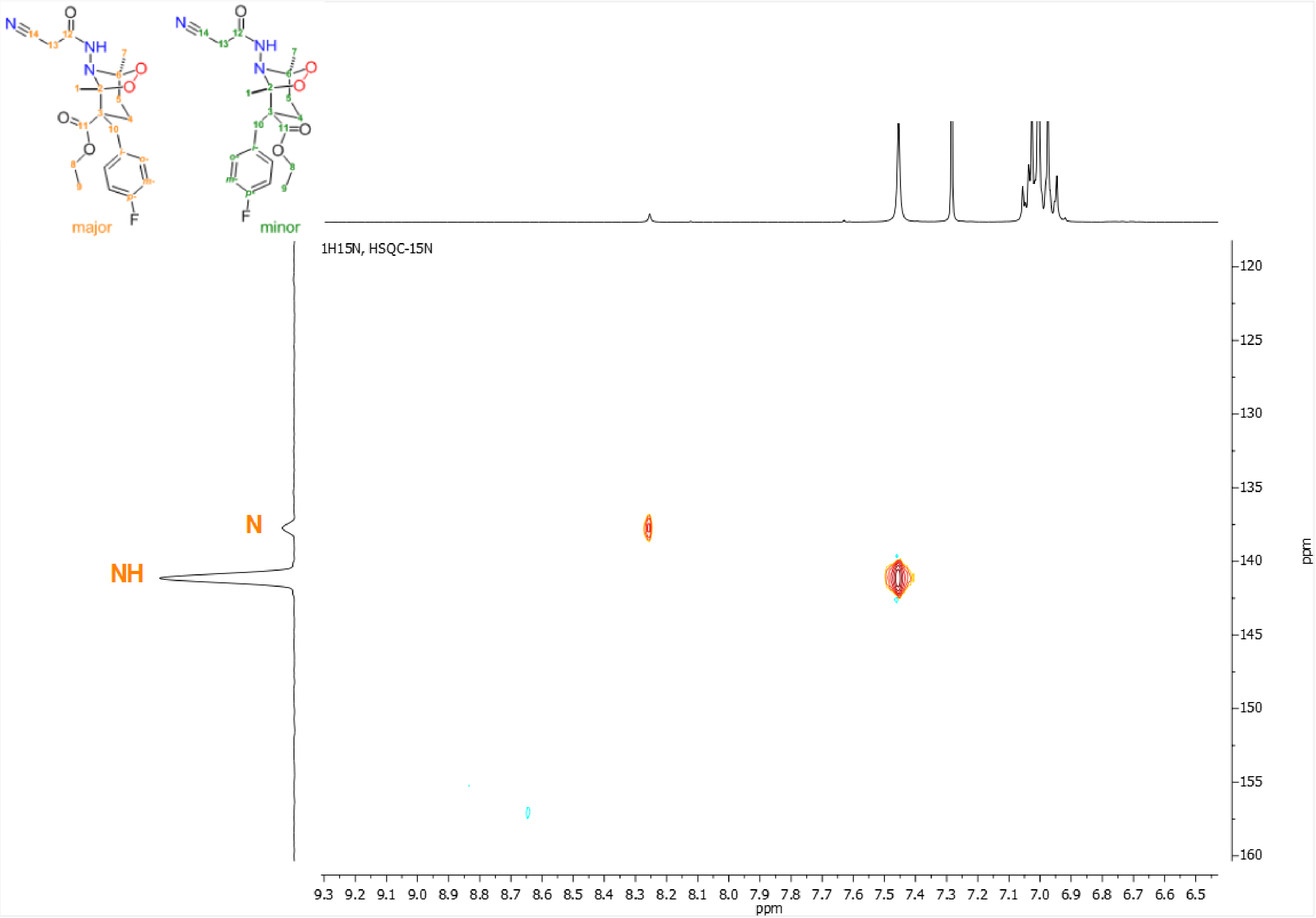

^{1}H NMR (300.13 MHz, $CDCl_3$). Ethyl 2-benzyl-8-(2-cyanoacetamido)-1,5-dimethyl-6,7-dioxa-8-azabicyclo[3.2.1]octane-2-carboxylate, 27a + 27b

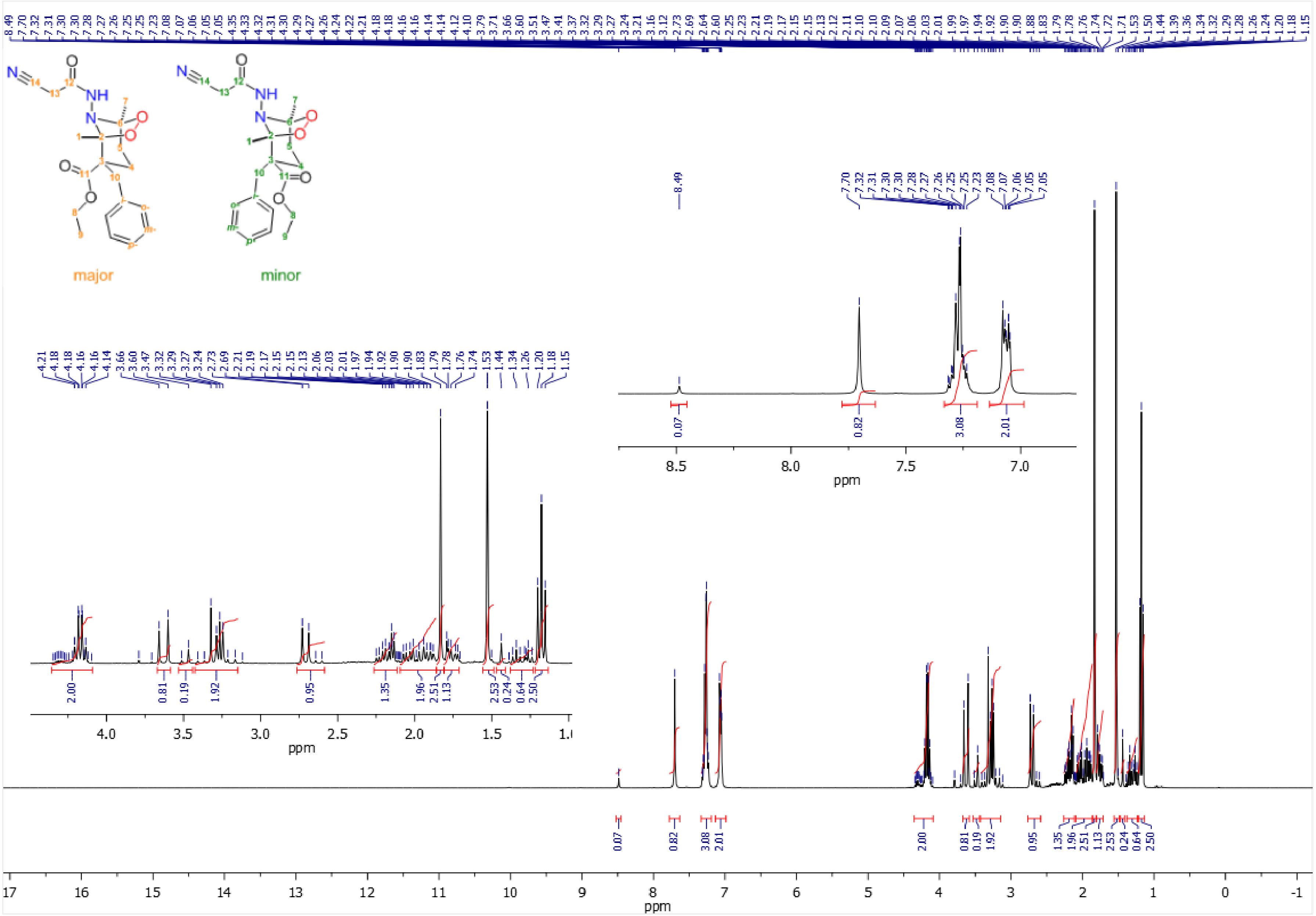

^1H NMR (300.13 MHz, $CDCl_3$). Ethyl 2-benzyl-8-(2-cyanoacetamido)-1,5-dimethyl-6,7-dioxa-8-azabicyclo[3.2.1]octane-2-carboxylate, 27a + 27b

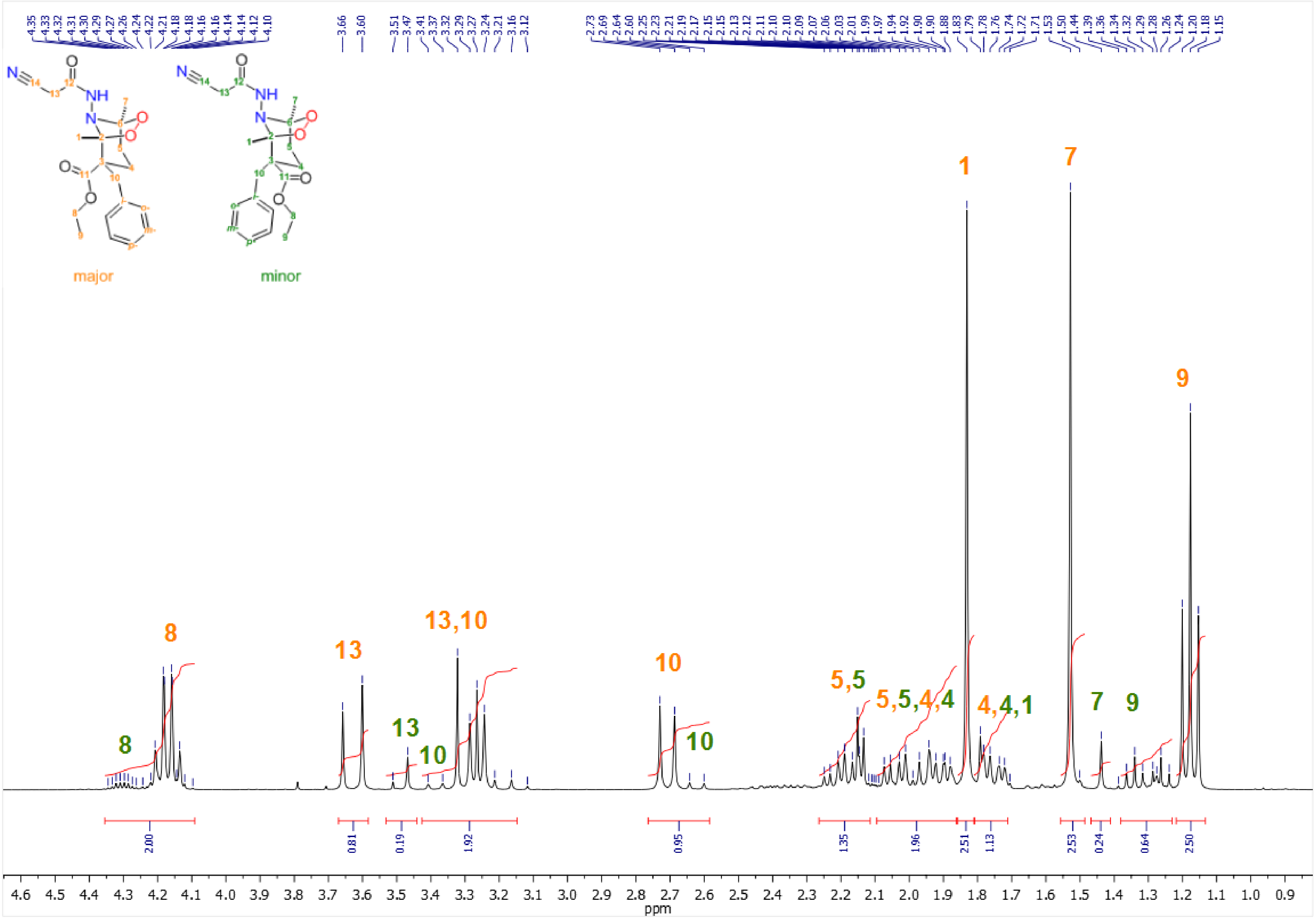

^{1}H NMR (300.13 MHz, $CDCl_3$). Ethyl 2-benzyl-8-(2-cyanoacetamido)-1,5-dimethyl-6,7-dioxa-8-azabicyclo[3.2.1]octane-2-carboxylate, 27a + 27b

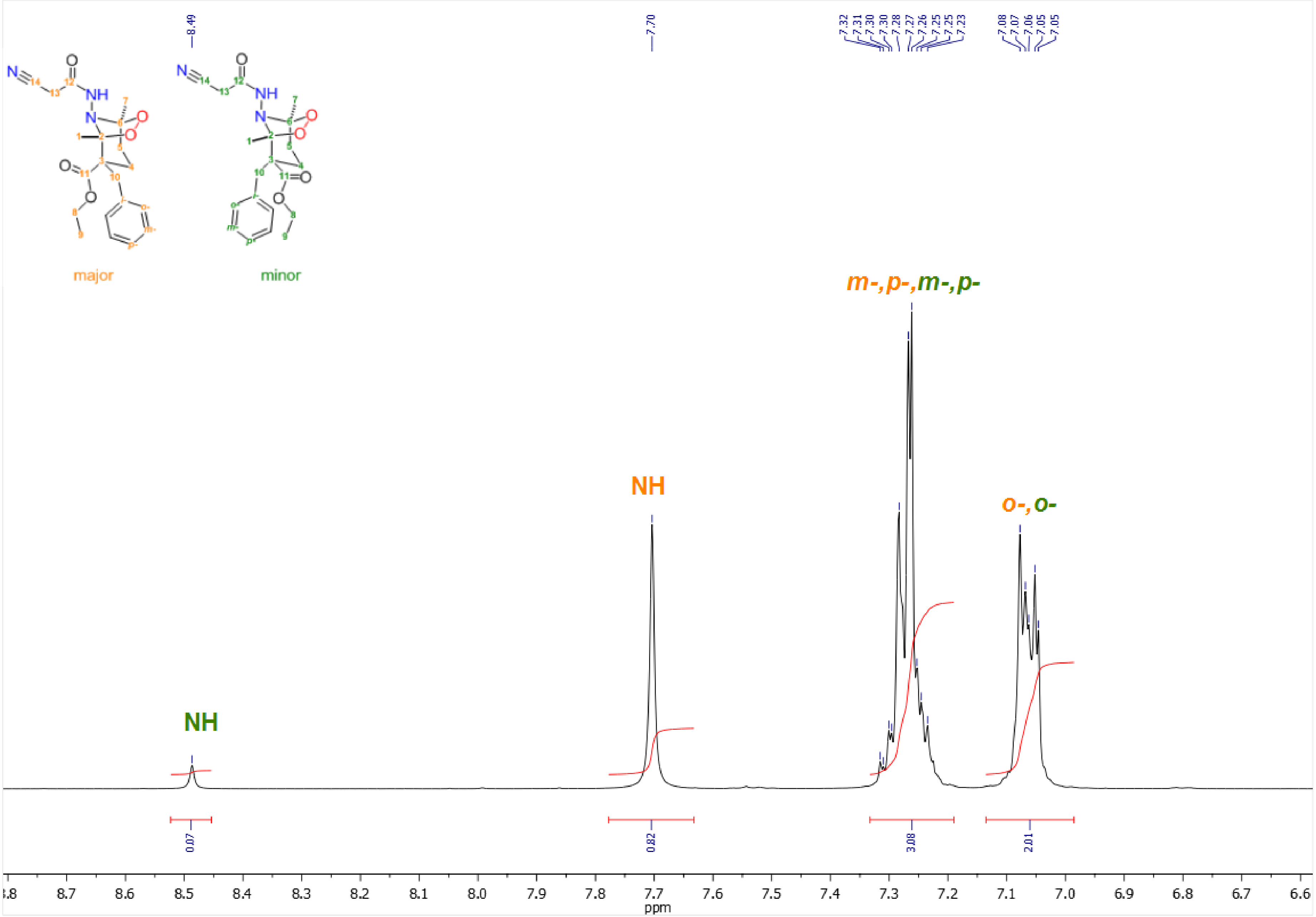

^{13}C NMR (75.48 MHz, $CDCl_3$). Ethyl 2-benzyl-8-(2-cyanoacetamido)-1,5-dimethyl-6,7-dioxa-8-azabicyclo[3.2.1]octane-2-carboxylate, 27a + 27b

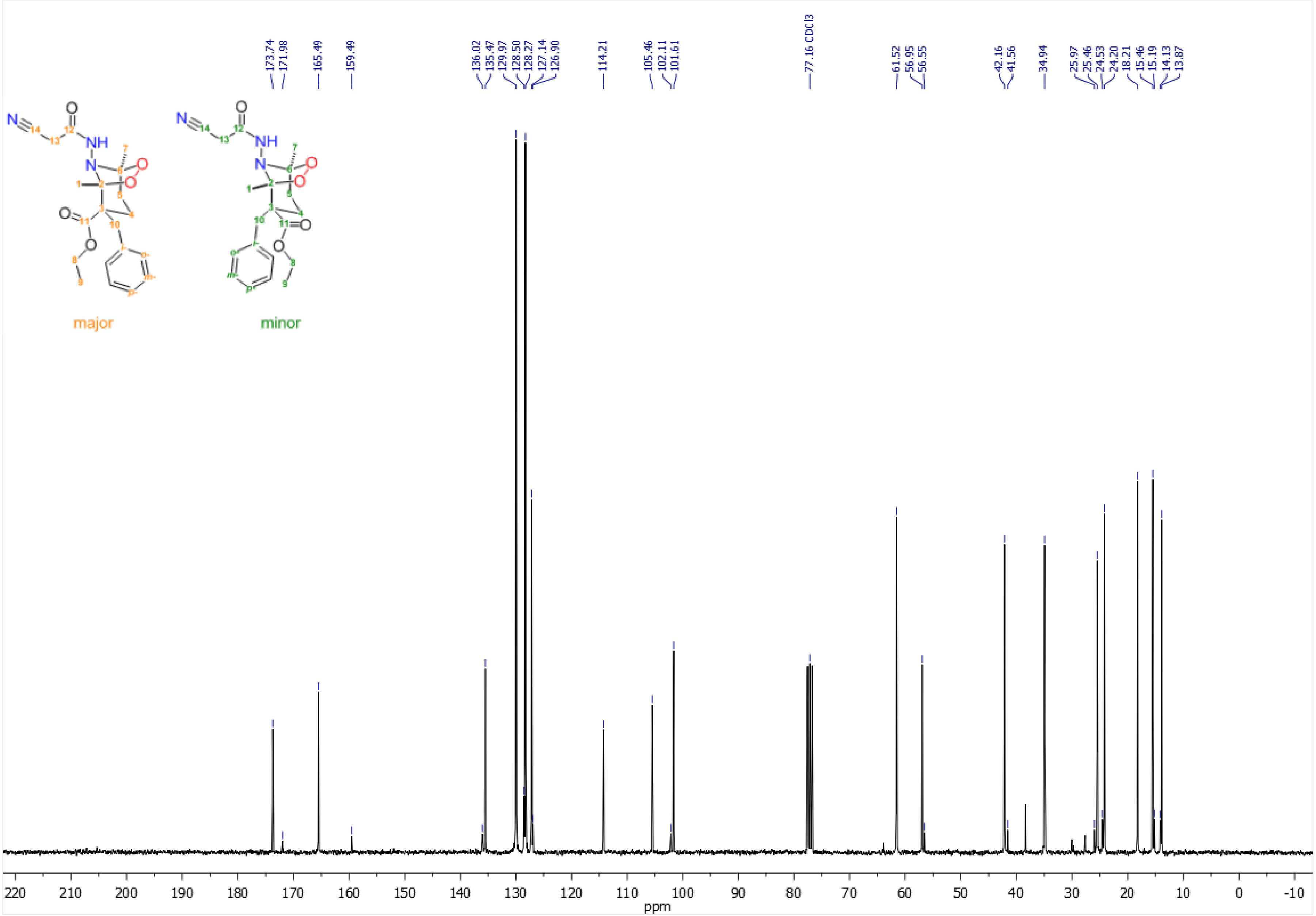

^{13}C NMR (75.48 MHz, $CDCl_3$). Ethyl 2-benzyl-8-(2-cyanoacetamido)-1,5-dimethyl-6,7-dioxa-8-azabicyclo[3.2.1]octane-2-carboxylate, 27a + 27b

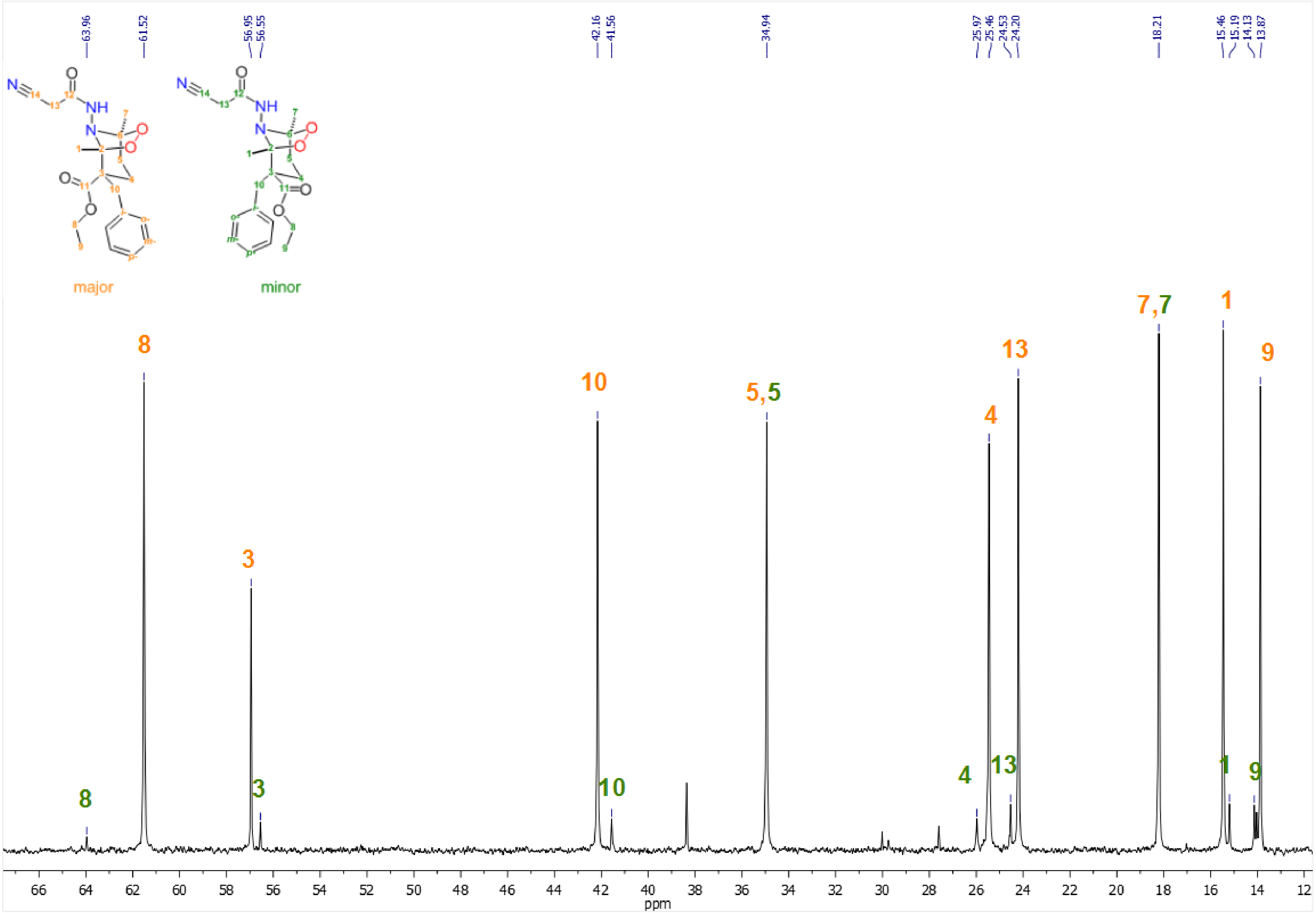

^{13}C NMR (75.48 MHz, $CDCl_3$). Ethyl 2-benzyl-8-(2-cyanoacetamido)-1,5-dimethyl-6,7-dioxa-8-azabicyclo[3.2.1]octane-2-carboxylate, 27a + 27b

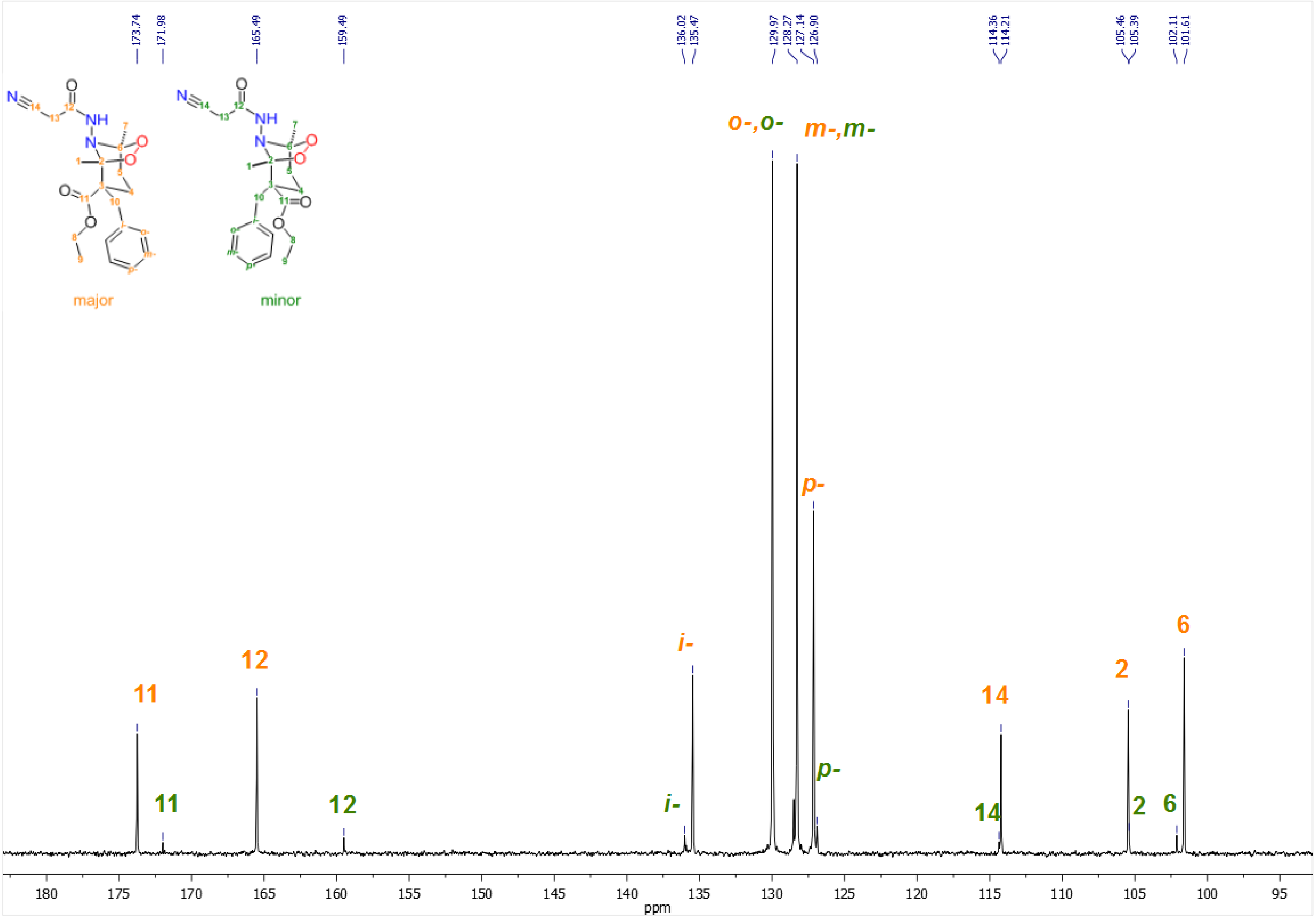

^{15}N (40.56 MHz, $CDCl_3$). Ethyl 2-benzyl-8-(2-cyanoacetamido)-1,5-dimethyl-6,7-dioxa-8-azabicyclo[3.2.1]octane-2-carboxylate, 27a + 27b

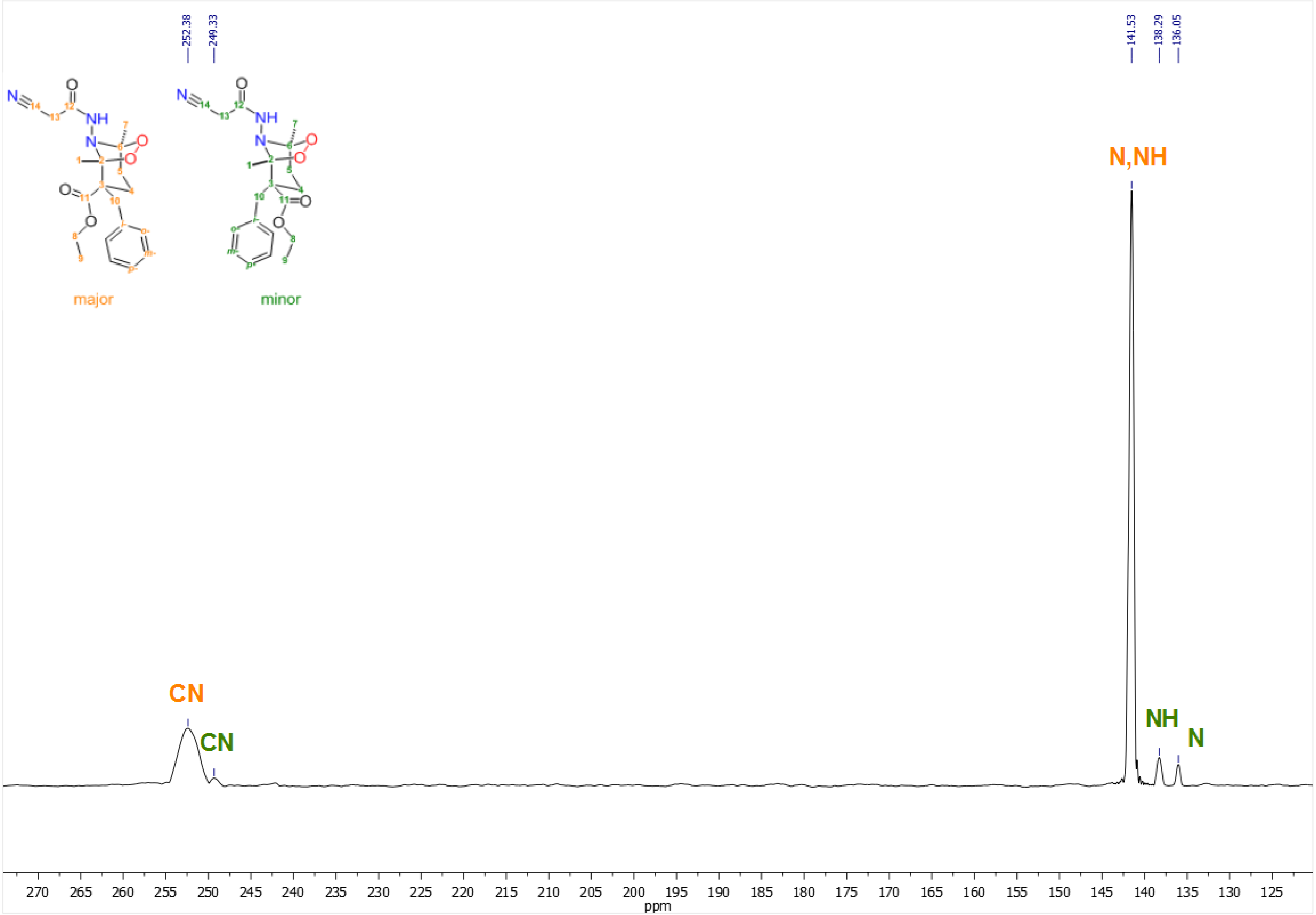

^{15}N (40.56 MHz, CDCl$_3$). Ethyl 2-benzyl-8-(2-cyanoacetamido)-1,5-dimethyl-6,7-dioxa-8-azabicyclo[3.2.1]octane-2-carboxylate, 27a + 27b

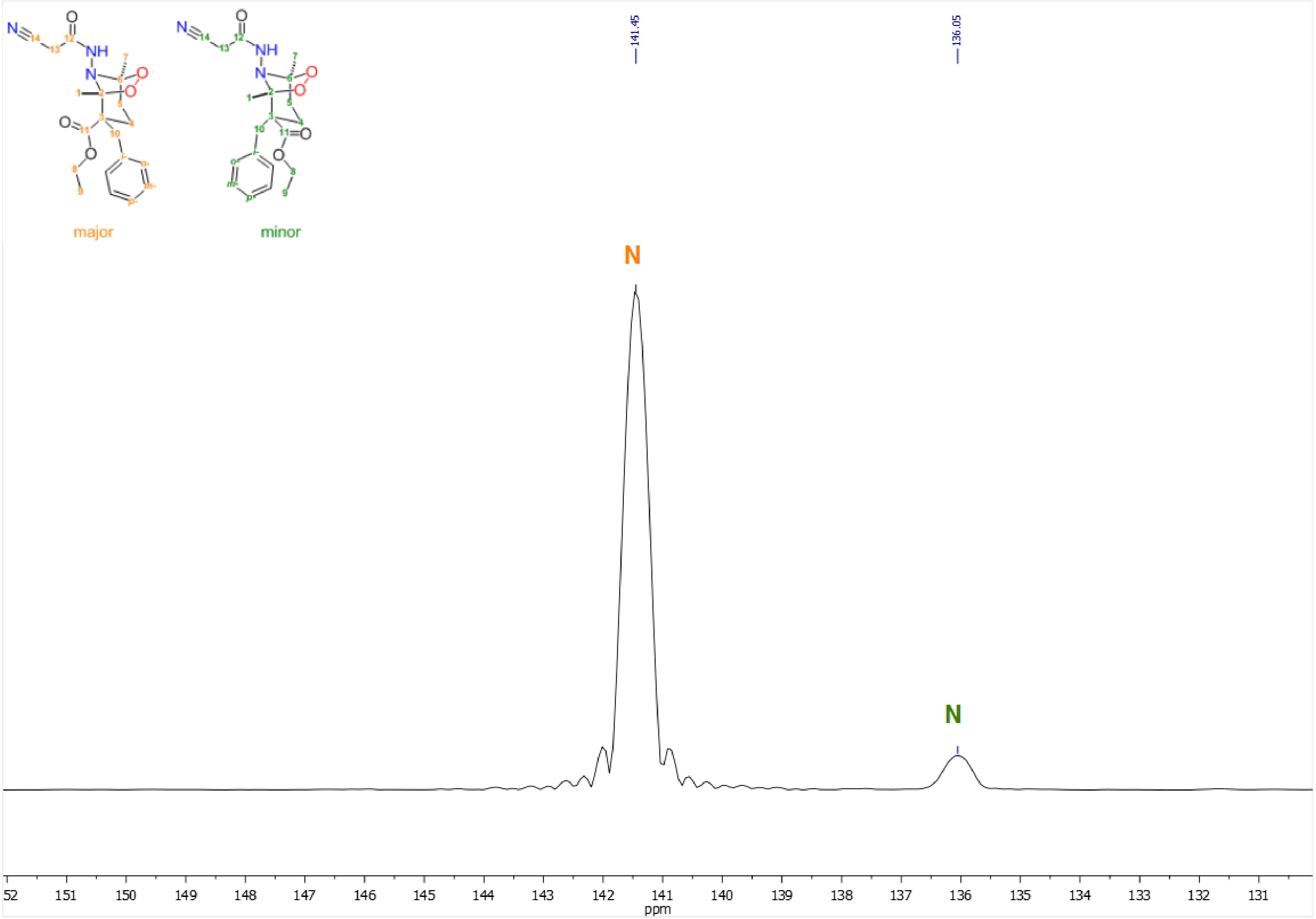

^{15}N (40.56 MHz, $CDCl_3$). Ethyl 2-benzyl-8-(2-cyanoacetamido)-1,5-dimethyl-6,7-dioxa-8-azabicyclo[3.2.1]octane-2-carboxylate, 27a + 27b

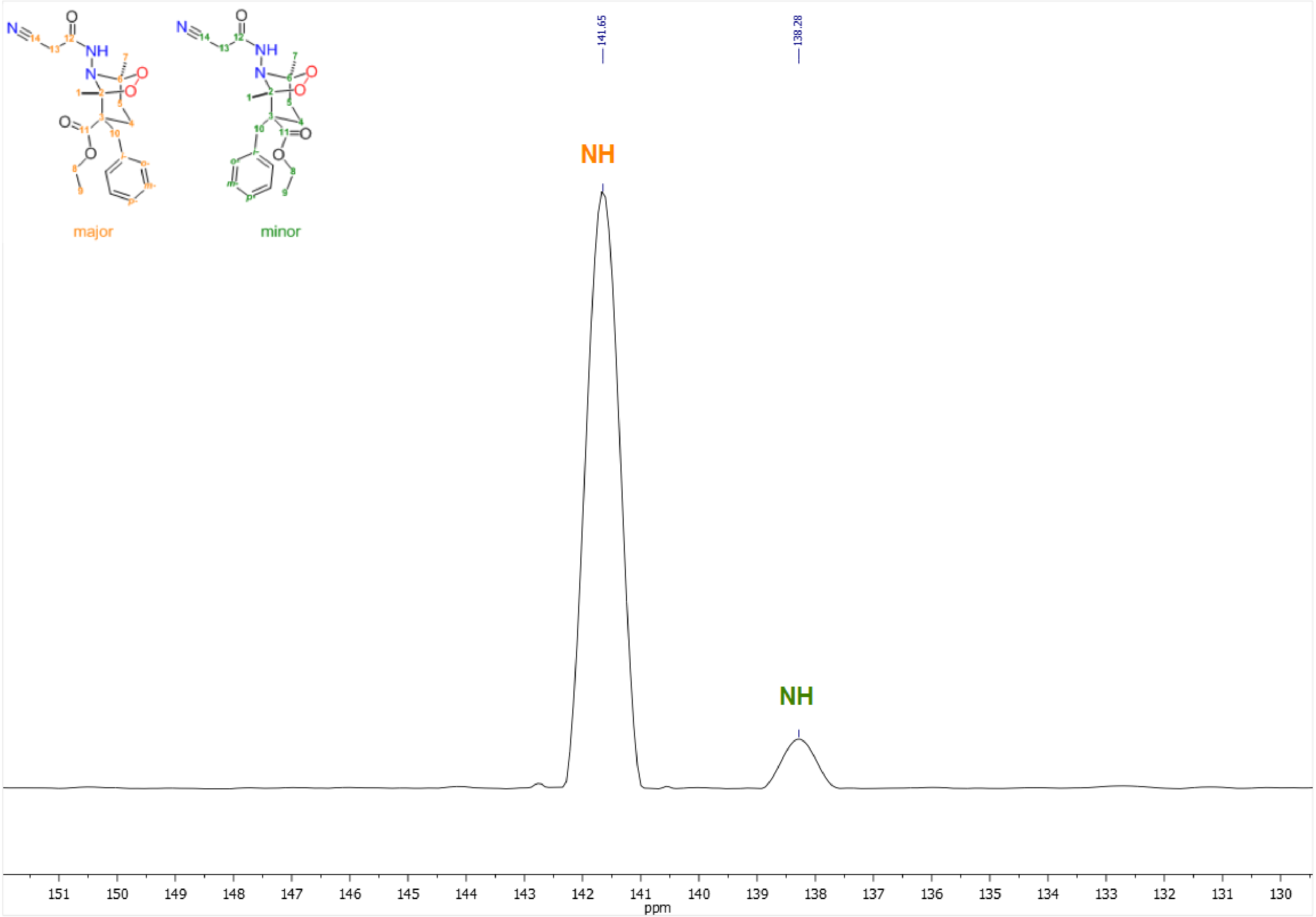

^{13}C NMR (75.48 MHz, $CDCl_3$). Ethyl 2-benzyl-8-(2-cyanoacetamido)-1,5-dimethyl-6,7-dioxa-8-azabicyclo[3.2.1]octane-2-carboxylate, 27a + 27b

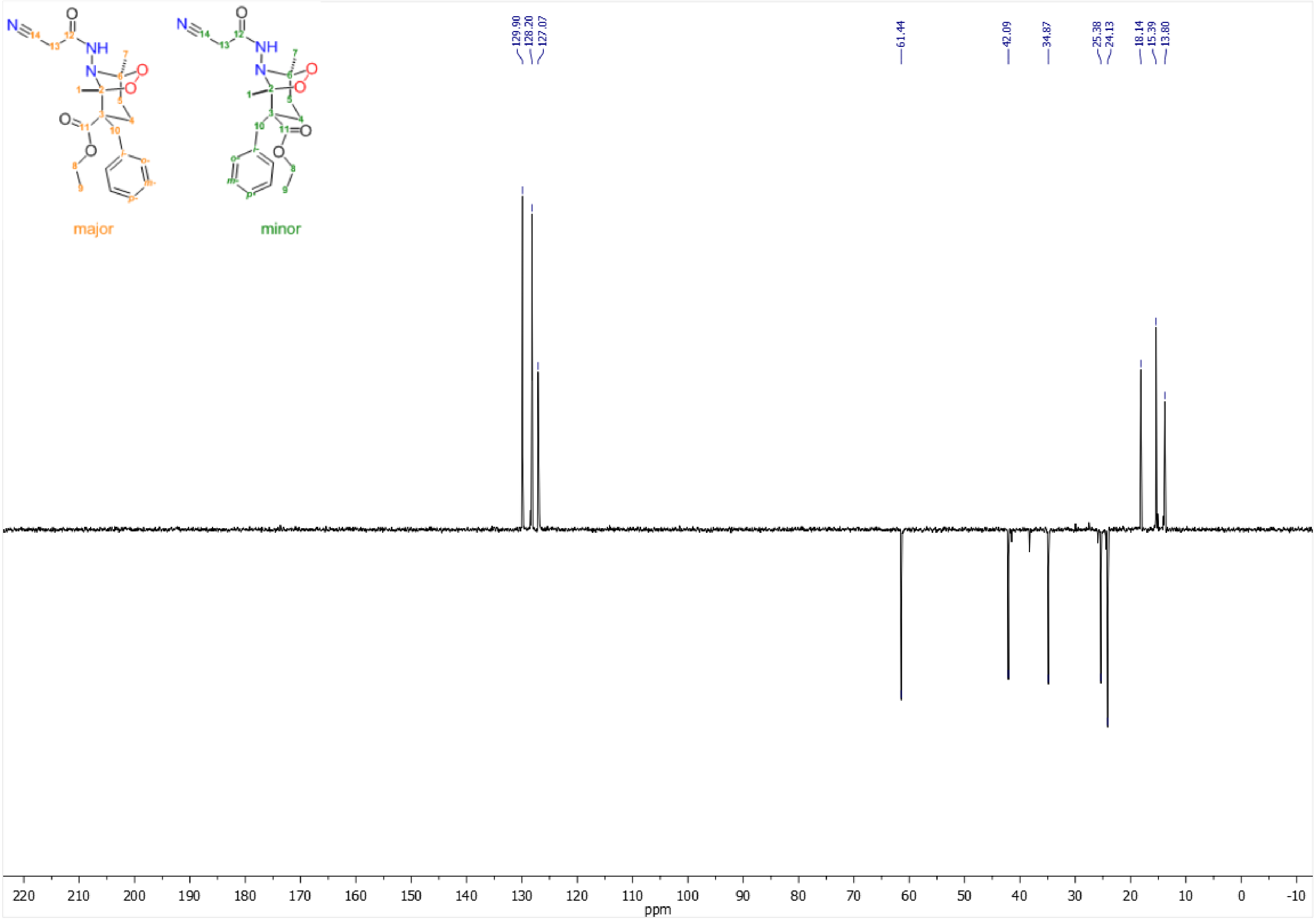

Ethyl 2-benzyl-8-(2-cyanoacetamido)-1,5-dimethyl-6,7-dioxa-8-azabicyclo[3.2.1]octane-2-carboxylate, 27a + 27b

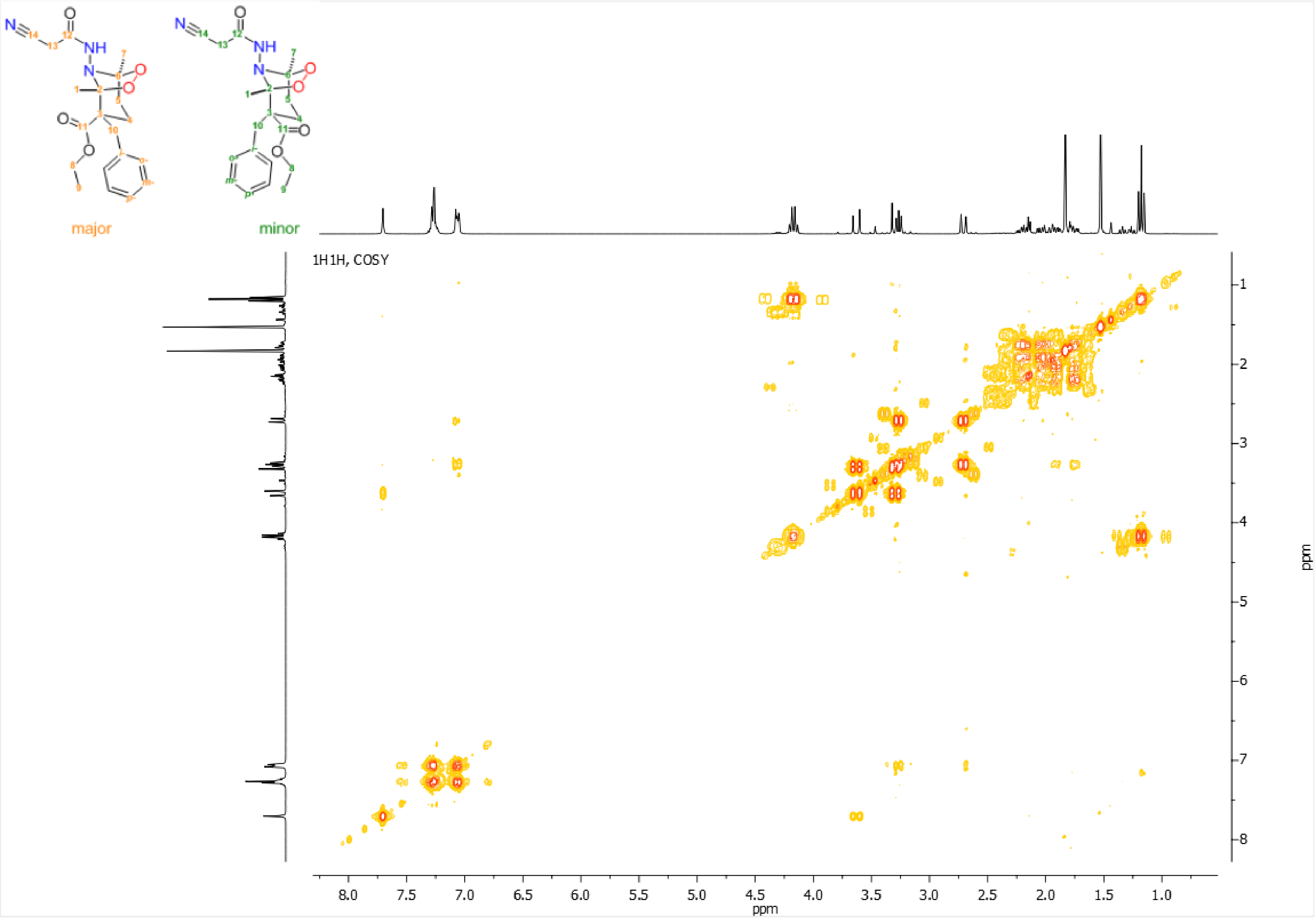

Ethyl 2-benzyl-8-(2-cyanoacetamido)-1,5-dimethyl-6,7-dioxa-8-azabicyclo[3.2.1]octane-2-carboxylate, 27a + 27b

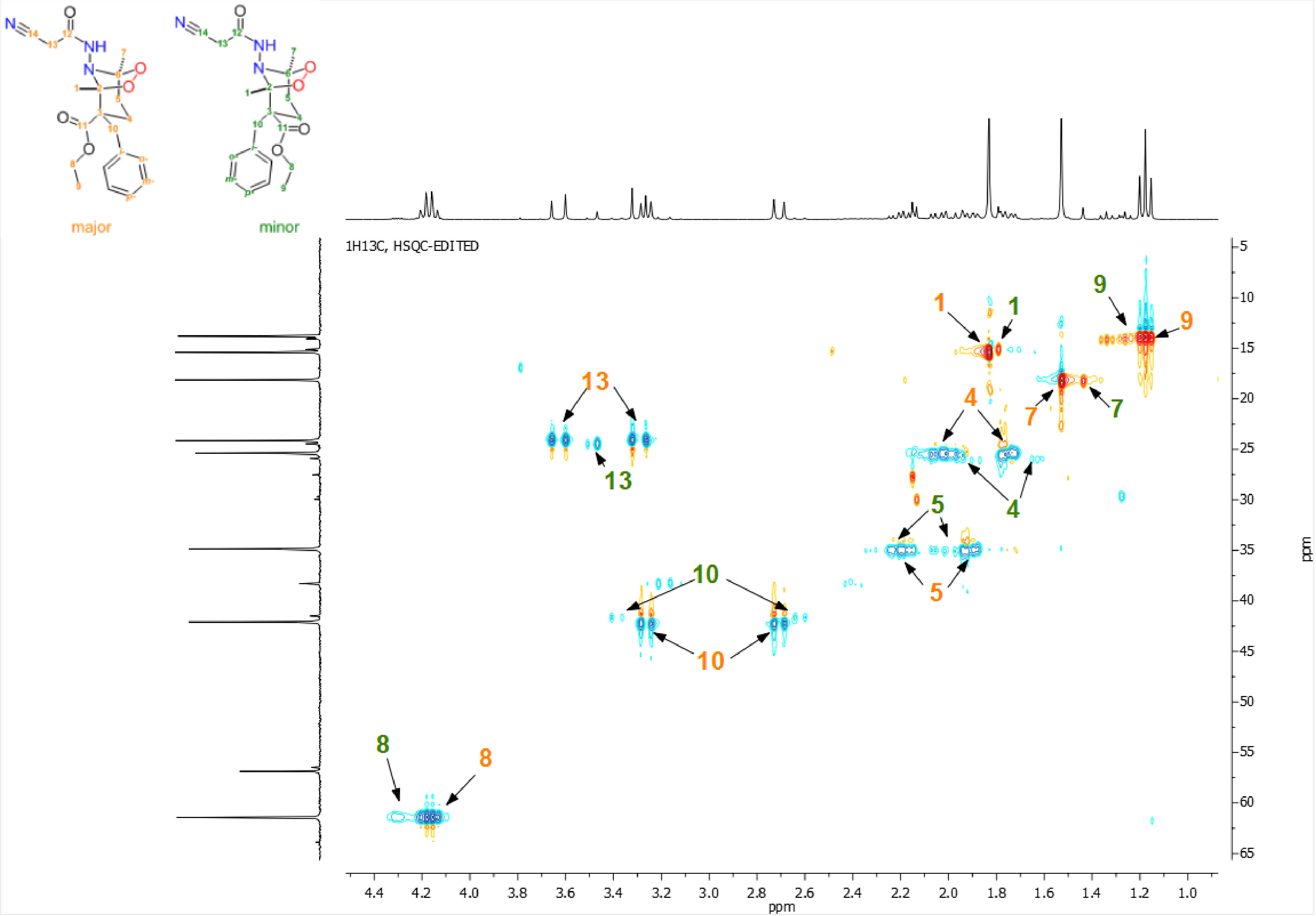

Ethyl 2-benzyl-8-(2-cyanoacetamido)-1,5-dimethyl-6,7-dioxa-8-azabicyclo[3.2.1]octane-2-carboxylate, 27a + 27b

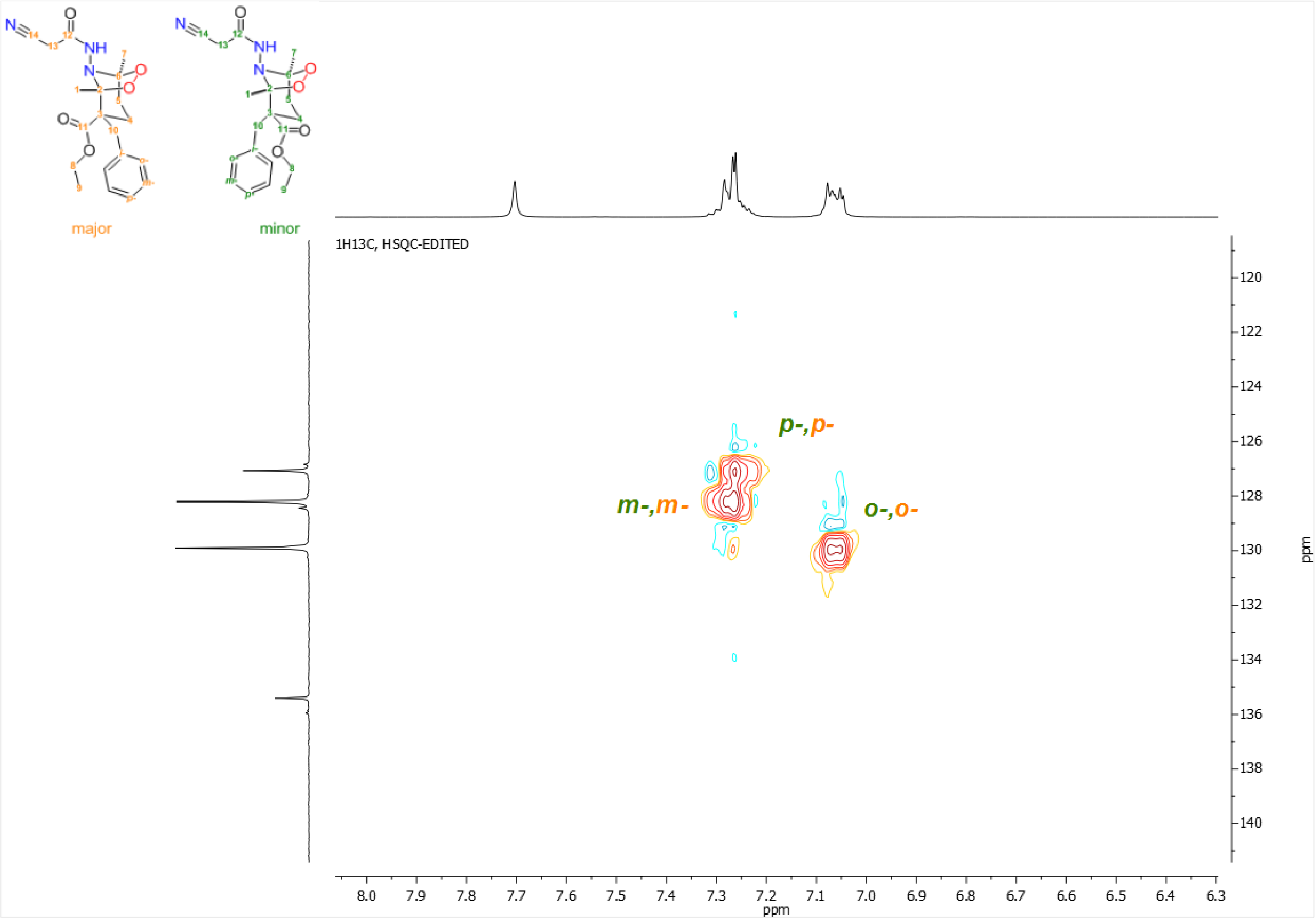

Ethyl 2-benzyl-8-(2-cyanoacetamido)-1,5-dimethyl-6,7-dioxa-8-azabicyclo[3.2.1]octane-2-carboxylate, 27a + 27b

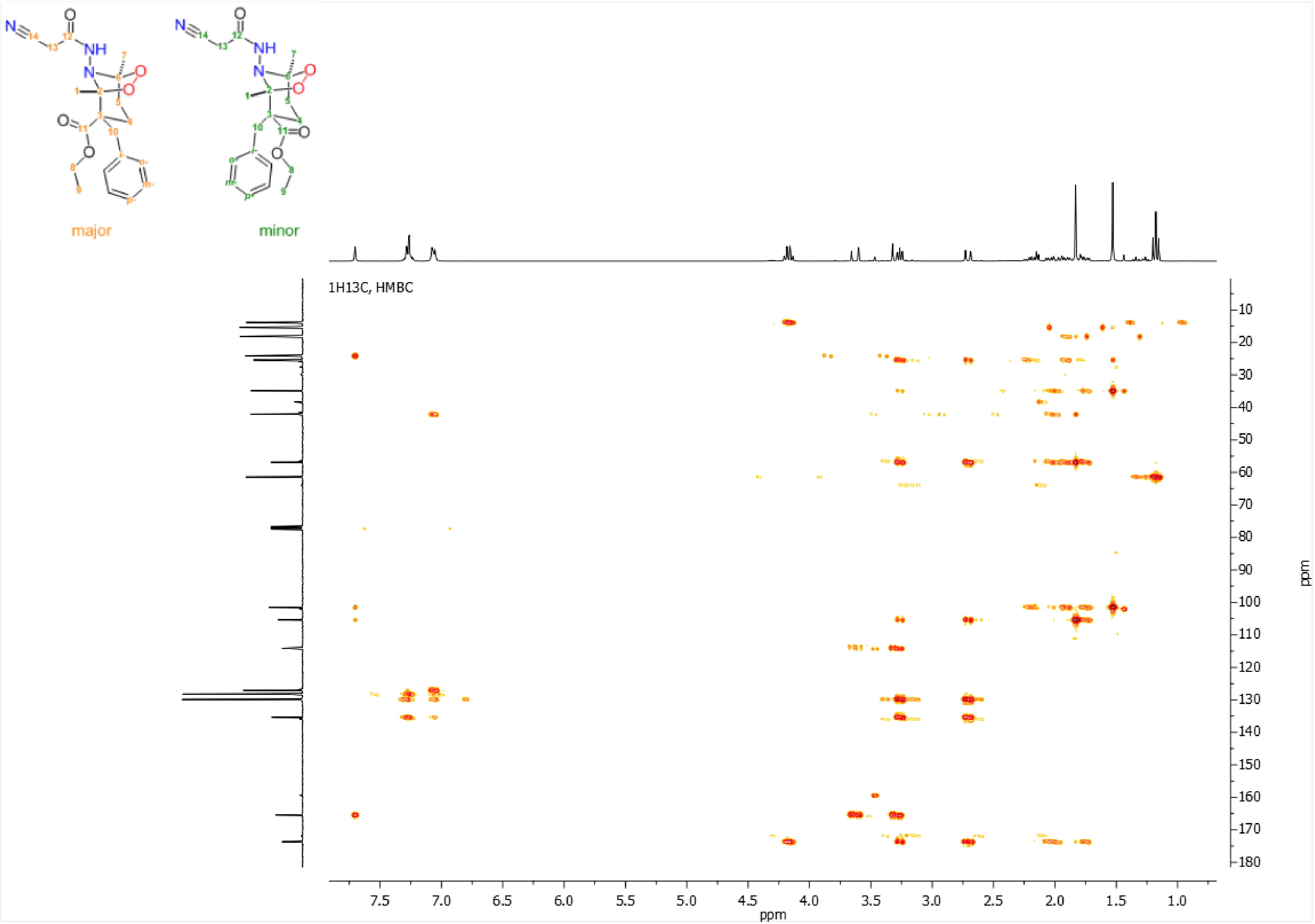

Ethyl 2-benzyl-8-(2-cyanoacetamido)-1,5-dimethyl-6,7-dioxa-8-azabicyclo[3.2.1]octane-2-carboxylate, 27a + 27b

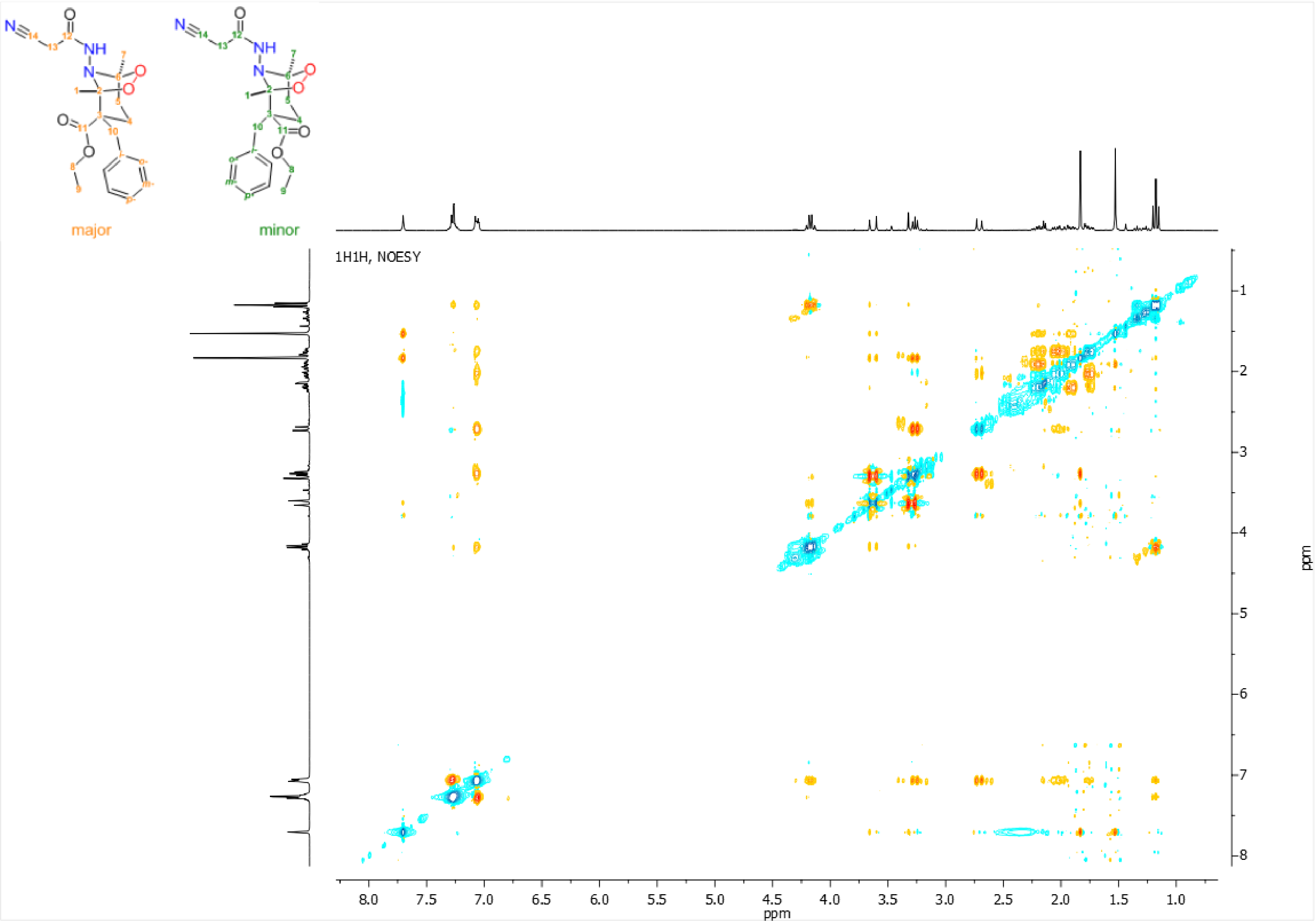

Ethyl 2-benzyl-8-(2-cyanoacetamido)-1,5-dimethyl-6,7-dioxa-8-azabicyclo[3.2.1]octane-2-carboxylate, 27a + 27b

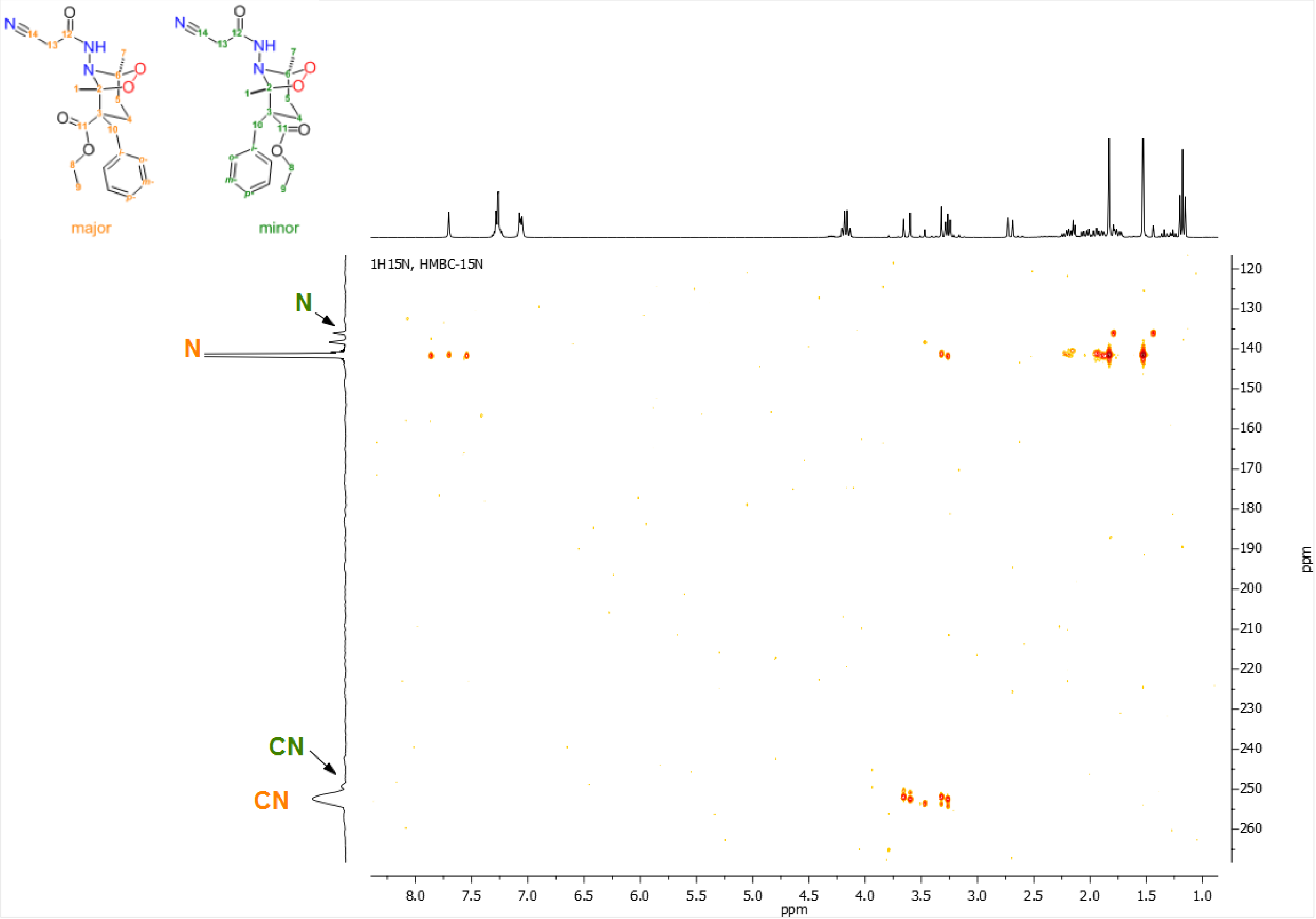

Ethyl 2-benzyl-8-(2-cyanoacetamido)-1,5-dimethyl-6,7-dioxa-8-azabicyclo[3.2.1]octane-2-carboxylate, 27a + 27b

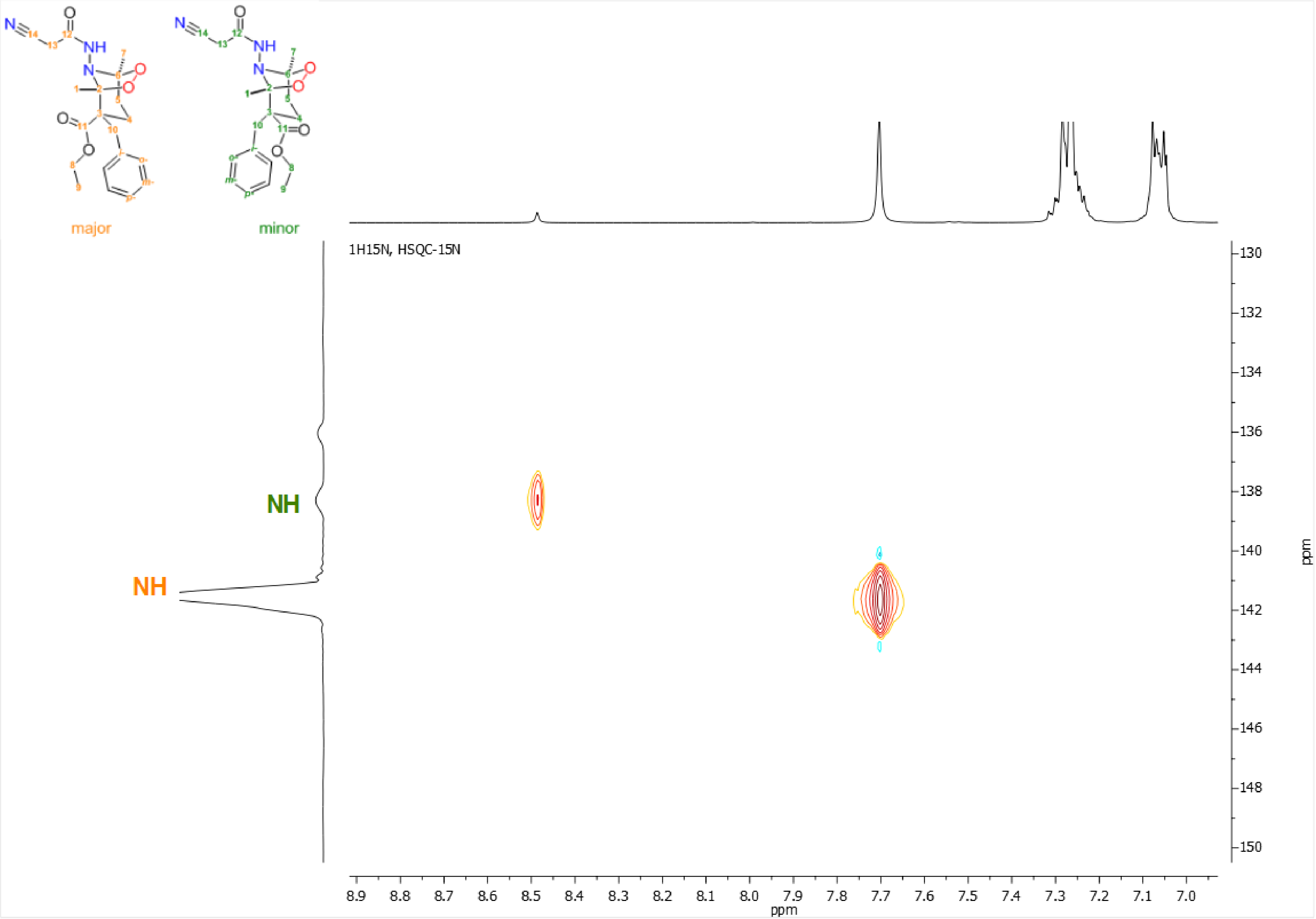

^{1}H NMR (300.13 MHz, $CDCl_3$). Allyl 2-allyl-8-(2-cyanoacetamido)-1,5-dimethyl-6,7-dioxa-8-azabicyclo[3.2.1]octane-2-carboxylate, 28a + 28b

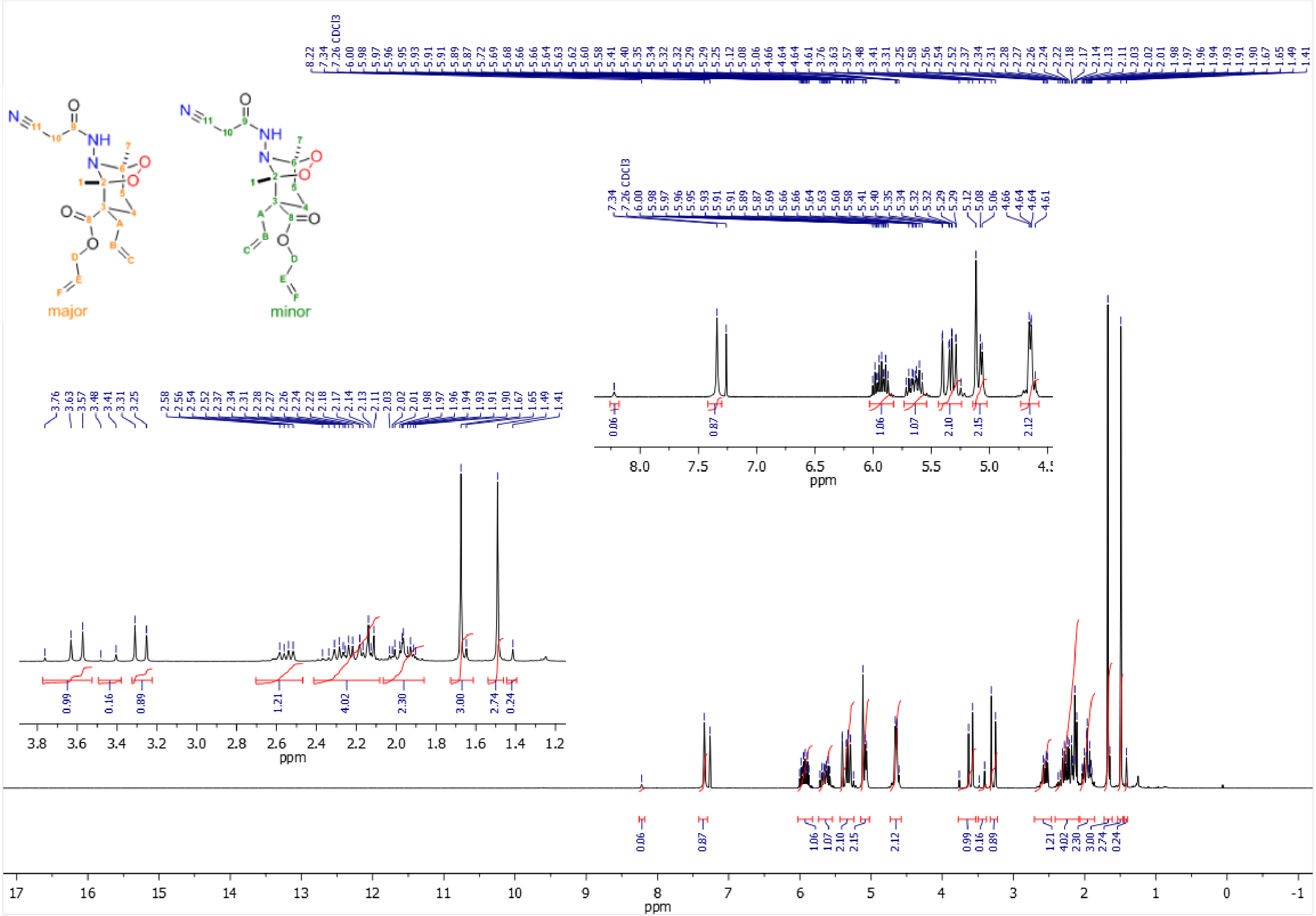

^{1}H NMR (300.13 MHz, CDCl₃). Allyl 2-allyl-8-(2-cyanoacetamido)-1,5-dimethyl-6,7-dioxa-8-azabicyclo[3.2.1]octane-2-carboxylate, 28a + 28b

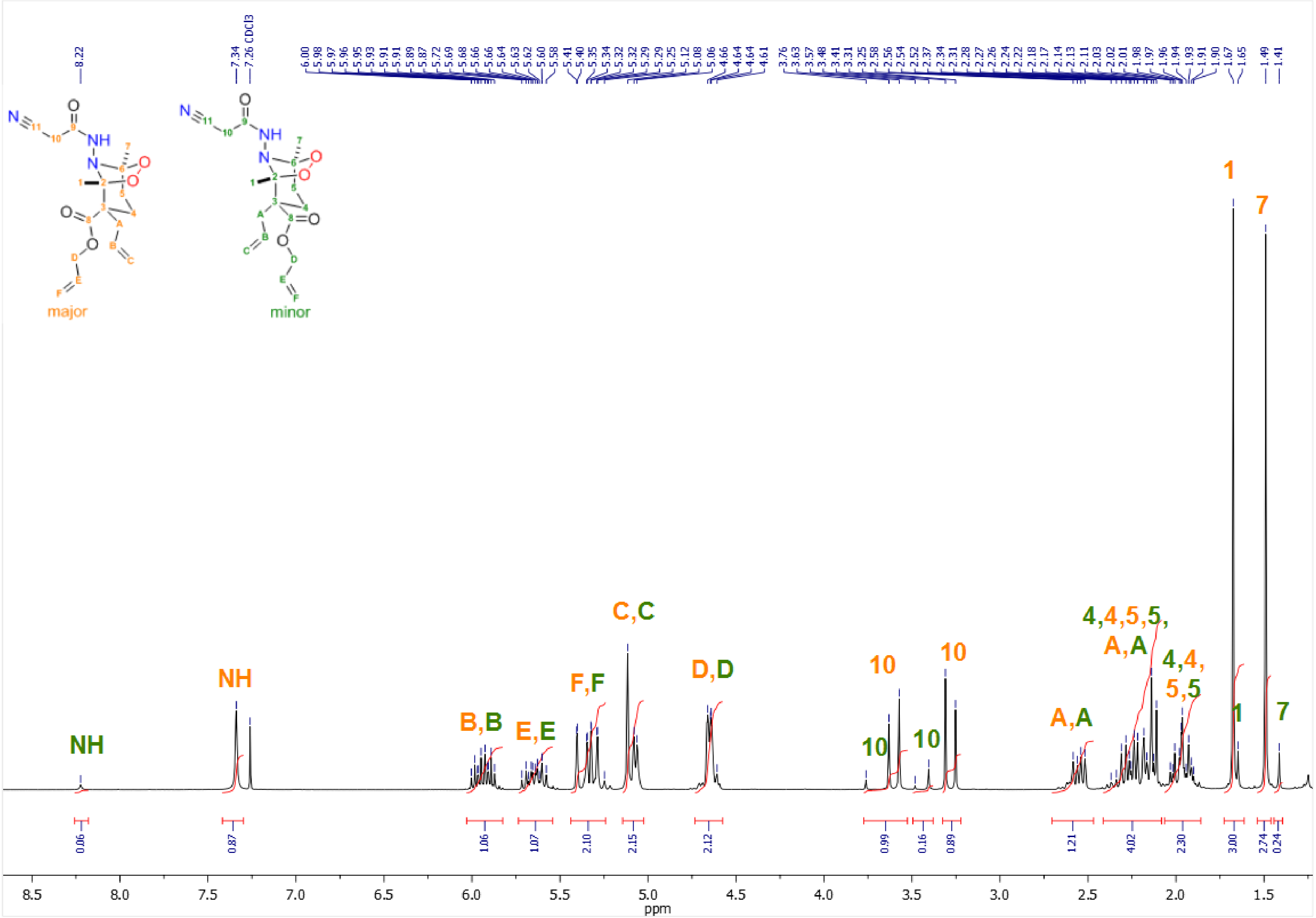

^{13}C NMR (75.48 MHz, CDCl$_3$). Allyl 2-allyl-8-(2-cyanoacetamido)-1,5-dimethyl-6,7-dioxa-8-azabicyclo[3.2.1]octane-2-carboxylate, 28a + 28b

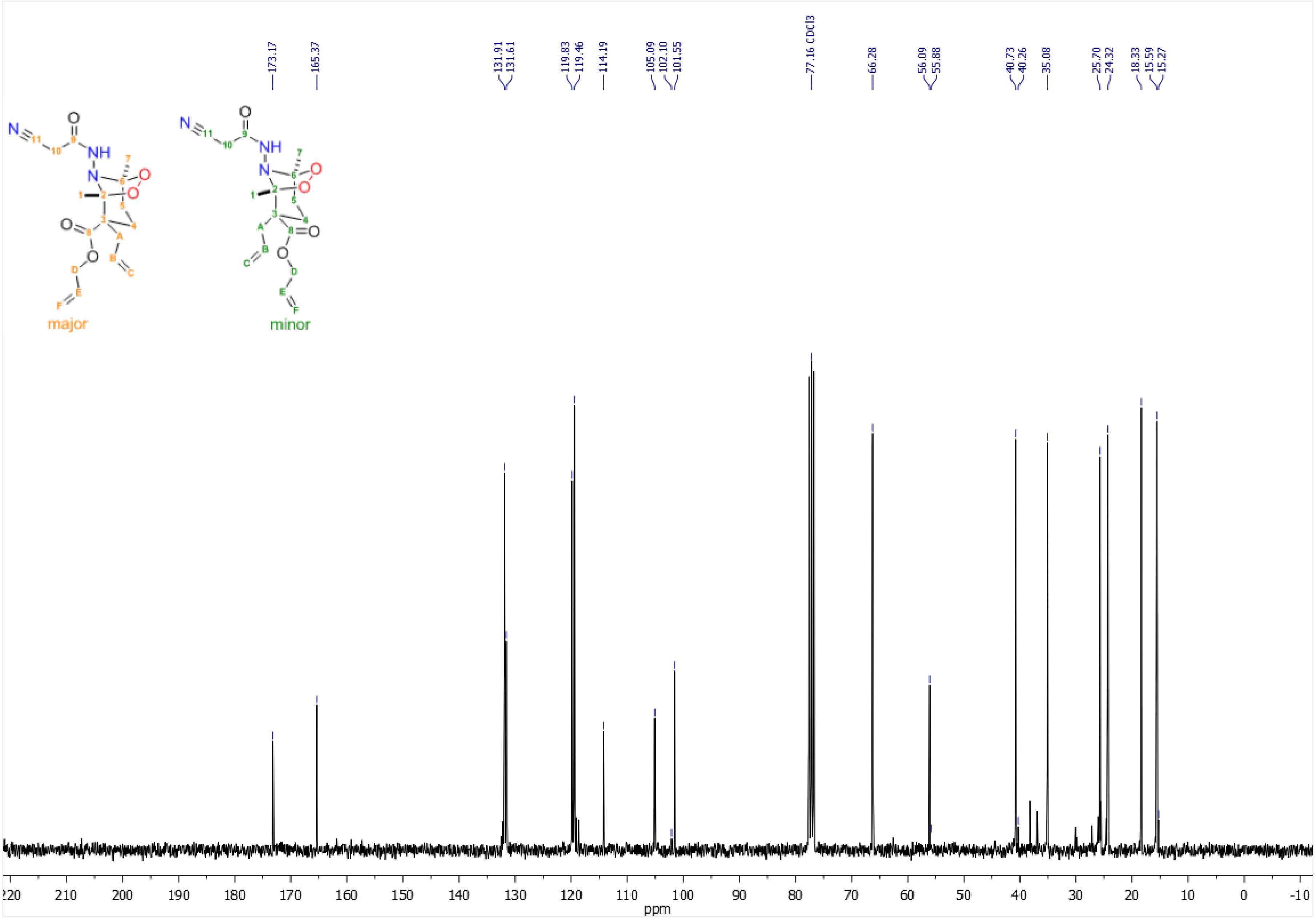

^{13}C NMR (75.48 MHz, CDCl3). Allyl 2-allyl-8-(2-cyanoacetamido)-1,5-dimethyl-6,7-dioxa-8-azabicyclo[3.2.1]octane-2-carboxylate, 28a + 28b

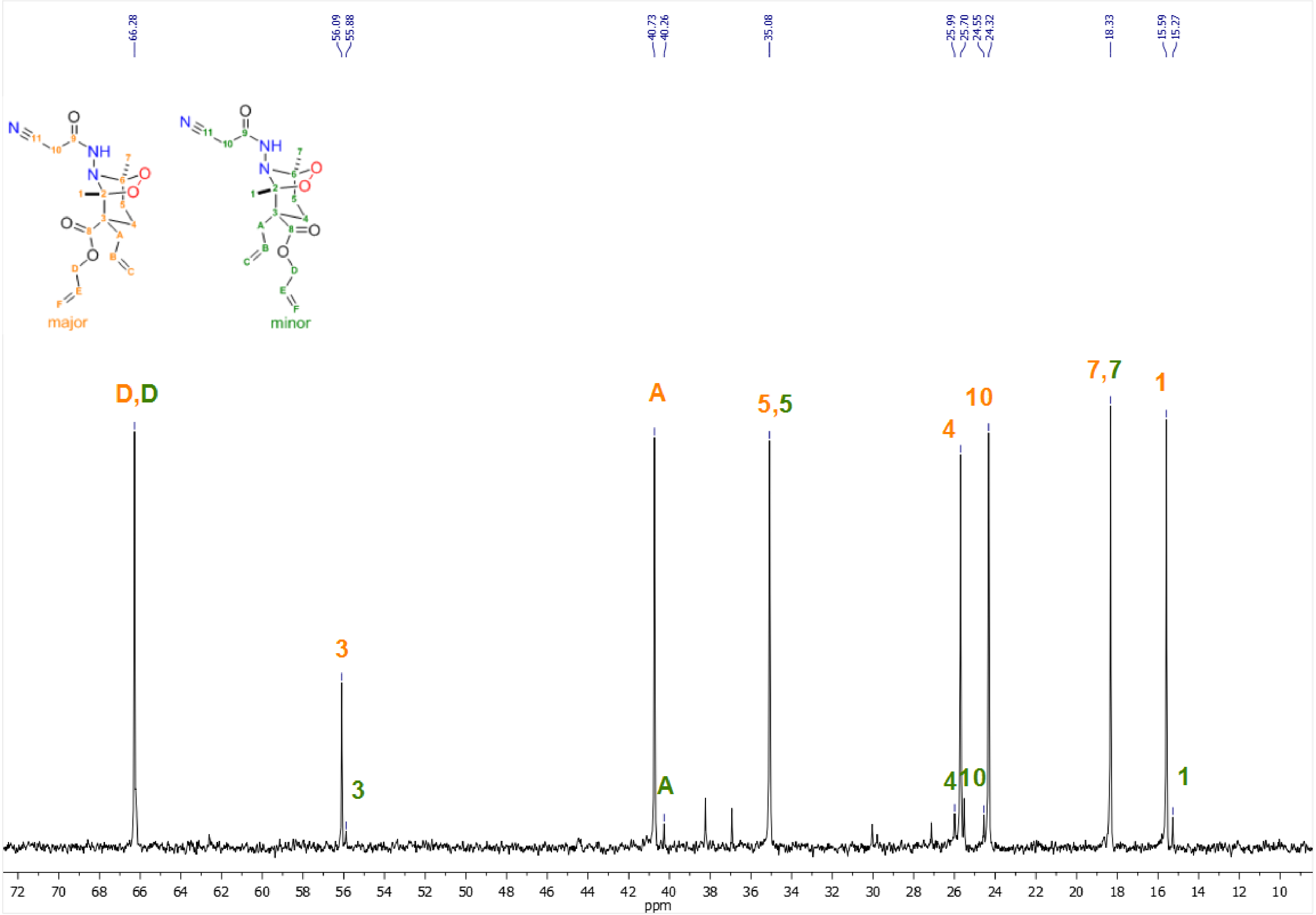

^{13}C NMR (75.48 MHz, CDCl$_3$). Allyl 2-allyl-8-(2-cyanoacetamido)-1,5-dimethyl-6,7-dioxa-8-azabicyclo[3.2.1]octane-2-carboxylate, 28a + 28b

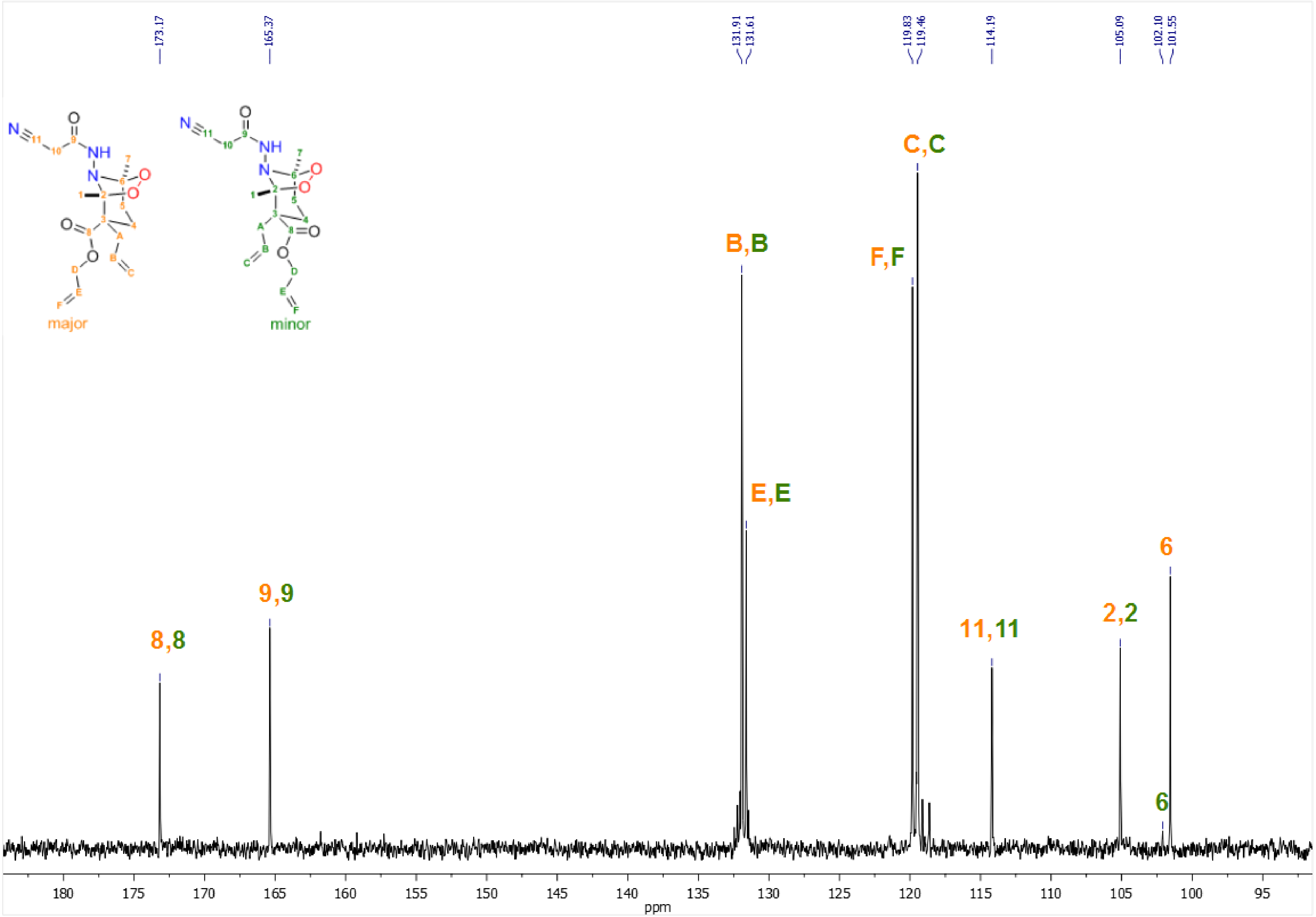

^{15}N (40.56 MHz, $CDCl_3$). Allyl 2-allyl-8-(2-cyanoacetamido)-1,5-dimethyl-6,7-dioxa-8-azabicyclo[3.2.1]octane-2-carboxylate, 28a + 28b

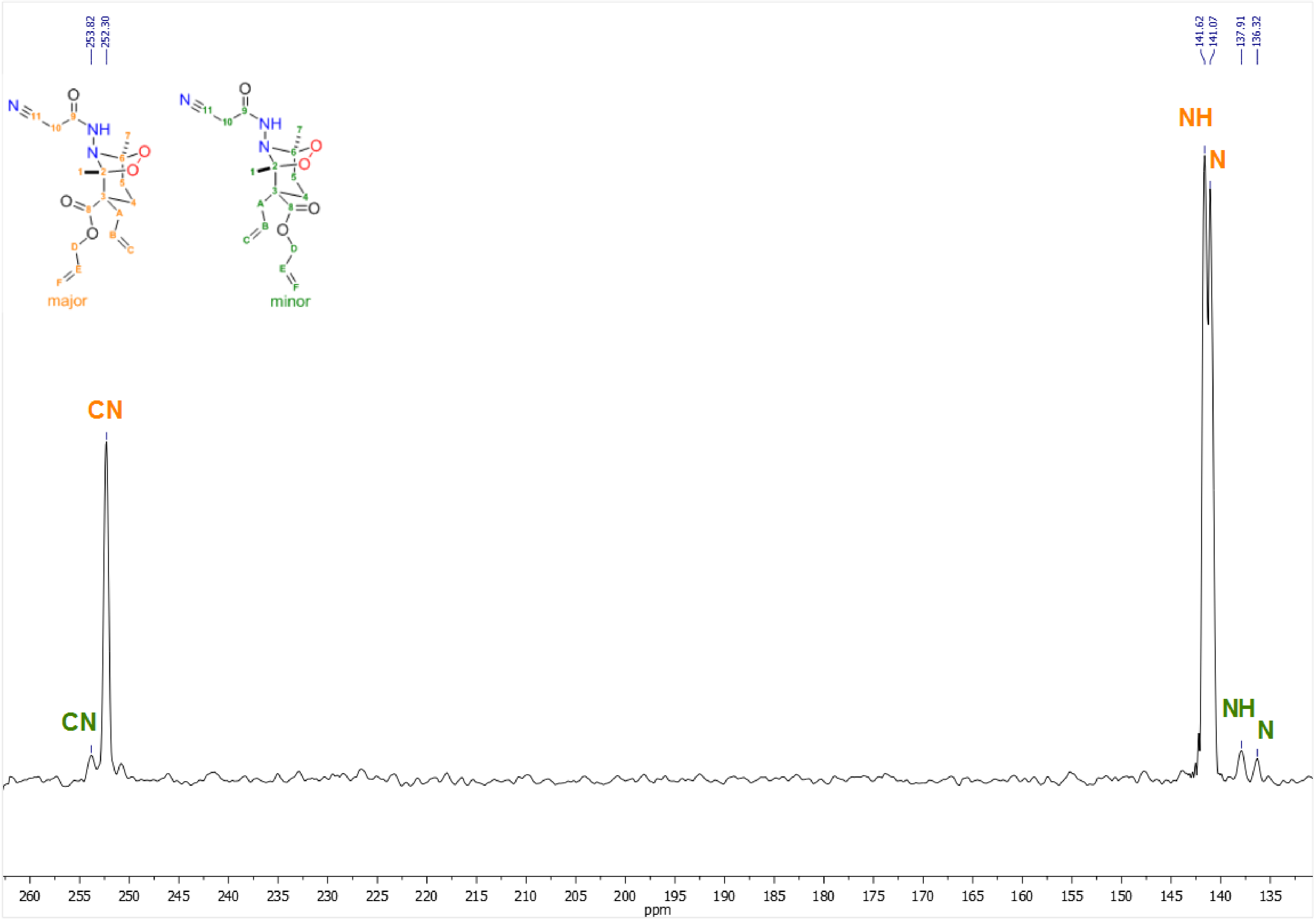

^{13}C NMR (75.48 MHz, $CDCl_3$). Allyl 2-allyl-8-(2-cyanoacetamido)-1,5-dimethyl-6,7-dioxa-8-azabicyclo[3.2.1]octane-2-carboxylate, 28a + 28b

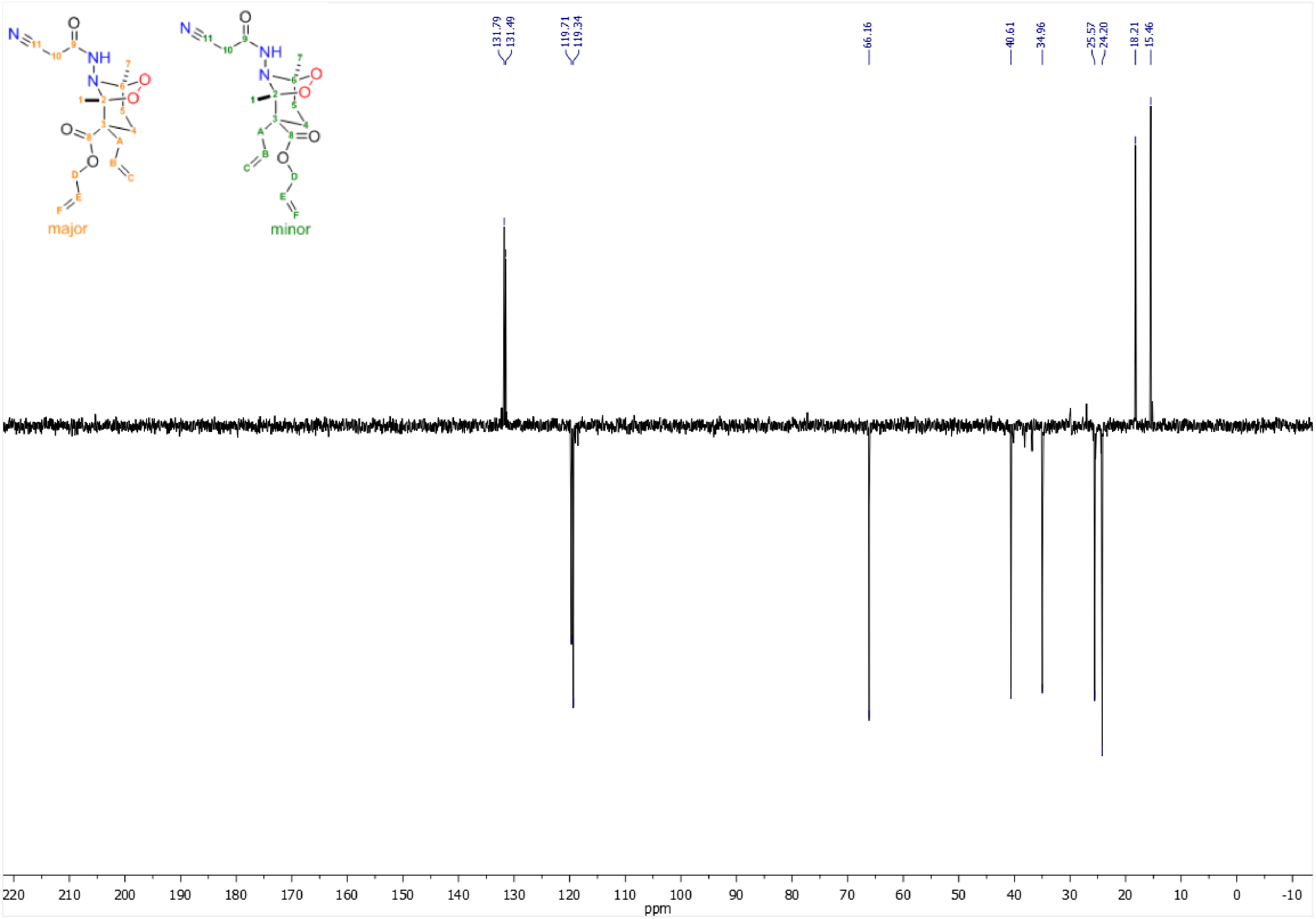

Allyl 2-allyl-8-(2-cyanoacetamido)-1,5-dimethyl-6,7-dioxa-8-azabicyclo[3.2.1]octane-2-carboxylate, 28a + 28b

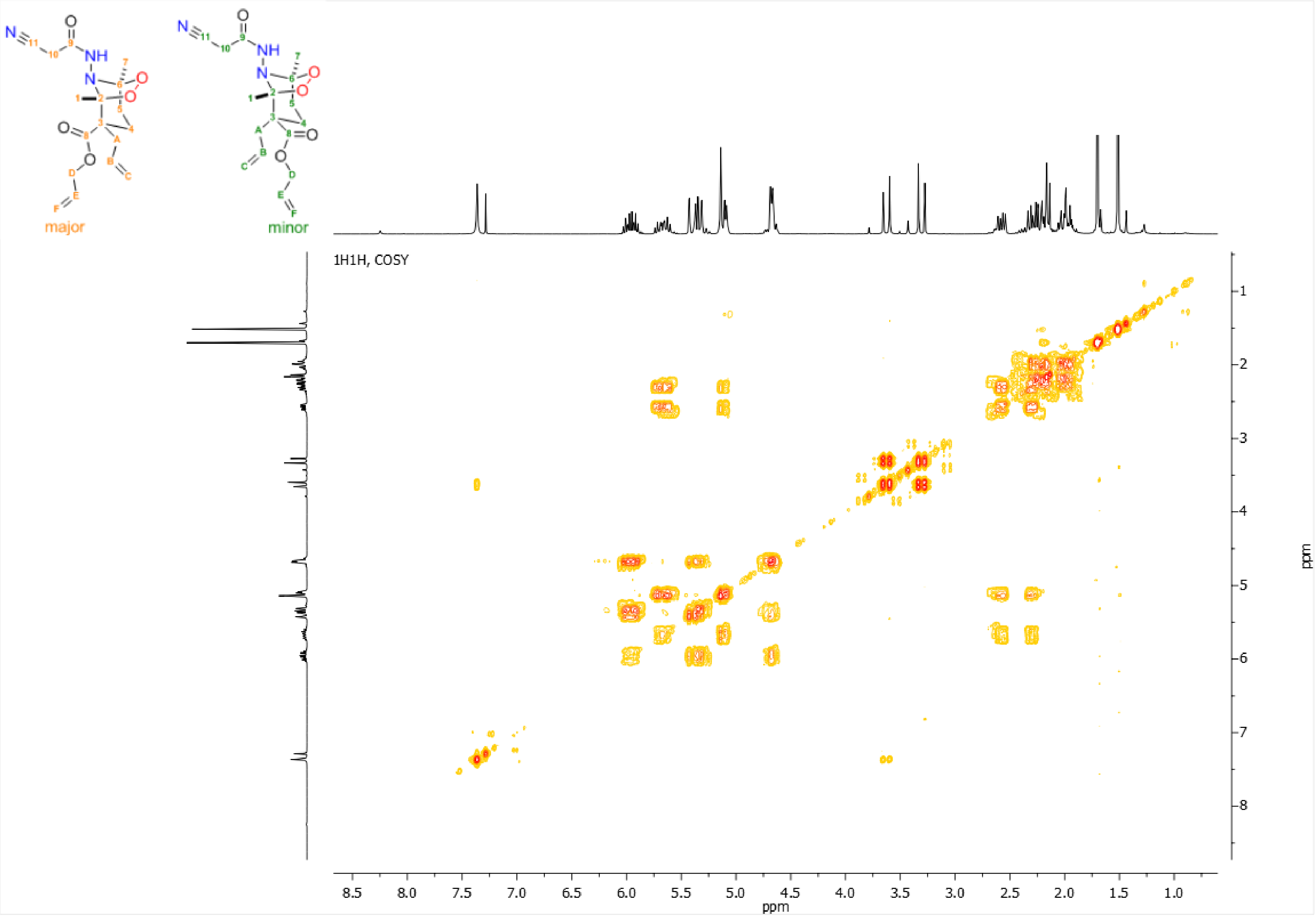

Allyl 2-allyl-8-(2-cyanoacetamido)-1,5-dimethyl-6,7-dioxa-8-azabicyclo[3.2.1]octane-2-carboxylate, 28a + 28b

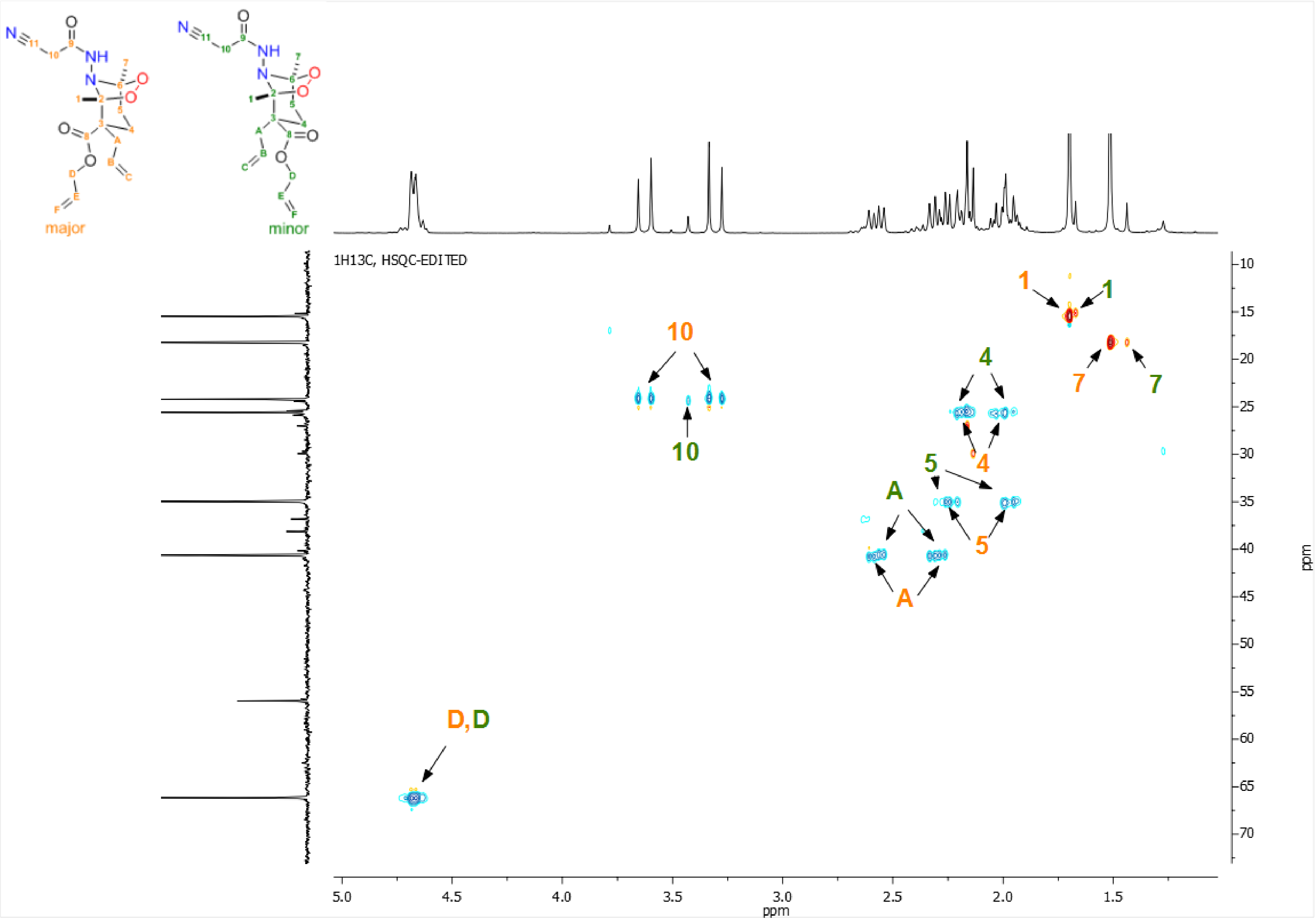

Allyl 2-allyl-8-(2-cyanoacetamido)-1,5-dimethyl-6,7-dioxa-8-azabicyclo[3.2.1]octane-2-carboxylate, 28a + 28b

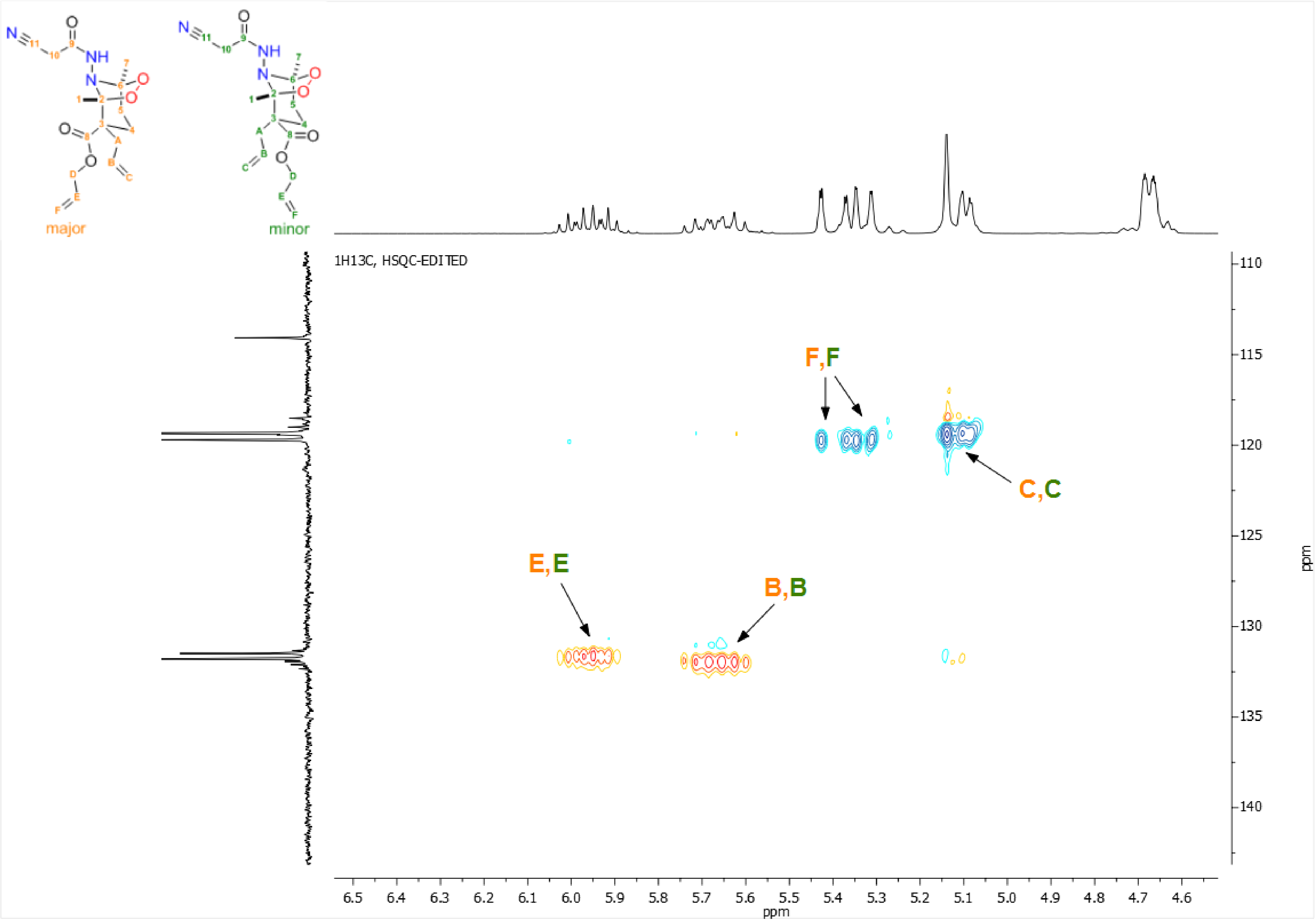

Allyl 2-allyl-8-(2-cyanoacetamido)-1,5-dimethyl-6,7-dioxa-8-azabicyclo[3.2.1]octane-2-carboxylate, 28a + 28b

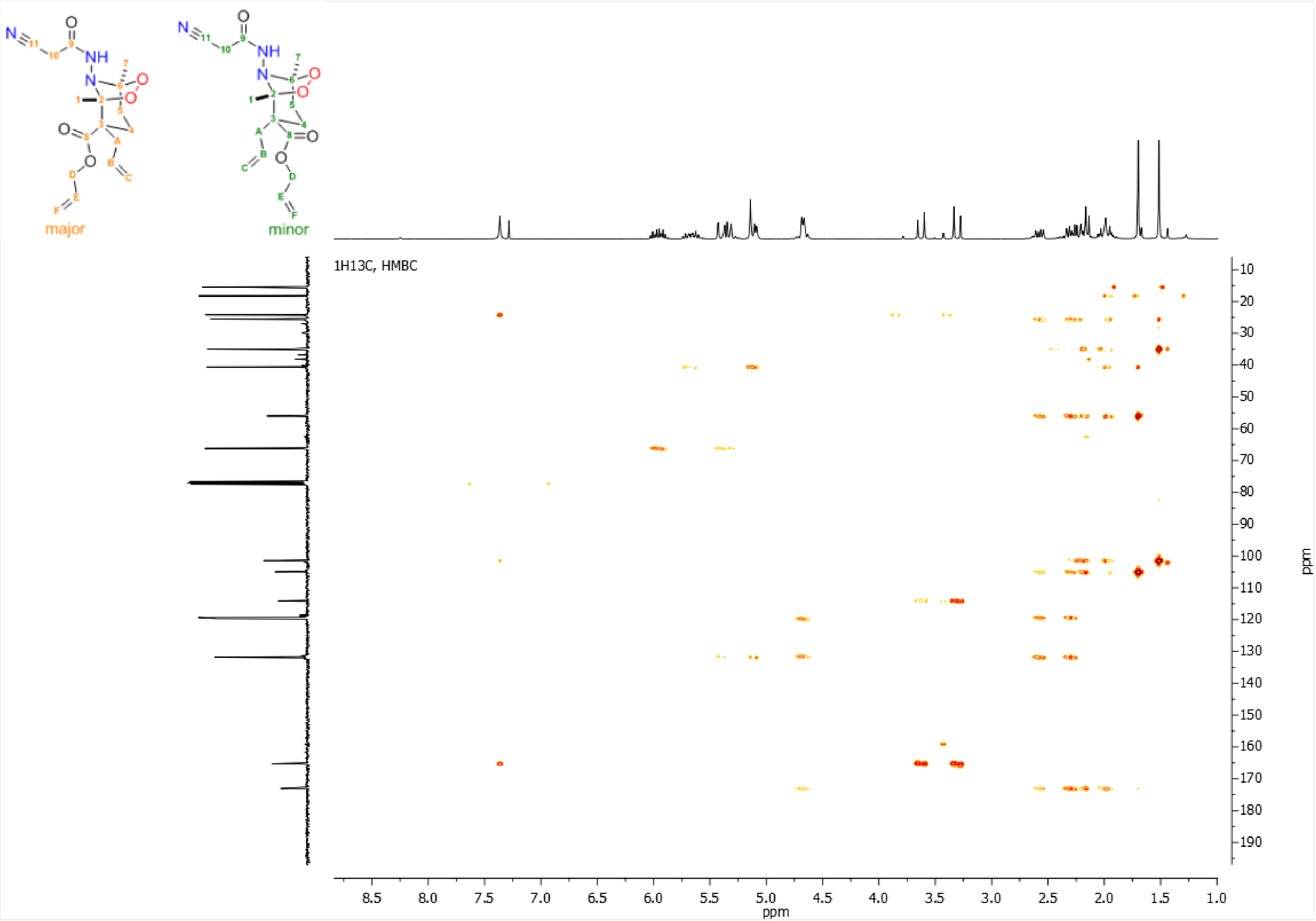

Allyl 2-allyl-8-(2-cyanoacetamido)-1,5-dimethyl-6,7-dioxa-8-azabicyclo[3.2.1]octane-2-carboxylate, 28a + 28b

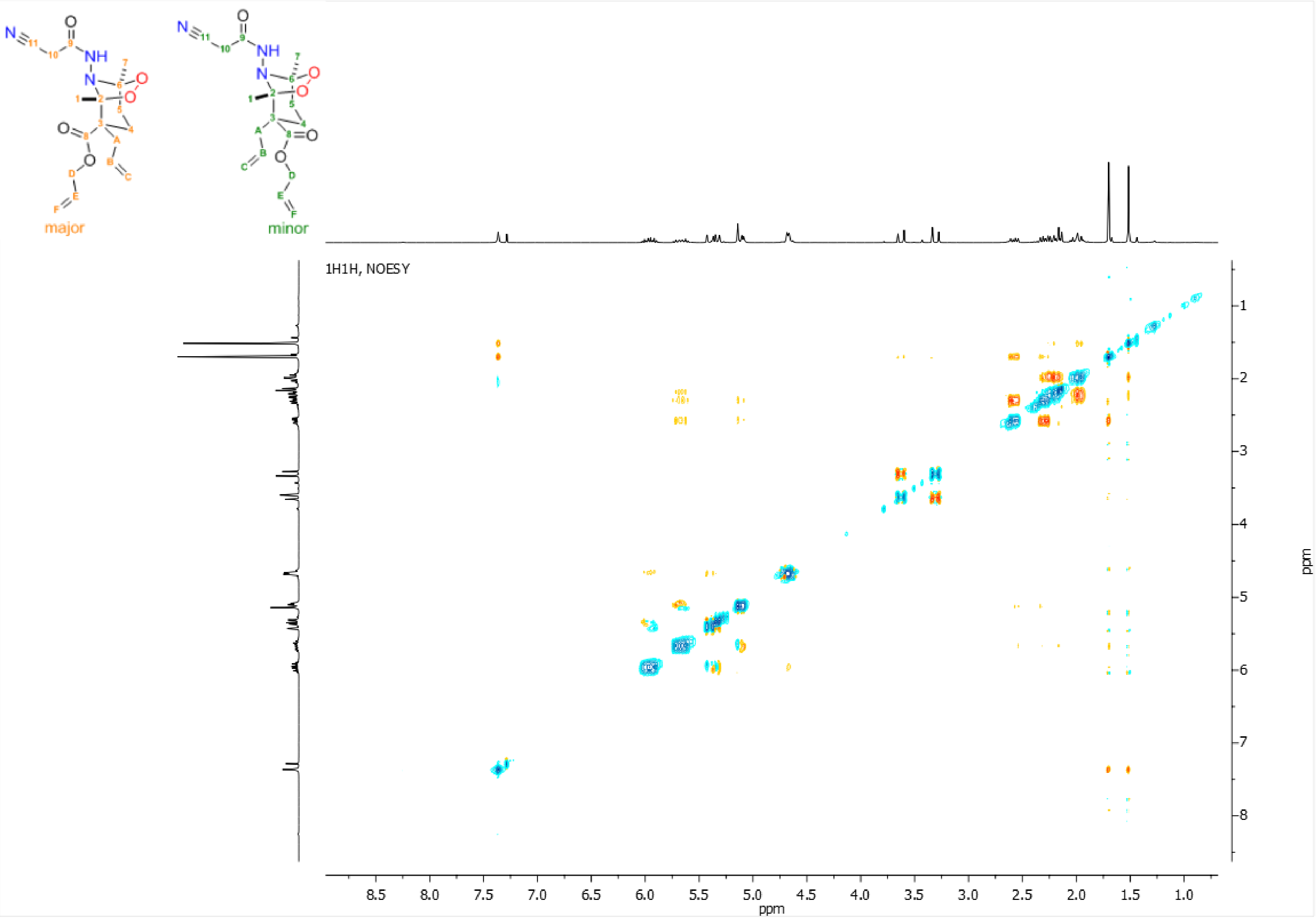

Allyl 2-allyl-8-(2-cyanoacetamido)-1,5-dimethyl-6,7-dioxa-8-azabicyclo[3.2.1]octane-2-carboxylate, 28a + 28b

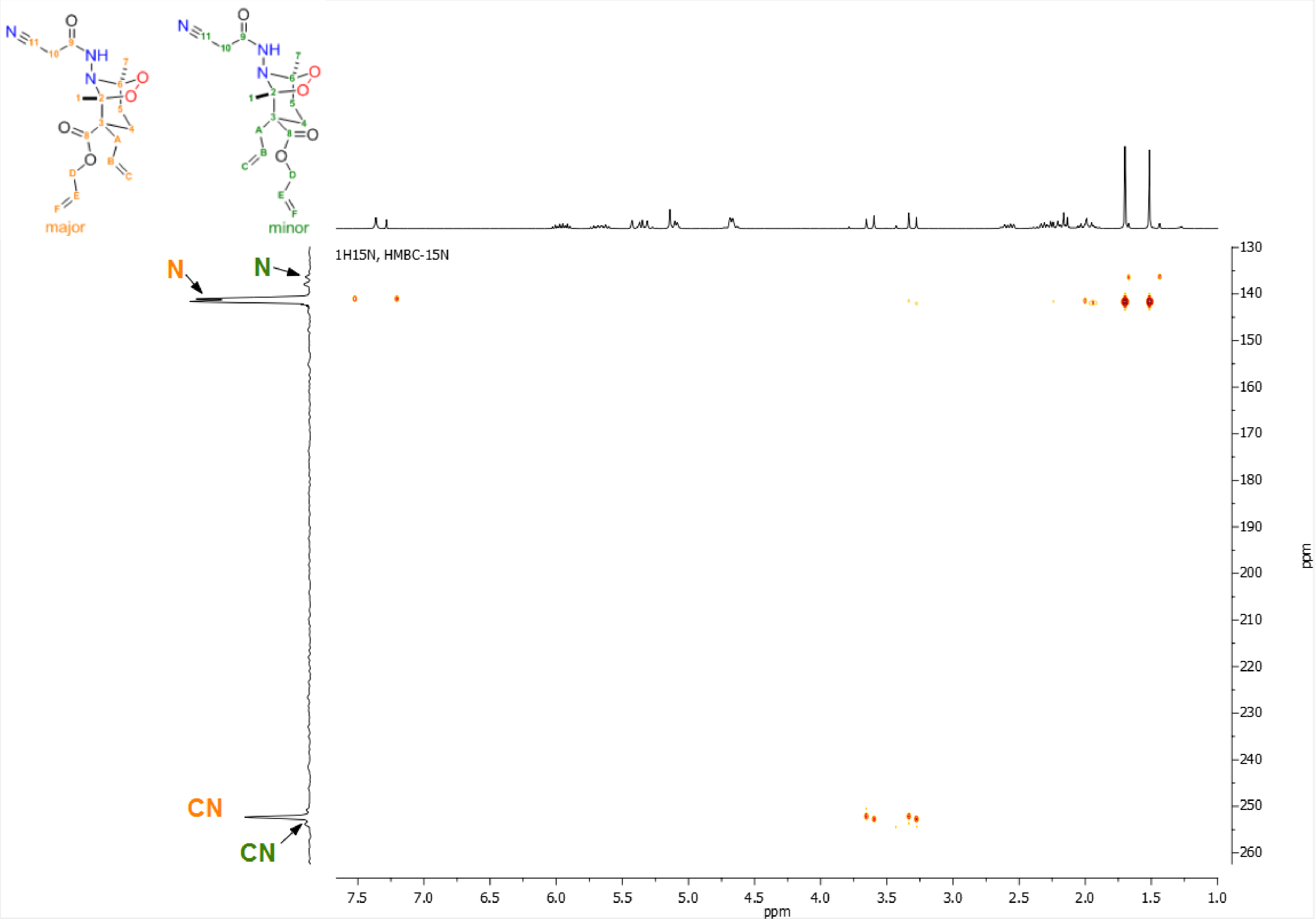

Allyl 2-allyl-8-(2-cyanoacetamido)-1,5-dimethyl-6,7-dioxa-8-azabicyclo[3.2.1]octane-2-carboxylate, 28a + 28b

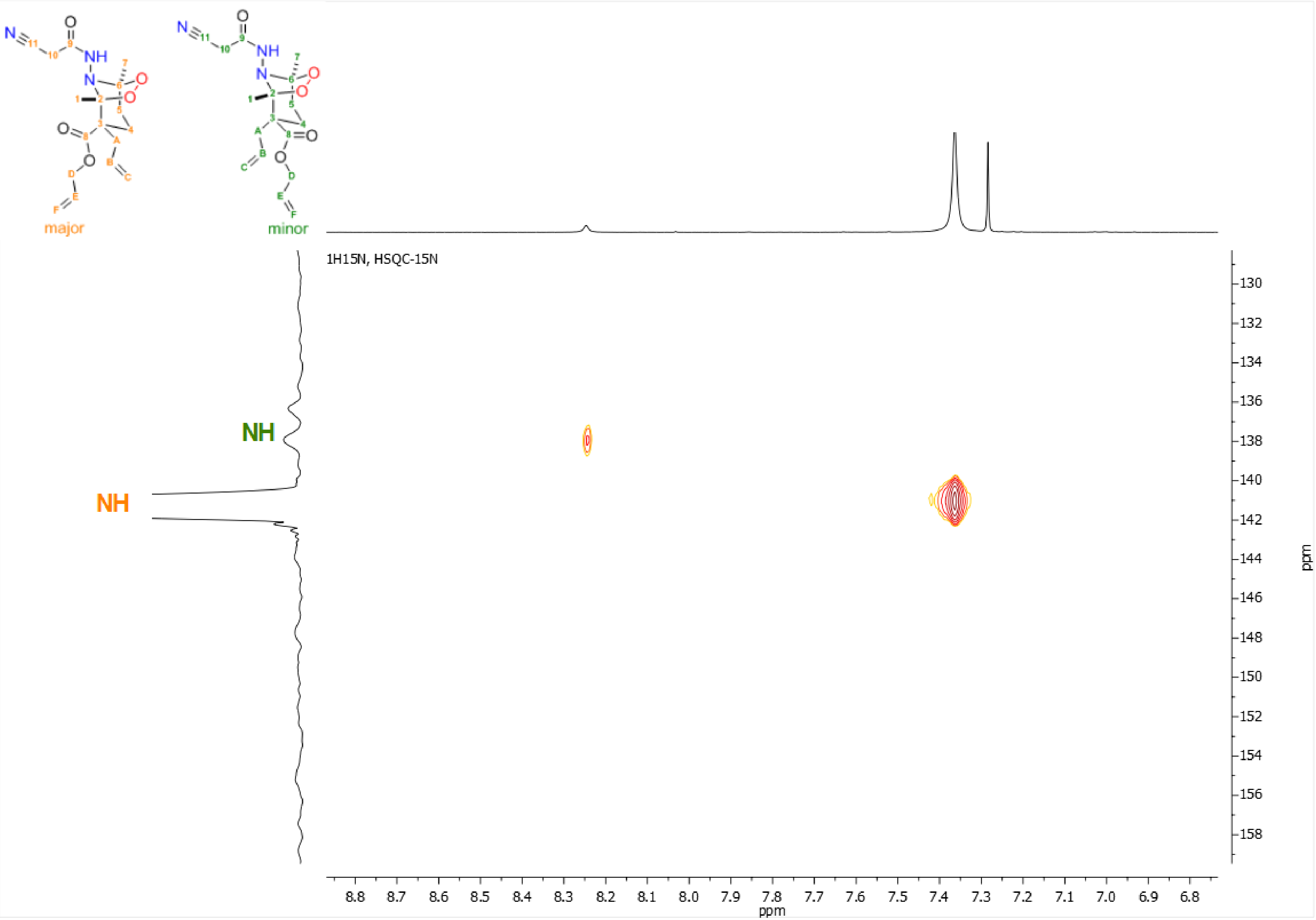

^{1}H NMR (300.13 MHz, CDCl$_3$). Ethyl 2-(4-chlorobenzyl)-1,5-dimethyl-8-((4-methylphenyl)sulfonamido)-6,7-dioxa-8-azabicyclo[3.2.1]octane-2-carboxylate, 29

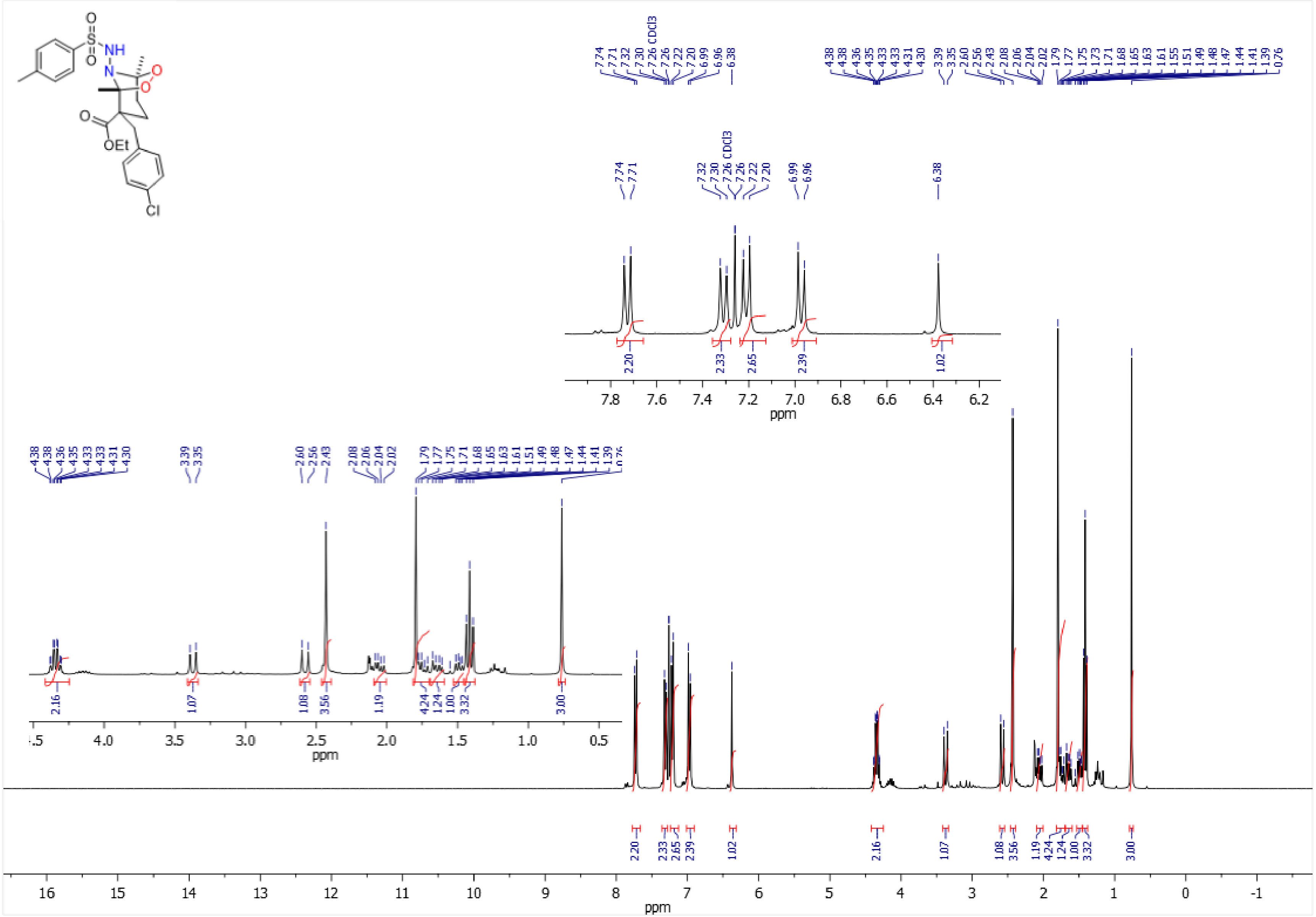

^{13}C NMR (75.48 MHz, $CDCl_3$). Ethyl 2-(4-chlorobenzyl)-1,5-dimethyl-8-((4-methylphenyl)sulfonamido)-6,7-dioxa-8-azabicyclo[3.2.1]octane-2-carboxylate, 29

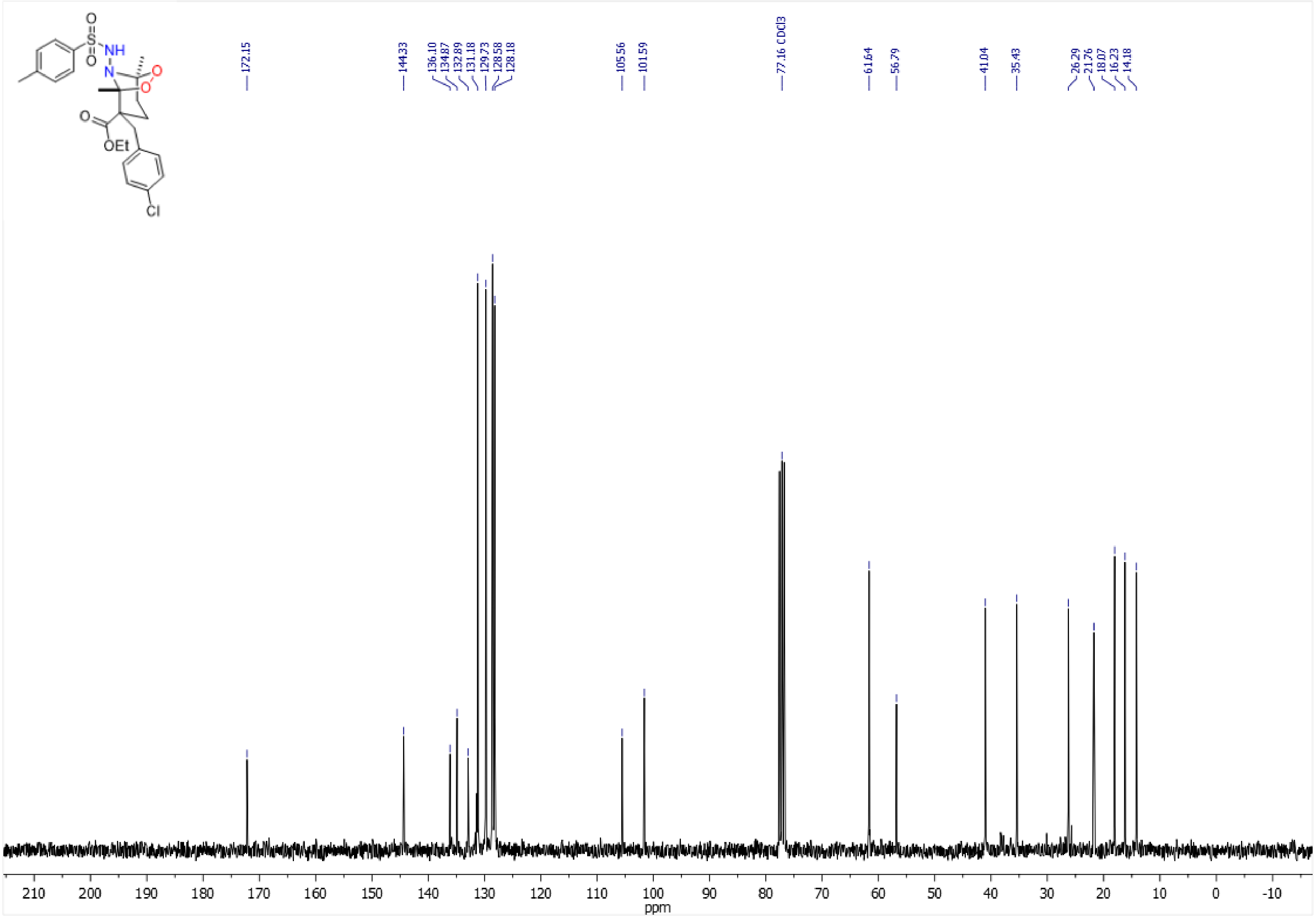

^{1}H NMR (300.13 MHz, CDCl3). Ethyl 2-(4-fluorobenzyl)-1,5-dimethyl-8-((4-methylphenyl)sulfonamido)-6,7-dioxa-8-azabicyclo[3.2.1]octane-2-carboxylate, 30

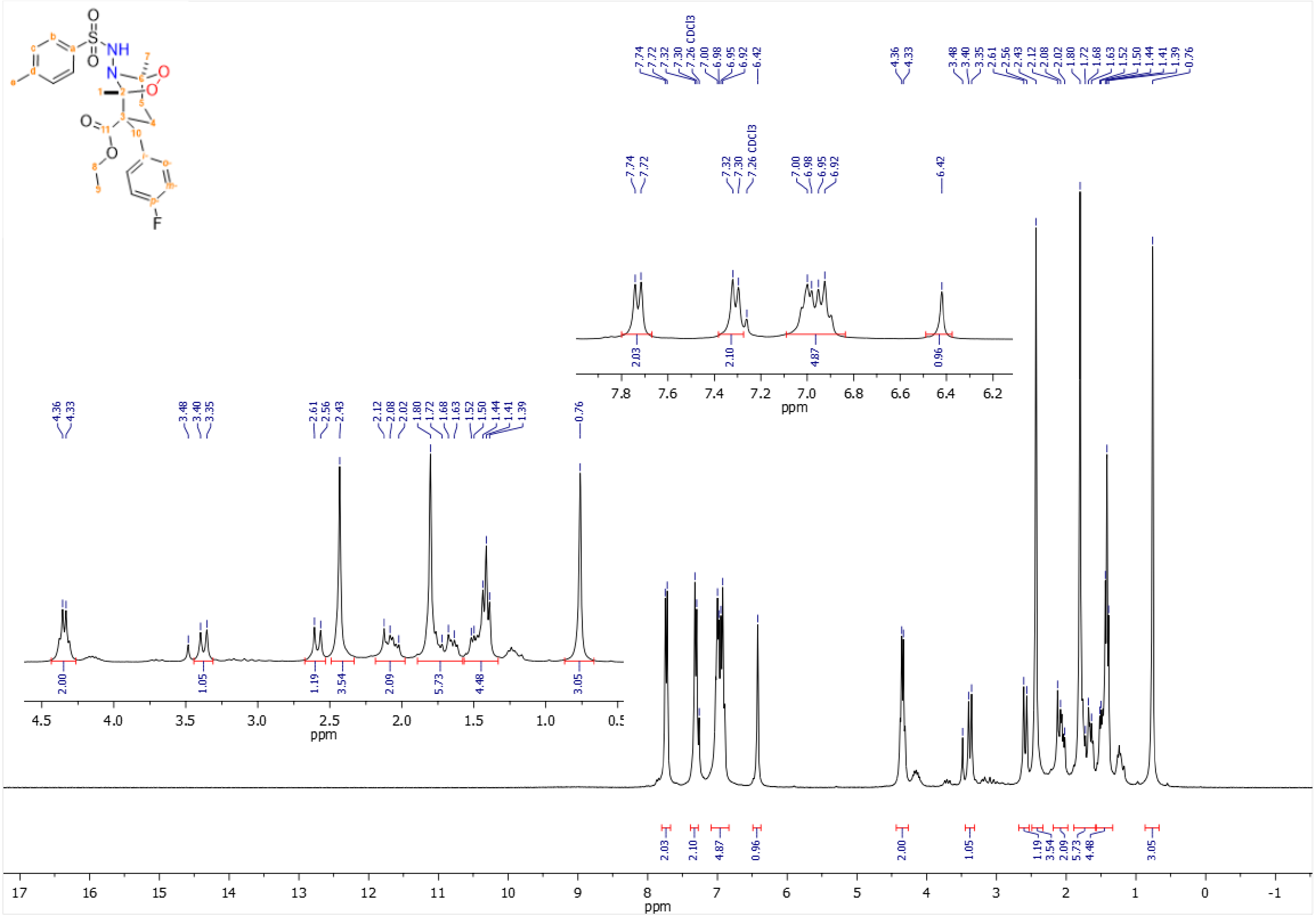

^{1}H NMR (300.13 MHz, $CDCl_3$). Ethyl 2-(4-fluorobenzyl)-1,5-dimethyl-8-((4-methylphenyl)sulfonamido)-6,7-dioxa-8-azabicyclo[3.2.1]octane-2-carboxylate, 30

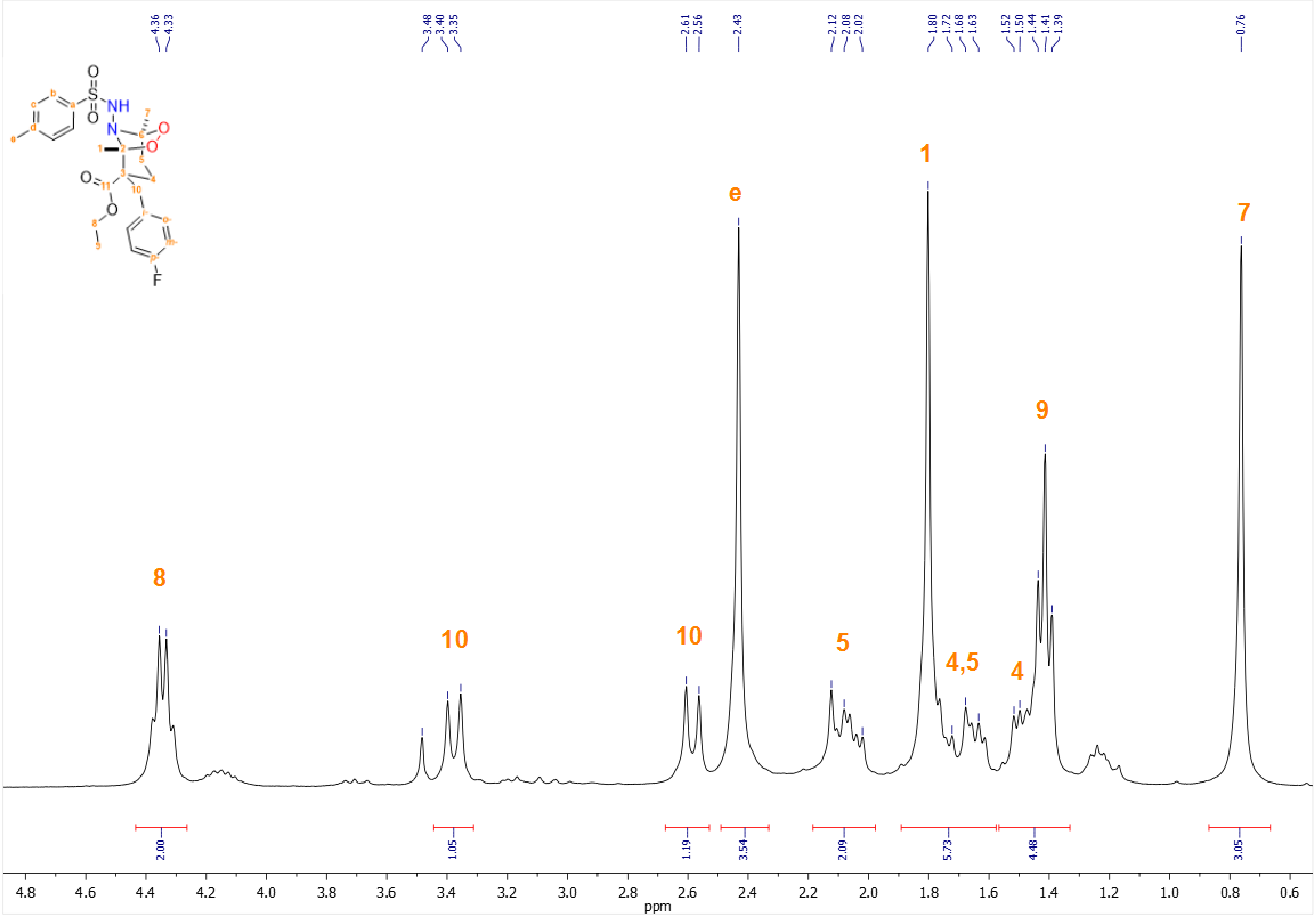

^{1}H NMR (300.13 MHz, $CDCl_3$). Ethyl 2-(4-fluorobenzyl)-1,5-dimethyl-8-((4-methylphenyl)sulfonamido)-6,7-dioxa-8-azabicyclo[3.2.1]octane-2-carboxylate, 30

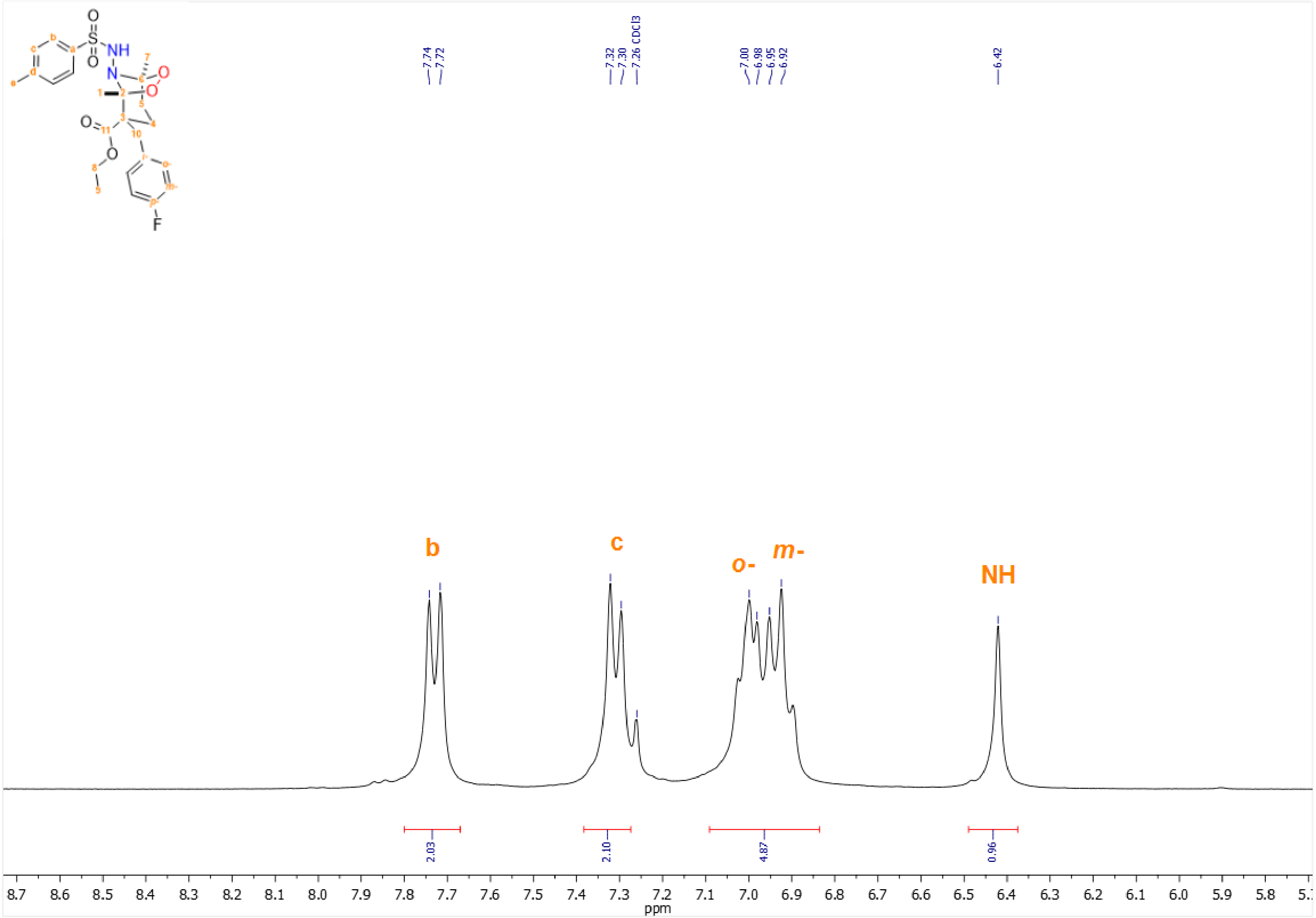

^{13}C NMR (75.48 MHz, $CDCl_3$). Ethyl 2-(4-fluorobenzyl)-1,5-dimethyl-8-((4-methylphenyl)sulfonamido)-6,7-dioxa-8-azabicyclo[3.2.1]octane-2-carboxylate, 30

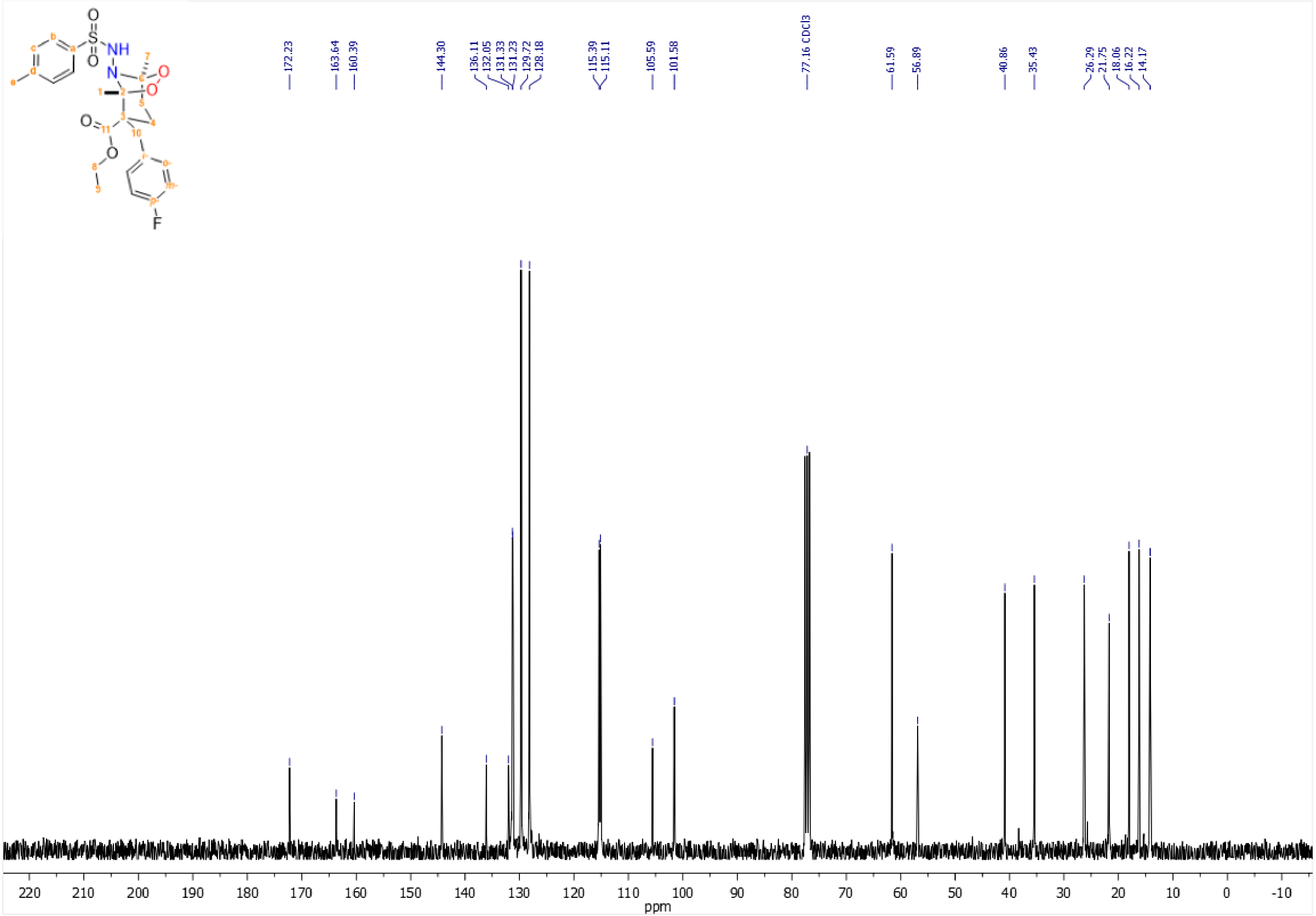

^{13}C NMR (75.48 MHz, $CDCl_3$). Ethyl 2-(4-fluorobenzyl)-1,5-dimethyl-8-((4-methylphenyl)sulfonamido)-6,7-dioxa-8-azabicyclo[3.2.1]octane-2-carboxylate, 30

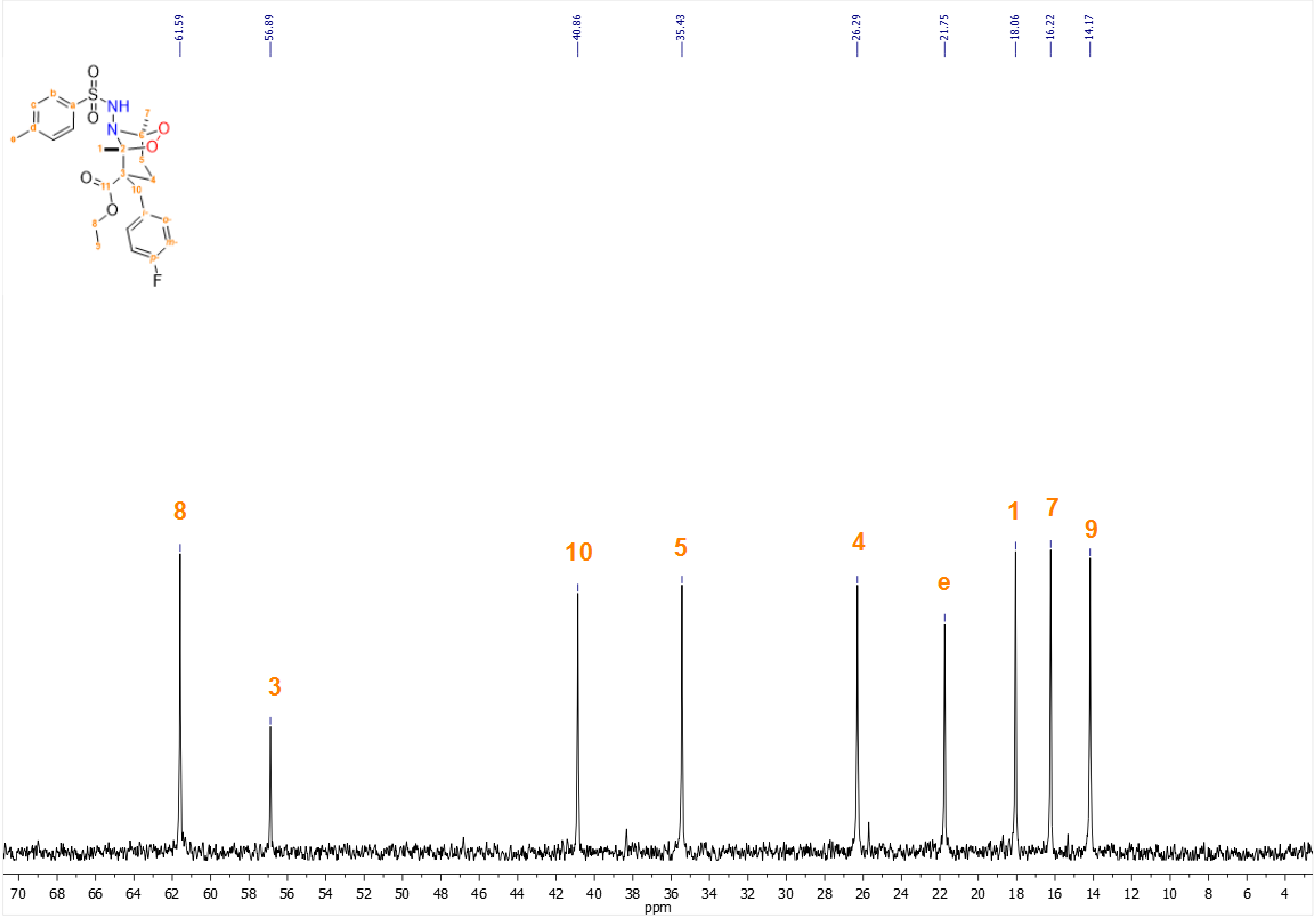

^{13}C NMR (75.48 MHz, $CDCl_3$). Ethyl 2-(4-fluorobenzyl)-1,5-dimethyl-8-((4-methylphenyl)sulfonamido)-6,7-dioxa-8-azabicyclo[3.2.1]octane-2-carboxylate, 30

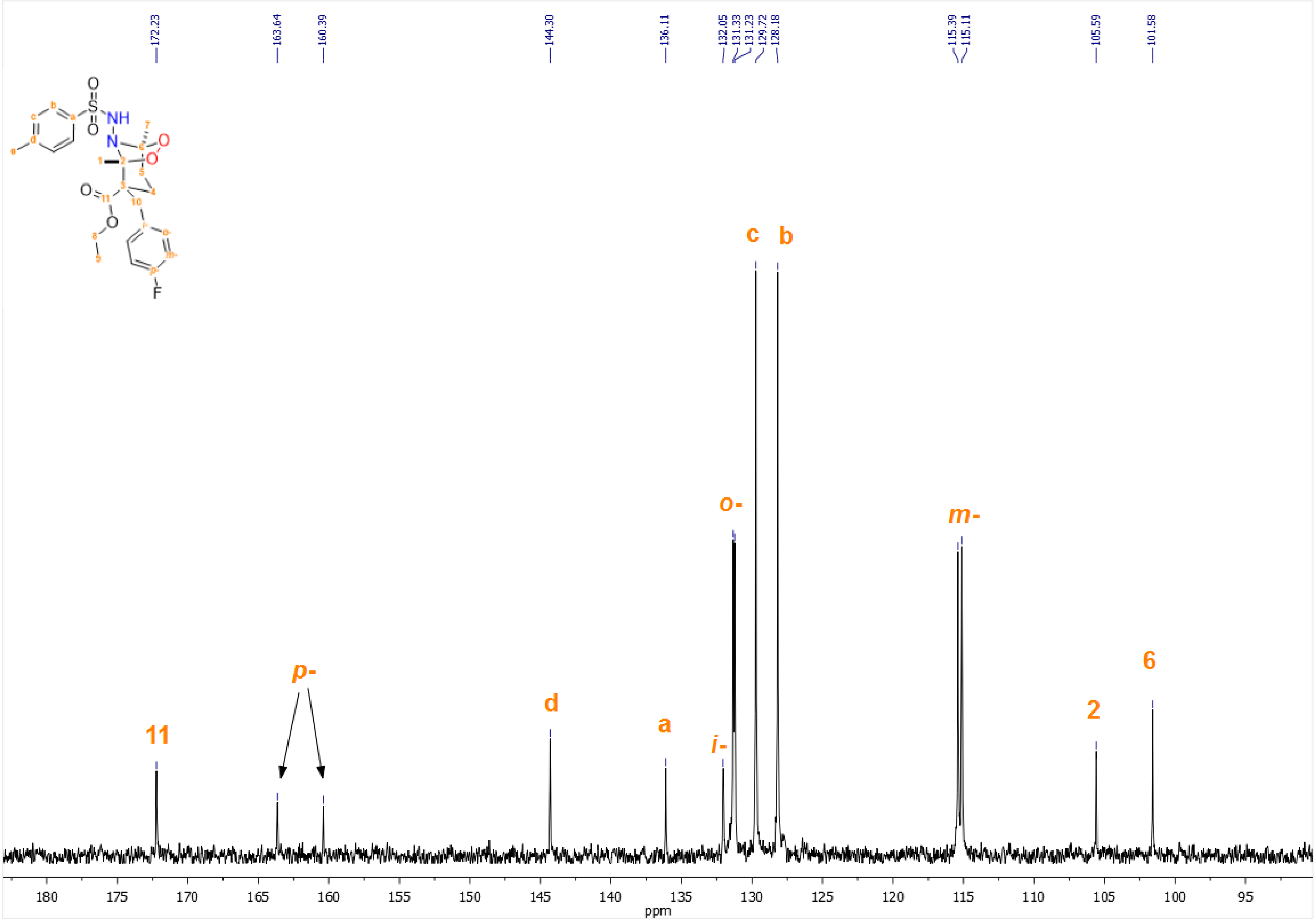

^{15}N (40.56 MHz, CDCl3). Ethyl 2-(4-fluorobenzyl)-1,5-dimethyl-8-((4-methylphenyl)sulfonamido)-6,7-dioxa-8-azabicyclo[3.2.1]octane-2-carboxylate, 30

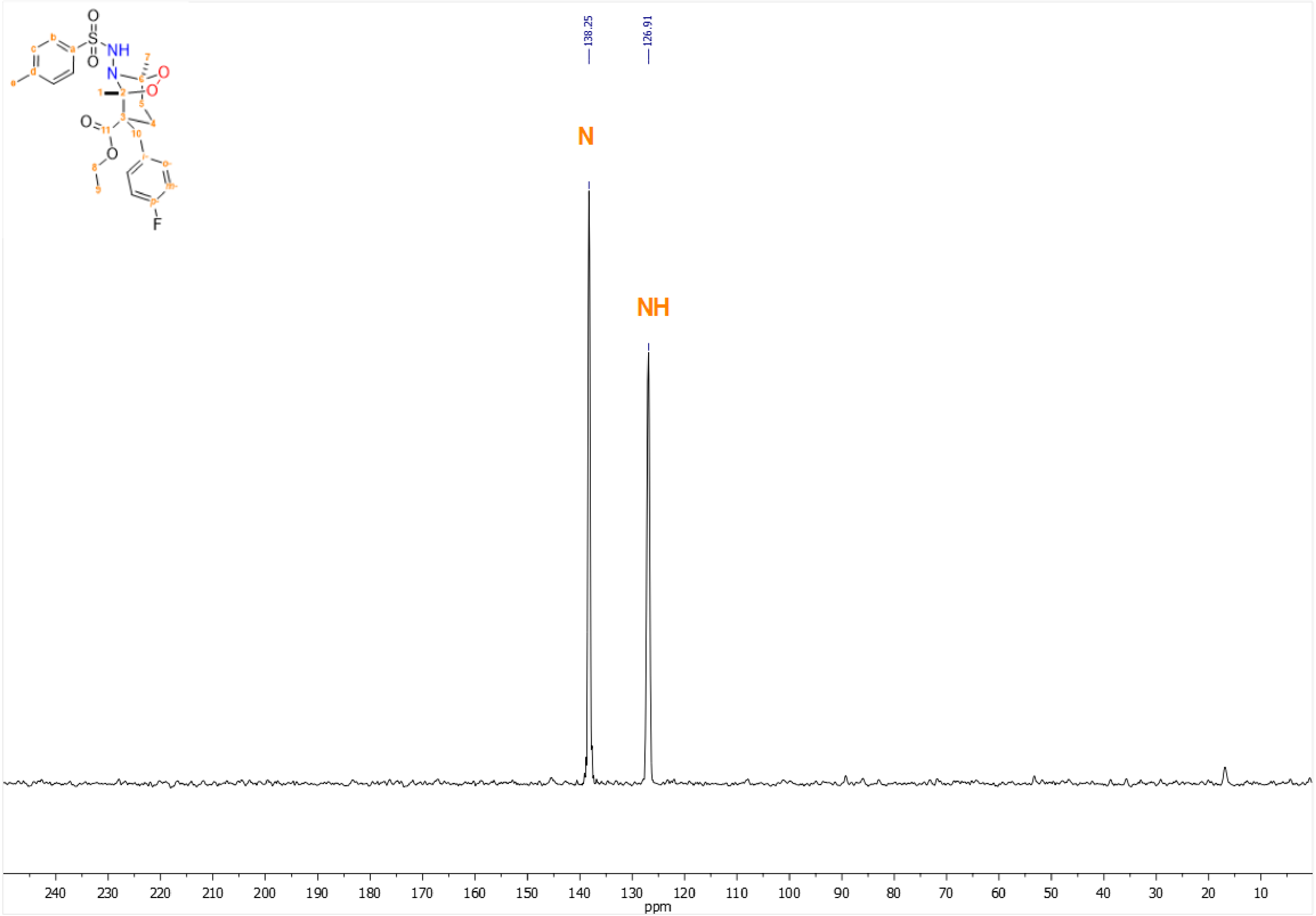

^{13}C NMR (75.48 MHz, $CDCl_3$). Ethyl 2-(4-fluorobenzyl)-1,5-dimethyl-8-((4-methylphenyl)sulfonamido)-6,7-dioxa-8-azabicyclo[3.2.1]octane-2-carboxylate, 30

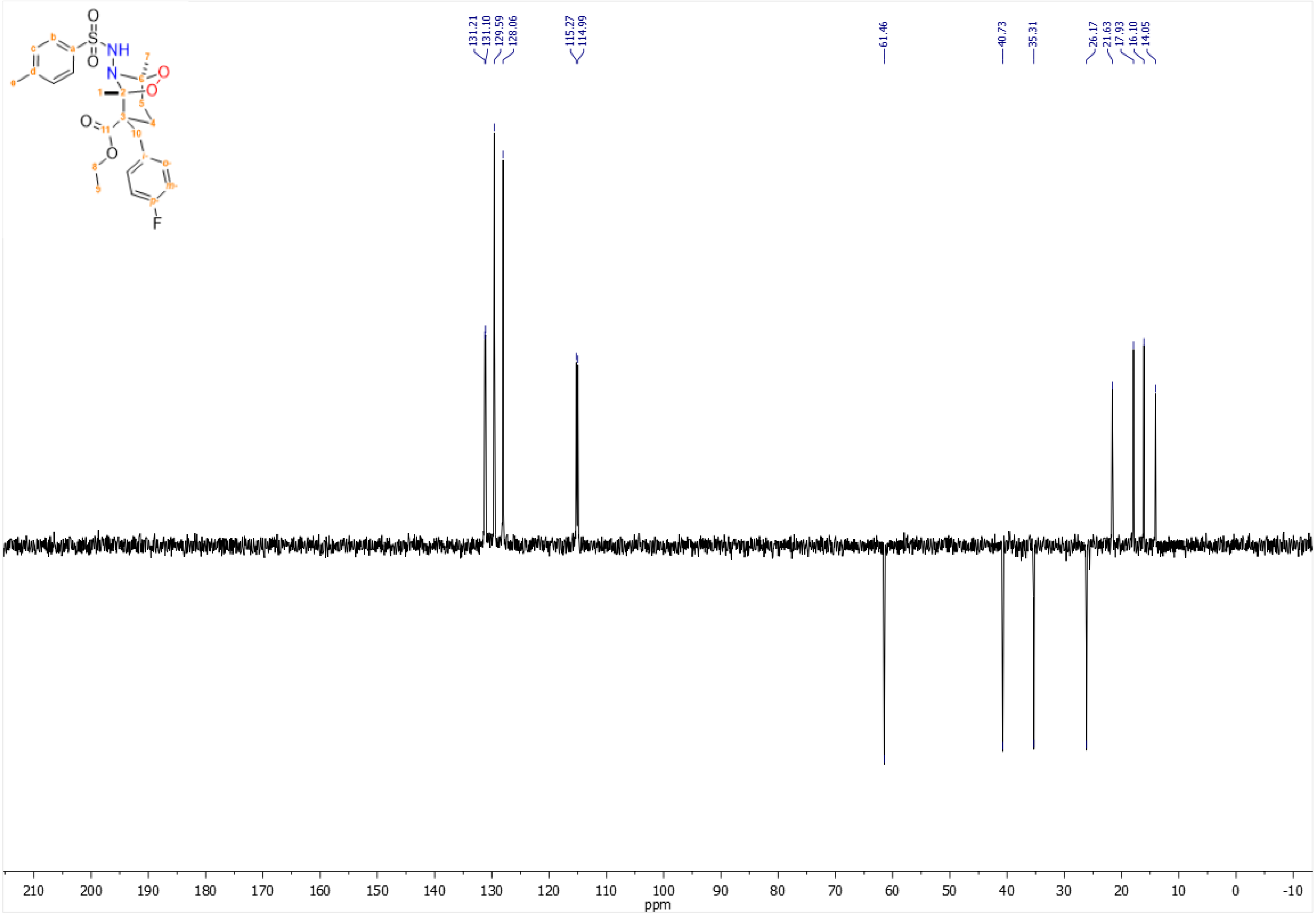

Ethyl 2-(4-fluorobenzyl)-1,5-dimethyl-8-((4-methylphenyl)sulfonamido)-6,7-dioxa-8-azabicyclo[3.2.1]octane-2-carboxylate, 30

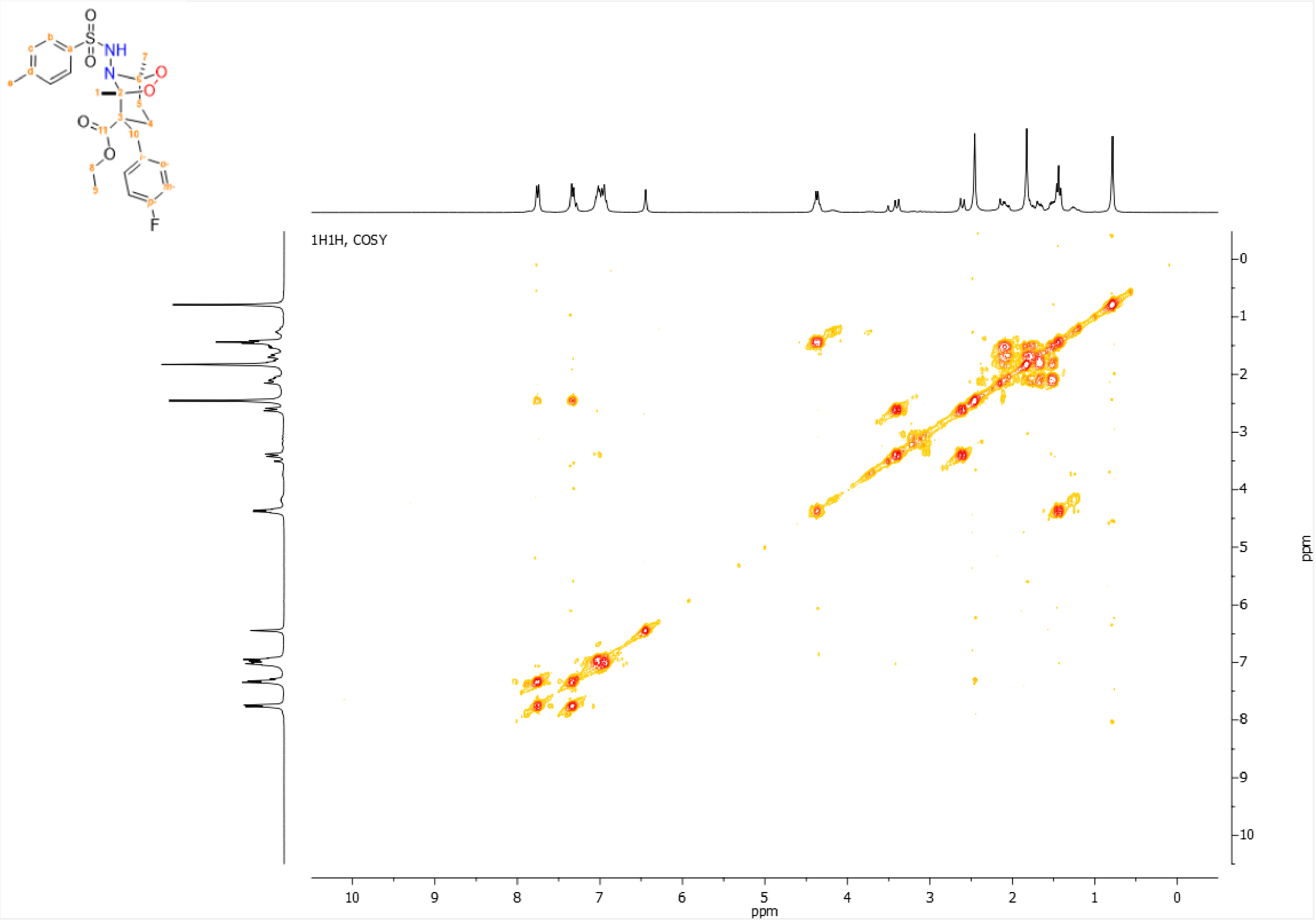

Ethyl 2-(4-fluorobenzyl)-1,5-dimethyl-8-((4-methylphenyl)sulfonamido)-6,7-dioxa-8-azabicyclo[3.2.1]octane-2-carboxylate, 30

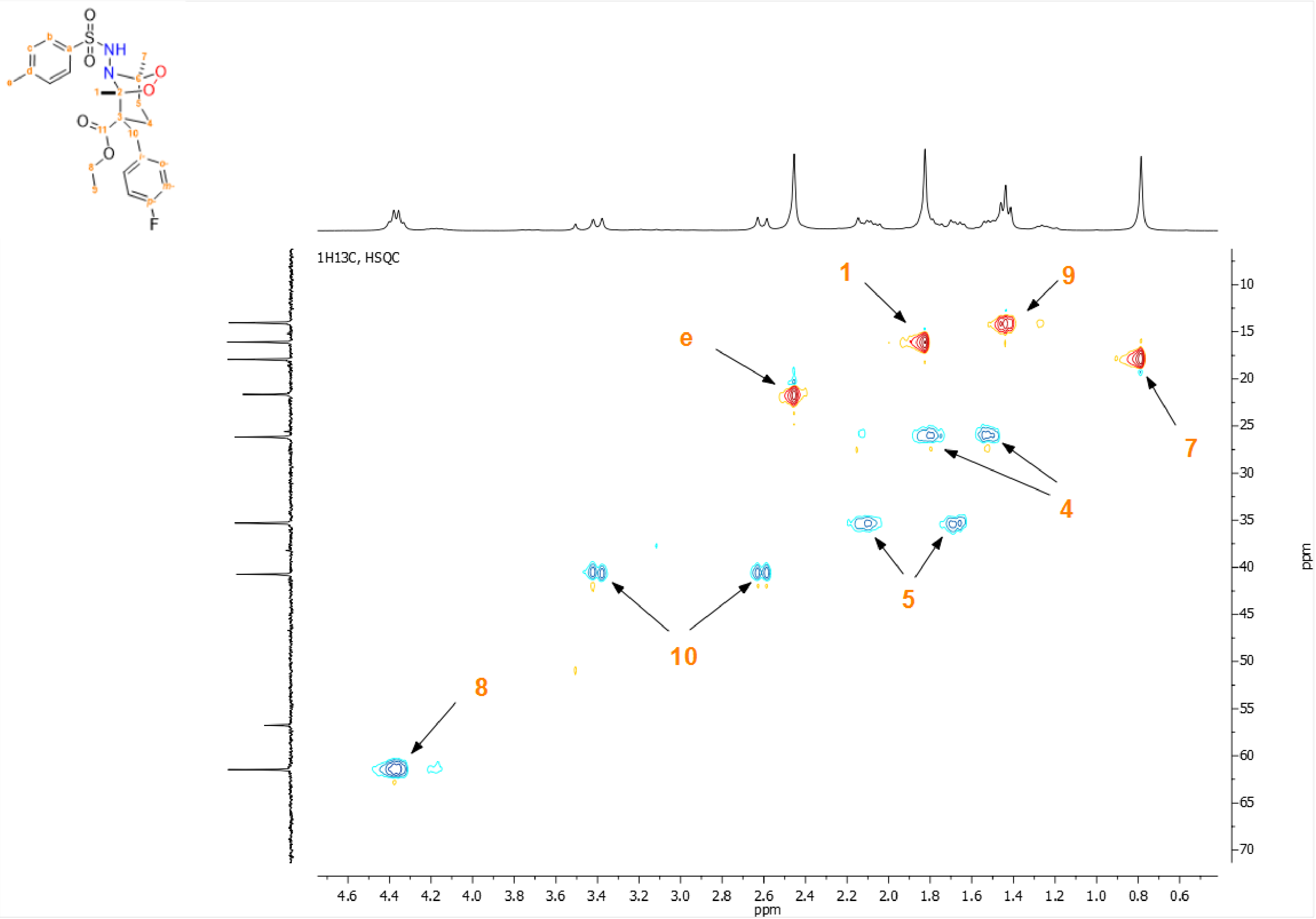

Ethyl 2-(4-fluorobenzyl)-1,5-dimethyl-8-((4-methylphenyl)sulfonamido)-6,7-dioxa-8-azabicyclo[3.2.1]octane-2-carboxylate, 30

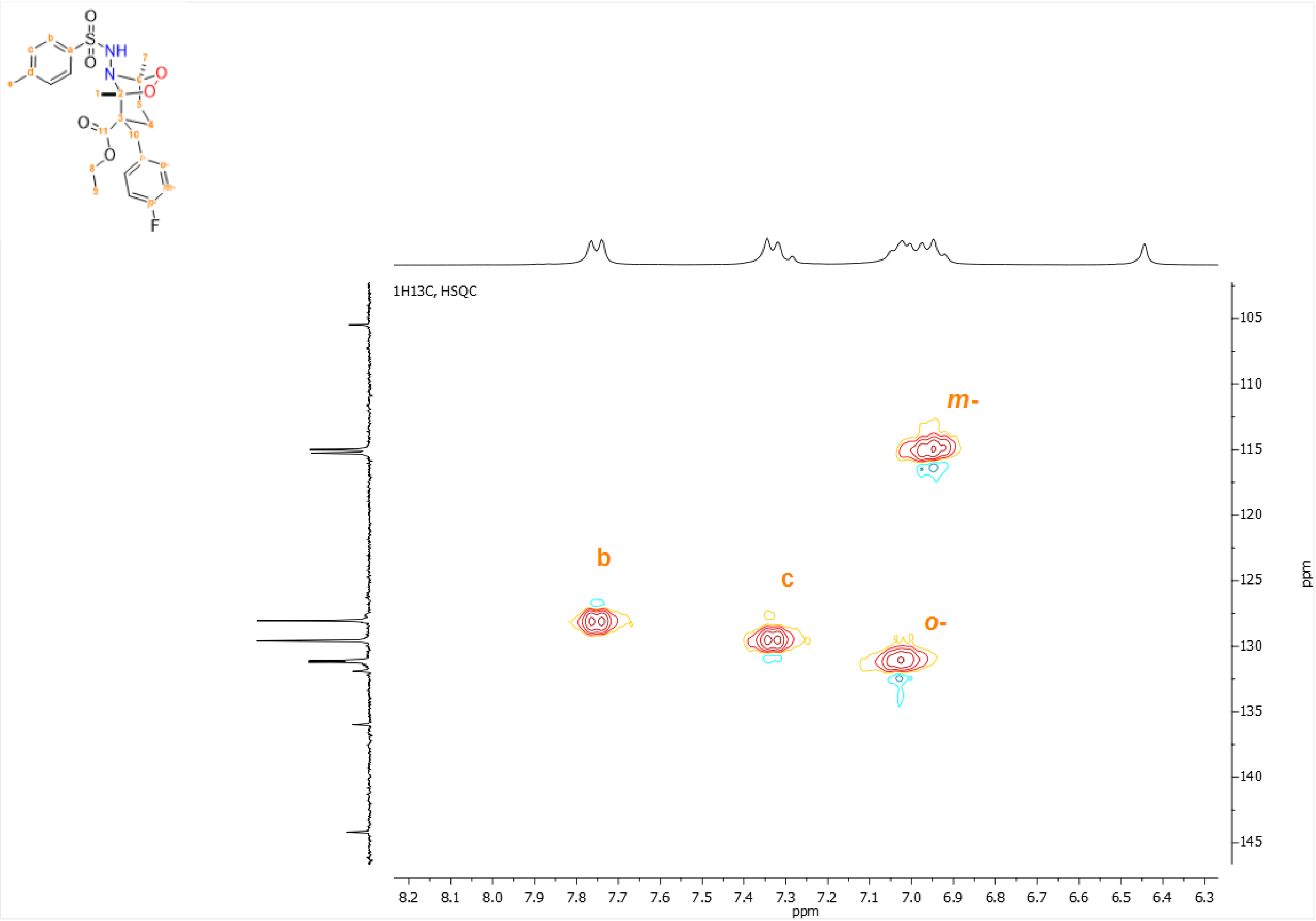

Ethyl 2-(4-fluorobenzyl)-1,5-dimethyl-8-((4-methylphenyl)sulfonamido)-6,7-dioxa-8-azabicyclo[3.2.1]octane-2-carboxylate, 30

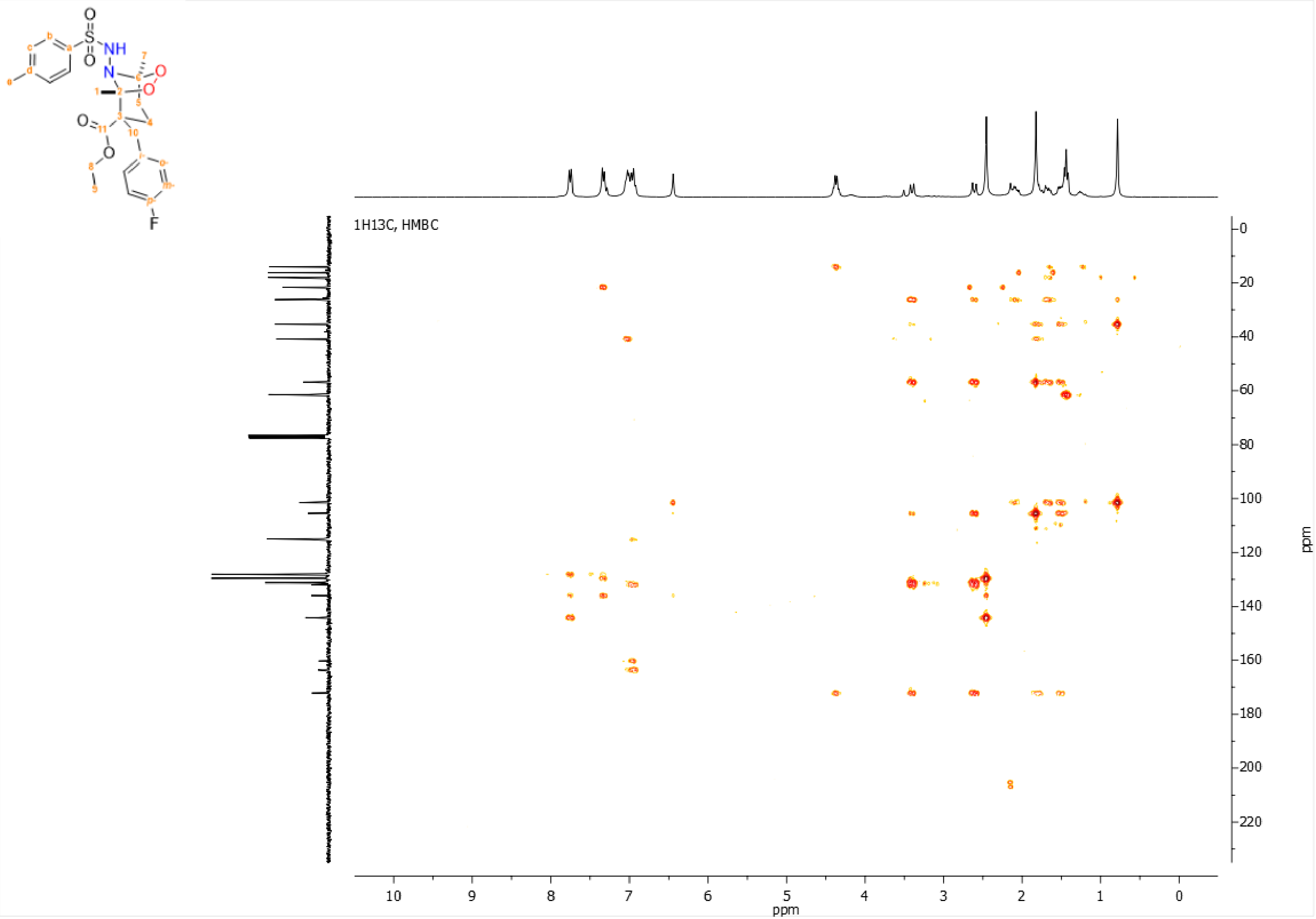

Ethyl 2-(4-fluorobenzyl)-1,5-dimethyl-8-((4-methylphenyl)sulfonamido)-6,7-dioxa-8-azabicyclo[3.2.1]octane-2-carboxylate, 30

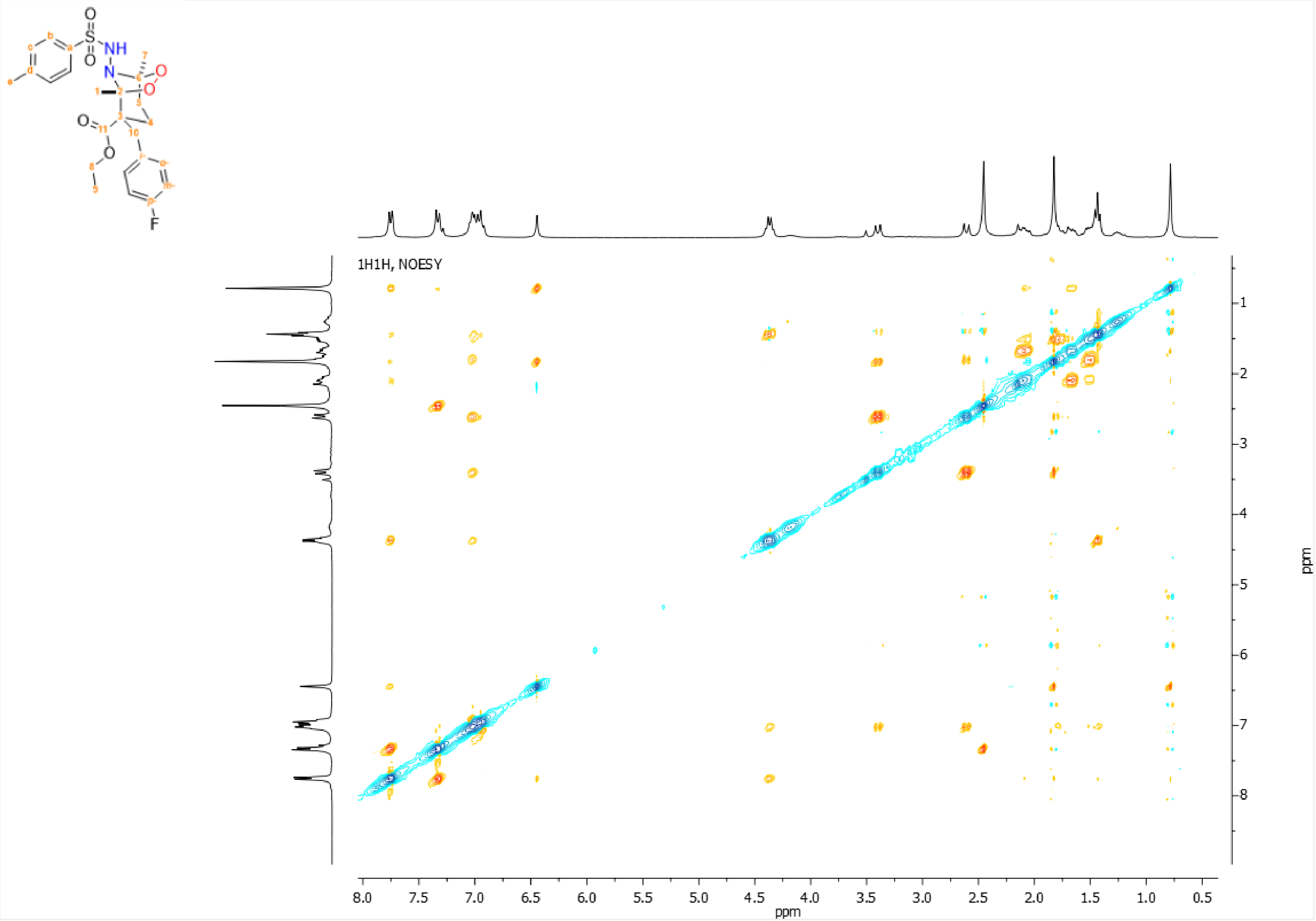

Ethyl 2-(4-fluorobenzyl)-1,5-dimethyl-8-((4-methylphenyl)sulfonamido)-6,7-dioxa-8-azabicyclo[3.2.1]octane-2-carboxylate, 30

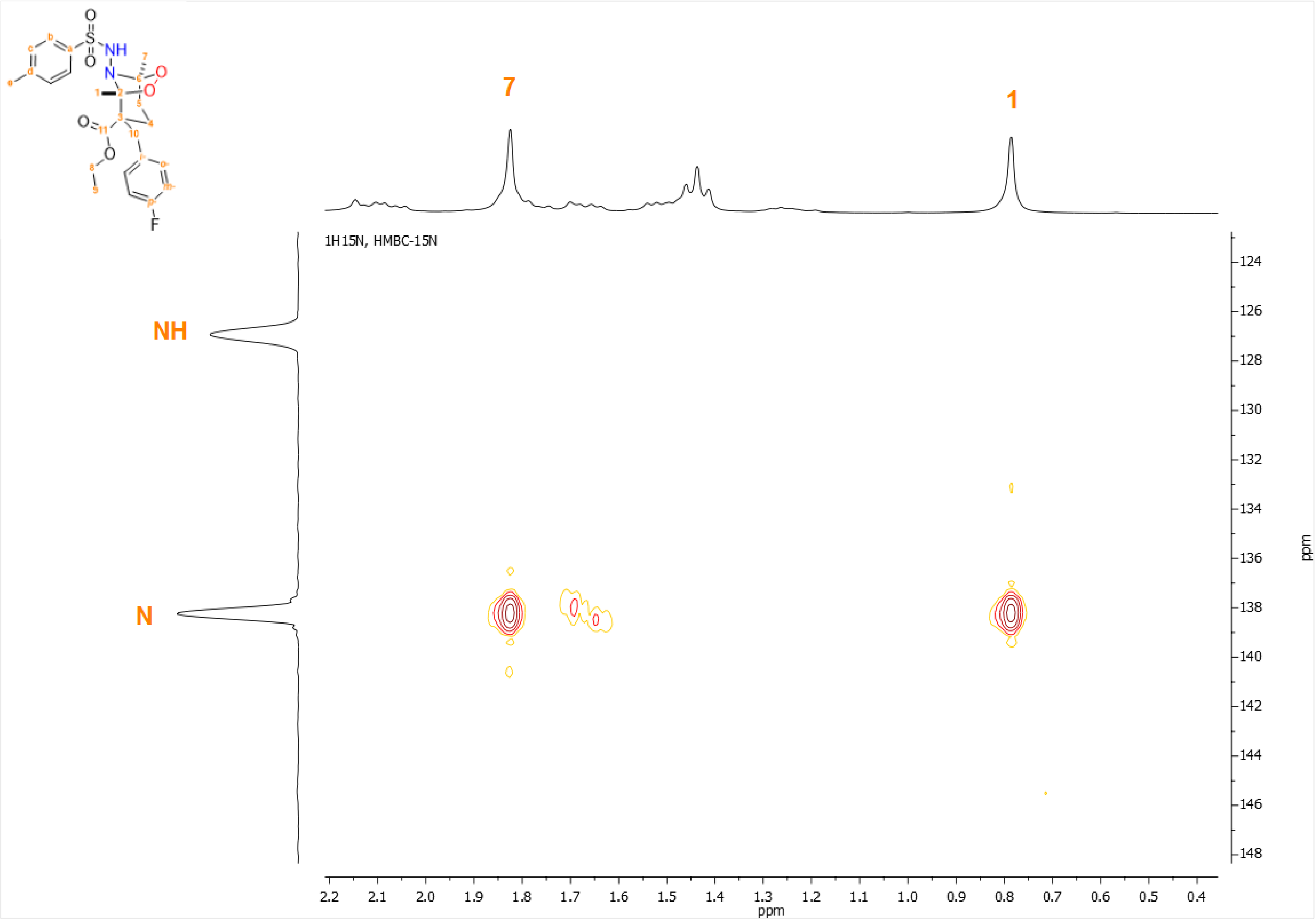

Ethyl 2-(4-fluorobenzyl)-1,5-dimethyl-8-((4-methylphenyl)sulfonamido)-6,7-dioxa-8-azabicyclo[3.2.1]octane-2-carboxylate, 30

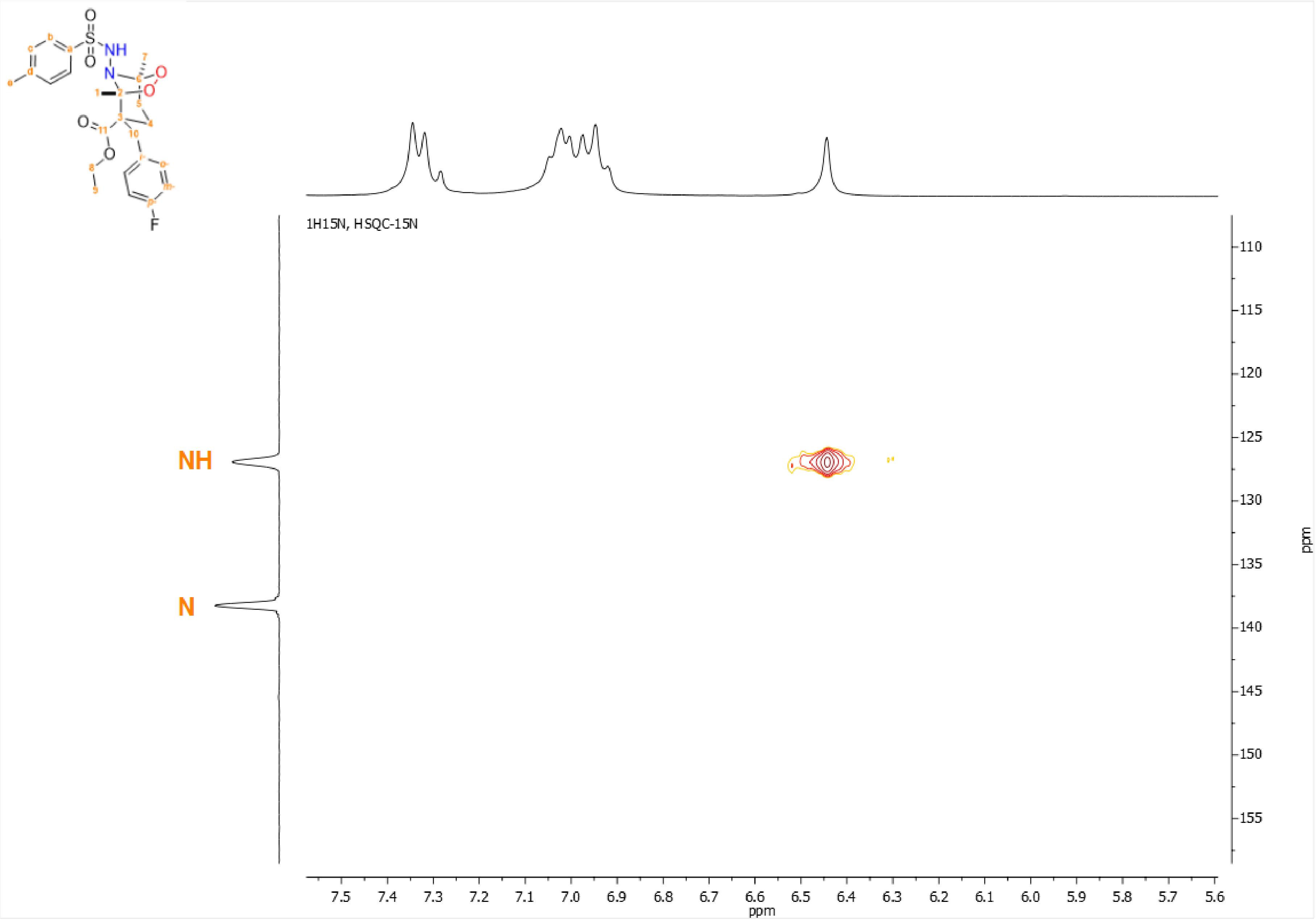

^{1}H NMR (300.13 MHz, CDCl$_3$). Ethyl 8-((3r,5r,7r)-adamantane-1-carboxamido)-2-(4-chlorobenzyl)-1,5-dimethyl-6,7-dioxa-8-azabicyclo[3.2.1]octane-2-carboxylate, 31a + 31b

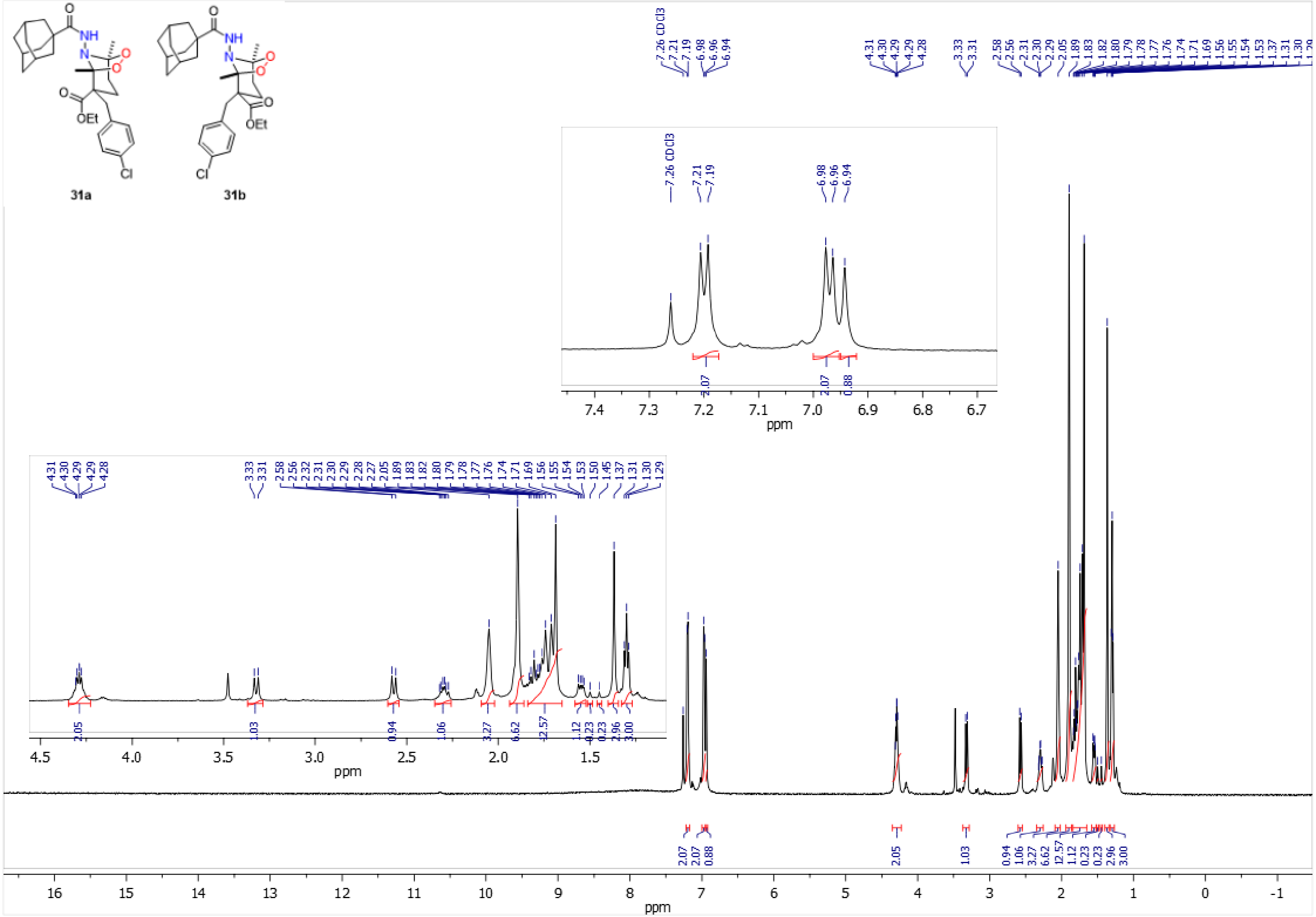

^{13}C NMR (75.48 MHz, CDCl$_3$). Ethyl 8-((3r,5r,7r)-adamantane-1-carboxamido)-2-(4-chlorobenzyl)-1,5-dimethyl-6,7-dioxa-8-azabicyclo[3.2.1]octane-2-carboxylate, 31a + 31b

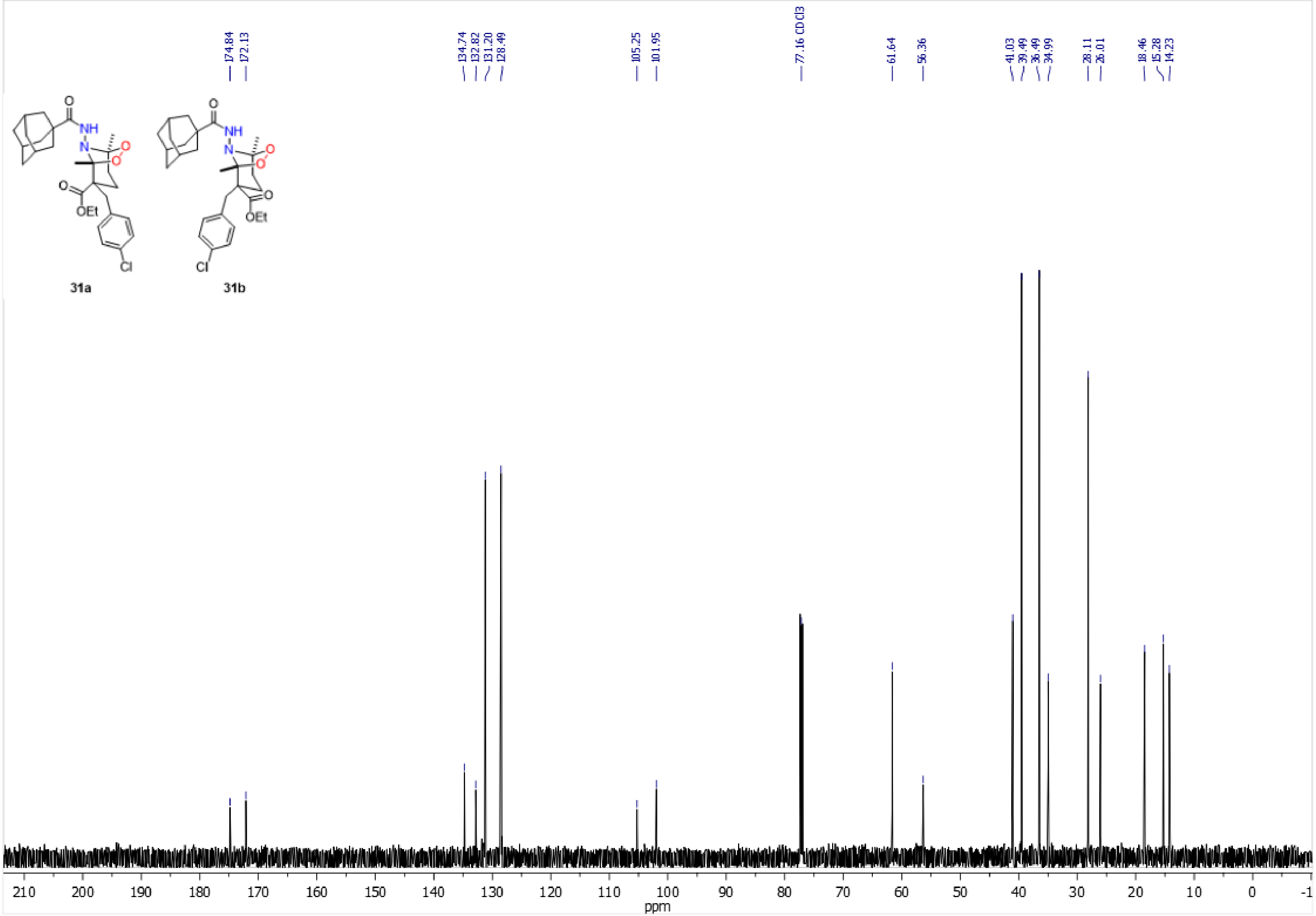

^{1}H NMR (300.13 MHz, CDCl3). 3a-Allyl-3,6,7a-trimethylhexahydro-3H-3,6-epoxy[1,2]dioxolo[3,4-b]pyridine, 32

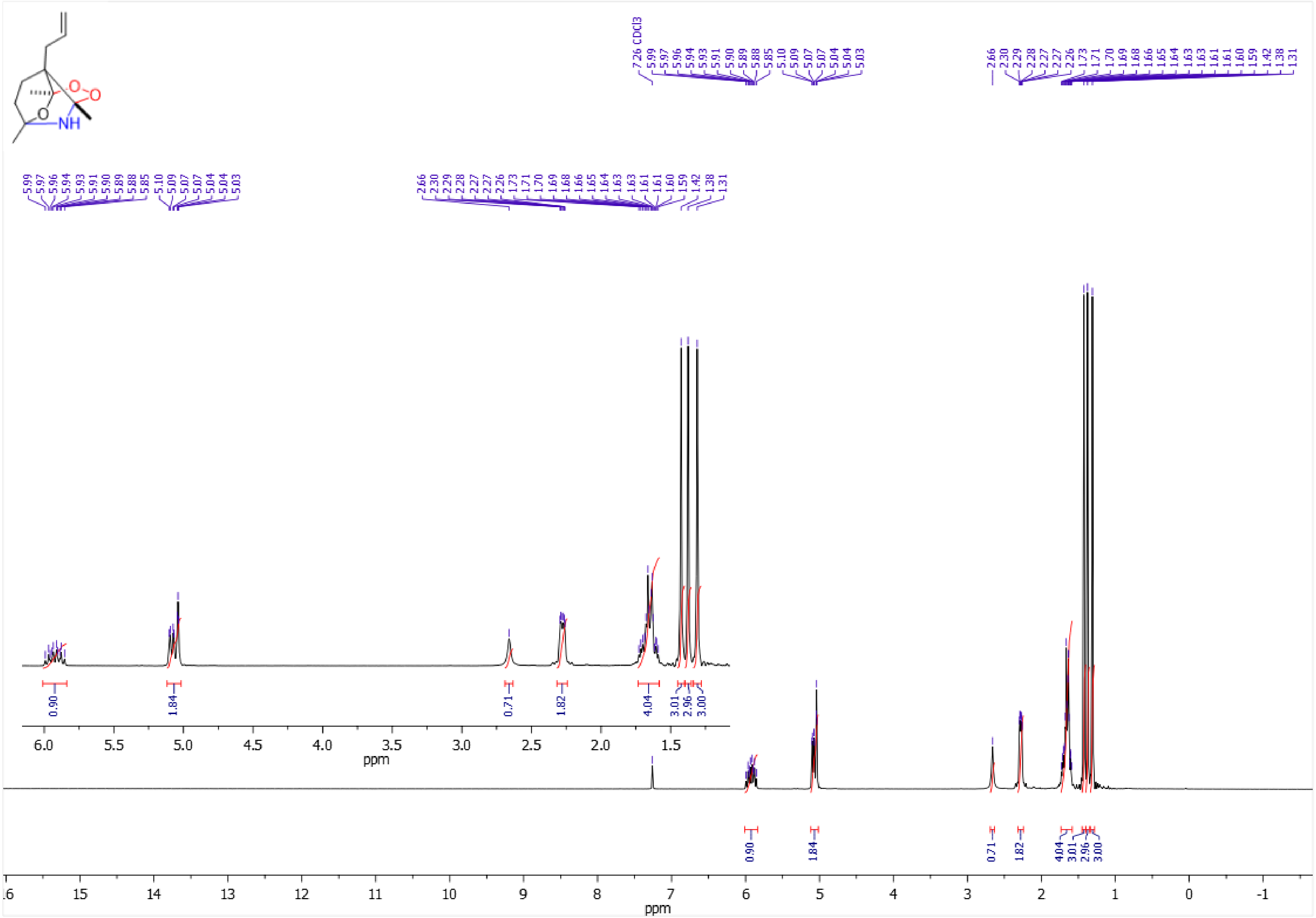

^{13}C NMR (75.48 MHz, CDCl₃). 3a-Allyl-3,6,7a-trimethylhexahydro-3H-3,6-epoxy[1,2]dioxolo[3,4-b]pyridine, 32

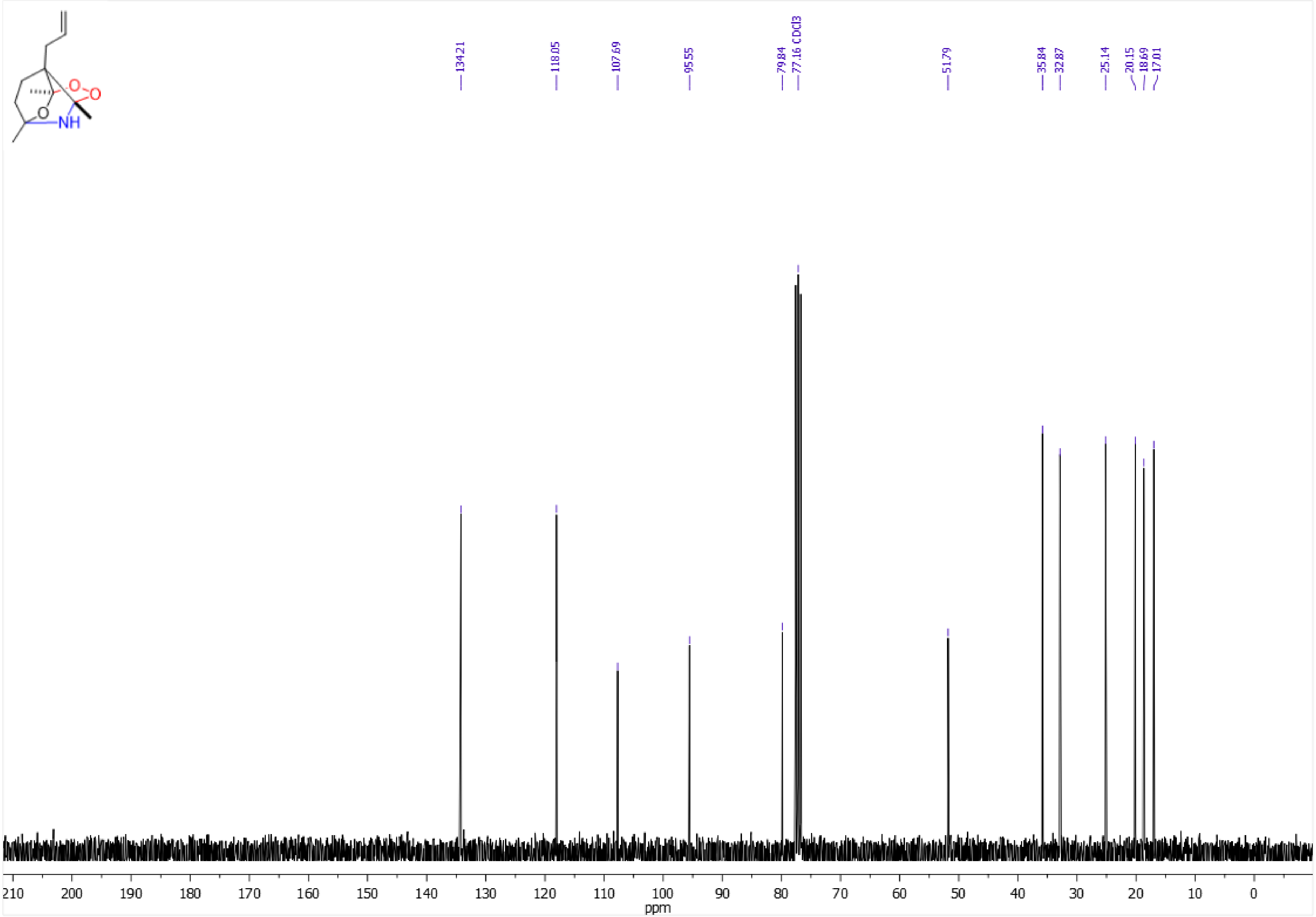

^{1}H NMR (300.13 MHz, $CDCl_3$). 3a-Hexyl-3,6,7a-trimethylhexahydro-3H-3,6-epoxy[1,2]dioxolo[3,4-b]pyridine, 33

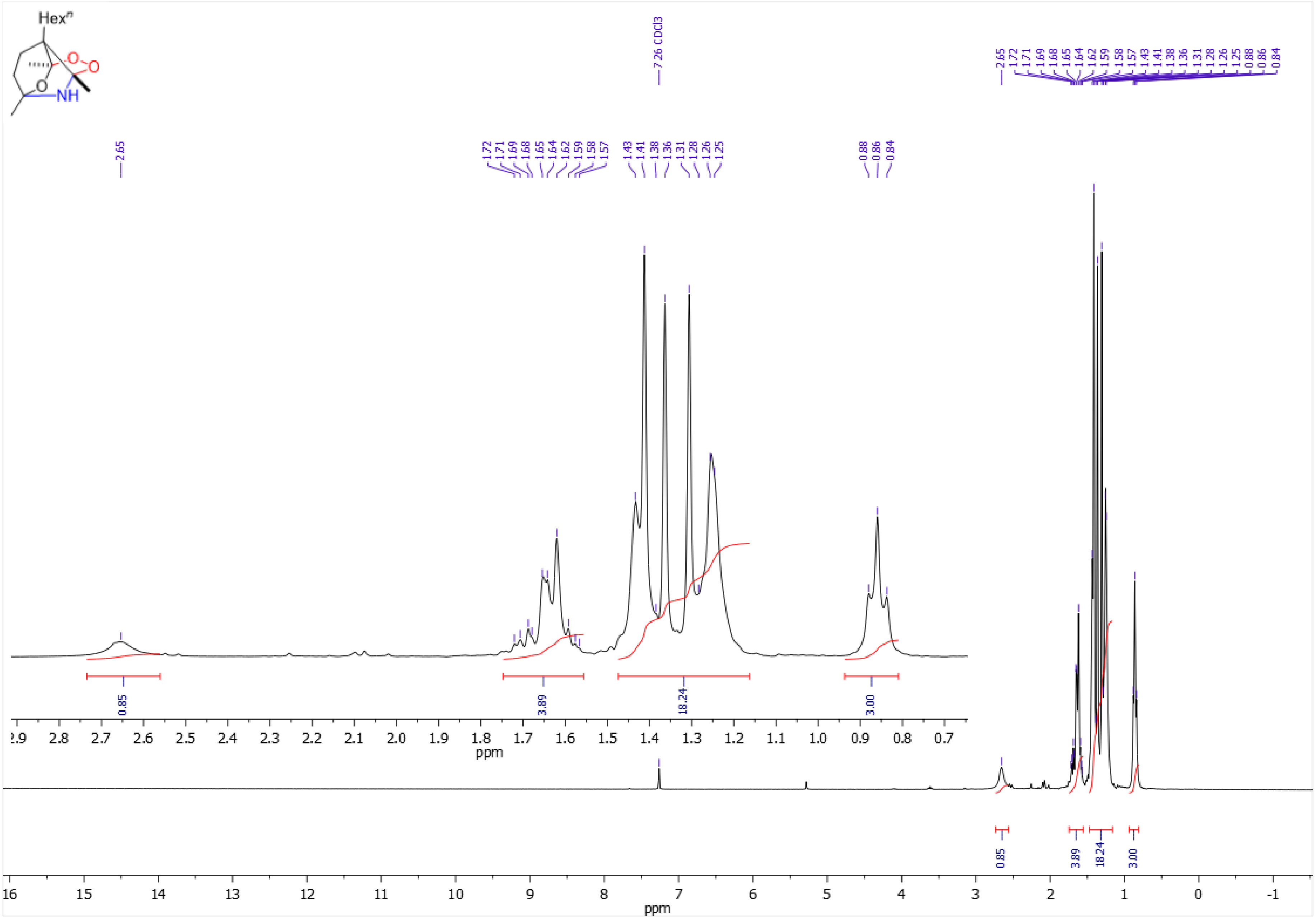

^{13}C NMR (75.48 MHz, $CDCl_3$). 3a-Hexyl-3,6,7a-trimethylhexahydro-3H-3,6-epoxy[1,2]dioxolo[3,4-b]pyridine, 33

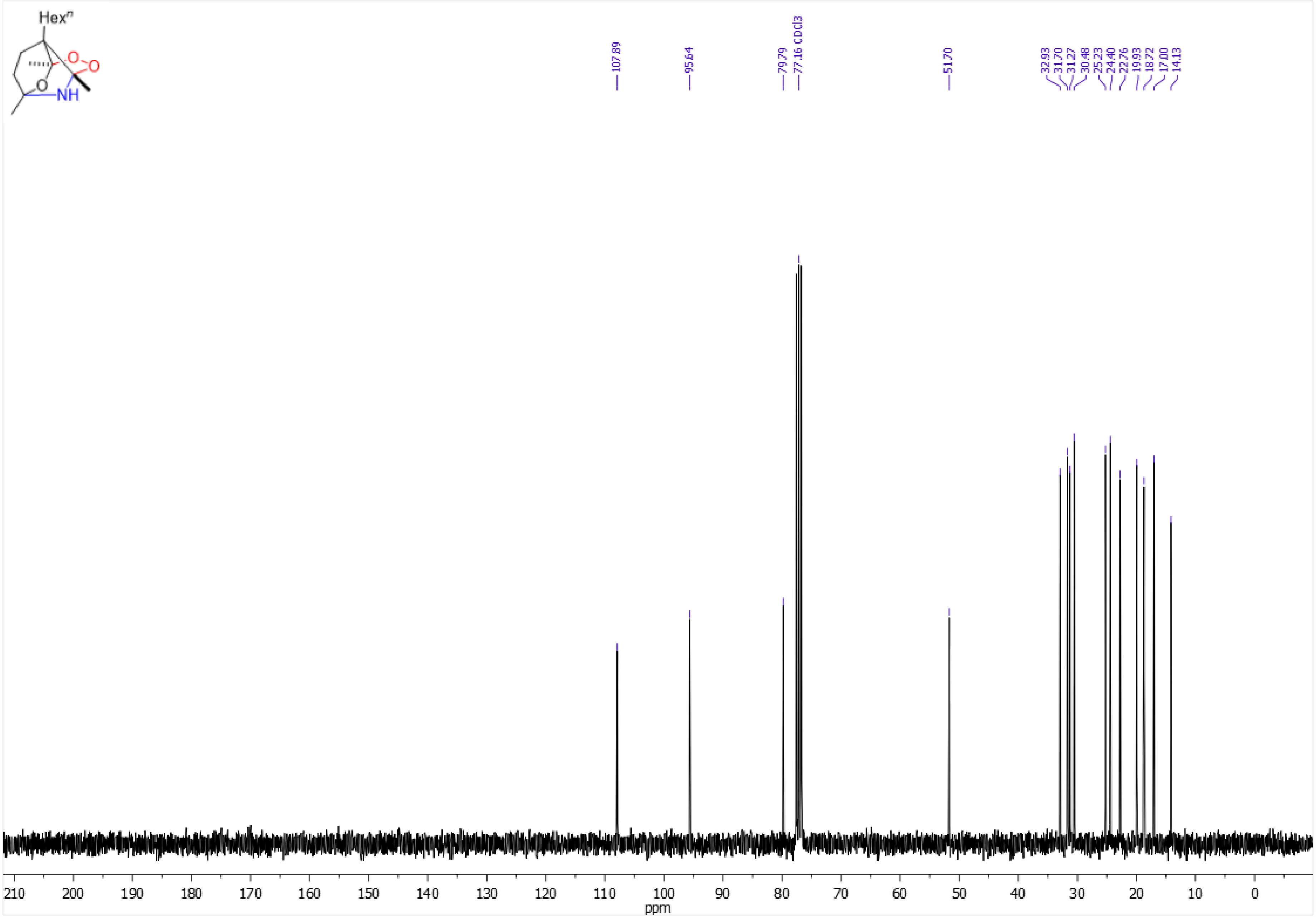

^{1}H NMR (300.13 MHz, $CDCl_3$). 3a-Isopentyl-3,6,7a-trimethylhexahydro-3H-3,6-epoxy[1,2]dioxolo[3,4-b]pyridine, 34

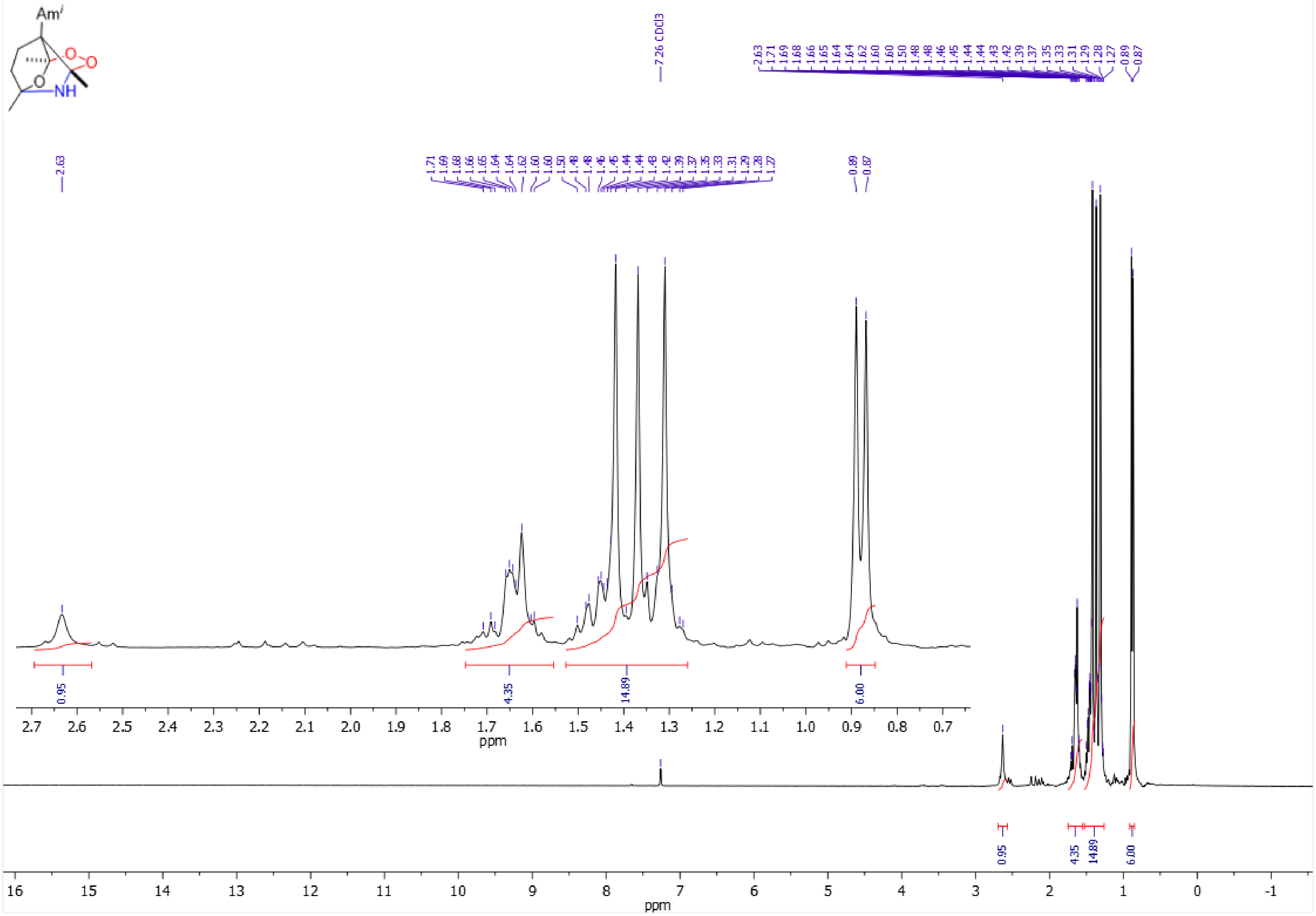

^{13}C NMR (75.48 MHz, $CDCl_3$). 3a-Isopentyl-3,6,7a-trimethylhexahydro-3H-3,6-epoxy[1,2]dioxolo[3,4-b]pyridine, 34

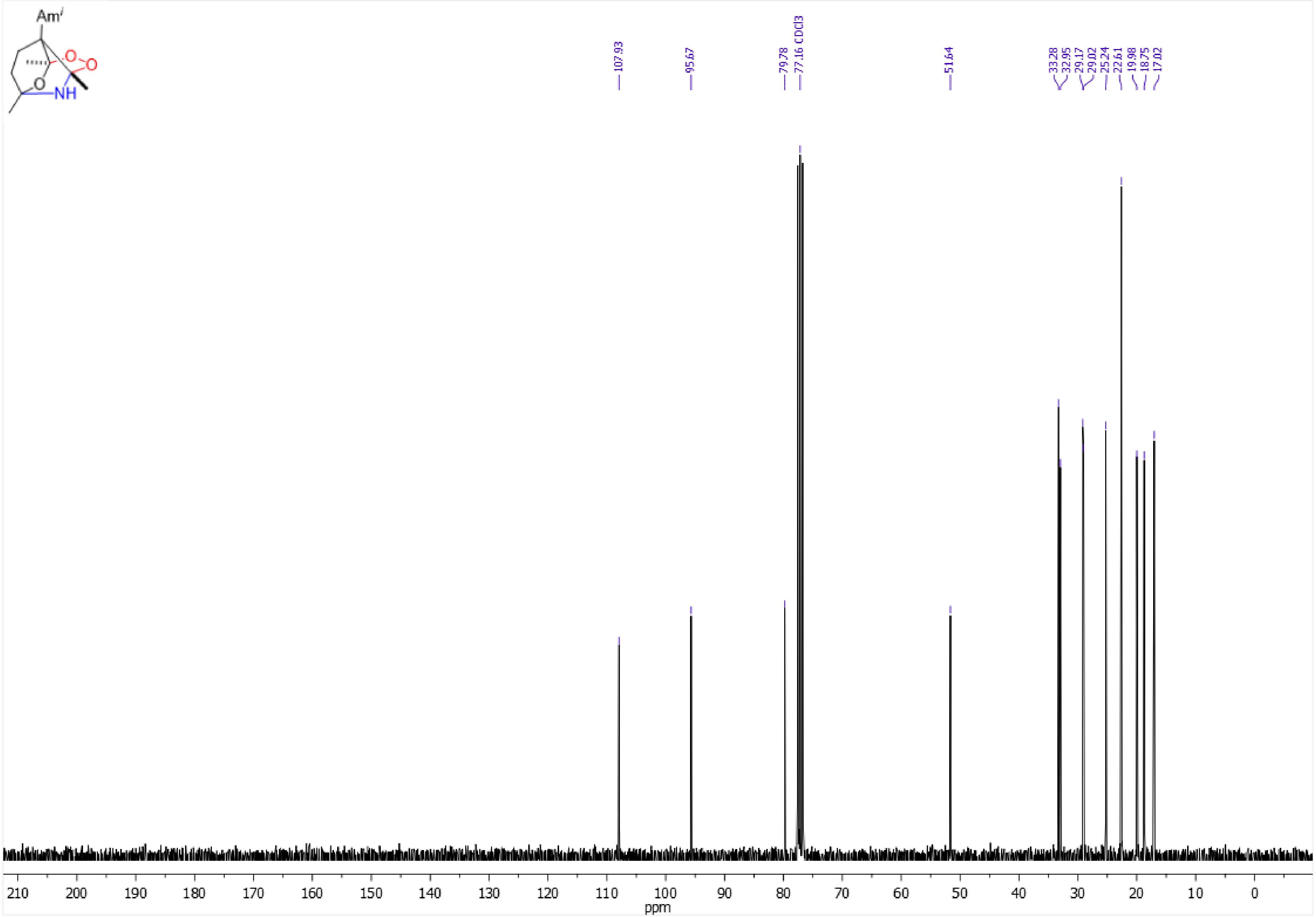

^{1}H NMR (300.13 MHz, $CDCl_3$). 3a-Butyl-3,6,7a-trimethylhexahydro-3H-3,6-epoxy[1,2]dioxolo[3,4-b]pyridine, 35

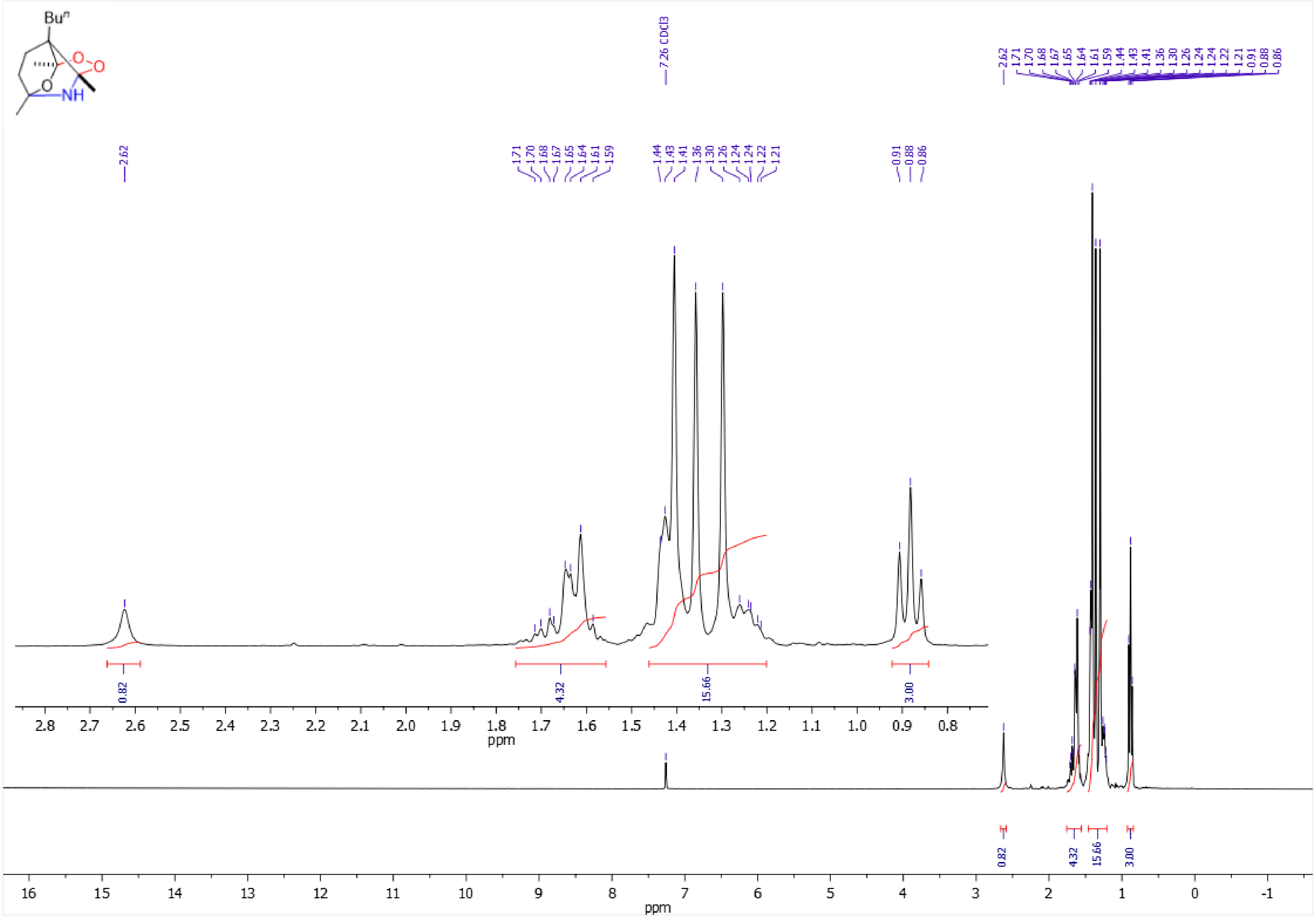

^{13}C NMR (75.48 MHz, CDCl₃). 3a-Butyl-3,6,7a-trimethylhexahydro-3H-3,6-epoxy[1,2]dioxolo[3,4-b]pyridine, 35

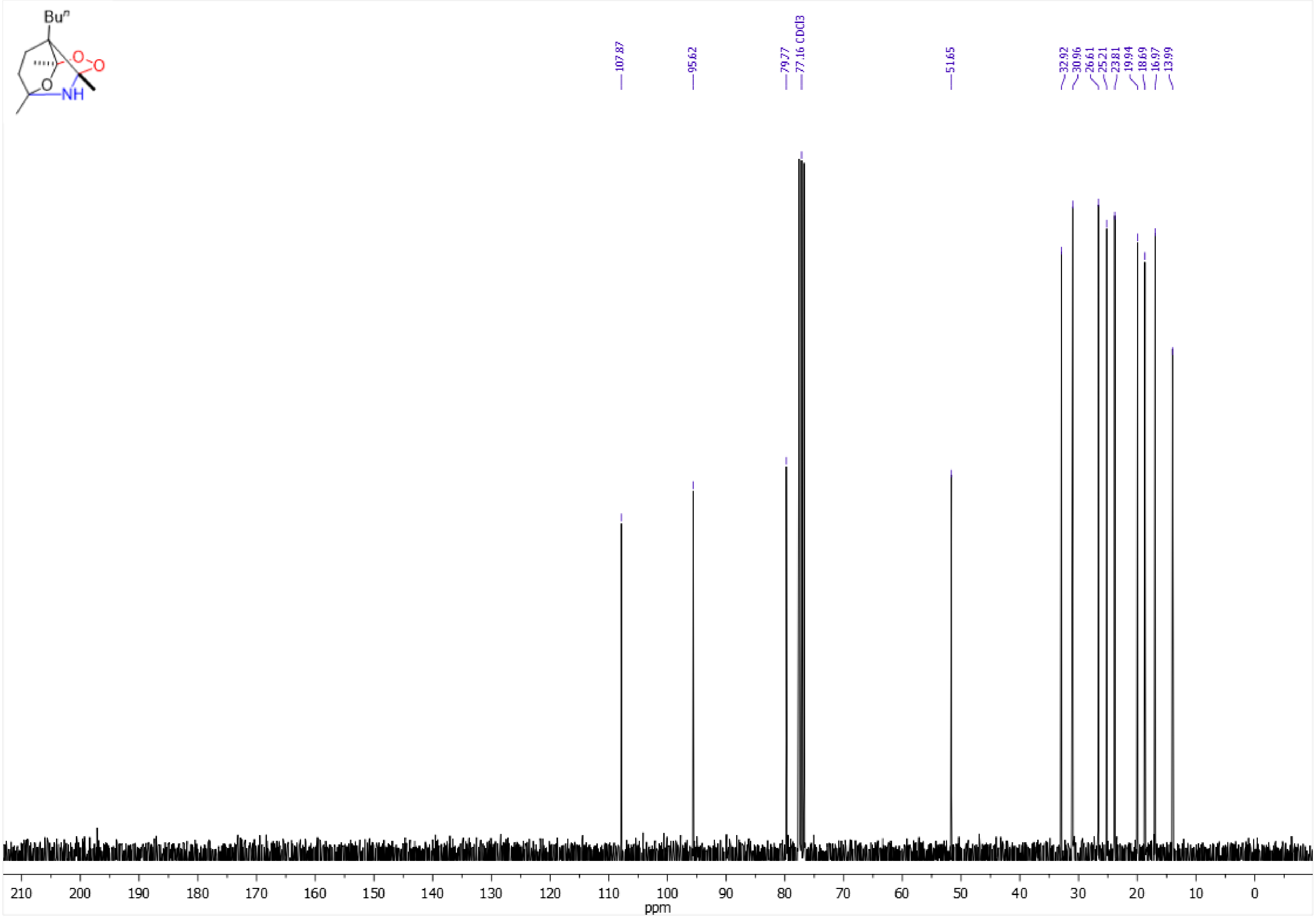

^{1}H NMR (300.13 MHz, $CDCl_3$). 3,3a,6,7a-Tetramethylhexahydro-3H-3,6-epoxy[1,2]dioxolo[3,4-b]pyridine, 36

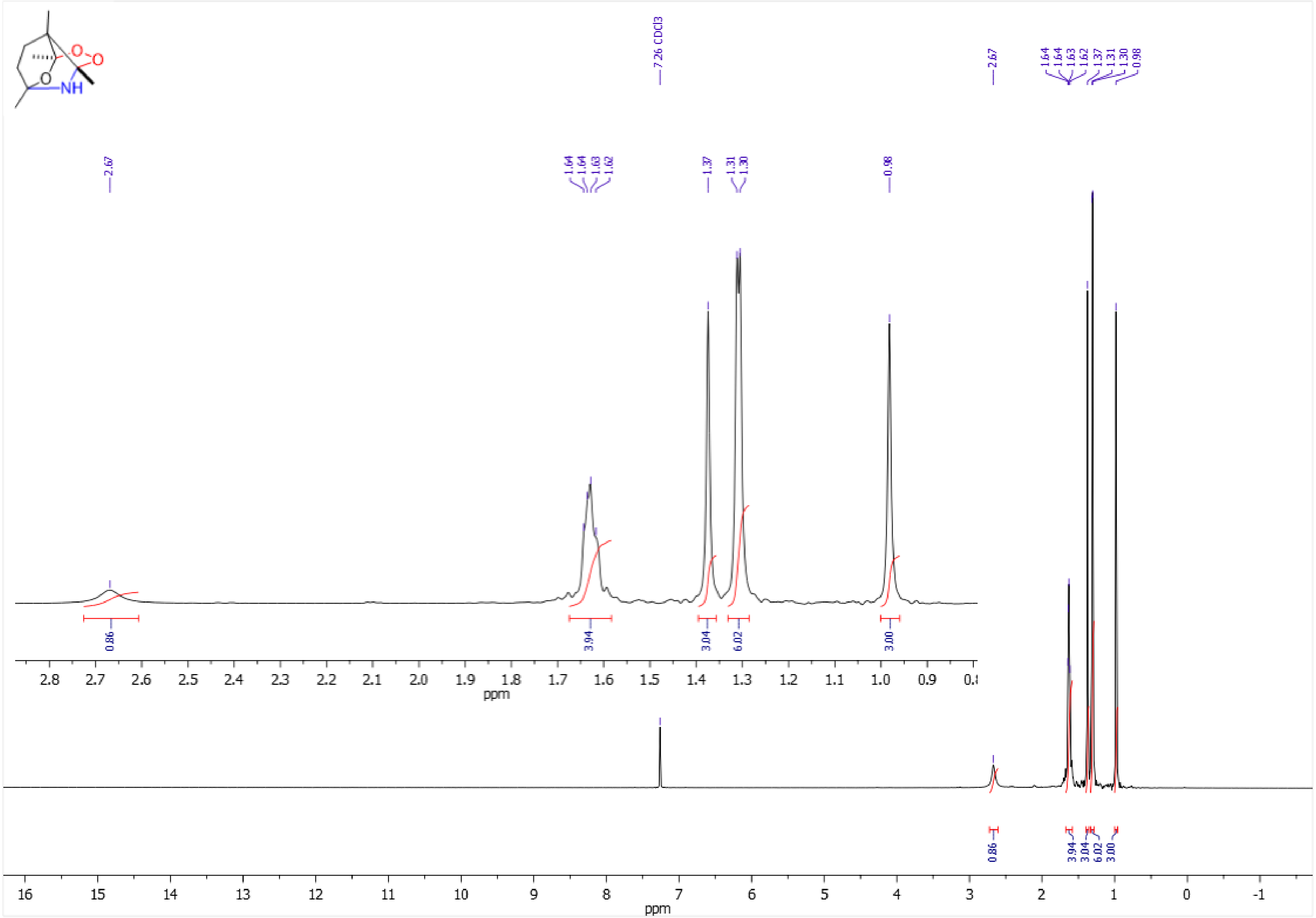

^{13}C NMR (75.48 MHz, CDCl$_3$). 3,3a,6,7a-Tetramethylhexahydro-3H-3,6-epoxy[1,2]dioxolo[3,4-b]pyridine, 36

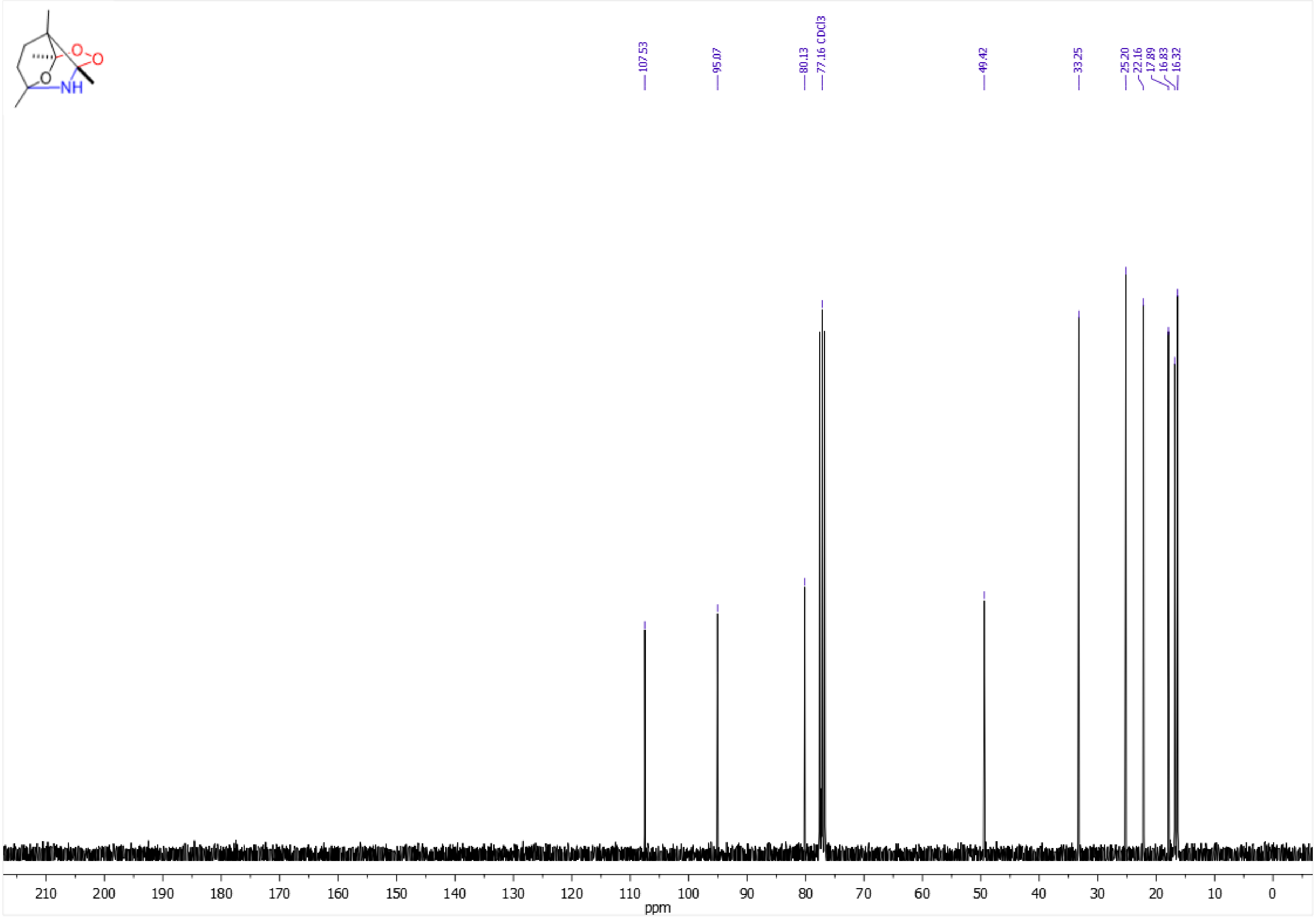

^{1}H NMR (300.13 MHz, CDCl3). 3a-(4-Bromobenzyl)-3,6,7a-trimethylhexahydro-3H-3,6-epoxy[1,2]dioxolo[3,4-b]pyridine, 37

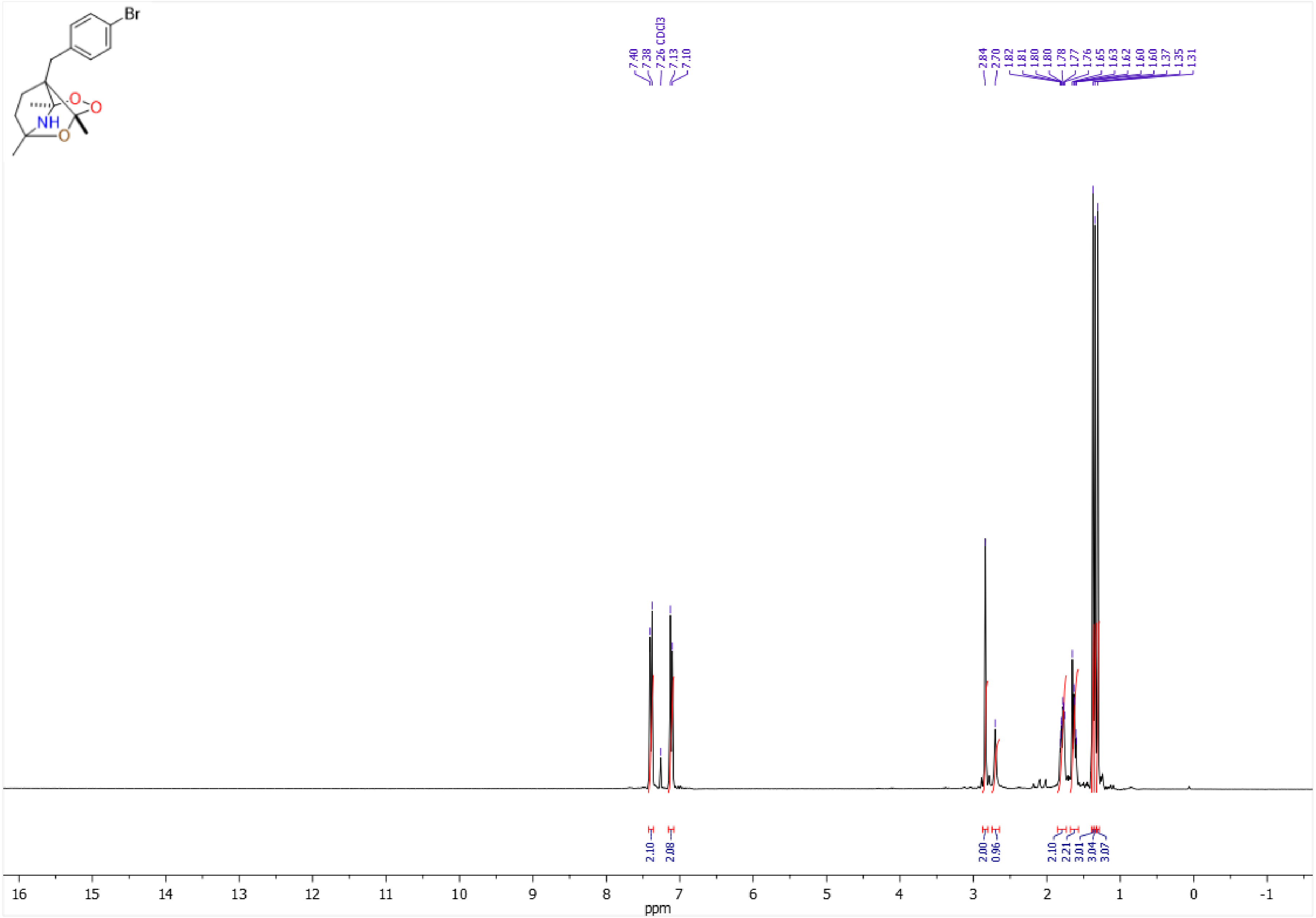

^{13}C NMR (75.48 MHz, $CDCl_3$). 3a-(4-Bromobenzyl)-3,6,7a-trimethylhexahydro-3H-3,6-epoxy[1,2]dioxolo[3,4-b]pyridine, 37

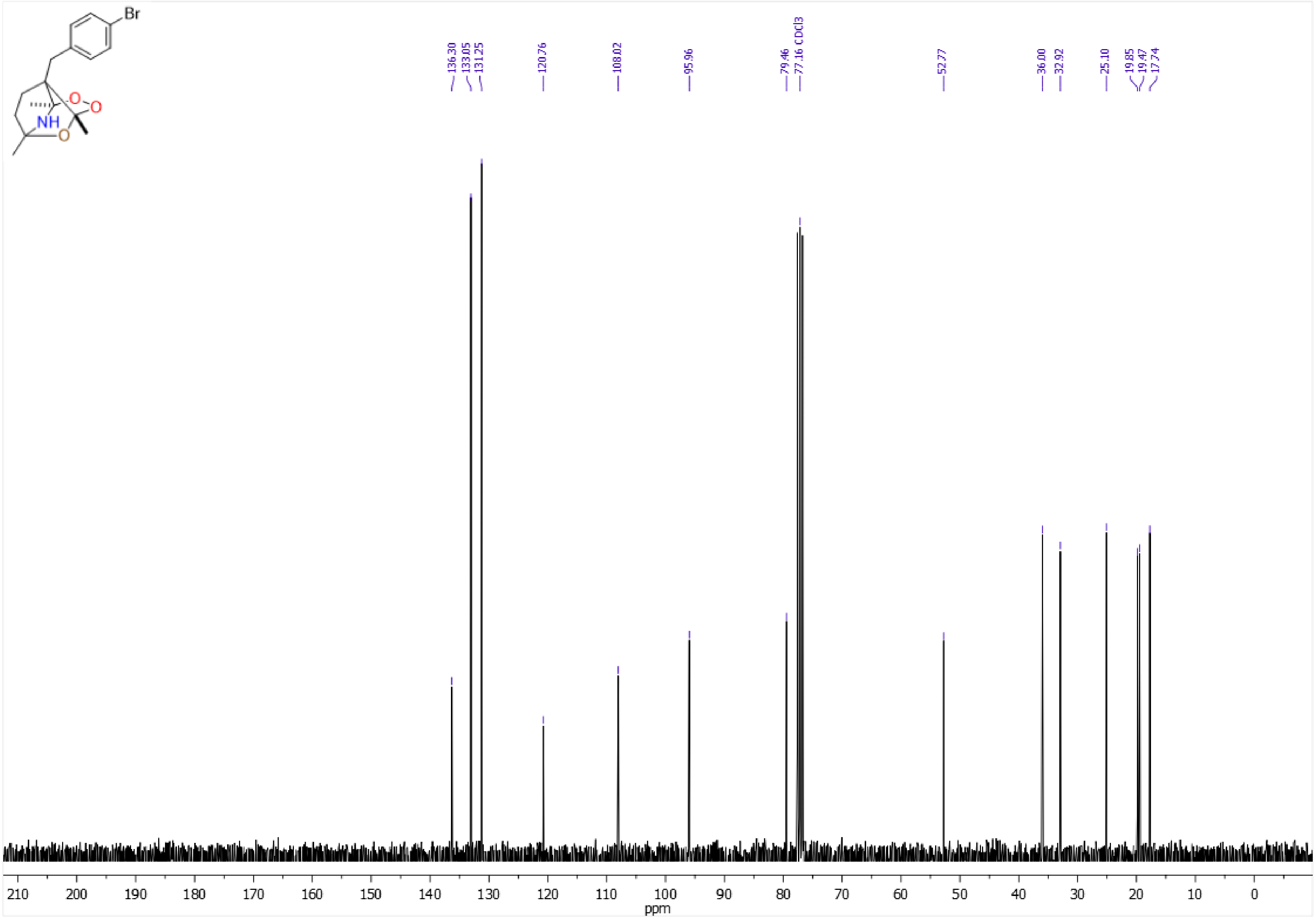

^{1}H NMR (300.13 MHz, CDCl3). 3a-Benzyl-3,6,7a-trimethylhexahydro-3H-3,6-epoxy[1,2]dioxolo[3,4-b]pyridine, 38

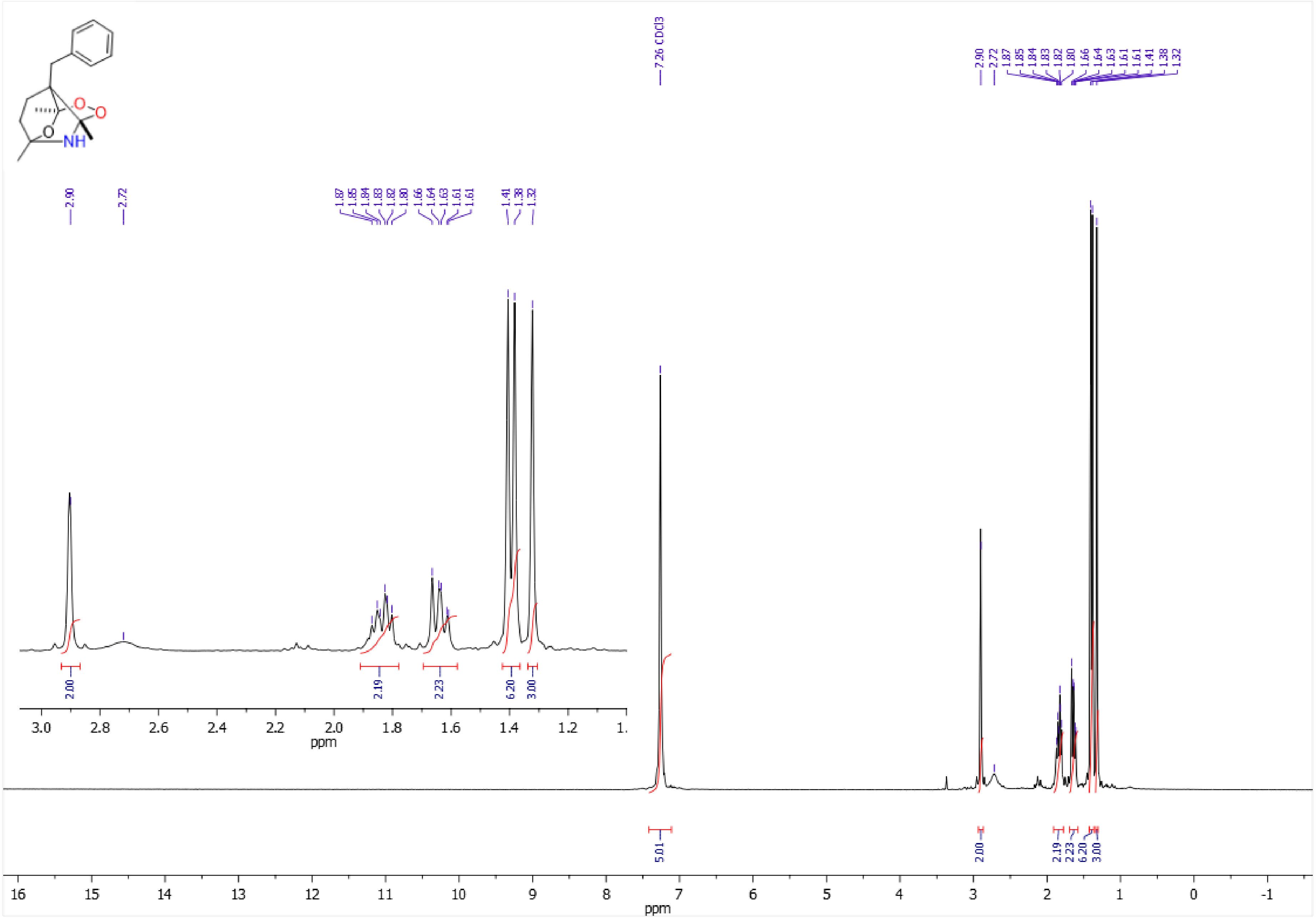

^{13}C NMR (75.48 MHz, CDCl3). 3a-Benzyl-3,6,7a-trimethylhexahydro-3H-3,6-epoxy[1,2]dioxolo[3,4-b]pyridine, 38

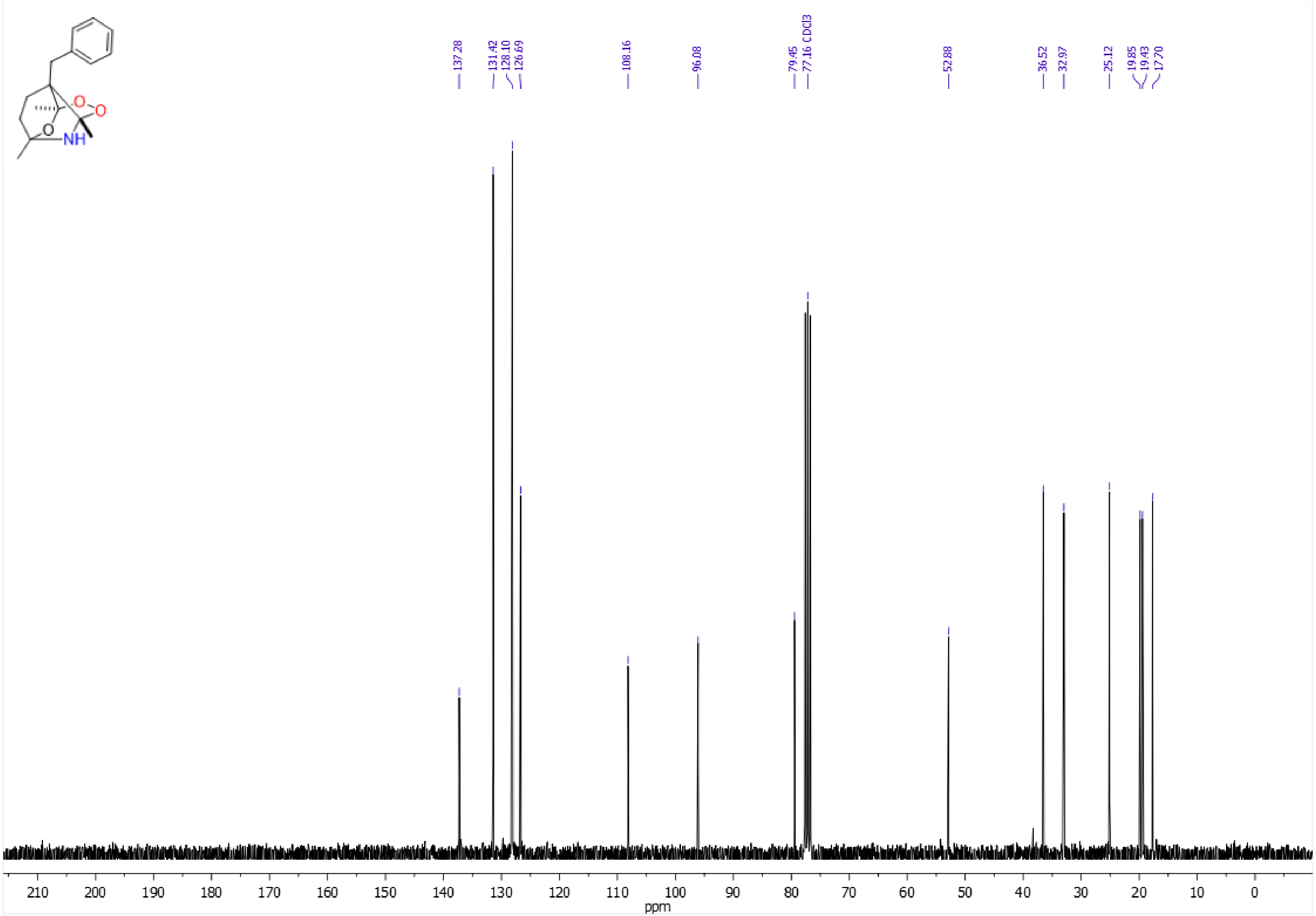

^{1}H NMR (300.13 MHz, $CDCl_3$). Ethyl 3-(3,6,7a-trimethyltetrahydro-3H-3,6-epoxy[1,2]dioxolo[3,4-b]pyridin-3a(4H)-yl)propanoate, 39

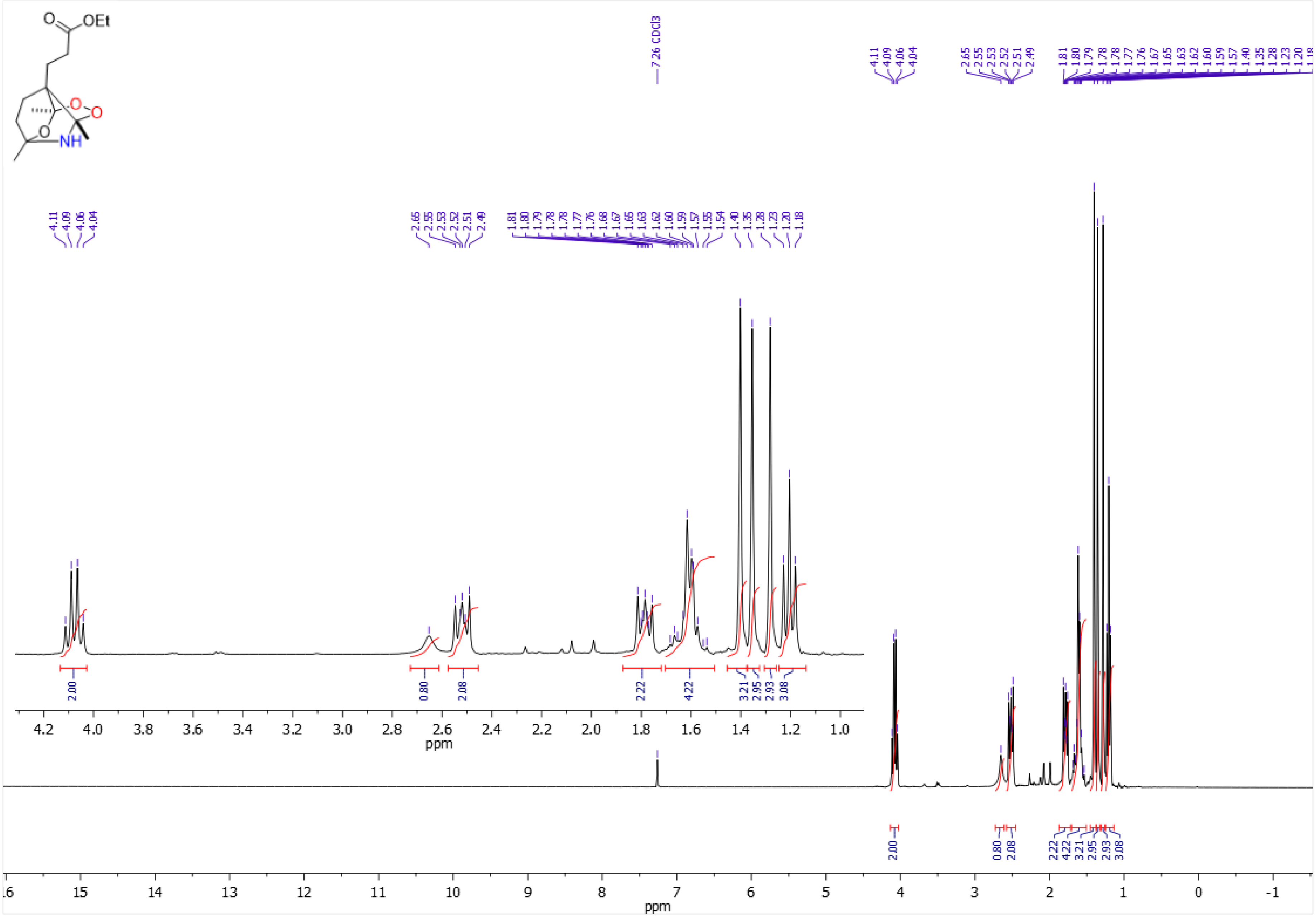

^{13}C NMR (75.48 MHz, CDCl3). Ethyl 3-(3,6,7a-trimethyltetrahydro-3H-3,6-epoxy[1,2]dioxolo[3,4-b]pyridin-3a(4H)-yl)propanoate, 39

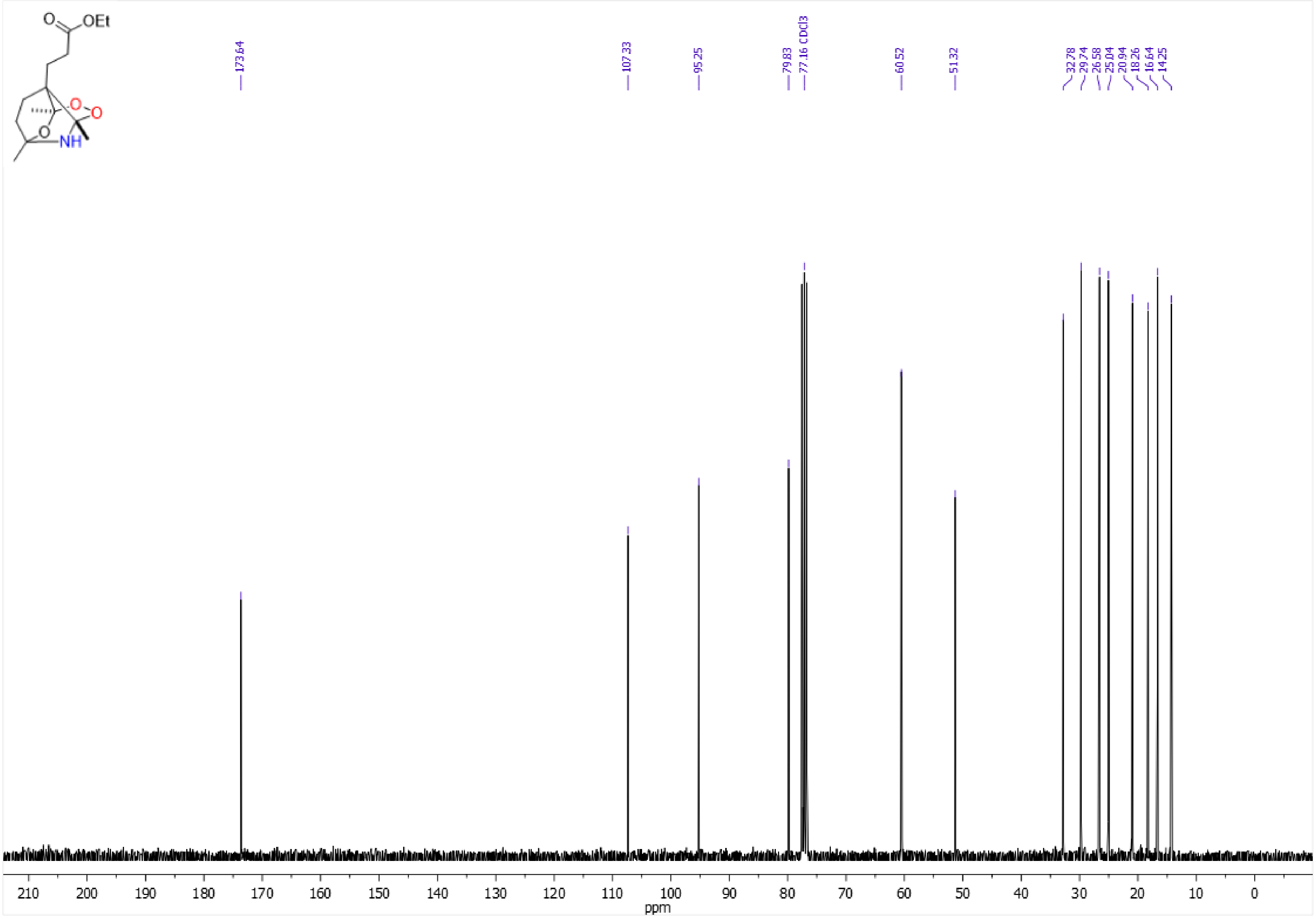

^{1}H NMR (300.13 MHz, CDCl3). 3-(-3,6,7a-Trimethyltetrahydro-3H-3,6-epoxy[1,2]dioxolo[3,4-b]pyridin-3a(4H)-yl)propanenitrile, 40

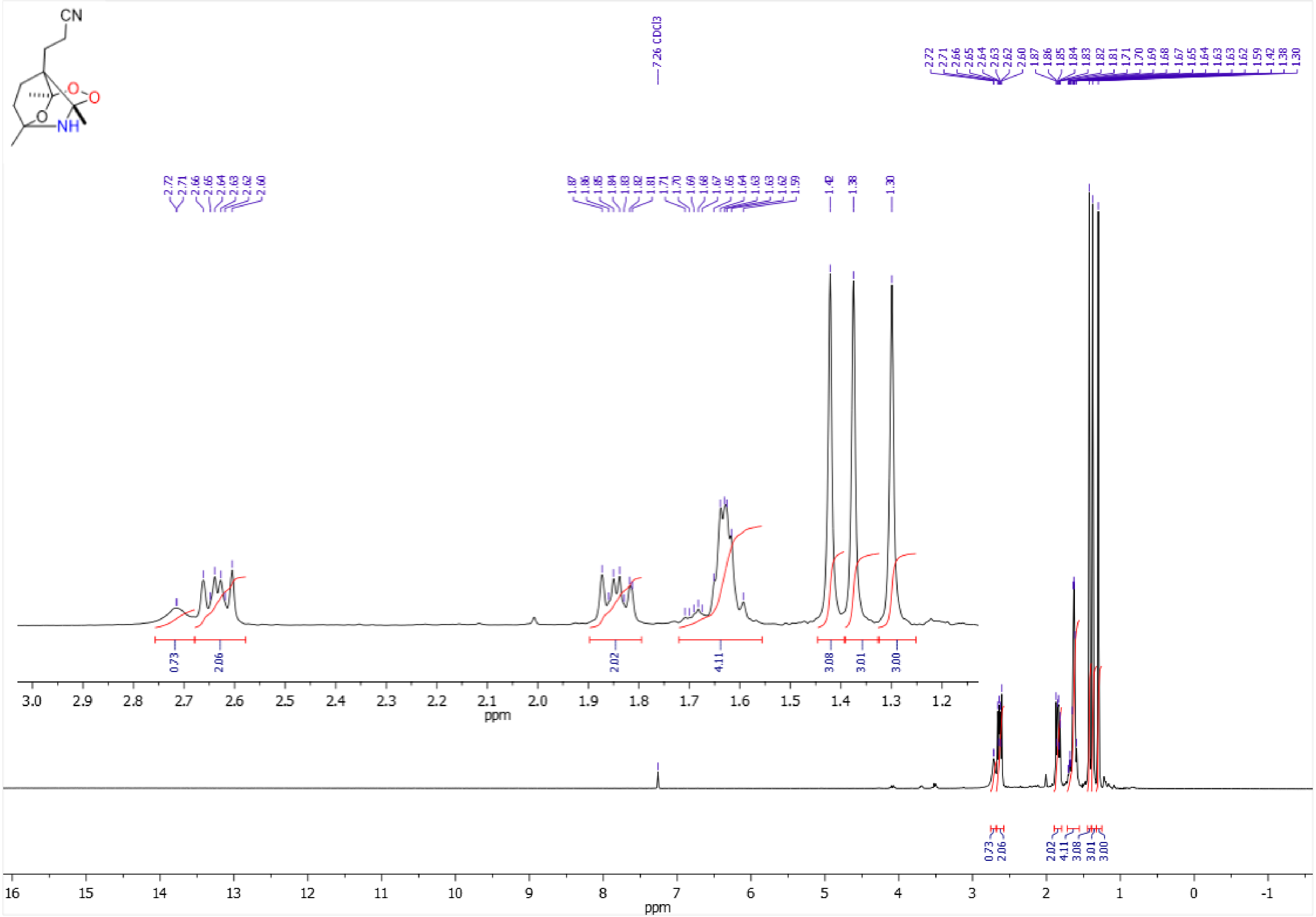

^{13}C NMR (75.48 MHz, $CDCl_3$). 3-(-3,6,7a-Trimethyltetrahydro-3H-3,6-epoxy[1,2]dioxolo[3,4-b]pyridin-3a(4H)-yl)propanenitrile, 40

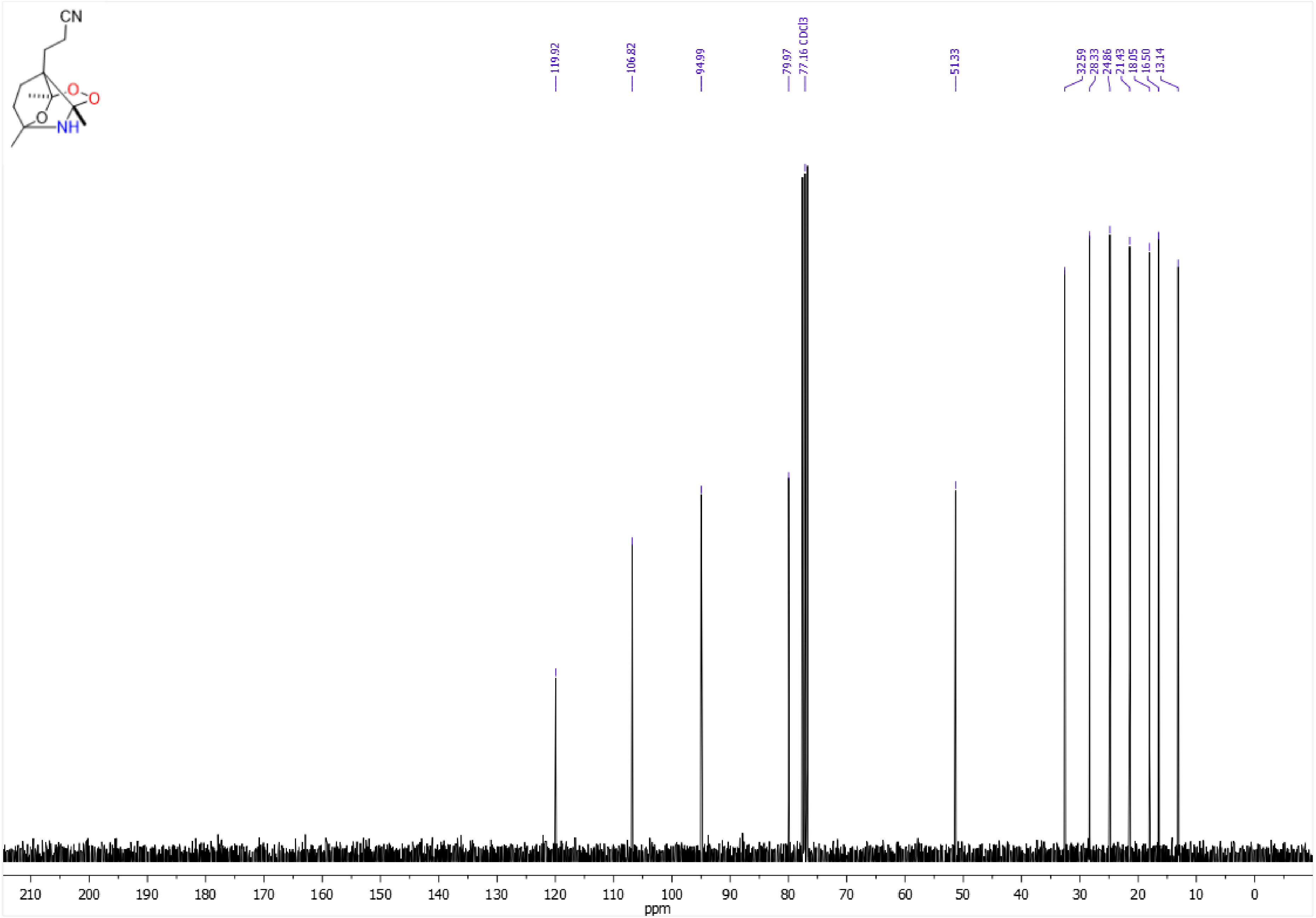

^{1}H NMR (300.13 MHz, CDCl3). 3a-Ethyl-3,6,7a-trimethylhexahydro-3H-3,6-epoxy[1,2]dioxolo[3,4-b]pyridine, 41

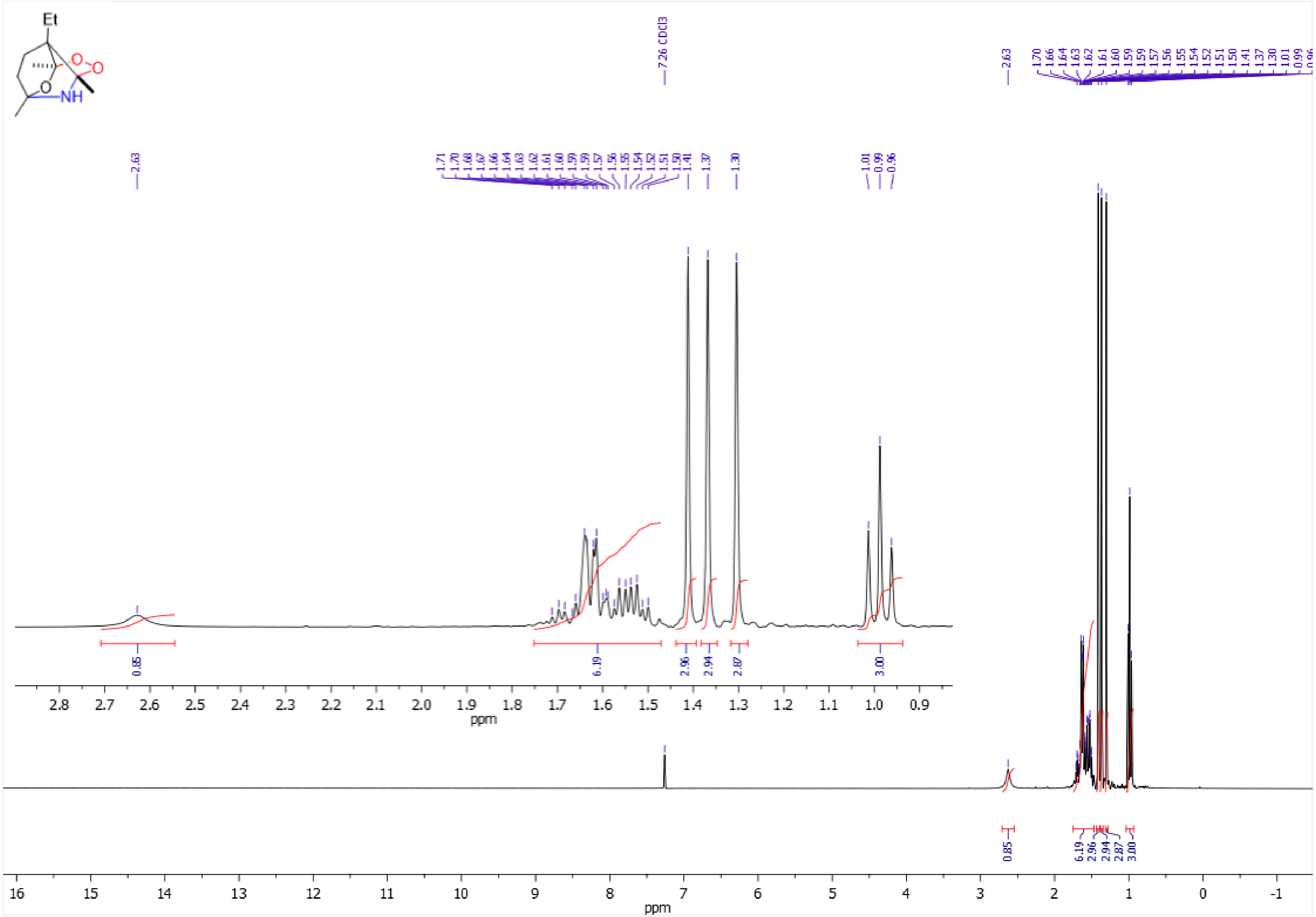

^{13}C NMR (75.48 MHz, $CDCl_3$). 3a-Ethyl-3,6,7a-trimethylhexahydro-3H-3,6-epoxy[1,2]dioxolo[3,4-b]pyridine, 41

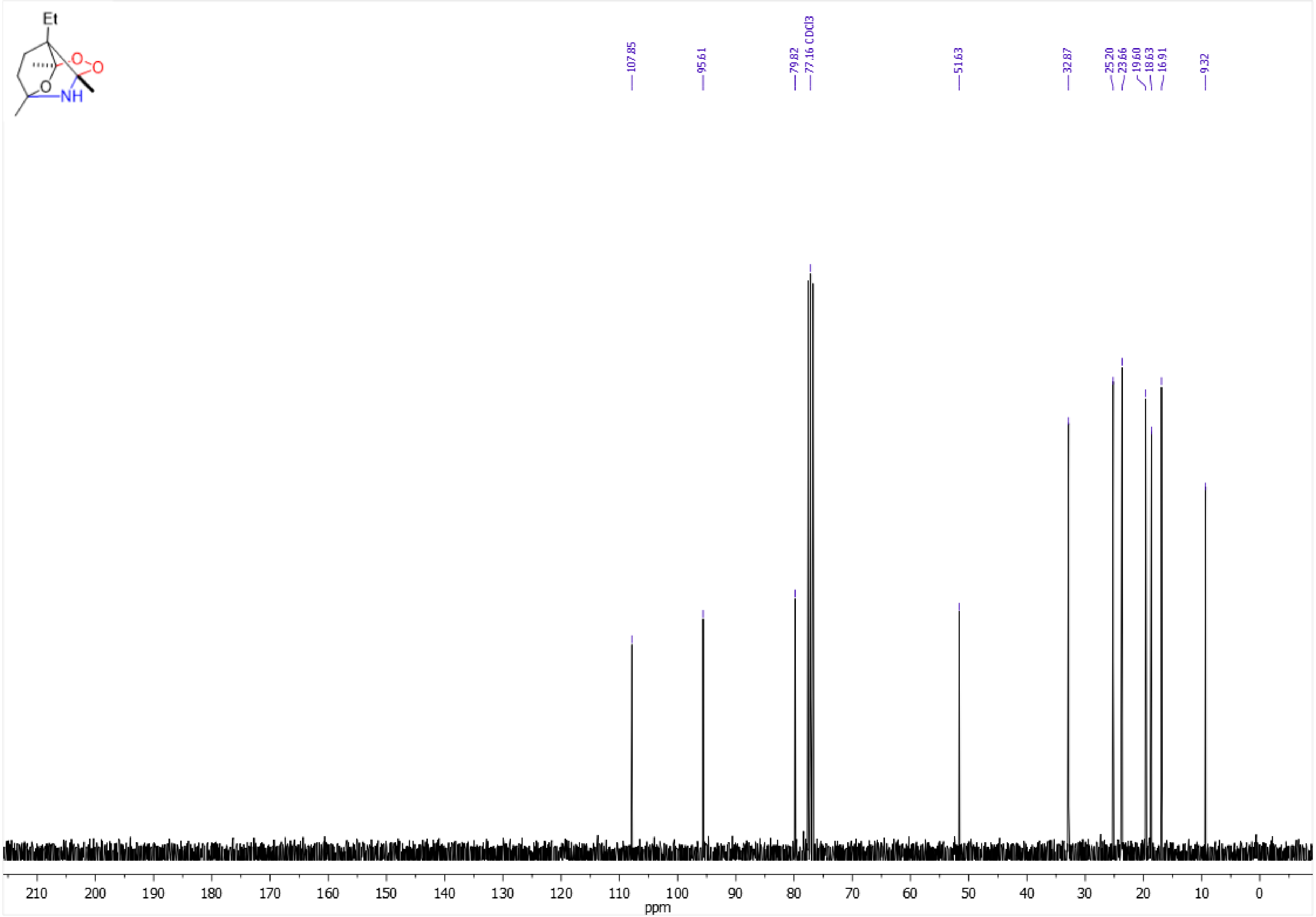

^{1}H NMR (300.13 MHz, CDCl3). 3,6,7a-Trimethyl-3a-octylhexahydro-3H-3,6-epoxy[1,2]dioxolo[3,4-b]pyridine, 42

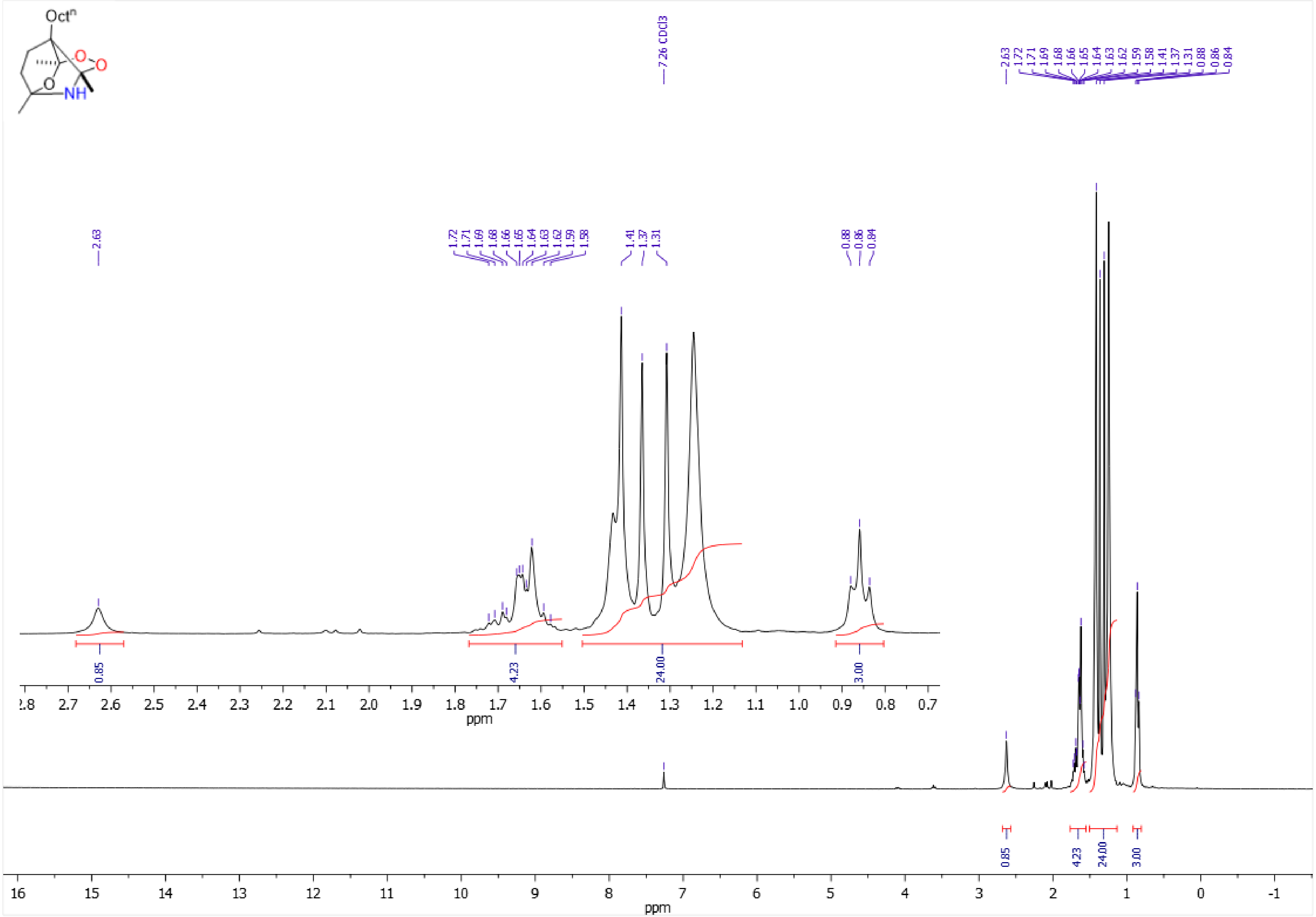

^{13}C NMR (75.48 MHz, $CDCl_3$). 3,6,7a-Trimethyl-3a-octylhexahydro-3H-3,6-epoxy[1,2]dioxolo[3,4-b]pyridine, 42

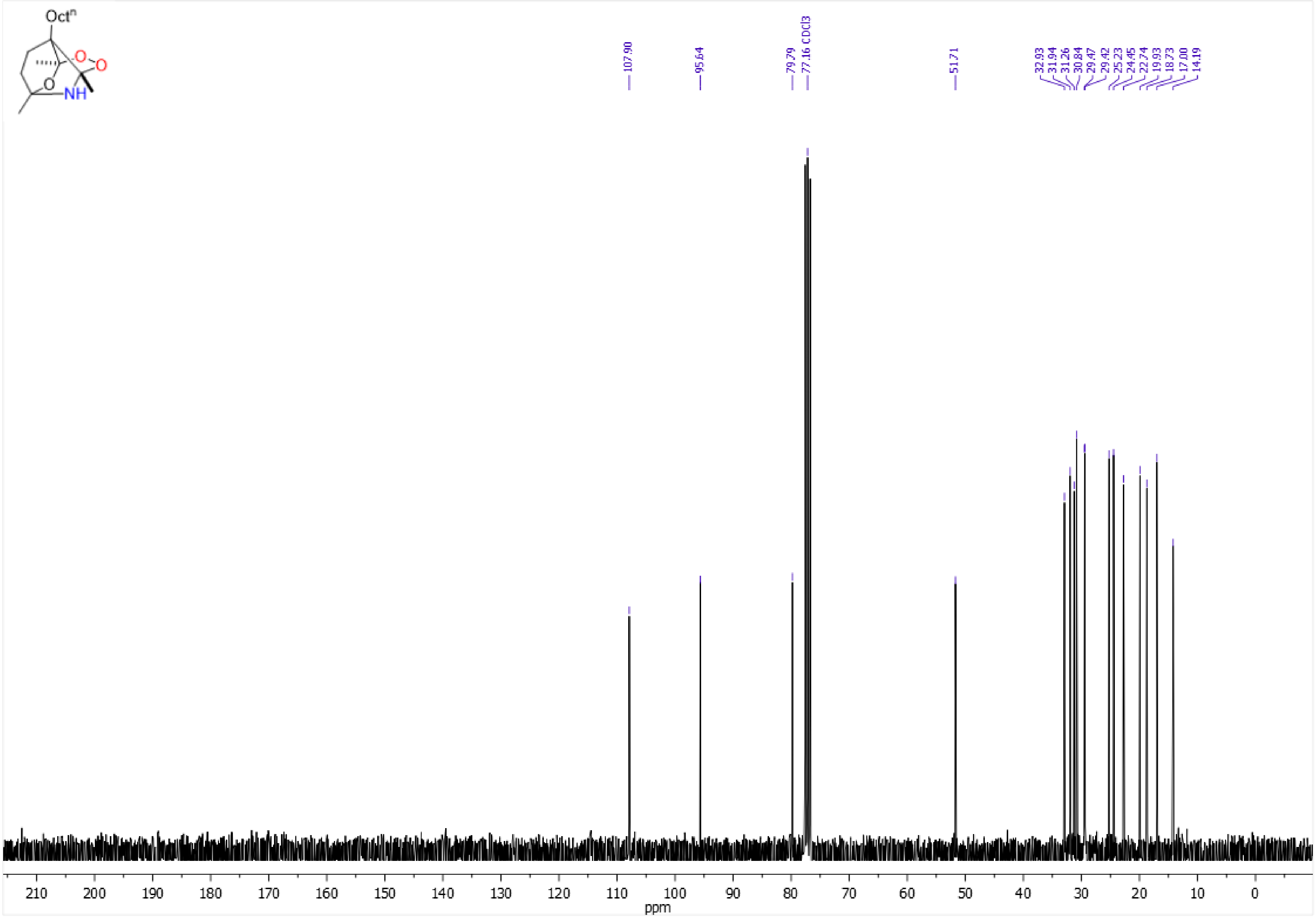

^{1}H NMR (300.13 MHz, $CDCl_3$). 3,6,7a-Trimethyl-3a-nonylhexahydro-3H-3,6-epoxy[1,2]dioxolo[3,4-b]pyridine, 43

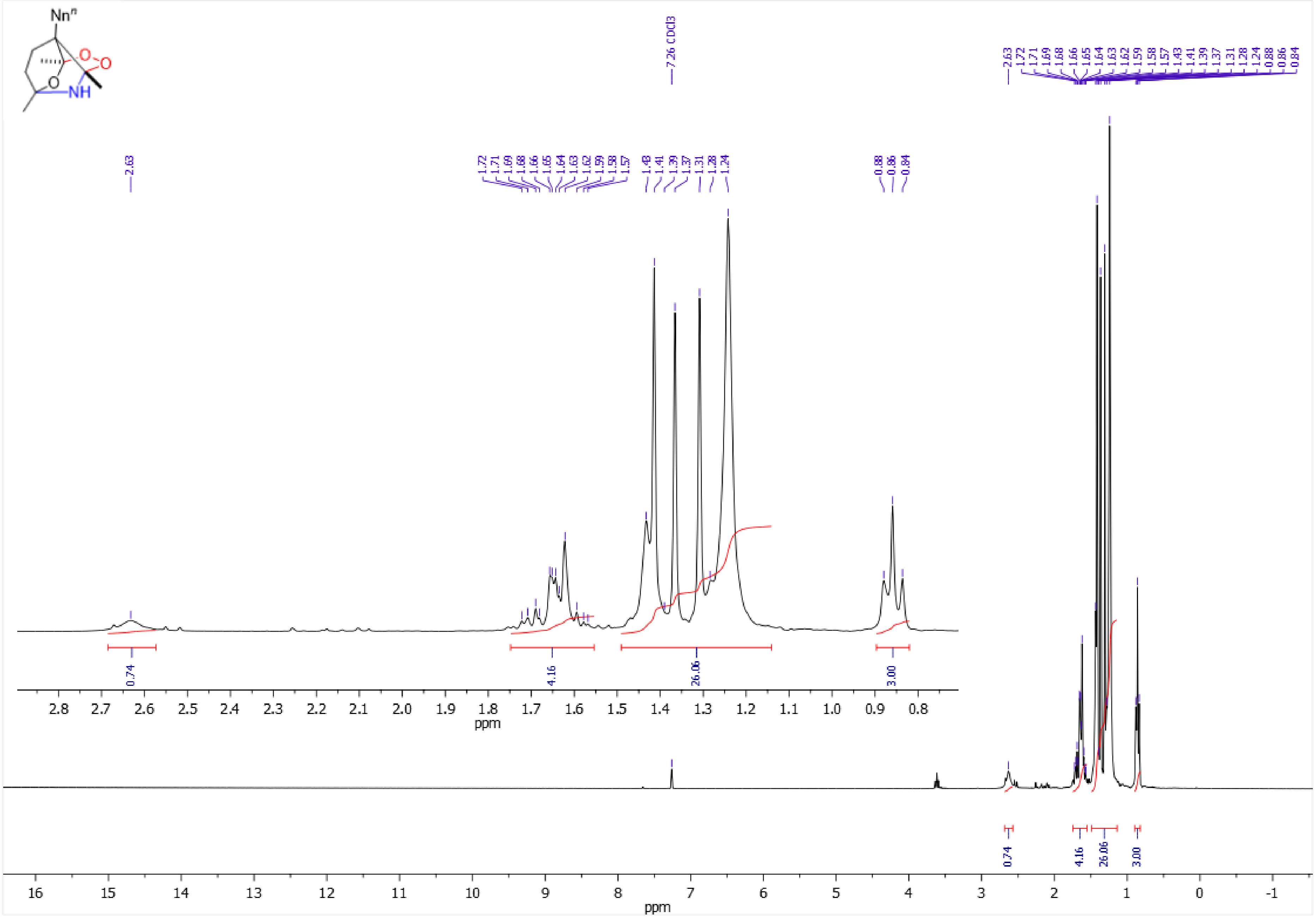

^{13}C NMR (75.48 MHz, $CDCl_3$). 3,6,7a-Trimethyl-3a-nonylhexahydro-3H-3,6-epoxy[1,2]dioxolo[3,4-b]pyridine, 43

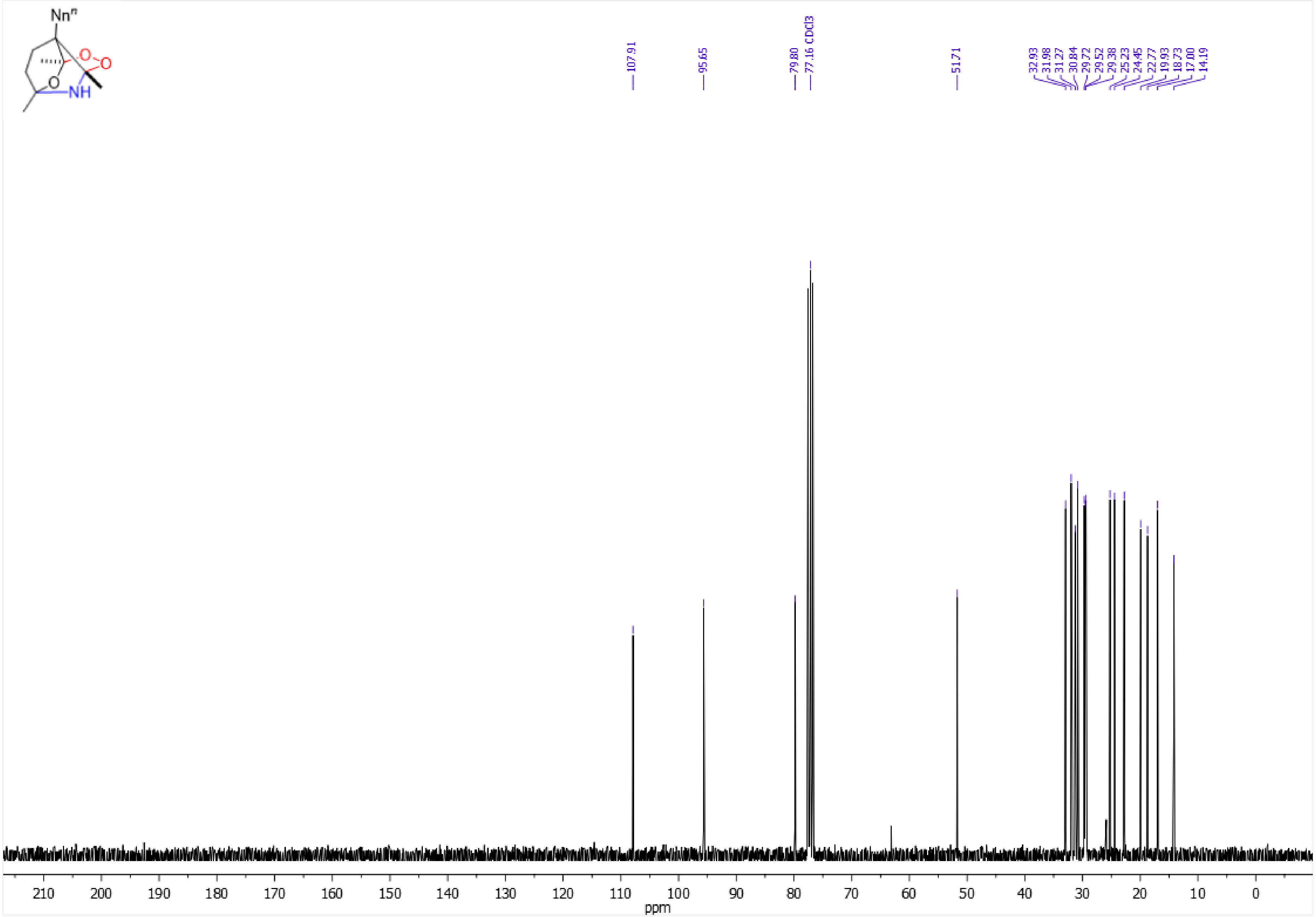

^{1}H NMR (300.13 MHz, CDCl3). 3,6,7a-Trimethyl-3a-(4-nitrobenzyl)hexahydro-3H-3,6-epoxy[1,2]dioxolo[3,4-b]pyridine, 44

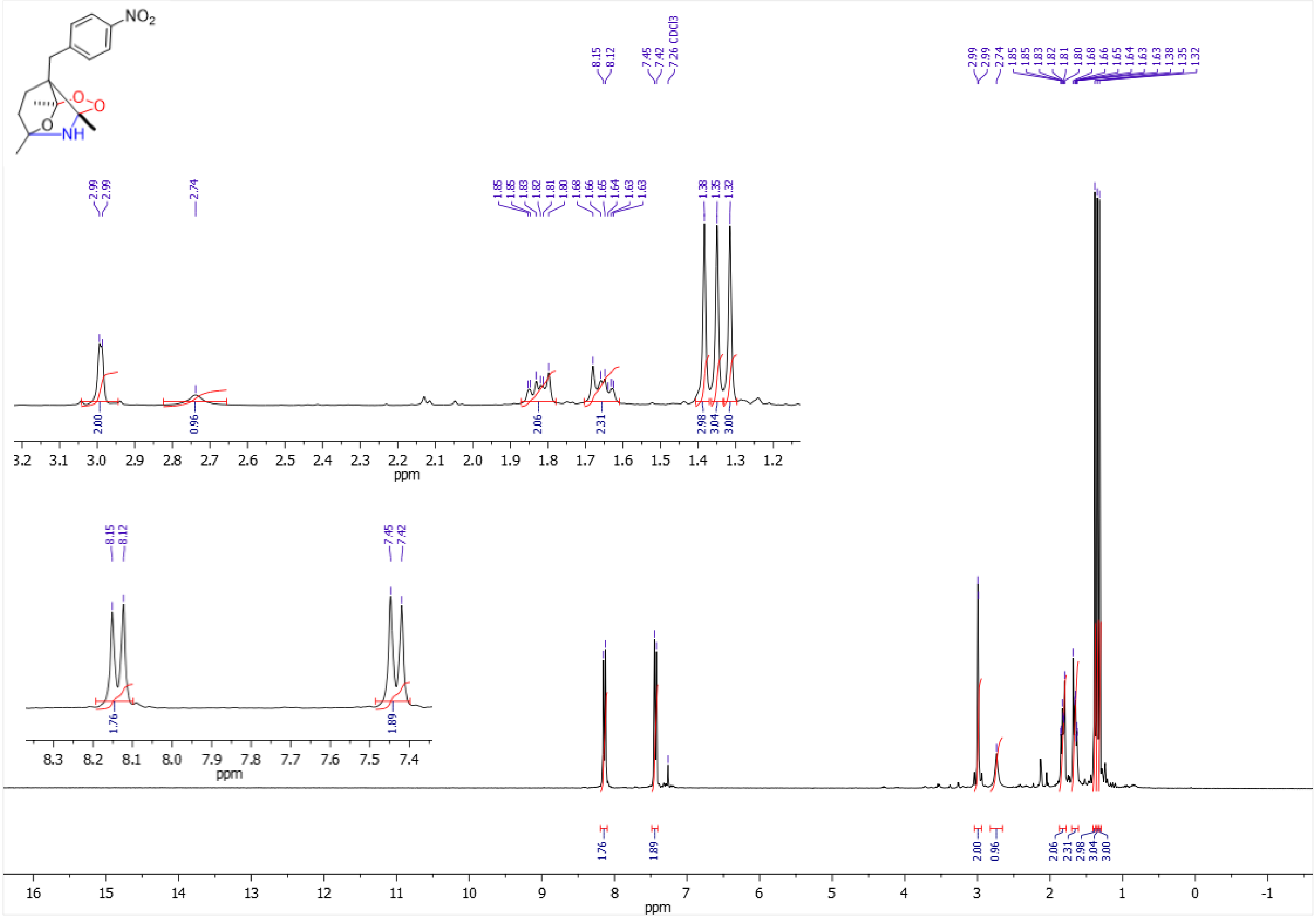

^{13}C NMR (75.48 MHz, $CDCl_3$). 3,6,7a-Trimethyl-3a-(4-nitrobenzyl)hexahydro-3H-3,6-epoxy[1,2]dioxolo[3,4-b]pyridine, 44

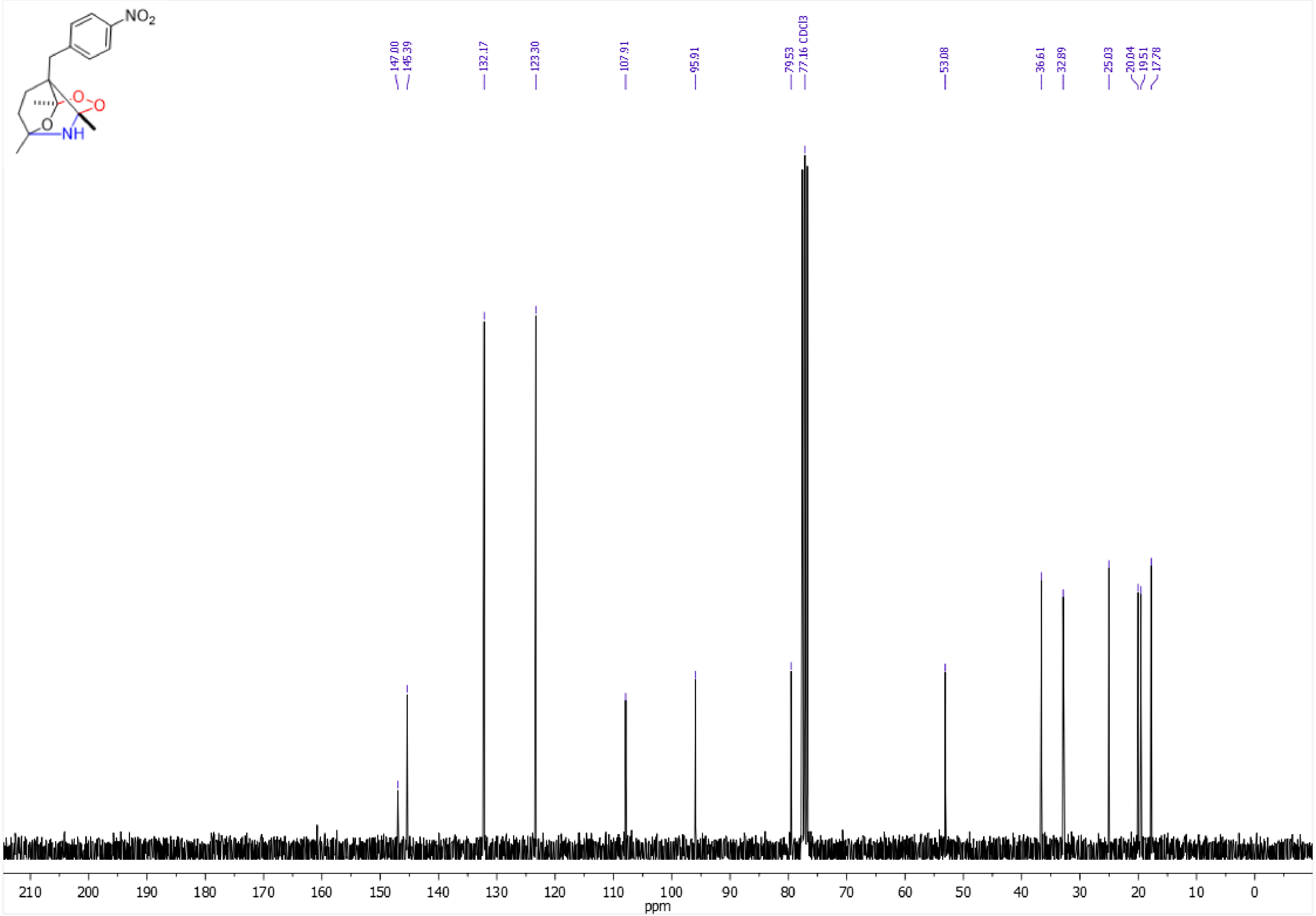

^{1}H NMR (300.13 MHz, $CDCl_3$). 3,7a-Diethyl-6-methylhexahydro-3H-3,6-epoxy[1,2]dioxolo[3,4-b]pyridine, 45

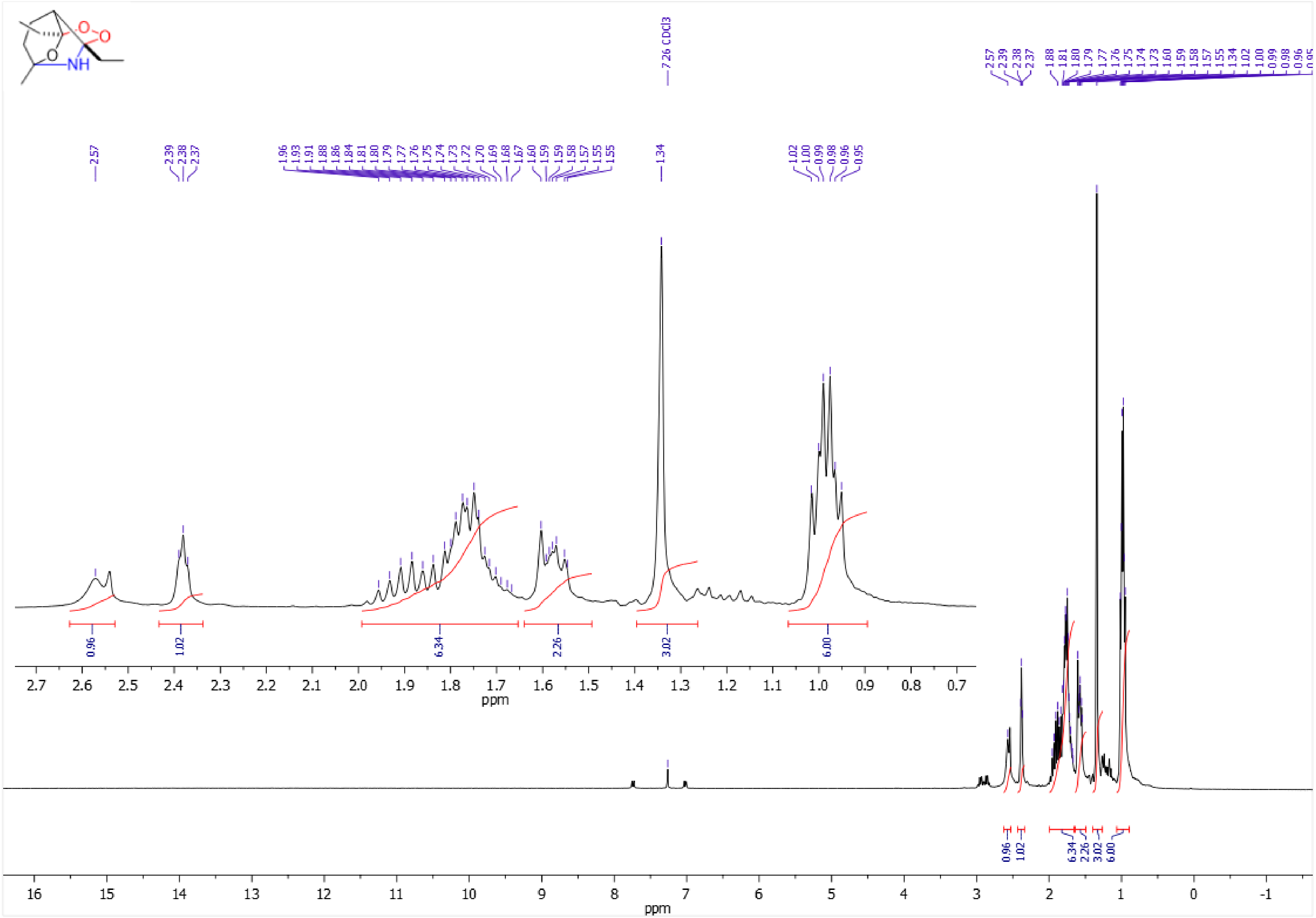

^{13}C NMR (75.48 MHz, CDCl3). 3,7a-Diethyl-6-methylhexahydro-3H-3,6-epoxy[1,2]dioxolo[3,4-b]pyridine, 45

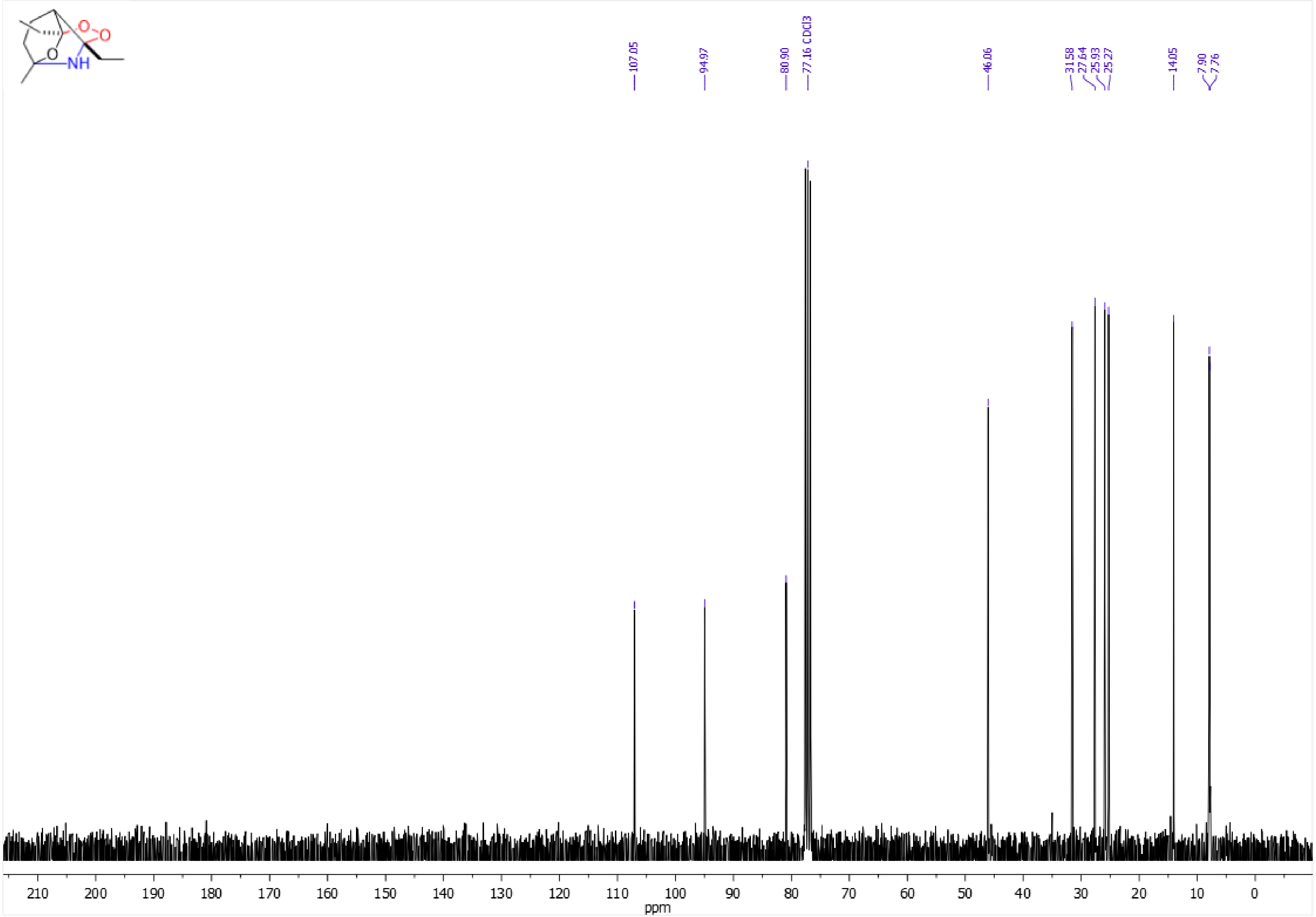

^{1}H NMR (300.13 MHz, $CDCl_3$). Ethyl (1*R,2*S**,5*S**)-1,5-dimethyl-6,7,8-trioxabicyclo[3.2.1]octane-2-carboxylate, 46**

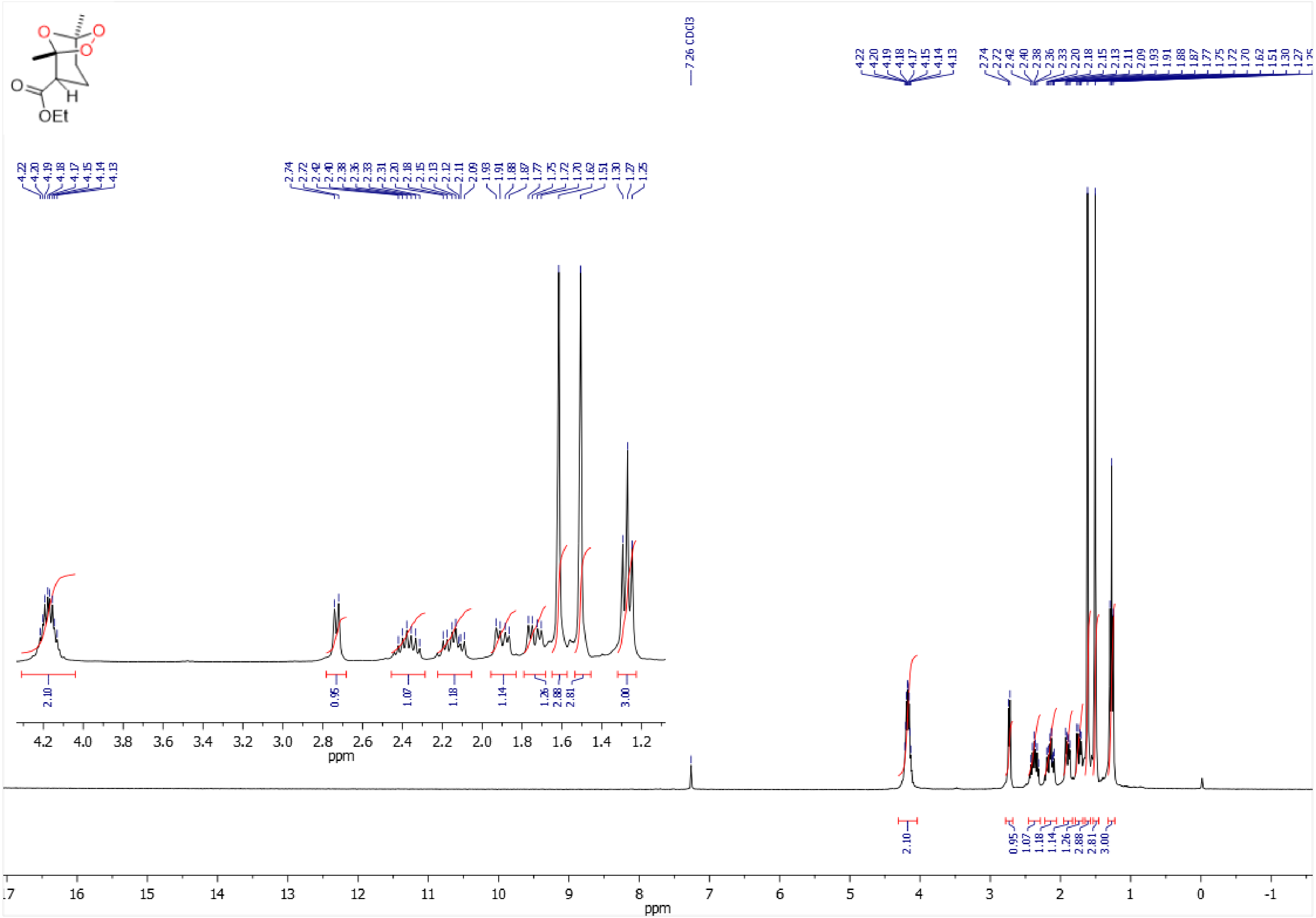

^{13}C NMR (75.48 MHz, $CDCl_3$). Ethyl (1*R**,2*S**,5*S**)-1,5-dimethyl-6,7,8-trioxabicyclo[3.2.1]octane-2-carboxylate, 46

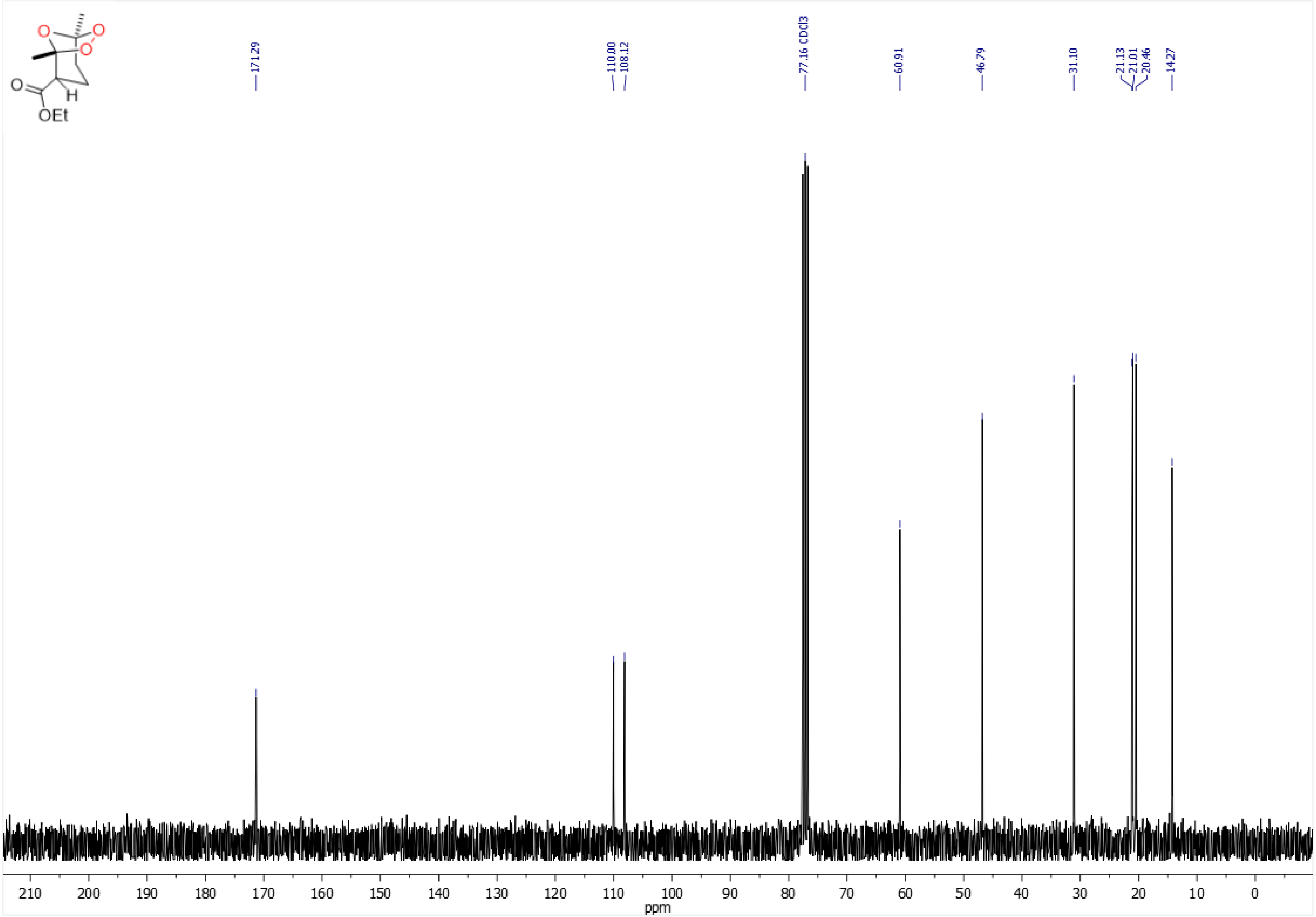

^{1}H NMR (300.13 MHz, CDCl3). Ethyl (1*S**,2*S**,5*R**)-1,5-dimethyl-6,7,8-trioxabicyclo[3.2.1]octane-2-carboxylate, 47

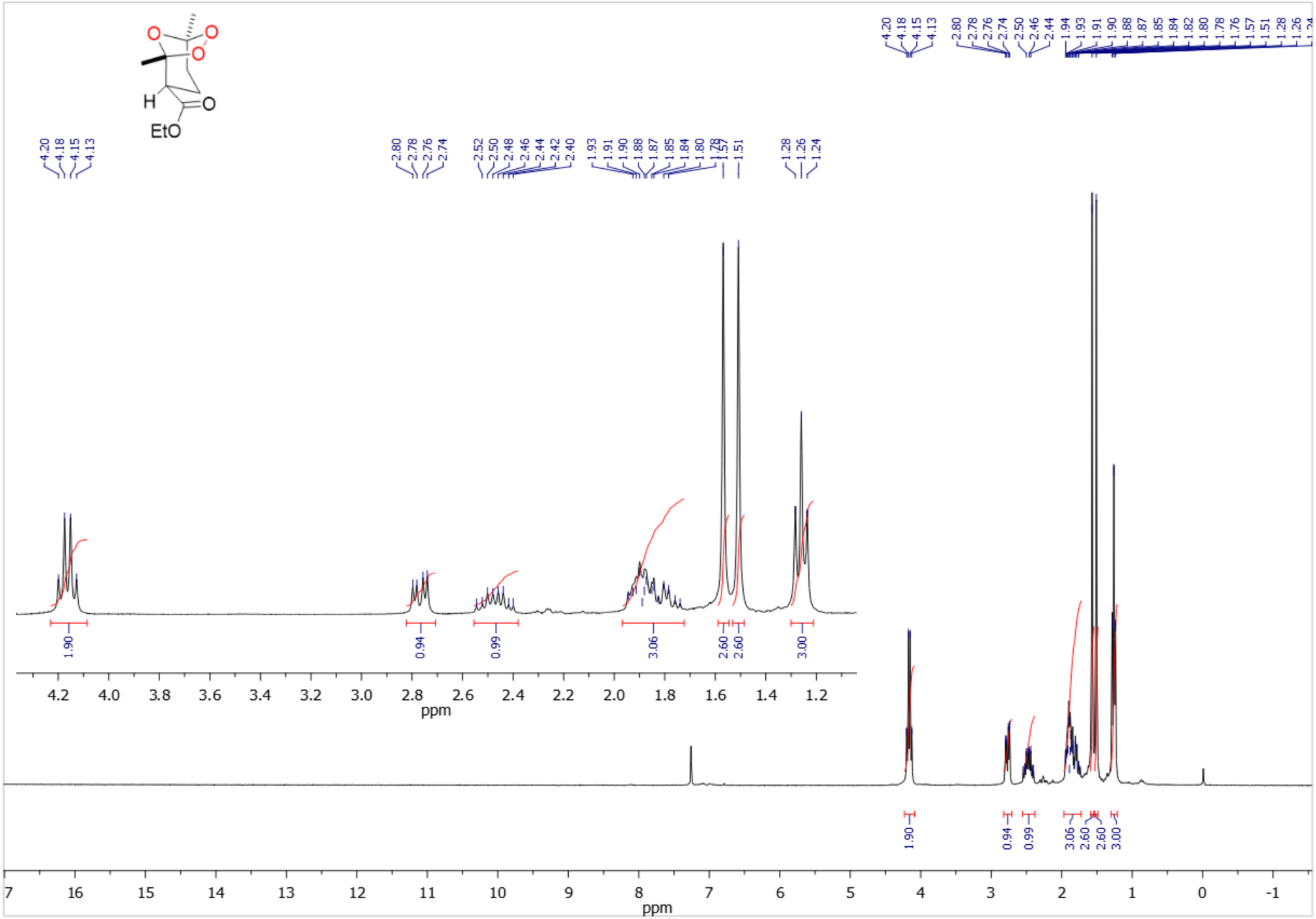

^{13}C NMR (75.48 MHz, $CDCl_3$). Ethyl (1*S**,2*S**,5*R**)-1,5-dimethyl-6,7,8-trioxabicyclo[3.2.1]octane-2-carboxylate, 47

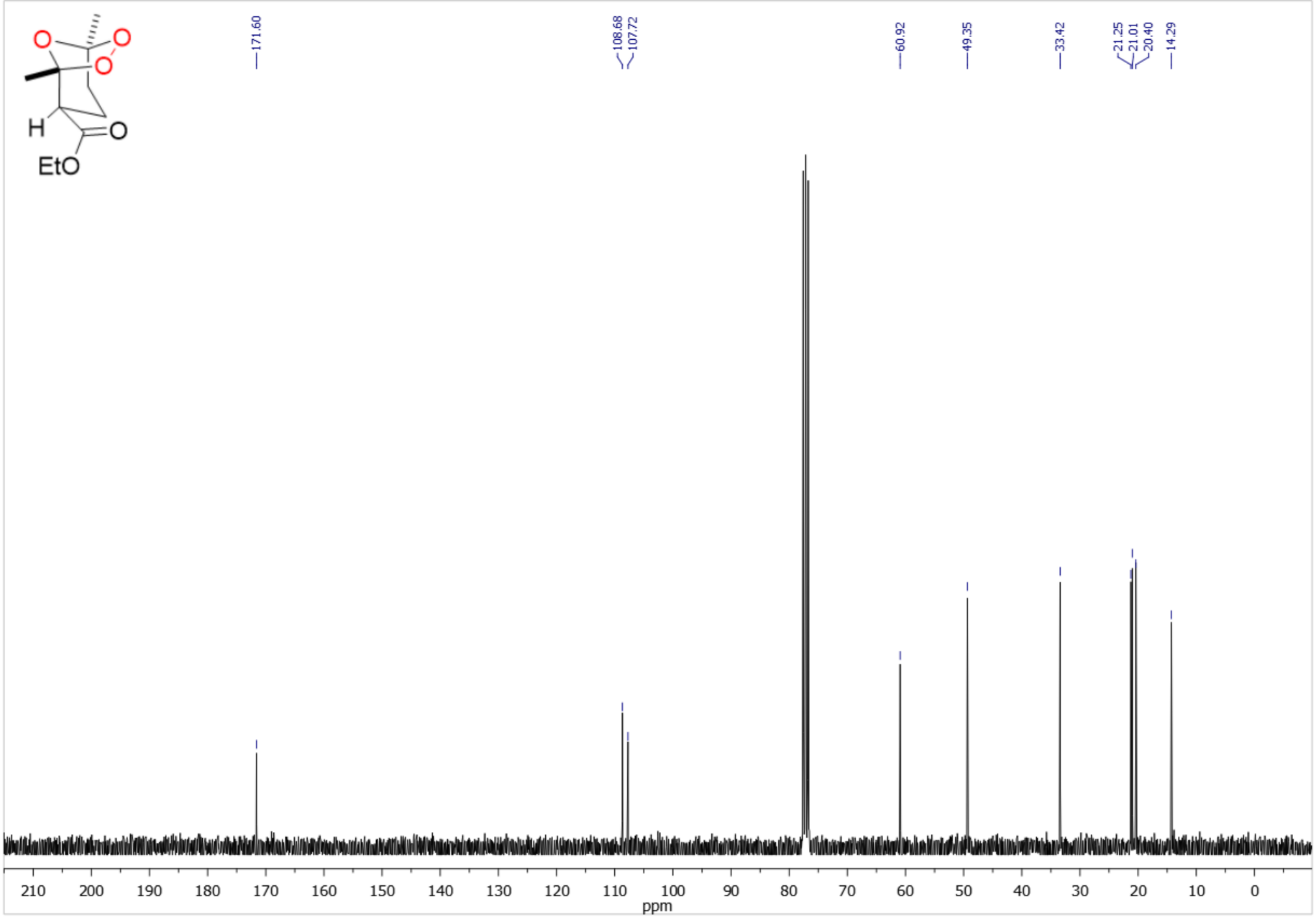

^{1}H NMR (300.13 MHz, CDCl3). Ethyl (1*R**,2*R**,5*S**)-1,5-dimethyl-2-(prop-2-yn-1-yl)-6,7,8-trioxabicyclo[3.2.1]octane-2-carboxylate, 48

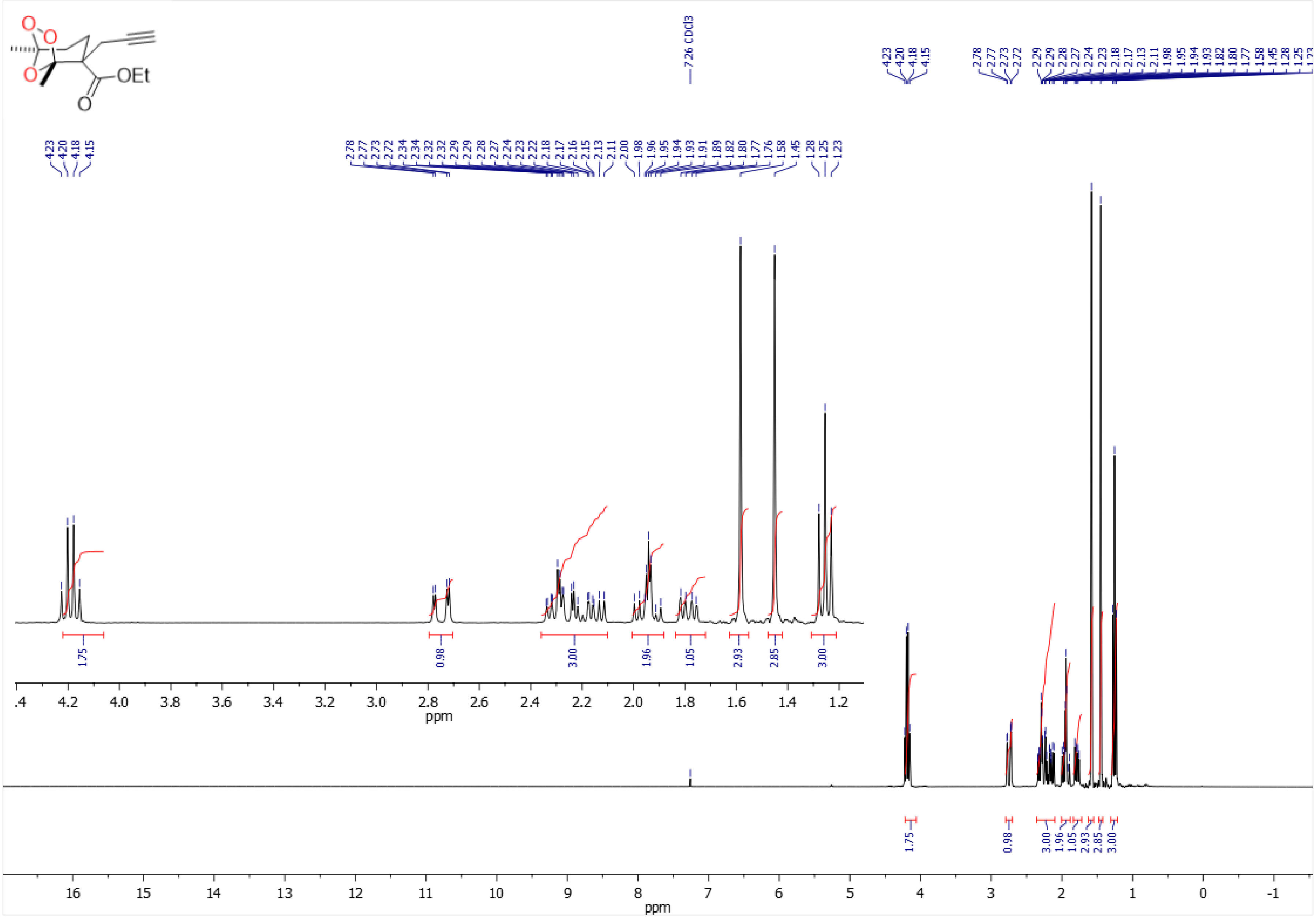

^{13}C NMR (75.48 MHz, CDCl$_3$). Ethyl (1*R**,2*R**,5*S**)-1,5-dimethyl-2-(prop-2-yn-1-yl)-6,7,8-trioxabicyclo[3.2.1]octane-2-carboxylate, 48

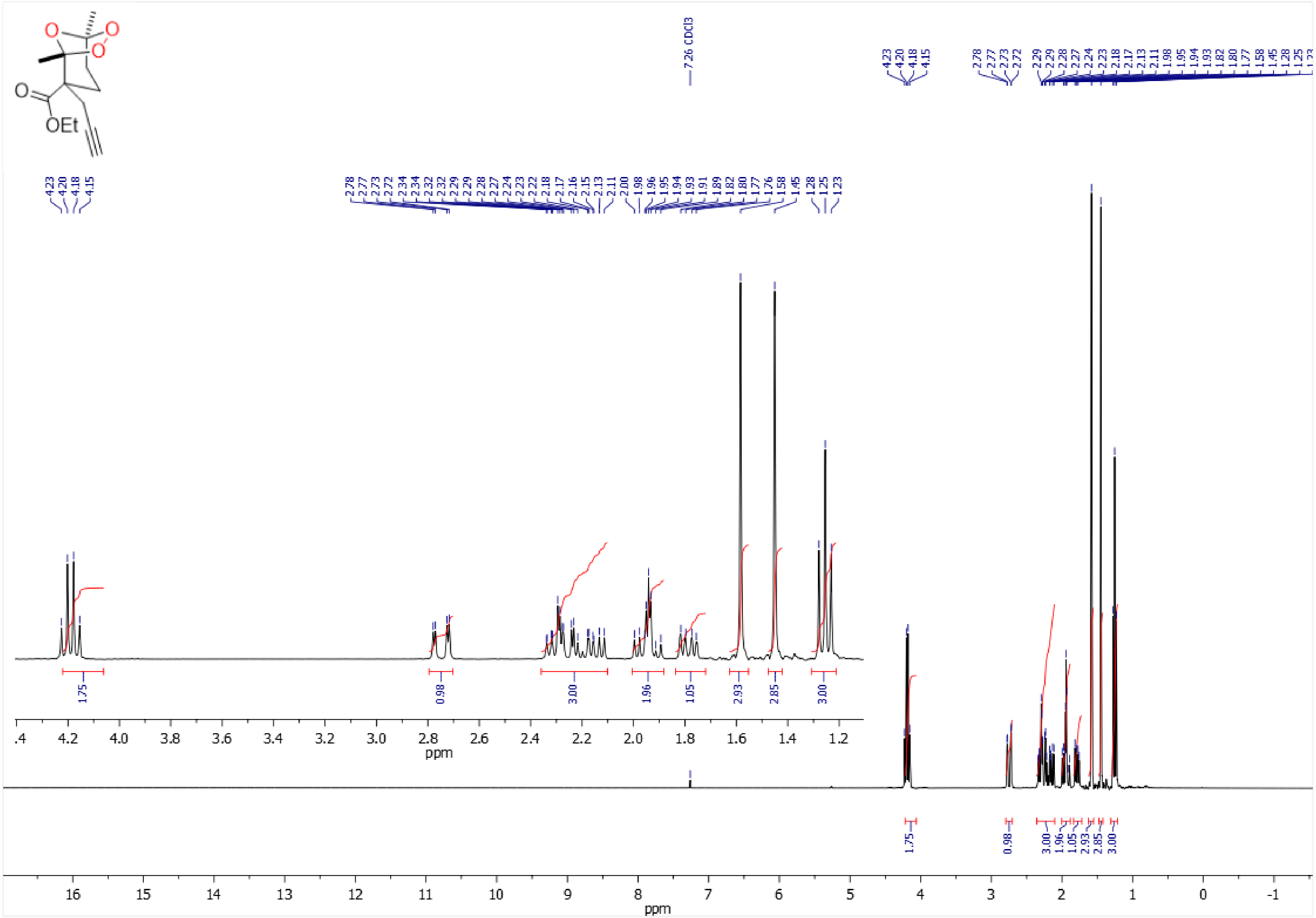

^{1}H NMR (300.13 MHz, CDCl3). Ethyl (1*S**,2*R**,5*R**)-1,5-dimethyl-2-(prop-2-yn-1-yl)-6,7,8-trioxabicyclo[3.2.1]octane-2-carboxylate, 49

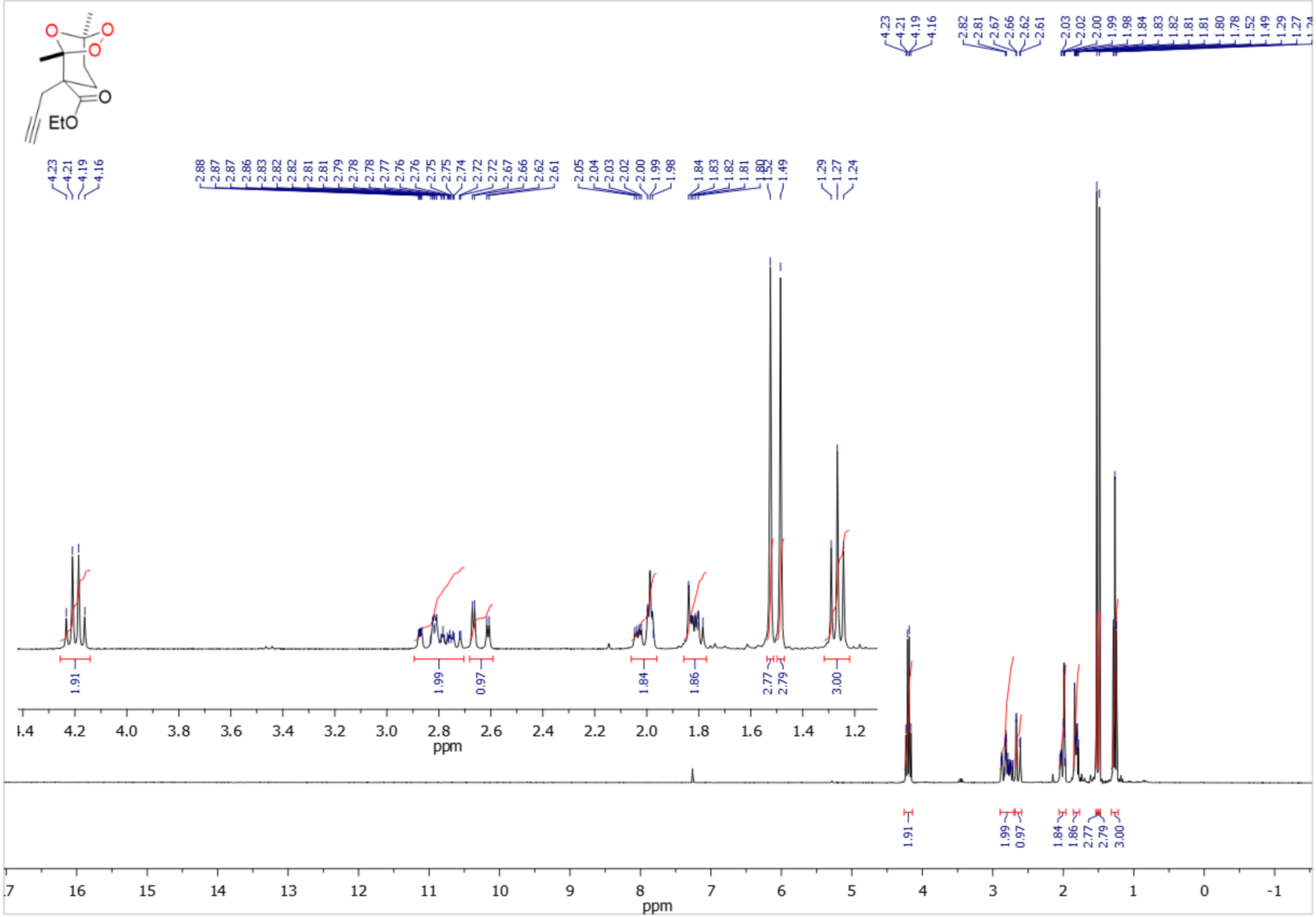

^{13}C NMR (75.48 MHz, $CDCl_3$). Ethyl (1*S**,2*R**,5*R**)-1,5-dimethyl-2-(prop-2-yn-1-yl)-6,7,8-trioxabicyclo[3.2.1]octane-2-carboxylate, 49

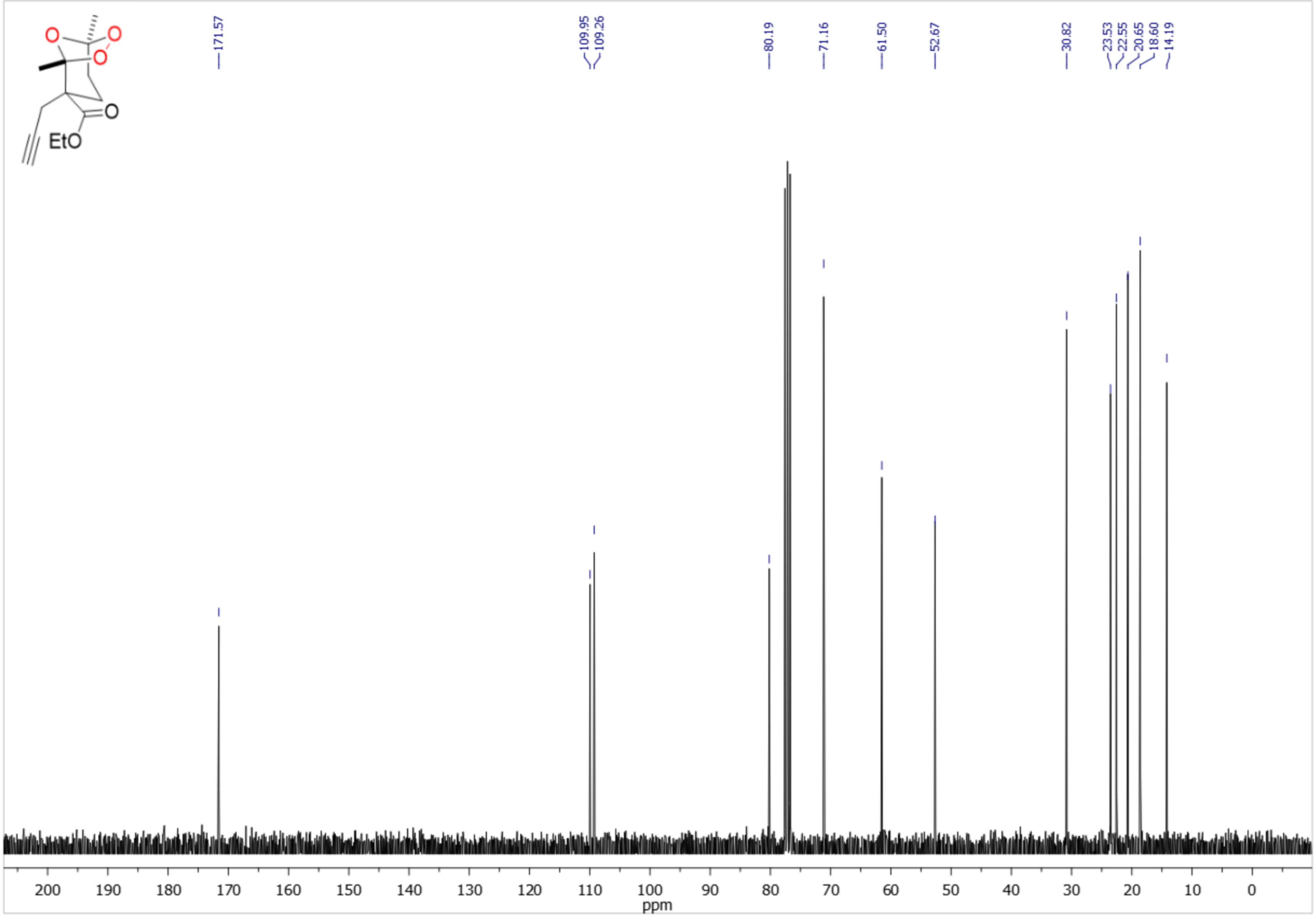

^{1}H NMR (300.13 MHz, CDCl$_3$). Ethyl (1*R,2*R**,5*S**)-2-allyl-1,5-dimethyl-6,7,8-trioxabicyclo[3.2.1]octane-2-carboxylate, 50**

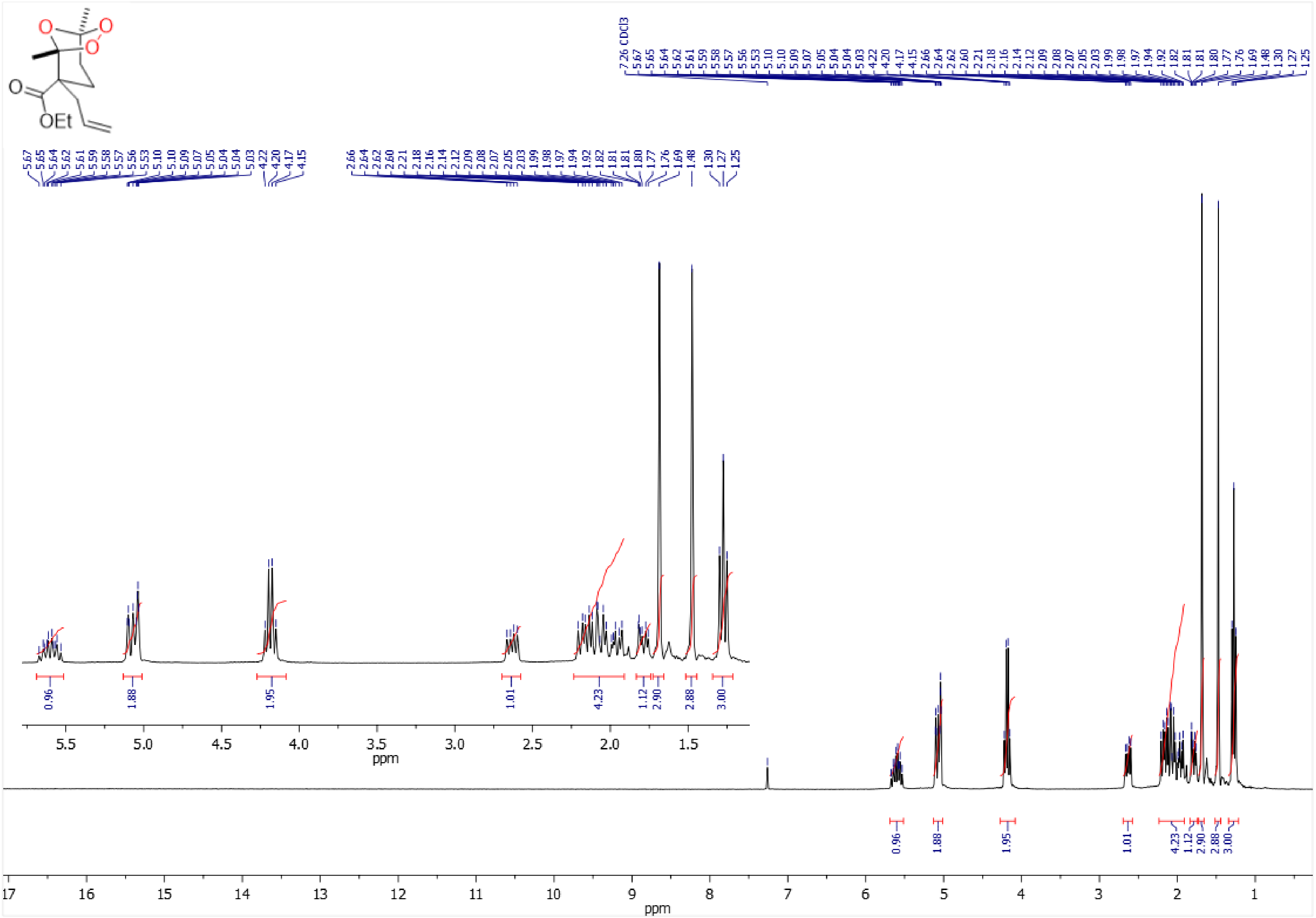

^{13}C NMR (75.48 MHz, CDCl$_3$). Ethyl (1*R**,2*R**,5*S**)-2-allyl-1,5-dimethyl-6,7,8-trioxabicyclo[3.2.1]octane-2-carboxylate, 50

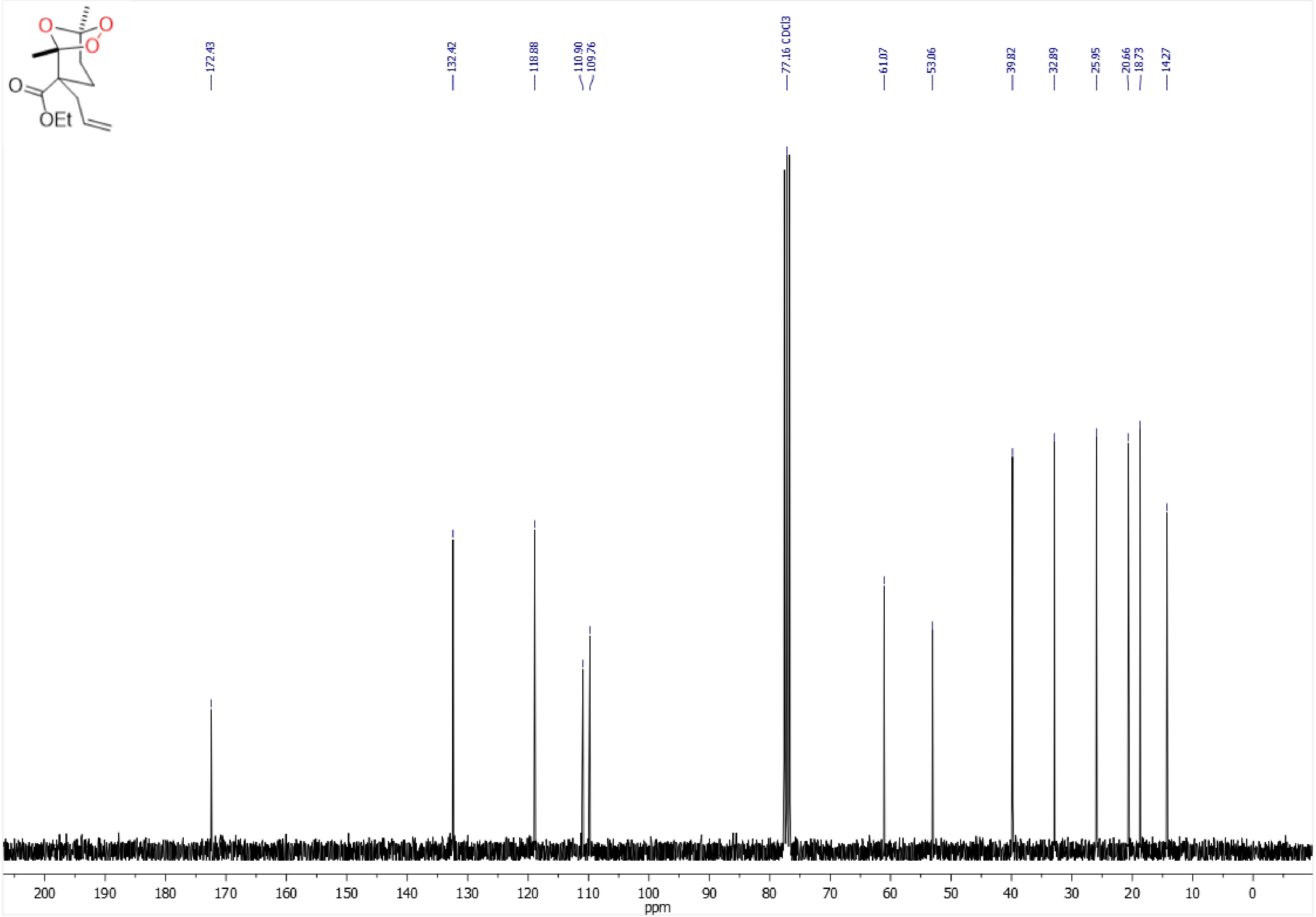

^{1}H NMR (300.13 MHz, CDCl3). Ethyl (1*S**,2*R**,5*R**)-2-allyl-1,5-dimethyl-6,7,8-trioxabicyclo[3.2.1]octane-2-carboxylate, 51

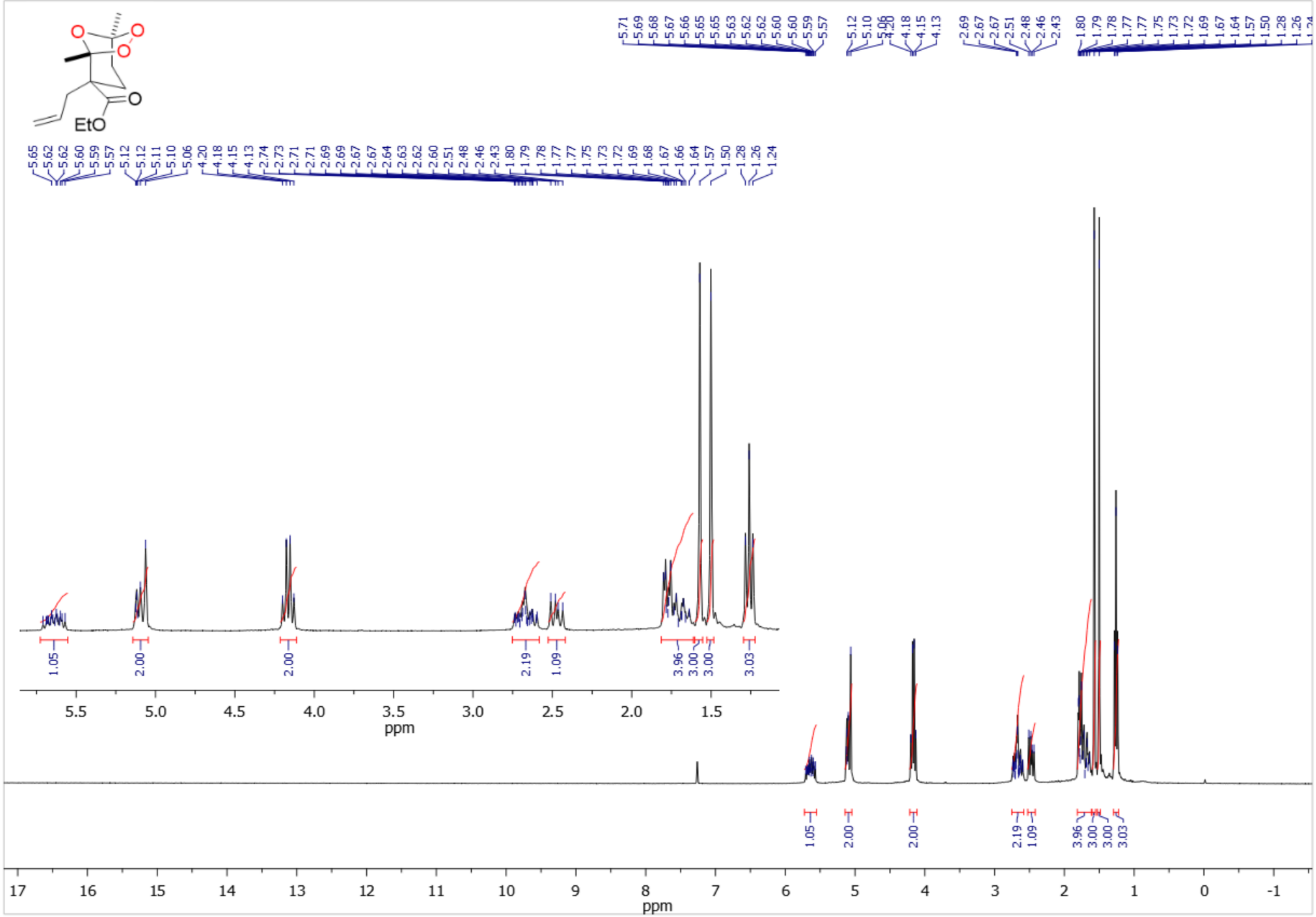

^{13}C NMR (75.48 MHz, $CDCl_3$). Ethyl (1*S**,2*R**,5*R**)-2-allyl-1,5-dimethyl-6,7,8-trioxabicyclo[3.2.1]octane-2-carboxylate, 51

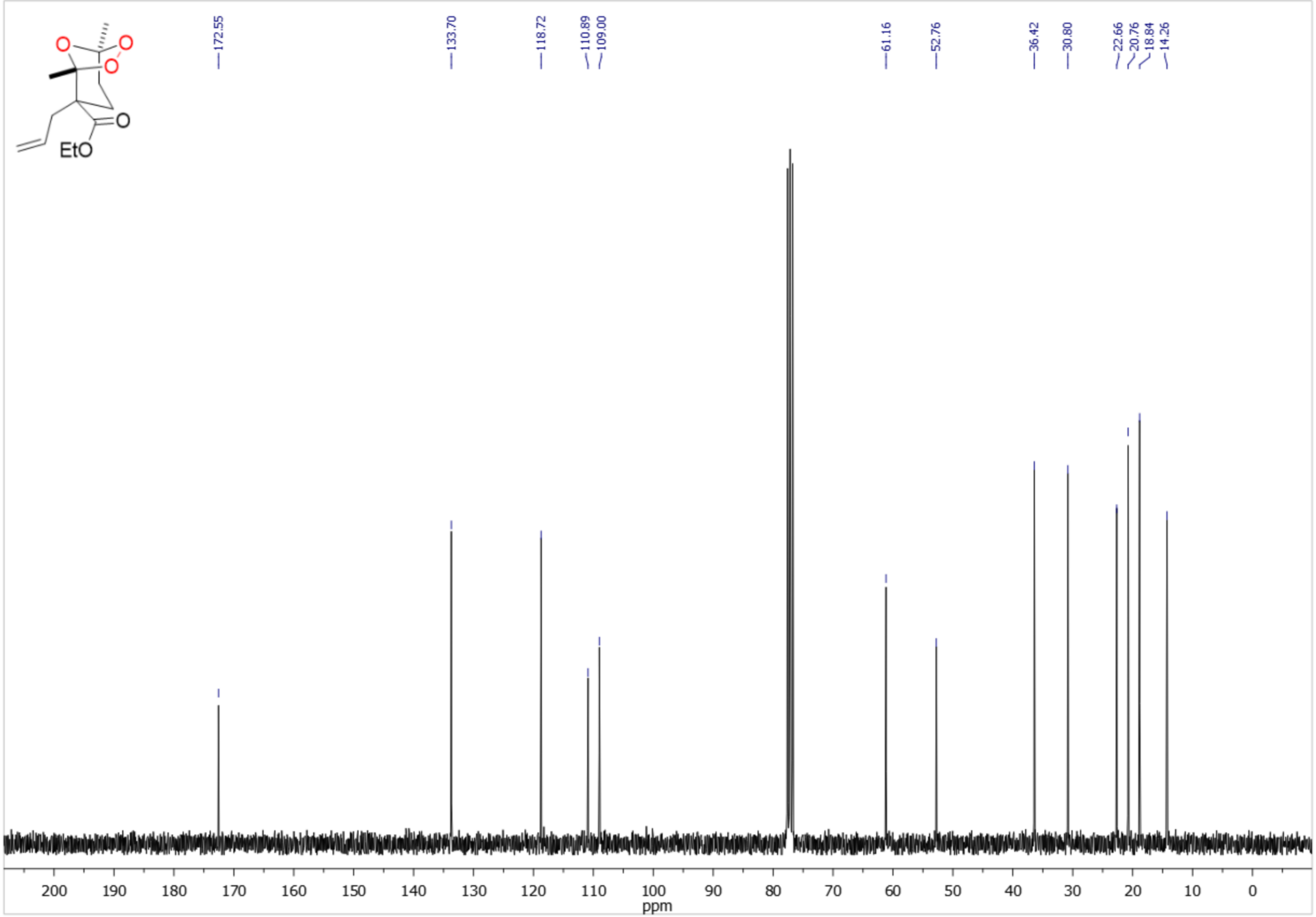

^{1}H NMR (300.13 MHz, CDCl3). Ethyl (1*R,2*S**,5*S**)-2-hexyl-1,5-dimethyl-6,7,8-trioxabicyclo[3.2.1]octane-2-carboxylate, 52**

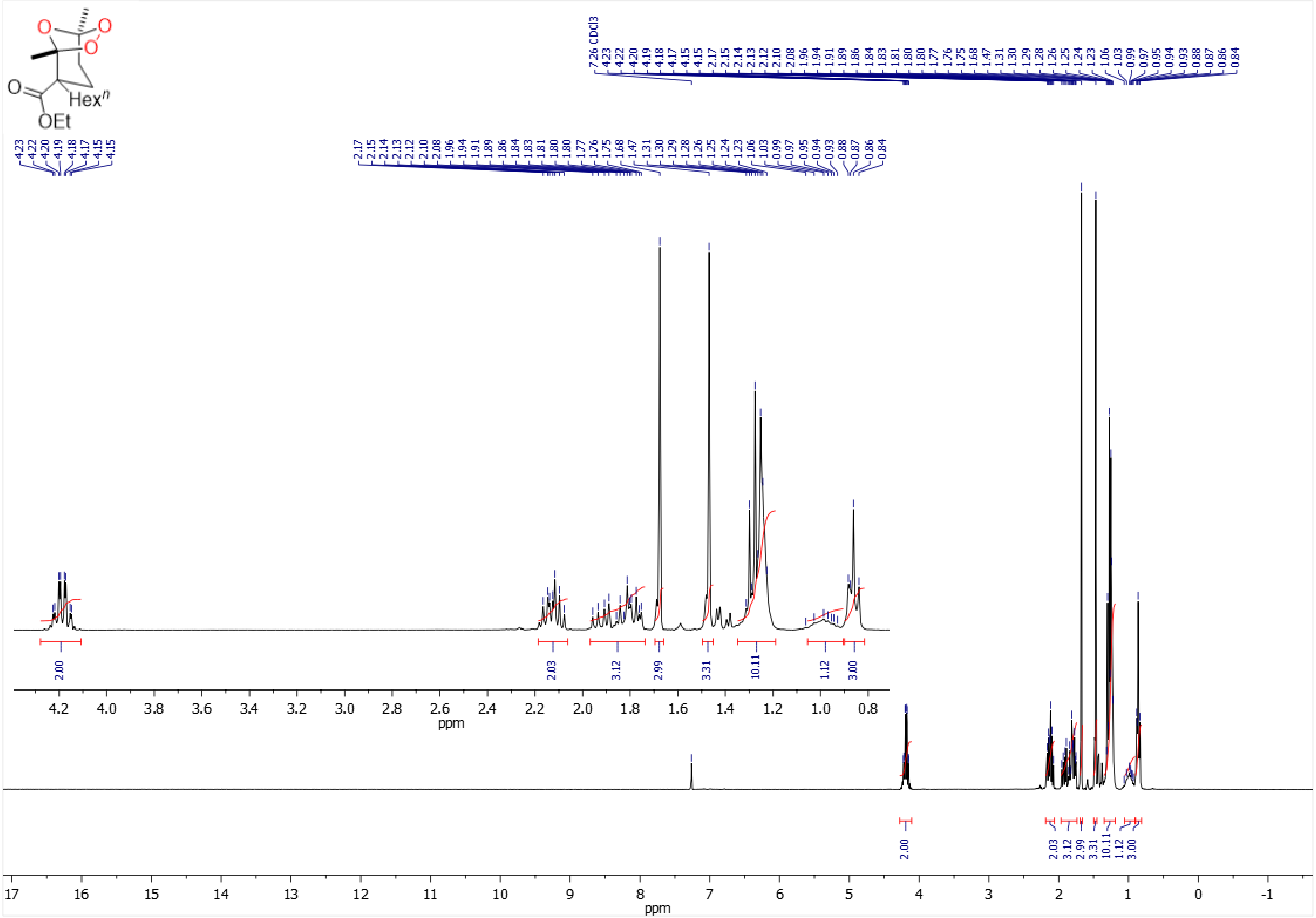

^{13}C NMR (75.48 MHz, CDCl$_3$). Ethyl (1*R**,2*S**,5*S**)-2-hexyl-1,5-dimethyl-6,7,8-trioxabicyclo[3.2.1]octane-2-carboxylate, 49a

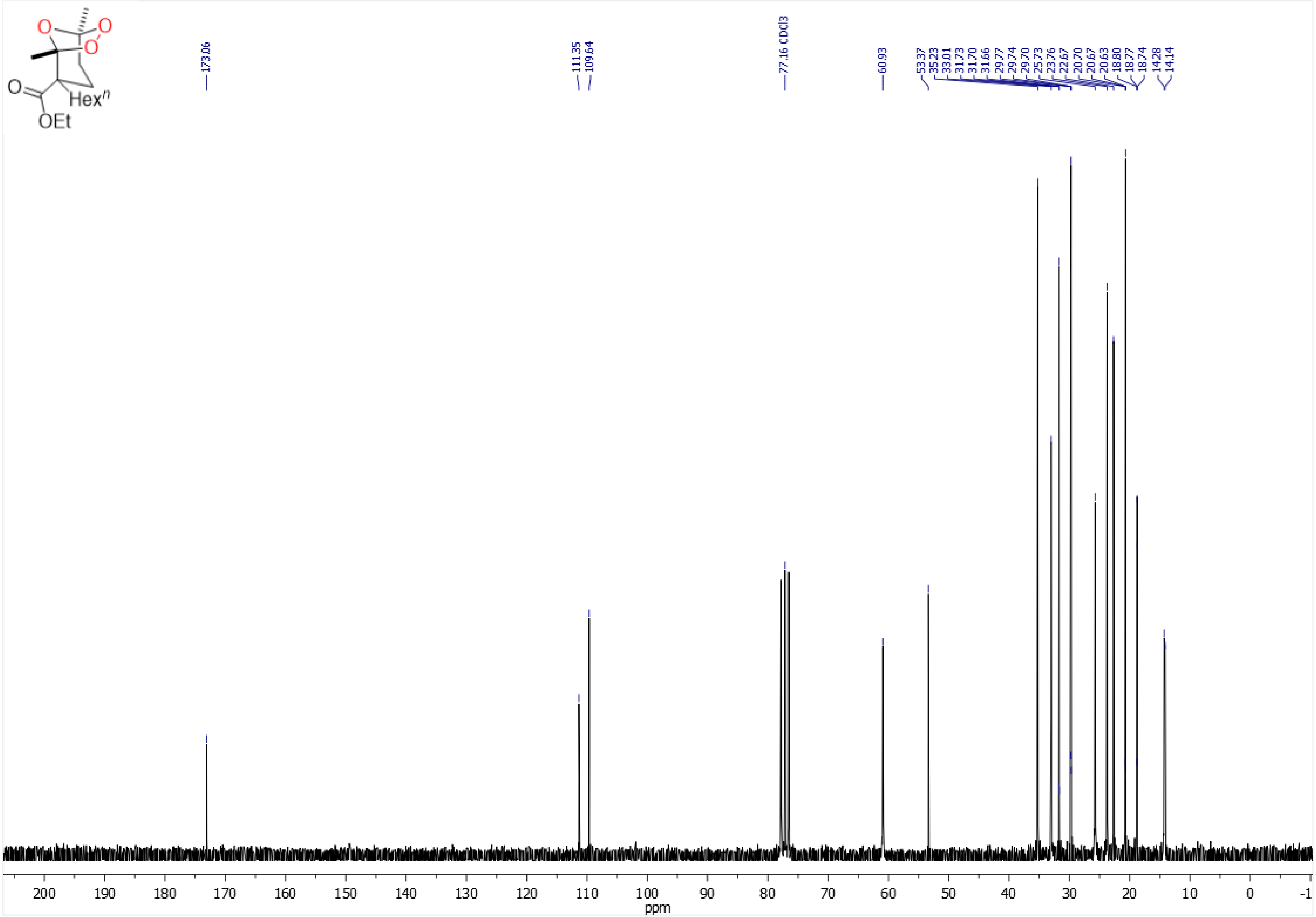

HRMS spectra of N-substituted azaozonides 1a+1b, 2a+2b, 8a', 8a'', 8b', 8b", 11, 12a+12b, 14, 16a+16b, 18a – 21a+18b –21b, 23a+23b, 26a – 28a+26b – 28b, 30

Ethyl 2-(4-fluorobenzyl)-1,5-dimethyl-6,7-dioxa-8-azabicyclo[3.2.1]octane-2-carboxylate, 1a+1b

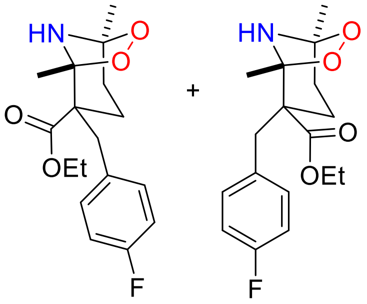

Display Report

Analysis Info

Analysis Name	D:\Data\Kolotyrkina\2020\Belyakova\0219005.d	Acquisition Date	19.02.2020 12:52:34
Method	tune_50-1600.m	Operator	BDAL@DE
Sample Name	/TERN BL-716	Instrument / Ser#	micrOTOF 10248
Comment	C17H22FNO4 mH 324.1605 alibrant added CH3CN		

Acquisition Parameter

Source Type	ESI	Ion Polarity	Positive	Set Nebulizer	1.0 Bar
Focus	Not active			Set Dry Heater	200 °C
Scan Begin	50 m/z	Set Capillary	4500 V	Set Dry Gas	4.0 l/min
Scan End	1600 m/z	Set End Plate Offset	-500 V	Set Divert Valve	Waste

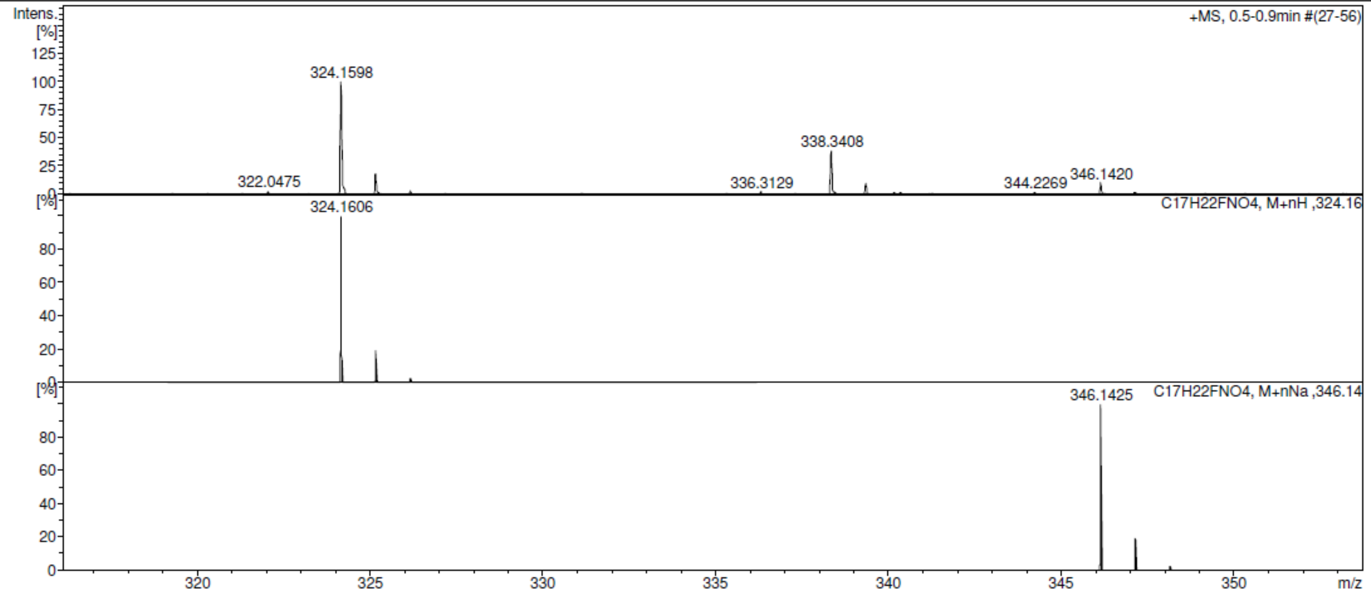

Bruker Compass DataAnalysis 4.0 printed: 19.02.2020 13:04:31 Page 1 of 1

Ethyl 2-decyl-1,5-dimethyl-6,7-dioxa-8-azabicyclo[3.2.1]octane-2-carboxylate, 2a+2b

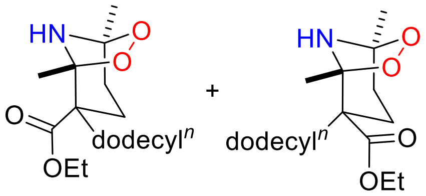

Display Report

Analysis Info			
Analysis Name	D:\Data\Kolotyrkina\2020\Belyakova\0206005.d	Acquisition Date	06.02.2020 12:42:13
Method	tune_50-1600.m	Operator	BDAL@DE
Sample Name	/TERN BL-698	Instrument / Ser#	micrOTOF 10248
Comment	C20H37NO4 mH 356.2795 calibrant added CH3CN		

Acquisition Parameter

Source Type	ESI	Ion Polarity	Positive	Set Nebulizer	1.0 Bar
Focus	Not active			Set Dry Heater	200 °C
Scan Begin	50 m/z	Set Capillary	4500 V	Set Dry Gas	4.0 l/min
Scan End	1600 m/z	Set End Plate Offset	-500 V	Set Divert Valve	Waste

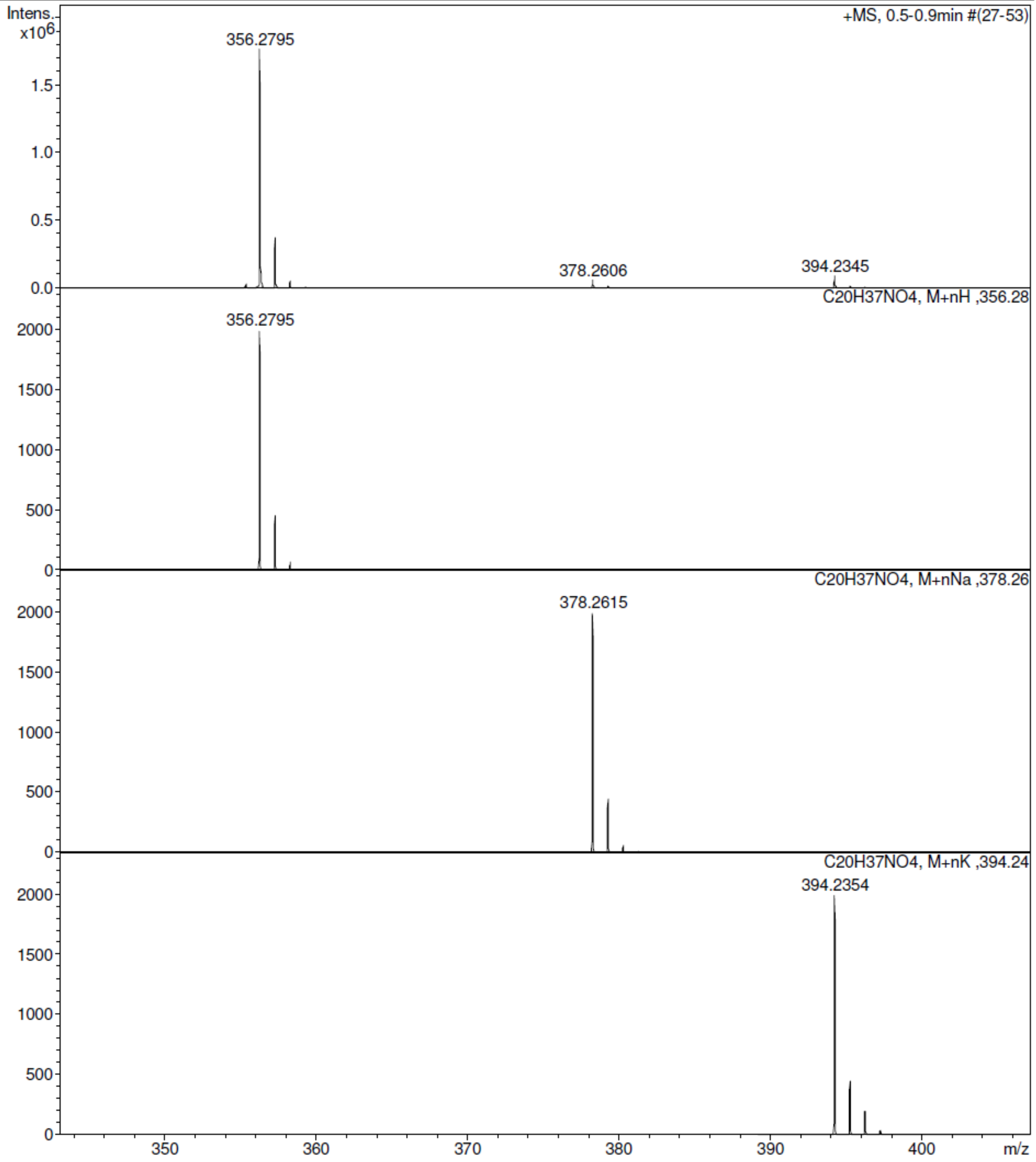

Bruker Compass DataAnalysis 4.0 printed: 06.02.2020 12:45:19 Page 1 of 1

1-ethoxy-1-oxopropan-2-yl -2-allyl-1,5-dimethyl-6,7-dioxa-8-azabicyclo[3.2.1]octane-2-carboxylate, 8a’, 8a’’, 8b’, 8b”

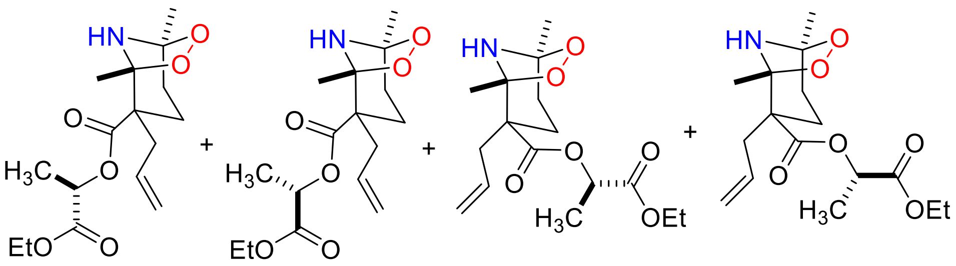

Display Report

Analysis Info

Analysis Name	D:\Data\Kolotyrkina\2020\Belyakova\0205009.d	Acquisition Date	05.02.2020 12:40:26
Method	tune_50-1600.m	Operator	BDAL@DE
Sample Name	/TERN BL-696	Instrument / Ser#	micrOTOF 10248
Comment	C16H25NO6 mH 328.1754 calibrant added CH3CN		

Acquisition Parameter

Source Type	ESI	Ion Polarity	Positive	Set Nebulizer	1.0 Bar
Focus	Not active			Set Dry Heater	200 °C
Scan Begin	50 m/z	Set Capillary	4500 V	Set Dry Gas	4.0 l/min
Scan End	1600 m/z	Set End Plate Offset	-500 V	Set Divert Valve	Waste

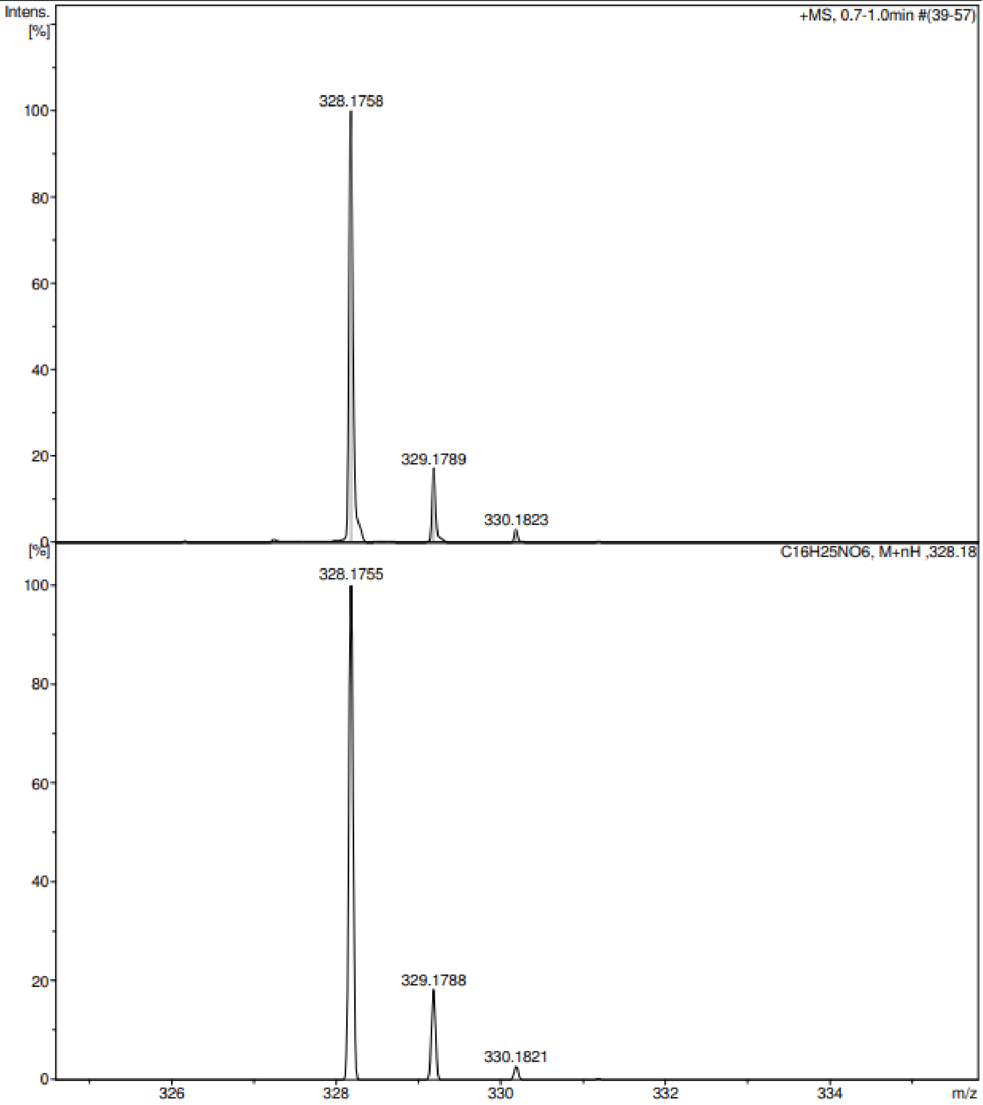

Bruker Compass DataAnalysis 4.0 printed: 05.02.2020 12:43:44 Page 1 of 1

Ethyl 2-(3-ethoxy-3-oxopropyl)-1,5-dimethyl-8-ureido-6,7-dioxa-8-azabicyclo[3.2.1]octane-2-carboxylate, 11a

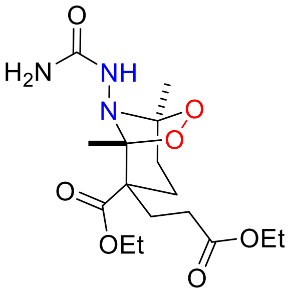

Display Report

Analysis Info

Analysis Name	D:\Data\Kolotyrkina\2019\Belyakova\1030010.d	Acquisition Date	30.10.2019 11:45:26
Method	tune_50-1600.m	Operator	BDAL@DE
Sample Name	/TERN BL-620	Instrument / Ser#	micrOTOF 10248
Comment	C16H27N3O7 mH 374.1921 calibrant added		

Acquisition Parameter

Source Type	ESI	Ion Polarity	Positive	Set Nebulizer	1.0 Bar
Focus	Not active			Set Dry Heater	200 °C
Scan Begin	50 m/z	Set Capillary	4500 V	Set Dry Gas	4.0 l/min
Scan End	1600 m/z	Set End Plate Offset	-500 V	Set Divert Valve	Waste

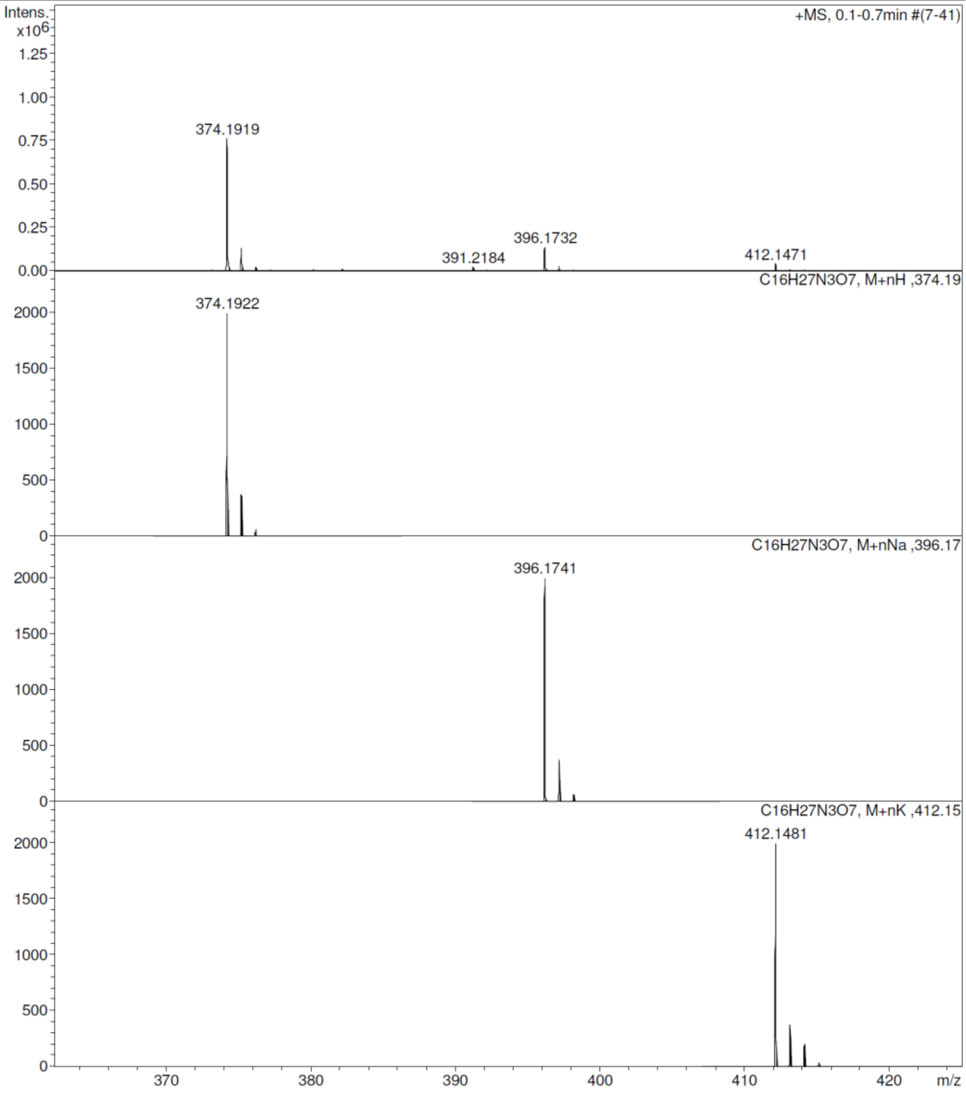

Bruker Compass DataAnalysis 4.0 printed: 30.10.2019 11:48:35 Page 1 of 1

Ethyl 2-(4-fluorobenzyl)-1,5-dimethyl-8-ureido-6,7-dioxa-8-azabicyclo[3.2.1]octane-2-carboxylate, 12a+12b

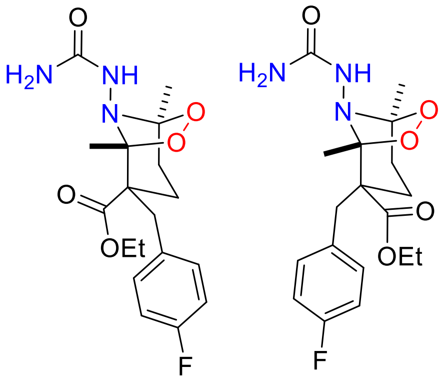

Display Report

Analysis Info

Analysis Name	D:\Data\Chizhov\Terentiev\Belyakova\bl-411_&clb.d	Acquisition Date	25.10.2019 13:53:13
Method	tune_wide.m	Operator	BDAL@DE
Sample Name	/TERN BL-411	Instrument / Ser#	micrOTOF 10248
Comment	CH3CN 100 %, dil. 200, calibrant added		

Acquisition Parameter

Source Type	ESI	Ion Polarity	Positive	Set Nebulizer	0.4 Bar
Focus	Not active			Set Dry Heater	180 °C
Scan Begin	50 m/z	Set Capillary	4500 V	Set Dry Gas	4.0 l/min
Scan End	3000 m/z	Set End Plate Offset	-500 V	Set Divert Valve	Waste

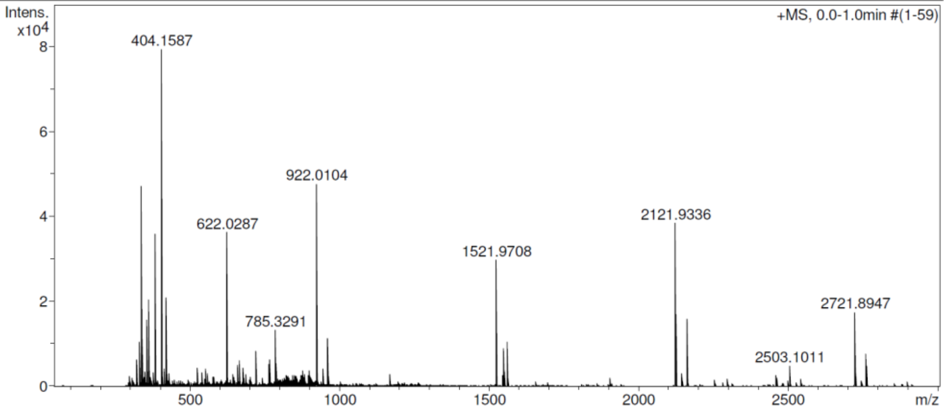

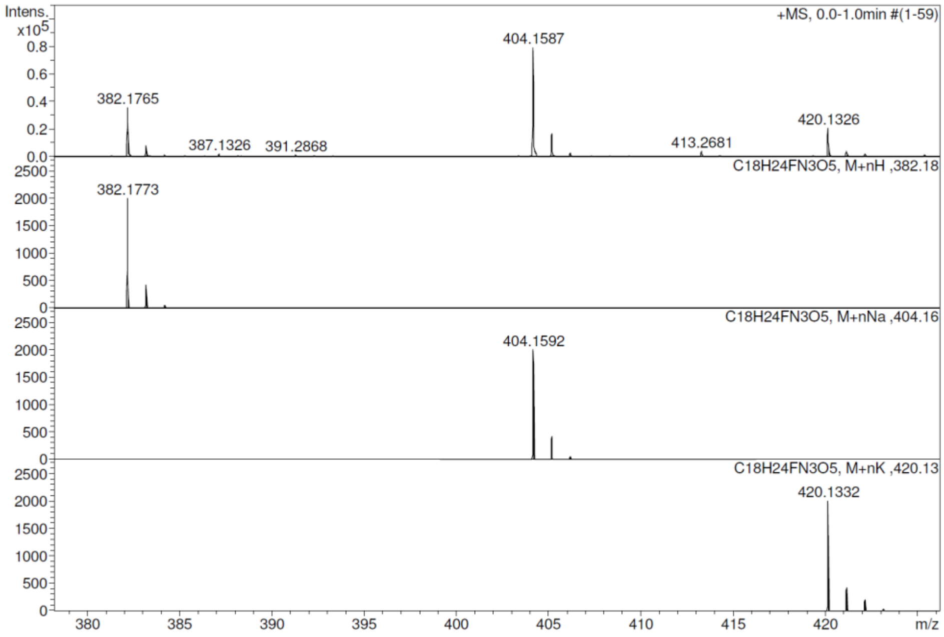

Bruker Compass DataAnalysis 4.0 printed: 25.10.2019 14:14:46 Page 1 of 1

Ethyl 2-benzyl-1,5-dimethyl-8-ureido-6,7-dioxa-8-azabicyclo[3.2.1]octane-2-carboxylate, 14

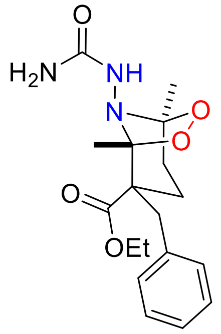

Display Report

**Analysis Info**		Acquisition Date	19.12.2019 13:20:58
Analysis Name	D:\Data\Kolotyrkina\2019\Belyakova\1219016.d		
Method	tune_50-1600.m	Operator	BDAL@DE
Sample Name	/TERN BL-597	Instrument / Ser#	micrOTOF 10248
Comment	C18H25N3O5 mH 364.1866 calibrant added CN3CN		

Acquisition Parameter

Source Type	ESI	Ion Polarity	Positive	Set Nebulizer	1.0 Bar
Focus	Not active			Set Dry Heater	200 °C
Scan Begin	50 m/z	Set Capillary	4500 V	Set Dry Gas	4.0 l/min
Scan End	1600 m/z	Set End Plate Offset	-500 V	Set Divert Valve	Waste

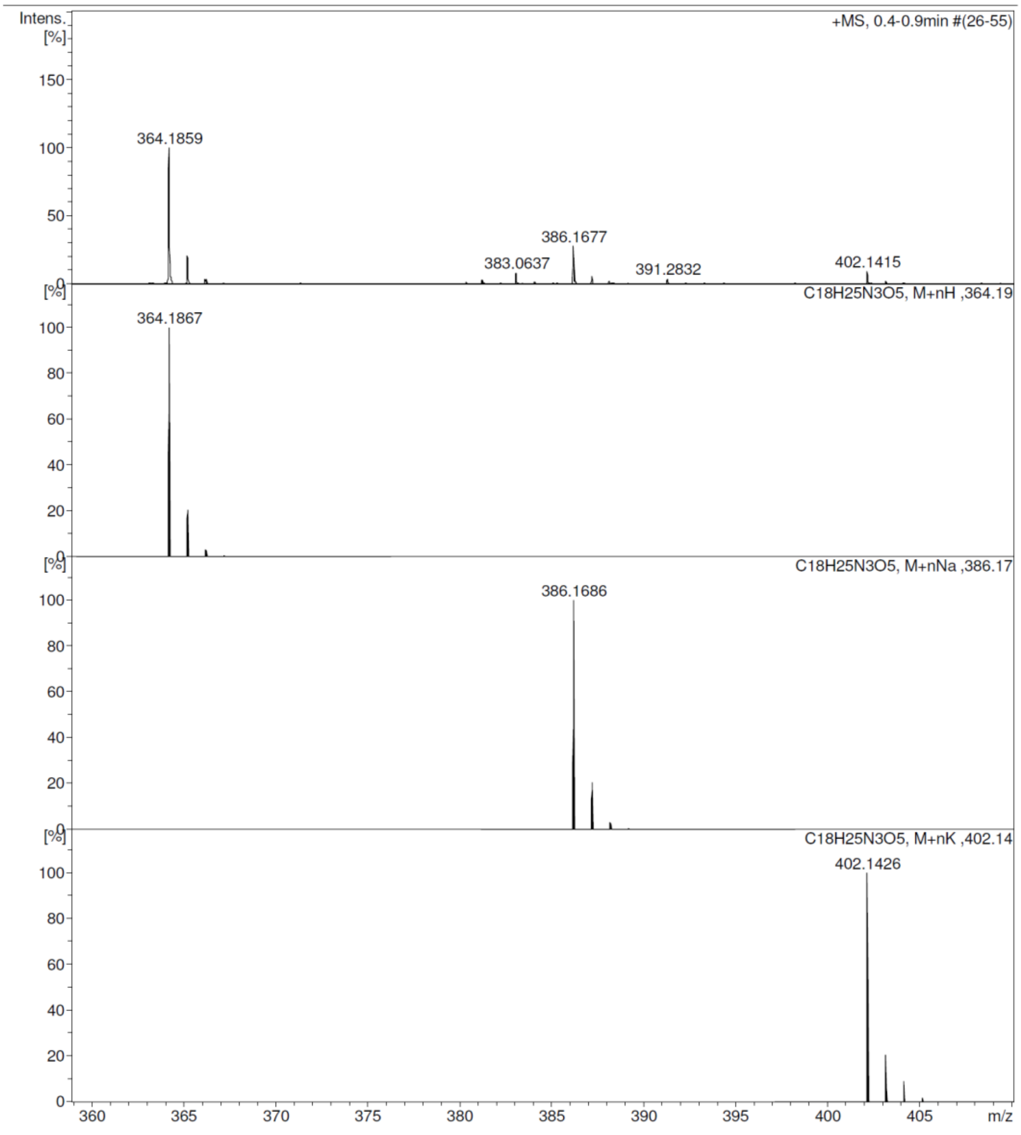

Bruker Compass DataAnalysis 4.0 printed: 19.12.2019 13:25:00 Page 1 of 1

Ethyl 8-(2-bromobenzamido)-2-ethyl-1,5-dimethyl-6,7-dioxa-8-azabicyclo[3.2.1]octane-2-carboxylate, 16a+16b

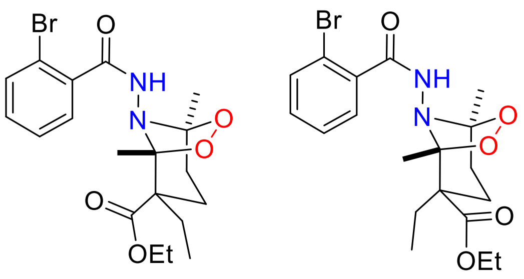

Display Report

Analysis Info

Analysis Name	D:\Data\Kolotyrkina\2019\Belyakova\12170016.d
Method	tune_50-1600.m
Sample Name	/TERN BL-610
Comment	C19H25BrN2O5 mH 441.1019 calibrant added
Acquisition Date	17.12.2019 14:42:28
Operator	BDAL@DE
Instrument / Ser#	micrOTOF 10248

Acquisition Parameter

Source Type	ESI	Ion Polarity	Positive	Set Nebulizer	1.0 Bar
Focus	Not active			Set Dry Heater	200 °C
Scan Begin	50 m/z	Set Capillary	4500 V	Set Dry Gas	4.0 l/min
Scan End	1600 m/z	Set End Plate Offset	-500 V	Set Divert Valve	Waste

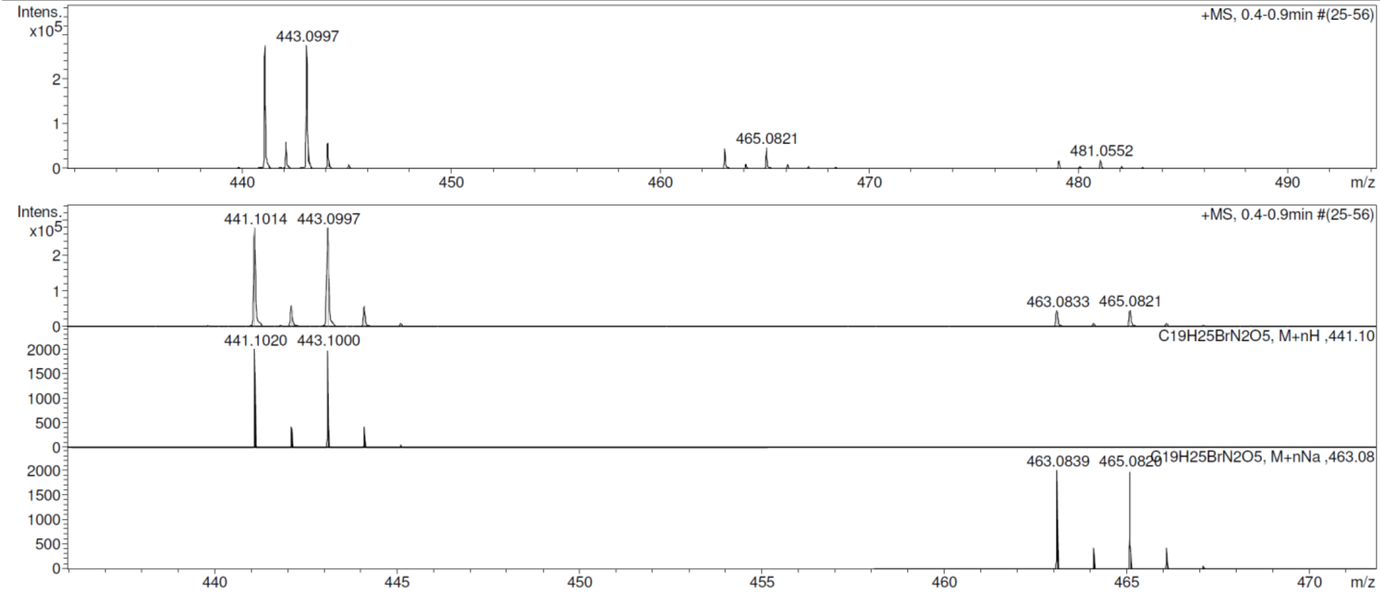

tert-Butyl 8-(2-bromobenzamido)-2-(4-bromobenzyl)-1,5-dimethyl-6,7-dioxa-8-azabicyclo[3.2.1]octane-2-carboxylate, 18a

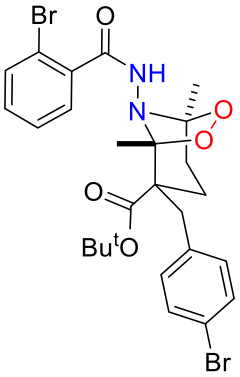

Display Report

Analysis Info

Analysis Name	D:\Data\Chizhov\Terentiev\Belyakova\bl-592_&clb.d	Acquisition Date	25.10.2019 14:05:40
Method	tune_wide.m	Operator	BDAL@DE
Sample Name	/TERN BL-592	Instrument / Ser#	micrOTOF 10248
Comment	CH3CN 100 %, dil. 200, calibrant added		

Acquisition Parameter

Source Type	ESI	Ion Polarity	Positive	Set Nebulizer	0.4 Bar
Focus	Not active			Set Dry Heater	180 °C
Scan Begin	50 m/z	Set Capillary	4500 V	Set Dry Gas	4.0 l/min
Scan End	3000 m/z	Set End Plate Offset	-500 V	Set Divert Valve	Waste

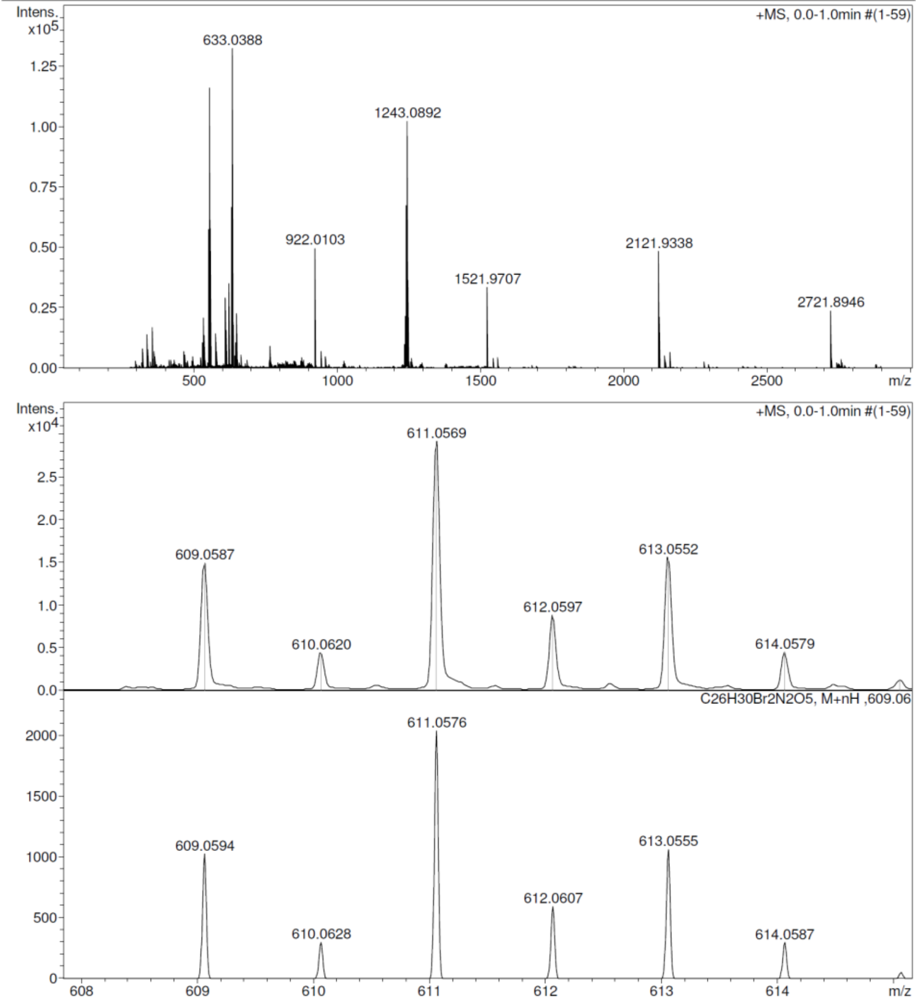

Bruker Compass DataAnalysis 4.0 printed: 25.10.2019 14:22:11 Page 1 of 1

Ethyl 8-(2-bromobenzamido)-2-(4-fluorobenzyl)-1,5-dimethyl-6,7-dioxa-8-azabicyclo[3.2.1]octane-2-carboxylate, 19a+19b

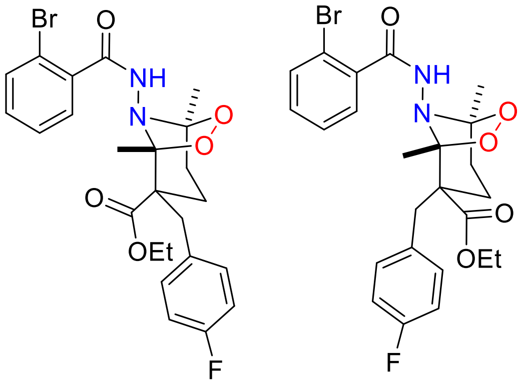

Display Report

Analysis Info

Analysis Name	D:\Data\Kolotyrkina\2019\Belyakova\1218025.d	Acquisition Date	18.12.2019 16:26:23
Method	tune_50-1600.m	Operator	BDAL@DE
Sample Name	/TERN BL-615	Instrument / Ser#	micrOTOF 10248
Comment	C24H26BrFN2O5 mH 521.1081 calibrant added CH3CN		

Acquisition Parameter

Source Type	ESI	Ion Polarity	Positive	Set Nebulizer	1.0 Bar
Focus	Not active			Set Dry Heater	200 °C
Scan Begin	50 m/z	Set Capillary	4500 V	Set Dry Gas	4.0 l/min
Scan End	1600 m/z	Set End Plate Offset	-500 V	Set Divert Valve	Waste

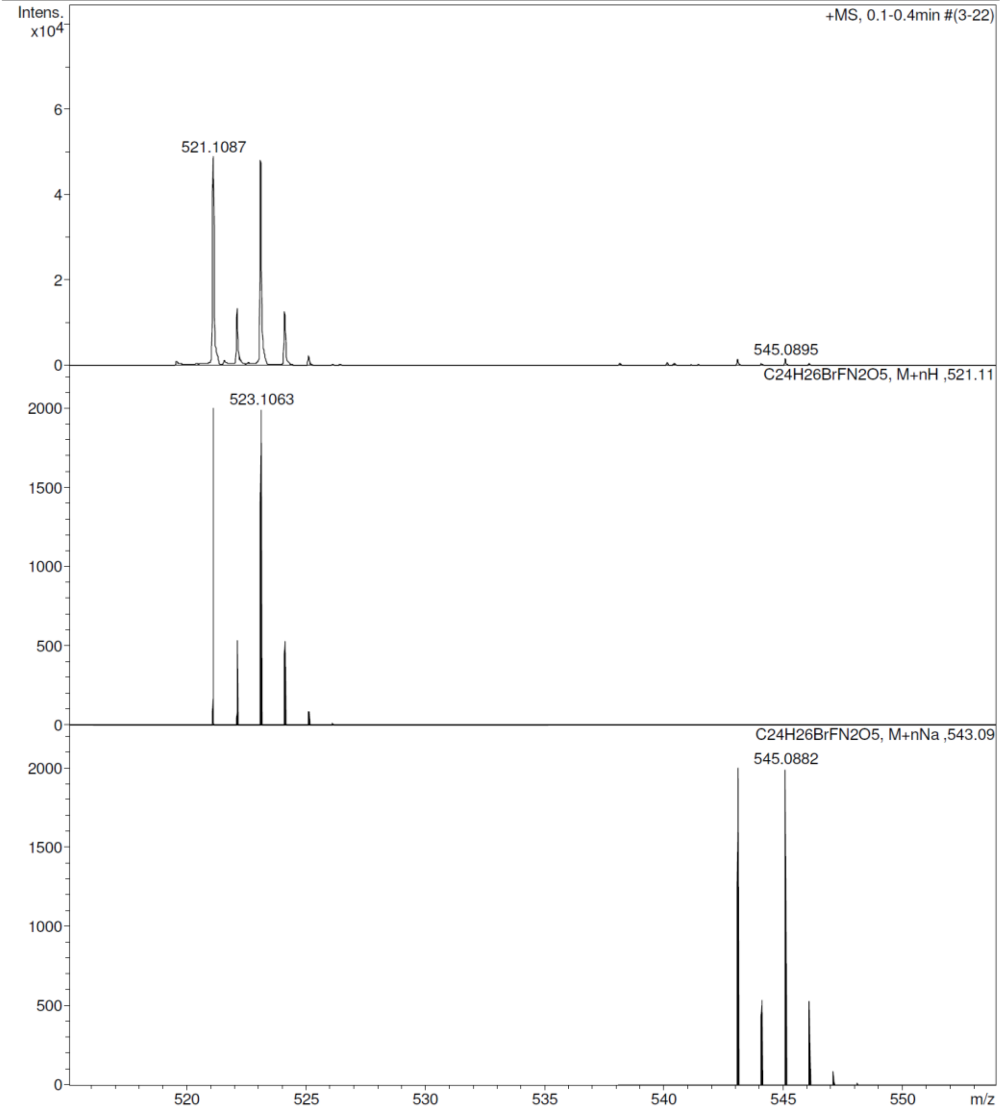

Ethyl 2-benzyl-8-(2-bromobenzamido)-1,5-dimethyl-6,7-dioxa-8-azabicyclo[3.2.1]octane-2-carboxylate, 20a+20b

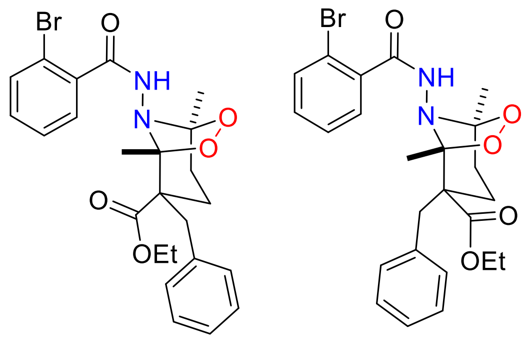

Display Report

Analysis Info

Analysis Name	D:\Data\Kolotyrkina\2019\Belyakova\12170014.d	Acquisition Date	17.12.2019 14:30:39
Method	tune_50-1600.m	Operator	BDAL@DE
Sample Name	/TERN BL-600	Instrument / Ser#	micrOTOF 10248
Comment	C24H27BrN2O5 mH 503.1176 calibrant added		

Acquisition Parameter

Source Type	ESI	Ion Polarity	Positive	Set Nebulizer	1.0 Bar
Focus	Not active			Set Dry Heater	200 °C
Scan Begin	50 m/z	Set Capillary	4500 V	Set Dry Gas	4.0 l/min
Scan End	1600 m/z	Set End Plate Offset	-500 V	Set Divert Valve	Waste

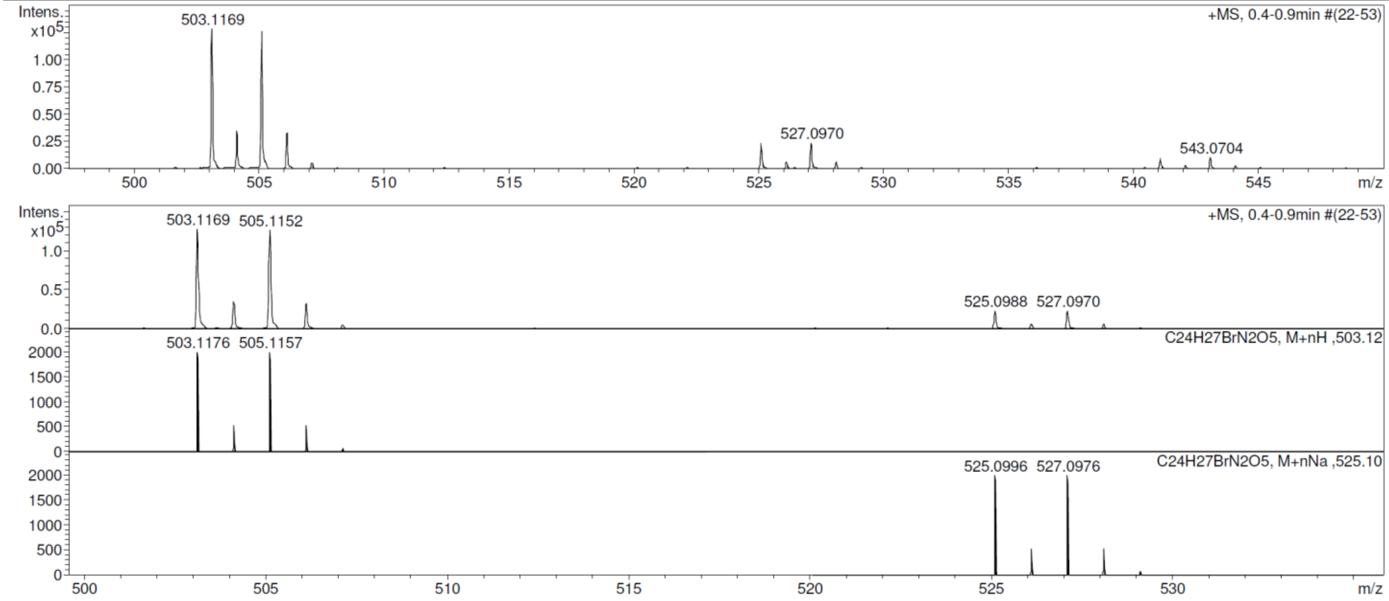

Benzyl 2-allyl-8-(2-bromobenzamido)-1,5-dimethyl-6,7-dioxa-8-azabicyclo[3.2.1]octane-2-carboxylate, 21a+21b

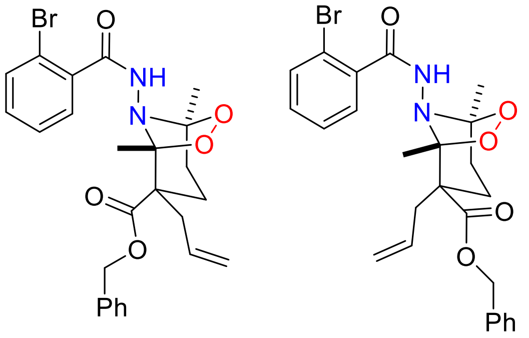

Display Report

Analysis Info

Analysis Name	D:\Data\Kolotyrkina\2019\Belyakova\12170017.d	Acquisition Date	17.12.2019 14:46:39
Method	tune_50-1600.m	Operator	BDAL@DE
Sample Name	/TERN BL-625	Instrument / Ser#	micrOTOF 10248
Comment	C25H27BrN2O5 mH 515.1176 calibrant added		

Acquisition Parameter

Source Type	ESI	Ion Polarity	Positive	Set Nebulizer	1.0 Bar
Focus	Not active			Set Dry Heater	200 °C
Scan Begin	50 m/z	Set Capillary	4500 V	Set Dry Gas	4.0 l/min
Scan End	1600 m/z	Set End Plate Offset	-500 V	Set Divert Valve	Waste

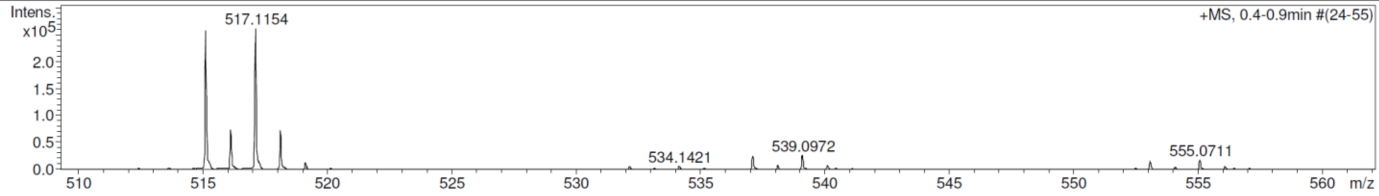

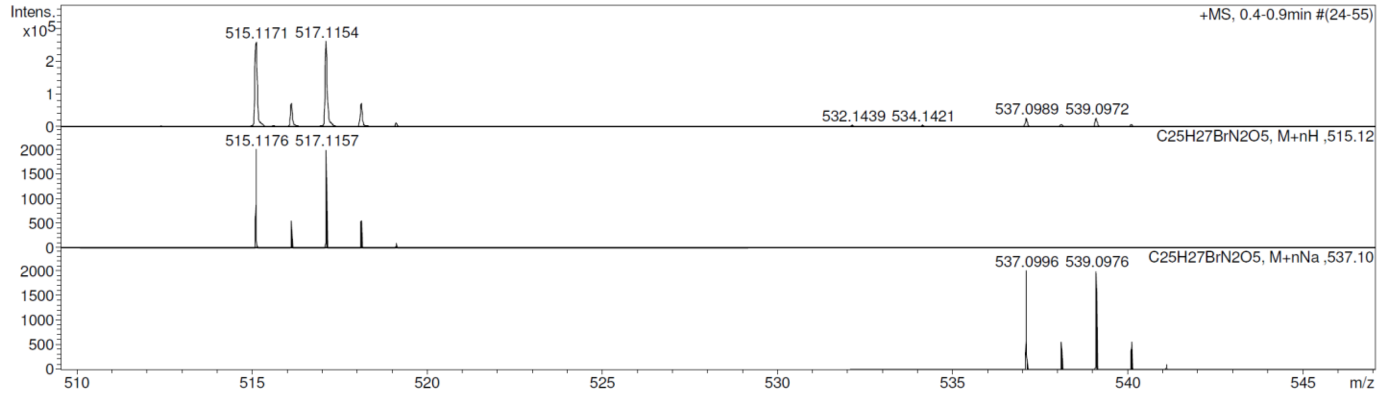

Ethyl 2-allyl-8-(2-bromobenzamido)-1,5-dimethyl-6,7-dioxa-8-azabicyclo[3.2.1]octane-2-carboxylate, 23a+23b

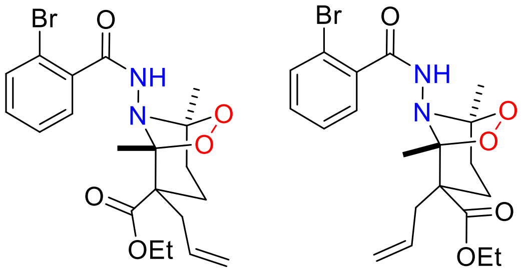

Display Report

Analysis Info

Analysis Name	D:\Data\Kolotyrkina\2019\Belyakova\1218026.d	Acquisition Date	18.12.2019 16:31:29
Method	tune_50-1600.m	Operator	BDAL@DE
Sample Name	/TERN BL-611	Instrument / Ser#	micrOTOF 10248
Comment	C20H25BrN2O5 mH 453.1019 calibrant added CH3CN		

Acquisition Parameter

Source Type	ESI	Ion Polarity	Positive	Set Nebulizer	1.0 Bar
Focus	Not active			Set Dry Heater	200 °C
Scan Begin	50 m/z	Set Capillary	4500 V	Set Dry Gas	4.0 l/min
Scan End	1600 m/z	Set End Plate Offset	-500 V	Set Divert Valve	Waste

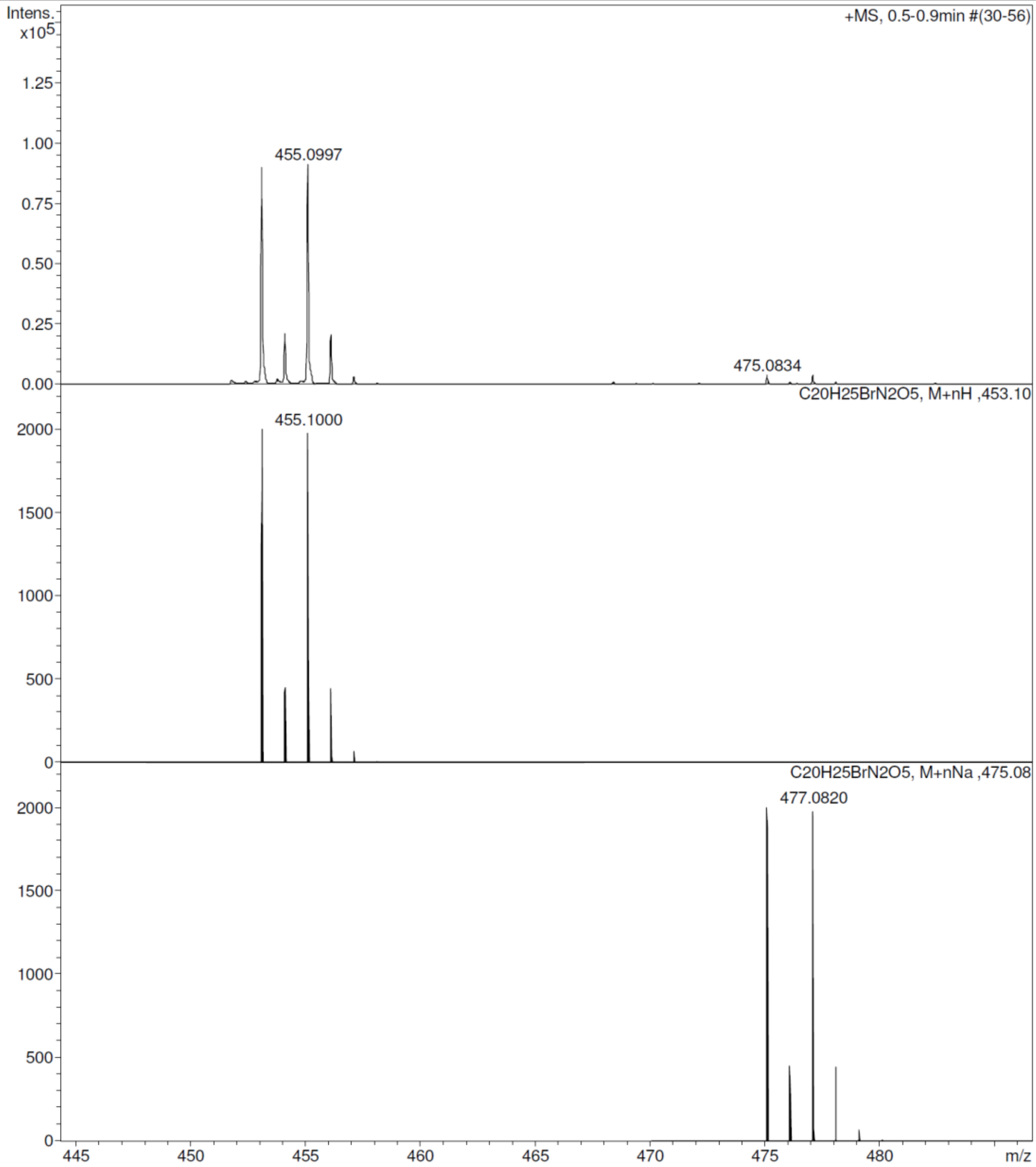

Ethyl 8-(2-cyanoacetamido)-2-(4-fluorobenzyl)-1,5-dimethyl-6,7-dioxa-8-azabicyclo[3.2.1]octane-2-carboxylate, 26a+26b

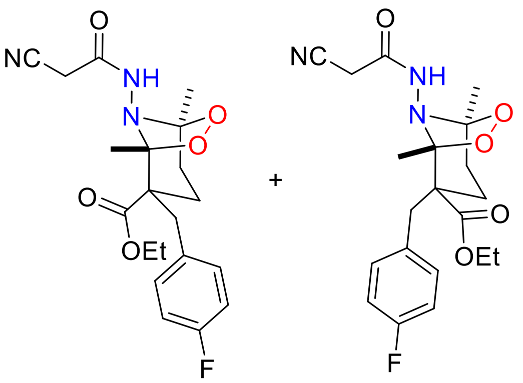

Display Report

Analysis Info

Analysis Name	D:\Data\Kolotyrkina\2019\Belyakova\1218022.d	Acquisition Date	18.12.2019 16:10:20
Method	tune_50-1600.m	Operator	BDAL@DE
Sample Name	/TERN BL-599	Instrument / Ser#	micrOTOF 10248
Comment	C20H24FN3O5 mH 406.1772 calibrant added CH3CN		

Acquisition Parameter

Source Type	ESI	Ion Polarity	Positive	Set Nebulizer	1.0 Bar
Focus	Not active			Set Dry Heater	200 °C
Scan Begin	50 m/z	Set Capillary	4500 V	Set Dry Gas	4.0 l/min
Scan End	1600 m/z	Set End Plate Offset	-500 V	Set Divert Valve	Waste

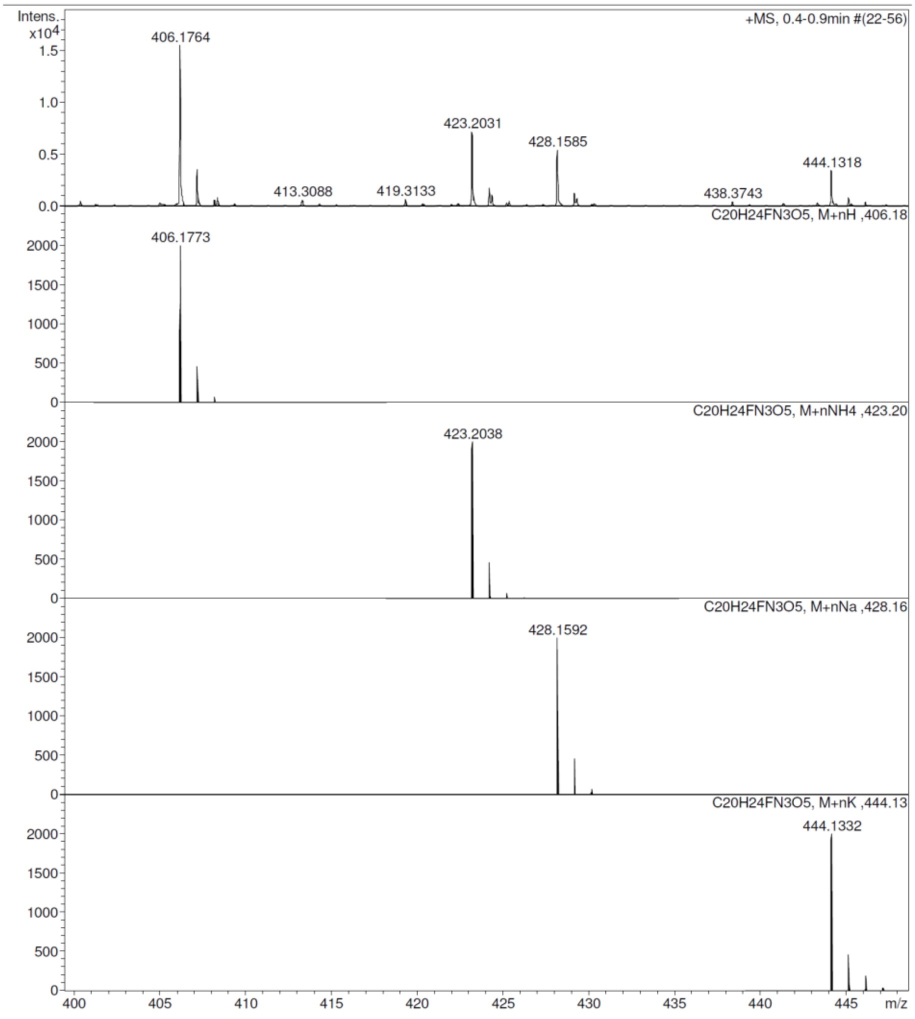

Bruker Compass DataAnalysis 4.0 printed: 18.12.2019 16:14:59 Page 1 of 1

Ethyl 2-benzyl-8-(2-cyanoacetamido)-1,5-dimethyl-6,7-dioxa-8-azabicyclo[3.2.1]octane-2-carboxylate, 27a+27b

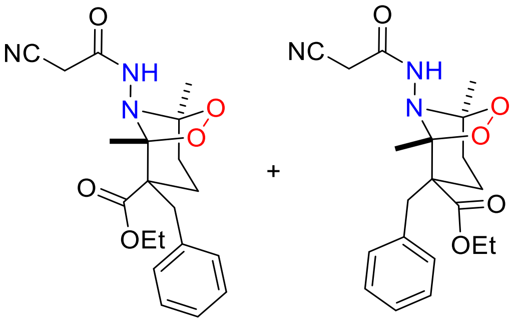

Display Report

Analysis Info

Analysis Name	D:\Data\Kolotyrkina\2019\Belyakova\1030009.d	Acquisition Date	30.10.2019 11:38:05
Method	tune_50-1600.m	Operator	BDAL@DE
Sample Name	/TERN BL-602	Instrument / Ser#	micrOTOF 10248
Comment	C20H25N3O5 mH 388.1867 calibrant added		

Acquisition Parameter

Source Type	ESI	Ion Polarity	Positive	Set Nebulizer	1.0 Bar
Focus	Not active			Set Dry Heater	200 °C
Scan Begin	50 m/z	Set Capillary	4500 V	Set Dry Gas	4.0 l/min
Scan End	1600 m/z	Set End Plate Offset	-500 V	Set Divert Valve	Waste

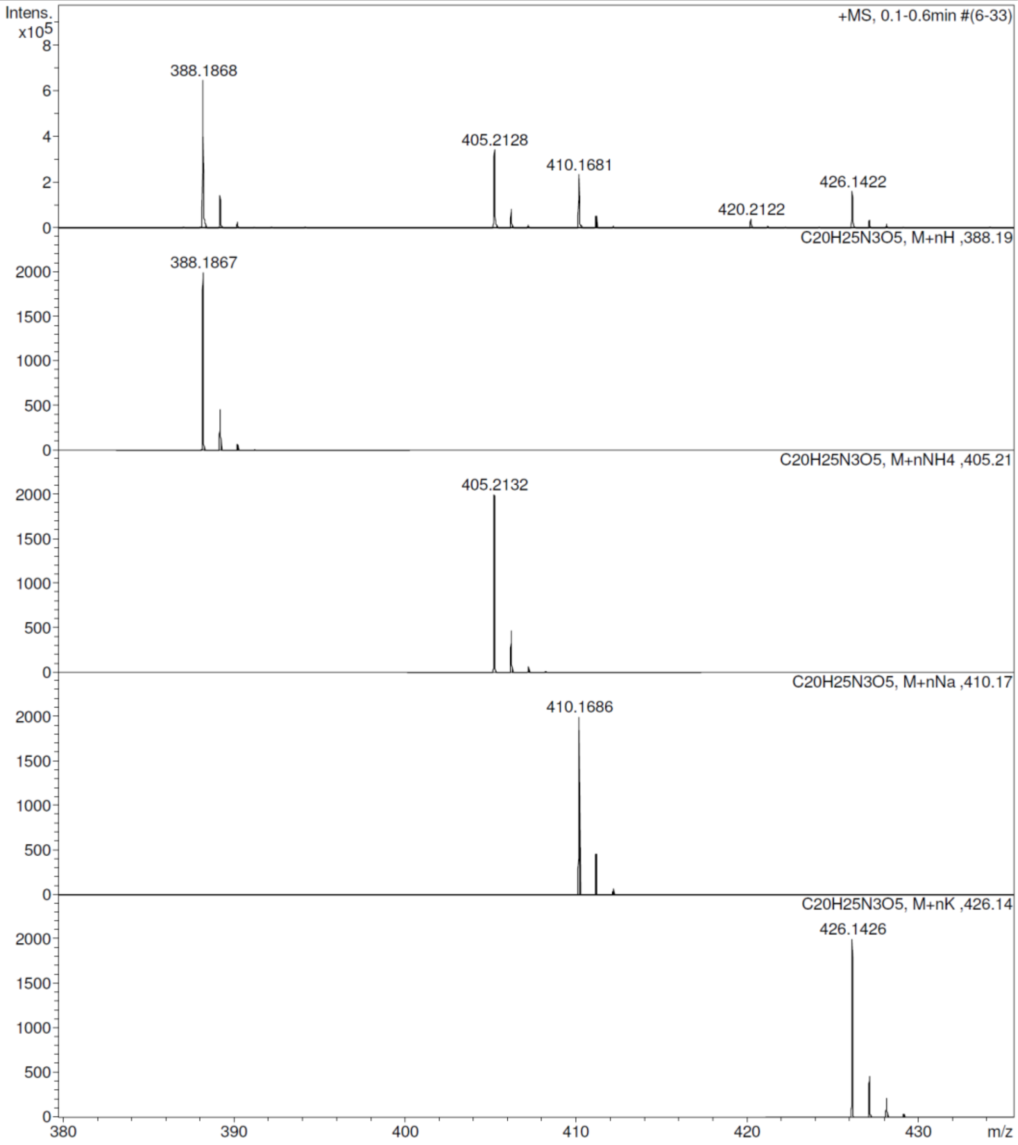

Allyl 2-allyl-8-(2-cyanoacetamido)-1,5-dimethyl-6,7-dioxa-8-azabicyclo[3.2.1]octane-2-carboxylate, 28a+28b

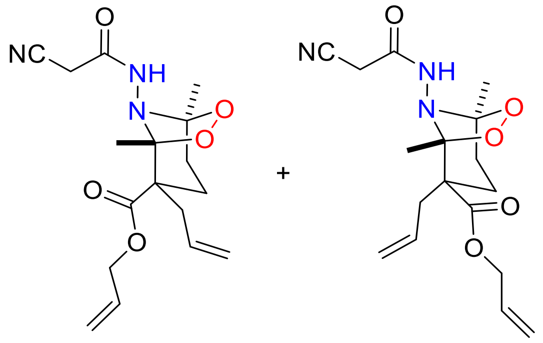

Display Report

Analysis Info

Analysis Name	D:\Data\Kolotyrkina\2019\Belyakova\1219017.d
Method	tune_50-1600.m
Sample Name	/TERN BL-606
Comment	C17H23N3O5 mH 350.1710 calibrant added CN3CN
Acquisition Date	19.12.2019 13:26:48
Operator	BDAL@DE
Instrument / Ser#	micrOTOF 10248

Acquisition Parameter

Source Type	ESI	Ion Polarity	Positive	Set Nebulizer	1.0 Bar
Focus	Not active			Set Dry Heater	200 °C
Scan Begin	50 m/z	Set Capillary	4500 V	Set Dry Gas	4.0 l/min
Scan End	1600 m/z	Set End Plate Offset	-500 V	Set Divert Valve	Waste

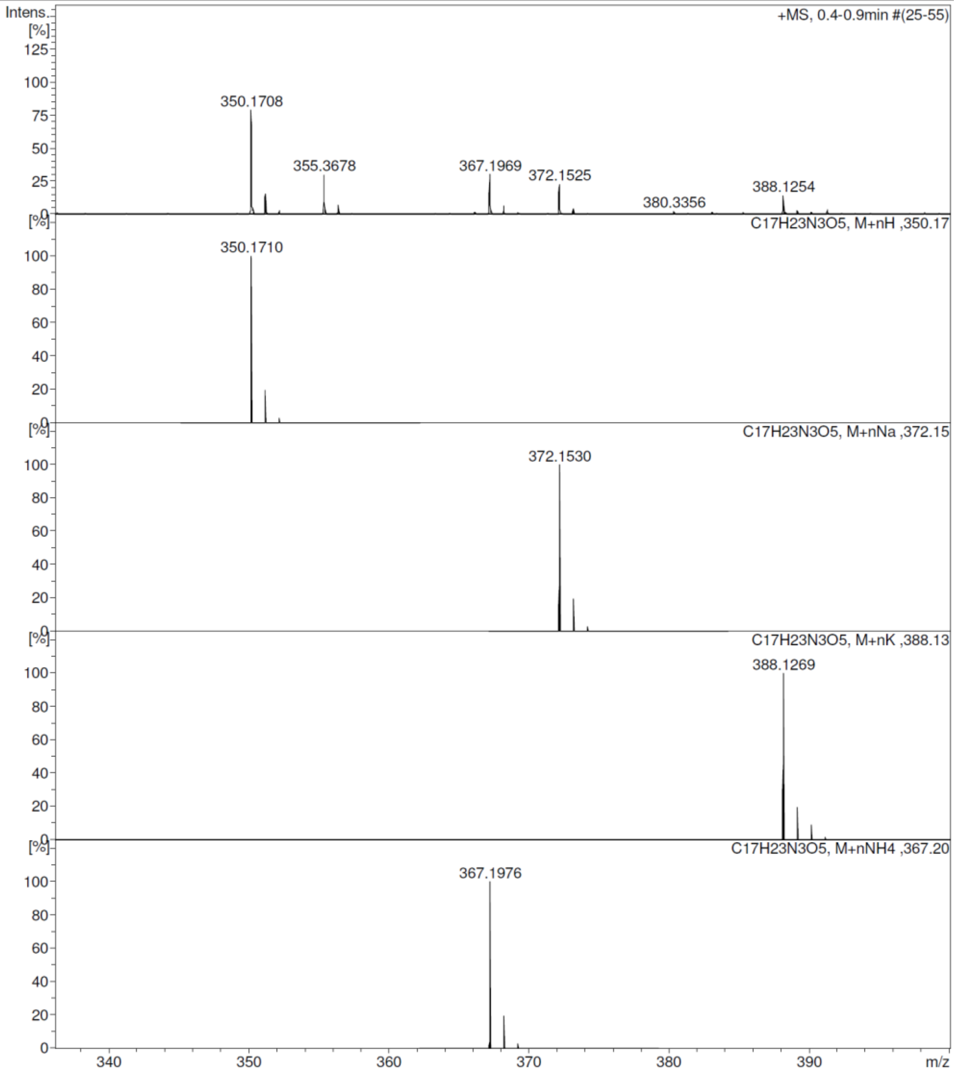

Bruker Compass DataAnalysis 4.0 printed: 19.12.2019 13:29:49 Page 1 of 1

Ethyl 2-(4-chlorobenzyl)-1,5-dimethyl-8-((4-methylphenyl)sulfonamido)-6,7-dioxa-8-azabicyclo[3.2.1]octane-2-carboxylate, 29

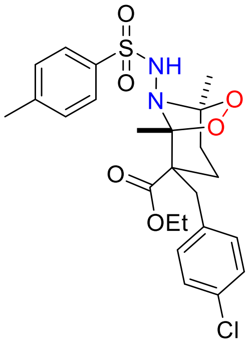

Display Report

Analysis Info

Analysis Name D:\Data\Chizhov\Terentiev\Radulov\bl-587-1_&clblow.d
Method tune_low.m
Sample Name /TERN BL-587-1
Comment CH3CN 100 %, dil. 200, calibrant added

Acquisition Date 27.07.2023 11:37:46

Operator BDAL@DE
Instrument / Ser# micrOTOF 10248

Acquisition Parameter

Source Type	ESI	Ion Polarity	Positive	Set Nebulizer	0.4 Bar
Focus	Not active			Set Dry Heater	180 °C
Scan Begin	50 m/z	Set Capillary	4500 V	Set Dry Gas	4.0 l/min
Scan End	2000 m/z	Set End Plate Offset	-500 V	Set Divert Valve	Waste

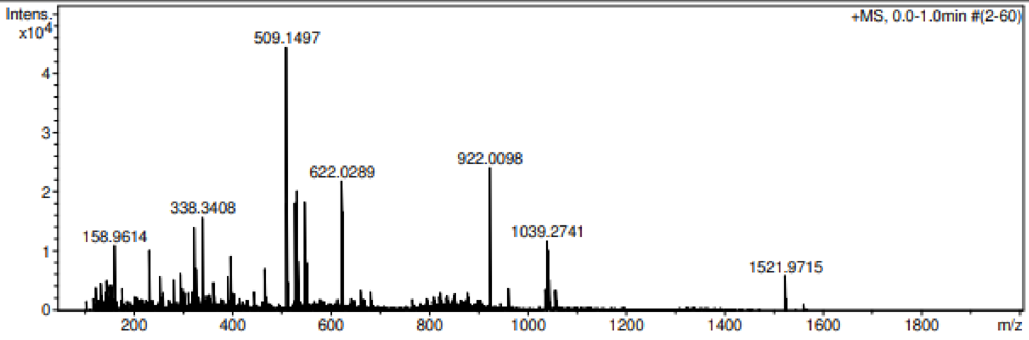

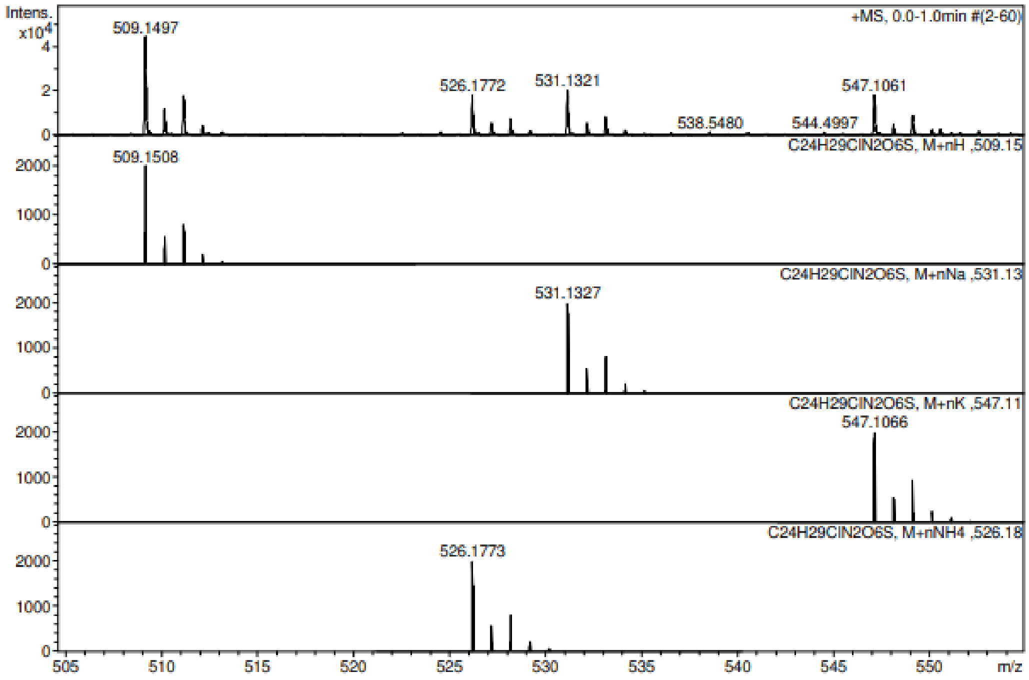

Bruker Compass DataAnalysis 4.0 printed: 27.07.2023 11:55:38 Page 1 of 1

Ethyl 2-(4-fluorobenzyl)-1,5-dimethyl-8-((4-methylphenyl)sulfonamido)-6,7-dioxa-8-azabicyclo[3.2.1]octane-2-carboxylate, 30

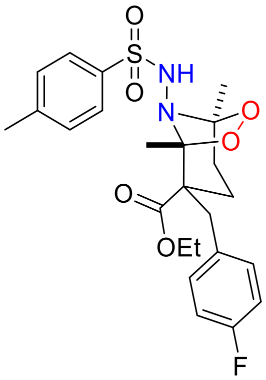

Display Report

Analysis Info

Analysis Name	D:\Data\Chizhov\Terentiev\Belyakova\bl-601_&clb.d	Acquisition Date	28.10.2019 13:38:41
Method	tune_wide.m	Operator	BDAL@DE
Sample Name	/TERN BL-601	Instrument / Ser#	micrOTOF 10248
Comment	CH3CN 100 %, dil. 200, calibrant added		

Acquisition Parameter

Source Type	ESI	Ion Polarity	Positive	Set Nebulizer	0.4 Bar
Focus	Not active			Set Dry Heater	180 °C
Scan Begin	50 m/z	Set Capillary	4500 V	Set Dry Gas	4.0 l/min
Scan End	3000 m/z	Set End Plate Offset	-500 V	Set Divert Valve	Waste

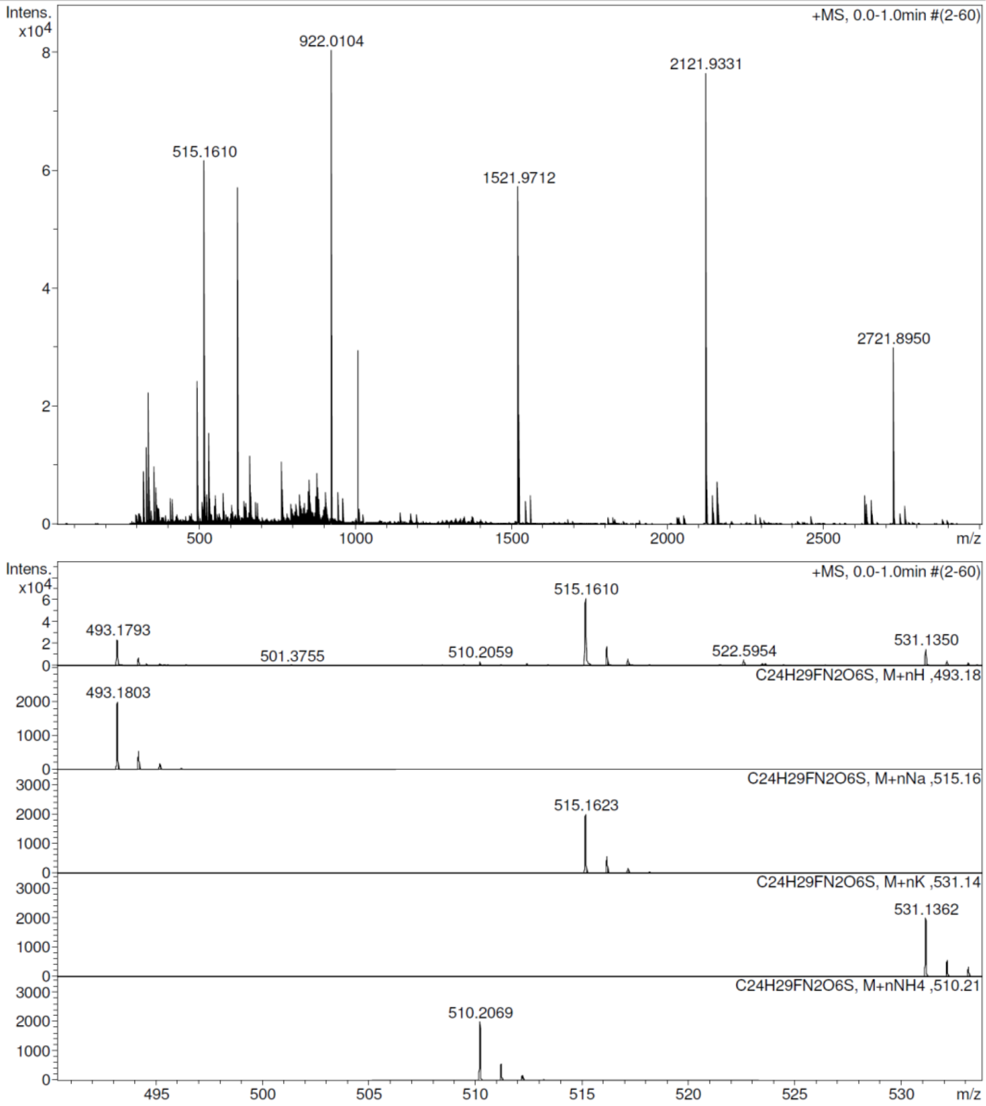

HPLC spectrum of N-substituted azaozonides 21a+21b

after storage for 2 years at -22°C

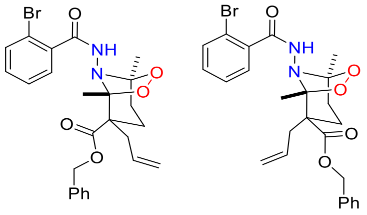

SHIMADZU LabSolutions

Analysis Report

<Sample Information>

Sample Name : Ivan_BL625
Sample ID : Ivan_BL625
Data Filename : Ivan_BL625_1.lcd
Method Filename : multiwave.lcm
Batch Filename :
Vial # : 1-96
Injection Volume : 5 uL
Date Acquired :
Date Processed :

Sample Type : Unknown
Acquired by : PZ
Processed by : PZ

<Chromatogram>

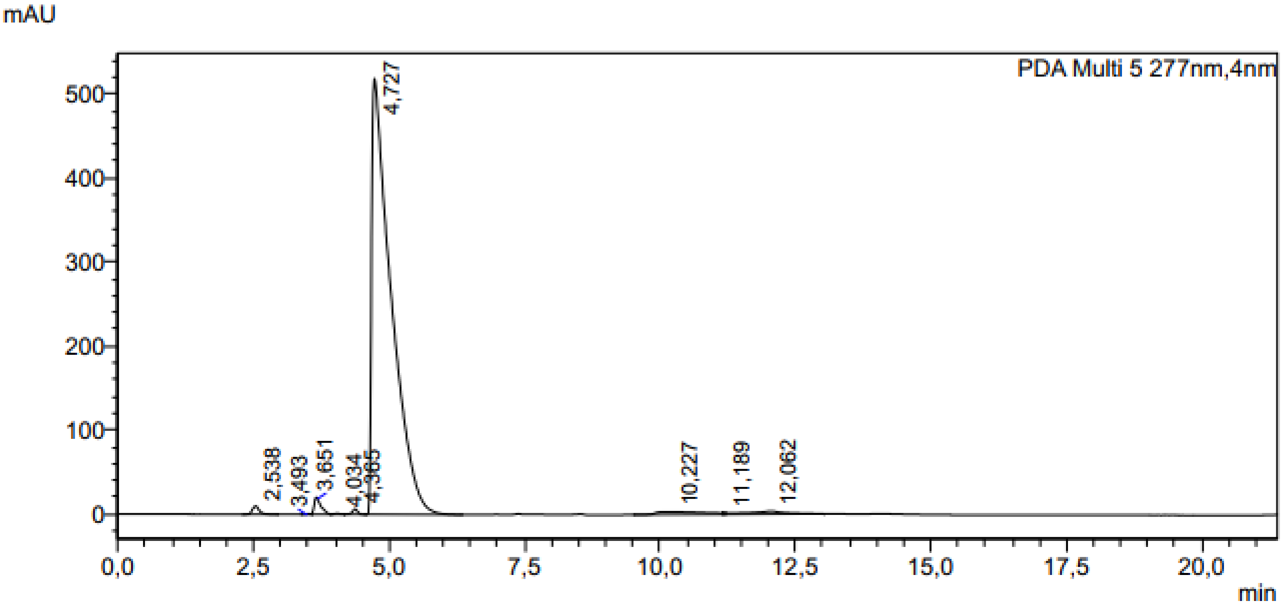

<Peak Table>

PDA Ch5 277nm

Peak#	Ret. Time	Area	Height	Conc.	Unit	Mark	Name
1	2,538	86199	9945	0,631		V	
2	3,493	4791	812	0,035			
3	3,651	177976	20362	1,303		V	
4	4,034	10521	1396	0,077		V	
5	4,365	46791	6608	0,343		V	
6	4,727	12960619	518775	94,875			
7	10,227	206606	3330	1,512			
8	11,189	2914	1538	0,021		V	
9	12,062	164257	3511	1,202		V	
Total		13660674	566278				

HPLC spectrum of N-substituted azaozonide 29 after storage for 2 years at -22°C

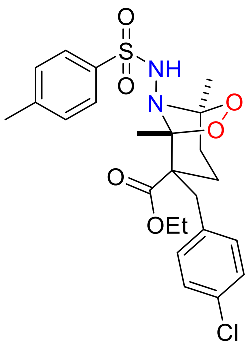

SHIMADZU LabSolutions

Analysis Report

<Sample Information>

Sample Name	: Ivan_BL499		
Sample ID	: Ivan_BL499		
Data Filename	: Ivan_BL499_1.lcd		
Method Filename	: multiwave.lcm		
Batch Filename	:		
Vial #	: 1-97	Sample Type	: Unknown
Injection Volume	: 5 uL		
Date Acquired	:	Acquired by	: PZ
Date Processed	:	Processed by	: PZ

<Chromatogram>

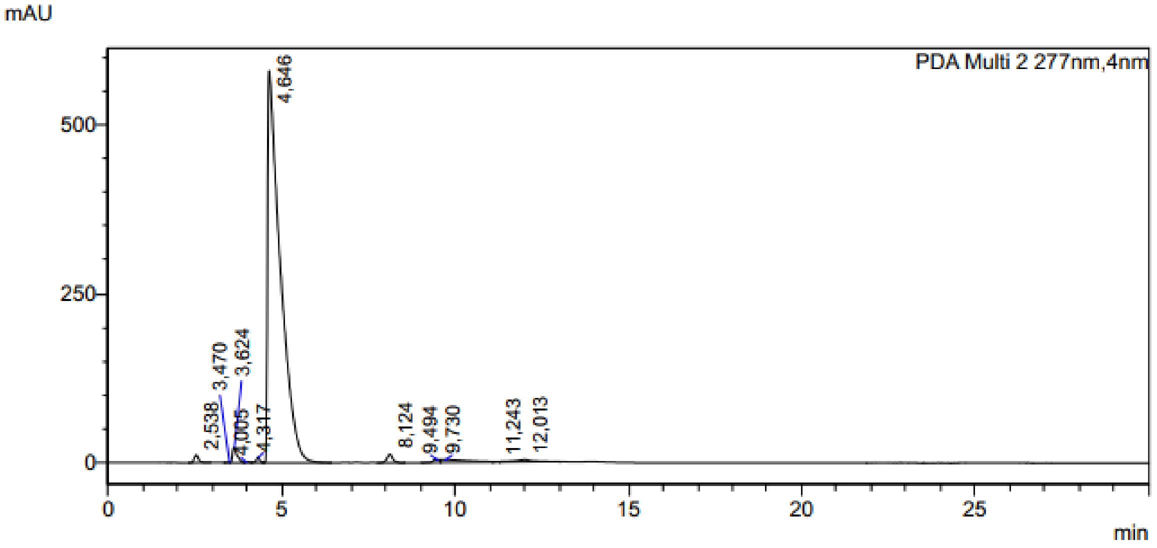

<Peak Table>

PDA Ch2 277nm

Peak#	Ret. Time	Area	Height	Conc.	Unit	Mark	Name
1	2,538	96055	11209	0,609			
2	3,470	5388	945	0,034			
3	3,624	204481	22702	1,297		V	
4	4,005	13510	1707	0,086		V	
5	4,317	55710	7979	0,353		V	
6	4,646	14805235	580181	93,884		V	
7	8,124	145625	12405	0,923			
8	9,494	69590	4535	0,441		V	
9	9,730	234784	4425	1,489		V	
10	11,243	8675	918	0,055		V	
11	12,013	130676	3591	0,829		V	
Total		15769730	650596				

1. Gomes, G. D.; Yaremenko, I. A.; Radulov, P. S.; Novikov, R. A.; Chernyshev, V. V.; Korlyukov, A. A.; Nikishin, G. I.; Alabugin, I. V.; Terent'ev, A. O., Stereoelectronic Control in the Ozone-Free Synthesis of Ozonides. *Angew Chem Int Edit* **2017,** *56* (18), 4955-4959.
2. Coghi, P.; Yaremenko, I. A.; Prommana, P.; Radulov, P. S.; Syroeshkin, M. A.; Wu, Y. J.; Gao, J. Y.; Gordillo, F. M.; Mok, S.; Wong, V. K. W.; Uthaipibull, C.; Terent'ev, A. O., Novel Peroxides as Promising Anticancer Agents with Unexpected Depressed Antimalarial Activity. *ChemMedChem* **2018,** *13* (9), 902-908.
3. Yaremenko, I. A.; Radulov, P. S.; Belyakova, Y. Y.; Demina, A. A.; Fomenkov, D. I.; Barsukov, D. V.; Subbotina, I. R.; Fleury, F.; Terent'ev, A. O., Catalyst Development for the Synthesis of Ozonides and Tetraoxanes Under Heterogeneous Conditions: Disclosure of an Unprecedented Class of Fungicides for Agricultural Application. *Chem-Eur J* **2020,** *26* (21), 4734-4751.
4. Yaremenko, I. A.; Coghi, P.; Prommana, P.; Qiu, C.; Radulov, P. S.; Qu, Y.; Belyakova, Y. Y.; Zanforlin, E.; Kokorekin, V. A.; Wu, Y. Y. J.; Fleury, F.; Wong, V. K. W.; Uthaipibull, C.; Terent'ev, A., Synthetic peroxides promote apoptosis of cancer cells inhibiting of P-glycoprotein ABCB5. *ChemMedChem* **2020,** *15* (13), 1118-1127.
5. Zhou, J.; Wakchaure, V.; Kraft, P.; List, B., Primary-amine-catalyzed enantioselective intramolecular aldolizations. *Angew Chem Int Edit* **2008,** *47* (40), 7656-7658.
6. Terent'ev, A. O.; Vil', V. A.; Yaremenko, I. A.; Bityukov, O. V.; Levitsky, D. O.; Chernyshev, V. V.; Nikishin, G. I.; Fleury, F., Preparation of a microsized cerium chloride-based catalyst and its application in the Michael addition of beta-diketones to vinyl ketones. *New J Chem* **2014,** *38* (4), 1493-1502.
7. Yaremenko, I. A.; Syroeshkin, M. A.; Levitsky, D. O.; Fleury, F.; Terent'ev, A. O., Cyclic peroxides as promising anticancer agents: in vitro cytotoxicity study of synthetic ozonides and tetraoxanes on human prostate cancer cell lines. *Med Chem Res* **2017,** *26* (1), 170-179.
8. Terent'ev, A. O.; Yaremenko, I. A.; Chernyshev, V. V.; Dembitsky, V. M.; Nikishin, G. I., Selective Synthesis of Cyclic Peroxides from Triketones and H2O2. *J Org Chem* **2012,** *77* (4), 1833-1842.
9. Yaremenko, I. A.; Belyakova, Y. Y.; Radulov, P. S.; Novikov, R. A.; Medvedev, M. G.; Krivoshchapov, N. V.; Korlyukov, A. A.; Alabugin, I. V.; Terent'ev, A. O., Marriage of Peroxides and Nitrogen Heterocycles: Selective Three-Component Assembly, Peroxide-Preserving Rearrangement, and Stereoelectronic Source of Unusual Stability of Bridged Azaozonides. *J Am Chem Soc* **2021,** *143* (17), 6634-6648.
10. Yaremenko, I. A.; Belyakova, Y. Y.; Radulov, P. S.; Novikov, R. A.; Medvedev, M. G.; Krivoshchapov, N. V.; Alabugin, I. V.; Terent’ev, A. O., Cascade Assembly of Bridged N-Substituted Azaozonides: The Counterintuitive Role of Nitrogen Source Nucleophilicity. *Org. Lett.* **2022,** *24* (36), 6582-6587.
11. Yaremenko, I. A.; Belyakova, Y. Y.; Radulov, P. S.; Novikov, R. A.; Medvedev, M. G.; Krivoshchapov, N. V.; Korlyukov, A. A.; Alabugin, I. V.; Terent′ev, A. O., Inverse α-Effect as the Ariadne’s Thread on the Way to Tricyclic Aminoperoxides: Avoiding Thermodynamic Traps in the Labyrinth of Possibilities. *J Am Chem Soc* **2022,** *144* (16), 7264-7282.
12. Yaremenko, I. A.; Gomes, G. D.; Radulov, P. S.; Belyakova, Y. Y.; Vilikotskiy, A. E.; Vil', V. A.; Korlyukov, A. A.; Nikishin, G. I.; Alabugin, I. V.; Terent'ev, A. O., Ozone-Free Synthesis of Ozonides: Assembling Bicyclic Structures from 1,5-Diketones and Hydrogen Peroxide. *J Org Chem* **2018,** *83* (8), 4402-4426.
